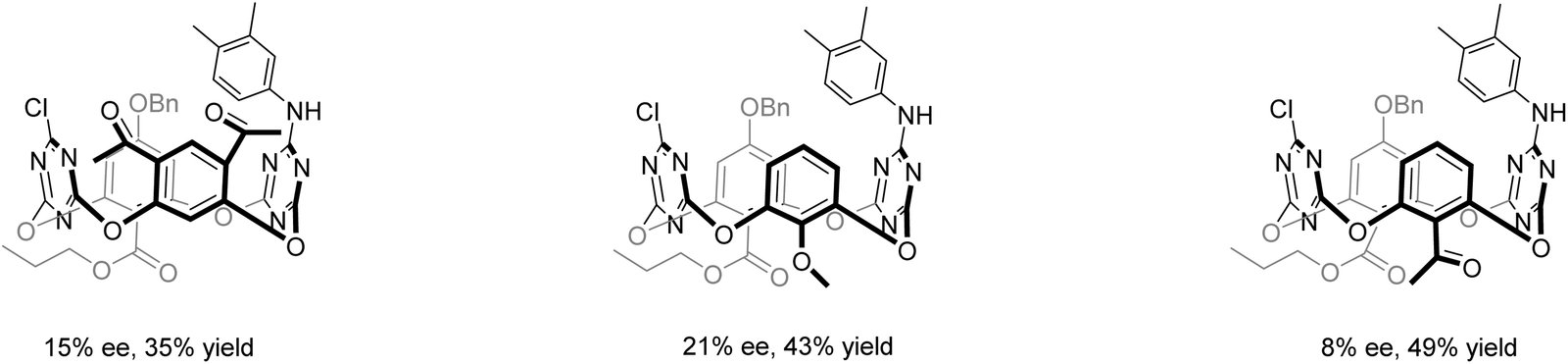# Remote desymmetrization of tetraoxacalix[2]arene[2]triazines enables the asymmetric synthesis of inherently chiral heteracalix[4]aromatics

**DOI:** 10.1039/d6sc03931f

**Published:** 2026-09-09

**Authors:** Zhanpeng Lin, Xinjie Wu, Xinyi Li, Yuanxing Kuang, Zhen Huang, Guofeng Li, Ming Zhang, Liang Hong

**Affiliations:** a Guangdong Key Laboratory of Chiral Molecule and Drug Discovery, School of Pharmaceutical Sciences, Sun Yat-Sen University Guangzhou 510006 China hongliang@sysu.edu.cn; b School of Pharmacy, Shenzhen University Medical School, Shenzhen University Shenzhen 518055 China liguofeng@szu.edu.cn zhangming23@szu.edu.cn

## Abstract

The catalytic enantioselective synthesis of inherently chiral macrocycles remains a major challenge, particularly through non-cyclization strategies. Herein, we report a chiral phosphoric acid (CPA)-catalyzed remote desymmetrization of tetraoxacalix[2]arene[2]triazines, enabling access to inherently chiral heteracalix[4]aromatics. The strategy relies on sequential S_N_Ar aminations, in which the first amination installs an aniline N–H unit as a “control handle” that both creates a hydrogen-bonding recognition site for the catalyst and suppresses macrocyclic ring inversion. In the subsequent CPA-catalyzed step, the monoaminated intermediate undergoes kinetic resolution, allowing asymmetric differentiation of two remote enantiotopic sites within a flexible macrocyclic scaffold. The method affords a broad range of inherently chiral heteracalix[4]aromatics in good yields and high to excellent enantioselectivities (up to 99% ee), and is further applicable to disubstituted products and diverse derivatizations. Mechanistic studies indicate that hydrogen-bond-assisted catalyst recognition and steric inhibition of conformational inversion are both crucial for enantiocontrol. This work establishes a practical asymmetric strategy for inherently chiral macrocycles and expands the scope of remote desymmetrization in flexible supramolecular scaffolds.

## Abstract 2

A remote desymmetrization strategy enables direct access to inherently chiral heteracalix[4]aromatics, where an aniline N–H handle guides CPA catalysis and suppresses ring inversion to achieve stereocontrol in a flexible macrocycle.

## Graphical abstract

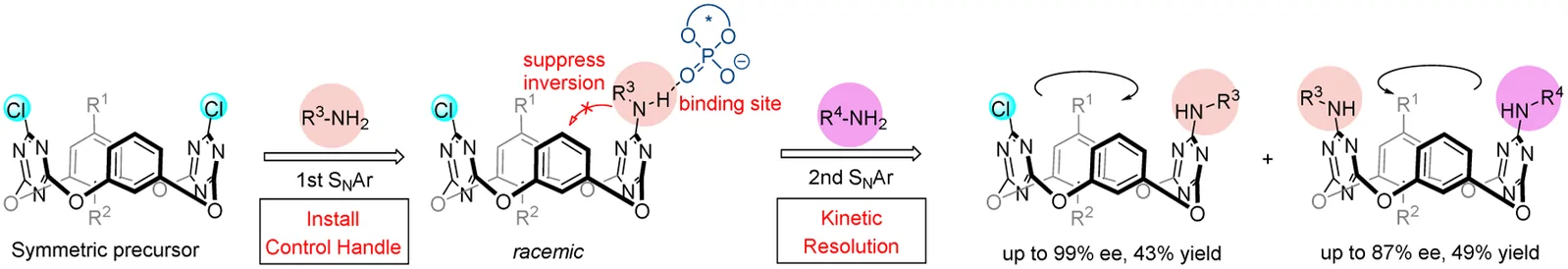

## Introduction

Inherent chirality, introduced by Böhmer in 1994 to describe calixarene frameworks lacking any symmetry elements other than a C1 asymmetry axis (Fig. 1a),^1^ has since emerged as a broader concept for curved chiral macrocycles that do not fit the classical categories of central, axial, planar, or helical chirality. Owing to their unique three-dimensional architectures, inherently chiral calixarenes have attracted considerable interest in chiral recognition,^2–4^ asymmetric catalysis,^5–7^ and chiral functional materials.^8^ Despite this promise, their catalytic enantioselective synthesis remains underdeveloped.^9–11^ A notable breakthrough was reported in 2020 by Wang, who realized the enantioselective synthesis of inherently chiral heteracalix[4]aromatics through intramolecular cyclization of linear precursors.^12–14^ Since then, additional asymmetric approaches to inherently chiral calixarenes have been developed.^15–21^ However, most of these methods rely on cyclization-based strategies to lock chiral conformations by constructing fused or bridged rings (Fig. 1b), whereas non-cyclization approaches remain rare.^22–25^ As a result, general and diverse catalytic routes to inherently chiral macrocycles are still lacking.

**Fig. 1:**
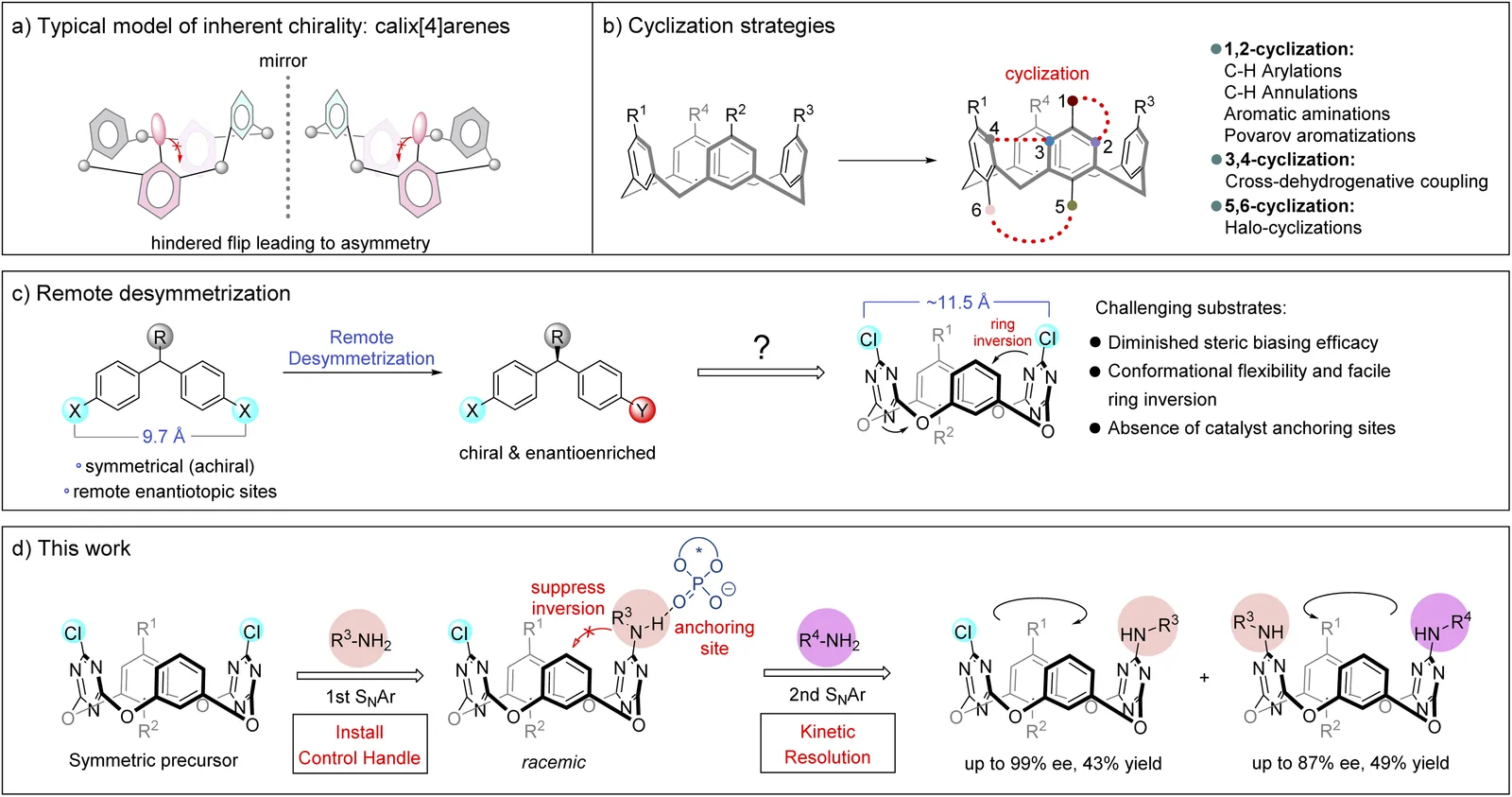
Background and design strategy. (a) Inherently chiral calixarenes. (b) Cyclization-based asymmetric strategy for inherently chiral macrocycles. (c) Challenges in remote desymmetrization of flexible macrocycles. (d) This work: control-handle-enabled CPA-catalyzed remote desymmetrization of tetraoxacalix[2]arene[2]triazines.

Enantioselective desymmetrization offers an efficient strategy for generating optically active molecules from achiral substrates by breaking their symmetry elements.^26–28^ Among such transformations, remote desymmetrization is especially challenging because stereochemical information must be transmitted over a long distance from the catalytic site to the prochiral element.^29–35^ Although important advances have been achieved in diarylmethane-derived systems (Fig. 1c), analogous strategies have not yet been successfully applied to inherently chiral macrocyclic frameworks.

Heteracalix[4]aromatics constitute an important family of supramolecular macrocycles with tunable cavities and rich recognition properties.^36^ We envisaged that dichlorinated tetraoxacalix[2]arene[2]triazines would serve as suitable prochiral substrates for remote desymmetrization because they are readily accessible and bear two symmetrically positioned reactive chlorine atoms.^37–39^ Selective functionalization of one chlorine atom would break the molecular symmetry and induce chirality in the macrocyclic scaffold. However, enantioselective differentiation of two identical reaction sites separated by approximately 11.5 Å on a flexible macrocycle presents several formidable challenges: diminished steric biasing efficacy by a chiral catalyst over large distances, conformational flexibility and facile ring inversion of the macrocycle complicating enantiocontrol, and the absence of built-in binding sites for catalyst recognizing and anchoring (Fig. 1c).

To address these issues, we sought to install a control handle into the macrocyclic framework. This unit was designed to serve a dual role: to create a defined hydrogen-bonding recognition site for a chiral catalyst and to suppress macrocyclic ring inversion. Herein, we report a chiral phosphoric acid (CPA)-catalyzed remote desymmetrization of tetraoxacalix[2]arene[2]triazines through sequential S_N_Ar amination. The first amination installs an aniline N–H unit as the control handle. In the subsequent CPA-catalyzed step, the monoaminated intermediate undergoes kinetic resolution, enabling asymmetric differentiation of two remote enantiotopic sites within a flexible macrocyclic framework and thereby providing direct access to inherently chiral heteracalix[4]aromatics (Fig. 1d).

## Results and discussion

We began our study with tetraoxacalix[2]arene[2]triazine 1a and aniline 2a as model substrates (Table 1). Thiourea catalysts, cinchona alkaloid-derived catalyst, and N-heterocyclic carbenes all proved ineffective, affording the mono-substituted product 3a with poor enantioselectivities. We then turned to chiral phosphoric acids, anticipating that the catalyst could engage the installed aniline N–H group through hydrogen bonding. Gratifyingly, H_8_-BINOL-derived CPA-3 delivered 3a in 44% ee (entry 3), supporting the feasibility of this design. Further catalyst optimization identified CPA-5 as superior, providing 3a in 94% ee, albeit in modest yield (entry 5). Variation of solvent, catalyst loading, and temperature did not improve both yield and enantioselectivity simultaneously (entries 6–10). Replacing K_2_CO_3_ with KHCO_3_ increased the yield of 3a but significantly eroded the ee (entry 11). We reasoned that the balance between formation of mono-substituted 3a and overreaction to disubstituted 4a was governed by the relative rates of the second substitution step. Indeed, a mixed-base system enabled finer control of reactivity. A 2 : 3 mixture of KHCO_3_/K_2_CO_3_ proved optimal, affording 3a in 42% yield and 95% ee together with 4a in 55% yield (entry 13).

**Table 1:** Optimization of reaction conditions^a^

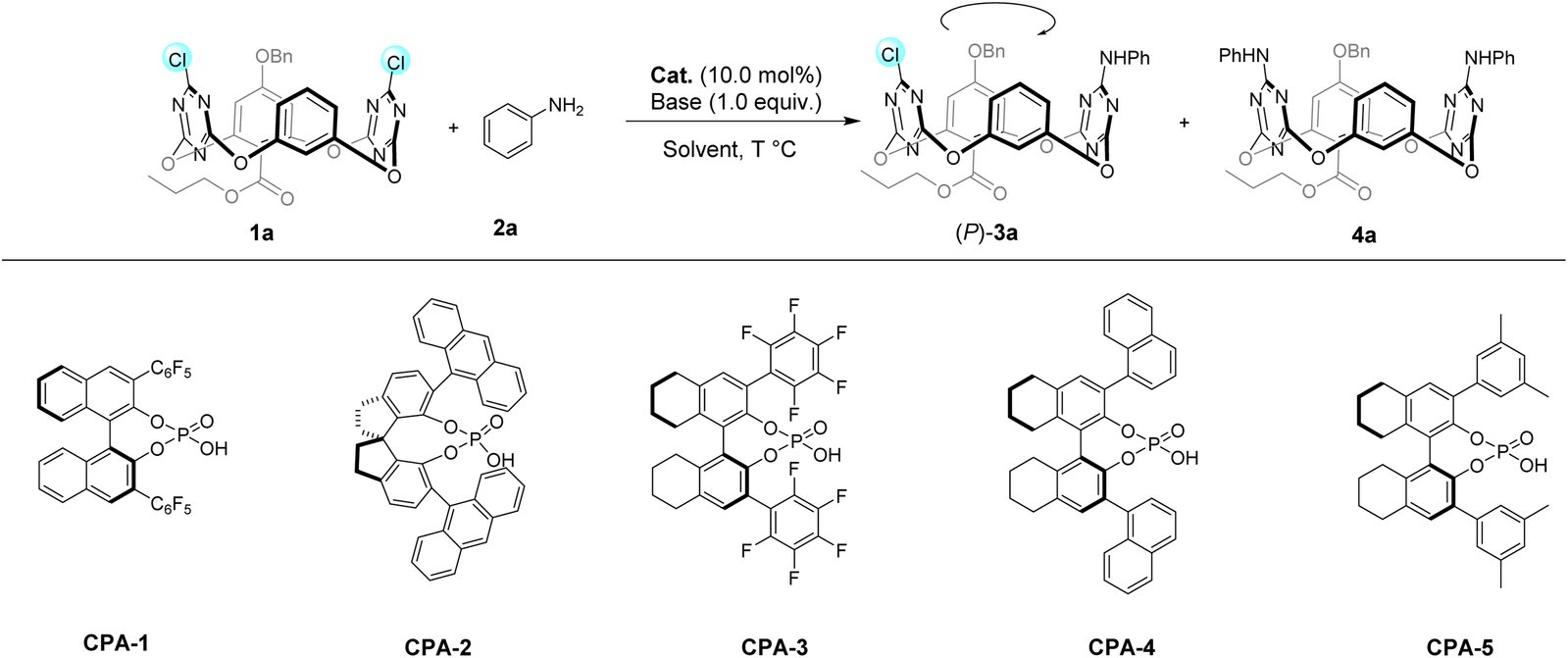						
Entry	Cat. (10 mol%)	Solvent	Base	4a, yield (%)	3a, yield (%)	3a, ee (%)
1	CPA-1	Tol	K_2_CO_3_	23	44	0
2	CPA-2	Tol	K_2_CO_3_	26	67	29
3	CPA-3	Tol	K_2_CO_3_	22	69	44
4	CPA-4	Tol	K_2_CO_3_	59	35	72
5	CPA-5	Tol	K_2_CO_3_	60	28	94
6	CPA-5	THF	K_2_CO_3_	30	69	15
7	CPA-5	MeCN	K_2_CO_3_	27	64	15
8	CPA-5	CH_3_OH	K_2_CO_3_	80	6	7
9^b^	CPA-5	Tol	K_2_CO_3_	50	49	20
10^c^	CPA-5	Tol	K_2_CO_3_	69	28	81
11	CPA-5	Tol	KHCO_3_	50	46	52
12	CPA-5	Tol	KHCO_3_ : K_2_CO_3_ = 1 : 1	48	36	93
13	CPA-5	Tol	KHCO_3_ : K_2_CO_3_ = 2 : 3	49	42	95

a Reaction conditions: unless otherwise noted, the reaction was performed with 1a (0.05 mmol), 2a (1.35 equiv., 0.068 mmol), catalyst (10 mol%, 0.005 mmol) and base (1.0 equiv., 0.05 mmol) in solvent (2.0 mL) at room temperature. Isolated yields were given. The ee values were determined by chiral HPLC analysis.

b 0 °C.

c 20 mol% of CPA-5.

With the optimized conditions established, we examined the amine scope (Scheme 1a). *Para*-substituted anilines bearing electron-donating groups furnished products 3b–3e with good to excellent enantioselectivities (83–98% ee). Electron-withdrawing substituents (3f–3j) gave slightly lower yields, presumably due to decreased nucleophilicity of the aniline nitrogen, while maintaining high to excellent enantioselectivities (80–99% ee). *Meta*-substituted and *meta*/*para*-disubstituted anilines were likewise compatible, affording 3k–3r with 88% to 99% ee. Aryl amines containing extended aromatic systems or heteroaromatic rings also reacted smoothly, delivering 3s–3x with good to excellent enantioselectivities (83–99% ee). These results demonstrate broad tolerance toward structurally diverse aniline partners.

**Scheme 1:**
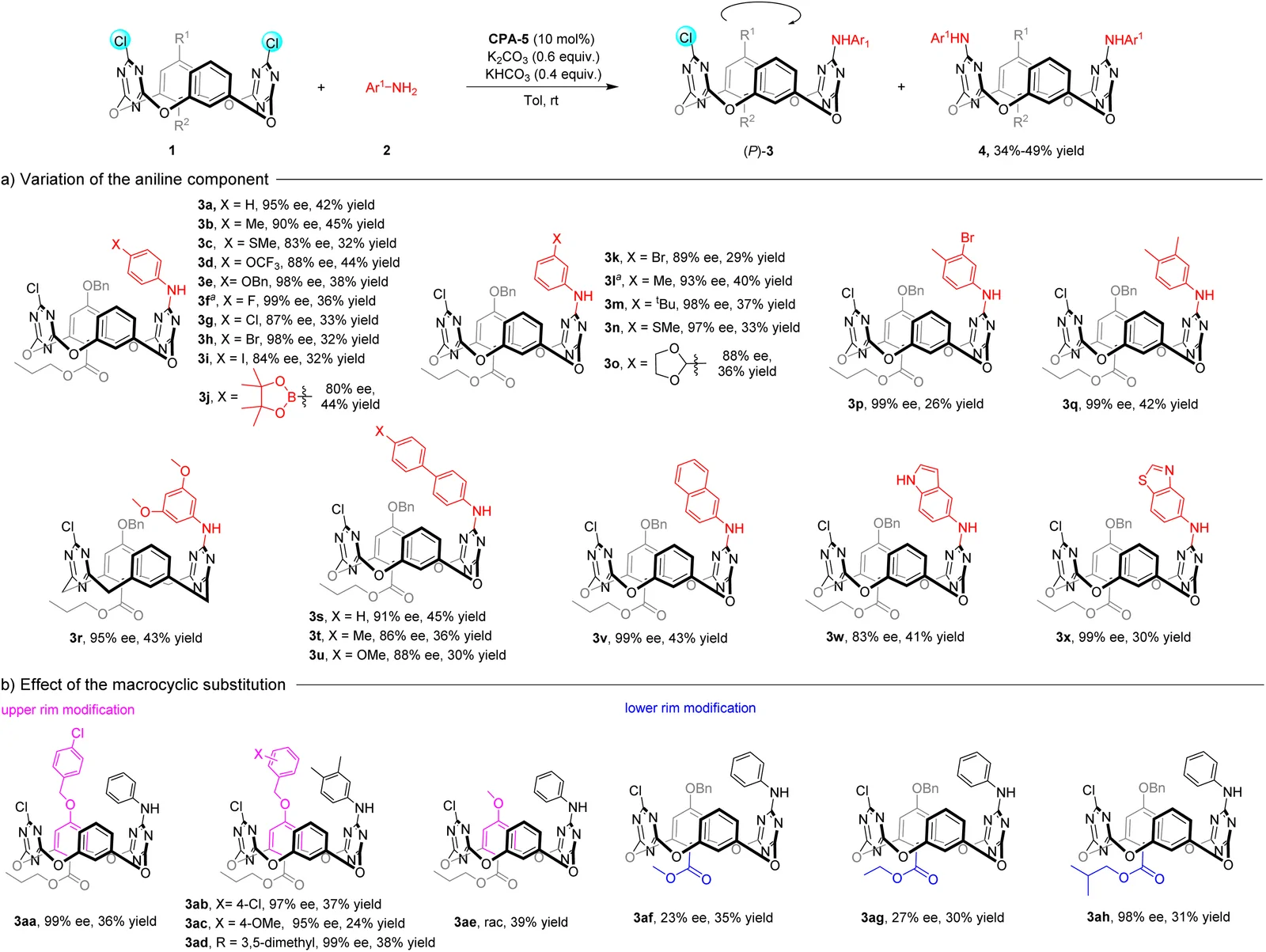
Scope of the remote desymmetrization. (a) Variation of the aniline component. (b) Effect of the macrocyclic substitution. Reaction conditions: 1 (0.05 mmol), 2 (1.35 equiv., 0.068 mmol), CPA-5 (10 mol%, 0.005 mmol), K_2_CO_3_ (0.6 equiv., 0.03 mmol), KHCO_3_ (0.4 equiv., 0.02 mmol) in toluene (2.0 mL) at room temperature for 10 h. ^*a*^m-Xylene was used instead of toluene and the amine was added dropwise.

We next evaluated the effect of structural modification on the macrocyclic scaffold (Scheme 1b). Alteration of the upper-rim substituents revealed a pronounced steric effect. Methylation of the phenolic oxygen gave racemic 3ae, presumably because the small substituent was insufficient to suppress macrocyclic inversion. By contrast, bulkier upper-rim substituents furnished the corresponding products with excellent enantioselectivities (3aa–3ad, 95–99% ee). A similar trend was observed at the lower rim. Substrates bearing methyl or ethyl ester groups gave 3af and 3ag with poor ee values (23% and 27%, respectively), whereas a more sterically demanding isobutyl ester afforded 3ah in 98% ee. These results support the view that the weakly substituted parent tetraoxa-calix[2]arene[2]triazine framework is intrinsically flexible. Efficient long-range stereocontrol is achieved only when sufficiently bulky substituents jointly restrict ring inversion.

Finally, we investigated the effect of different substituents on the benzene ring on this reaction. Unfortunately, all substrates gave the desired products with only low enantiomeric excess, and the underlying reasons for this outcome remain unclear.^40^

To demonstrate the versatility of the method, we subjected racemic mono-substituted rac-3 to a second aminative substitution with various anilines (Scheme 2). This strategy enabled the stereoselective synthesis of disubstituted inherently chiral heteracalix[4]aromatics (M)-4 from a common intermediate. For example, treatment of rac-3a with 3-(*tert*-butyl)aniline gave disubstituted product 4b in 48% yield and 84% ee, while the unreacted 3a was recovered in 49% yield and 86% ee. Starting from rac-3a and various anilines, a series of diamine-substituted products 4c–4h was obtained with 74–87% ee, and the recovered monoaniline 3a was obtained with good enantiopurities. Other mono-substituted substrates also underwent the transformation successfully to furnish 4i–4o with similarly useful selectivity.

**Scheme 2:**
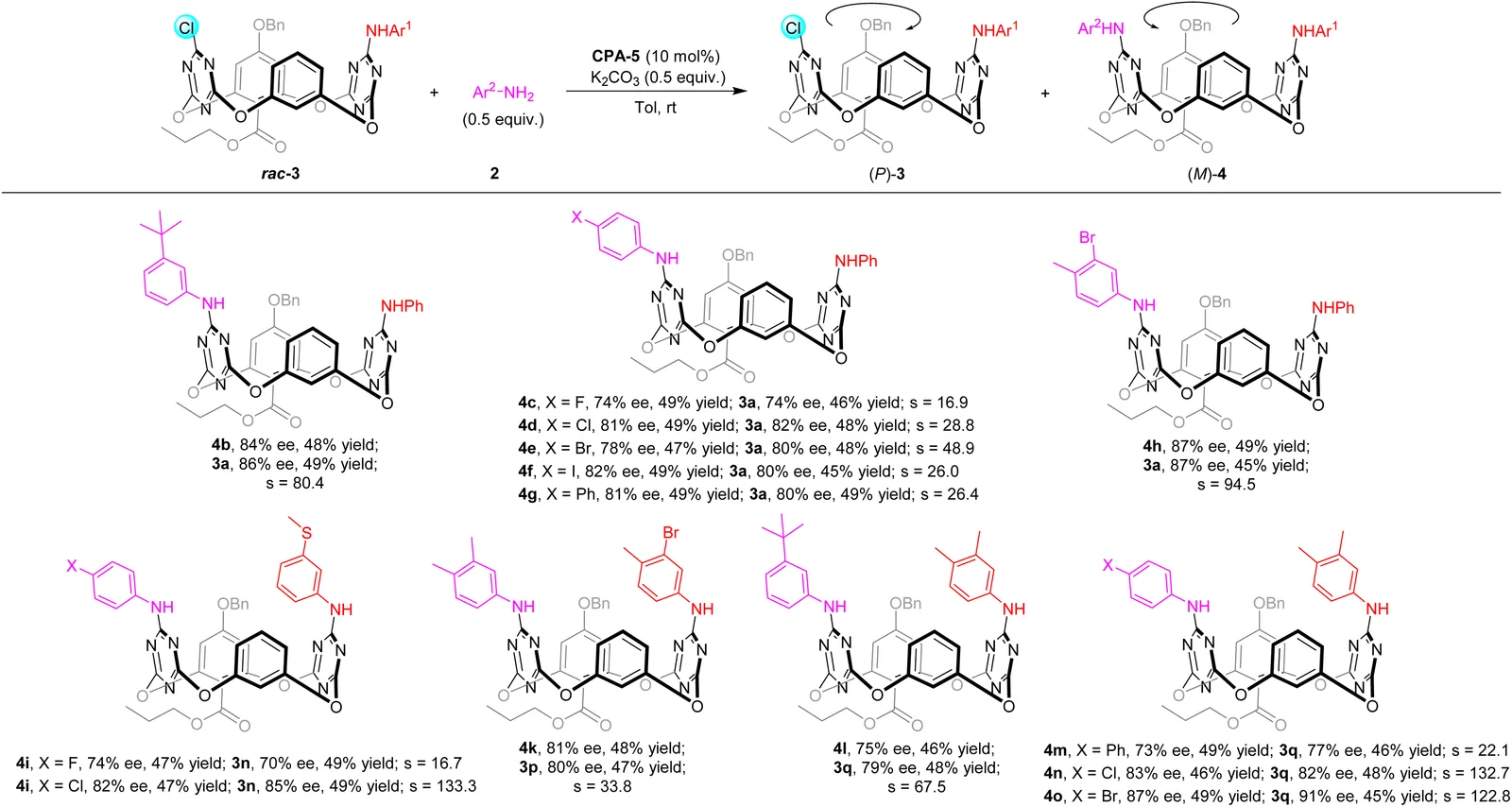
Scope of disubstituted inherently chiral heteracalix[4]aromatics^*a*^. ^*a*^Reaction conditions: rac-3 (0.05 mmol), 2 (0.5 equiv., 0.025 mmol), CPA-5 (10 mol%, 0.005 mmol) and K_2_CO_3_ (0.5 equiv., 0.025 mmol) in toluene (2.0 mL) at room temperature for 2 h.

The synthetic utility of the method was demonstrated by a 0.5 mmol scale-up of the model reaction, which furnished 3a in 35% yield and 94% ee. The optical purity of 3a could be further improved to 99% ee by a simple recrystallization (Scheme 3a). We next explored derivatization of 3a (Scheme 3b). Suzuki–Miyaura coupling with phenylboronic acid gave 5a in 66% yield with complete retention of ee. Additional substitution reactions with amino acids, hydroquinine, phenol, and thiophenol provided structurally diverse derivatives that generally maintained high optical purity. The diminished ee observed for 5e is likely attributable to partial racemization under the elevated reaction temperature required for this specific transformation. These transformations underscore the utility of the inherently chiral heteracalixarene scaffold as a versatile chiral platform. We also examined the chiroptical properties of enantiopure 3n (Scheme 3c). The CD spectra of the two enantiomers showed mirror-image profiles, with characteristic Cotton effects at 218 and 249 nm. Circularly polarized luminescence measurements revealed emission maxima at 380 and 423 nm and a maximum luminescence dissymmetry factor of |*g*_lum_| = 1.4 × 10^−3^, indicating promising chiroptical activity.

**Scheme 3:**
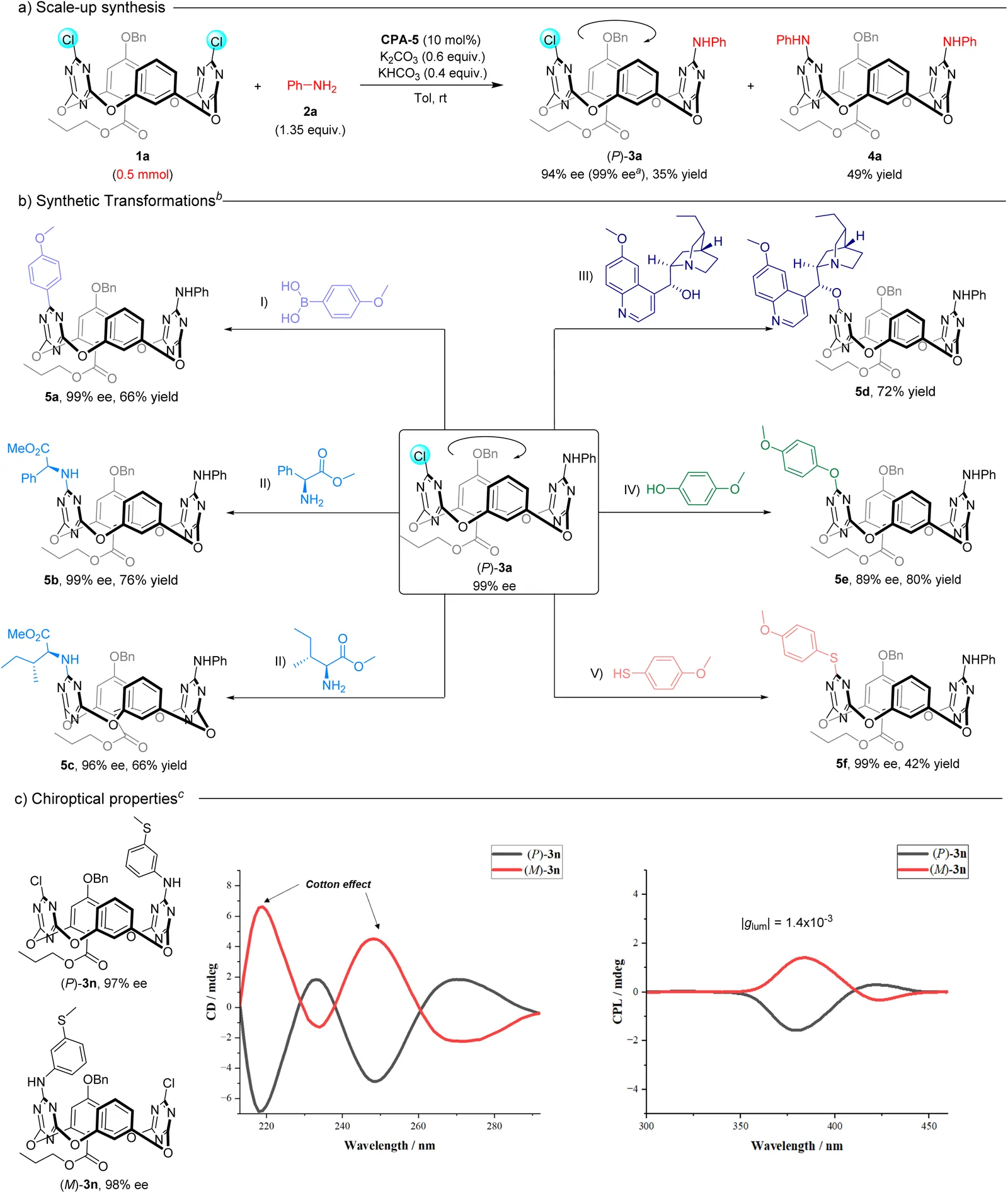
Synthetic utility and chiroptical properties. (a) After recrystallization. (b) Detailed experimental procedures and conditions are provided in the SI. (c) CD (left, 7 × 10^−5^ M) and CPL (right, 1 × 10^−2^ M, *λ*_ex_ = 375 nm) of product 3n in CH_3_CN.

### Mechanistic studies

To gain mechanistic insight, we carried out some control experiments (Scheme 4a). Quenching the model reaction after 5 min gave only rac-3a, indicating that the initial amination proceeds without enantioselection. In contrast, subjecting rac-3a to the standard conditions in the presence of 0.5 equiv of aniline 2a afforded the resolved (P)-3a in 36% yield and 96% ee together with disubstituted 4a in 49% yield, clearly establishing that the second substitution step operates as a kinetic resolution process. The importance of hydrogen bonding was demonstrated by the *N*-methylated analogue of rac-6a, which gave a racemic product under otherwise identical conditions. Kinetic analysis of the two enantiomers further showed that the (M)-enantiomer was consumed more rapidly than the (P)-enantiomer (*k*_M_*k*_P_^−1^ = 7.32), directly accounting for the observed enantiomeric enrichment (Scheme 4b). Rotational barrier calculation revealed a clear correlation between steric bulk and configurational stability, supporting the role of steric hindrance in suppressing macrocyclic ring inversion (Scheme 4c).

**Scheme 4:**
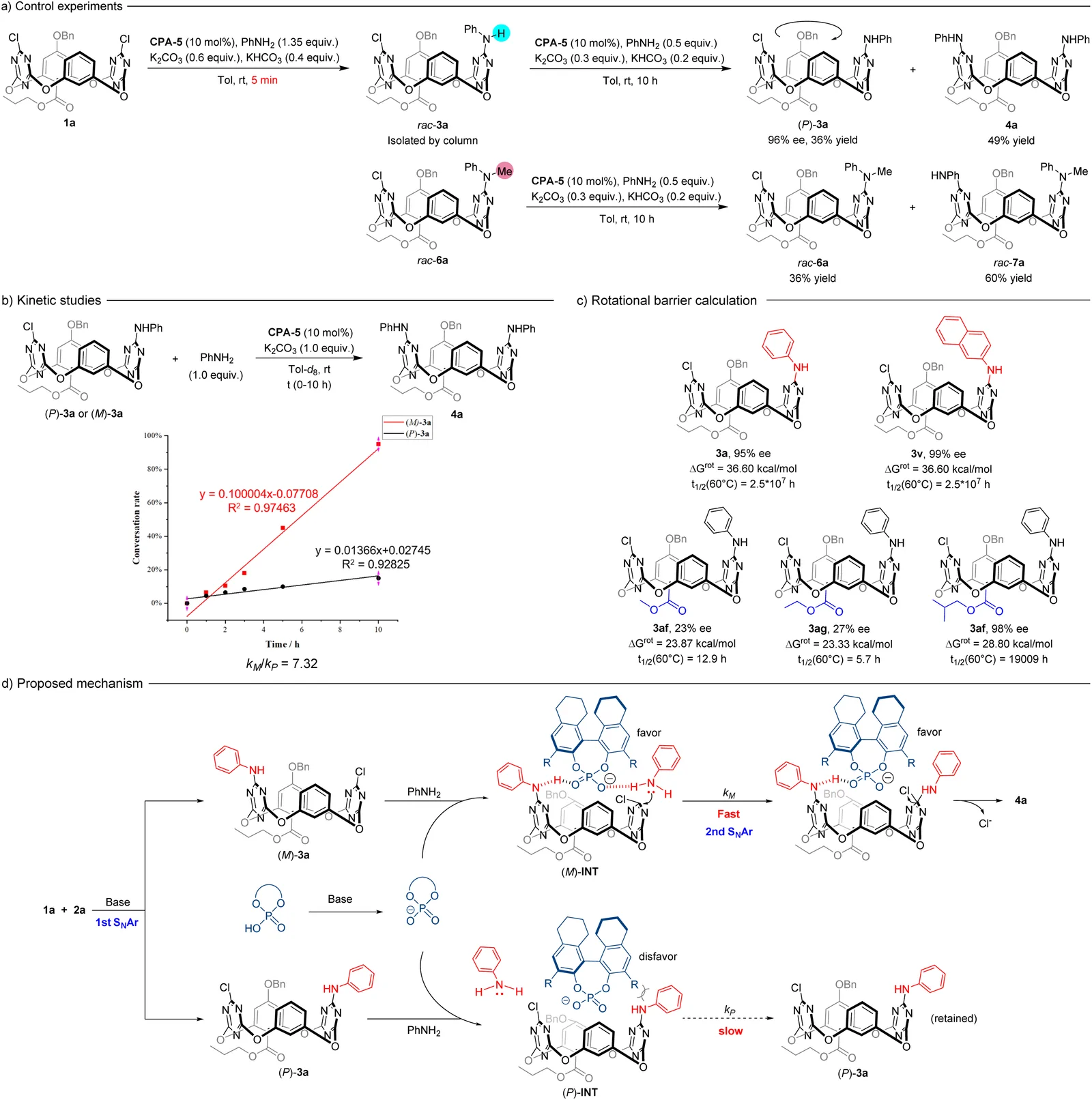
Mechanistic studies. (a) Evidence for kinetic resolution and role of hydrogen bonding. (b) Kinetic profiles of the two enantiomers. (c) Relationship between steric bulk, rotational barrier, and enantiopurity. (d) Proposed mechanism for CPA-catalyzed kinetic resolution.

On the basis of these observations, we propose the mechanism shown in Scheme 4d. The prochiral substrate 1a first undergoes nonselective S_N_Ar amination with aniline 2a to form rac-3a. Under CPA catalysis, one enantiomer of 3a engages more favorably with the chiral phosphate catalyst through hydrogen bonding and a matched steric environment, leading to more rapid activation and the second amination to form the disubstituted product 4a. The mismatched enantiomer reacts more slowly and is therefore retained as the mono-substituted product. The observed enantioselectivity thus arises from hydrogen-bond-assisted kinetic resolution within a conformationally biased macrocyclic framework.

## Conclusions

In conclusion, we have developed a remote desymmetrization strategy that directly affords inherently chiral heteracalix[4]aromatics. Installation of an aniline N–H control handle enables hydrogen-bond-assisted CPA-catalyzed kinetic resolution while suppressing macrocyclic ring inversion, thereby solving remote stereocontrol in a flexible macrocyclic framework. This dual role enables the subsequent substitution to proceed as an efficient kinetic resolution of monoaminated tetraoxacalix[2]arene[2]triazines, affording inherently chiral heteracalix[4]aromatics with high to excellent enantioselectivities, up to 99% ee.

## Author contributions

Z. L , X. W. and X. L. performed the experiments. All authors contributed to analyzing the experimental results. L. H. supervised the project and wrote the paper.

## Conflicts of interest

The authors declare no competing financial interest.

## Supplementary Material

SC-017-D6SC03931F-s001

## Data Availability

The authors declare that the data supporting the findings of this study are available within the article and the supplementary information (SI) as well as from the authors upon request. Supplementary information: experimental procedures, experimental equipment, synthetic applications, characterization data, computational results, HRMS, HPLC and NMR spectra for all new compounds. See DOI: https://doi.org/10.1039/d6sc03931f.
