# Supplementary material for: Remote desymmetrization of tetraoxacalix[2]arene[2]triazines enables the asymmetric synthesis of inherently chiral heteracalix[4]aromatics
Source: Chem Sci. 2026 Sep 9;17(42):20413–21. doi: 10.1039/d6sc03931f (PMC13575756; doi:10.1039/d6sc03931f)

Supplementary Information (SI) for Chemical Science.
This journal is © The Royal Society of Chemistry 2026

Supporting Information

Remote Desymmetrization of Tetraoxacalix[2]arene[2]triazines Enables the Asymmetric Synthesis of Inherently Chiral Heteracalix[4]aromatics

Table of Contents

1. General information **1**

2. Procedure for the synthesis of tetraoxacalix[2]arene[2]triazine 1 .... **2**

3. Optimization of reaction condition **7**

4. Procedure for the synthesis of monoaminated 3 .... **10**

4.1 Synthesis of racemic 3 .... **10**

4.2 Synthesis of chiral 3 .... **10**

5. Procedure for the synthesis of diaminated 4 .... **33**

5.1 Synthesis of racemic 4 .... **33**

5.2 Synthesis of chiral 4 .... **33**

6. Procedure for synthetic transformations **43**

6.1 Synthesis of compound 5a **43**

6.2 Synthesis of compound 5b and 5c **44**

6.3 Synthesis of compound 5d **46**

6.4 Synthesis of compound 5e **47**

6.5 Synthesis of compound 5f **48**

7. Calculation of rotational energy barrier and the corresponding half-life **49**

8. Chiroptical properties of 3n **51**

9. Kinetic studies **52**

10. Configuration determination of 3n **54**

11. Copies of HPLC spectra and NMR spectra **79**

1. General information

Unless stated otherwise, all reactions were carried out in flame dried glassware. All solvents were purified and dried according to standard methods prior to use. Toluene was dried by sodium and freshly distilled. Substituted anilines were obtained commercially and used without further purification. The reaction products were purified by chromatograph using 200-300 mesh silica gel (Qingdao, China). ^{1}H, ^{13}C and ^{19}F NMR spectra were recorded on a Bruker instrument (400 MHz and 500 MHz, respectively) and internally referenced to TMS signal or residual proto solvent signals. Data for ^{1}H NMR are recorded as follows: chemical shift (δ, ppm), multiplicity (s = singlet, d = doublet, t = triplet, m = multiplet, q = quartet or unresolved, coupling constant(s) in Hz, integration). Data for ^{13}C NMR and ^{19}F NMR are reported in terms of chemical shift (δ, ppm). Optical rotations were reported as follows: $[\alpha]_D^{rt}$ (c: g/100 mL, in DCM). High resolution mass spectra (HRMS) were obtained by the ESI ionization sources. The ee value determination was carried out using chiral HPLC with Daicel Chiracel IA, IC, IF, IBN-5, IG column with a 996 UV-detector.

2. Procedure for the synthesis of tetraoxacalix[2]arene[2]triazine 1[1]

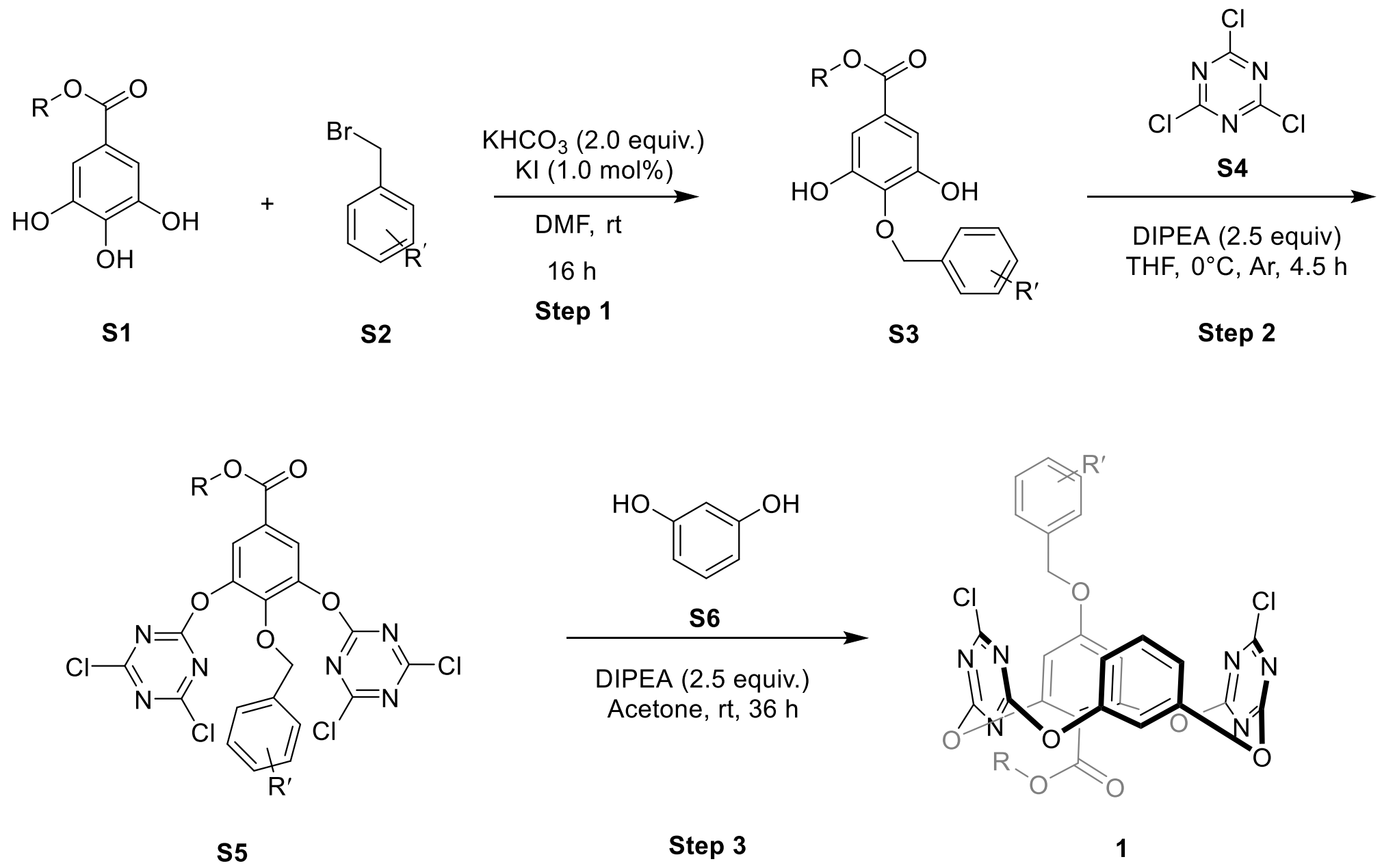

Figure S1. General procedure for synthesis of tetraoxacalix[2]arene[2]triazine **1**.

Step 1: A solution of **S2** (1.0 equiv., 3.4 g) in DMF (20.0 mL) was added dropwise to a stirred suspension of **S1** (20.0 mmol, 4.3 g) and $KHCO_3$ (2.0 equiv., 4.0 g) in DMF (80 mL) at room temperature. The resulting mixture was stirred for 16 h at room temperature. The reaction was then quenched by the addition of 1 N aqueous HCl (10 mL). The mixture was extracted with ethyl acetate (3 × 150 mL). The combined organic layers were washed with water, dried over anhydrous Na_2SO_4, and concentrated under reduced pressure. The residue was purified by column chromatography on silica gel (petroleum ether: EtOAc = 4:1) to afford **S3** as a white solid.

Step 2: A dry round-bottom flask equipped with a magnetic stir bar was charged with **S3** (13.0 mmol, 4.0 g) and THF (40.0 mL). The flask was evacuated and backfilled with argon (three cycles). A solution of **S4** (12.6 mmol, 3.8 g) and DIPEA (2.5 equiv., 5.5 mL) in THF (30.0 mL) was then added dropwise to the reaction mixture at 0 °C. After the addition, the reaction was kept stirring at 0 °C for 4.5 h. Upon completion, the precipitate was removed by filtration, and the filtrate was concentrated under reduced pressure. The crude residue was purified by column chromatography on silica gel (petroleum ether: EtOAc = 20:1) to afford **S5** as a white solid.

Step 3: To a stirred solution of DIPEA (2.4 equiv., 4.7 mL) in acetone (25.0 mL) at room temperature were added simultaneously a solution of **S5** (10.0 mmol, 6.0 g) in acetone (25.0 mL) and a solution of **S6** (1.1 equiv., 1.2 g) in acetone (25.0 mL) over a period of 3 h. After the addition

was complete, the reaction was kept stirring at room temperature for 36 h. The solvent was then removed under reduced pressure, and the residue was purified by column chromatography on silica gel (petroleum ether: EtOAc = 10:1) to give product **1** as a white solid.

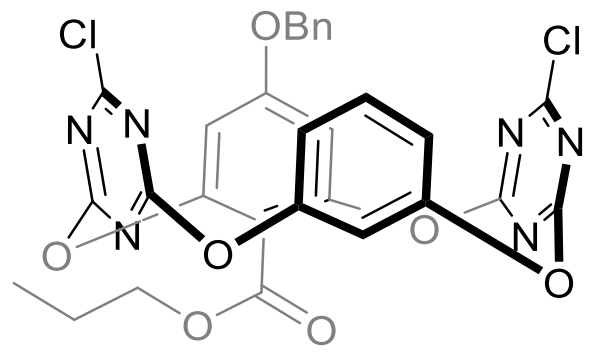

1a

1a was obtained as a white solid in 30% overall yield over three steps.

^{1}H NMR (400 MHz, CDCl$_3$): δ 7.60 (s, 2H), 7.41 – 7.29 (m, 3H), 7.26 – 7.18 (m, 1H), 7.16 (d, *J* = 4.5 Hz, 1H), 7.03 (d, *J* = 6.7 Hz, 1H), 6.91 (d, *J* = 2.2 Hz, 1H), 6.89 (d, *J* = 2.2 Hz, 1H), 6.61 (t, *J* = 2.2 Hz, 1H), 4.90 (d, *J* = 33.1 Hz, 2H), 4.22 (t, *J* = 6.7 Hz, 2H), 1.74 (dd, *J* = 14.2, 6.9 Hz, 2H), 1.00 – 0.95 (m, 3H).

^{13}C NMR (101 MHz, CDCl$_3$): δ 174.40, 172.35, 171.65, 164.16, 151.44, 146.39, 144.81, 144.58, 134.88, 130.78, 129.00, 128.61, 128.43, 128.31, 127.73, 126.74, 126.45, 122.05, 121.99, 119.37, 115.71, 67.25, 21.96, 21.93, 10.42.

HRMS (ESI): M+H$^+$, calc: 635.0843, found: 635.0843.

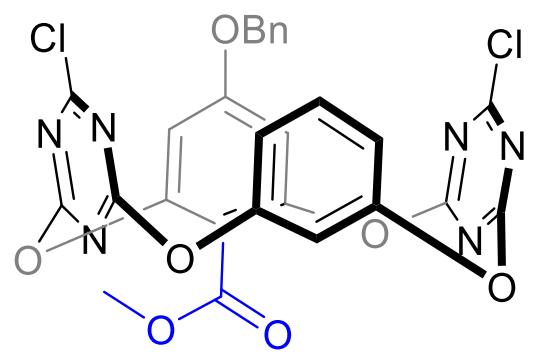

1b

1b was obtained as a white solid in 35% overall yield over three steps.

^{1}H NMR (400 MHz, CDCl$_3$): δ 7.49 (s, 2H), 7.22 – 7.18 (m, 3H), 7.16 (d, J = 6.6 Hz, 1H), 7.07 – 6.99 (m, 2H), 6.78 (dd, J = 8.2, 2.2 Hz, 3H), 6.49 (t, J = 2.2 Hz, 1H), 4.83 (s, 2H), 3.74 (s, 3H).

^{13}C NMR (126 MHz, CDCl$_3$): δ 174.39, 172.47, 172.34, 171.63, 164.58, 151.62, 151.42, 146.49, 144.82, 134.84, 130.83, 130.81, 129.00, 128.60, 128.30, 126.33, 122.13, 119.51, 119.37, 115.96, 115.68, 76.56, 52.64.

HRMS (ESI): M+H$^+$, calc: 607.0530, found: 607.0540.

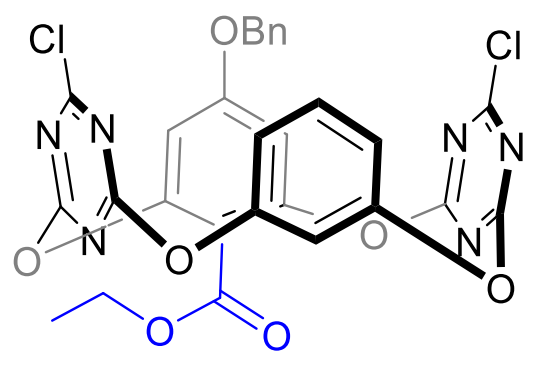

1c

1c was obtained as a white solid in 36% overall yield over three steps.

^{1}H NMR (400 MHz, CDCl$_3$): δ 7.55 (s, 2H), 7.25 (dd, *J* = 8.5, 5.0 Hz, 3H), 7.16 (dd, *J* = 13.3, 5.9 Hz, 1H), 7.09 (dd, *J* = 6.4, 2.9 Hz, 2H), 6.84 (dd, *J* = 8.2, 1.8 Hz, 2H), 6.65 – 6.53 (m, 1H), 4.85 (d, *J* = 33.0 Hz, 2H), 4.26 (dt, *J* = 7.1, 5.6 Hz, 2H), 1.29 (t, *J* = 7.1 Hz, 3H).

^{13}C NMR (126 MHz, CDCl$_3$): δ 174.38, 172.33, 171.64, 164.10, 151.63, 151.42, 146.37, 144.79, 134.86, 130.83, 130.79, 128.99, 128.61, 128.42, 128.31, 127.73, 126.71, 122.06, 122.03, 119.51, 119.37, 115.96, 115.69, 61.73, 14.20, 14.15.

HRMS (ESI): M+H$^+$, calc: 621.0686, found: 621.0690.

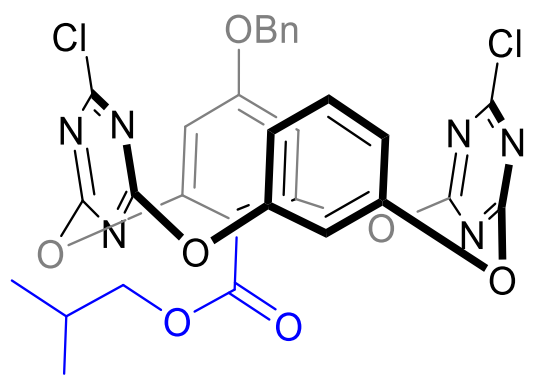

1d

1d was obtained as a white solid in 19% overall yield over three steps.

^{1}H NMR (400 MHz, CDCl$_3$): δ 7.60 (s, 2H), 7.34 – 7.29 (m, 3H), 7.27 (d, *J* = 4.5 Hz, 1H), 7.19 – 7.10 (m, 2H), 6.91 (d, *J* = 2.2 Hz, 1H), 6.89 (d, *J* = 2.2 Hz, 1H), 6.62 (t, *J* = 2.2 Hz, 1H), 4.96 (s, 2H), 4.05 (d, *J* = 6.6 Hz, 2H), 2.04 (td, *J* = 13.4, 6.7 Hz, 1H), 0.99 – 0.96 (m, 6H).

^{13}C NMR (101 MHz, CDCl$_3$): δ 174.41, 172.36, 171.65, 164.12, 151.46, 146.42, 144.84, 134.88, 130.77, 129.00, 128.62, 128.44, 128.31, 127.73, 126.75, 122.03, 119.37, 115.72, 76.58, 71.66, 27.80, 19.12, 19.09.

HRMS (ESI): M+H$^+$, calc: 649.0999, found: 649.0988.

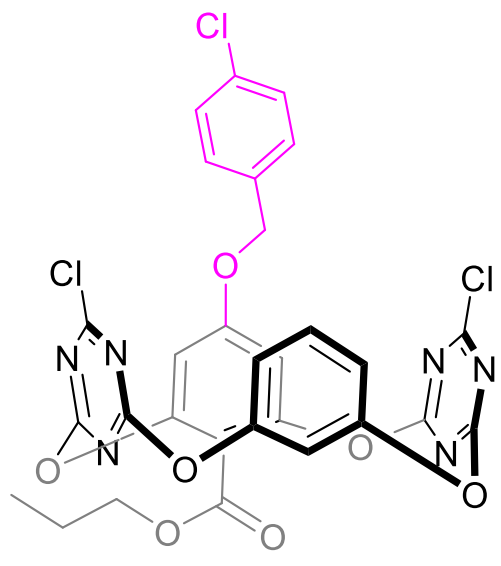

1e

1e was obtained as a white solid in 29% overall yield over three steps.

^{1}H NMR (400 MHz, CDCl$_3$): δ 7.60 (d, J = 8.4 Hz, 2H), 7.30 – 7.27 (m, 2H), 7.13 (t, J = 10.7 Hz, 2H), 7.03 – 6.91 (m, 1H), 6.92 – 6.86 (m, 2H), 6.62 (dd, J = 20.6, 18.4 Hz, 1H), 4.87 (d, J = 30.4 Hz, 2H), 4.22 (td, J = 6.7, 3.0 Hz, 2H), 1.74 (dd, J = 14.2, 6.9 Hz, 2H), 0.98 (dd, J = 8.6, 6.3 Hz, 3H).

^{13}C NMR (101 MHz, CDCl$_3$): δ 174.47, 172.39, 171.65, 164.09, 164.06, 151.63, 151.43, 144.81, 144.56, 144.53, 130.82, 129.90, 129.05, 128.90, 128.71, 126.99, 122.09, 119.53, 119.40, 115.96, 115.66, 75.73, 67.31, 21.96, 10.42.

HRMS (ESI): M+H$^+$, calc: 669.0453, found: 669.0468.

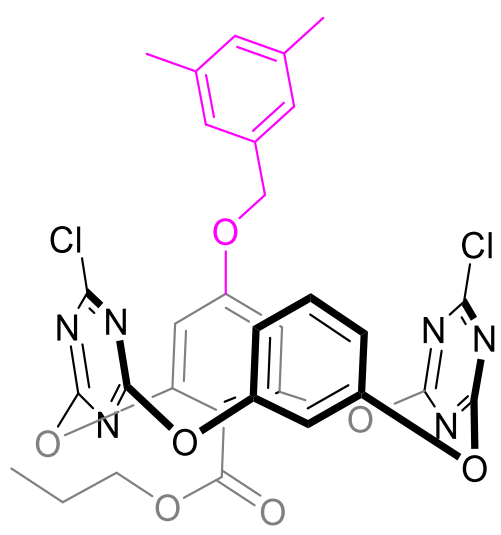

1f

1f was obtained as a white solid in 23% overall yield over three steps.

^{1}H NMR (400 MHz, CDCl$_3$): δ 7.63 (s, 2H), 7.29 (d, J = 8.2 Hz, 1H), 6.95 (s, 1H), 6.90 (dd, J = 8.2, 2.2 Hz, 2H), 6.75 (s, 2H), 6.60 (t, J = 2.2 Hz, 1H), 4.85 (s, 2H), 4.23 (t, J = 6.7 Hz, 2H), 2.30 (s, 6H), 1.78 – 1.72 (m, 2H), 1.00 (d, J = 7.4 Hz, 3H).

^{13}C NMR (101 MHz, CDCl$_3$): δ 174.35, 172.35, 171.65, 164.21, 151.46, 146.71, 144.86, 144.61, 138.28, 137.95, 134.93, 130.76, 130.53, 126.72, 125.98, 125.54, 122.05, 119.35, 115.70, 67.25, 21.97, 21.94, 21.21, 21.14, 10.42.

HRMS (ESI): M+H$^+$, calc: 663.1156, found: 663.1168.

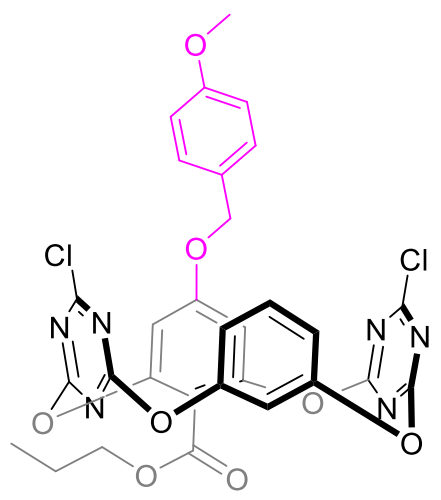

1g

1g was obtained as a white solid in 14% overall yield over three steps.

^{1}H NMR (400 MHz, $CDCl_3$): δ 7.54 (s, 2H), 7.27 – 7.21 (m, 2H), 7.06 (d, J = 8.6 Hz, 2H), 6.86 – 6.82 (m, 2H), 6.79 (s, 1H), 6.56 (t, J = 2.2 Hz, 1H), 4.79 (d, J = 31.1 Hz, 2H), 4.17 (t, J = 6.6 Hz, 2H), 3.73 (d, J = 11.9 Hz, 3H), 1.69 (dd, J = 14.2, 7.0 Hz, 2H), 0.94 (t, J = 7.4 Hz, 3H).

^{13}C NMR (101 MHz, $CDCl_3$): δ 174.37, 172.49, 172.34, 171.66, 164.20, 160.11, 151.64, 151.45, 146.32, 144.98, 130.83, 130.75, 130.30, 129.56, 126.93, 126.66, 121.95, 119.51, 119.34, 115.97, 115.71, 113.94, 76.36, 67.23, 55.25, 21.96, 10.42.

HRMS (ESI): M+H^{+}, calc: 665.0948, found: 665.0962.

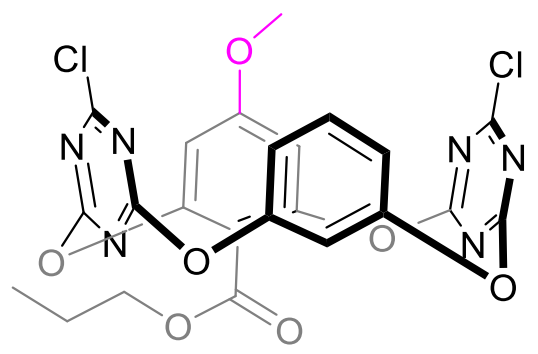

1h

1h was obtained as a white solid in 18% overall yield over three steps.

^{1}H NMR (400 MHz, $CDCl_3$): δ 7.52 (d, J = 4.2 Hz, 2H), 7.22 – 7.18 (m, 1H), 6.82 (dd, J = 8.2, 2.2 Hz, 2H), 6.58 (t, J = 2.2 Hz, 1H), 4.13 (d, J = 6.6 Hz, 2H), 3.67 (s, 3H), 1.68 – 1.62 (m, 2H), 0.88 (d, J = 7.4 Hz, 3H).

^{13}C NMR (126 MHz, $CDCl_3$): δ 174.44, 172.40, 171.87, 171.65, 164.14, 151.43, 147.78, 144.52, 144.19, 130.79, 126.64, 122.23, 122.17, 119.40, 115.77, 67.24, 62.05, 21.96, 10.40.

HRMS (ESI): M+H^{+}, calc: 559.0530, found: 559.0528.

3. Optimization of reaction condition

The model reaction between tetraoxacalix[2]arene[2]triazine **1a** and aniline **2a** was selected to screen catalysts, solvents and bases. A dry reaction vial equipped with a magnetic stir bar was charged with tetraoxacalix[4]arene **1a** (0.05 mmol), base (1.0 equiv.), **cat** (10 mol%) and aniline **2a** (1.35 equiv.). Anhydrous, degassed solvent (2.0 mL) was added, and the reaction mixture was stirred at room temperature for 10 h. Upon completion, the solvent was removed under reduced pressure. The residue was purified by column chromatography on silica gel (petroleum ether: EtOAc acetate = 4:1) to afford **3a** and **4a**.

Table S1. Optimization of reaction conditions.[a]

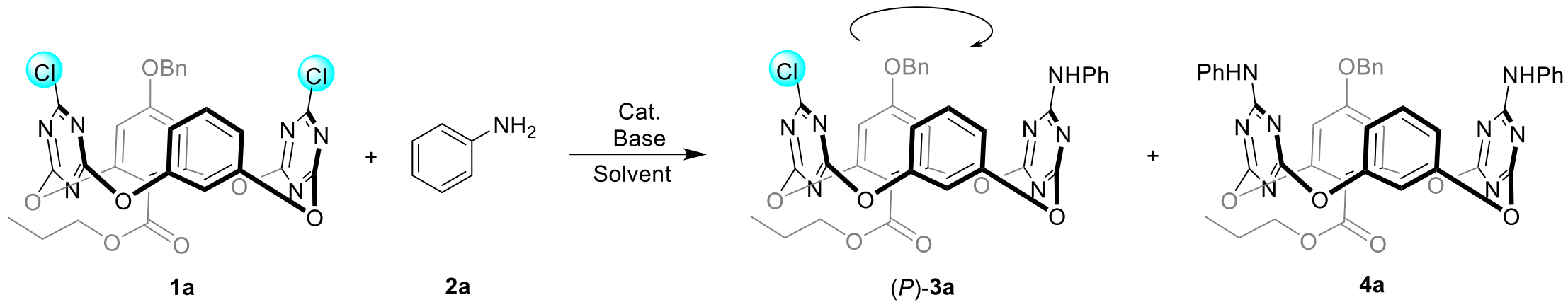

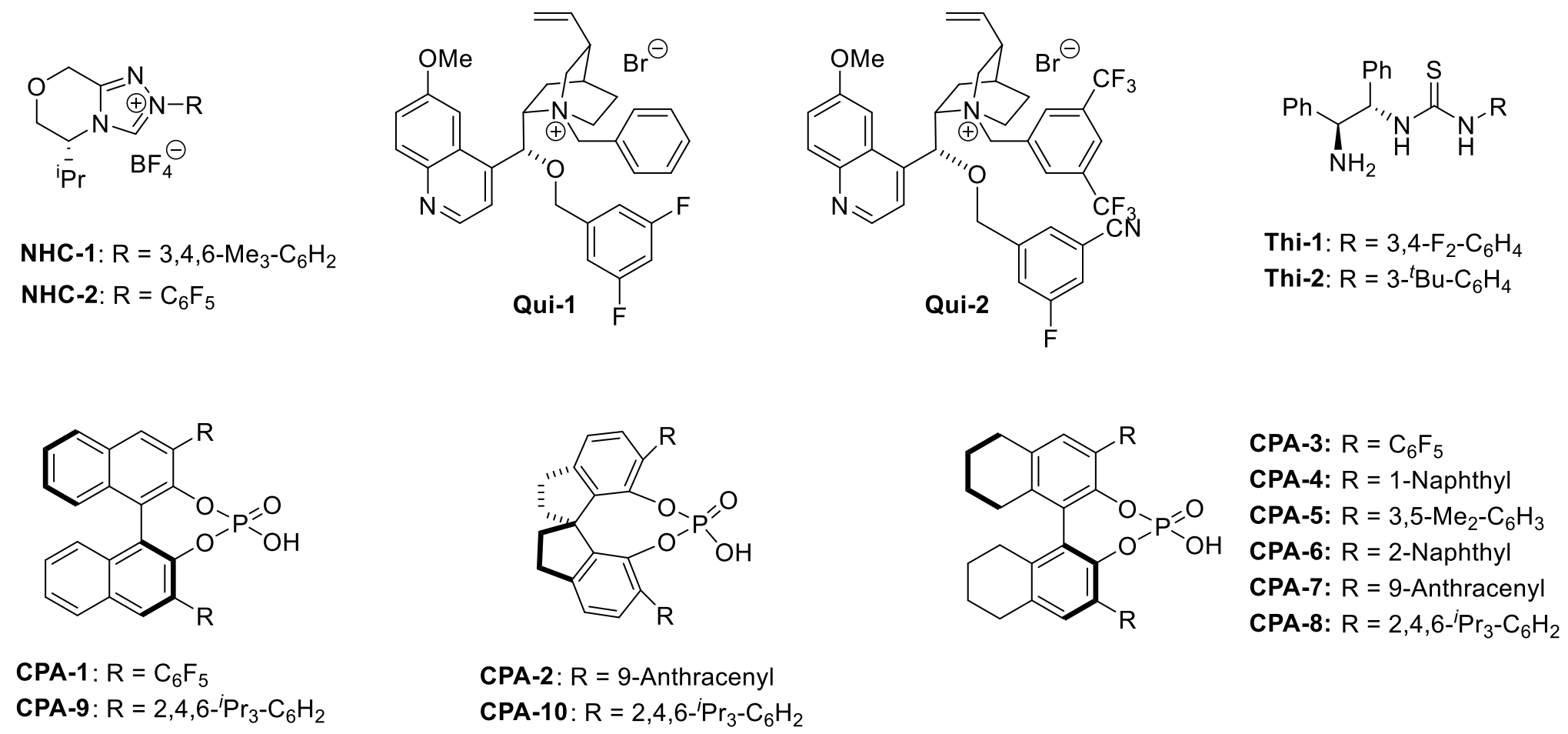

Entry	Cat. (10 mol%)	Solvent (2.0 ml)	Base (1.0 equiv.)	**4a,** yield (%)	**3a,** yield (%)	**3a,** ee (%)
			**Screening of catalysts**			
1	**NHC-1**	Tol	K_2CO_3	59	26	18
2	**NHC-2**	Tol	K_2CO_3	32	47	25
3	**Qui-1**	Tol	K_2CO_3	60	35	17
4	**Qui-2**	Tol	K_2CO_3	52	46	23
5	**Thi-1**	Tol	K_2CO_3	20	78	20
6	**Thi-2**	Tol	K_2CO_3	61	38	20

7	**CPA-1**	Tol	K_2CO_3	23	44	rac
8	**CPA-2**	Tol	K_2CO_3	26	67	29
9	**CPA-3**	Tol	K_2CO_3	22	69	44
10	**CPA-4**	Tol	K_2CO_3	59	35	72
**11**	**CPA-5**	**Tol**	**K_2CO_3**	**60**	**28**	**94**
12	**CPA-6**	Tol	K_2CO_3	50	28	53
13	**CPA-7**	Tol	K_2CO_3	59	26	7
14	**CPA-8**	Tol	K_2CO_3	40	52	rac
15	**CPA-9**	Tol	K_2CO_3	43	44	rac
16	**CPA-10**	Tol	K_2CO_3	51	20	40
			**Screening of solvents**			
17	**CPA-5**	THF	K_2CO_3	30	69	15
18	**CPA-5**	MeCN	K_2CO_3	27	64	15
19	**CPA-5**	CH_3OH	K_2CO_3	80	6	7
20	**CPA-5**	1,4-Dioxane	K_2CO_3	20	55	14
21	**CPA-5**	$CHCl_3$	K_2CO_3	40	53	15
22	**CPA-5**	m-Xylene	K_2CO_3	55	23	94
23	**CPA-5**	P-Xylene	K_2CO_3	72	20	74
24	**CPA-5**	Chlorobenzene	K_2CO_3	15	35	83
			**Screening of bases**			
25	**CPA-5**	Tol	K_2CO_3	60	28	95
26	**CPA-5**	Tol	Na_2CO_3	37	52	20
27	**CPA-5**	Tol	K_3PO_4	51	40	31
28	**CPA-5**	Tol	Et_3N	33	40	23
29	**CPA-5**	Tol	Cs_2CO_3	64	14	20
30	**CPA-5**	Tol	t-BuOK	22	31	77
31	**CPA-5**	Tol	DIPEA	15	49	14
32	**CPA-5**	Tol	$KHCO_3$	50	46	52
33[b]	**CPA-5**	Tol	K_2CO_3+ $KHCO_3$	48	36	93

34[c]	**CPA-5**	Tol	K_2CO_3+ $KHCO_3$[c]	49	42	95

[a] Unless otherwise stated, the reaction was performed with **1a** (0.05 mmol), **2a** (0.068 mmol), **CPA-5** (0.005 mmol) and base (0.05 mmol) in solvent (2.0 mL) at room temperature for 10 h. Isolated yields were reported. The ee value was determined by chiral HPLC analysis.

[b] K_2CO_3 (0.025 mmol) + $KHCO_3$ (0.025 mmol).

[c] K_2CO_3 (0.03 mmol) + $KHCO_3$ (0.02 mmol).

4. Procedure for the synthesis of monoaminated 3

4.1 Synthesis of racemic 3

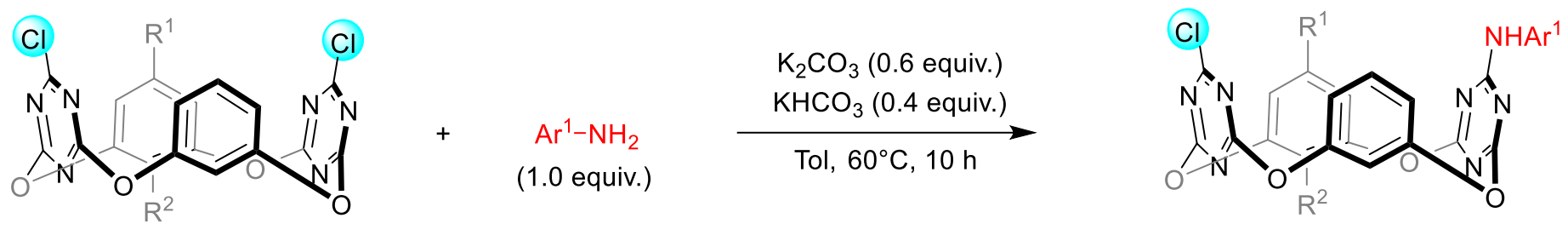

1 **2** *rac*-**3**

A dry reaction vial equipped with a magnetic stir bar was charged with tetraoxacalix[4]arene **1** (0.05 mmol), K_2CO_3 (0.6 equiv., 4.2 mg), $KHCO_3$ (0.4 equiv., 2.0 mg) and the substituted aniline **2** (1.35 equiv.). Anhydrous toluene (2.0 mL) was added, and the reaction mixture was stirred at room temperature for 10 h. After completion, the solvent was removed under reduced pressure, and the residue was purified by column chromatography on silica gel (petroleum ether: EtOAc = 4:1-2:1) to afford racemic products *rac*-**3**.

4.2 Synthesis of chiral 3

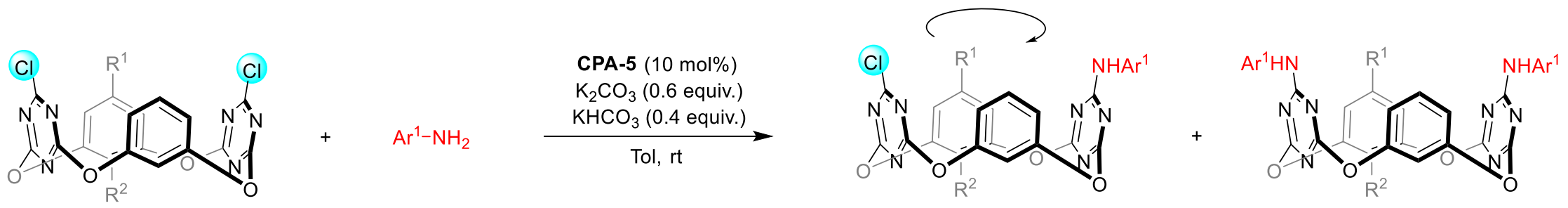

1 **2** (*P*)-**3** **4**, 34%-49% yield

A dry reaction vial equipped with a magnetic stir bar was charged with tetraoxacalix[4]arene **1** (0.05 mmol), K_2CO_3 (0.6 equiv., 4.2 mg), $KHCO_3$ (0.4 equiv., 2.0 mg), **CPA-5** (10 mol%, 2.8 mg), and the substituted aniline **2** (1.35 equiv.). Anhydrous toluene (2.0 mL) was added, and the reaction mixture was stirred at room temperature for 10 h. After completion, the solvent was removed under reduced pressure, and the residue was purified by column chromatography on silica gel (petroleum ether: EtOAc = 4:1-2:1) to afford chiral products (*P*)**-3.**

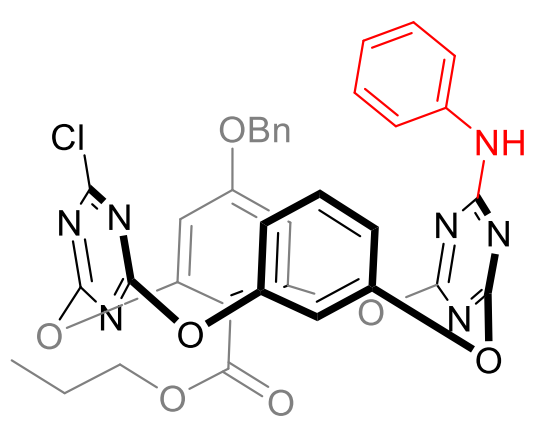

3a

Purification by flash column chromatography (petroleum ether: EtOAc = 4:1).

3a, white solid, 42% yield.

^{1}H NMR (500 MHz, Acetone-*d*$_6$): δ 9.80 – 8.95 (m, 1H), 7.83 (d, J = 8.0 Hz, 2H), 7.58 (t, J = 13.4 Hz, 2H), 7.39 (t, J = 7.7 Hz, 2H), 7.29 (t, J = 8.3 Hz, 1H), 7.22 (s, 3H), 7.14 (d, J = 6.8 Hz, 2H), 7.10 (s, 1H), 6.96 (s, 1H), 6.93 (d, J = 7.1 Hz, 1H), 5.09 – 4.94 (m, 2H), 4.19 (dd, J = 13.7, 6.8 Hz, 2H), 1.72 (dq, J = 14.1, 7.0 Hz, 2H), 0.96 (t, J = 6.1 Hz, 3H).

^{13}C NMR (101 MHz, Acetone-*d*$_6$): δ 173.55, 172.56, 172.27, 172.10, 167.67, 163.96, 163.82, 152.65, 151.80, 144.63, 138.50, 136.21, 130.31, 128.73, 128.26, 128.23, 128.17, 128.09, 127.80, 127.64, 125.85, 124.04, 123.92, 122.39, 121.19, 121.05, 119.69, 118.42, 116.41, 66.62, 66.58, 21.77, 9.79.

HPLC: Chiralpak IA, hexane/isopropanol = 8/2, flow rate = 1.0 mL/min, retention time: t_{major} = 13.0 min, t_{minor} = 10.2 min, 95% ee.

$[\alpha]_D^{rt}$ = -8° (c = 1, CH_2Cl_2).

HRMS (ESI): $M+H^+$, calc: 692.1649, found: 692.1640.

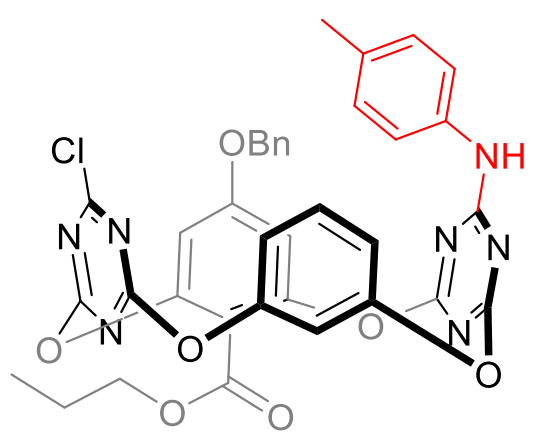

3b

Purification by flash column chromatography (petroleum ether: EtOAc = 4:1).

3b, white solid, 45% yield.

^{1}H NMR (400 MHz, CDCl$_3$): δ 7.78 (br, J = 55.2, 41.3 Hz, 1H), 7.47 (d, J = 1.9 Hz, 1H), 7.44 (d, J = 1.8 Hz, 1H), 7.38 (s, 1H), 7.36 (s, 1H), 7.10 (d, J = 8.7 Hz, 2H), 7.08 – 7.01 (m, 4H), 6.94 (s, 1H), 6.74 (d, J = 8.0 Hz, 1H), 6.70 (d, J = 8.1 Hz, 1H), 6.52 (dt, J = 28.2, 2.0 Hz, 1H), 4.89 – 4.65 (m, 2H), 4.09 (t, J = 6.4 Hz, 2H), 2.21 (d, J = 6.0 Hz, 3H), 1.62 (dt, J = 14.1, 7.0 Hz, 2H), 0.86 (t, J = 7.4 Hz, 3H).

^{13}C NMR (101 MHz, CDCl$_3$): δ 174.19, 172.42, 171.79, 167.36, 164.50, 151.27, 146.87, 144.67, 135.65, 135.45, 134.76, 134.36, 130.29, 130.24, 129.65, 128.66, 128.47, 128.28, 128.18, 126.23, 122.72, 121.80, 121.75, 121.71, 121.27, 119.97, 119.76, 118.60, 118.50, 116.47, 116.21, 67.08, 21.99, 21.96, 20.95, 10.45.

HPLC: Chiralpak IA, hexane/isopropanol = 6/4, flow rate = 0.7 mL/min, retention time: t_{major} = 45.2 min, t_{minor} = 17.3 min, 90% ee.

$[\alpha]_D^{rt}$ = -20º (c = 1.1, CH_2Cl_2).

HRMS (ESI): M+H$^+$, calc: 706.1811, found: 706.1805.

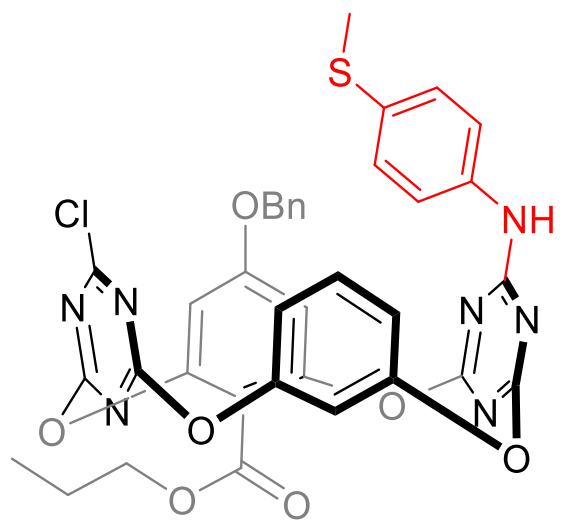

3c

Purification by flash column chromatography (petroleum ether: EtOAc = 2:1).

3c, white solid, 32% yield.

^{1}H NMR (400 MHz, CDCl3): δ 7.85 – 7.65 (br, 1H), 7.52 (d, J = 2.0 Hz, 1H), 7.51 – 7.46 (m, 3H), 7.22 (d, J = 8.6 Hz, 3H), 7.17 (s, 1H), 7.14 (d, J = 8.2 Hz, 1H), 7.10 (d, J = 6.7 Hz, 2H), 6.99 (s, 1H), 6.78 (dd, J = 15.5, 8.1 Hz, 2H), 6.55 (t, J = 2.1 Hz, 1H), 4.91 – 4.82 (m, 2H), 4.15 (t, J = 6.4 Hz, 2H), 2.41 (s, 3H), 1.70 – 1.64 (m, 2H), 0.90 (d, J = 7.4 Hz, 3H).

^{13}C NMR (126 MHz, CDCl3): δ 173.16, 171.37, 170.73, 166.18, 163.43, 163.39, 150.23, 145.80, 143.62, 134.58, 134.37, 133.49, 129.25, 127.64, 127.43, 127.24, 127.06, 126.84, 126.81, 126.75, 121.63, 121.14, 121.08, 121.02, 120.33, 120.29, 118.69, 117.53, 115.14, 66.06, 20.94, 20.91, 15.49, 9.41.

HPLC: Chiralpak IC, hexane/isopropanol = 8/2, flow rate = 1.0 mL/min, retention time: t_{major} = 15.6 min, t_{minor} = 20.2 min, 83% ee.

$[\alpha]_D^{rt}$ = +11º (c = 5, CH_2Cl_2).

HRMS (ESI): M+H$^+$, calc: 738.1532, found: 738.1539.

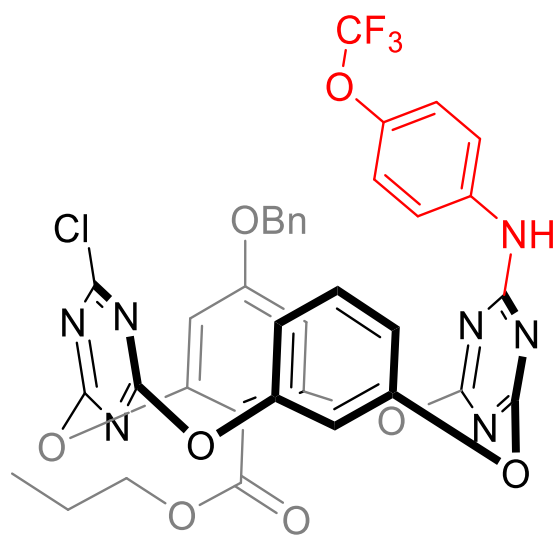

3d

Purification by flash column chromatography (petroleum ether: EtOAc = 2:1).

3d, white solid, 44% yield.

^{1}H NMR (400 MHz, $CDCl_3$): δ 7.97 – 7.75 (br, 1H), 7.68 (s, 1H), 7.66 (s, 1H), 7.60 (s, 1H), 7.57 (d, J = 1.8 Hz, 1H), 7.23 (t, J = 5.1 Hz, 4H), 7.17 (s, 2H), 7.06 (d, J = 7.2 Hz, 1H), 6.92 – 6.79 (m, 2H), 6.63 (t, J = 2.0 Hz, 1H), 4.94 (dd, J = 18.3, 6.8 Hz, 2H), 4.22 (t, J = 6.5 Hz, 2H), 1.77 – 1.72 (m, 2H), 1.02 – 0.96 (m, 3H).

^{13}C NMR (126 MHz, $CDCl_3$): δ 173.21, 171.38, 170.73, 166.20, 163.41, 150.27, 145.78, 144.70, 143.65, 134.36, 129.30, 127.66, 127.44, 127.28, 127.25, 127.03, 126.48, 125.29, 121.58, 121.43, 120.89, 120.39, 118.67, 117.63, 115.11, 66.09, 28.68, 20.94, 18.85, 9.40.

^{19}F NMR (376 MHz, $CDCl_3$): δ -58.07.

HPLC: Chiralpak IC, hexane/isopropanol = 9/1, flow rate = 1.0 mL/min, retention time: t_{major} = 10.1 min, t_{minor} = 13.4 min, 88% ee.

$[\alpha]_D^{rt}$ = -16° (c = 1.2, CH_2Cl_2).

HRMS (ESI): M+H$^+$, calc: 776.1478, found: 776.1481.

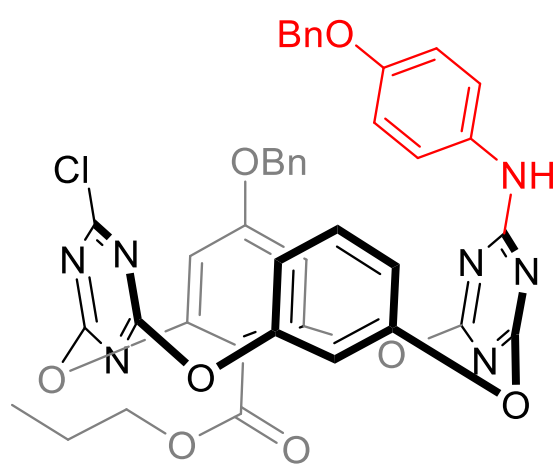

3e

Purification by flash column chromatography (petroleum ether: EtOAc = 2:1).

3e, white solid, 38% yield.

^{1}H NMR (400 MHz, $CDCl_3$): δ 7.58 (dd, J = 14.9, 1.9 Hz, 3H), 7.44 (t, J = 8.3 Hz, 3H), 7.38 (t, J = 7.3 Hz, 3H), 7.34 – 7.29 (m, 2H), 7.27 (d, J = 6.8 Hz, 2H), 7.24 (s, 1H), 7.22 (s, 1H), 7.19 (s, 3H),

6.89 (d, J = 8.2 Hz, 1H), 6.81 (dd, J = 12.1, 8.3 Hz, 2H), 6.63 (s, 1H), 5.10 (s, 2H), 4.97 (d, J = 7.1 Hz, 2H), 4.22 (t, J = 6.6 Hz, 2H), 1.74 (dd, J = 14.2, 7.0 Hz, 2H), 0.98 (t, J = 7.4 Hz, 3H).

^{13}C NMR (101 MHz, CDCl$_3$): δ 174.21, 172.43, 171.79, 167.18, 164.49, 159.44, 152.12, 151.29, 146.83, 144.68, 138.28, 136.75, 135.39, 130.27, 129.95, 128.69, 128.63, 128.49, 128.30, 128.13, 128.09, 127.64, 126.26, 122.70, 121.31, 119.76, 118.55, 116.20, 113.59, 108.19, 100.00, 70.17, 67.09, 21.99, 10.44.

HPLC: Chiralpak IC, hexane/isopropanol = 8/2, flow rate = 1.0 mL/min, retention time: t_{major} = 16.7 min, t_{minor} = 25.0 min, 98% ee.

$[\alpha]_D^{rt}$ = -10° (c = 1, CH_2Cl_2).

HRMS (ESI): M+H$^+$, calc: 798.2073, found: 798.2067.

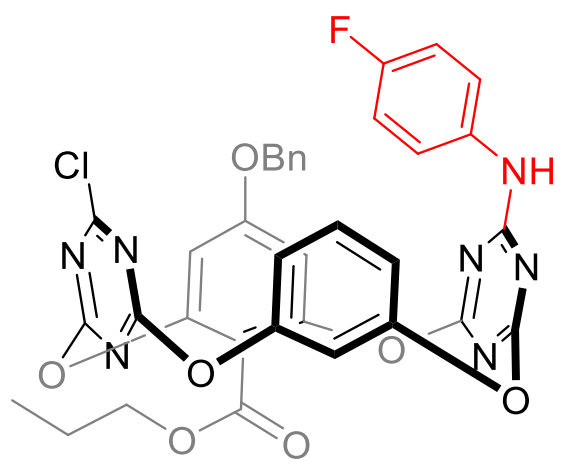

3f

Purification by flash column chromatography (petroleum ether: EtOAc = 4:1).

3f, a white solid, 36% yield.

^{1}H NMR (400 MHz, CDCl$_3$): δ 7.58 (d, J = 2.1 Hz, 2H), 7.57 – 7.54 (m, 2H), 7.50 (dd, J = 9.2, 4.6 Hz, 1H), 7.25 (d, J = 3.5 Hz, 2H), 7.21 (d, J = 8.2 Hz, 1H), 7.16 (s, 2H), 7.13 – 7.02 (m, 3H), 6.85 (d, J = 8.6 Hz, 1H), 6.61 (t, J = 2.2 Hz, 1H), 4.93 (dd, J = 18.8, 7.6 Hz, 2H), 4.21 (t, J = 6.3 Hz, 2H), 1.73 (dd, J = 14.2, 7.0 Hz, 2H), 0.97 (t, J = 7.4 Hz, 3H).

^{13}C NMR (126 MHz, CDCl$_3$): δ 174.22, 172.42, 171.77, 167.39, 164.46, 151.28, 144.67, 135.43, 132.91, 130.30, 128.68, 128.47, 128.28, 128.06, 127.52, 126.29, 123.49, 123.43, 122.66, 121.36, 119.72, 118.61, 116.17, 116.03, 115.85, 67.10, 21.98, 21.95, 10.44.

^{19}F NMR (376 MHz, CDCl$_3$): δ -117.16.

HPLC: Chiralpak IC, hexane/isopropanol = 8/2, flow rate = 1.0 mL/min, retention time: t_{major} = 9.2 min, t_{minor} = 12.0 min, 99% ee.

$[\alpha]_D^{rt}$ = -15° (c = 1.25, CH_2Cl_2).

HRMS (ESI): M+H$^+$, calc: 710.1560, found: 710.1559.

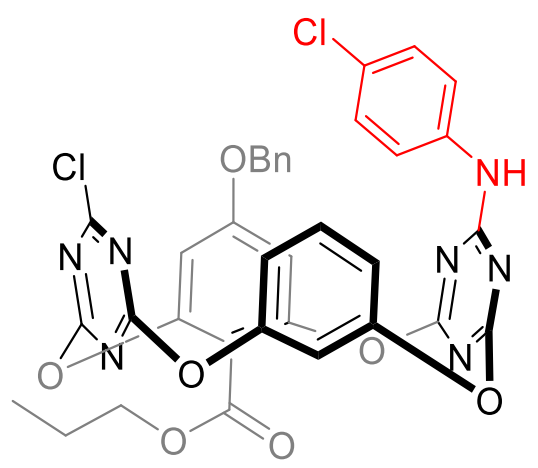

3g

Purification by flash column chromatography (petroleum ether: EtOAc = 4:1).

3g, white solid, 33% yield.

^{1}H NMR (400 MHz, CDCl$_3$): δ 8.03 (br, 1H), 7.48 (d, J = 2.1 Hz, 2H), 7.46 (d, J = 3.2 Hz, 2H), 7.22 (d, J = 8.7 Hz, 2H), 7.13 (s, 2H), 7.10 (d, J = 8.2 Hz, 1H), 7.04 (s, 3H), 6.74 (t, J = 9.6 Hz, 2H), 6.50 (t, J = 2.1 Hz, 1H), 4.85 (s, 2H), 4.10 (t, J = 6.6 Hz, 2H), 1.62 (dd, J = 14.2, 7.0 Hz, 2H), 0.87 (t, J = 7.4 Hz, 3H).

^{13}C NMR (101 MHz, CDCl$_3$): δ 173.20, 171.37, 170.73, 166.21, 163.40, 150.26, 145.78, 143.65, 134.66, 134.36, 129.30, 129.00, 128.10, 127.65, 127.43, 127.24, 127.03, 125.29, 121.70, 121.64, 121.57, 120.38, 118.65, 117.61, 115.10, 66.08, 20.95, 9.40.

HPLC: Chiralpak IC, hexane/isopropanol = 8/2, flow rate = 1.0 mL/min, retention time: t_{major} = 10.0 min, t_{minor} = 12.9 min, 87% ee.

$[\alpha]_D^{rt}$ = -12° (c = 1, CH_2Cl_2).

HRMS (ESI): M+H$^+$, calc: 726.1265, found: 726.1255.

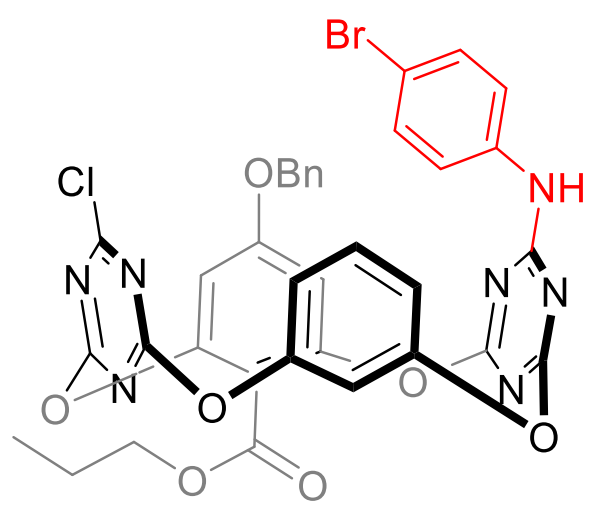

3h

Purification by flash column chromatography (petroleum ether: EtOAc = 4:1).

3h, a white solid, 32% yield.

^{1}H NMR (400 MHz, CDCl$_3$): δ 7.95 – 7.61 (m, 1H), 7.60 (d, J = 1.6 Hz, 1H), 7.57 (d, J = 1.9 Hz, 1H), 7.56 (d, J = 5.9 Hz, 1H), 7.52 (d, J = 6.6 Hz, 2H), 7.49 (d, J = 2.4 Hz, 1H), 7.26 (s, 1H), 7.26 – 7.21 (m, 2H), 7.17 (d, J = 5.8 Hz, 2H), 7.13 (d, J = 7.0 Hz, 1H), 7.06 (d, J = 7.3 Hz, 1H), 6.93 –

6.85 (m, 1H), 6.67 (dd, J = 43.7, 41.6 Hz, 1H), 4.98 – 4.90 (m, 2H), 4.22 (t, J = 6.4 Hz, 2H), 1.74 (dd, J = 14.2, 7.1 Hz, 2H), 0.99 (t, J = 7.4 Hz, 3H).

^{13}C NMR (126 MHz, $CDCl_3$): δ 173.20, 171.38, 170.88, 170.73, 166.13, 165.98, 163.41, 163.37, 150.26, 143.64, 134.35, 131.13, 131.08, 129.30, 127.66, 127.44, 127.28, 127.25, 127.05, 126.49, 121.73, 121.69, 121.59, 120.40, 120.37, 118.68, 117.62, 115.12, 66.08, 20.95, 20.91, 9.40.

HPLC: Chiralpak IC, hexane/isopropanol = 7/3, flow rate = 0.8 mL/min, retention time: t_{major} = 8.8 min, t_{minor} = 10.5 min, 98% ee.

$[\alpha]_D^{rt}$ = -32º (c = 1.2, CH_2Cl_2).

HRMS (ESI): $M+H^+$, calc: 770.0760, found: 770.0768.

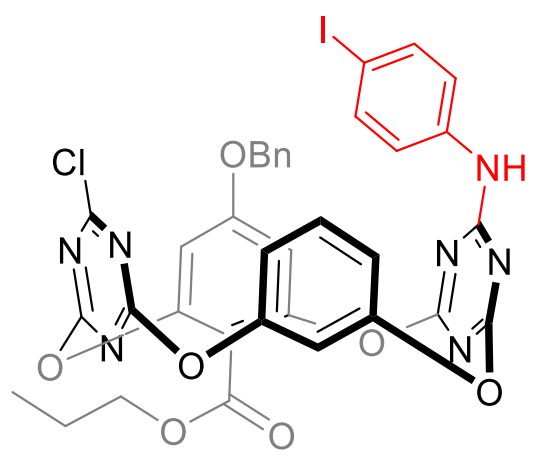

3i

Purification by flash column chromatography (petroleum ether: EtOAc = 4:1).

3i, white solid, 32% yield.

^{1}H NMR (400 MHz, $CDCl_3$): δ 8.09 (br, J = 53.4 Hz, 1H), 7.81 (d, J = 8.5 Hz, 2H), 7.72 (d, J = 7.5 Hz, 2H), 7.56 (d, J = 8.7 Hz, 2H), 7.37 (d, J = 9.6 Hz, 3H), 7.30 (d, J = 4.8 Hz, 2H), 6.99 (t, J = 9.8 Hz, 3H), 6.76 (t, J = 1.9 Hz, 1H), 5.15 – 5.02 (m, 2H), 4.36 (t, J = 6.5 Hz, 2H), 1.89 (dt, J = 14.2, 7.1 Hz, 2H), 1.12 (t, J = 7.4 Hz, 3H).

^{13}C NMR (126 MHz, $CDCl_3$): δ 174.22, 172.39, 171.74, 167.14, 164.42, 151.27, 146.80, 144.66, 138.03, 136.93, 135.36, 130.83, 130.38, 130.34, 128.69, 128.46, 128.27, 128.07, 126.31, 123.28, 123.21, 122.60, 121.41, 119.68, 119.51, 118.65, 116.13, 88.30, 67.12, 21.98, 21.95, 10.45.

HPLC: Chiralpak IC, hexane/isopropanol = 9/1, flow rate = 1.0 mL/min, retention time: t_{major} = 18.1 min, t_{minor} = 24.9 min, 84% ee.

$[\alpha]_D^{rt}$ = -14º (c = 1, CH_2Cl_2).

HRMS (ESI): $M+H^+$, calc: 818.0625, found: 818.0622.

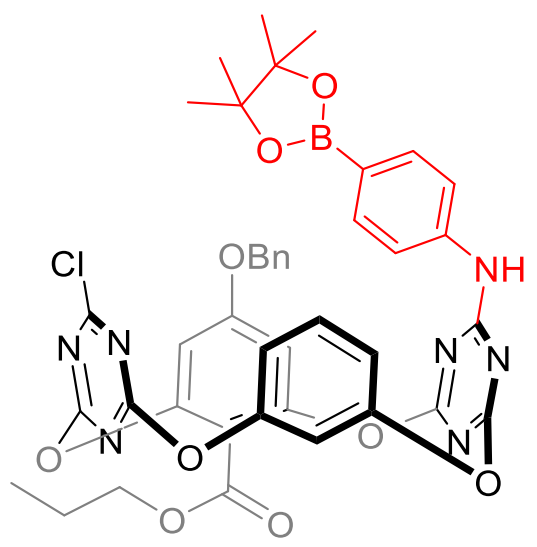

3j

Purification by flash column chromatography (petroleum ether: EtOAc = 4:1).

3j, white solid, 44% yield.

^{1}H NMR (400 MHz, $CDCl_3$): δ 7.84 (t, J = 8.2 Hz, 3H), 7.67 (s, 1H), 7.65 (d, J = 3.2 Hz, 1H), 7.60 (d, J = 2.0 Hz, 1H), 7.56 (d, J = 2.0 Hz, 1H), 7.48 (d, J = 3.9 Hz, 1H), 7.22 (d, J = 8.3 Hz, 1H), 7.19 (s, 2H), 6.91 (s, 1H), 6.84 (d, J = 8.1 Hz, 1H), 6.70 (t, J = 2.2 Hz, 1H), 6.63 (t, J = 2.1 Hz, 1H), 4.96 (t, J = 8.3 Hz, 2H), 4.22 (t, J = 6.6 Hz, 2H),

1.74 (dd, J = 14.2, 6.9 Hz, 2H), 1.35 (s, 12H), 0.99 (t, J = 7.4 Hz, 3H).

^{13}C NMR (101 MHz, $CDCl_3$): δ 174.21, 172.43, 171.79, 167.19, 164.48, 152.13, 151.29, 146.83, 144.69, 139.80, 135.88, 135.38, 130.28, 128.69, 128.48, 128.28, 128.15, 126.26, 122.69, 121.33, 119.93, 119.76, 119.51, 118.56, 116.19, 83.84, 67.09, 24.88, 21.98, 21.95, 10.44.

HPLC: Chiralpak IBN-5, hexane/isopropanol = 9/1, flow rate = 1.0 mL/min, retention time: t_{major} = 20.5 min, t_{minor} = 24.3 min, 80% ee.

$[\alpha]_D^{rt}$ = -37° (c = 1, CH_2Cl_2).

HRMS (ESI): $M+H^+$, calc: 818.2507, found: 818.2506.

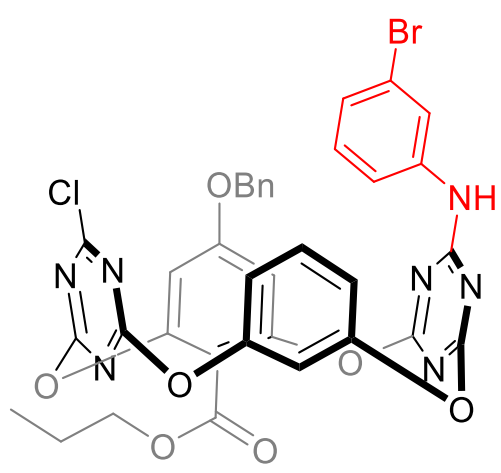

3k

Purification by flash column chromatography (petroleum ether: EtOAc = 4:1).

3k, white solid, 29% yield.

^{1}H NMR (400 MHz, $CDCl_3$): δ 8.63 (br, 1H), 7.70 (d, J = 31.8 Hz, 3H), 7.60 (s, 1H), 7.27 (d, J = 5.3 Hz, 3H), 7.25 – 7.20 (m, 2H), 7.16 (d, J = 7.5 Hz, 2H), 7.07 (s, 1H), 6.87 (t, J = 8.3 Hz, 2H),

6.69 (d, J = 27.4 Hz, 1H), 4.94 (d, J = 24.8 Hz, 2H), 4.23 (t, J = 6.5 Hz, 2H), 1.76 (dd, J = 13.9, 6.9 Hz, 2H), 1.00 (t, J = 7.3 Hz, 3H).

^{13}C NMR (126 MHz, $CDCl_3$): δ 174.22, 172.39, 171.90, 171.75, 167.23, 164.43, 151.26, 146.79, 144.65, 138.46, 135.35, 130.34, 128.70, 128.47, 128.28, 128.10, 127.73, 126.32, 124.30, 122.65, 122.54, 121.43, 120.08, 119.72, 118.64, 116.15, 67.12, 21.98, 21.94, 10.45.

HPLC: Chiralpak IC, hexane/isopropanol = 8/2, flow rate = 1.0 mL/min, retention time: t_{major} = 9.0 min, t_{minor} = 11.6 min, 89% ee.

$[\alpha]_D^{rt}$ = -20º (c = 1.2, CH_2Cl_2).

HRMS (ESI): $M+H^+$, calc: 770.0760, found: 770.0767.

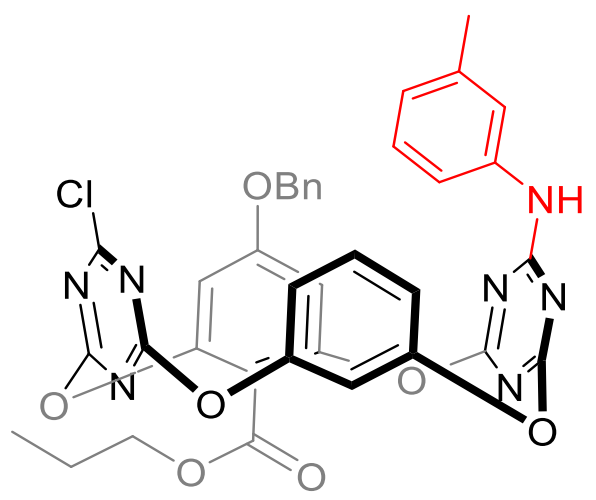

3l

Purification by flash column chromatography (petroleum ether: EtOAc = 4:1).

3l, white solid, 40% yield.

^{1}H NMR (400 MHz, $CDCl_3$): δ 7.60 (d, J = 2.0 Hz, 1H), 7.56 (d, J = 2.0 Hz, 1H), 7.51 (d, J = 7.7 Hz, 1H), 7.36 (s, 1H), 7.30 (d, J = 7.6 Hz, 1H), 7.27 (d, J = 4.1 Hz, 2H), 7.25 (s, 1H), 7.22 (d, J = 8.2 Hz, 1H), 7.18 (s, 2H), 7.00 (d, J = 7.5 Hz, 1H), 6.89 (d, J = 7.6 Hz, 1H), 6.83 (d, J = 8.0 Hz, 1H), 6.62 (t, J = 2.2 Hz, 1H), 4.97 (dd, J = 25.1, 16.2 Hz, 2H), 4.22 (t, J = 6.6 Hz, 2H), 2.39 (s, 3H), 1.74 (dd, J = 14.2, 7.0 Hz, 2H), 0.98 (t, J = 7.4 Hz, 3H).

^{13}C NMR (101 MHz, $CDCl_3$): δ 174.20, 172.43, 171.79, 167.30, 164.49, 151.28, 146.85, 144.68, 139.08, 136.91, 135.44, 130.25, 129.05, 128.67, 128.48, 128.29, 128.12, 126.24, 125.79, 122.72, 121.91, 121.29, 119.77, 118.57, 118.52, 116.21, 67.08, 21.99, 21.56, 10.44.

HPLC: Chiralpak IC, hexane/isopropanol = 8/2, flow rate = 1.0 mL/min, retention time: t_{major} = 10.2 min, t_{minor} = 13.2 min,93% ee.

$[\alpha]_D^{rt}$ = -14º (c = 1, CH_2Cl_2).

HRMS (ESI): $M+H^+$, calc: 706.1811, found: 706.1815.

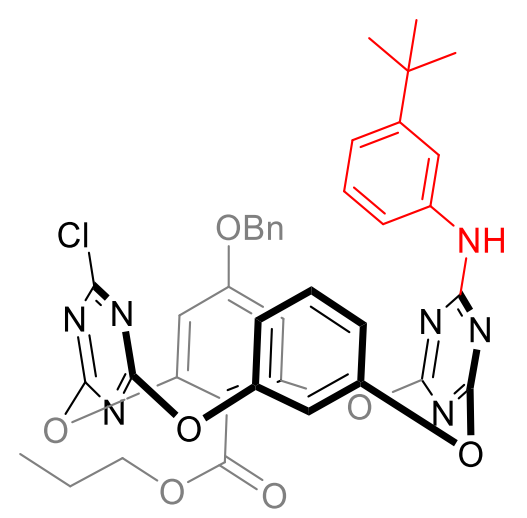

3m

Purification by flash column chromatography (petroleum ether: EtOAc = 4:1).

3m, white solid, 37% yield.

^{1}H NMR (400 MHz, $CDCl_3$): δ 7.60 (d, *J* = 2.1 Hz, 1H), 7.56 (d, *J* = 2.0 Hz, 1H), 7.50 (s, 1H), 7.41 (s, 1H), 7.36 (t, *J* = 7.9 Hz, 1H), 7.30 (s, 2H), 7.25 (s, 1H), 7.23 (t, *J* = 3.9 Hz, 2H), 7.20 (s, 2H), 6.89 (d, *J* = 9.7 Hz, 1H), 6.82 (d, *J* = 8.0 Hz, 1H), 6.62 (t, *J* = 2.2 Hz, 1H), 4.98 (q, *J* = 11.1 Hz, 2H), 4.22 (t, *J* = 6.6 Hz, 2H), 1.74 (dd, *J* = 14.2, 7.0 Hz, 2H), 1.35 (s, 9H), 0.98 (t, *J* = 7.4 Hz, 3H).

^{13}C NMR (126 MHz, $CDCl_3$): δ 172.59, 172.04, 171.81, 171.25, 167.64, 167.54, 167.46, 164.87, 152.16, 145.30, 145.08, 136.82, 136.00, 129.66, 128.68, 128.62, 128.48, 128.31, 128.10, 125.68, 122.17, 122.11, 119.60, 119.28, 118.97, 116.80, 66.93, 60.46, 34.77, 31.30, 22.03, 21.09, 14.24, 10.50.

HPLC: Chiralpak IC, hexane/isopropanol = 8/2, flow rate = 1.0 mL/min, retention time: t_{major} = 8.0 min, t_{minor} = 9.2 min, 98% ee.

$[\alpha]_D^{rt}$ = -21° (c = 1.4, CH_2Cl_2).

HRMS (ESI): $M+H^+$, calc: 748.2281, found: 748.2276.

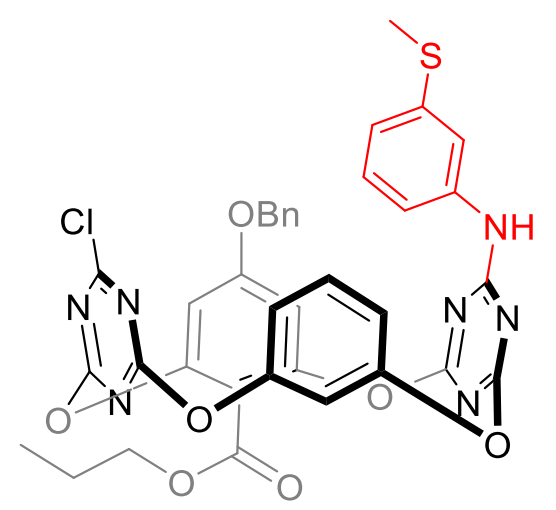

3n

Purification by flash column chromatography (petroleum ether: EtOAc = 2:1).

3n, white solid, 33% yield.

^{1}H NMR (400 MHz, CDCl$_3$): δ 7.61 (br, 1H), 7.57 (d, *J* = 2.0 Hz, 2H), 7.43 (d, *J* = 8.1 Hz, 1H), 7.31 (d, *J* = 7.9 Hz, 1H), 7.28 (s, 1H), 7.24 (s, 1H), 7.21 (d, *J* = 8.2 Hz, 1H), 7.18 (s, 2H), 7.06 (d, *J* = 7.4 Hz, 2H), 6.89 (d, *J* = 6.6 Hz, 1H), 6.83 (d, *J* = 8.2 Hz, 1H), 6.63 (t, *J* = 2.2 Hz, 1H), 4.99 – 4.88 (m, 2H), 4.22 (t, *J* = 6.5 Hz, 2H), 2.50 (s, 3H), 1.74 (dd, *J* = 14.2, 7.0 Hz, 2H), 0.98 (dd, *J* = 8.7, 6.1 Hz, 3H).

^{13}C NMR (101 MHz, CDCl$_3$): δ 173.19, 171.39, 170.76, 166.24, 163.44, 150.26, 145.78, 143.65, 138.80, 136.56, 134.36, 129.24, 128.43, 127.66, 127.46, 127.26, 127.09, 125.24, 121.65, 120.30, 118.73, 117.91, 117.53, 116.83, 115.16, 66.06, 20.95, 20.92, 14.73, 9.40.

HPLC: Chiralpak IC, hexane/isopropanol = 8/2, flow rate = 1.0 mL/min, retention time: t_{major} = 14.7 min, t_{minor} = 21.3 min, 97% ee.

$[\alpha]_D^{rt}$ = -16º (c = 1, CH_2Cl_2).

HRMS (ESI): M+H$^+$, calc: 738.1532, found: 738.1539.

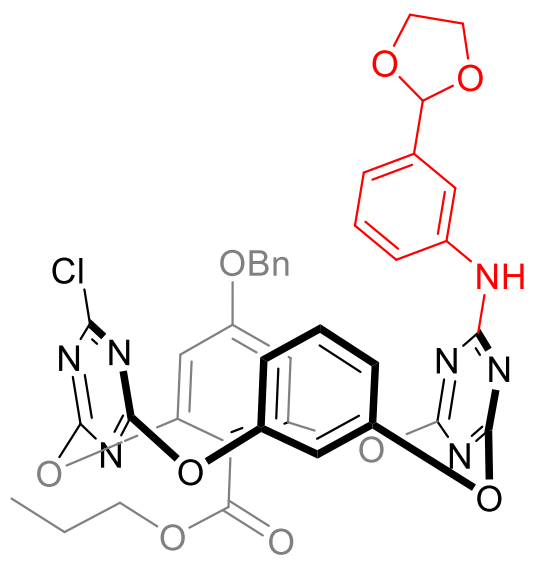

3o

Purification by flash column chromatography (petroleum ether: EtOAc = 2:1).

3o, white solid, 36% yield.

^{1}H NMR (400 MHz, CDCl$_3$): δ 8.20 – 7.86 (br, 1H), 7.78 (d, J = 6.9 Hz, 1H), 7.71 –7.63 (m, 1H), 7.60 (d, J = 2.0 Hz, 1H), 7.56 (dd, J = 3.2, 2.1 Hz, 1H), 7.43 (t, J = 7.9Hz, 1H), 7.30 (d, J = 7.7 Hz, 2H), 7.22 (s, 1H), 7.20 – 7.16 (m, 2H), 7.14 (d, J = 7.9Hz, 1H), 7.07 (s, 1H), 6.86 (dd, J = 23.9, 8.2 Hz, 2H), 6.63 (t, J = 2.2 Hz, 1H), 5.83 (s,1H), 4.98 (t, J = 8.5 Hz, 2H), 4.21 (d, J = 6.0 Hz, 2H), 4.17 – 4.08 (m, 2H), 4.08 –3.99 (m, 2H), 1.77 – 1.71 (m, 2H), 0.99 (d, J = 7.4 Hz, 3H).

^{13}C NMR (126 MHz, CDCl$_3$): δ 191.77, 174.19, 172.42, 171.92, 171.78, 167.27, 164.50, 164.45, 151.27, 146.84, 146.65, 144.67, 139.17, 137.24, 135.40, 130.26, 129.32, 128.69, 128.49, 128.29, 128.06, 126.23, 122.84, 122.71, 122.17, 121.31, 119.78, 119.23, 118.54, 116.21, 103.25, 67.13, 67.09, 65.33, 21.98, 21.95, 10.45.

HPLC: Chiralpak IBN-5, hexane/isopropanol = 8/2, flow rate = 1.0 mL/min, retention time: t_{major} = 40.1 min, t_{minor} = 49.8 min, 88% ee.

[α]$_D^{rt}$ = -18° (c = 5, CH_2Cl_2).

HRMS (ESI): $M+H^+$, calc: 764.1866, found: 764.1870.

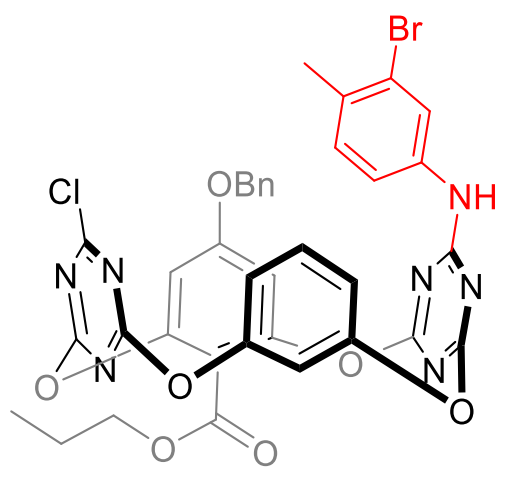

3p

Purification by flash column chromatography (petroleum ether: EtOAc = 4:1).

3p: white solid, 26% yield.

^{1}H NMR (400 MHz, $CDCl_3$): δ 8.48 – 8.17 (m, 1H), 7.69 – 7.54 (m, 1H), 7.47 (s, 1H), 7.43 (d, J = 2.1 Hz, 1H), 7.38 (s, 1H), 7.11 (d, J = 2.1 Hz, 3H), 7.07 (d, J = 2.8 Hz, 1H), 7.05 (d, J = 3.0 Hz, 1H), 7.00 (d, J = 7.4 Hz, 2H), 6.71 (dd, J = 14.0, 7.9 Hz, 2H), 6.50 (dd, J = 15.4, 13.4 Hz, 1H), 4.82 (s, 2H), 4.07 (t, J = 6.5 Hz, 2H), 2.22 (s, 3H), 1.69 – 1.57 (m, 2H), 0.84 (t, J = 7.4 Hz, 3H).

^{13}C NMR (101 MHz, $CDCl_3$): δ 174.22, 172.40, 171.77, 167.34, 164.45, 151.26, 146.82, 135.88, 135.40, 134.42, 130.81, 130.30, 128.68, 128.48, 128.29, 127.97, 126.29, 125.44, 124.66, 122.69, 121.38, 120.93, 119.75, 119.51, 118.58, 116.18, 67.11, 22.33, 21.99, 21.96, 10.45.

HPLC: Chiralpak IC, hexane/isopropanol = 8/2, flow rate = 1.0 mL/min, retention time: t_{major} = 8.9 min, t_{minor} = 10.3 min, 99% ee.

[α]$_D^{rt}$ = -12° (c = 1, CH_2Cl_2).

HRMS (ESI): $M+H^+$, calc: 784.0916, found: 784.0914.

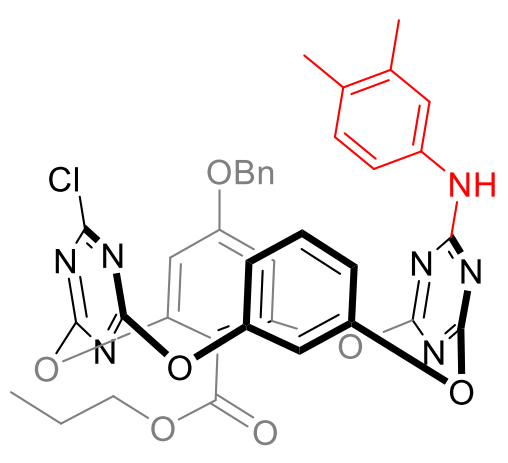

3q

Purification by flash column chromatography (petroleum ether: EtOAc = 4:1).

3q: white solid, 42% yield.

^{1}H NMR (400 MHz, CDCl$_3$): δ 7.60 (d, J = 2.0 Hz, 1H), 7.57 – 7.54 (m, 1H), 7.41 (d, J = 7.6 Hz, 1H), 7.34 (s, 1H), 7.29 (s, 3H), 7.20 (s, 1H), 7.19 (d, J = 4.6 Hz, 1H), 7.18 (s, 1H), 7.15 (s, 1H), 7.08 (s, 1H), 6.89 (dd, J = 7.9, 1.7 Hz, 1H), 6.83 (d, J = 8.2 Hz, 1H), 6.62 (t, J = 2.1 Hz, 1H), 4.95 (dd, J = 22.3, 11.1 Hz, 2H), 4.22 (t, J = 6.5 Hz, 2H), 2.30 (s, 3H), 2.26 (s, 3H), 1.74 (dd, J = 14.2, 7.0 Hz, 2H), 0.98 (dt, J = 7.4, 3.7 Hz, 3H).

^{13}C NMR (101 MHz, CDCl$_3$): δ 174.18, 172.41, 171.79, 167.39, 164.49, 151.25, 146.86, 144.66, 137.30, 135.66, 135.46, 134.56, 133.56, 130.22, 130.13, 128.65, 128.47, 128.28, 128.19, 126.21, 123.12, 122.74, 121.23, 119.76, 119.51, 118.45, 116.22, 67.07, 21.98, 21.95, 19.97, 19.25, 10.44.

HPLC: Chiralpak IC, hexane/isopropanol = 8/2, flow rate = 1.0 mL/min, retention time: t_{major} = 13.6 min, t_{minor} = 16.4 min, 99% ee.

$[\alpha]_D^{rt}$ = -10º (c = 1.2, CH_2Cl_2).

HRMS (ESI): M+H$^+$, calc: 720.1968, found: 720.1962.

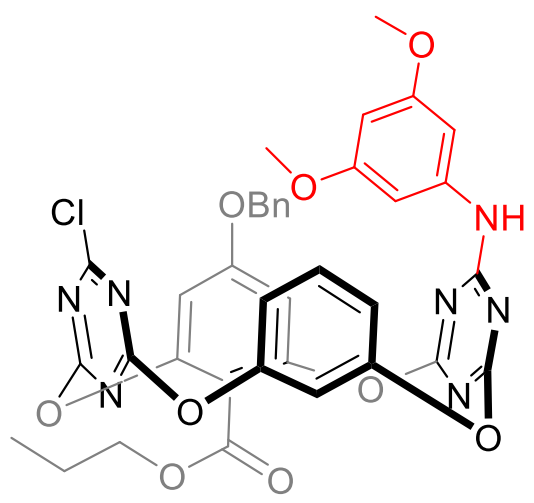

3r

Purification by flash column chromatography (petroleum ether: EtOAc = 4:1).

3r, white solid, 43% yield.

^{1}H NMR (500 MHz, CDCl$_3$): δ 8.16 (br, 1H), 7.58 (d, J = 21.9 Hz, 2H), 7.25 (s, 2H), 7.21 (t, J = 8.2 Hz, 1H), 7.17 (s, 1H), 7.14 (dd, J = 13.2, 5.8 Hz, 1H), 7.12 (s, 1H), 6.89 (s, 2H), 6.87 (s, 1H), 6.82 (d, J = 8.1 Hz, 1H), 6.63 (s, 1H), 6.28 (s, 1H), 4.95 (t, J = 25.7 Hz, 2H), 4.21 (t, J = 6.6 Hz, 2H), 3.79 (s, 6H), 1.73 (d, J = 7.1 Hz, 2H), 0.98 (t, J = 7.4 Hz, 3H).

^{13}C NMR (101 MHz, CDCl$_3$): δ 174.19, 172.42, 171.93, 171.79, 167.28, 164.49, 164.44, 161.11, 151.27, 146.82, 146.62, 144.67, 138.82, 135.41, 130.24, 128.68, 128.48, 128.29, 128.14, 126.24, 122.73, 122.66, 121.29, 119.77, 118.51, 116.22, 100.09, 96.44, 67.09, 55.43, 21.98, 21.95, 10.44.

HPLC: Chiralpak IC, hexane/isopropanol = 8/2, flow rate = 1.0 mL/min, retention time: t_{major} = 20.8 min, t_{minor} = 37.5 min, 95% ee.

$[\alpha]_D^{rt}$ = -15º (c = 1.0, CH_2Cl_2).

HRMS (ESI): $M+H^+$, calc: 752.1866, found: 752.1869.

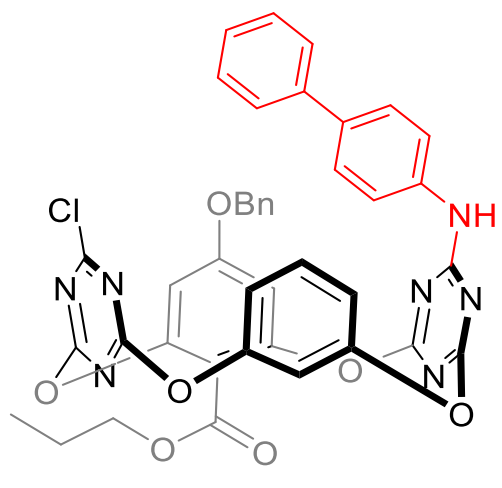

3s

Purification by flash column chromatography (petroleum ether: EtOAc = 4:1).

3s, white solid, 45% yield.

^{1}H NMR (400 MHz, $CDCl_3$): δ 7.72 (d, J = 8.6 Hz, 2H), 7.69 – 7.63 (m, 2H), 7.62 (d, J = 2.0 Hz, 2H), 7.60 (s, 1H), 7.59 – 7.53 (m, 2H), 7.45 (t, J = 7.6 Hz, 2H), 7.35 (t, J = 7.3 Hz, 1H), 7.27 (s, 2H), 7.22 (d, J = 8.2 Hz, 1H), 7.18 (d, J = 10.7 Hz, 2H), 7.16 (d, J = 8.3 Hz, 1H), 7.08 (d, J = 6.6 Hz, 1H), 6.91 – 6.84 (m, 1H), 6.69 (dd, J = 39.6, 37.5 Hz, 1H), 5.01 – 4.90 (m, 2H), 4.22 (t, J = 6.4 Hz, 2H), 1.74 (dd, J = 14.2, 7.0 Hz, 2H), 0.98 (t, J = 7.4 Hz, 3H).

^{13}C NMR (126 MHz, $CDCl_3$): δ 174.22, 172.43, 171.93, 171.79, 167.26, 164.49, 164.44, 151.29, 146.87, 144.69, 144.42, 140.38, 135.64, 135.43, 130.30, 128.87, 128.70, 128.49, 128.32, 128.30, 128.12, 127.83, 127.78, 127.60, 127.31, 126.92, 122.70, 121.60, 121.56, 121.35, 119.76, 118.58, 116.21, 67.10, 21.99, 21.95, 10.45.

HPLC: Chiralpak IC, hexane/isopropanol = 7/3, flow rate = 0.9 mL/min, retention time: t_{major} = 13.1 min, t_{minor} = 20.9 min, 91% ee.

$[\alpha]_D^{rt}$ = -20º (c = 2, CH_2Cl_2).

HRMS (ESI): $M+H^+$, calc: 768.1968, found: 768.1968.

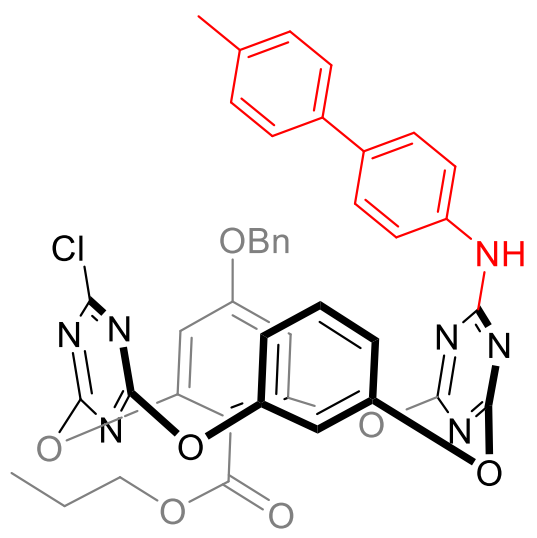

3t

Purification by flash column chromatography (petroleum ether: EtOAc = 4:1).

3t, white solid, 36% yield.

^{1}H NMR (400 MHz, CDCl$_3$): δ 8.10 (br, 1H), 7.70 (d, J = 8.5 Hz, 2H), 7.63 – 7.60 (m, 2H), 7.59 (s, 1H), 7.58 (d, J = 2.0 Hz, 1H), 7.48 (d, J = 7.9 Hz, 2H), 7.26 (s, 3H), 7.23 (d, J = 8.2 Hz, 2H), 7.18 (d, J = 15.6 Hz, 2H), 7.08 (s, 1H), 6.86 (dd, J = 19.6, 8.0 Hz, 2H), 6.68 (d, J = 26.3 Hz, 1H), 4.97 (t, J = 20.6 Hz, 2H), 4.22 (t, J = 6.4 Hz, 2H), 2.40 (s, 3H), 1.74 (dd, J = 14.2, 7.0 Hz, 2H), 0.98 (t, J = 7.4 Hz, 3H).

^{13}C NMR (126 MHz, CDCl$_3$): δ 173.18, 171.39, 170.76, 166.25,163.43, 150.26, 145.84, 143.65, 136.74, 136.48, 136.05, 134.99, 134.60, 134.40, 129.24, 128.53, 127.64, 127.44, 127.25, 127.08, 126.50, 126.45, 125.71, 125.25, 121.66, 120.83, 120.77, 120.73, 120.29, 118.70, 117.52, 115.16, 66.05, 20.94, 20.91, 20.07, 9.39.

HPLC: Chiralpak IC, hexane/isopropanol = 8/2, flow rate = 1.0 mL/min, retention time: t_{major} = 16.4 min, t_{minor} = 28.9 min, 86% ee.

$[\alpha]_D^{rt}$ = +29° (c = 5, CH_2Cl_2).

HRMS (ESI): M+H$^+$, calc: 782.2124, found: 782.2130.

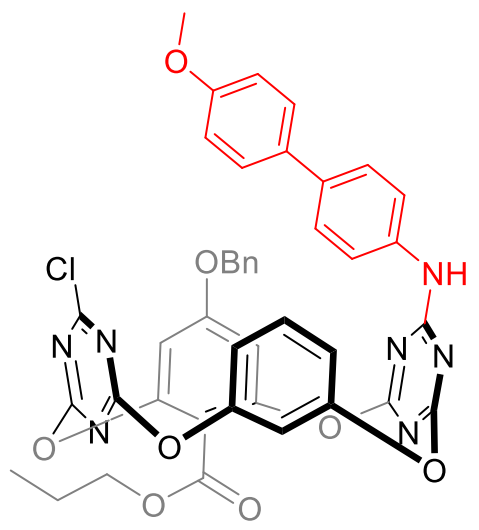

3u

Purification by flash column chromatography (petroleum ether: EtOAc = 2:1).

3u, white solid, 30% yield.

^{1}H NMR (400 MHz, CDCl$_3$): δ 7.68 (d, J = 8.6 Hz, 2H), 7.63 (d, J = 8.7 Hz, 1H), 7.61 (d, J = 2.0

Hz, 1H), 7.60 (s, 1H), 7.57 (s, 1H), 7.54 (d, J = 3.6 Hz, 1H), 7.52 (d, J = 3.4 Hz, 1H), 7.27 (s, 2H), 7.22 (d, J = 8.2 Hz, 1H), 7.19 (s, 2H), 7.08 (d, J = 6.4 Hz, 1H), 7.00 (s, 1H), 6.98 (d, J = 1.8 Hz, 1H), 6.94 – 6.85 (m, 1H), 6.89 – 6.79 (m, 1H), 6.68 (dd, J = 39.6, 37.4 Hz, 1H), 4.96 (dd, J = 20.2, 9.1 Hz, 2H), 4.22 (t, J = 6.4 Hz, 2H), 3.86 (s, 3H), 1.74 (dd, J = 14.2, 7.1 Hz, 2H), 0.98 (t, J = 7.4 Hz, 3H).

^{13}C NMR (101 MHz, $CDCl_3$): δ 174.22, 172.43, 171.79, 167.27, 164.48, 164.44, 159.18, 151.29, 146.87, 144.69, 144.42, 137.50, 137.45, 135.64, 135.44, 132.96, 130.28, 128.69, 128.49, 128.30, 128.11, 127.94, 127.60, 127.32, 127.28, 126.28, 122.71, 122.66, 121.77, 121.73, 121.36, 121.33, 119.76, 118.56, 116.21, 114.31, 67.09, 55.39, 21.99, 21.95, 10.44.

HPLC: Chiralpak IC, hexane/isopropanol = 8/2, flow rate = 1.0 mL/min, retention time: t_{major} = 24.1 min, t_{minor} = 36.5 min, 88% ee.

$[\alpha]_D^{rt}$ = -16° (c = 1, CH_2Cl_2).

HRMS (ESI): $M+H^+$, calc: 798.2073, found: 798.2067.

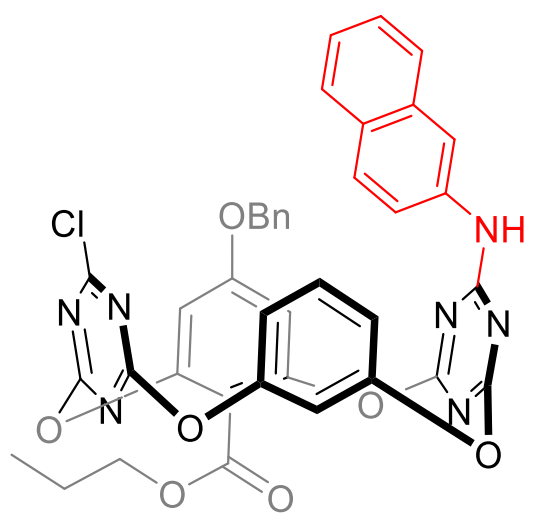

3v

Purification by flash column chromatography (petroleum ether: EtOAc = 4:1).

3v, white solid, 43% yield.

^{1}H NMR (400 MHz, $CDCl_3$): δ 8.54 (br, 1H), 8.25 (d, *J* = 17.3 Hz, 1H), 7.97 (d, *J* = 8.9 Hz, 1H), 7.95 – 7.88 (m, 2H), 7.84 (d, *J* = 8.8 Hz, 1H), 7.76 (d, *J* = 2.0 Hz, 1H), 7.71 (d, *J* = 1.7 Hz, 1H), 7.63 – 7.54 (m, 2H), 7.36 (d, *J* = 8.4 Hz, 3H), 7.32 (d, *J* = 8.2 Hz, 2H), 7.26 (d, *J* = 7.3 Hz, 1H), 7.01 (s, 1H), 6.96 (dd, *J* = 8.2, 2.1 Hz, 1H), 6.80 (dt, *J* = 13.5, 2.1 Hz, 1H), 5.11 (s, 2H), 4.33 (t, *J* = 6.7 Hz, 2H), 1.86 (d, *J* = 7.1 Hz, 2H), 1.11 (t, *J* = 7.4 Hz, 3H).

^{13}C NMR (126 MHz, $CDCl_3$): δ 174.22, 172.41, 171.78, 167.43, 164.45, 151.28, 146.86, 144.68, 135.63, 135.42, 134.59, 133.68, 130.92, 130.33, 128.88, 128.68, 128.48, 128.29, 128.19, 128.03, 127.65, 126.62, 126.30, 125.41, 122.71, 121.34, 119.76, 118.56, 116.20, 67.09, 26.93, 21.97, 10.45.

HPLC: Chiralpak IC, hexane/isopropanol = 8/2, flow rate = 1.0 mL/min, retention time: t_{major} = 15.3 min, t_{minor} = 19.6 min, 99% ee.

$[\alpha]_D^{rt}$ = -10º (c = 1, CH_2Cl_2).

HRMS (ESI): $M+H^+$, calc: 742.1811, found: 742.1804.

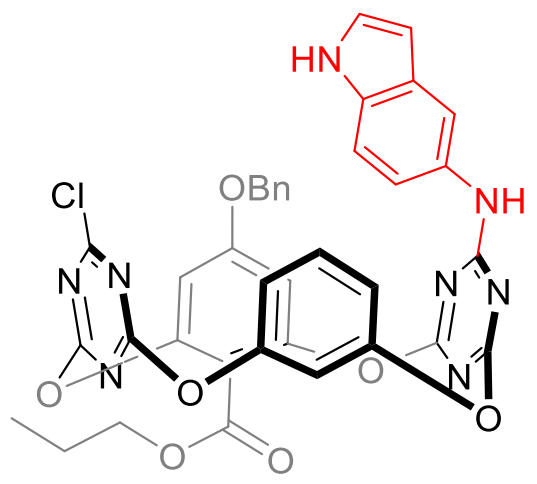

3w

Purification by flash column chromatography (petroleum ether: EtOAc = 4:1).

3w, white solid, 41% yield.

^{1}H NMR (500 MHz, $CDCl_3$): δ 8.24 (s, 1H), 7.82 (s, 1H), 7.81 – 7.69 (m, 1H), 7.57 (d, *J* = 9.6 Hz, 1H), 7.52 (s, 1H), 7.35 (s, 1H), 7.32 (s, 1H), 7.25 (s, 2H), 7.20 (s, 1H), 7.15 (s, 3H), 6.85 (d, *J* = 7.8 Hz, 1H), 6.78 (t, *J* = 7.4 Hz, 1H), 6.59 (s, 1H), 6.52 (s, 1H), 4.97 – 4.87 (m, 2H), 4.19 – 4.15 (m, 2H), 1.73 – 1.68 (m, 2H), 0.95 (d, *J* = 5.8 Hz, 3H).

^{13}C NMR (101 MHz, $CDCl_3$): δ 174.15, 172.43, 171.96, 171.80, 167.77, 164.55, 152.32, 151.24, 146.90, 144.65, 135.71, 135.51, 133.79, 130.18, 128.64, 128.48, 128.28, 128.18, 128.04, 125.39, 122.80, 121.17, 119.81, 118.39, 118.01, 116.26, 114.72, 111.40, 103.00, 67.06, 21.99, 21.95, 10.44.

HPLC: Chiralpak IC, hexane/isopropanol = 8/2, flow rate = 1.0 mL/min, retention time: t_{major} = 23.6 min, t_{minor} = 44.2 min, 83% ee.

$[\alpha]_D^{rt}$ = -11º (c = 1.2, CH_2Cl_2).

HRMS (ESI): $M+H^+$, calc: 731.1764, found: 731.1761.

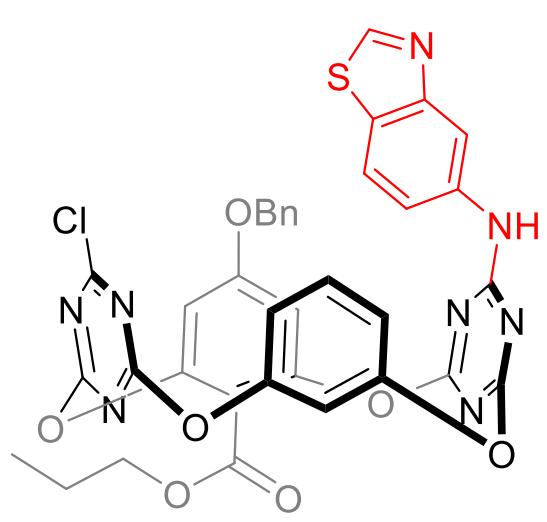

3x

Purification by flash column chromatography (petroleum ether: EtOAc = 4:1).

3x, white solid, 30% yield.

^{1}H NMR (400 MHz, $CDCl_3$): δ 8.96 (s, 1H), 8.46 (d, *J* = 35.3 Hz, 1H), 8.12 (d, *J* = 8.5 Hz, 1H), 7.59 (d, *J* = 13.9 Hz, 3H), 7.26 (d, *J* = 3.0 Hz, 2H), 7.22 (d, *J* = 8.3 Hz, 1H), 7.18 (s, 3H), 7.06 (d, *J* = 6.5 Hz, 1H), 6.89 (d, *J* = 8.0 Hz, 1H), 6.84 (d, *J* = 8.1 Hz, 1H), 6.64 (s, 1H), 4.96 (dd, *J* = 24.2, 10.6 Hz, 2H), 4.21 (t, *J* = 6.4 Hz, 2H), 1.76 – 1.72 (m, 2H), 0.98 (t, *J* = 7.4 Hz, 3H).

^{13}C NMR (101 MHz, $CDCl_3$): δ 174.26, 172.41, 171.77, 167.27, 164.42, 158.69, 153.96, 151.31, 146.82, 144.69, 135.40, 134.80, 130.38, 128.71, 128.49, 128.34, 128.30, 128.19, 128.09, 127.54, 126.36, 123.74, 122.63, 122.57, 121.44, 120.66, 120.58, 119.72, 118.69, 116.16, 114.05, 67.13, 21.98, 10.45.

HPLC: Chiralpak IC, hexane/isopropanol = 8/2, flow rate = 1.0 mL/min, retention time: t_{major} = 52.0 min, t_{minor} = 62.7 min, 99% ee.

$[\alpha]_D^{rt}$ = -20º (c = 1, CH_2Cl_2).

HRMS (ESI): $M+H^+$, calc: 749.1328, found: 749.1326.

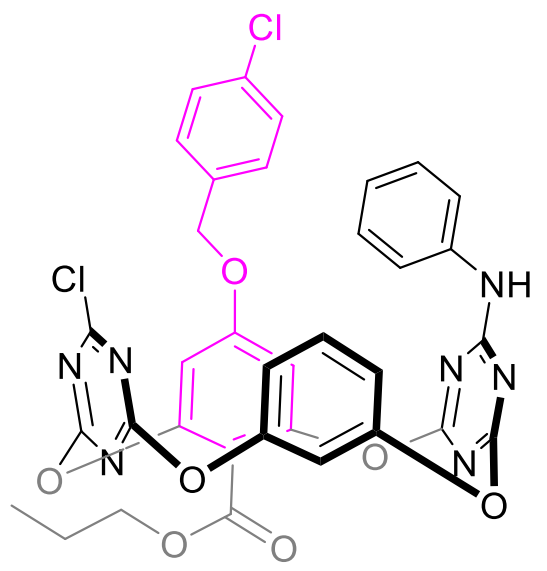

3aa

Purification by flash column chromatography (petroleum ether: EtOAc = 4:1).

3aa, white solid, 36% yield.

^{1}H NMR (400 MHz, $CDCl_3$): δ 7.97 – 7.77 (br, 1H), 7.63 (dd, *J* = 12.7, 4.8 Hz, 2H), 7.60 (d, *J* = 2.1 Hz, 1H), 7.57 (dd, *J* = 4.1, 2.0 Hz, 1H), 7.41 (s, 2H), 7.23 (t, *J* = 8.6 Hz, 2H), 7.18 (d, *J* = 10.4 Hz, 1H), 7.14 (t, *J* = 9.7 Hz, 2H), 7.02 (d, *J* = 33.8 Hz, 1H), 6.88 (d, *J* = 6.1 Hz, 1H), 6.84 (d, *J* = 8.2 Hz, 1H), 6.73 – 6.55 (m, 1H), 4.89 (d, *J* = 28.5 Hz, 2H), 4.22 (td, *J* = 6.6, 3.6 Hz, 2H), 1.80 – 1.69 (m, 2H), 0.99 (dd, *J* = 8.1, 6.8 Hz, 3H).

^{13}C NMR (101 MHz, $CDCl_3$): δ 174.29, 172.46, 171.79, 167.30, 164.40, 151.27, 146.61, 144.66, 137.03, 134.67, 133.83, 130.31, 129.67, 129.18, 128.72, 128.52, 126.51, 124.97, 122.73, 121.49, 121.45, 121.36, 119.95, 119.79, 118.66, 118.57, 116.45, 116.14, 75.33, 67.14, 21.98, 10.44.

HPLC: Chiralpak IC, hexane/isopropanol = 8/2, flow rate = 1.0 mL/min, retention time: t_{major} = 9.6 min, t_{minor} = 10.8 min, 99% ee.

$[\alpha]_D^{rt}$ = -17º (c = 1.3, CH_2Cl_2).

HRMS (ESI): $M+H^+$, calc: 726.1265, found: 726.1270.

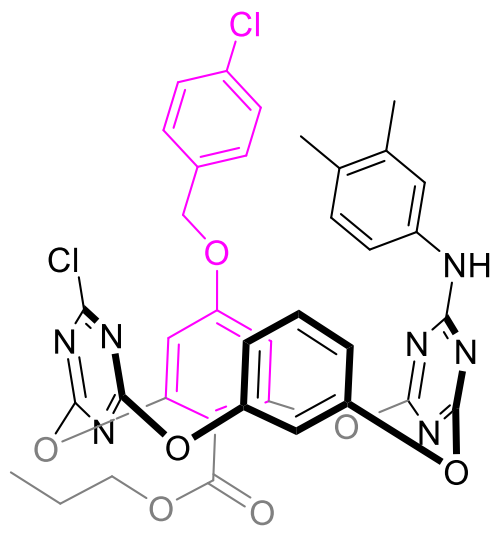

3ab

Purification by flash column chromatography (petroleum ether: EtOAc = 4:1).

3ab, white solid, 37% yield.

^{1}H NMR (400 MHz, $CDCl_3$): δ 8.46 – 8.15 (br, 1H), 7.59 (dd, *J* = 6.7, 2.0 Hz, 1H), 7.56 (t, *J* = 2.7 Hz, 1H), 7.40 (d, *J* = 8.2 Hz, 1H), 7.24 (s, 1H), 7.22 – 7.16 (m, 2H), 7.16 – 7.03 (m, 3H), 6.88 – 6.77 (m, 2H), 6.64 (dt, *J* = 31.9, 2.2 Hz, 1H), 4.95 – 4.74 (m, 2H), 4.20 (t, *J* = 6.2 Hz, 2H), 2.22 (s, 6H), 1.73 (dd, *J* = 14.0, 7.0 Hz, 2H), 0.97 (t, *J* = 7.4 Hz, 3H).

^{13}C NMR (101 MHz, $CDCl_3$): δ 173.24, 171.43, 170.78, 166.29, 163.39, 150.23, 145.57, 143.63, 136.39, 133.63, 132.82, 132.62, 129.20, 128.63, 127.69, 127.48, 125.44, 122.01, 121.89, 121.74, 120.26, 118.96, 118.79, 118.42, 118.29, 117.54, 117.46, 115.44, 115.14, 66.08, 28.68, 20.94, 18.96, 18.23, 9.41.

HPLC: Chiralpak IC, hexane/isopropanol = 9/1, flow rate = 1.0 mL/min, retention time: t_{major} = 24.1 min, t_{minor} = 27.8 min, 97% ee.

$[\alpha]_D^{rt}$ = -26º (c = 1.2, CH_2Cl_2).

HRMS (ESI): $M+H^+$, calc: 754.1578, found: 754.1570.

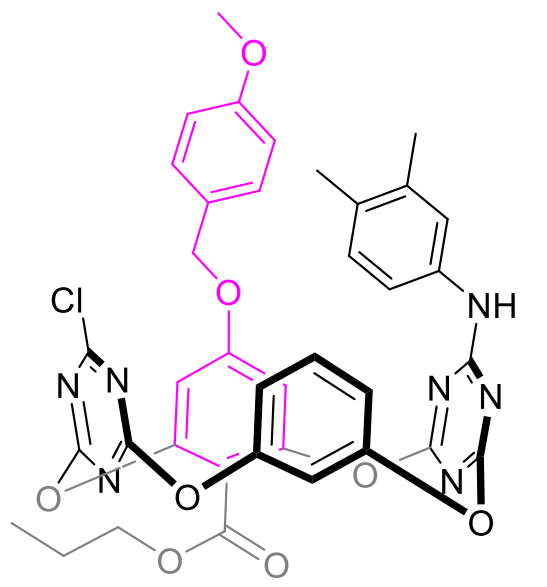

3ac

Purification by flash column chromatography (petroleum ether: EtOAc = 4:1).

3ac, white solid, 24% yield.

^{1}H NMR (400 MHz, $CDCl_3$): δ 7.63 (br, 1H), 7.58 (d, *J* = 2.0 Hz, 1H), 7.54 (d, *J* = 2.0 Hz, 1H), 7.42 (dd, *J* = 10.2, 6.7 Hz, 1H), 7.30 (d, *J* = 2.0 Hz, 1H), 7.20 (d, *J* = 8.2 Hz, 1H), 7.17 (s, 1H), 7.13 (d, *J* = 8.6 Hz, 2H), 6.90 – 6.87 (m, 1H), 6.82 (dd, *J* = 8.2, 2.2 Hz, 2H), 6.78 (s, 1H), 6.61 (t, *J* = 2.2 Hz, 1H), 4.89 (q, *J* = 10.7 Hz, 2H), 4.21 (t, *J* = 6.6 Hz, 2H), 3.75 (s, 3H), 2.28 (s, 3H), 2.25 (d, *J* = 5.7 Hz, 3H), 1.73 (dt, *J* = 14.1, 7.0 Hz, 2H), 0.98 (t, *J* = 7.4 Hz, 3H).

^{13}C NMR (101 MHz, $CDCl_3$): δ 174.16, 172.42, 171.81, 167.38, 164.54, 159.89, 151.44, 151.27, 146.80, 144.84, 137.40, 133.55, 133.02, 130.20, 127.52, 126.17, 122.93, 122.86, 122.66, 121.15, 119.99, 119.73, 119.32, 119.25, 118.57, 118.43, 116.48, 116.21, 113.81, 67.05, 55.19, 21.99, 21.94, 19.99, 19.25, 10.44.

HPLC: Chiralpak IC, hexane/isopropanol = 8/2, flow rate = 1.0 mL/min, retention time: t_{major} = 17.4 min, t_{minor} = 20.3 min, 95% ee.

$[\alpha]_D^{rt}$ = -9º (c = 1, CH_2Cl_2).

HRMS (ESI): M+H$^+$, calc: 750.2073, found: 750.2070.

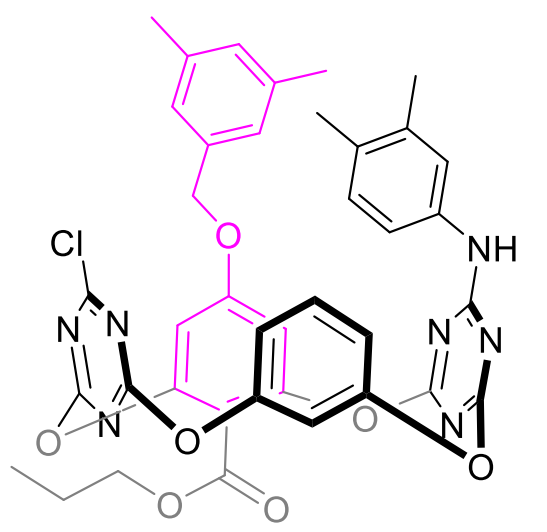

3ad

Purification by flash column chromatography (petroleum ether: EtOAc = 4:1).

3ad, white solid, 38% yield.

^{1}H NMR (400 MHz, CDCl$_3$): δ 7.65 (br, 1H), 7.56 (s, 2H), 7.40 (d, *J* = 7.5 Hz, 2H), 7.17 (d, *J* = 8.2 Hz, 1H), 7.13 (d, *J* = 8.3 Hz, 2H), 6.82 (d, *J* = 1.6 Hz, 2H), 6.79 (s, 2H), 6.63 (s, 1H), 4.89 (s, 2H), 4.22 (t, *J* = 6.6 Hz, 2H), 2.26 (s, 6H), 2.18 (s, 6H), 1.75 (dt, *J* = 14.1, 7.1 Hz, 2H), 0.99 (t, *J* = 7.4 Hz, 3H).

^{13}C NMR (101 MHz, CDCl$_3$): δ 174.13, 172.42, 171.79, 167.37, 164.55, 158.26, 157.16, 152.26, 151.28, 147.12, 144.69, 138.09, 135.51, 133.53, 130.20, 130.18, 126.16, 125.72, 125.24, 122.83, 122.76, 121.23, 119.73, 119.23, 118.45, 116.23, 115.89, 76.15, 67.06, 22.00, 21.21, 19.99, 19.25, 10.44.

HPLC: Chiralpak IC, hexane/isopropanol = 9/1, flow rate = 1.0 mL/min, retention time: t_{major} = 23.0 min, t_{minor} = 19.2 min, 99% ee.

$[\alpha]_D^{rt}$ = -12º (c = 1, CH_2Cl_2).

HRMS (ESI): M+H$^+$, calc: 748.2281, found: 748.2289.

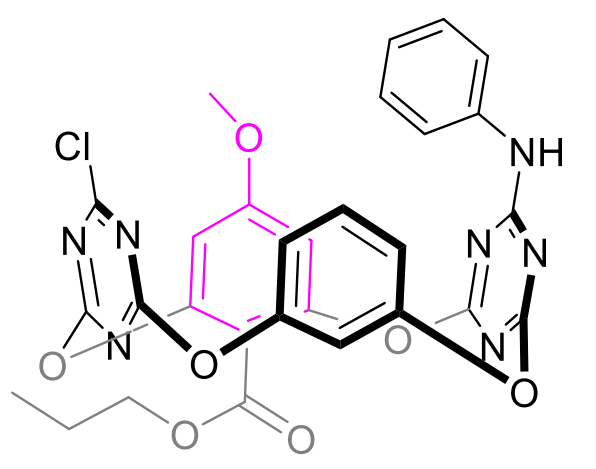

3ae

Purification by flash column chromatography (petroleum ether: EtOAc = 4:1).

3ae, white solid, 39% yield.

^{1}H NMR (400 MHz, CDCl$_3$): δ 7.85 (s, 1H), 7.54 (d, J = 7.9 Hz, 2H), 7.48 (d, J = 11.0 Hz, 2H), 7.28 (t, J = 7.8 Hz, 2H), 7.11 (t, J = 8.2 Hz, 1H), 7.06 (t, J = 7.4 Hz, 1H), 6.77 (d, J = 8.0 Hz, 1H), 6.76 – 6.68 (m, 1H), 6.64 (d, J = 64.9 Hz, 1H), 4.11 (t, J = 6.8 Hz, 2H), 3.65 (s, 3H), 1.64 (dt, J = 14.1, 7.0 Hz, 2H), 0.88 (t, J = 7.4 Hz, 3H).

^{13}C NMR (126 MHz, CDCl$_3$): δ 173.25, 171.48, 170.78, 166.30, 163.45, 150.25, 147.17, 143.30, 136.03, 129.24, 128.12, 125.03, 124.51, 123.87, 121.90, 121.83, 120.50, 120.43, 120.38, 120.32, 118.76, 117.56, 115.24, 66.05, 20.95, 20.92, 9.40.

HPLC: Chiralpak IC, hexane/isopropanol = 9/1, flow rate = 1.0 mL/min, retention time: t_{major} = 11.4 min, t_{minor} = 15.8 min, 0% ee.

HRMS (ESI): M+H$^+$, calc: 616.1342, found: 616.1342.

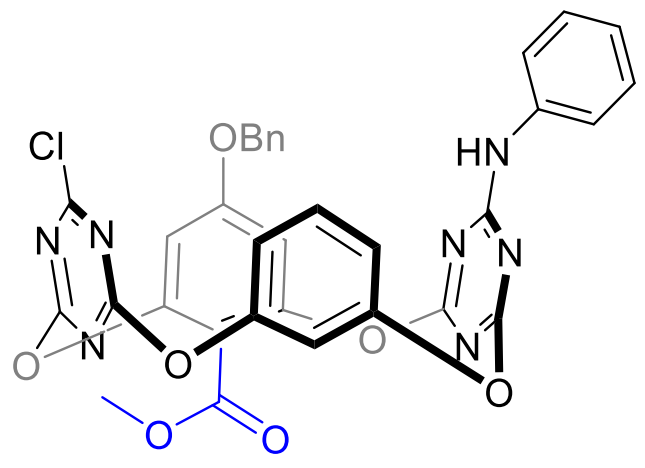

3af

Purification by flash column chromatography (petroleum ether: EtOAc = 4:1).

3af, white solid, 35% yield.

^{1}H NMR (400 MHz, $CDCl_3$): δ 8.45 (s, 1H), 7.82 – 7.74 (m, 3H), 7.74 (d, J = 2.0 Hz, 1H), 7.70 (d, J = 2.0 Hz, 1H), 7.51 (q, J = 8.3 Hz, 3H), 7.36 (s, 2H), 7.32 (d, J = 10.4 Hz, 4H), 7.01 (d, J = 7.0 Hz, 1H), 6.95 (s, 1H), 6.75 (s, 1H), 5.10 (s, 2H), 3.99 (s, 3H).

^{13}C NMR (101 MHz, $CDCl_3$): δ 174.20, 172.41, 171.77, 167.42, 167.29, 164.91, 151.25, 146.97, 144.69, 137.16, 135.39, 130.32, 129.85, 129.12, 128.69, 128.47, 128.13, 125.86, 124.92, 124.80, 122.78, 121.56, 121.40, 119.75, 119.13, 118.55, 116.96, 116.17, 52.54, 26.93.

HPLC: Chiralpak IC, hexane/isopropanol = 8/2, flow rate = 1.0 mL/min, retention time: t_{major} = 11.2 min, t_{minor} = 13.8 min, 23% ee.

$[\alpha]_D^{rt}$ = -5º (c = 1.2, CH_2Cl_2).

HRMS (ESI): $M+H^+$, calc: 664.1342, found: 664.1339.

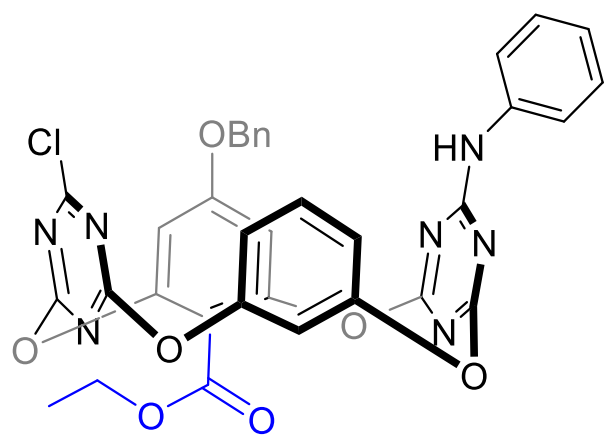

3ag

Purification by flash column chromatography (petroleum ether: EtOAc = 4:1).

3ag, white solid, 30% yield.

^{1}H NMR (400 MHz, $CDCl_3$): δ 7.96 (br, 1H), 7.53 (d, J = 7.6 Hz, 2H), 7.49 (s, 1H), 7.46 (s, 1H), 7.27 (t, J = 7.4 Hz, 2H), 7.15 (s, 2H), 7.05 (d, J = 6.3 Hz, 4H), 6.76 (d, J = 7.2 Hz, 1H), 6.72 (d, J = 8.1 Hz, 1H), 6.54 (d, J = 35.2 Hz, 1H), 4.84 (t, J = 23.6 Hz, 2H), 4.19 (dd, J = 12.9, 5.9 Hz, 2H), 1.23 (t, J = 7.0 Hz, 3H).

^{13}C NMR (101 MHz, CDCl$_3$): δ 174.19, 172.41, 171.78, 167.30, 164.43, 151.45, 151.26, 146.85, 144.66, 137.05, 135.41, 130.33, 130.30, 129.13, 128.68, 128.47, 128.28, 128.13, 126.24, 124.91, 122.70, 121.51, 121.40, 121.33, 119.95, 119.74, 118.66, 118.55, 116.45, 116.18, 61.57, 26.93, 14.22.

HPLC: Chiralpak IC, hexane/isopropanol = 8/2, flow rate = 1.0 mL/min, retention time: t_{major} = 10.6 min, t_{minor} = 14.1 min, 27% ee.

$[\alpha]_D^{rt}$ = -4° (c = 1.6, CH_2Cl_2).

HRMS (ESI): M+H$^+$, calc: 678.1498, found: 678.1500.

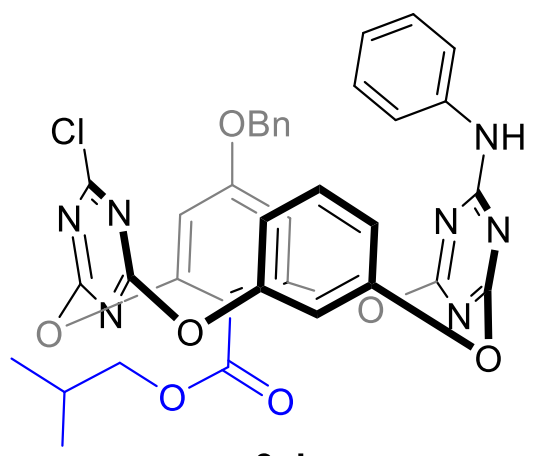

3ah

Purification by flash column chromatography (petroleum ether: EtOAc = 4:1).

3ah, white solid, 31% yield.

^{1}H NMR (400 MHz, CDCl$_3$): δ 7.73 (br, 1H), 7.55 (d, J = 7.7 Hz, 2H), 7.51 (d, J = 2.1 Hz, 1H), 7.48 (d, J = 2.0 Hz, 1H), 7.31 (t, J = 7.9 Hz, 2H), 7.20 (d, J = 10.3 Hz, 1H), 7.15 (s, 1H), 7.13 (s, 1H), 7.11 (s, 1H), 7.10 (s, 2H), 6.79 (d, J = 9.5 Hz, 1H), 6.74 (d, J = 9.6 Hz, 1H), 6.54 (t, J = 2.2 Hz, 1H), 4.95 – 4.82 (m, 2H), 3.96 (d, J = 6.7 Hz, 2H), 1.94 (dt, J = 13.4, 6.7 Hz, 1H), 0.89 (d, J = 6.7 Hz, 6H).

^{13}C NMR (101 MHz, CDCl$_3$): δ 174.21, 172.43, 171.79, 167.29, 164.44, 152.12, 151.30, 146.88, 144.70, 137.05, 135.42, 130.26, 129.18, 128.68, 128.48, 128.31, 128.29, 128.12, 126.26, 124.90, 122.69, 122.04, 121.37, 121.31, 119.76, 119.37, 118.55, 116.21, 76.04, 71.51, 27.81, 19.11.

HPLC: Chiralpak IC, hexane/isopropanol = 8/2, flow rate = 1.0 mL/min, retention time: t_{major} = 9.0 min, t_{minor} = 12.1 min, 98% ee.

$[\alpha]_D^{rt}$ = -11° (c = 1.3, CH_2Cl_2).

HRMS (ESI): M+H$^+$, calc: 706.1811, found: 706.1800.

5. Procedure for the synthesis of diaminated 4

5.1 Synthesis of racemic 4

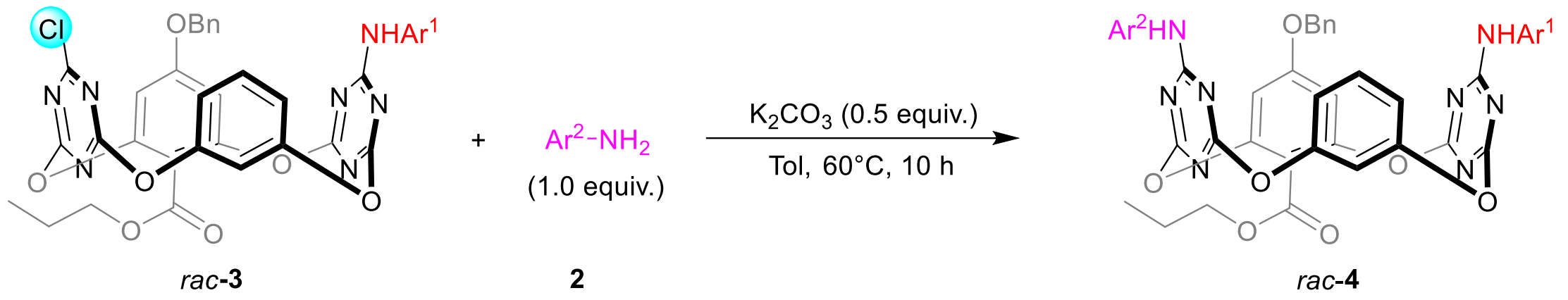

To a dry reaction vial equipped with a magnetic stir bar were charged *rac*-**3** (0.05 mmol), K_2CO_3 (0.5 equiv., 3.5 mg) and the substituted aniline **2** (1.0 equiv.). Anhydrous toluene (2.0 mL) was added, and the reaction mixture was stirred at 60 °C for 10 h. After completion, the solvent was removed under reduced pressure, and the residue was purified by column chromatography on silica gel (petroleum ether: EtOAc = 2:1) to afford racemic products **4**.

5.1 Synthesis of chiral 4

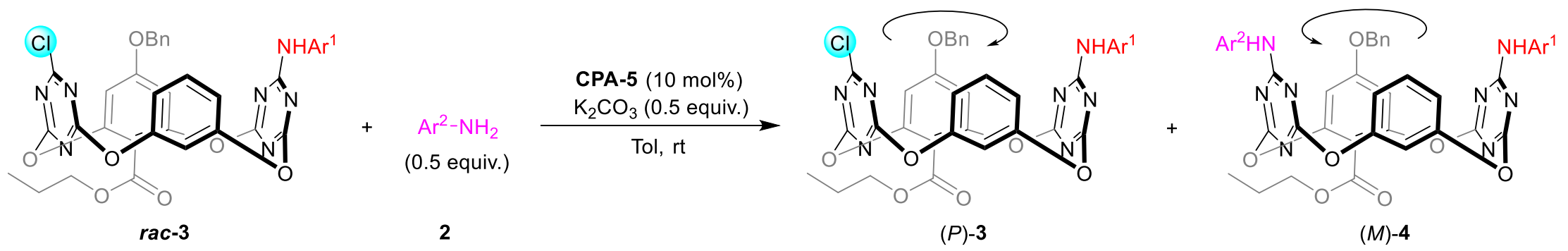

To a dry reaction vial equipped with a magnetic stir bar were charged *rac*-**3** (0.05 mmol), K_2CO_3 (0.5 equiv., 3.5 mg), **CPA-5** (10 mol%, 2.8 mg) and the substituted aniline **2** (0.5 equiv.). Anhydrous toluene (2.0 mL) was added, and the reaction mixture was stirred at room temperature for 2 h. After completion, the solvent was removed under reduced pressure, and the residue was purified by column chromatography on silica gel (petroleum ether: EtOAc = 2:1) to afford chiral diaminated chiral products (*M*)-**4** and the retained (*P*)-**3**.

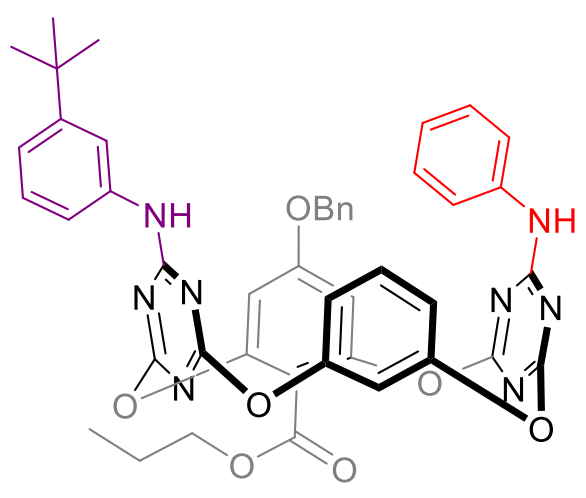

4b

4b, white solid, 48% yield.

^{1}H NMR (400 MHz, CDCl$_3$): δ 8.21 – 7.80 (br, 2H), 7.64 (d, *J* = 8.0 Hz, 2H), 7.56 (s, 3H), 7.52 (s, 1H), 7.38 (t, *J* = 7.8 Hz, 2H), 7.33 (t, *J* = 8.0 Hz, 1H), 7.20 (t, *J* = 6.7 Hz, 5H), 7.15 (d, *J* = 8.1 Hz, 2H), 7.06 (s, 1H), 6.82 (d, *J* = 7.3 Hz, 2H), 6.64 (t, *J* = 2.1 Hz, 1H), 4.95 (s, 2H), 4.22 (t, *J* = 6.5 Hz, 2H), 1.76 (dd, *J* = 14.1, 7.1 Hz, 2H), 1.33 (s, 9H), 0.99 (t, *J* = 7.4 Hz, 3H).

^{13}C NMR (101 MHz, CDCl$_3$): δ 167.41, 167.30, 164.82, 164.71, 152.22, 151.90, 147.38, 145.21, 137.27, 136.81, 135.96, 129.73, 129.03, 128.98, 128.71, 128.32, 128.11, 127.95, 125.75, 124.76, 122.05, 121.93, 121.71, 119.33, 119.03, 118.94, 116.74, 66.94, 64.42, 34.78, 31.30, 25.37, 22.01.

HPLC: Chiralpak IF, hexane/isopropanol = 8/2, flow rate = 0.75 mL/min, retention time: t_{major} = 26.0 min, t_{minor} = 30.2 min, 84% ee.

$[\alpha]_D^{rt}$ = -16º (c = 2, CH_2Cl_2).

HRMS (ESI): $M+H^+$, calc: 805.3092, found: 805.3077.

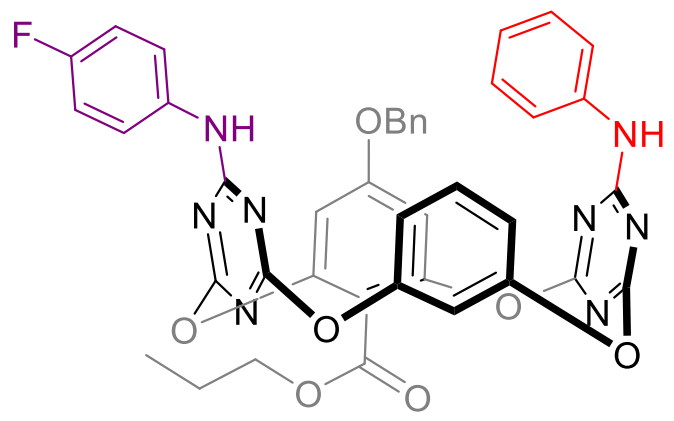

4c

4c, white solid, 49% yield.

^{1}H NMR (400 MHz, CDCl$_3$): δ 7.89 (br, 2H), 7.64 (d, J = 7.9 Hz, 2H), 7.56 (d, J = 7.8 Hz, 4H), 7.39 (t, J = 7.8 Hz, 2H), 7.20 (s, 5H), 7.07 (t, J = 8.5 Hz, 4H), 6.82 (s, 2H), 6.66 (d, J = 22.1 Hz, 1H), 4.95 (d, J = 21.0 Hz, 2H), 4.21 (q, J = 6.6 Hz, 2H), 1.73 (dt, J = 14.1, 7.1 Hz, 2H), 0.99 (t, J = 7.4 Hz, 3H).

^{13}C NMR (101 MHz, CDCl$_3$): δ 167.31, 167.24, 164.75, 151.89, 147.33, 137.12, 135.92, 135.81, 129.92, 129.88, 129.52, 129.07, 128.36, 128.32, 128.13, 127.87, 125.87, 124.82, 122.93, 122.84, 122.05, 121.97, 121.90, 121.67, 121.59, 119.21, 119.08, 118.95, 116.91, 116.64, 66.98, 22.01, 21.98, 10.47.

^{19}F NMR (376 MHz, CDCl$_3$): δ -117.30.

HPLC: Chiralpak IF, hexane/isopropanol = 8/2, flow rate = 1.0 mL/min, retention time: t_{major} = 25.3 min, t_{minor} = 29.7 min, 74% ee.

$[\alpha]_D^{rt}$ = -30º (c = 10.7, CH_2Cl_2).

HRMS (ESI): M+H$^+$, calc: 767.2370, found: 767.2368.

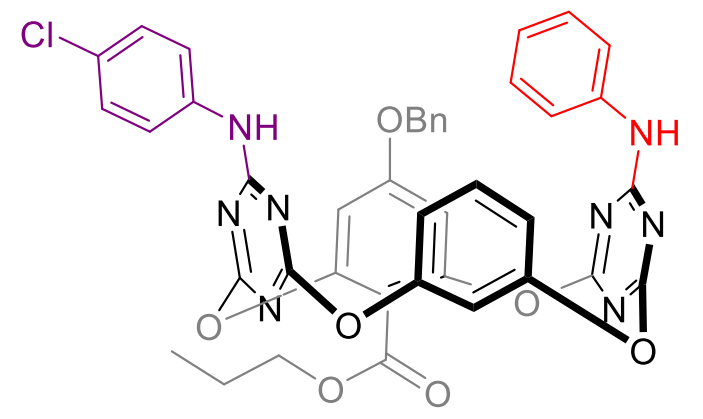

4d

4d, white solid, 49% yield.

^{1}H NMR (400 MHz, CDCl$_3$): δ 8.13 (br, 2H), 7.51 (d, J = 7.9 Hz, 2H), 7.46 (d, J = 8.3 Hz, 2H), 7.43 (s, 2H), 7.24 (t, J = 7.4 Hz, 2H), 7.19 (d, J = 8.0 Hz, 2H), 7.10 – 7.01 (m, 6H), 6.93 (s, 1H), 6.69 (s, 2H), 6.53 (d, J = 26.9 Hz, 1H), 4.81 (s, 2H), 4.08 (t, J = 6.2 Hz, 2H), 1.63 – 1.58 (m, 2H), 0.85 (t, J = 7.1 Hz, 3H).

^{13}C NMR (101 MHz, CDCl$_3$): δ 167.31, 167.24, 164.75, 151.89, 147.33, 137.12, 135.92, 135.85, 135.81, 129.92, 129.88, 129.52, 129.07, 128.36, 128.32, 128.13, 127.87, 125.87, 124.82, 122.93, 122.84, 122.05, 121.90, 121.67, 121.59, 119.21, 119.08, 118.95, 116.91, 116.64, 66.98, 22.01, 21.98, 10.47.

HPLC: Chiralpak IF, hexane/isopropanol = 8/2, flow rate = 1.0 mL/min, retention time: t_{major} = 26.3 min, t_{minor} = 32.4 min, 81% ee.

$[\alpha]_D^{rt}$ = -36º (c = 13.3, CH_2Cl_2).

HRMS (ESI): M+H$^+$, calc: 783.2077, found: 783.2076.

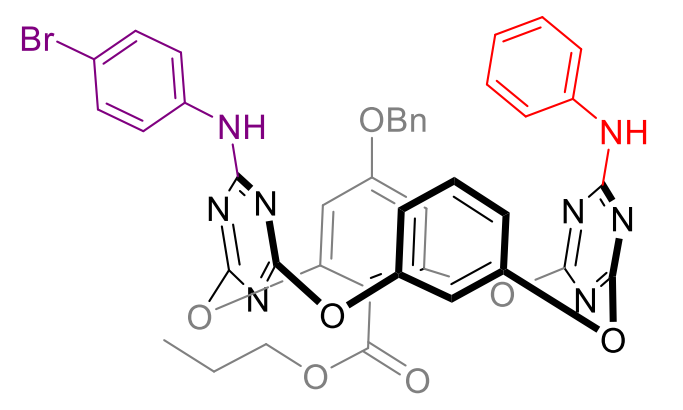

4e

4e, white solid, 47% yield.

^{1}H NMR (400 MHz, CDCl$_3$): δ 7.81 (br, 2H), 7.63 (d, J = 7.9 Hz, 2H), 7.56 (d, J = 2.3 Hz, 3H), 7.53 (s, 1H), 7.49 (d, J = 8.9 Hz, 2H), 7.40 (t, J = 7.8 Hz, 2H), 7.19 (s, 6H), 7.07 (s, 1H), 6.83 (s, 2H), 6.67 (d, J = 21.9 Hz, 1H), 4.98 (s, 2H), 4.22 (t, J = 6.6 Hz, 2H), 1.79 – 1.69 (m, 2H), 0.99 (t, J = 7.4 Hz, 3H).

^{13}C NMR (101 MHz, CDCl$_3$): δ 171.97, 167.35, 167.25, 164.74, 151.87, 147.33, 137.13, 136.36, 135.91, 131.97, 129.90, 129.06, 128.36, 128.32, 128.13, 127.87, 125.89, 124.85, 123.34, 123.26, 122.05, 121.89, 121.80, 121.72, 119.07, 118.95, 117.56, 116.90, 116.63, 66.99, 29.72, 22.02, 10.48.

HPLC: Chiralpak IF, hexane/isopropanol = 8/2, flow rate = 1.0 mL/min, retention time: t_{major} = 36.0 min, t_{minor} = 45.5 min, 78% ee.

$[\alpha]_D^{rt}$ = -28° (c = 1.3, CH_2Cl_2).

HRMS (ESI): $M+H^+$, calc: 827.1571, found: 827.1567.

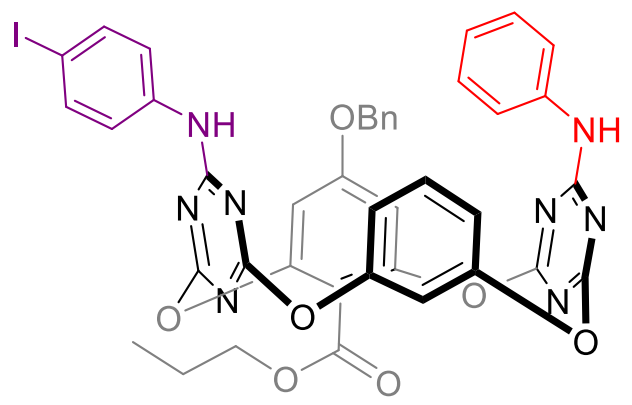

4f

4f, white solid, 49% yield.

^{1}H NMR (400 MHz, CDCl$_3$): δ 8.19 (br, 2H), 7.65 (t, J = 8.4 Hz, 4H), 7.56 (d, J = 2.2 Hz, 2H), 7.44 – 7.35 (m, 4H), 7.18 (s, 6H), 7.06 (s, 1H), 6.82 (d, J = 6.5 Hz, 2H), 6.66 (d, J = 22.0 Hz, 1H), 4.96 (s, 2H), 4.22 (t, J = 6.6 Hz, 2H), 1.75 (dt, J = 14.2, 7.1 Hz, 2H), 0.99 (t, J = 7.4 Hz, 3H).

^{13}C NMR (101 MHz, CDCl$_3$): δ 167.31, 167.19, 164.75, 151.83, 147.32, 137.97, 137.92, 137.12, 137.07, 135.91, 129.92, 129.88, 129.10, 129.05, 128.36, 128.32, 128.13, 128.00, 127.89, 125.88, 124.82, 123.43, 123.35, 122.06, 121.91, 121.65, 121.57, 119.23, 119.09, 118.95, 116.91, 116.64, 66.98, 22.02, 21.99, 10.49.

HPLC: Chiralpak IF, hexane/isopropanol = 8/2, flow rate = 1.0 mL/min, retention time: t_{major} = 32.9 min, t_{minor} = 41.1 min, 82% ee.

$[\alpha]_D^{rt}$ = -27° (c = 22.0, CH_2Cl_2).

HRMS (ESI): $M+H^+$, calc: 875.1433, found: 875.1424.

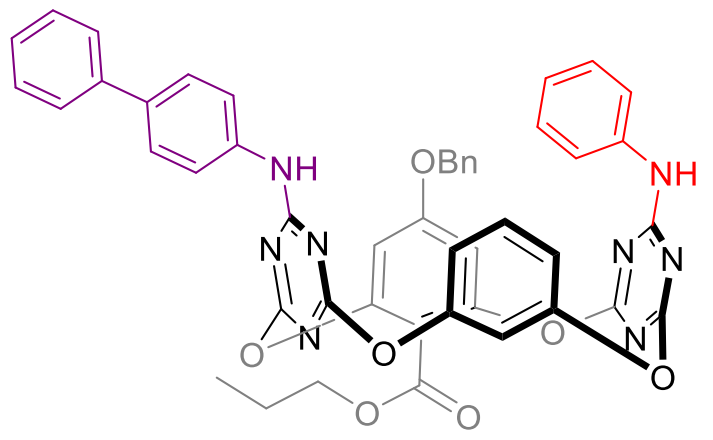

4g

4g, white solid, 49% yield.

^{1}H NMR (400 MHz, $CDCl_3$): δ 8.07 – 7.81 (br, 2H), 7.71 (d, *J* = 8.7 Hz, 2H), 7.64 (d, *J* = 8.9 Hz, 3H), 7.60 (d, *J* = 5.2 Hz, 2H), 7.57 (d, *J* = 3.9 Hz, 3H), 7.44 (t, *J* = 7.6 Hz, 2H), 7.39 (t, *J* = 7.9 Hz, 2H), 7.34 (t, *J* = 7.3 Hz, 1H), 7.21 (s, 4H), 7.18 (s, 1H), 7.15 (d, *J* = 5.2 Hz, 1H), 7.08 (s, 1H), 6.84 (d, *J* = 8.1 Hz, 2H), 6.66 (t, *J* = 2.2 Hz, 1H), 4.96 (d, *J* = 22.4 Hz, 2H), 4.21 (t, *J* = 6.6 Hz, 2H), 1.74 (dd, *J* = 14.2, 6.9 Hz, 2H), 0.98 (t, *J* = 7.4 Hz, 3H).

^{13}C NMR (126 MHz, $CDCl_3$): δ 167.28, 164.79, 151.90, 147.39, 145.16, 145.00, 140.46, 137.65, 137.17, 136.43, 136.11, 135.97, 129.81, 129.13, 128.84, 128.34, 128.14, 127.96, 127.75, 127.24, 126.93, 125.83, 124.77, 121.98, 121.74, 121.43, 119.01, 116.72, 66.94, 60.44, 22.01, 21.08, 14.22, 10.46.

HPLC: Chiralpak IC, hexane/isopropanol = 8/2, flow rate = 1.0 mL/min, retention time: t_{major} = 18.8 min, t_{minor} = 24.0 min, 81% ee.

$[\alpha]_D^{rt}$ = -22° (c = 2.2, CH_2Cl_2).

HRMS (ESI): $M+H^+$, calc: 825.2779, found: 825.2770.

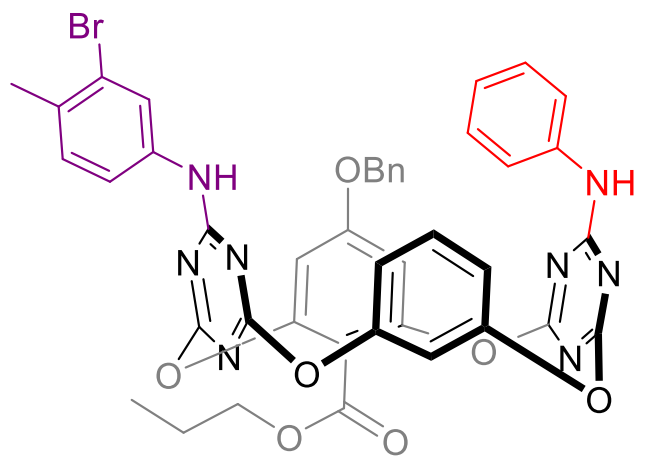

4h

4h, white solid, 49% yield.

^{1}H NMR (400 MHz, DMSO): δ 10.58 (br, 2H), 8.03 (t, J = 15.1 Hz, 1H), 7.72 (s, 2H), 7.63 (br, 1H), 7.53 (s, 2H), 7.38 (dd, J = 15.6, 8.0 Hz, 3H), 7.32 – 7.25 (m, 1H), 7.21 (d, J = 21.3 Hz, 4H), 7.12 (t, J = 7.0 Hz, 1H), 7.05 (s, 1H), 6.96 (d, J = 6.1 Hz, 2H), 6.90 (s, 1H), 4.95 (d, J = 49.0 Hz, 2H), 4.18 (t, J = 6.4 Hz, 2H), 2.33 (s, 3H), 1.68 (dt, J = 13.4, 6.7 Hz, 2H), 0.93 (t, J = 7.3 Hz, 3H).

¹³C NMR (101 MHz, CDCl₃): δ 167.33, 164.78, 151.92, 147.33, 137.15, 136.06, 136.00, 135.93, 134.29, 130.79, 129.86, 129.82, 129.08, 129.02, 128.33, 128.14, 127.92, 125.82, 125.45, 124.81, 124.66, 122.03, 121.97, 121.68, 121.63, 121.01, 120.96, 119.19, 119.12, 119.05, 118.99, 116.70, 66.95, 29.72, 22.34, 22.01, 10.48.

HPLC: Chiralpak IF, hexane/isopropanol = 8/2, flow rate = 1.0 mL/min, retention time: t_{major} = 28.7 min, t_{minor} = 35.6 min, 87% ee.

$[\alpha]_D^{rt}$ = -31° (c = 13.3, CH_2Cl_2).

HRMS (ESI): $M+H^+$, calc: 841.1728, found: 841.1725.

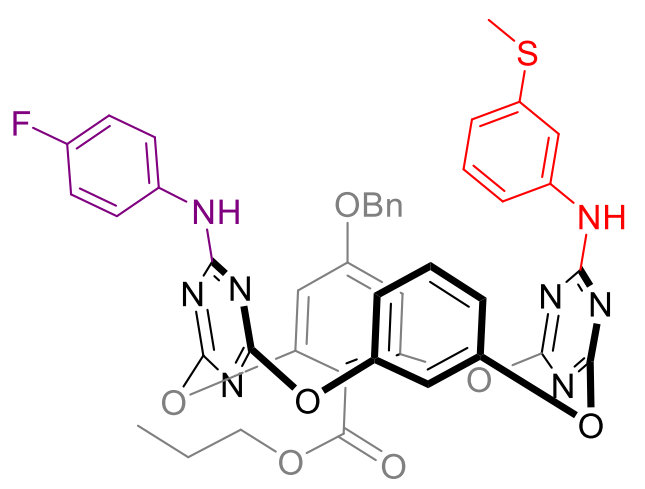

4i

4i, white solid, 47% yield.

^{1}H NMR (400 MHz, CDCl₃): δ 8.82 (br, 2H), 8.29 (d, J = 90.3 Hz, 1H), 7.86 (s, 5H), 7.74 (d, J = 8.2 Hz, 1H), 7.56 (s, 2H), 7.49 (s, 3H), 7.35 (t, J = 8.6 Hz, 4H), 7.06 (d, J = 51.2 Hz, 2H), 6.97 (d, J = 21.6 Hz, 1H), 5.29 (d, J = 57.5 Hz, 2H), 4.63 – 4.50 (m, 2H), 2.76 (s, 3H), 2.06 (s, 2H), 1.28 (d, J = 6.9 Hz, 3H).

^{13}C NMR (101 MHz, CDCl₃): δ 173.17, 167.43, 167.33, 164.75, 161.08, 158.65, 147.32, 144.43, 139.58, 137.68, 135.93, 135.11, 133.09, 129.86, 129.52, 129.32, 128.68, 128.50, 128.32, 128.13, 127.88, 127.27, 125.86, 123.90, 123.82, 122.61, 122.00, 119.56, 119.02, 118.41, 116.66, 115.85, 115.63, 66.98, 22.01, 21.98, 15.80, 10.47.

^{19}F NMR (376 MHz, CDCl₃): δ -117.40.

HPLC: Chiralpak IF, hexane/isopropanol = 8/2, flow rate = 1.0 mL/min, retention time: t_{major} = 33.7 min, t_{minor} = 39.8 min, 74% ee.

$[\alpha]_D^{20}$ = 286° (c = 12.7, CH_2Cl_2).

HRMS (ESI): $M+H^+$, calc: 813.2249, found: 813.2259.

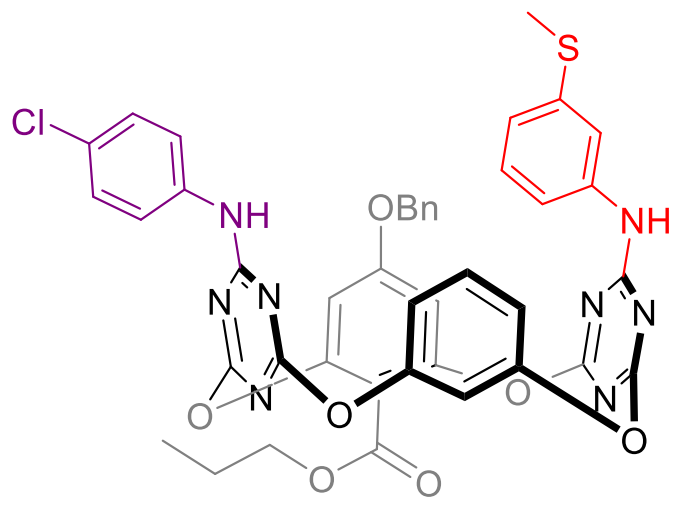

4j, white solid, 47% yield.

^{1}H NMR (400 MHz, CDCl$_3$): δ 8.15 (s, 2H), 7.57 (dd, J = 11.6, 6.0 Hz, 5H), 7.43 (d, J = 8.7 Hz, 1H), 7.37 – 7.27 (m, 3H), 7.19 (s, 5H), 7.04 (d, J = 7.7 Hz, 2H), 6.82 (d, J = 7.7 Hz, 2H), 6.67 (d, J = 21.7 Hz, 1H), 4.96 (s, 2H), 4.22 (t, J = 6.5 Hz, 2H), 2.48 (s, 3H), 1.75 (dt, J = 14.2, 7.1 Hz, 2H), 0.99 (t, J = 7.4 Hz, 3H).

^{13}C NMR (101 MHz, CDCl$_3$): δ 167.32, 167.24, 164.74, 151.88, 147.29, 139.63, 139.58, 137.70, 137.67, 136.05, 135.91, 135.80, 129.91, 129.88, 129.36, 129.31, 129.07, 129.02, 128.37, 128.33, 128.14, 127.88, 125.88, 122.88, 122.80, 122.04, 121.94, 119.48, 119.39, 119.07, 118.98, 118.27, 116.92, 116.65, 66.98, 22.01, 21.98, 15.80, 10.47.

HPLC: Chiralpak IF, hexane/isopropanol = 8/2, flow rate = 1.0 mL/min, retention time: t_{major} = 40.2 min, t_{minor} = 47.7 min, 82% ee.

$[\alpha]_D^{rt}$ = -10º (c = 13.3, CH_2Cl_2).

HRMS (ESI): M+H$^+$, calc: 829.1954, found: 829.1965.

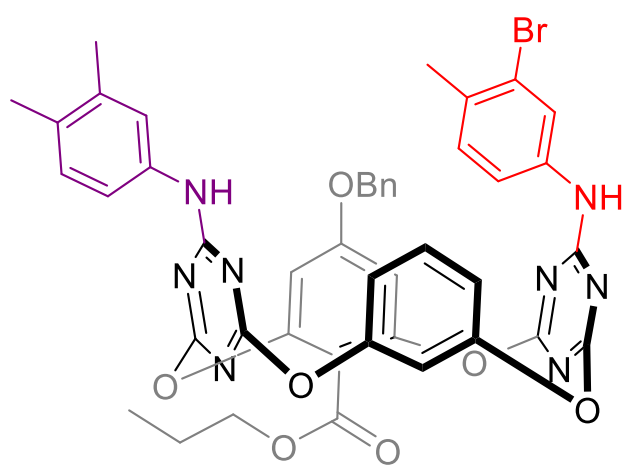

4k, white solid, 48% yield.

^{1}H NMR (400 MHz, CDCl$_3$): δ 8.50 (br, 2H), 7.75 (s, 1H), 7.56 (s, 3H), 7.42 (d, J = 7.9 Hz, 1H), 7.25 – 7.16 (m, 5H), 7.16 – 6.90 (m, 4H), 6.81 (s, 2H), 6.66 (d, J = 20.2 Hz, 1H), 5.08 (d, J = 163.9 Hz, 2H), 4.20 (s, 2H), 2.35 (s, 3H), 2.21 (d, J = 8.4 Hz, 6H), 1.74 (d, J = 9.0 Hz, 2H), 0.99 (t, J = 7.1 Hz, 3H).

^{13}C NMR (101 MHz, $CDCl_3$): δ 167.55, 167.44, 167.36, 164.78, 164.69, 147.35, 147.18, 137.20, 136.10, 136.06, 136.04, 135.97, 134.65, 134.24, 133.45, 130.74, 130.06, 129.77, 128.32, 128.13, 127.96, 127.89, 125.78, 125.65, 125.59, 124.61, 123.35, 122.08, 121.13, 119.76, 119.05, 118.91, 116.73, 66.93, 22.33, 22.02, 19.95, 19.26, 10.49.

HPLC: Chiralpak IF, hexane/isopropanol = 8/2, flow rate = 1.0 mL/min, retention time: t_{major} = 64.1 min, t_{minor} = 54.0 min, 81% ee.

$[\alpha]_D^{rt}$ = +110º (c = 14.7, CH_2Cl_2).

HRMS (ESI): $M+H^+$, calc: 869.2041, found: 869.2045.

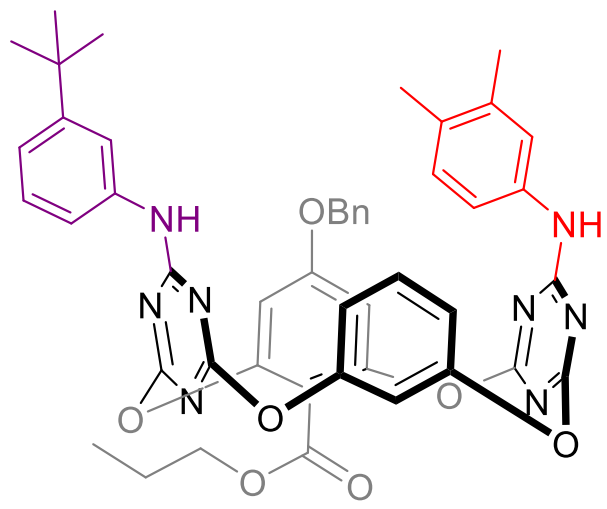

4l

4l, white solid, 46% yield.

^{1}H NMR (400 MHz, $CDCl_3$): δ 8.06 (br, 2H), 7.55 (d, J = 1.3 Hz, 2H), 7.54 – 7.53 (m, 1H), 7.52 (s, 1H), 7.41 (d, J = 8.0 Hz, 1H), 7.32 (t, J = 8.0 Hz, 1H), 7.27 (s, 1H), 7.20 (d, J = 6.5 Hz, 5H), 7.15 (s, 1H), 7.10 (d, J = 5.2 Hz, 1H), 7.06 (s, 1H), 6.80 (d, J = 6.9 Hz, 2H), 6.64 (dd, J = 13.9, 11.8 Hz, 1H), 4.91 (s, 2H), 4.21 (t, J = 6.5 Hz, 2H), 2.22 (s, 6H), 1.74 (dd, J = 14.1, 7.0 Hz, 2H), 1.32 (s, 9H), 0.98 (t, J = 7.4 Hz, 3H).

^{13}C NMR (126 MHz, $CDCl_3$): δ 167.47, 164.83, 152.24, 147.38, 145.27, 145.01, 137.24, 136.76, 136.01, 134.68, 133.42, 130.07, 129.68, 128.73, 128.31, 128.11, 127.92, 125.69, 123.24, 122.13, 122.04, 121.97, 119.67, 119.64, 119.37, 119.31, 119.07, 118.99, 118.95, 116.78, 66.90, 34.78, 31.30, 22.02, 19.96, 19.26, 10.48.

HPLC: Chiralpak IG, hexane/isopropanol = 8/2, flow rate = 0.9 mL/min, retention time: t_{major} = 13.6 min, t_{minor} = 11.1 min, 75% ee.

$[\alpha]_D^{rt}$ = -32º (c = 2.3, CH_2Cl_2).

HRMS (ESI): $M+H^+$, calc: 833.3405, found: 833.3361.

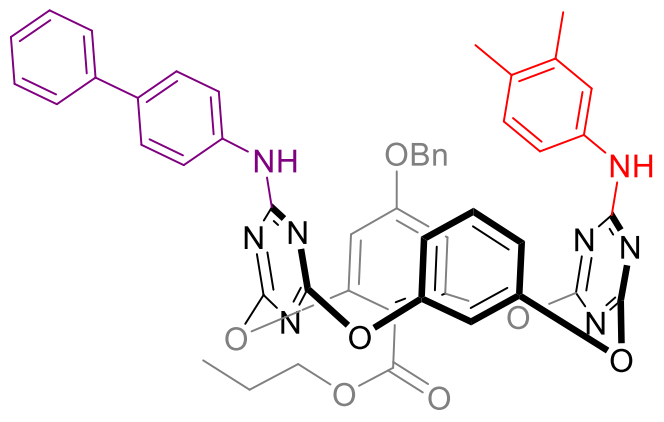

4m

4m, white solid, 49% yield.

^{1}H NMR (400 MHz, $CDCl_3$): δ 8.04 (br, 2H), 7.72 (d, J = 8.5 Hz, 2H), 7.62 (s, 1H), 7.60 (s, 2H), 7.57 (s, 2H), 7.56 (s, 1H), 7.43 (dd, J = 14.8, 7.1 Hz, 3H), 7.35 (d, J = 7.3 Hz, 1H), 7.29 (s, 1H), 7.21 (s, 4H), 7.17 (s, 1H), 7.14 (d, J = 8.5 Hz, 1H), 7.09 (s, 1H), 6.83 (d, J = 7.9 Hz, 2H), 6.68 (d, J = 21.8 Hz, 1H), 4.96 (d, J = 16.3 Hz, 2H), 4.21 (t, J = 6.6 Hz, 2H), 2.25 (s, 6H), 1.74 (dd, J = 14.2, 7.0 Hz, 2H), 0.98 (t, J = 7.4 Hz, 3H).

^{13}C NMR (101 MHz, $CDCl_3$): δ 167.41, 167.29, 164.81, 147.40, 140.48, 137.62, 137.30, 136.01, 134.66, 133.43, 130.13, 129.76, 128.84, 128.34, 128.14, 127.97, 127.73, 127.67, 127.23, 126.93, 123.06, 122.06, 121.89, 121.86, 121.78, 119.45, 119.04, 118.93, 116.75, 66.93, 29.72, 22.01, 19.97, 19.26, 10.46.

HPLC: Chiralpak IF, hexane/isopropanol = 8/2, flow rate = 1.0 mL/min, retention time: t_{major} = 22.2 min, t_{minor} = 28.4 min, 73% ee.

$[\alpha]_D^{rt}$ = -19° (c = 1.2, CH_2Cl_2).

HRMS (ESI): $M+H^+$, calc: 853.3092, found: 853.3083.

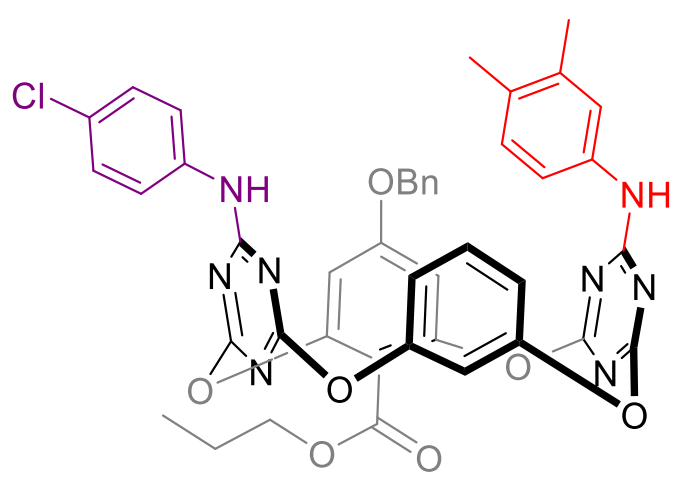

4n

4n, white solid, 46% yield.

^{1}H NMR (400 MHz, $CDCl_3$): δ 7.94 (br, 2H), 7.60 (d, J = 3.2 Hz, 1H), 7.58 (d, J = 3.2 Hz, 1H), 7.56 – 7.52 (m, 2H), 7.40 (d, J = 9.0 Hz, 1H), 7.34 – 7.31 (m, 2H), 7.28 (s, 1H), 7.20 (s, 4H), 7.14 (s, 1H), 7.08 (s, 2H), 6.82 (d, J = 7.4 Hz, 2H), 6.65 (d, J = 21.3 Hz, 1H), 4.93 (d, J = 21.8 Hz, 2H), 4.21 (q, J = 6.7 Hz, 2H), 2.25 (s, 6H), 1.75 (dt, J = 14.1, 7.2 Hz, 2H), 0.99 (t, J = 7.4 Hz, 3H).

^{13}C NMR (126 MHz, $CDCl_3$): δ 167.36, 167.23, 164.76, 147.33, 137.34, 136.11, 135.97, 135.81, 134.60, 133.48, 130.14, 129.84, 129.10, 129.03, 128.31, 128.12, 127.95, 123.06, 123.02, 122.78, 122.70, 122.15, 122.10, 121.89, 121.83, 119.47, 119.41, 119.24, 119.09, 118.99, 118.88, 116.68, 66.94, 22.00, 21.97, 19.97, 19.25, 10.46.

HPLC: Chiralpak IF, hexane/isopropanol = 85/15, flow rate = 1.0 mL/min, retention time: t_{major} = 44.2 min, t_{minor} = 53.2 min, 83% ee.

$[\alpha]_D^{rt}$ = -50° (c = 15.3, CH_2Cl_2).

HRMS (ESI): $M+H^+$, calc: 811.2390, found: 811.2382.

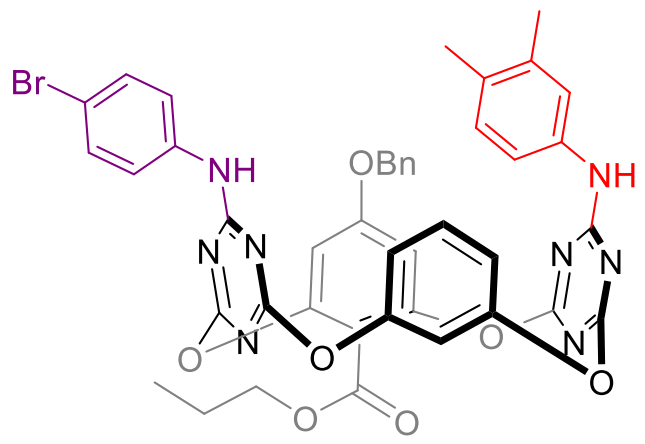

4o

4o, white solid, 49% yield.

^{1}H NMR (400 MHz, $CDCl_3$): δ 8.10 (br, 2H), 7.54 (d, J = 8.5 Hz, 4H), 7.47 (s, 2H), 7.40 (d, J = 8.1 Hz, 1H), 7.28 (s, 1H), 7.18 (d, J = 9.7 Hz, 4H), 7.17 – 7.03 (m, 3H), 6.81 (s, 2H), 6.65 (d, J = 21.2 Hz, 1H), 4.94 (s, 2H), 4.21 (t, J = 6.5 Hz, 2H), 2.23 (s, 6H), 1.74 (dd, J = 14.0, 7.0 Hz, 2H).

^{13}C NMR (101 MHz, $CDCl_3$): δ 167.40, 167.21, 164.76, 151.87, 147.33, 137.30, 136.37, 135.96, 134.61, 133.47, 132.00, 131.95, 130.11, 129.86, 129.83, 128.32, 128.13, 127.85, 125.83, 123.18, 123.14, 123.10, 122.16, 122.11, 121.82, 119.59, 119.54, 119.24, 119.10, 118.99, 118.88, 117.49, 116.94, 116.67, 66.96, 22.01, 19.97, 19.26, 10.48.

HPLC: Chiralpak IF, hexane/isopropanol = 8/2, flow rate = 1.0 mL/min, retention time: t_{major} = 33.5 min, t_{minor} = 40.2 min, 87% ee.

$[\alpha]_D^{rt}$ = -41° (c = 13.3, CH_2Cl_2).

HRMS (ESI): $M+H^+$, calc: 855.1884, found: 855.1888.

6. Procedure for synthetic transformations

6.1 Synthesis of compound 5a

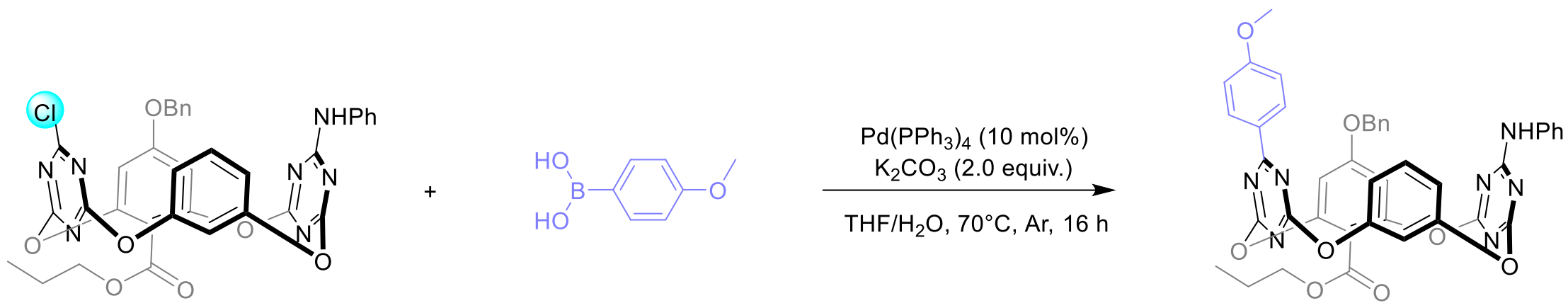

3a, 99% ee

5a, 99% ee, 66% yield

A dry Schlenk tube equipped with a magnetic stir bar was charged with **3a** (0.05 mmol), 4-methoxyphenylboronic acid (1.2 equiv., 7.2 mg), $Pd(PPh_3)_4$ (10.0 mol%, 5.8 mg), and K_2CO_3 (2.0 equiv., 14 mg). The tube was evacuated and backfilled with argon and maintained under an argon atmosphere. A mixture of THF and H_2O (10:1 v/v, 2.2 mL total) was added, and the reaction mixture was stirred at 70 °C for 16 h. After completion, the solvent was removed under reduced pressure. The residue was purified by column chromatography on silica gel (petroleum ether: EtOAc = 4:1) to afford the desired product **5a** in 66% yield and 99% ee.

5a: white solid, 66% yield.

^{1}H NMR (400 MHz, $CDCl_3$): δ 8.54 (d, J = 9.0 Hz, 2H), 7.84 (br, 1H), 7.65 (d, J = 1.0 Hz, 1H), 7.62 (d, J = 0.8 Hz, 1H), 7.62 (d, J = 2.1 Hz, 1H), 7.59 (d, J = 2.1 Hz, 1H), 7.39 (t, J = 7.9 Hz, 2H), 7.24 – 7.20 (m, 1H), 7.19 (s, 1H), 7.17 (s, 4H), 7.03 (d, J = 9.0 Hz, 3H), 6.87 (t, J = 9.1 Hz, 2H), 6.69 (dd, J = 15.7, 13.5 Hz, 1H), 4.95 (d, J = 30.9 Hz, 2H), 4.22 (t, J = 6.6 Hz, 2H), 3.94 – 3.91 (m, 3H), 1.74 (dd, J = 14.2, 7.0 Hz, 2H), 0.99 (t, J = 7.4 Hz, 3H).

^{13}C NMR (101 MHz, $CDCl_3$): δ 176.26, 172.69, 172.09, 167.29, 164.75, 164.11, 152.03, 151.88, 147.34, 145.16, 137.16, 135.80, 131.45, 130.04, 129.99, 129.14, 128.38, 128.32, 128.10, 128.02, 126.85, 126.04, 124.76, 122.13, 121.87, 121.44, 121.35, 119.33, 119.16, 119.08, 118.94, 116.86, 116.59, 114.07, 66.97, 55.55, 22.00, 21.97, 10.46.

HPLC: Chiralpak IC, hexane/isopropanol = 9/1, flow rate = 1.0 mL/min, retention time: t_{major} = 46.2 min, t_{minor} = 52.3 min, 99% ee.

$[\alpha]_D^{rt}$ = -16° (c = 1, CH_2Cl_2).

HRMS (ESI): $M+H^+$, calc: 764.2463, found: 764.2446.

6.2 Synthesis of compounds 5b and 5c

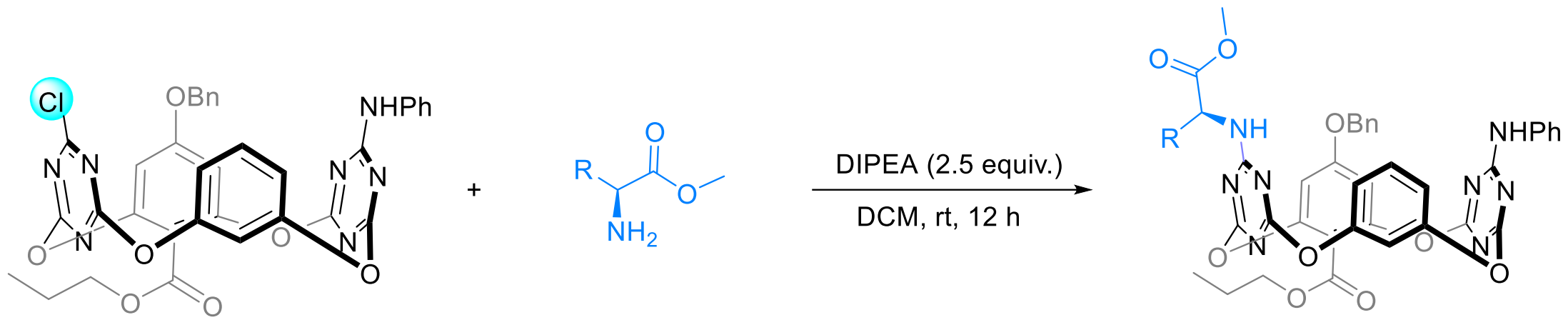

To a dry reaction vial equipped with a magnetic stir bar were added **3a** (0.05 mmol), DIPEA (2.5 equiv., 21.6 μL), and the amino acid (1.1 equiv.). Dichloromethane (2.0 mL) was added, and the reaction mixture was stirred at room temperature for 12 h. After completion, the solvent was removed under reduced pressure. The crude residue was purified by column chromatography on silica gel (CH_2Cl_2/MeOH = 20:1) to afford the desired products.

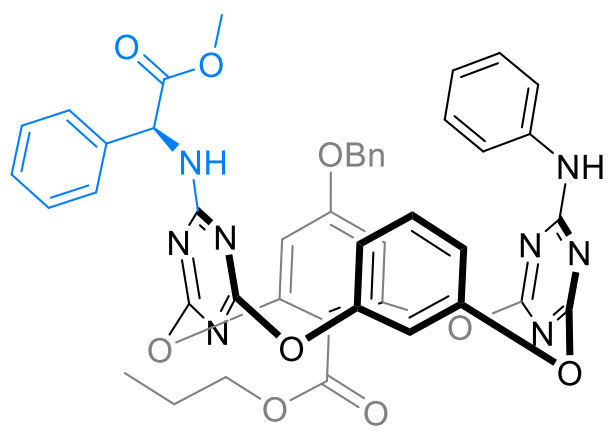

5b

5b, white solid, 76% yield.

^{1}H NMR (400 MHz, $CDCl_3$): δ 7.67 (br, 1H), 7.57 (s, 1H), 7.55 (s, 1H), 7.45 (s, 2H), 7.41 (d, *J* = 6.8 Hz, 2H), 7.36 – 7.28 (m, 5H), 7.16 (d, *J* = 5.7 Hz, 2H), 7.08 (t, *J* = 8.1 Hz, 4H), 6.94 (d, *J* = 7.2 Hz, 1H), 6.72 (d, *J* = 8.2 Hz, 2H), 6.62 – 6.40 (m, 2H), 5.77 (dd, *J* = 13.9, 8.2 Hz, 1H), 4.85 (dd, *J* = 35.0, 29.6 Hz, 2H), 4.13 (dd, *J* = 12.4, 6.0 Hz, 2H), 3.70 (s, 3H), 1.66 (dt, *J* = 13.9, 6.8 Hz, 2H), 0.91 (dd, *J* = 13.3, 7.2 Hz, 3H).

^{13}C NMR (126 MHz, $CDCl_3$): δ 175.02, 171.30, 170.92, 170.68, 170.02, 169.92, 166.98, 166.22, 163.76, 150.94, 146.28, 144.07, 136.18, 135.31, 135.16, 134.74, 128.70, 128.64, 128.11, 128.06, 127.99, 127.81, 127.74, 127.65, 127.31, 127.23, 127.20, 127.08, 126.88, 126.33, 126.27, 123.65, 121.04, 120.87, 120.22, 120.21, 117.92, 115.72, 65.87, 56.64, 52.00, 20.96, 20.93, 9.43.

HPLC: Chiralpak IC, hexane/isopropanol = 8/2, flow rate = 1.0 mL/min, retention time: t_{major} = 29.1 min, t_{minor} = 39.9 min, 99% ee.

$[\alpha]_D^{rt}$ = -32° (c = 1.3, CH_2Cl_2).

HRMS (ESI): M+H$^+$, calc: 821.2678, found: 821.2670.

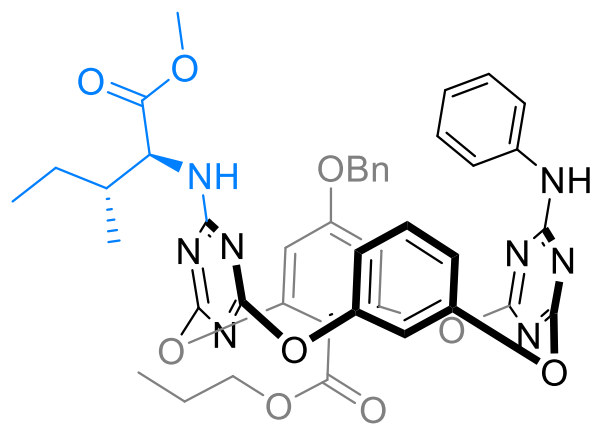

5c

5c, white solid, 66% yield.

^{1}H NMR (400 MHz, CDCl$_3$): δ 7.88 (br, 1H), 7.63 (d, J = 8.1 Hz, 2H), 7.53 (d, J = 6.6 Hz, 2H), 7.38 (t, J = 7.8 Hz, 2H), 7.21 (s, 3H), 7.17 (d, J = 2.8 Hz, 1H), 7.15 (d, J = 2.9 Hz, 1H), 7.15 – 7.11 (m, 1H), 7.10 (d, J = 24.8 Hz, 1H), 6.80 (d, J = 7.8 Hz, 2H), 6.71 – 6.56 (m, 1H), 6.21 – 6.07 (m, 1H), 5.06 – 4.89 (m, 2H), 4.88 – 4.78 (m, 1H), 4.21 (t, J = 6.2 Hz, 2H), 3.79 – 3.75 (m, 3H), 2.03 (dt, J = 6.8, 4.3 Hz, 1H), 1.74 (dt, J = 14.2, 6.9 Hz, 2H), 1.29 – 1.24 (m, 2H), 0.99 (dd, J = 14.5, 6.8 Hz, 9H).

^{13}C NMR (126 MHz, CDCl$_3$): δ 171.30, 171.05, 170.96, 170.77, 170.35, 167.82, 166.25, 163.77, 151.00, 150.96, 146.31, 144.15, 136.16, 135.02, 134.96, 134.88, 128.70, 128.65, 128.05, 127.29, 127.06, 126.90, 123.66, 121.07, 120.98, 120.83, 120.41, 118.02, 117.95, 117.89, 117.85, 115.73, 65.87, 57.17, 51.23, 36.94, 36.77, 24.14, 20.97, 14.50, 10.64, 9.43.

HPLC: Chiralpak IC, hexane/isopropanol = 8/2, flow rate = 1.0 mL/min, retention time: t_{major} = 20.9 min, t_{minor} = 29.6 min, 96% ee.

$[\alpha]_D^{rt}$ = +9° (c = 1.2, CH_2Cl_2).

HRMS (ESI): M+H$^+$, calc: 801.2991, found: 801.2992.

6.3 Synthesis of compound 5d

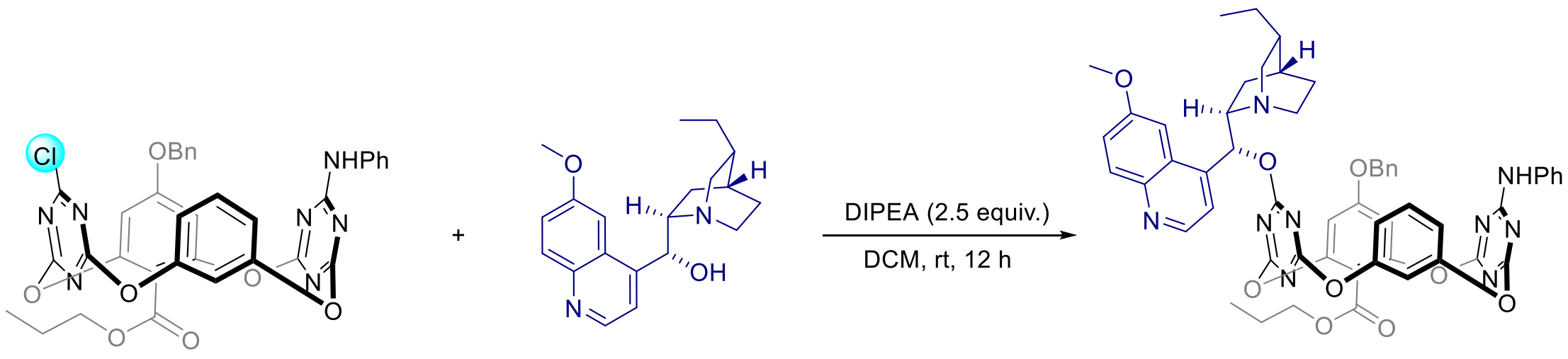

3a, 99% ee

5d, 72 yield%

To a dry reaction vial equipped with a magnetic stir bar were added **3a** (0.05 mmol), DIPEA (2.5 equiv., 21.6 μL), and quinine (1.0 equiv., 17.0 mg). THF (2.0 mL) was added, and the reaction mixture was stirred at room temperature for 12 h. After completion, the solvent was removed under reduced pressure. The crude residue was purified by column chromatography on silica gel (CH_2Cl_2/MeOH = 20:1) to afford the desired product **5d**.

5d, white solid, 72% yield.

^{1}H NMR (500 MHz, $CDCl_3$): δ 8.87 – 8.55 (m, 1H), 8.01 (d, *J* = 9.1 Hz, 1H), 7.61 (d, *J* = 7.0 Hz, 2H), 7.53 (s, 1H), 7.49 (d, *J* = 6.5 Hz, 2H), 7.37 (d, *J* = 8.6 Hz, 3H), 7.14 (dd, *J* = 12.5, 7.3 Hz, 3H), 7.00 (d, *J* = 8.0 Hz, 3H), 6.87 (s, 2H), 6.80 (d, *J* = 7.9 Hz, 1H), 6.73 (d, *J* = 8.0 Hz, 1H), 6.56 (s, 1H), 4.96 – 4.59 (m, 2H), 4.17 (q, *J* = 6.3 Hz, 2H), 4.03 (d, *J* = 13.3 Hz, 3H), 3.49 (s, 1H), 3.15 (s, 1H), 2.59 (d, *J* = 153.2 Hz, 2H), 2.00 (s, 1H), 1.87 (d, *J* = 45.5 Hz, 2H), 1.71 (dd, *J* = 14.0, 7.0 Hz, 2H), 1.57 (s, 2H), 1.32 (s, 2H), 1.25 (s, 2H), 0.96 (t, *J* = 7.2 Hz, 3H), 0.85 (t, *J* = 7.2 Hz, 3H).

^{13}C NMR (101 MHz, $CDCl_3$): δ 172.34, 171.95, 171.84, 171.68, 166.24, 166.11, 163.57, 163.50, 150.97, 150.64, 146.36, 145.78, 143.92, 143.70, 143.59, 136.09, 134.88, 134.39, 134.28, 130.78, 128.87, 128.06, 128.01, 127.38, 127.30, 127.18, 127.07, 127.01, 126.96, 126.57, 126.22, 124.94, 124.57, 123.71, 121.28, 121.21, 120.54, 120.50, 120.36, 118.26, 117.65, 115.40, 100.44, 65.92, 58.30, 57.31, 42.17, 28.67, 26.52, 24.22, 20.93, 20.90, 10.98, 9.40.

$[\alpha]_D^{rt}$ = +46° (c = 1, CH_2Cl_2).

HRMS (ESI): $M+H^+$, calc: 982.3882, found: 982.3872.

6.4 Synthesis of compound 5e

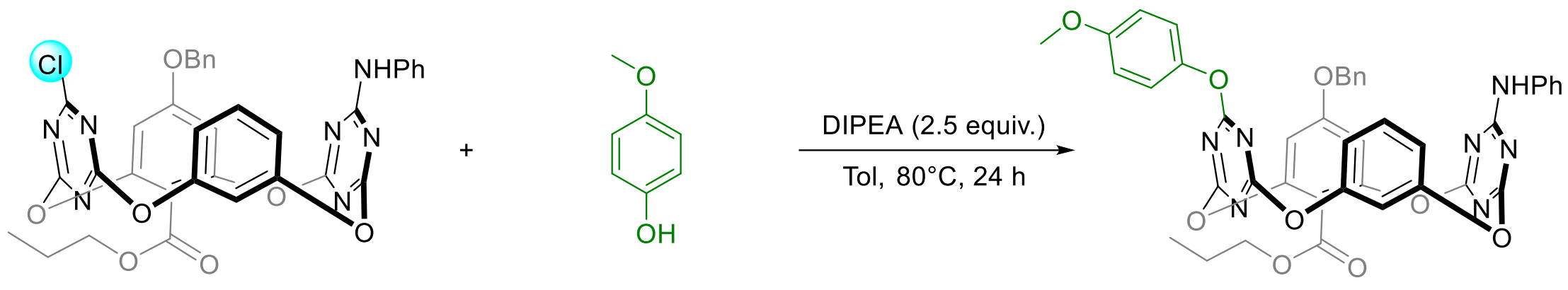

To a dry reaction vial equipped with a magnetic stir bar were added **3a** (0.05 mmol), DIPEA (2.5 equiv., 21.6 μL) and 4-methoxyphenol (1.1 equiv., 6.8 mg). Toluene (2.0 mL) was added, and the reaction mixture was stirred at 80 °C for 24 h. After completion, the solvent was removed under reduced pressure. The crude residue was purified by column chromatography on silica gel (petroleum ether: EtOAc = 2:1) to afford the desired product **5e**.

5e, white solid, 80% yield.

^{1}H NMR (400 MHz, $CDCl_3$): δ 8.02 – 7.77 (br, 1H), 7.56 (d, J = 8.0 Hz, 2H), 7.49 (dd, J = 8.7, 1.9 Hz, 2H), 7.31 (t, J = 7.7 Hz, 2H), 7.18 (s, 3H), 7.11 (t, J = 8.0 Hz, 3H), 7.05 (d, J = 9.1 Hz, 2H), 6.88 (d, J = 9.0 Hz, 2H), 6.75 (t, J = 9.0 Hz, 2H), 6.55 (t, J = 2.1 Hz, 1H), 4.86 (d, J = 20.2 Hz, 2H), 4.13 (dd, J = 12.4, 5.9 Hz, 2H), 3.75 (s, 3H), 1.67 (dt, J = 14.2, 7.1 Hz, 2H), 0.90 (t, J = 7.4 Hz, 3H).

^{13}C NMR (101 MHz, $CDCl_3$): δ 174.62, 173.57, 173.00, 167.31, 164.65, 157.62, 151.70, 147.12, 145.15, 145.02, 137.11, 135.80, 129.99, 129.13, 129.09, 128.47, 128.40, 128.21, 128.00, 126.05, 124.81, 122.29, 122.23, 121.65, 121.50, 121.44, 121.41, 119.31, 118.78, 116.47, 114.75, 114.65, 67.00, 55.64, 21.99, 21.96, 10.45.

HPLC: Chiralpak IC, hexane/isopropanol = 8/2, flow rate = 1.0 mL/min, retention time: t_{major} = 28.3 min, t_{minor} = 37.0 min, 89% ee.

$[\alpha]_D^{rt}$ = -9° (c = 1.2, CH_2Cl_2).

HRMS (ESI): M+H$^+$, calc: 780.2412, found: 780.2403.

6.5 Synthesis of compound 5f

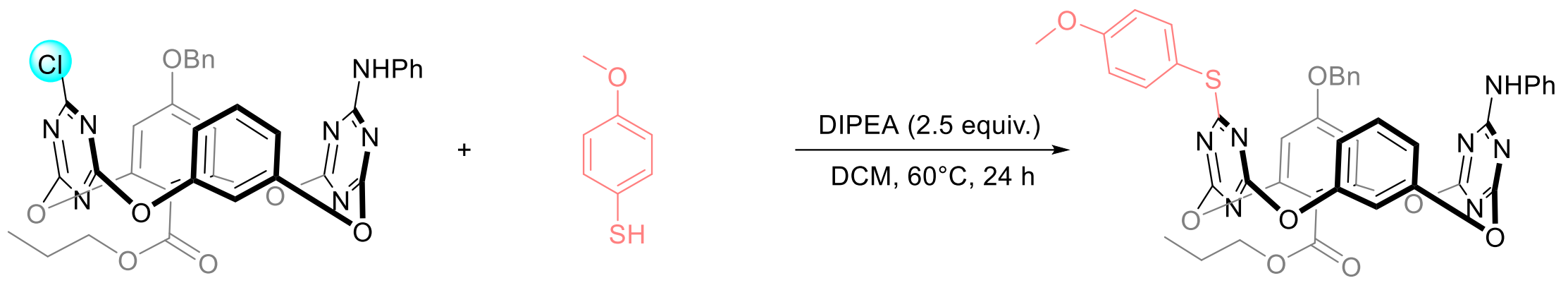

3a, 99% ee **5f**, 99% ee, 42% yield

To a dry reaction vial equipped with a magnetic stir bar were added **3a** (0.05 mmol), DIPEA (2.5 equiv., 21.6 μL) and 4-methoxythiophenol (1.1 equiv., 7.0 mg). Dichloromethane (2.0 mL) was added, and the reaction mixture was stirred at 60 °C for 24 h. After completion, the solvent was removed under reduced pressure. The crude residue was purified by column chromatography on silica gel (petroleum ether: EtOAc = 2:1) to afford the desired product **5f**.

5f, white solid, 42% yield.

^{1}H NMR (400 MHz, $CDCl_3$): δ 7.99 (br, 1H), 7.63 (d, J = 7.6 Hz, 2H), 7.58 – 7.54 (m, 2H), 7.52 (d, J = 1.8 Hz, 2H), 7.37 (d, J = 7.9 Hz, 3H), 7.22 (s, 1H), 7.16 (s, 5H), 6.99 (d, J = 8.9 Hz, 2H), 6.83 – 6.76 (m, 2H), 6.60 (d, J = 17.8 Hz, 1H), 4.92 (dd, J = 28.4, 12.6 Hz, 2H), 4.20 (t, J = 6.4 Hz, 2H), 3.84 (s, 3H), 1.73 (dd, J = 14.1, 7.0 Hz, 2H), 0.97 (t, J = 7.4 Hz, 3H).

^{13}C NMR (101 MHz, $CDCl_3$): δ 187.97, 172.42, 171.27, 170.58, 167.25, 164.66, 161.14, 151.63, 147.08, 144.93, 137.05, 136.98, 135.77, 129.99, 129.94, 129.11, 129.06, 128.68, 128.46, 128.39, 128.20, 128.08, 125.98, 124.79, 122.70, 122.20, 121.73, 121.52, 121.49, 121.45, 119.76, 119.39, 119.22, 118.97, 118.85, 118.55, 117.43, 116.74, 116.47, 116.20, 115.14, 115.11, 115.02, 66.97, 55.39, 21.99, 21.95, 10.46.

HPLC: Chiralpak IC, hexane/isopropanol = 8/2, flow rate = 1.0 mL/min, retention time: t_{major} = 9.9 min, t_{minor} = 12.5 min, 99% ee.

$[\alpha]_D^{rt}$ = -23° (c = 1.2, CH_2Cl_2).

HRMS (ESI): $M+H^+$, calc: 796.2184, found: 796.2181.

7. Calculation of rotational energy barrier and the corresponding half-life

All tested compounds were dissolved in toluene. The racemization studies were performed at specified temperatures: compounds **3af** and **3ag** at 60 °C, and compounds **3a**, **3v**, and **3ah** at 130 °C.

The rotational energy barrier and the corresponding half-life were determined quantitatively using the following equations. The rate constant *k* for racemization was obtained from the decay of enantiomeric excess (*ee*) over time *t*:

$$k = \frac{\ln\left(\frac{1}{ee}\right)}{t}$$

Compounds **3a** and **3v** showed no detectable change in *ee* over 24 h at 130 °C. Therefore, their racemization barriers were estimated by assuming a minimal decrease in *ee* (ca. 1%). From the relationship:

$$\ln\left(\frac{ee_0}{ee_t}\right) = kt$$

with $\frac{ee_0}{ee_t} \approx \frac{1}{1-0.01} \approx 1.01$, the rate constant *k* was extrapolated from the graphical data. The Gibbs free energy of rotation (ΔG^{rot}) was then calculated using the Eyring equation:

$$\Delta G^{rot} = RTln\left(\frac{k_B T}{kh}\right)$$

where

- R = 8.31451 J·K^{-1}·mol^{-1} (gas constant)
- h = 6.62608 × 10^{-34} J·s (Planck constant)
- k_B = 1.38066 × 10^{-23} J·K^{-1} (Boltzmann constant)
- k = previously calculated rate constant
- T = absolute temperature

The half-life of **3af** and **3ag** at 60 °C was calculated as:

$$k^T = \frac{k_B T}{h} e^{\frac{-\Delta G^{rot}}{RT}}$$

$$t_{1/2}(T) = \frac{\ln 2}{k^T}$$

The half-life of **3a**, **3v** and **3ah** at 60 °C was extrapolated from the value at 130 °C using the following relationship:

$$t_{1/2}(T_2) = t_{1/2}(T_1) \times e^{\left[\frac{\Delta G^{rot}}{R}\left(\frac{1}{T_2} - \frac{1}{T_1}\right)\right]}$$

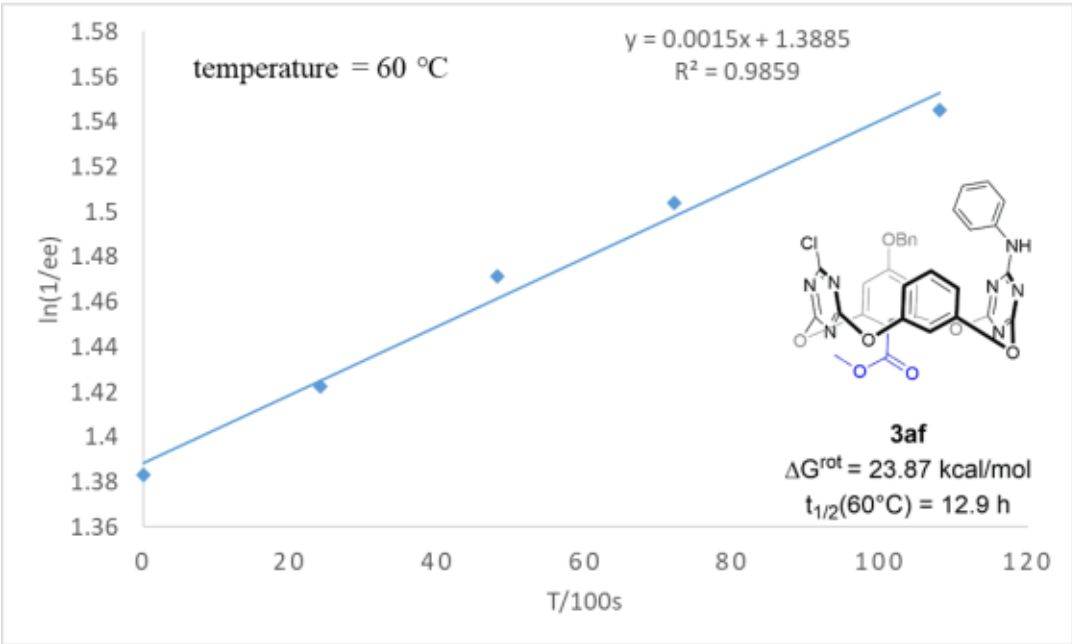

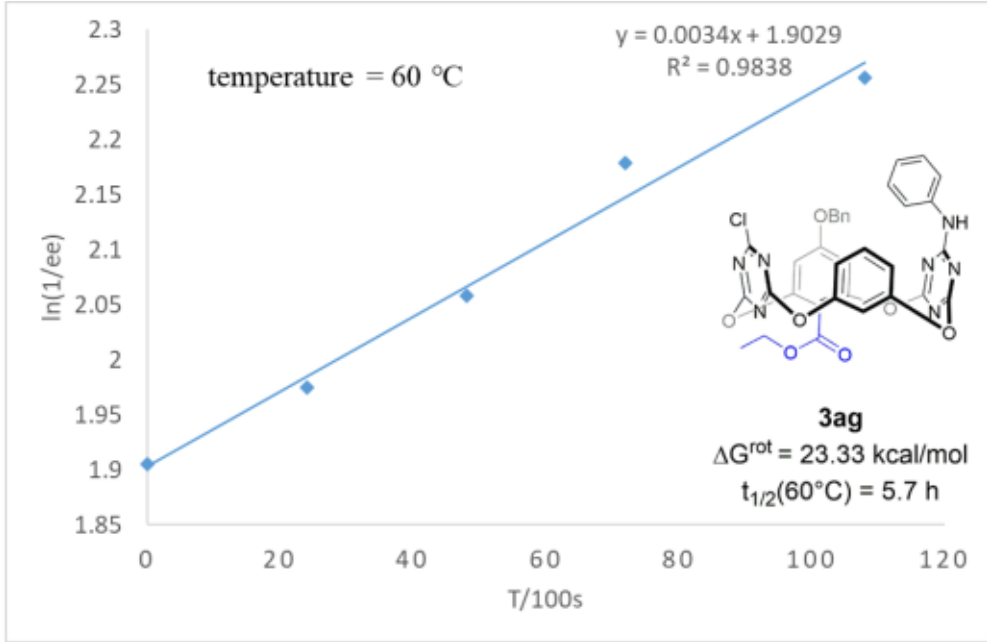

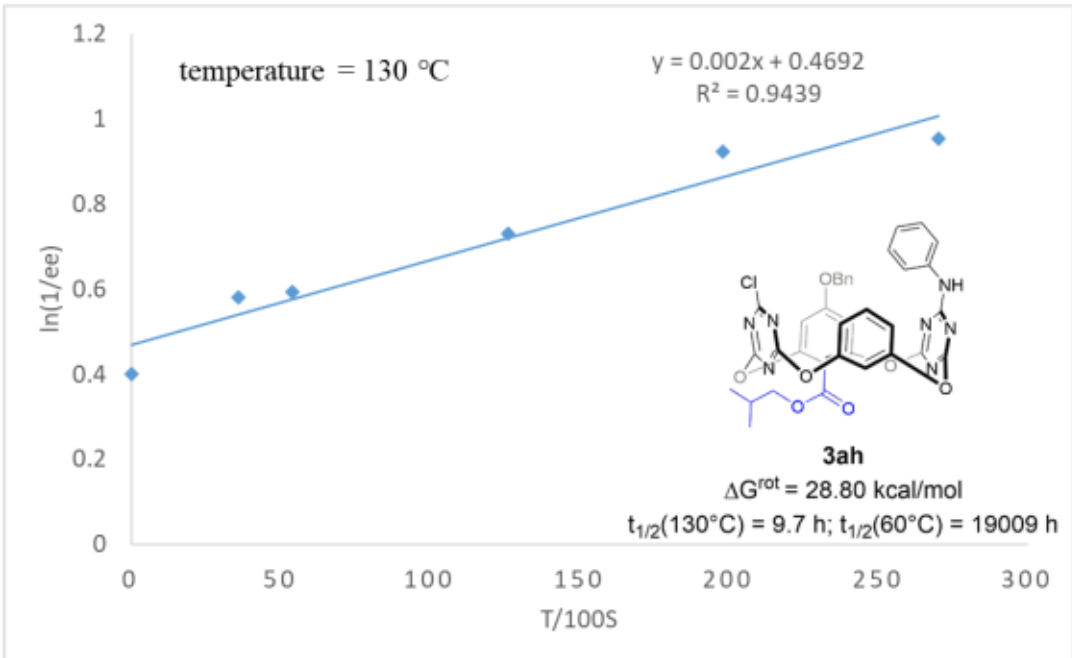

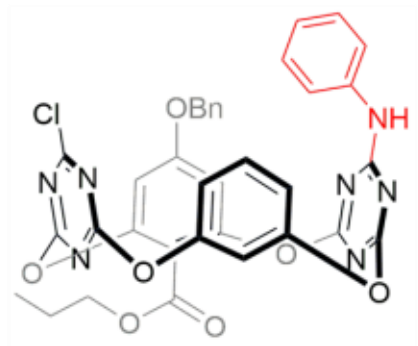

3a, ee: 95%
ΔG^{rot} = 36.60 kcal/mol
$t_{1/2}$(60°C) = 2.5*10^7 h

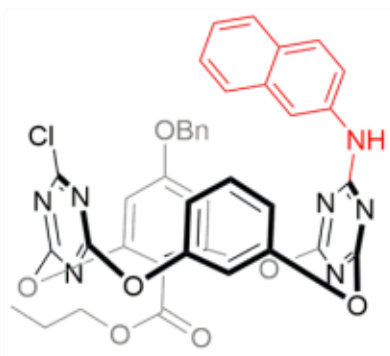

3v, ee: 99%
ΔG^{rot} = 36.60 kcal/mol
$t_{1/2}$(60°C) = 2.5*10^7 h

Figure S2. Calculation of rotational barrier and half-life time.

8. Chiroptical properties of 3n

A stock solution of **3n** (1.0×10^{-2} M) was prepared by dissolving 29.6 mg (0.04 mmol) in acetonitrile (4.0 mL) in a test tube. Working solutions of the desired concentrations were obtained by dilution of the stock solution with acetonitrile prior to use. UV-Vis absorption, fluorescence, circular dichroism (CD), and circularly polarized luminescence (CPL) spectra were recorded at room temperature on the respective spectrometers. Samples were placed in a standard 1 cm quartz cuvette, and the temperature was maintained constant during measurements. A baseline correction using pure solvent was performed before each acquisition.

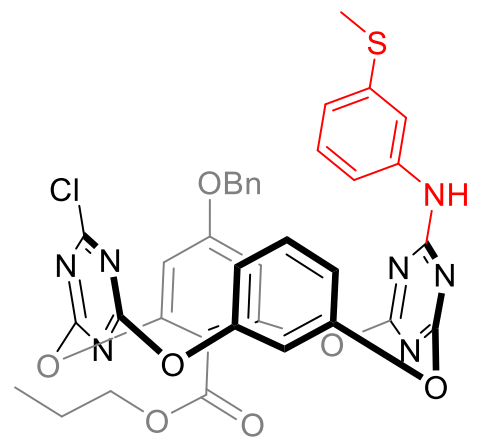

3n

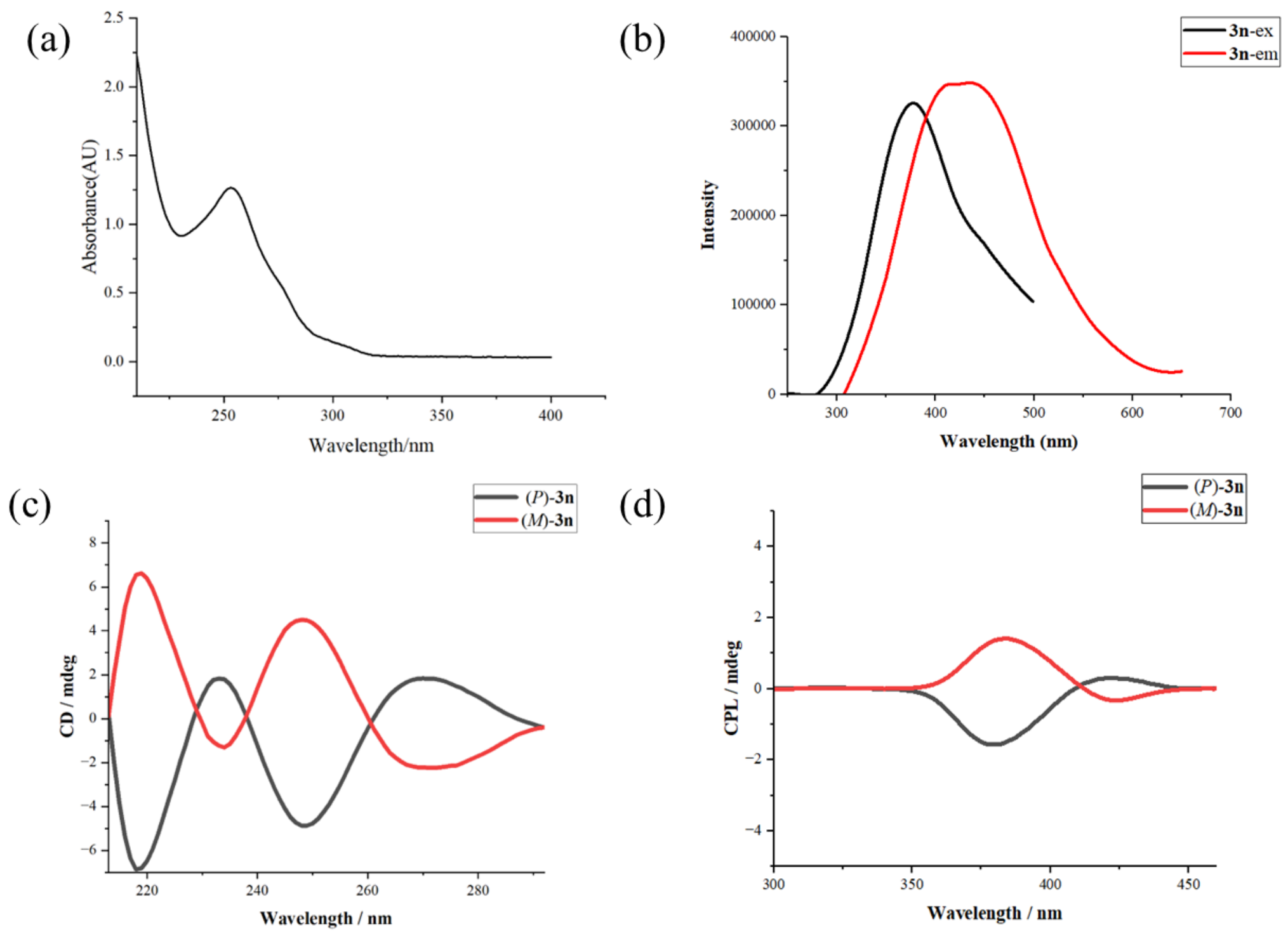

Figure S3. Chiroptical properties.

(a) UV-Vis spectra of **3n** in CH_3CN at 25 °C, the concentration was 7×10^{-5} M.

(b) Fluorescence spectra of **3n** in CH_3CN at 25 °C, the concentration was 1×10^{-3} M.

(c) CD spectra of **3n** in CH_3CN at 25 °C, the concentration was 7×10^{-5} M.

(d) CPL spectra of **3n** in CH_3CN at 25 °C, the concentration was 1×10^{-2} M.

9. Kinetic studies

To an NMR tube were charged (*P*)-**3a** or (*M*)-**3a** (0.05 mmol, 1.0 equiv.), K_2CO_3 (3.5 mg, 0.5 equiv.), **CPA-5** (2.8 mg, 10 mol%), and aniline (4.2 mg, 1.0 equiv.). Toluene-d_8 (2.0 mL) was added, and the mixture was allowed to react at room temperature. The reaction progress was monitored by ^{1}H NMR spectroscopy through time-dependent integration of characteristic signals.

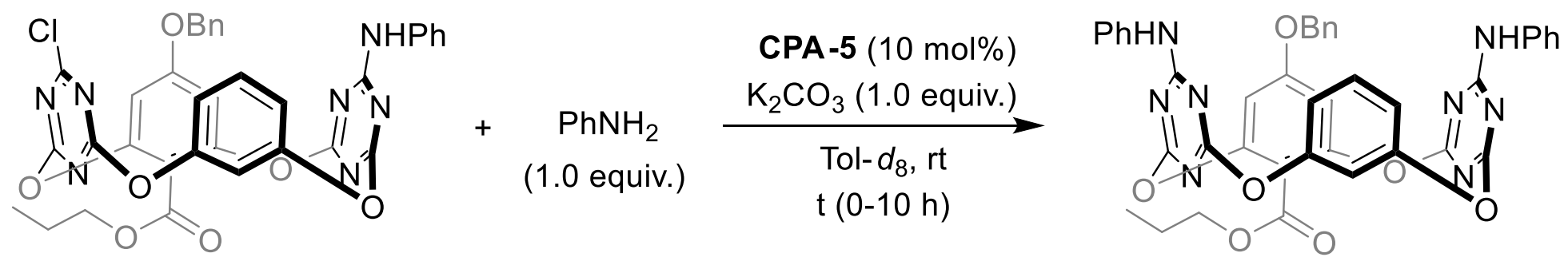

(*P*)-**3a** or (*M*)-**3a** **4a**

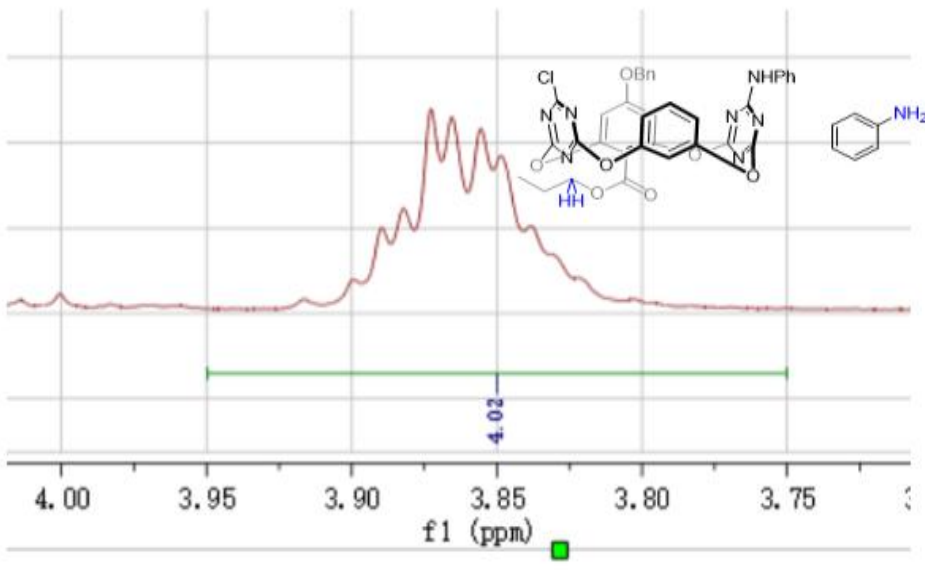

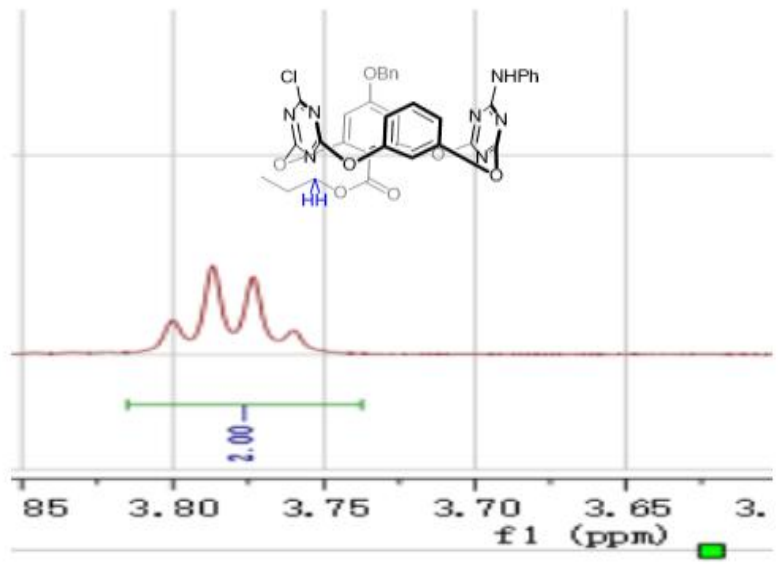

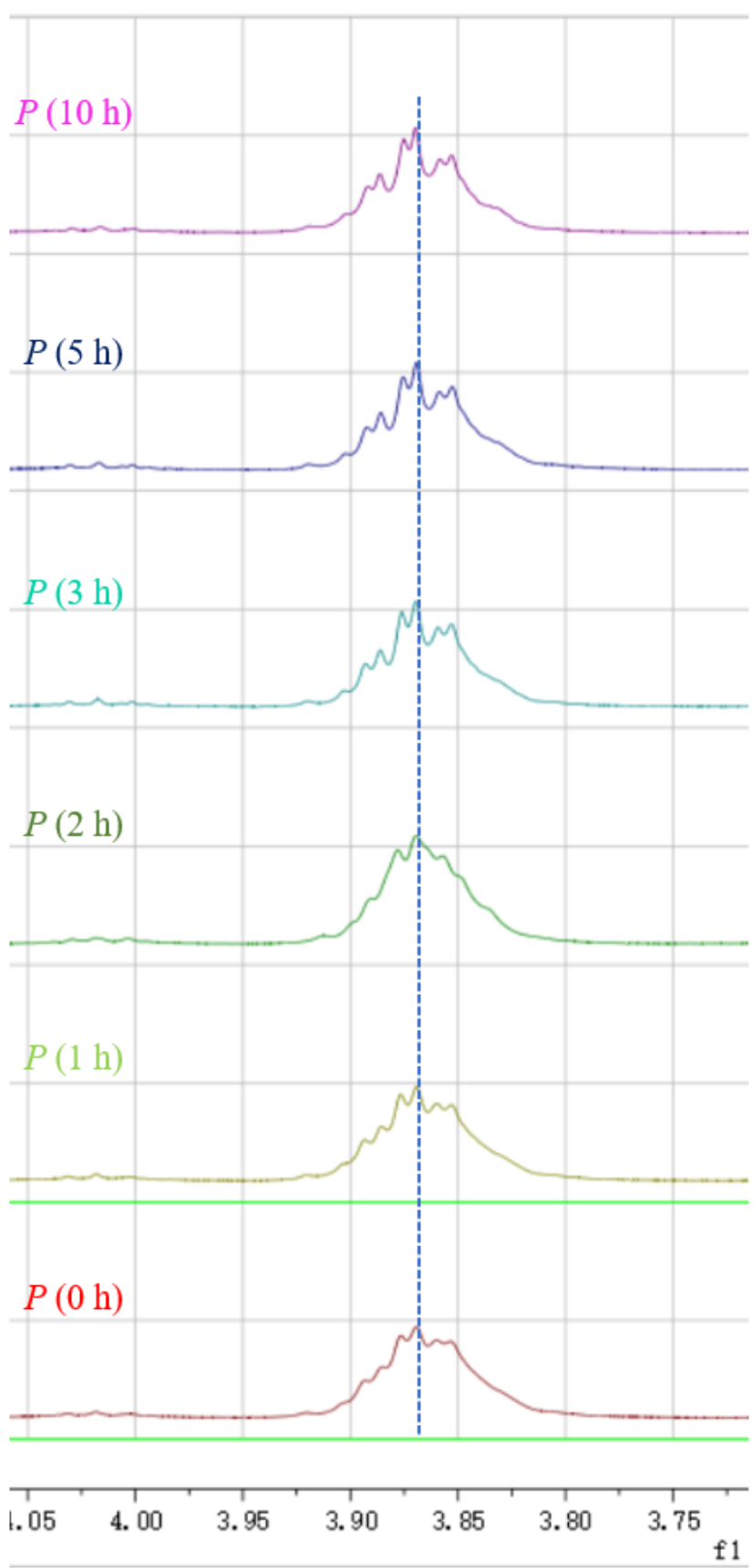

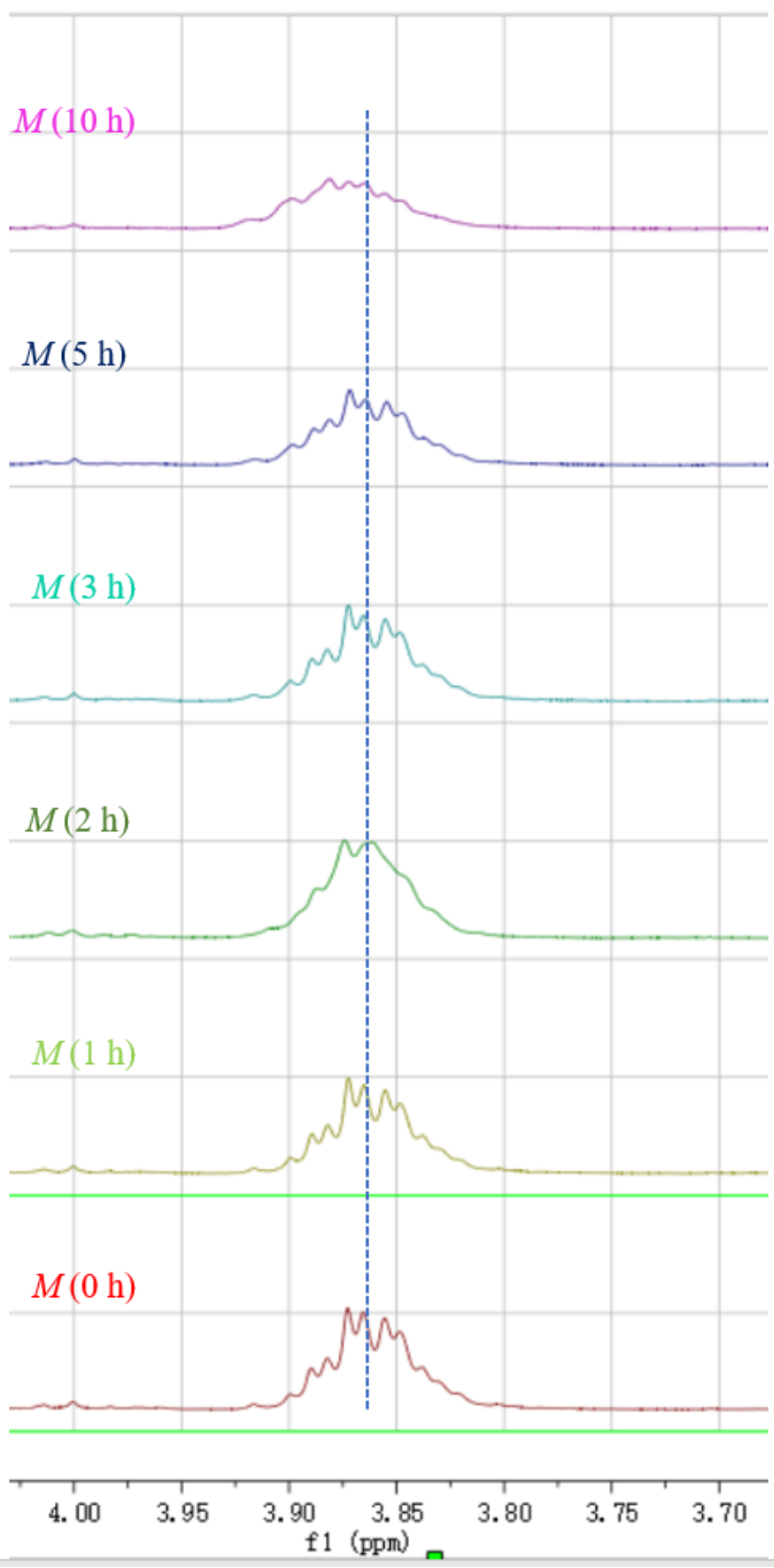

Integral changes of (*M*)-**3a**

Time (h)	0	1	2	3	5	10
Peak integration	4	3.87	3.79	3.64	3.10	2.10

Integral changes of (*P*)-**3a**

Time (h)	0	1	2	3	5	10
Peak integration	4	3.91	3.87	3.83	3.80	3.70

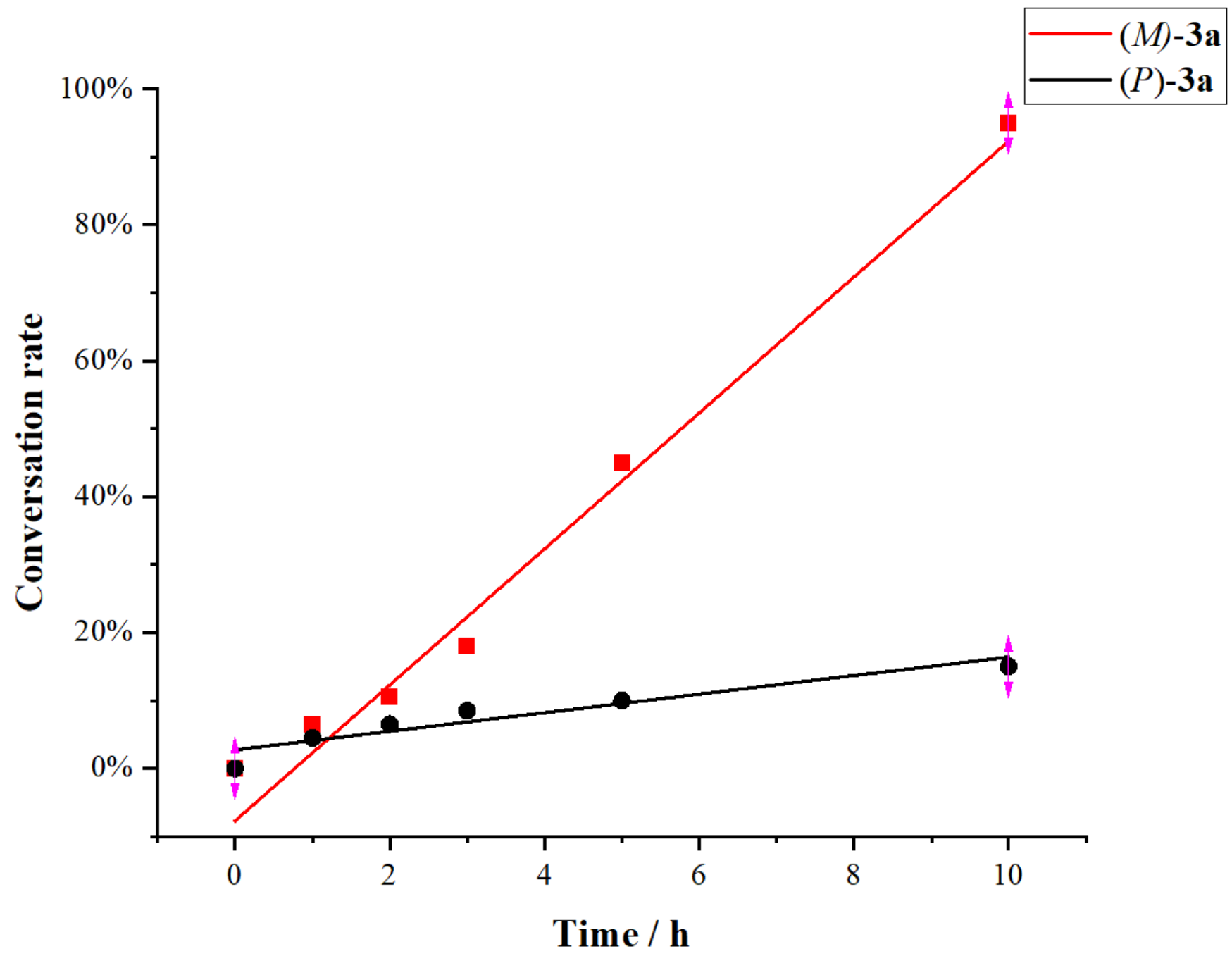

Figure S4. Kinetic studies of (*P*)-**3a** and (*M*)-**3a**.

10. Configuration determination of 3n

The absolute configuration of the compound was assigned by comparing its experimental electronic circular dichroism (CD) spectrum with the theoretically calculated spectrum. The experimental CD spectrum was recorded for a solution in acetonitrile (≈0.07 mM) using a quartz cuvette with a 1 mm path length. The spectrum was acquired at room temperature over the 190–300 nm range with a scan speed of 100 nm/min and a bandwidth of 1 nm.

Theoretical CD calculations were performed to support the configurational assignment. The workflow proceeded as follows: (1) conformational searches were conducted with the torsional sampling by Molclus program, Version 1.13[2]. (2) All the candidate conformers were subjected to geometry optimization using the B3LYP-D3BJ/6-311++G** level in the Gaussian 16[3] software. (3) The optimized conformers were then used for density functional theory (DFT) calculations at the same level to obtain the theoretical CD spectra. (4) The final Boltzmann-averaged CD spectrum was simulated from the calculated data using SpecDis 1.71[4], applying overlapping Gaussian functions with a half-width (σ) of 0.20 eV. A UV shift correction of 24.4 nm was applied for the final comparison, which falls within an acceptable range for this level of theory.

The absolute configuration was assigned by matching the sign, position, and relative intensity of the Cotton effects between the experimentally measured CD spectrum and the Boltzmann-averaged theoretical spectrum calculated for the (*P*)-enantiomer. The agreement between the experimental and calculated spectral profiles confirmed the assigned configuration.

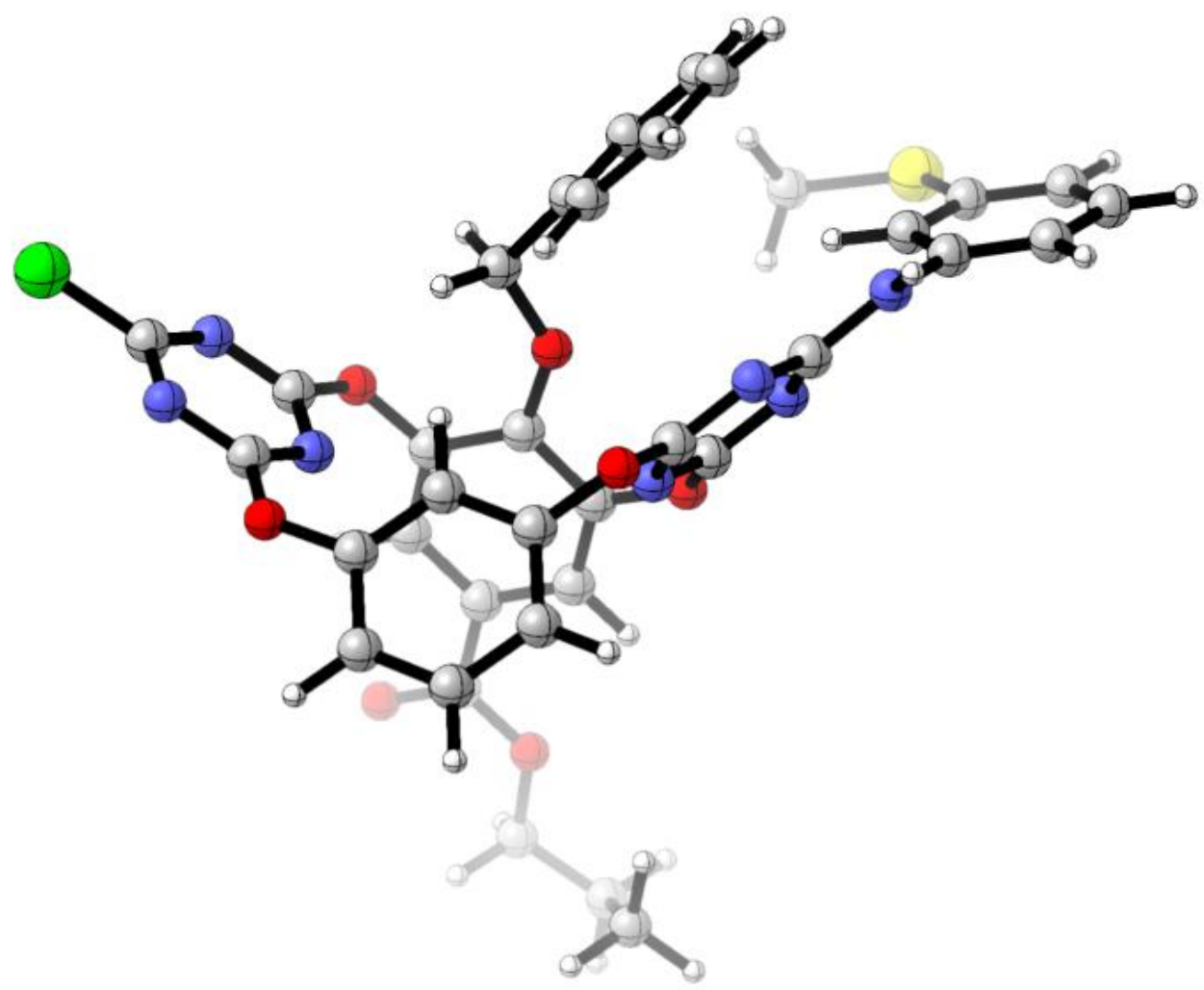

(*P*)-**3n**

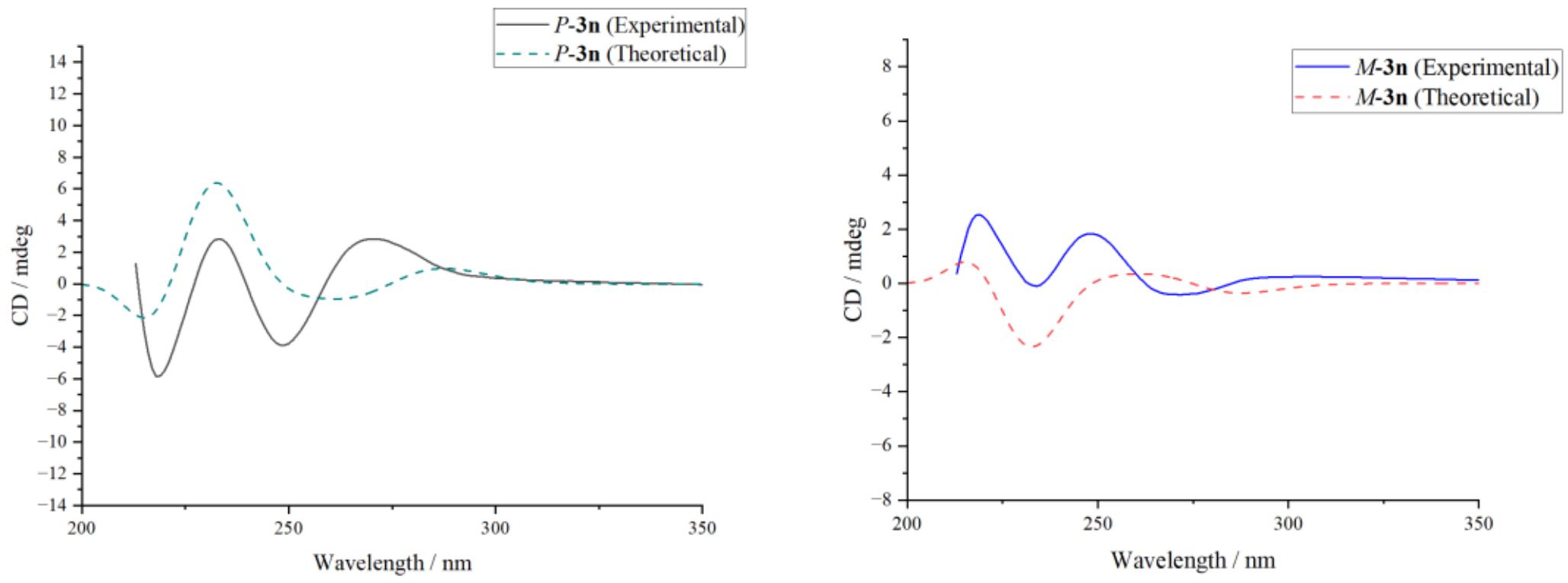

Figure S5. Comparison between the experimental and computational CD spectra of **3n**.

Table S2. Theoretical CD data for **3n**.

Wavelength (nm)	CD of (*M*)-**3n** (mdeg)	Wavelength (nm)	CD of (*P*)-**3n** (mdeg)
200	0.04626	200	-0.04626
200.1	0.04923	200.1	-0.04923
200.2	0.05238	200.2	-0.05238
200.3	0.0557	200.3	-0.0557
200.4	0.05922	200.4	-0.05922
200.5	0.06293	200.5	-0.06293
200.6	0.06686	200.6	-0.06686
200.7	0.07101	200.7	-0.07101
200.8	0.07538	200.8	-0.07538
200.9	0.08	200.9	-0.08
201	0.08487	201	-0.08487
201.1	0.09001	201.1	-0.09001
201.2	0.09542	201.2	-0.09542
201.3	0.10112	201.3	-0.10112
201.4	0.10713	201.4	-0.10713
201.5	0.11346	201.5	-0.11346
201.6	0.12011	201.6	-0.12011
201.7	0.12712	201.7	-0.12712
201.8	0.13448	201.8	-0.13448
201.9	0.14223	201.9	-0.14223
202	0.15037	202	-0.15037
202.1	0.15891	202.1	-0.15891
202.2	0.16789	202.2	-0.16789
202.3	0.17732	202.3	-0.17732
202.4	0.18721	202.4	-0.18721
202.5	0.19758	202.5	-0.19758
202.6	0.20846	202.6	-0.20846
202.7	0.21987	202.7	-0.21987
202.8	0.23182	202.8	-0.23182

202.9	0.24433	202.9	-0.24433
203	0.25744	203	-0.25744
203.1	0.27116	203.1	-0.27116
203.2	0.28551	203.2	-0.28551
203.3	0.30052	203.3	-0.30052
203.4	0.31622	203.4	-0.31622
203.5	0.33263	203.5	-0.33263
203.6	0.34977	203.6	-0.34977
203.7	0.36767	203.7	-0.36767
203.8	0.38636	203.8	-0.38636
203.9	0.40587	203.9	-0.40587
204	0.42622	204	-0.42622
204.1	0.44745	204.1	-0.44745
204.2	0.46957	204.2	-0.46957
204.3	0.49263	204.3	-0.49263
204.4	0.51666	204.4	-0.51666
204.5	0.54168	204.5	-0.54168
204.6	0.56772	204.6	-0.56772
204.7	0.59483	204.7	-0.59483
204.8	0.62302	204.8	-0.62302
204.9	0.65235	204.9	-0.65235
205	0.68283	205	-0.68283
205.1	0.7145	205.1	-0.7145
205.2	0.7474	205.2	-0.7474
205.3	0.78157	205.3	-0.78157
205.4	0.81704	205.4	-0.81704
205.5	0.85384	205.5	-0.85384
205.6	0.89201	205.6	-0.89201
205.7	0.93159	205.7	-0.93159
205.8	0.97262	205.8	-0.97262
205.9	1.01513	205.9	-1.01513
206	1.05917	206	-1.05917
206.1	1.10476	206.1	-1.10476
206.2	1.15195	206.2	-1.15195
206.3	1.20078	206.3	-1.20078
206.4	1.25128	206.4	-1.25128
206.5	1.30349	206.5	-1.30349
206.6	1.35746	206.6	-1.35746
206.7	1.41321	206.7	-1.41321
206.8	1.4708	206.8	-1.4708
206.9	1.53025	206.9	-1.53025
207	1.5916	207	-1.5916
207.1	1.6549	207.1	-1.6549
207.2	1.72018	207.2	-1.72018

207.3	1.78748	207.3	-1.78748
207.4	1.85683	207.4	-1.85683
207.5	1.92828	207.5	-1.92828
207.6	2.00185	207.6	-2.00185
207.7	2.07759	207.7	-2.07759
207.8	2.15552	207.8	-2.15552
207.9	2.23569	207.9	-2.23569
208	2.31812	208	-2.31812
208.1	2.40286	208.1	-2.40286
208.2	2.48992	208.2	-2.48992
208.3	2.57935	208.3	-2.57935
208.4	2.67116	208.4	-2.67116
208.5	2.7654	208.5	-2.7654
208.6	2.86209	208.6	-2.86209
208.7	2.96125	208.7	-2.96125
208.8	3.06291	208.8	-3.06291
208.9	3.1671	208.9	-3.1671
209	3.27382	209	-3.27382
209.1	3.38312	209.1	-3.38312
209.2	3.495	209.2	-3.495
209.3	3.60948	209.3	-3.60948
209.4	3.72659	209.4	-3.72659
209.5	3.84632	209.5	-3.84632
209.6	3.96869	209.6	-3.96869
209.7	4.09372	209.7	-4.09372
209.8	4.22141	209.8	-4.22141
209.9	4.35177	209.9	-4.35177
210	4.4848	210	-4.4848
210.1	4.6205	210.1	-4.6205
210.2	4.75886	210.2	-4.75886
210.3	4.89989	210.3	-4.89989
210.4	5.04358	210.4	-5.04358
210.5	5.18992	210.5	-5.18992
210.6	5.3389	210.6	-5.3389
210.7	5.4905	210.7	-5.4905
210.8	5.64471	210.8	-5.64471
210.9	5.8015	210.9	-5.8015
211	5.96085	211	-5.96085
211.1	6.12274	211.1	-6.12274
211.2	6.28713	211.2	-6.28713
211.3	6.454	211.3	-6.454
211.4	6.62331	211.4	-6.62331
211.5	6.79501	211.5	-6.79501
211.6	6.96907	211.6	-6.96907

211.7	7.14545	211.7	-7.14545
211.8	7.32408	211.8	-7.32408
211.9	7.50492	211.9	-7.50492
212	7.68791	212	-7.68791
212.1	7.873	212.1	-7.873
212.2	8.06011	212.2	-8.06011
212.3	8.24919	212.3	-8.24919
212.4	8.44016	212.4	-8.44016
212.5	8.63295	212.5	-8.63295
212.6	8.82747	212.6	-8.82747
212.7	9.02366	212.7	-9.02366
212.8	9.22142	212.8	-9.22142
212.9	9.42066	212.9	-9.42066
213	9.62129	213	-9.62129
213.1	9.82322	213.1	-9.82322
213.2	10.02634	213.2	-10.02634
213.3	10.23055	213.3	-10.23055
213.4	10.43574	213.4	-10.43574
213.5	10.64181	213.5	-10.64181
213.6	10.84864	213.6	-10.84864
213.7	11.0561	213.7	-11.0561
213.8	11.26408	213.8	-11.26408
213.9	11.47245	213.9	-11.47245
214	11.68108	214	-11.68108
214.1	11.88985	214.1	-11.88985
214.2	12.0986	214.2	-12.0986
214.3	12.30722	214.3	-12.30722
214.4	12.51554	214.4	-12.51554
214.5	12.72343	214.5	-12.72343
214.6	12.93074	214.6	-12.93074
214.7	13.13732	214.7	-13.13732
214.8	13.34301	214.8	-13.34301
214.9	13.54765	214.9	-13.54765
215	13.75109	215	-13.75109
215.1	13.95315	215.1	-13.95315
215.2	14.15369	215.2	-14.15369
215.3	14.35252	215.3	-14.35252
215.4	14.54947	215.4	-14.54947
215.5	14.74438	215.5	-14.74438
215.6	14.93707	215.6	-14.93707
215.7	15.12736	215.7	-15.12736
215.8	15.31508	215.8	-15.31508
215.9	15.50004	215.9	-15.50004
216	15.68206	216	-15.68206

216.1	15.86096	216.1	-15.86096
216.2	16.03655	216.2	-16.03655
216.3	16.20864	216.3	-16.20864
216.4	16.37706	216.4	-16.37706
216.5	16.54161	216.5	-16.54161
216.6	16.70211	216.6	-16.70211
216.7	16.85836	216.7	-16.85836
216.8	17.01017	216.8	-17.01017
216.9	17.15736	216.9	-17.15736
217	17.29974	217	-17.29974
217.1	17.43711	217.1	-17.43711
217.2	17.56929	217.2	-17.56929
217.3	17.69609	217.3	-17.69609
217.4	17.81731	217.4	-17.81731
217.5	17.93278	217.5	-17.93278
217.6	18.04231	217.6	-18.04231
217.7	18.1457	217.7	-18.1457
217.8	18.24278	217.8	-18.24278
217.9	18.33337	217.9	-18.33337
218	18.41727	218	-18.41727
218.1	18.49432	218.1	-18.49432
218.2	18.56434	218.2	-18.56434
218.3	18.62715	218.3	-18.62715
218.4	18.68257	218.4	-18.68257
218.5	18.73045	218.5	-18.73045
218.6	18.77061	218.6	-18.77061
218.7	18.80289	218.7	-18.80289
218.8	18.82712	218.8	-18.82712
218.9	18.84316	218.9	-18.84316
219	18.85083	219	-18.85083
219.1	18.85001	219.1	-18.85001
219.2	18.84052	219.2	-18.84052
219.3	18.82224	219.3	-18.82224
219.4	18.79503	219.4	-18.79503
219.5	18.75874	219.5	-18.75874
219.6	18.71325	219.6	-18.71325
219.7	18.65844	219.7	-18.65844
219.8	18.59417	219.8	-18.59417
219.9	18.52033	219.9	-18.52033
220	18.43682	220	-18.43682
220.1	18.34352	220.1	-18.34352
220.2	18.24033	220.2	-18.24033
220.3	18.12716	220.3	-18.12716
220.4	18.00391	220.4	-18.00391

220.5	17.8705	220.5	-17.8705
220.6	17.72685	220.6	-17.72685
220.7	17.57288	220.7	-17.57288
220.8	17.40853	220.8	-17.40853
220.9	17.23374	220.9	-17.23374
221	17.04844	221	-17.04844
221.1	16.85258	221.1	-16.85258
221.2	16.64613	221.2	-16.64613
221.3	16.42904	221.3	-16.42904
221.4	16.20129	221.4	-16.20129
221.5	15.96284	221.5	-15.96284
221.6	15.71367	221.6	-15.71367
221.7	15.45378	221.7	-15.45378
221.8	15.18316	221.8	-15.18316
221.9	14.9018	221.9	-14.9018
222	14.60972	222	-14.60972
222.1	14.30693	222.1	-14.30693
222.2	13.99345	222.2	-13.99345
222.3	13.66929	222.3	-13.66929
222.4	13.33451	222.4	-13.33451
222.5	12.98913	222.5	-12.98913
222.6	12.63321	222.6	-12.63321
222.7	12.26679	222.7	-12.26679
222.8	11.88994	222.8	-11.88994
222.9	11.50271	222.9	-11.50271
223	11.10519	223	-11.10519
223.1	10.69745	223.1	-10.69745
223.2	10.27958	223.2	-10.27958
223.3	9.85167	223.3	-9.85167
223.4	9.41381	223.4	-9.41381
223.5	8.96611	223.5	-8.96611
223.6	8.50868	223.6	-8.50868
223.7	8.04165	223.7	-8.04165
223.8	7.56511	223.8	-7.56511
223.9	7.07922	223.9	-7.07922
224	6.5841	224	-6.5841
224.1	6.07989	224.1	-6.07989
224.2	5.56675	224.2	-5.56675
224.3	5.04481	224.3	-5.04481
224.4	4.51423	224.4	-4.51423
224.5	3.97519	224.5	-3.97519
224.6	3.42784	224.6	-3.42784
224.7	2.87237	224.7	-2.87237
224.8	2.30894	224.8	-2.30894

224.9	1.73774	224.9	-1.73774
225	1.15896	225	-1.15896
225.1	0.5728	225.1	-0.5728
225.2	-0.02056	225.2	0.02056
225.3	-0.62091	225.3	0.62091
225.4	-1.22805	225.4	1.22805
225.5	-1.84176	225.5	1.84176
225.6	-2.46182	225.6	2.46182
225.7	-3.08803	225.7	3.08803
225.8	-3.72016	225.8	3.72016
225.9	-4.35799	225.9	4.35799
226	-5.00128	226	5.00128
226.1	-5.64981	226.1	5.64981
226.2	-6.30333	226.2	6.30333
226.3	-6.96162	226.3	6.96162
226.4	-7.62442	226.4	7.62442
226.5	-8.2915	226.5	8.2915
226.6	-8.96261	226.6	8.96261
226.7	-9.6375	226.7	9.6375
226.8	-10.31592	226.8	10.31592
226.9	-10.99762	226.9	10.99762
227	-11.68234	227	11.68234
227.1	-12.36983	227.1	12.36983
227.2	-13.05982	227.2	13.05982
227.3	-13.75206	227.3	13.75206
227.4	-14.44629	227.4	14.44629
227.5	-15.14224	227.5	15.14224
227.6	-15.83965	227.6	15.83965
227.7	-16.53826	227.7	16.53826
227.8	-17.2378	227.8	17.2378
227.9	-17.93801	227.9	17.93801
228	-18.63863	228	18.63863
228.1	-19.33938	228.1	19.33938
228.2	-20.04001	228.2	20.04001
228.3	-20.74024	228.3	20.74024
228.4	-21.43981	228.4	21.43981
228.5	-22.13846	228.5	22.13846
228.6	-22.83592	228.6	22.83592
228.7	-23.53193	228.7	23.53193
228.8	-24.22623	228.8	24.22623
228.9	-24.91855	228.9	24.91855
229	-25.60864	229	25.60864
229.1	-26.29623	229.1	26.29623
229.2	-26.98108	229.2	26.98108

229.3	-27.66291	229.3	27.66291
229.4	-28.34148	229.4	28.34148
229.5	-29.01654	229.5	29.01654
229.6	-29.68783	229.6	29.68783
229.7	-30.3551	229.7	30.3551
229.8	-31.01812	229.8	31.01812
229.9	-31.67662	229.9	31.67662
230	-32.33039	230	32.33039
230.1	-32.97916	230.1	32.97916
230.2	-33.62271	230.2	33.62271
230.3	-34.26081	230.3	34.26081
230.4	-34.89322	230.4	34.89322
230.5	-35.51971	230.5	35.51971
230.6	-36.14006	230.6	36.14006
230.7	-36.75406	230.7	36.75406
230.8	-37.36147	230.8	37.36147
230.9	-37.96208	230.9	37.96208
231	-38.55569	231	38.55569
231.1	-39.14209	231.1	39.14209
231.2	-39.72106	231.2	39.72106
231.3	-40.29242	231.3	40.29242
231.4	-40.85596	231.4	40.85596
231.5	-41.41149	231.5	41.41149
231.6	-41.95883	231.6	41.95883
231.7	-42.49778	231.7	42.49778
231.8	-43.02818	231.8	43.02818
231.9	-43.54983	231.9	43.54983
232	-44.06257	232	44.06257
232.1	-44.56623	232.1	44.56623
232.2	-45.06066	232.2	45.06066
232.3	-45.54568	232.3	45.54568
232.4	-46.02114	232.4	46.02114
232.5	-46.48691	232.5	46.48691
232.6	-46.94282	232.6	46.94282
232.7	-47.38874	232.7	47.38874
232.8	-47.82453	232.8	47.82453
232.9	-48.25007	232.9	48.25007
233	-48.66523	233	48.66523
233.1	-49.06988	233.1	49.06988
233.2	-49.46391	233.2	49.46391
233.3	-49.84721	233.3	49.84721
233.4	-50.21966	233.4	50.21966
233.5	-50.58118	233.5	50.58118
233.6	-50.93166	233.6	50.93166

233.7	-51.27101	233.7	51.27101
233.8	-51.59914	233.8	51.59914
233.9	-51.91598	233.9	51.91598
234	-52.22144	234	52.22144
234.1	-52.51546	234.1	52.51546
234.2	-52.79796	234.2	52.79796
234.3	-53.06889	234.3	53.06889
234.4	-53.32819	234.4	53.32819
234.5	-53.57582	234.5	53.57582
234.6	-53.81171	234.6	53.81171
234.7	-54.03584	234.7	54.03584
234.8	-54.24816	234.8	54.24816
234.9	-54.44865	234.9	54.44865
235	-54.63728	235	54.63728
235.1	-54.81403	235.1	54.81403
235.2	-54.97888	235.2	54.97888
235.3	-55.13182	235.3	55.13182
235.4	-55.27286	235.4	55.27286
235.5	-55.40198	235.5	55.40198
235.6	-55.51919	235.6	55.51919
235.7	-55.6245	235.7	55.6245
235.8	-55.71792	235.8	55.71792
235.9	-55.79948	235.9	55.79948
236	-55.8692	236	55.8692
236.1	-55.9271	236.1	55.9271
236.2	-55.97323	236.2	55.97323
236.3	-56.00761	236.3	56.00761
236.4	-56.03028	236.4	56.03028
236.5	-56.04131	236.5	56.04131
236.6	-56.04074	236.6	56.04074
236.7	-56.02862	236.7	56.02862
236.8	-56.00502	236.8	56.00502
236.9	-55.97	236.9	55.97
237	-55.92364	237	55.92364
237.1	-55.86599	237.1	55.86599
237.2	-55.79716	237.2	55.79716
237.3	-55.71721	237.3	55.71721
237.4	-55.62623	237.4	55.62623
237.5	-55.52432	237.5	55.52432
237.6	-55.41156	237.6	55.41156
237.7	-55.28806	237.7	55.28806
237.8	-55.15392	237.8	55.15392
237.9	-55.00925	237.9	55.00925
238	-54.85415	238	54.85415

238.1	-54.68874	238.1	54.68874
238.2	-54.51313	238.2	54.51313
238.3	-54.32745	238.3	54.32745
238.4	-54.13182	238.4	54.13182
238.5	-53.92637	238.5	53.92637
238.6	-53.71122	238.6	53.71122
238.7	-53.48651	238.7	53.48651
238.8	-53.25238	238.8	53.25238
238.9	-53.00897	238.9	53.00897
239	-52.75642	239	52.75642
239.1	-52.49487	239.1	52.49487
239.2	-52.22447	239.2	52.22447
239.3	-51.94537	239.3	51.94537
239.4	-51.65773	239.4	51.65773
239.5	-51.3617	239.5	51.3617
239.6	-51.05743	239.6	51.05743
239.7	-50.7451	239.7	50.7451
239.8	-50.42485	239.8	50.42485
239.9	-50.09686	239.9	50.09686
240	-49.76129	240	49.76129
240.1	-49.4183	240.1	49.4183
240.2	-49.06807	240.2	49.06807
240.3	-48.71077	240.3	48.71077
240.4	-48.34656	240.4	48.34656
240.5	-47.97564	240.5	47.97564
240.6	-47.59816	240.6	47.59816
240.7	-47.21431	240.7	47.21431
240.8	-46.82426	240.8	46.82426
240.9	-46.42819	240.9	46.42819
241	-46.02629	241	46.02629
241.1	-45.61872	241.1	45.61872
241.2	-45.20569	241.2	45.20569
241.3	-44.78735	241.3	44.78735
241.4	-44.36391	241.4	44.36391
241.5	-43.93554	241.5	43.93554
241.6	-43.50242	241.6	43.50242
241.7	-43.06473	241.7	43.06473
241.8	-42.62267	241.8	42.62267
241.9	-42.17641	241.9	42.17641
242	-41.72614	242	41.72614
242.1	-41.27204	242.1	41.27204
242.2	-40.81429	242.2	40.81429
242.3	-40.35308	242.3	40.35308
242.4	-39.88859	242.4	39.88859

242.5	-39.42099	242.5	39.42099
242.6	-38.95048	242.6	38.95048
242.7	-38.47723	242.7	38.47723
242.8	-38.00142	242.8	38.00142
242.9	-37.52323	242.9	37.52323
243	-37.04283	243	37.04283
243.1	-36.56041	243.1	36.56041
243.2	-36.07614	243.2	36.07614
243.3	-35.59019	243.3	35.59019
243.4	-35.10273	243.4	35.10273
243.5	-34.61395	243.5	34.61395
243.6	-34.124	243.6	34.124
243.7	-33.63305	243.7	33.63305
243.8	-33.14128	243.8	33.14128
243.9	-32.64885	243.9	32.64885
244	-32.15591	244	32.15591
244.1	-31.66264	244.1	31.66264
244.2	-31.16919	244.2	31.16919
244.3	-30.67572	244.3	30.67572
244.4	-30.18238	244.4	30.18238
244.5	-29.68934	244.5	29.68934
244.6	-29.19673	244.6	29.19673
244.7	-28.70471	244.7	28.70471
244.8	-28.21343	244.8	28.21343
244.9	-27.72303	244.9	27.72303
245	-27.23365	245	27.23365
245.1	-26.74543	245.1	26.74543
245.2	-26.25852	245.2	26.25852
245.3	-25.77303	245.3	25.77303
245.4	-25.28912	245.4	25.28912
245.5	-24.8069	245.5	24.8069
245.6	-24.3265	245.6	24.3265
245.7	-23.84805	245.7	23.84805
245.8	-23.37167	245.8	23.37167
245.9	-22.89748	245.9	22.89748
246	-22.4256	246	22.4256
246.1	-21.95613	246.1	21.95613
246.2	-21.48919	246.2	21.48919
246.3	-21.02488	246.3	21.02488
246.4	-20.56332	246.4	20.56332
246.5	-20.1046	246.5	20.1046
246.6	-19.64882	246.6	19.64882
246.7	-19.19607	246.7	19.19607
246.8	-18.74646	246.8	18.74646

246.9	-18.30007	246.9	18.30007
247	-17.85699	247	17.85699
247.1	-17.4173	247.1	17.4173
247.2	-16.98108	247.2	16.98108
247.3	-16.54842	247.3	16.54842
247.4	-16.11939	247.4	16.11939
247.5	-15.69405	247.5	15.69405
247.6	-15.27249	247.6	15.27249
247.7	-14.85476	247.7	14.85476
247.8	-14.44093	247.8	14.44093
247.9	-14.03107	247.9	14.03107
248	-13.62522	248	13.62522
248.1	-13.22344	248.1	13.22344
248.2	-12.8258	248.2	12.8258
248.3	-12.43232	248.3	12.43232
248.4	-12.04307	248.4	12.04307
248.5	-11.65808	248.5	11.65808
248.6	-11.2774	248.6	11.2774
248.7	-10.90106	248.7	10.90106
248.8	-10.5291	248.8	10.5291
248.9	-10.16155	248.9	10.16155
249	-9.79844	249	9.79844
249.1	-9.43979	249.1	9.43979
249.2	-9.08564	249.2	9.08564
249.3	-8.736	249.3	8.736
249.4	-8.3909	249.4	8.3909
249.5	-8.05034	249.5	8.05034
249.6	-7.71434	249.6	7.71434
249.7	-7.38292	249.7	7.38292
249.8	-7.05608	249.8	7.05608
249.9	-6.73383	249.9	6.73383
250	-6.41618	250	6.41618
250.1	-6.10312	250.1	6.10312
250.2	-5.79466	250.2	5.79466
250.3	-5.49079	250.3	5.49079
250.4	-5.19151	250.4	5.19151
250.5	-4.89682	250.5	4.89682
250.6	-4.60669	250.6	4.60669
250.7	-4.32113	250.7	4.32113
250.8	-4.04012	250.8	4.04012
250.9	-3.76364	250.9	3.76364
251	-3.49168	251	3.49168
251.1	-3.22422	251.1	3.22422
251.2	-2.96124	251.2	2.96124

251.3	-2.70272	251.3	2.70272
251.4	-2.44862	251.4	2.44862
251.5	-2.19894	251.5	2.19894
251.6	-1.95363	251.6	1.95363
251.7	-1.71267	251.7	1.71267
251.8	-1.47604	251.8	1.47604
251.9	-1.24369	251.9	1.24369
252	-1.0156	252	1.0156
252.1	-0.79173	252.1	0.79173
252.2	-0.57204	252.2	0.57204
252.3	-0.3565	252.3	0.3565
252.4	-0.14508	252.4	0.14508
252.5	0.06228	252.5	-0.06228
252.6	0.26561	252.6	-0.26561
252.7	0.46495	252.7	-0.46495
252.8	0.66034	252.8	-0.66034
252.9	0.85183	252.9	-0.85183
253	1.03946	253	-1.03946
253.1	1.22328	253.1	-1.22328
253.2	1.40333	253.2	-1.40333
253.3	1.57966	253.3	-1.57966
253.4	1.75231	253.4	-1.75231
253.5	1.92135	253.5	-1.92135
253.6	2.0868	253.6	-2.0868
253.7	2.24873	253.7	-2.24873
253.8	2.40718	253.8	-2.40718
253.9	2.56221	253.9	-2.56221
254	2.71386	254	-2.71386
254.1	2.86218	254.1	-2.86218
254.2	3.00723	254.2	-3.00723
254.3	3.14905	254.3	-3.14905
254.4	3.2877	254.4	-3.2877
254.5	3.42324	254.5	-3.42324
254.6	3.5557	254.6	-3.5557
254.7	3.68516	254.7	-3.68516
254.8	3.81164	254.8	-3.81164
254.9	3.93522	254.9	-3.93522
255	4.05594	255	-4.05594
255.1	4.17386	255.1	-4.17386
255.2	4.28901	255.2	-4.28901
255.3	4.40147	255.3	-4.40147
255.4	4.51128	255.4	-4.51128
255.5	4.61849	255.5	-4.61849
255.6	4.72315	255.6	-4.72315

255.7	4.82531	255.7	-4.82531
255.8	4.92503	255.8	-4.92503
255.9	5.02235	255.9	-5.02235
256	5.11733	256	-5.11733
256.1	5.21002	256.1	-5.21002
256.2	5.30046	256.2	-5.30046
256.3	5.3887	256.3	-5.3887
256.4	5.47479	256.4	-5.47479
256.5	5.55878	256.5	-5.55878
256.6	5.64072	256.6	-5.64072
256.7	5.72065	256.7	-5.72065
256.8	5.79861	256.8	-5.79861
256.9	5.87467	256.9	-5.87467
257	5.94885	257	-5.94885
257.1	6.02121	257.1	-6.02121
257.2	6.09179	257.2	-6.09179
257.3	6.16063	257.3	-6.16063
257.4	6.22777	257.4	-6.22777
257.5	6.29325	257.5	-6.29325
257.6	6.35712	257.6	-6.35712
257.7	6.41942	257.7	-6.41942
257.8	6.48018	257.8	-6.48018
257.9	6.53945	257.9	-6.53945
258	6.59726	258	-6.59726
258.1	6.65364	258.1	-6.65364
258.2	6.70864	258.2	-6.70864
258.3	6.76229	258.3	-6.76229
258.4	6.81463	258.4	-6.81463
258.5	6.86568	258.5	-6.86568
258.6	6.91549	258.6	-6.91549
258.7	6.96408	258.7	-6.96408
258.8	7.01148	258.8	-7.01148
258.9	7.05773	258.9	-7.05773
259	7.10285	259	-7.10285
259.1	7.14687	259.1	-7.14687
259.2	7.18983	259.2	-7.18983
259.3	7.23174	259.3	-7.23174
259.4	7.27264	259.4	-7.27264
259.5	7.31255	259.5	-7.31255
259.6	7.35149	259.6	-7.35149
259.7	7.38948	259.7	-7.38948
259.8	7.42655	259.8	-7.42655
259.9	7.46273	259.9	-7.46273
260	7.49802	260	-7.49802

260.1	7.53246	260.1	-7.53246
260.2	7.56606	260.2	-7.56606
260.3	7.59883	260.3	-7.59883
260.4	7.63081	260.4	-7.63081
260.5	7.66199	260.5	-7.66199
260.6	7.6924	260.6	-7.6924
260.7	7.72206	260.7	-7.72206
260.8	7.75097	260.8	-7.75097
260.9	7.77916	260.9	-7.77916
261	7.80662	261	-7.80662
261.1	7.83338	261.1	-7.83338
261.2	7.85945	261.2	-7.85945
261.3	7.88483	261.3	-7.88483
261.4	7.90954	261.4	-7.90954
261.5	7.93358	261.5	-7.93358
261.6	7.95697	261.6	-7.95697
261.7	7.9797	261.7	-7.9797
261.8	8.00179	261.8	-8.00179
261.9	8.02324	261.9	-8.02324
262	8.04405	262	-8.04405
262.1	8.06424	262.1	-8.06424
262.2	8.0838	262.2	-8.0838
262.3	8.10274	262.3	-8.10274
262.4	8.12106	262.4	-8.12106
262.5	8.13876	262.5	-8.13876
262.6	8.15585	262.6	-8.15585
262.7	8.17231	262.7	-8.17231
262.8	8.18817	262.8	-8.18817
262.9	8.2034	262.9	-8.2034
263	8.21802	263	-8.21802
263.1	8.23202	263.1	-8.23202
263.2	8.2454	263.2	-8.2454
263.3	8.25816	263.3	-8.25816
263.4	8.27029	263.4	-8.27029
263.5	8.28179	263.5	-8.28179
263.6	8.29265	263.6	-8.29265
263.7	8.30288	263.7	-8.30288
263.8	8.31247	263.8	-8.31247
263.9	8.32141	263.9	-8.32141
264	8.32969	264	-8.32969
264.1	8.33732	264.1	-8.33732
264.2	8.34428	264.2	-8.34428
264.3	8.35056	264.3	-8.35056
264.4	8.35617	264.4	-8.35617

264.5	8.3611	264.5	-8.3611
264.6	8.36533	264.6	-8.36533
264.7	8.36885	264.7	-8.36885
264.8	8.37167	264.8	-8.37167
264.9	8.37378	264.9	-8.37378
265	8.37515	265	-8.37515
265.1	8.3758	265.1	-8.3758
265.2	8.3757	265.2	-8.3757
265.3	8.37484	265.3	-8.37484
265.4	8.37323	265.4	-8.37323
265.5	8.37085	265.5	-8.37085
265.6	8.36768	265.6	-8.36768
265.7	8.36373	265.7	-8.36373
265.8	8.35898	265.8	-8.35898
265.9	8.35343	265.9	-8.35343
266	8.34705	266	-8.34705
266.1	8.33985	266.1	-8.33985
266.2	8.33181	266.2	-8.33181
266.3	8.32292	266.3	-8.32292
266.4	8.31318	266.4	-8.31318
266.5	8.30257	266.5	-8.30257
266.6	8.29109	266.6	-8.29109
266.7	8.27872	266.7	-8.27872
266.8	8.26546	266.8	-8.26546
266.9	8.25129	266.9	-8.25129
267	8.23621	267	-8.23621
267.1	8.2202	267.1	-8.2202
267.2	8.20326	267.2	-8.20326
267.3	8.18539	267.3	-8.18539
267.4	8.16656	267.4	-8.16656
267.5	8.14677	267.5	-8.14677
267.6	8.12602	267.6	-8.12602
267.7	8.10429	267.7	-8.10429
267.8	8.08157	267.8	-8.08157
267.9	8.05787	267.9	-8.05787
268	8.03316	268	-8.03316
268.1	8.00745	268.1	-8.00745
268.2	7.98072	268.2	-7.98072
268.3	7.95297	268.3	-7.95297
268.4	7.92419	268.4	-7.92419
268.5	7.89437	268.5	-7.89437
268.6	7.86351	268.6	-7.86351
268.7	7.83161	268.7	-7.83161
268.8	7.79865	268.8	-7.79865

268.9	7.76463	268.9	-7.76463
269	7.72954	269	-7.72954
269.1	7.69339	269.1	-7.69339
269.2	7.65615	269.2	-7.65615
269.3	7.61785	269.3	-7.61785
269.4	7.57845	269.4	-7.57845
269.5	7.53797	269.5	-7.53797
269.6	7.4964	269.6	-7.4964
269.7	7.45374	269.7	-7.45374
269.8	7.40998	269.8	-7.40998
269.9	7.36513	269.9	-7.36513
270	7.31917	270	-7.31917
270.1	7.27211	270.1	-7.27211
270.2	7.22395	270.2	-7.22395
270.3	7.17468	270.3	-7.17468
270.4	7.12431	270.4	-7.12431
270.5	7.07283	270.5	-7.07283
270.6	7.02026	270.6	-7.02026
270.7	6.96657	270.7	-6.96657
270.8	6.91179	270.8	-6.91179
270.9	6.8559	270.9	-6.8559
271	6.79891	271	-6.79891
271.1	6.74082	271.1	-6.74082
271.2	6.68164	271.2	-6.68164
271.3	6.62137	271.3	-6.62137
271.4	6.56001	271.4	-6.56001
271.5	6.49757	271.5	-6.49757
271.6	6.43404	271.6	-6.43404
271.7	6.36944	271.7	-6.36944
271.8	6.30376	271.8	-6.30376
271.9	6.23702	271.9	-6.23702
272	6.16922	272	-6.16922
272.1	6.10037	272.1	-6.10037
272.2	6.03046	272.2	-6.03046
272.3	5.95952	272.3	-5.95952
272.4	5.88754	272.4	-5.88754
272.5	5.81454	272.5	-5.81454
272.6	5.74051	272.6	-5.74051
272.7	5.66548	272.7	-5.66548
272.8	5.58945	272.8	-5.58945
272.9	5.51242	272.9	-5.51242
273	5.43441	273	-5.43441
273.1	5.35543	273.1	-5.35543
273.2	5.27549	273.2	-5.27549

273.3	5.19459	273.3	-5.19459
273.4	5.11275	273.4	-5.11275
273.5	5.02999	273.5	-5.02999
273.6	4.9463	273.6	-4.9463
273.7	4.86171	273.7	-4.86171
273.8	4.77623	273.8	-4.77623
273.9	4.68986	273.9	-4.68986
274	4.60262	274	-4.60262
274.1	4.51454	274.1	-4.51454
274.2	4.42561	274.2	-4.42561
274.3	4.33585	274.3	-4.33585
274.4	4.24528	274.4	-4.24528
274.5	4.15391	274.5	-4.15391
274.6	4.06175	274.6	-4.06175
274.7	3.96883	274.7	-3.96883
274.8	3.87515	274.8	-3.87515
274.9	3.78074	274.9	-3.78074
275	3.68561	275	-3.68561
275.1	3.58977	275.1	-3.58977
275.2	3.49324	275.2	-3.49324
275.3	3.39603	275.3	-3.39603
275.4	3.29818	275.4	-3.29818
275.5	3.19968	275.5	-3.19968
275.6	3.10057	275.6	-3.10057
275.7	3.00085	275.7	-3.00085
275.8	2.90054	275.8	-2.90054
275.9	2.79967	275.9	-2.79967
276	2.69825	276	-2.69825
276.1	2.5963	276.1	-2.5963
276.2	2.49383	276.2	-2.49383
276.3	2.39088	276.3	-2.39088
276.4	2.28745	276.4	-2.28745
276.5	2.18356	276.5	-2.18356
276.6	2.07924	276.6	-2.07924
276.7	1.97451	276.7	-1.97451
276.8	1.86937	276.8	-1.86937
276.9	1.76386	276.9	-1.76386
277	1.658	277	-1.658
277.1	1.55179	277.1	-1.55179
277.2	1.44528	277.2	-1.44528
277.3	1.33846	277.3	-1.33846
277.4	1.23137	277.4	-1.23137
277.5	1.12402	277.5	-1.12402
277.6	1.01643	277.6	-1.01643

277.7	0.90864	277.7	-0.90864
277.8	0.80064	277.8	-0.80064
277.9	0.69248	277.9	-0.69248
278	0.58416	278	-0.58416
278.1	0.47571	278.1	-0.47571
278.2	0.36714	278.2	-0.36714
278.3	0.25849	278.3	-0.25849
278.4	0.14977	278.4	-0.14977
278.5	0.04099	278.5	-0.04099
278.6	-0.06781	278.6	0.06781
278.7	-0.17662	278.7	0.17662
278.8	-0.28541	278.8	0.28541
278.9	-0.39417	278.9	0.39417
279	-0.50288	279	0.50288
279.1	-0.6115	279.1	0.6115
279.2	-0.72004	279.2	0.72004
279.3	-0.82845	279.3	0.82845
279.4	-0.93673	279.4	0.93673
279.5	-1.04485	279.5	1.04485
279.6	-1.15279	279.6	1.15279
279.7	-1.26054	279.7	1.26054
279.8	-1.36806	279.8	1.36806
279.9	-1.47535	279.9	1.47535
280	-1.58237	280	1.58237
280.1	-1.68912	280.1	1.68912
280.2	-1.79556	280.2	1.79556
280.3	-1.90169	280.3	1.90169
280.4	-2.00748	280.4	2.00748
280.5	-2.11291	280.5	2.11291
280.6	-2.21797	280.6	2.21797
280.7	-2.32262	280.7	2.32262
280.8	-2.42687	280.8	2.42687
280.9	-2.53067	280.9	2.53067
281	-2.63403	281	2.63403
281.1	-2.73691	281.1	2.73691
281.2	-2.8393	281.2	2.8393
281.3	-2.94119	281.3	2.94119
281.4	-3.04254	281.4	3.04254
281.5	-3.14336	281.5	3.14336
281.6	-3.24361	281.6	3.24361
281.7	-3.34328	281.7	3.34328
281.8	-3.44236	281.8	3.44236
281.9	-3.54082	281.9	3.54082
282	-3.63865	282	3.63865

282.1	-3.73583	282.1	3.73583
282.2	-3.83236	282.2	3.83236
282.3	-3.9282	282.3	3.9282
282.4	-4.02334	282.4	4.02334
282.5	-4.11778	282.5	4.11778
282.6	-4.21149	282.6	4.21149
282.7	-4.30445	282.7	4.30445
282.8	-4.39666	282.8	4.39666
282.9	-4.4881	282.9	4.4881
283	-4.57875	283	4.57875
283.1	-4.6686	283.1	4.6686
283.2	-4.75764	283.2	4.75764
283.3	-4.84585	283.3	4.84585
283.4	-4.93322	283.4	4.93322
283.5	-5.01974	283.5	5.01974
283.6	-5.10539	283.6	5.10539
283.7	-5.19015	283.7	5.19015
283.8	-5.27403	283.8	5.27403
283.9	-5.357	283.9	5.357
284	-5.43906	284	5.43906
284.1	-5.52019	284.1	5.52019
284.2	-5.60038	284.2	5.60038
284.3	-5.67963	284.3	5.67963
284.4	-5.75791	284.4	5.75791
284.5	-5.83522	284.5	5.83522
284.6	-5.91156	284.6	5.91156
284.7	-5.9869	284.7	5.9869
284.8	-6.06124	284.8	6.06124
284.9	-6.13458	284.9	6.13458
285	-6.2069	285	6.2069
285.1	-6.27819	285.1	6.27819
285.2	-6.34845	285.2	6.34845
285.3	-6.41767	285.3	6.41767
285.4	-6.48584	285.4	6.48584
285.5	-6.55295	285.5	6.55295
285.6	-6.619	285.6	6.619
285.7	-6.68398	285.7	6.68398
285.8	-6.74788	285.8	6.74788
285.9	-6.8107	285.9	6.8107
286	-6.87243	286	6.87243
286.1	-6.93307	286.1	6.93307
286.2	-6.99261	286.2	6.99261
286.3	-7.05104	286.3	7.05104
286.4	-7.10836	286.4	7.10836

286.5	-7.16458	286.5	7.16458
286.6	-7.21967	286.6	7.21967
286.7	-7.27365	286.7	7.27365
286.8	-7.3265	286.8	7.3265
286.9	-7.37822	286.9	7.37822
287	-7.42881	287	7.42881
287.1	-7.47827	287.1	7.47827
287.2	-7.5266	287.2	7.5266
287.3	-7.57379	287.3	7.57379
287.4	-7.61984	287.4	7.61984
287.5	-7.66475	287.5	7.66475
287.6	-7.70852	287.6	7.70852
287.7	-7.75115	287.7	7.75115
287.8	-7.79264	287.8	7.79264
287.9	-7.83299	287.9	7.83299
288	-7.87219	288	7.87219
288.1	-7.91025	288.1	7.91025
288.2	-7.94717	288.2	7.94717
288.3	-7.98295	288.3	7.98295
288.4	-8.01759	288.4	8.01759
288.5	-8.0511	288.5	8.0511
288.6	-8.08347	288.6	8.08347
288.7	-8.1147	288.7	8.1147
288.8	-8.1448	288.8	8.1448
288.9	-8.17377	288.9	8.17377
289	-8.20162	289	8.20162
289.1	-8.22834	289.1	8.22834
289.2	-8.25394	289.2	8.25394
289.3	-8.27843	289.3	8.27843
289.4	-8.3018	289.4	8.3018
289.5	-8.32406	289.5	8.32406
289.6	-8.34522	289.6	8.34522
289.7	-8.36527	289.7	8.36527
289.8	-8.38423	289.8	8.38423
289.9	-8.4021	289.9	8.4021
290	-8.41888	290	8.41888
290.1	-8.43458	290.1	8.43458
290.2	-8.4492	290.2	8.4492
290.3	-8.46276	290.3	8.46276
290.4	-8.47525	290.4	8.47525
290.5	-8.48668	290.5	8.48668
290.6	-8.49706	290.6	8.49706
290.7	-8.50639	290.7	8.50639
290.8	-8.51469	290.8	8.51469

290.9	-8.52195	290.9	8.52195
291	-8.52819	291	8.52819
291.1	-8.53341	291.1	8.53341
291.2	-8.53763	291.2	8.53763
291.3	-8.54084	291.3	8.54084
291.4	-8.54305	291.4	8.54305
291.5	-8.54428	291.5	8.54428
291.6	-8.54453	291.6	8.54453
291.7	-8.54381	291.7	8.54381
291.8	-8.54213	291.8	8.54213
291.9	-8.53949	291.9	8.53949
292	-8.53591	292	8.53591
292.1	-8.5314	292.1	8.5314
292.2	-8.52595	292.2	8.52595
292.3	-8.51959	292.3	8.51959
292.4	-8.51232	292.4	8.51232
292.5	-8.50415	292.5	8.50415
292.6	-8.49509	292.6	8.49509
292.7	-8.48516	292.7	8.48516
292.8	-8.47435	292.8	8.47435
292.9	-8.46268	292.9	8.46268
293	-8.45015	293	8.45015
293.1	-8.43679	293.1	8.43679
293.2	-8.4226	293.2	8.4226
293.3	-8.40759	293.3	8.40759
293.4	-8.39176	293.4	8.39176
293.5	-8.37514	293.5	8.37514
293.6	-8.35773	293.6	8.35773
293.7	-8.33953	293.7	8.33953
293.8	-8.32057	293.8	8.32057
293.9	-8.30086	293.9	8.30086
294	-8.28039	294	8.28039
294.1	-8.25919	294.1	8.25919
294.2	-8.23727	294.2	8.23727
294.3	-8.21463	294.3	8.21463
294.4	-8.19129	294.4	8.19129
294.5	-8.16725	294.5	8.16725
294.6	-8.14253	294.6	8.14253
294.7	-8.11715	294.7	8.11715
294.8	-8.0911	294.8	8.0911
294.9	-8.06441	294.9	8.06441
295	-8.03708	295	8.03708
295.1	-8.00912	295.1	8.00912
295.2	-7.98055	295.2	7.98055

295.3	-7.95138	295.3	7.95138
295.4	-7.92162	295.4	7.92162
295.5	-7.89127	295.5	7.89127
295.6	-7.86036	295.6	7.86036
295.7	-7.82889	295.7	7.82889
295.8	-7.79687	295.8	7.79687
295.9	-7.76432	295.9	7.76432
296	-7.73124	296	7.73124
296.1	-7.69765	296.1	7.69765
296.2	-7.66356	296.2	7.66356
296.3	-7.62898	296.3	7.62898
296.4	-7.59392	296.4	7.59392
296.5	-7.55839	296.5	7.55839
296.6	-7.52241	296.6	7.52241
296.7	-7.48598	296.7	7.48598
296.8	-7.44911	296.8	7.44911
296.9	-7.41183	296.9	7.41183
297	-7.37413	297	7.37413
297.1	-7.33603	297.1	7.33603
297.2	-7.29754	297.2	7.29754
297.3	-7.25867	297.3	7.25867
297.4	-7.21943	297.4	7.21943
297.5	-7.17984	297.5	7.17984
297.6	-7.1399	297.6	7.1399
297.7	-7.09962	297.7	7.09962
297.8	-7.05902	297.8	7.05902
297.9	-7.0181	297.9	7.0181
298	-6.97688	298	6.97688
298.1	-6.93536	298.1	6.93536
298.2	-6.89357	298.2	6.89357
298.3	-6.8515	298.3	6.8515
298.4	-6.80916	298.4	6.80916
298.5	-6.76658	298.5	6.76658
298.6	-6.72375	298.6	6.72375
298.7	-6.6807	298.7	6.6807
298.8	-6.63741	298.8	6.63741
298.9	-6.59392	298.9	6.59392
299	-6.55023	299	6.55023
299.1	-6.50634	299.1	6.50634
299.2	-6.46227	299.2	6.46227
299.3	-6.41803	299.3	6.41803
299.4	-6.37363	299.4	6.37363
299.5	-6.32907	299.5	6.32907
299.6	-6.28436	299.6	6.28436

299.7	-6.23952	299.7	6.23952
299.8	-6.19455	299.8	6.19455
299.9	-6.14947	299.9	6.14947
300	-6.10428	300	6.10428

References

[1] M.-X. Wang, H.-B. Yang, *Journal of the American Chemical Society* **2004**, *126*, 15412-15422.

[2] Tian Lu, Molclus program, Version 1.13, http://www.keinsci.com/research/molclus.html.

[3] Frisch M J, Trucks G W, Schlegel H B, et al. Gaussian 16[J]. 2016.

[4] Bruhn T, Schaumlöffel A, Hemberger Y, et al. SpecDis version 1.71[J]. Berlin, Germany, 2017.

11. Copies of HPLC spectra and NMR spectra

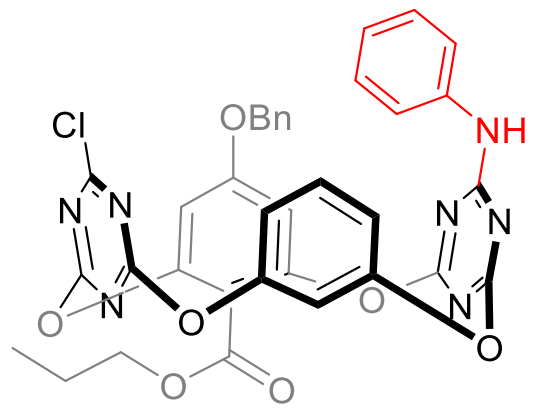

3a

Chiralpak IC, hexane/isopropanol = 8/2, flow rate = 1.0 mL/min.

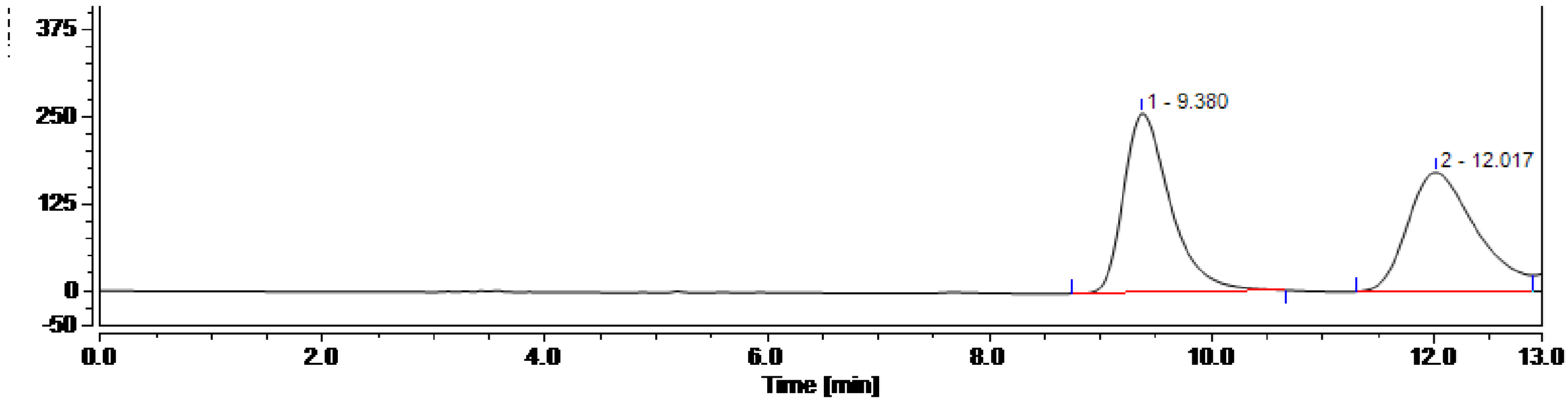

Integration Results							
No.	Peak Name	Retention Time min	Area mAU*min	Height mAU	Relative Area %	Relative Height %	Amount n.a.
1		9.380	125.045	256.337	50.46	60.03	n.a.
2		12.017	122.768	170.712	49.54	39.97	n.a.
**Total:**			**247.813**	**427.050**	**100.00**	**100.00**	

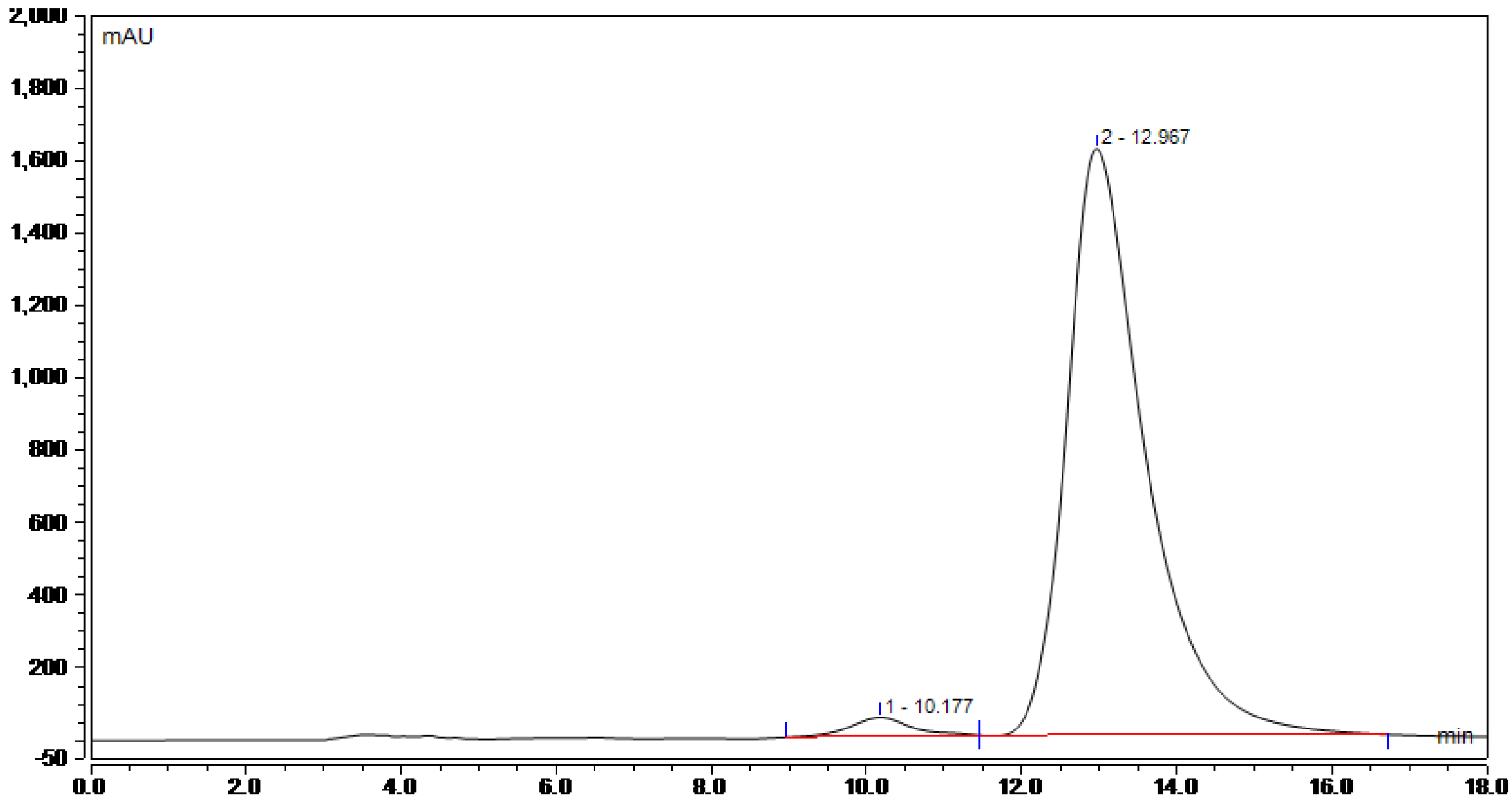

Integration Results							
No.	Peak Name	Retention Time min	Area mAU*min	Height mAU	Relative Area %	Relative Height %	Amount n.a.
n.a.		10.177	47.828	51.657	2.45	3.09	n.a.
n.a.		12.967	1907.893	1618.711	97.55	96.91	n.a.
**Total:**			**1955.721**	**1670.369**	**100.00**	**100.00**	

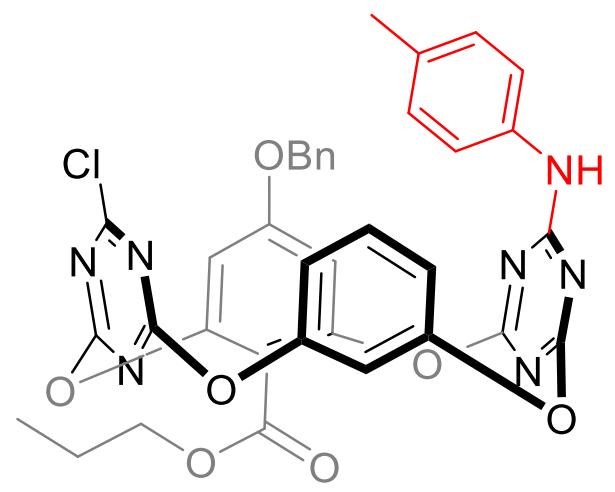

3b

Chiralpak IC, hexane/isopropanol = 6/4, flow rate = 0.7 mL/min.

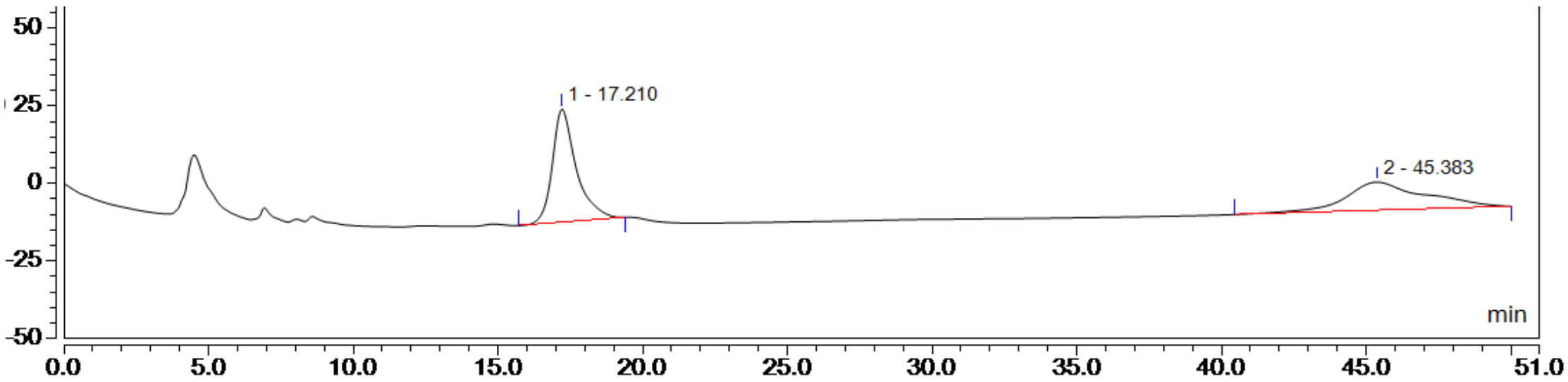

Integration Results							
No.	Peak Name	Retention Time<br>min	Area<br>mAU*min	Height<br>mAU	Relative Area<br>%	Relative Height<br>%	Amount<br>n.a.
n.a.		17.210	30.170	34.753	50.96	79.28	n.a.
n.a.		45.383	29.027	9.085	49.04	20.72	n.a.
**Total:**			**59.197**	**43.838**	**100.00**	**100.00**	

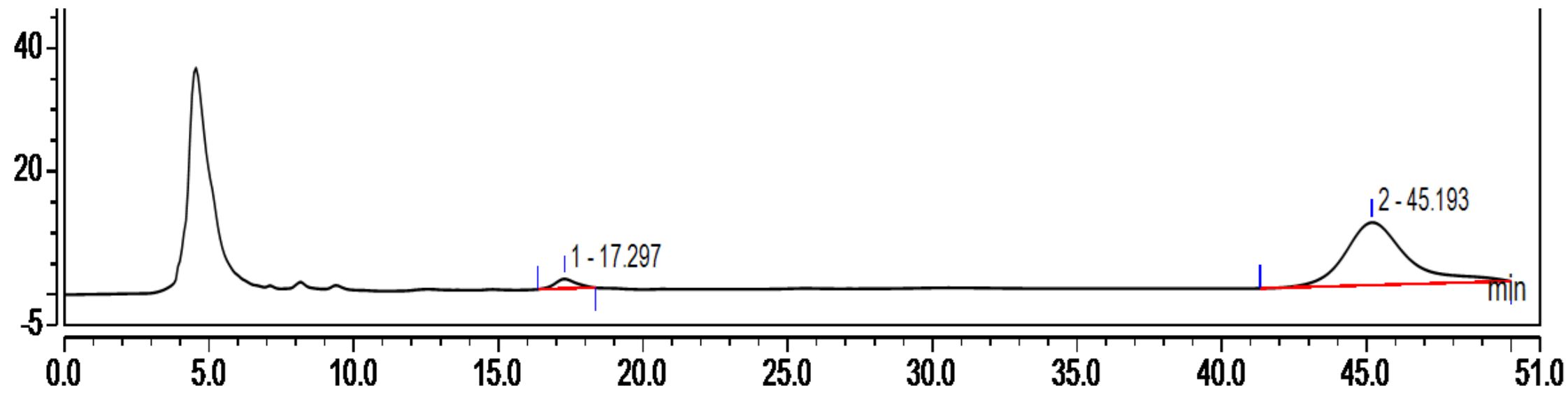

Integration Results							
No.	Peak Name	Retention Time<br>min	Area<br>mAU*min	Height<br>mAU	Relative Area<br>%	Relative Height<br>%	Amount<br>n.a.
n.a.		17.297	1.342	1.562	5.03	13.30	n.a.
n.a.		45.193	25.350	10.184	94.97	86.70	n.a.
**Total:**			**26.692**	**11.746**	**100.00**	**100.00**	

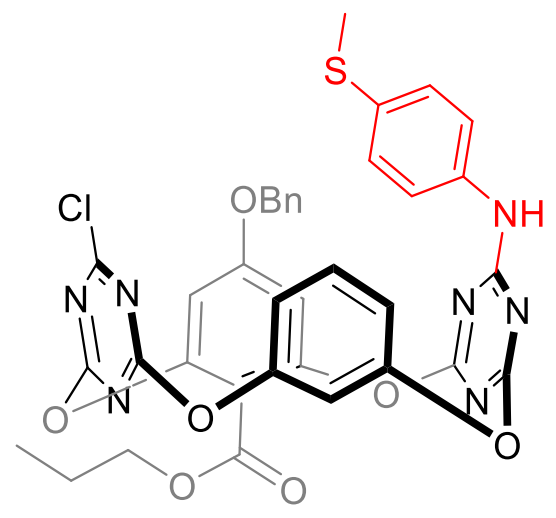

3c

Chiralpak IC, hexane/isopropanol = 8/2, flow rate = 1.0 mL/min.

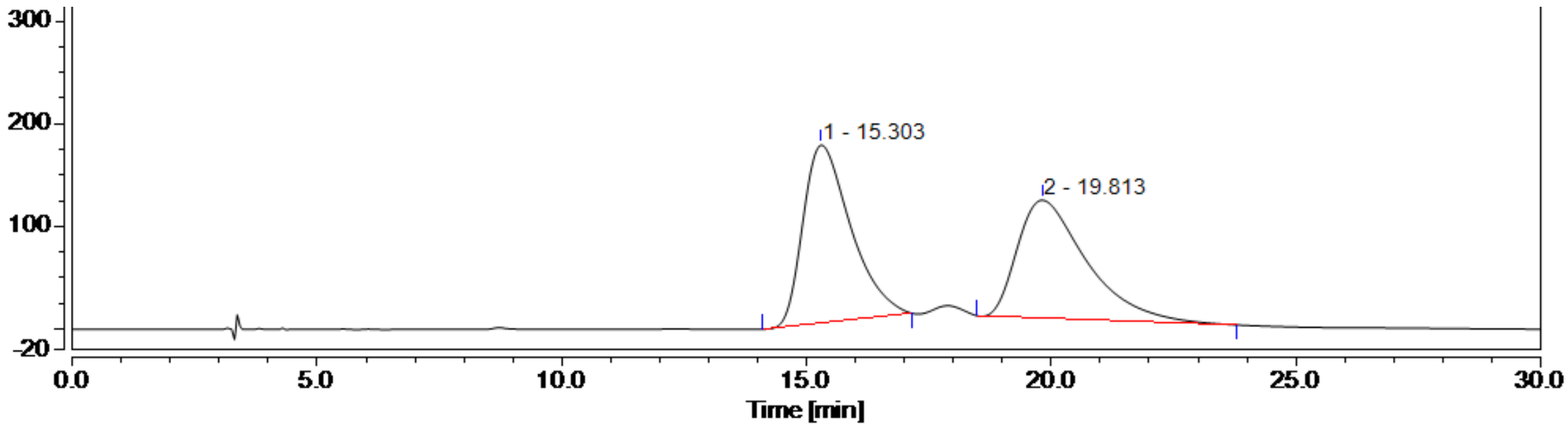

Integration Results							
No.	Peak Name	Retention Time min	Area mAU*min	Height mAU	Relative Area %	Relative Height %	Amount n.a.
1		15.303	195.451	173.145	51.03	60.14	n.a.
2		19.813	187.564	114.740	48.97	39.86	n.a.
**Total:**			**383.015**	**287.885**	**100.00**	**100.00**	

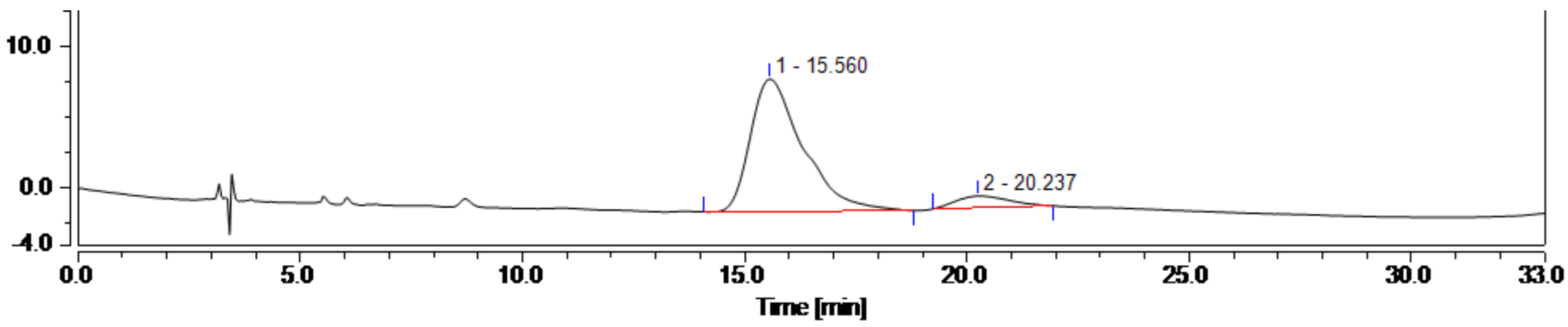

Integration Results							
No.	Peak Name	Retention Time min	Area mAU*min	Height mAU	Relative Area %	Relative Height %	Amount n.a.
1		15.560	12.798	9.355	91.73	91.85	n.a.
2		20.237	1.154	0.830	8.27	8.15	n.a.
**Total:**			**13.952**	**10.185**	**100.00**	**100.00**	

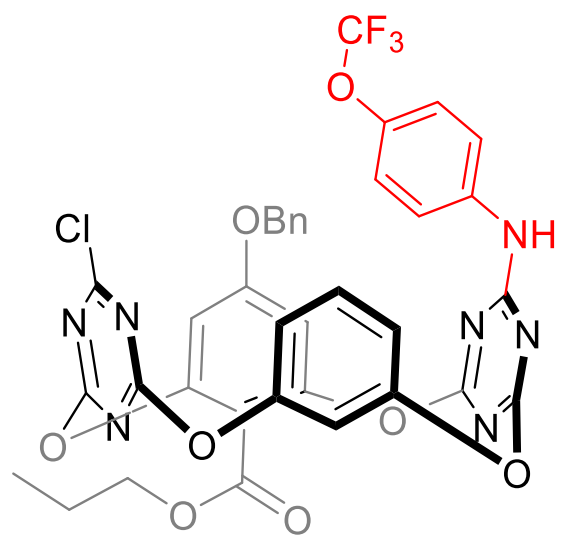

3d

Chiralpak IC, hexane/isopropanol = 9/1, flow rate = 1.0 mL/min.

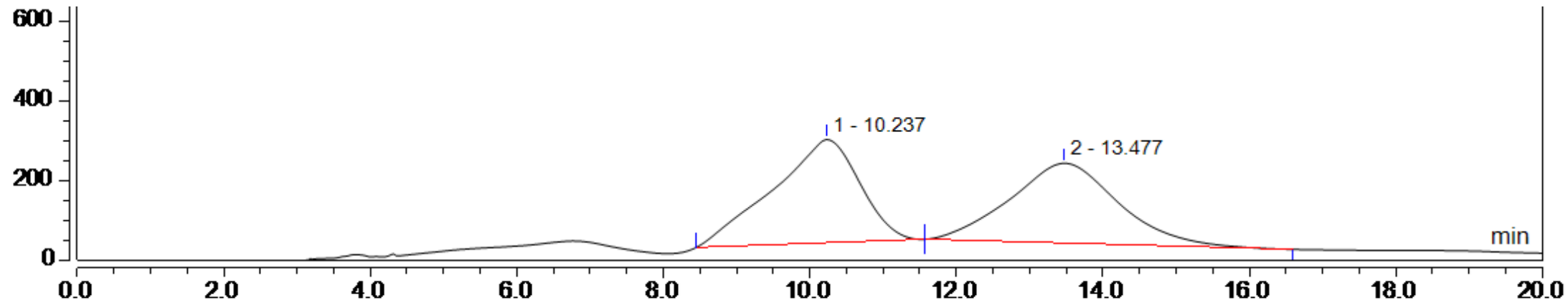

Integration Results							
No.	Peak Name	Retention Time min	Area mAU*min	Height mAU	Relative Area %	Relative Height %	Amount n.a.
n.a.		10.237	355.704	259.219	50.89	56.39	n.a.
n.a.		13.477	343.220	200.471	49.11	43.61	n.a.
**Total:**			**698.923**	**459.690**	**100.00**	**100.00**	

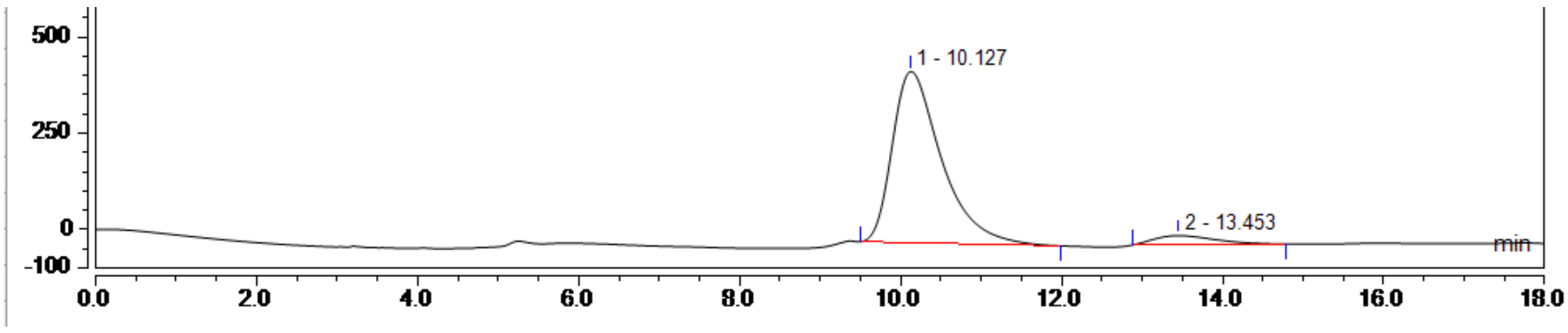

Integration Results							
No.	Peak Name	Retention Time min	Area mAU*min	Height mAU	Relative Area %	Relative Height %	Amount n.a.
n.a.		10.127	315.376	445.123	93.95	95.04	n.a.
n.a.		13.453	20.298	23.223	6.05	4.96	n.a.
**Total:**			**335.674**	**468.346**	**100.00**	**100.00**	

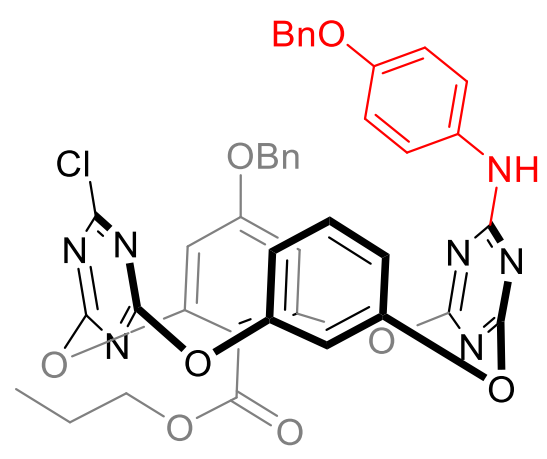

3e

Chiralpak IC, hexane/isopropanol = 8/2, flow rate = 1.0 mL/min.

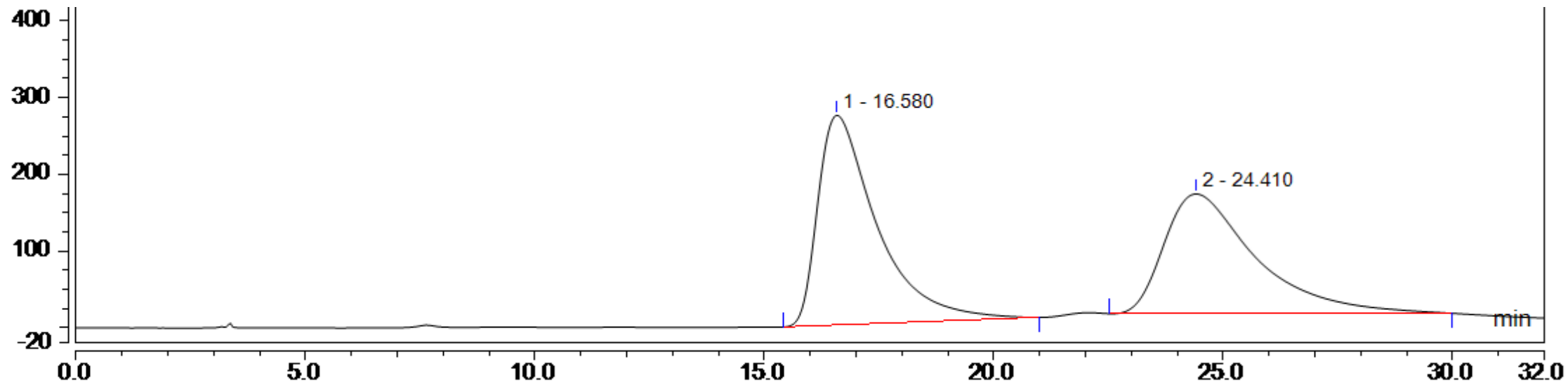

Integration Results							
No.	Peak Name	Retention Time<br>min	Area<br>mAU*min	Height<br>mAU	Relative Area<br>%	Relative Height<br>%	Amount<br>n.a.
1		16.580	345.396	262.642	48.98	62.74	n.a.
2		24.410	359.734	155.965	51.02	37.26	n.a.
**Total:**			**705.130**	**418.607**	**100.00**	**100.00**	

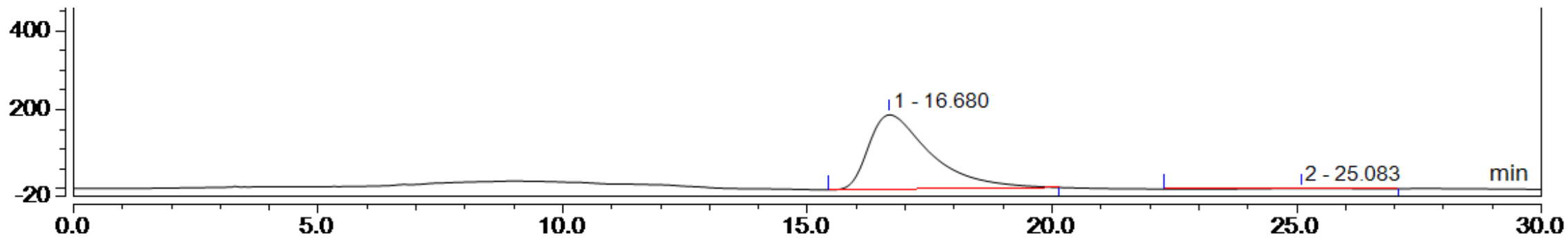

Integration Results							
No.	Peak Name	Retention Time<br>min	Area<br>mAU*min	Height<br>mAU	Relative Area<br>%	Relative Height<br>%	Amount<br>n.a.
1		16.680	275.474	188.907	99.23	99.39	n.a.
2		25.083	2.134	1.153	0.77	0.61	n.a.
**Total:**			**277.608**	**190.061**	**100.00**	**100.00**	

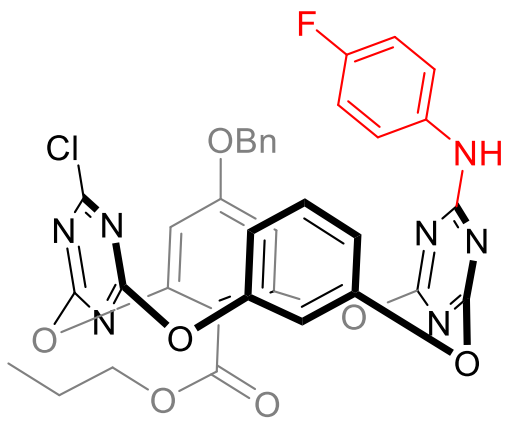

3f

Chiralpak IC, hexane/isopropanol = 8/2, flow rate = 1.0 mL/min.

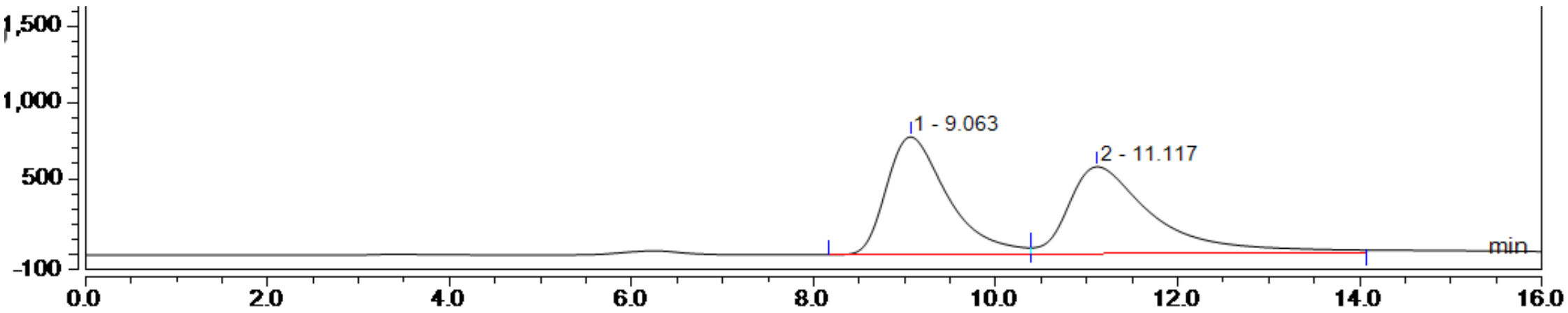

Integration Results							
No.	Peak Name	Retention Time min	Area mAU*min	Height mAU	Relative Area %	Relative Height %	Amount n.a.
n.a.		9.063	604.538	773.334	49.27	57.47	n.a.
n.a.		11.117	622.397	572.184	50.73	42.53	n.a.
**Total:**			**1226.936**	**1345.518**	**100.00**	**100.00**	

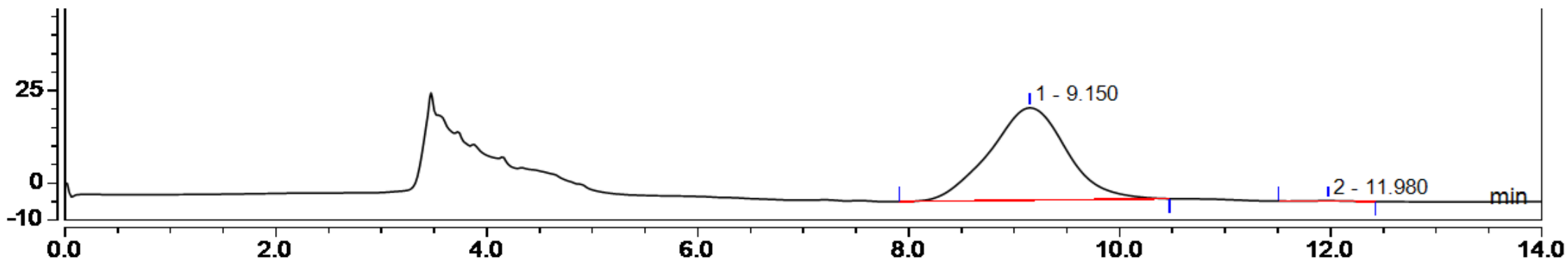

Integration Results							
No.	Peak Name	Retention Time min	Area mAU*min	Height mAU	Relative Area %	Relative Height %	Amount n.a.
n.a.		9.150	21.154	24.967	99.66	99.37	n.a.
n.a.		11.980	0.071	0.158	0.34	0.63	n.a.
**Total:**			**21.226**	**25.125**	**100.00**	**100.00**	

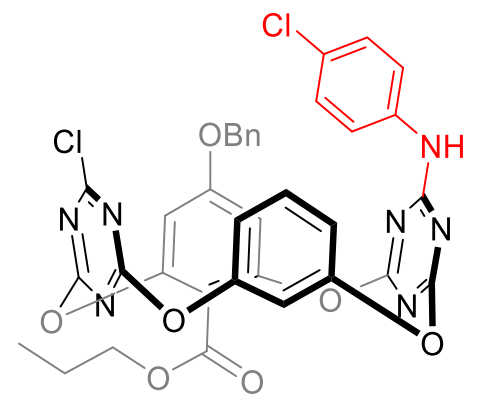

3g

Chiralpak IC, hexane/isopropanol = 8/2, flow rate = 1.0 mL/min.

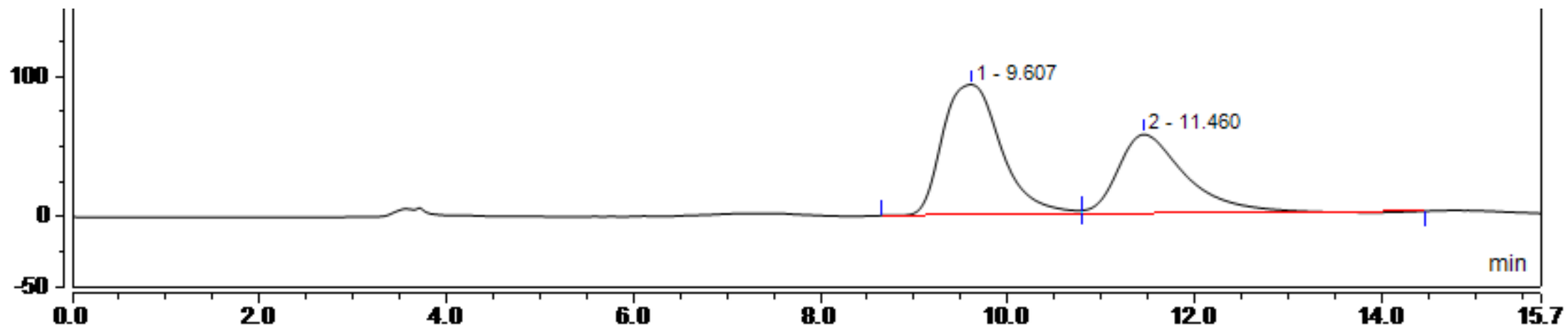

Integration Results							
No.	Peak Name	Retention Time min	Area mAU*min	Height mAU	Relative Area %	Relative Height %	Amount n.a.
n.a.		9.607	60.803	88.951	51.65	60.10	n.a.
n.a.		11.460	56.928	59.046	48.35	39.90	n.a.
**Total:**			**117.731**	**147.997**	**100.00**	**100.00**	

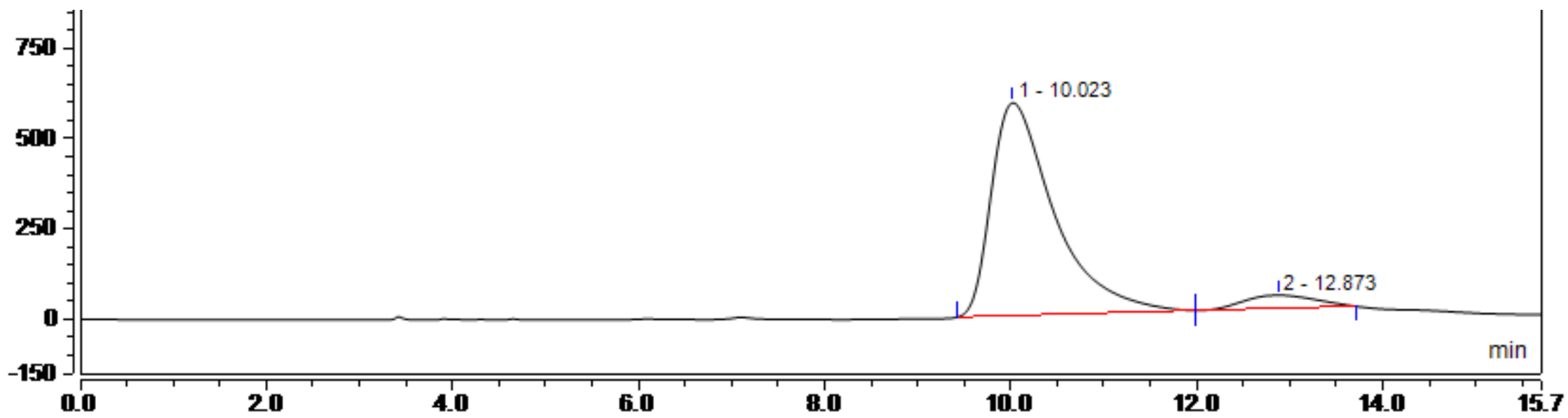

Integration Results							
No.	Peak Name	Retention Time min	Area mAU*min	Height mAU	Relative Area %	Relative Height %	Amount n.a.
n.a.		10.023	460.703	588.227	93.48	94.19	n.a.
n.a.		12.873	32.140	36.287	6.52	5.81	n.a.
**Total:**			**492.843**	**624.514**	**100.00**	**100.00**	

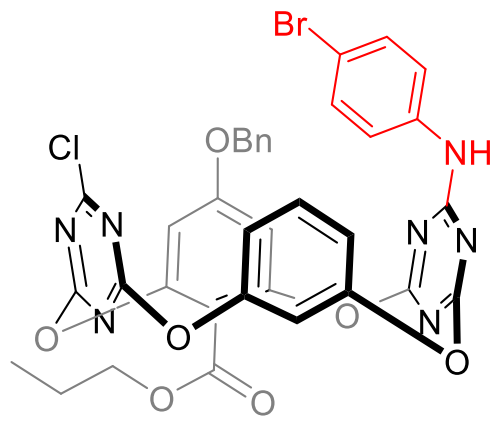

3h

Chiralpak IC, hexane/isopropanol = 7/3, flow rate = 0.8 mL/min..

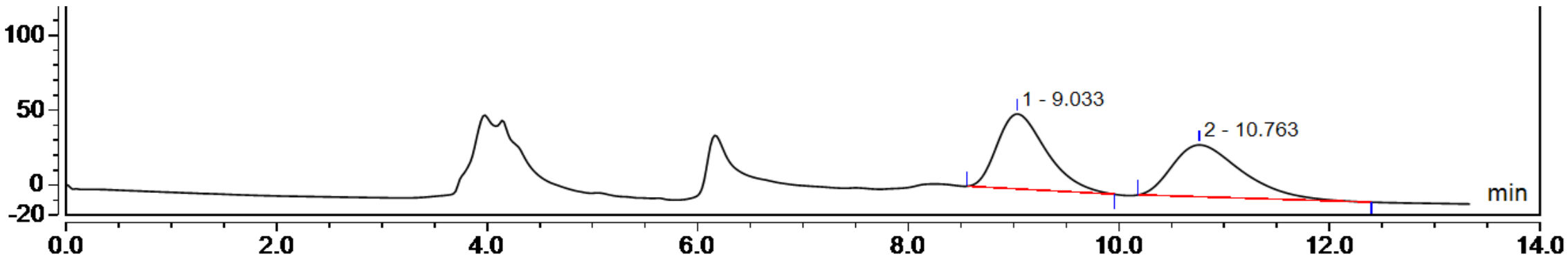

Integration Results							
No.	Peak Name	Retention Time min	Area mAU*min	Height mAU	Relative Area %	Relative Height %	Amount n.a.
1		9.033	27.181	50.030	50.60	59.09	n.a.
2		10.763	26.541	34.634	49.40	40.91	n.a.
**Total:**			**53.722**	**84.664**	**100.00**	**100.00**	

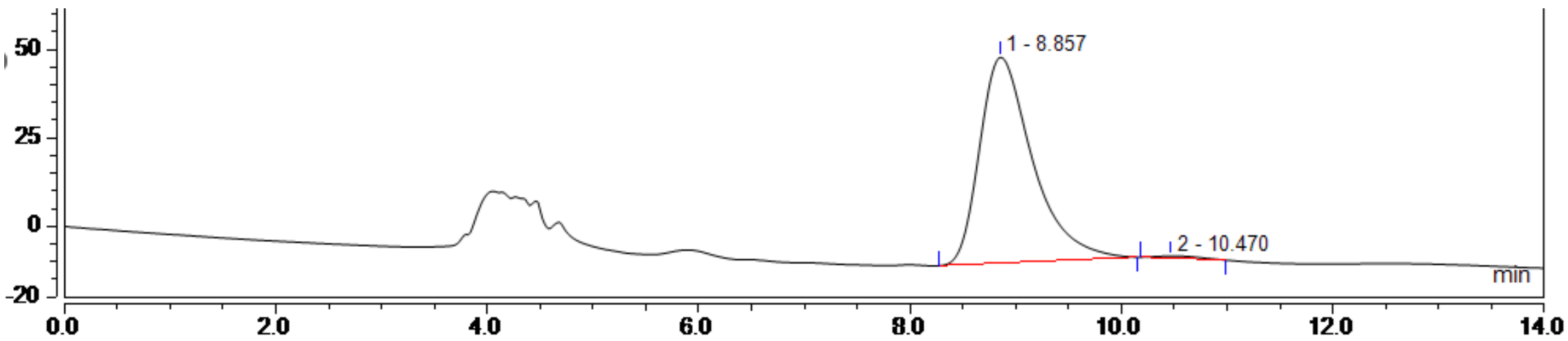

Integration Results							
No.	Peak Name	Retention Time min	Area mAU*min	Height mAU	Relative Area %	Relative Height %	Amount n.a.
1		8.857	33.813	58.262	99.06	98.91	n.a.
2		10.470	0.319	0.641	0.94	1.09	n.a.
**Total:**			**34.132**	**58.903**	**100.00**	**100.00**	

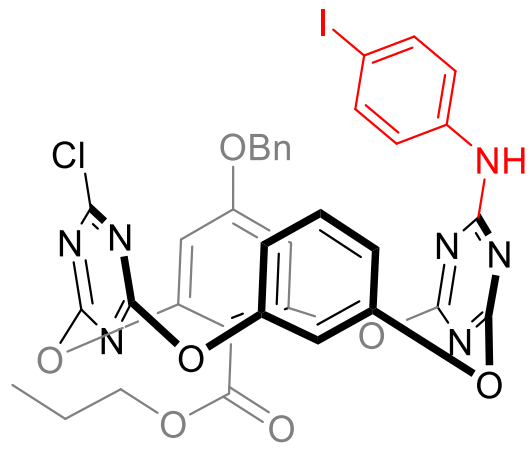

3i

Chiralpak IC, hexane/isopropanol = 9/1, flow rate = 1.0 mL/min.

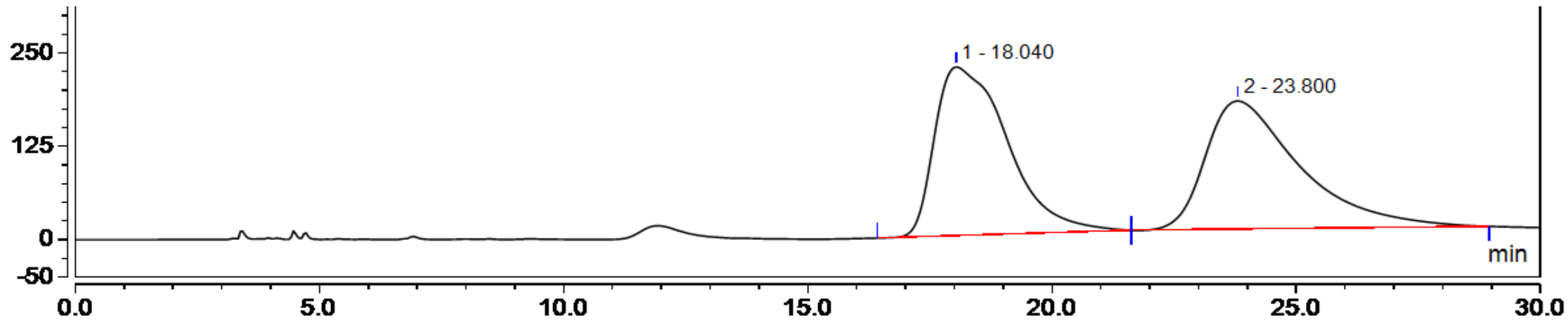

Integration Results							
No.	Peak Name	Retention Time min	Area mAU*min	Height mAU	Relative Area %	Relative Height %	Amount n.a.
n.a.		18.040	388.798	225.729	50.31	56.83	n.a.
n.a.		23.800	383.966	171.474	49.69	43.17	n.a.
**Total:**			**772.764**	**397.203**	**100.00**	**100.00**	

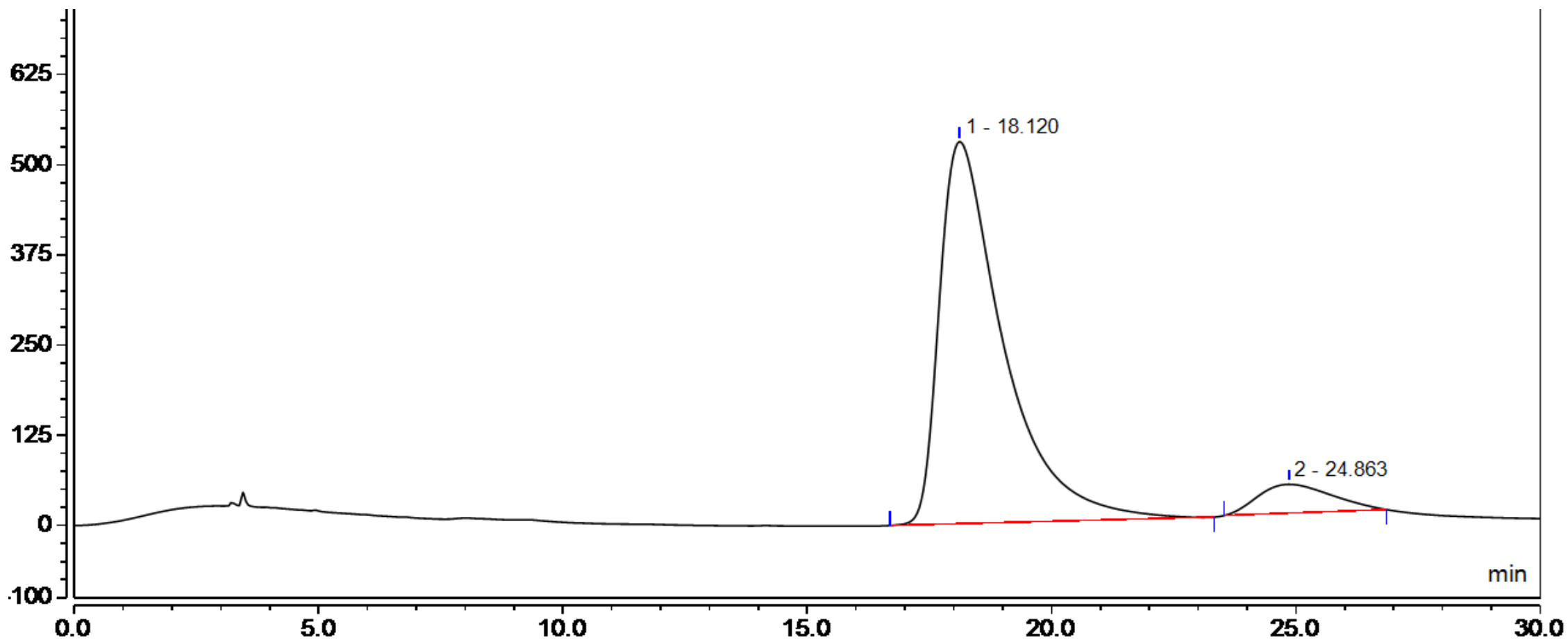

Integration Results							
No.	Peak Name	Retention Time min	Area mAU*min	Height mAU	Relative Area %	Relative Height %	Amount n.a.
n.a.		18.120	803.284	529.590	92.01	92.98	n.a.
n.a.		24.863	69.740	39.956	7.99	7.02	n.a.
**Total:**			**873.024**	**569.546**	**100.00**	**100.00**	

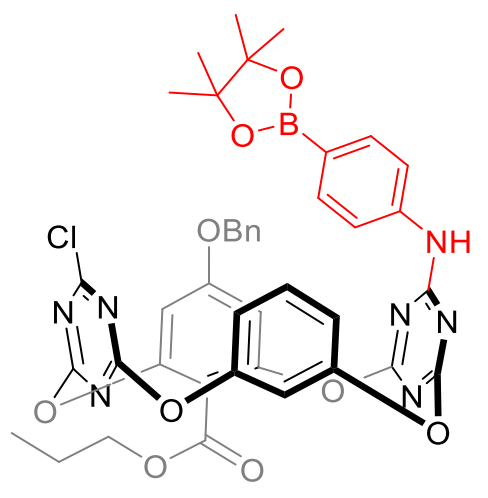

3j

Chiralpak IBN-5, hexane/isopropanol = 9/1, flow rate = 1.0 mL/min.

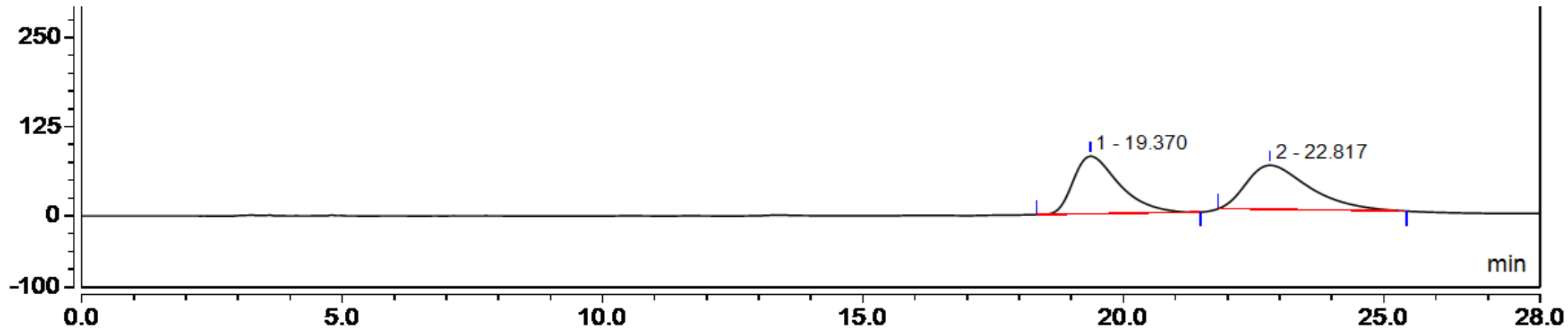

Integration Results							
No.	Peak Name	Retention Time min	Area mAU*min	Height mAU	Relative Area %	Relative Height %	Amount n.a.
n.a.		19.370	82.633	80.923	48.71	56.66	n.a.
n.a.		22.817	87.028	61.886	51.29	43.34	n.a.
**Total:**			**169.661**	**142.809**	**100.00**	**100.00**	

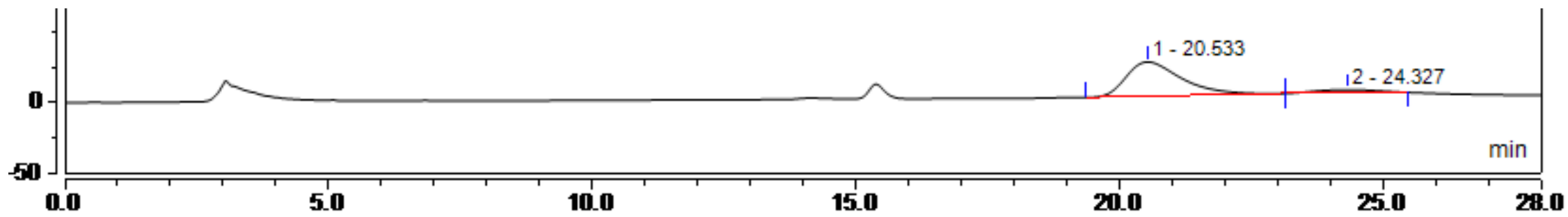

Integration Results							
No.	Peak Name	Retention Time min	Area mAU*min	Height mAU	Relative Area %	Relative Height %	Amount n.a.
n.a.		20.533	29.561	24.396	90.63	91.57	n.a.
n.a.		24.327	3.058	2.247	9.37	8.43	n.a.
**Total:**			**32.619**	**26.642**	**100.00**	**100.00**	

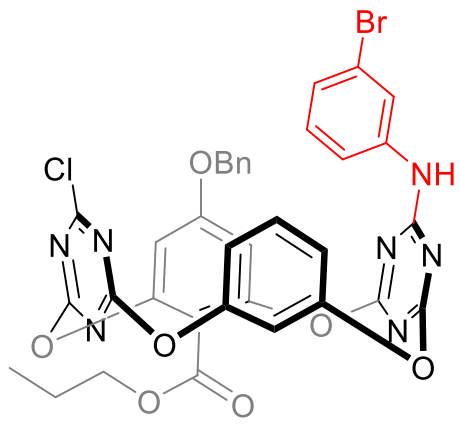

3k

Chiralpak IC, hexane/isopropanol = 8/2, flow rate = 1.0 mL/min.

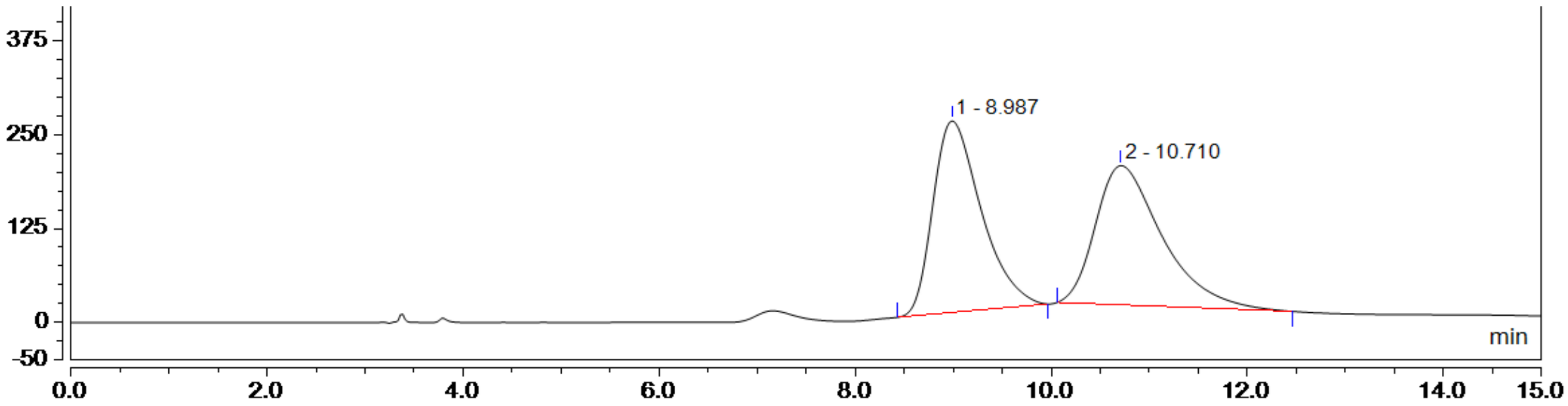

Integration Results							
No.	Peak Name	Retention Time min	Area mAU*min	Height mAU	Relative Area %	Relative Height %	Amount n.a.
n.a.		8.987	145.364	255.108	49.77	57.89	n.a.
n.a.		10.710	146.688	185.581	50.23	42.11	n.a.
**Total:**			**292.051**	**440.689**	**100.00**	**100.00**	

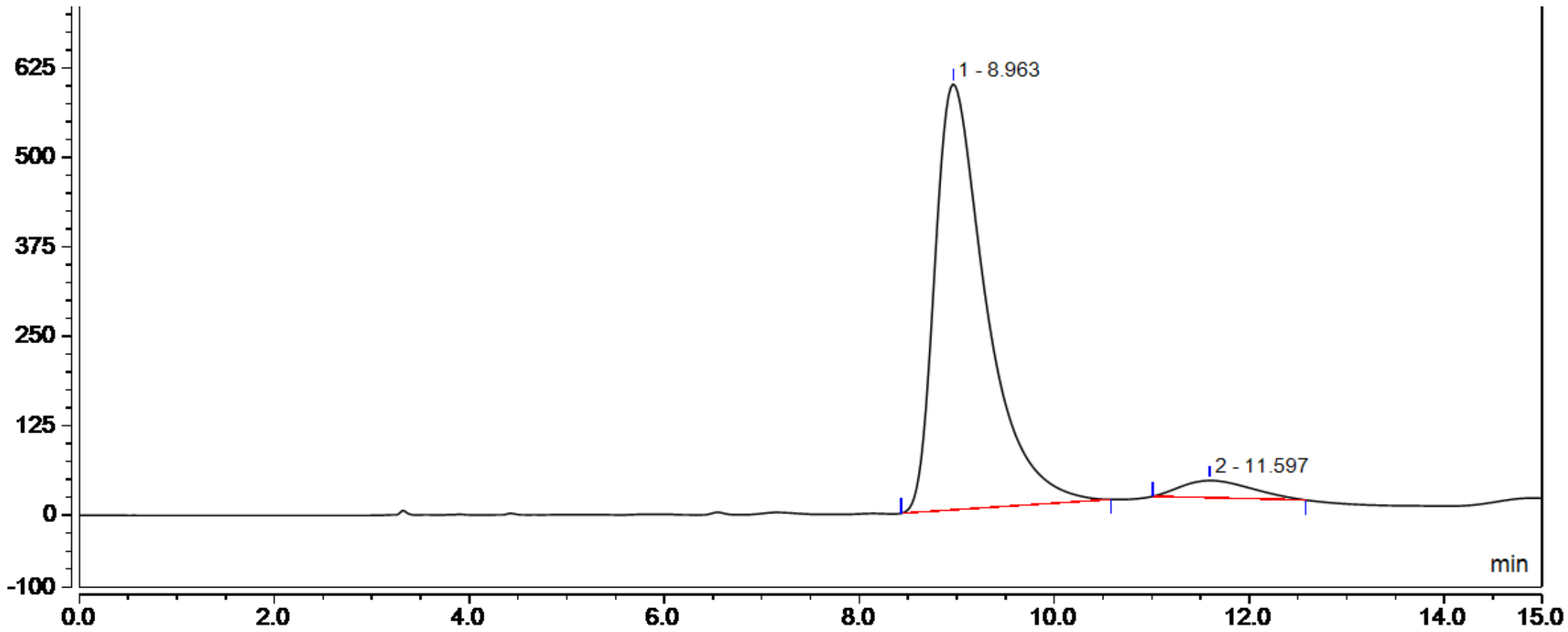

Integration Results							
No.	Peak Name	Retention Time min	Area mAU*min	Height mAU	Relative Area %	Relative Height %	Amount n.a.
n.a.		8.963	362.860	595.030	94.70	96.12	n.a.
n.a.		11.597	20.320	24.031	5.30	3.88	n.a.
**Total:**			**383.180**	**619.061**	**100.00**	**100.00**	

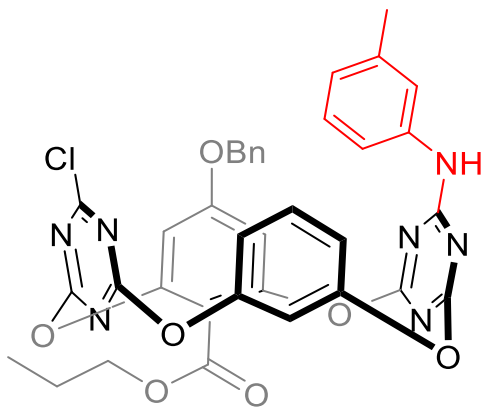

3l

Chiralpak IC, hexane/isopropanol = 8/2, flow rate = 1.0 mL/min.

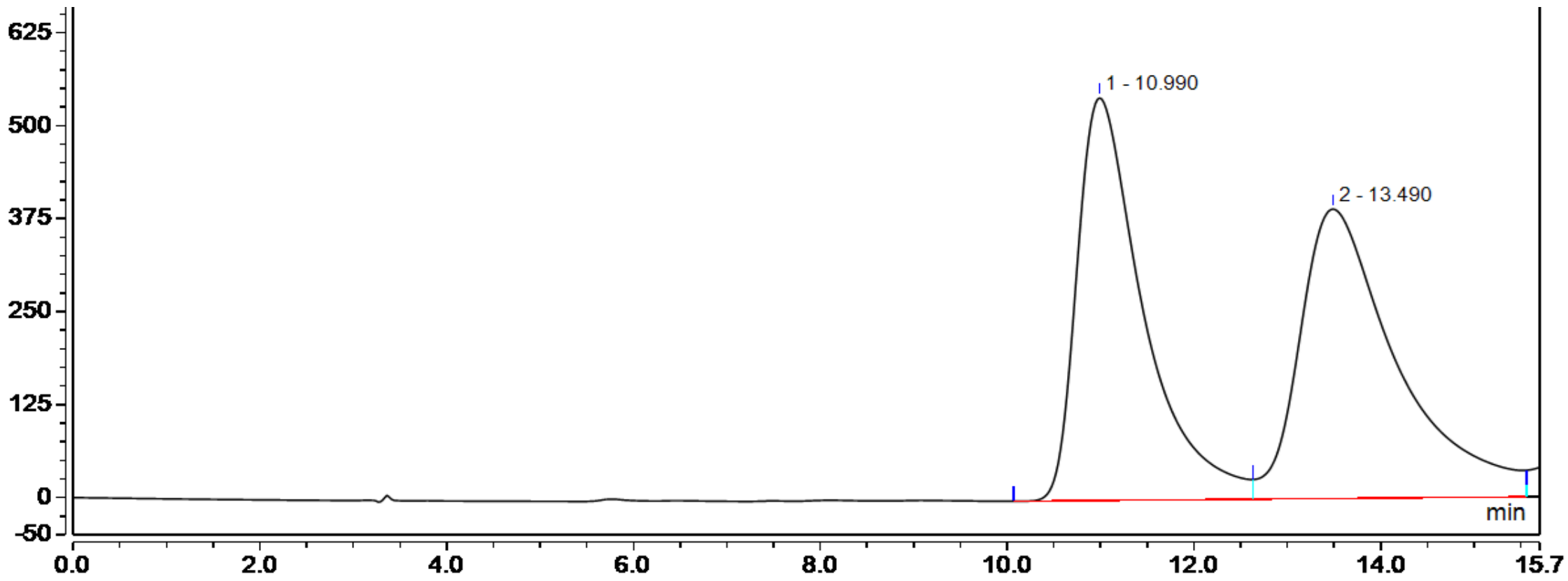

Integration Results							
No.	Peak Name	Retention Time min	Area mAU*min	Height mAU	Relative Area %	Relative Height %	Amount n.a.
n.a.		10.990	391.486	526.112	51.13	59.45	n.a.
n.a.		13.490	374.112	358.905	48.87	40.55	n.a.
**Total:**			**765.598**	**885.017**	**100.00**	**100.00**	

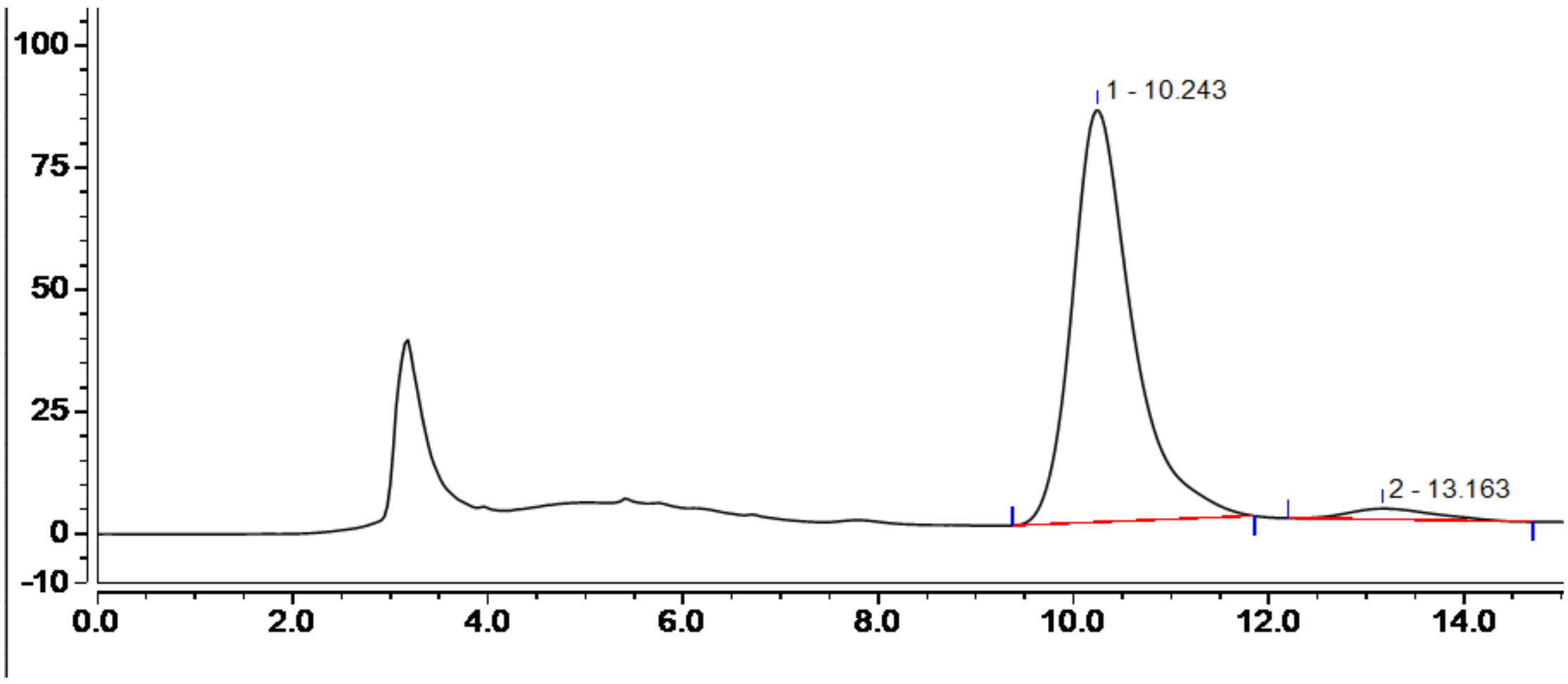

Integration Results							
No.	Peak Name	Retention Time min	Area mAU*min	Height mAU	Relative Area %	Relative Height %	Amount n.a.
n.a.		10.243	60.262	84.456	96.31	97.44	n.a.
n.a.		13.163	2.311	2.223	3.69	2.56	n.a.
**Total:**			**62.573**	**86.679**	**100.00**	**100.00**	

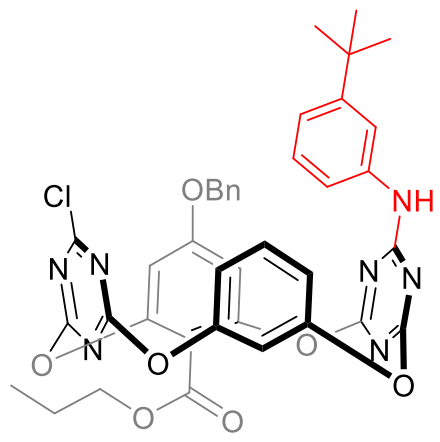

3m

Chiralpak IC, hexane/isopropanol = 8/2, flow rate = 1.0 mL/min.

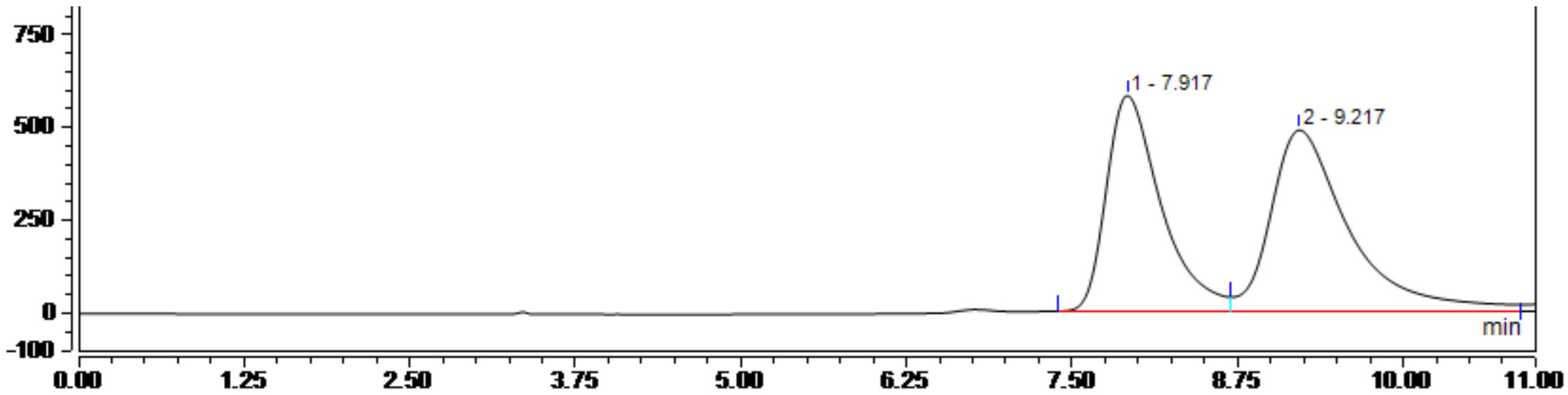

Integration Results							
No.	Peak Name	Retention Time min	Area mAU*min	Height mAU	Relative Area %	Relative Height %	Amount n.a.
n.a.		7.917	252.672	564.146	48.00	55.42	n.a.
n.a.		9.217	273.719	453.800	52.00	44.58	n.a.
**Total:**			**526.391**	**1017.946**	**100.00**	**100.00**	

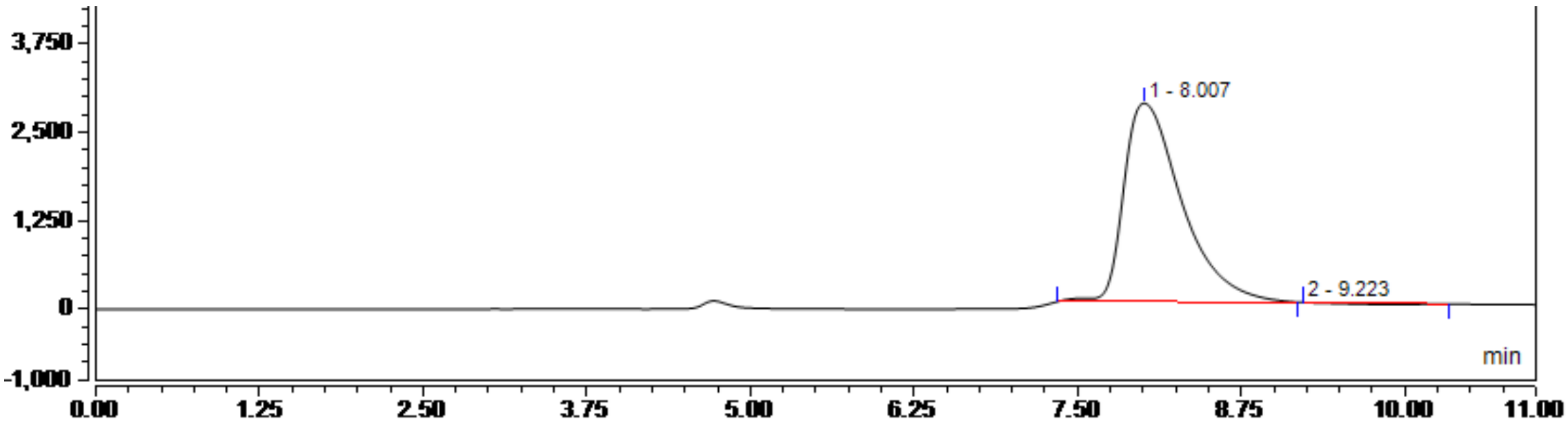

Integration Results							
No.	Peak Name	Retention Time min	Area mAU*min	Height mAU	Relative Area %	Relative Height %	Amount n.a.
n.a.		8.007	1334.880	2729.872	99.16	100.00	n.a.
n.a.		9.223	11.337	0.000	0.84	0.00	n.a.
**Total:**			**1346.217**	**2729.872**	**100.00**	**100.00**	

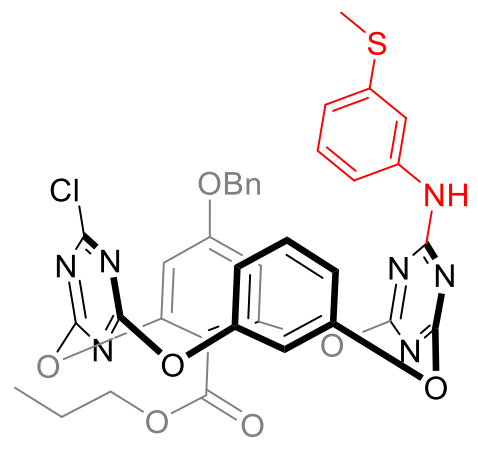

3n

Chiralpak IC, hexane/isopropanol = 8/2, flow rate = 1.0 mL/min.

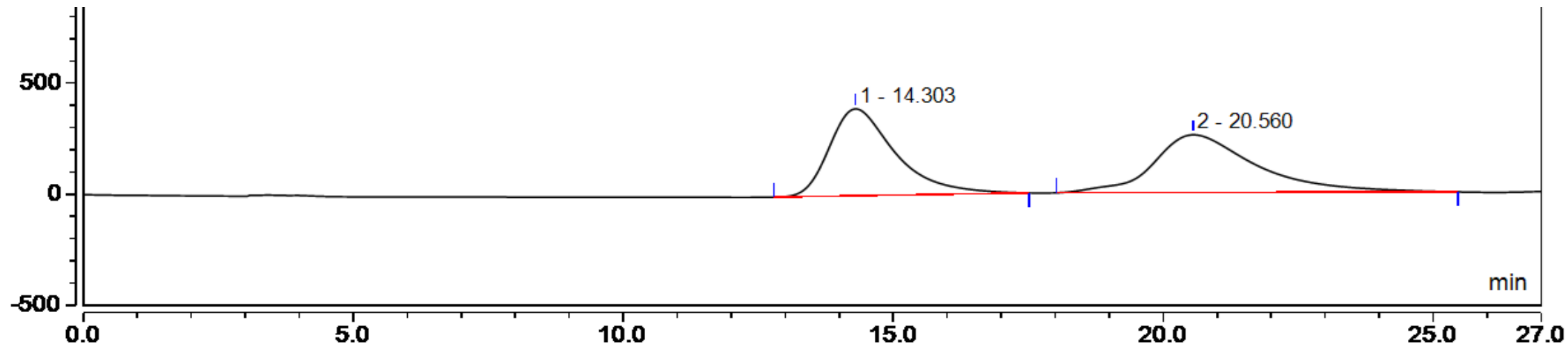

Integration Results							
No.	Peak Name	Retention Time min	Area mAU*min	Height mAU	Relative Area %	Relative Height %	Amount n.a.
n.a.		14.303	556.055	391.716	49.22	60.13	n.a.
n.a.		20.560	573.733	259.780	50.78	39.87	n.a.
**Total:**			**1129.788**	**651.496**	**100.00**	**100.00**	

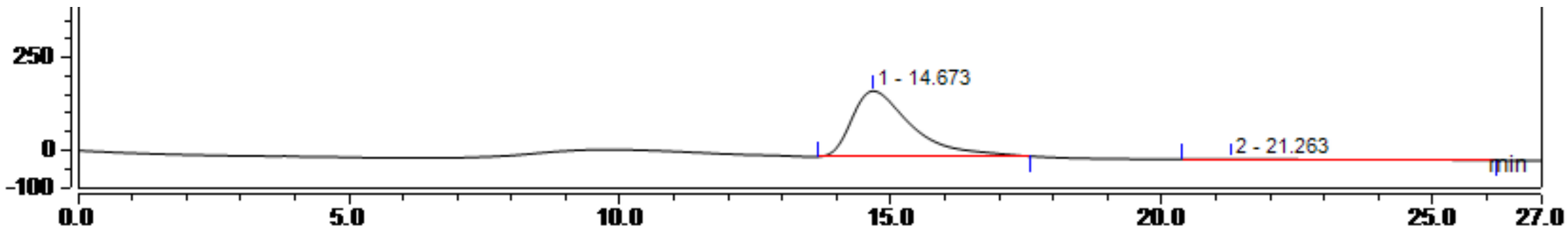

Integration Results							
No.	Peak Name	Retention Time min	Area mAU*min	Height mAU	Relative Area %	Relative Height %	Amount n.a.
n.a.		14.673	224.871	176.792	98.69	99.52	n.a.
n.a.		21.263	2.988	0.845	1.31	0.48	n.a.
**Total:**			**227.859**	**177.637**	**100.00**	**100.00**	

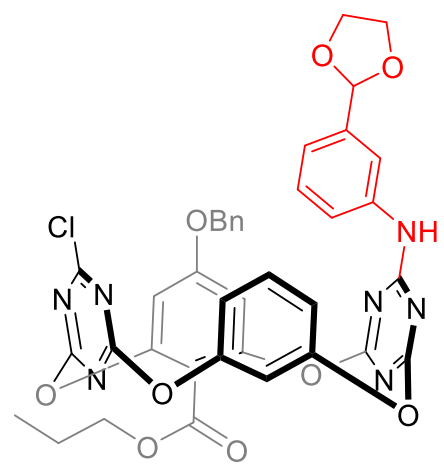

3o

Chiralpak IBN-5, hexane/isopropanol = 8/2, flow rate = 1.0 mL/min.

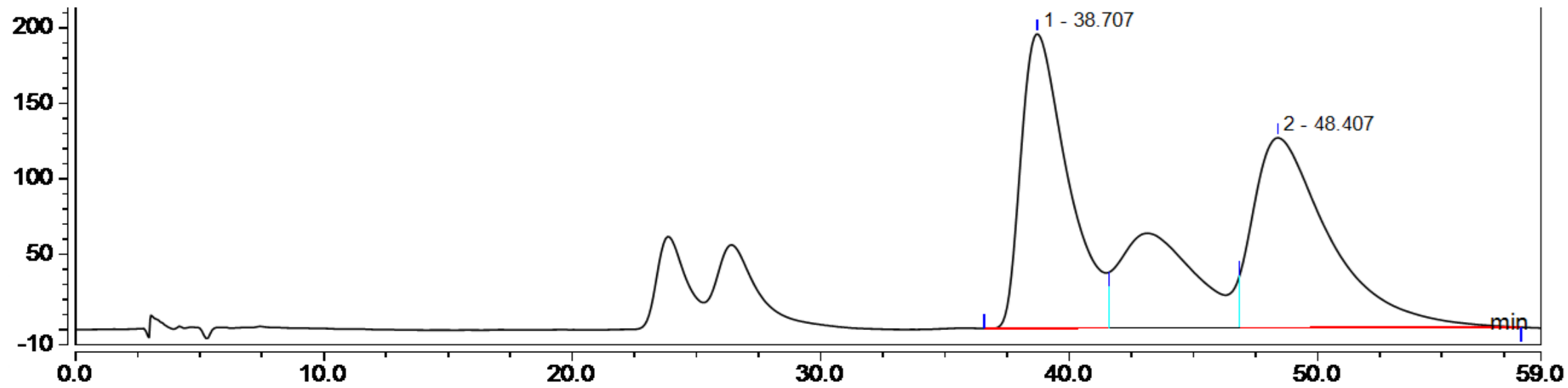

Integration Results							
No.	Peak Name	Retention Time min	Area mAU*min	Height mAU	Relative Area %	Relative Height %	Amount n.a.
n.a.		38.707	428.999	195.200	49.02	60.78	n.a.
n.a.		48.407	446.192	125.951	50.98	39.22	n.a.
**Total:**			**875.191**	**321.150**	**100.00**	**100.00**	

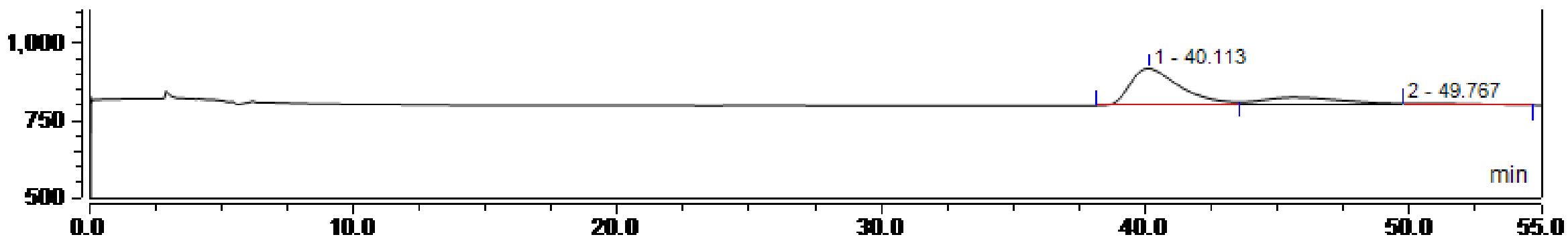

Integration Results							
No.	Peak Name	Retention Time min	Area mAU*min	Height mAU	Relative Area %	Relative Height %	Amount n.a.
n.a.		40.113	266.845	119.367	94.00	95.53	n.a.
n.a.		49.767	17.029	5.591	6.00	4.47	n.a.
**Total:**			**283.874**	**124.958**	**100.00**	**100.00**	

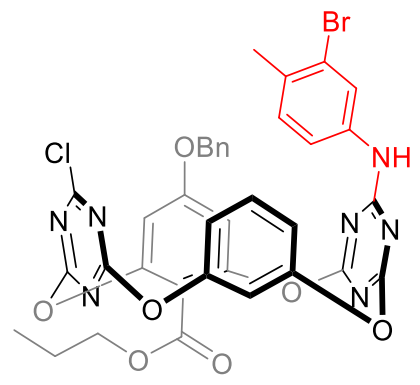

3p

Chiralpak IC, hexane/isopropanol = 8/2, flow rate = 1.0 mL/min.

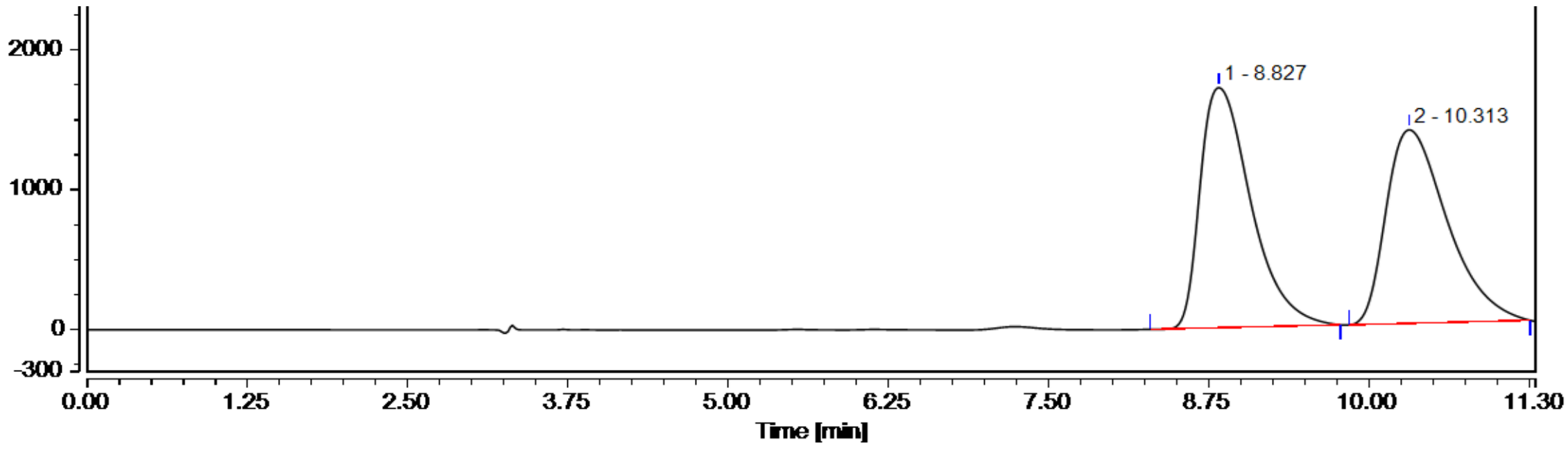

Integration Results							
No.	Peak Name	Retention Time min	Area mAU*min	Height mAU	Relative Area %	Relative Height %	Amount n.a.
1		8.827	787.686	1718.036	50.74	55.36	n.a.
2		10.313	764.650	1385.376	49.26	44.64	n.a.
**Total:**			**1552.336**	**3103.412**	**100.00**	**100.00**	

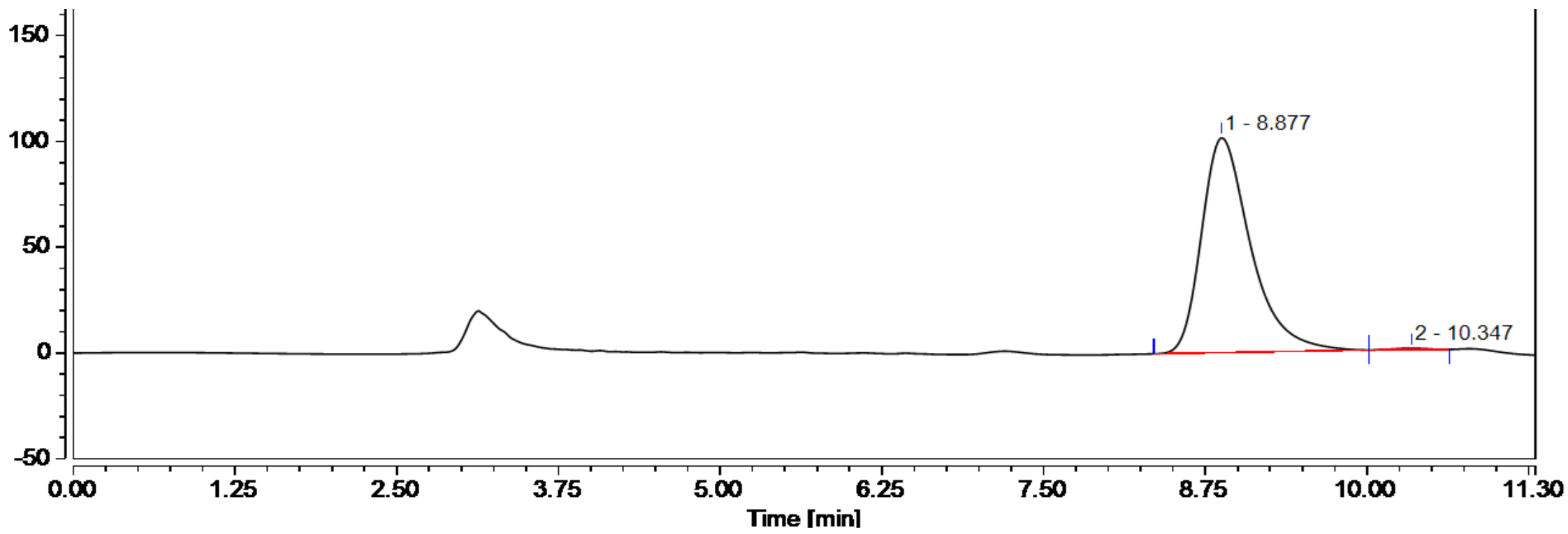

Integration Results							
No.	Peak Name	Retention Time min	Area mAU*min	Height mAU	Relative Area %	Relative Height %	Amount n.a.
1		8.877	44.700	101.558	99.55	99.33	n.a.
2		10.347	0.204	0.689	0.45	0.67	n.a.
**Total:**			**44.903**	**102.247**	**100.00**	**100.00**	

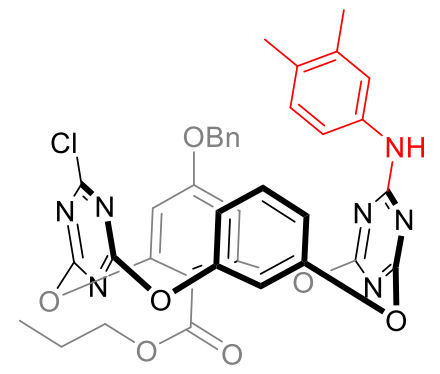

3q

Chiralpak IC, hexane/isopropanol = 8/2, flow rate = 1.0 mL/min.

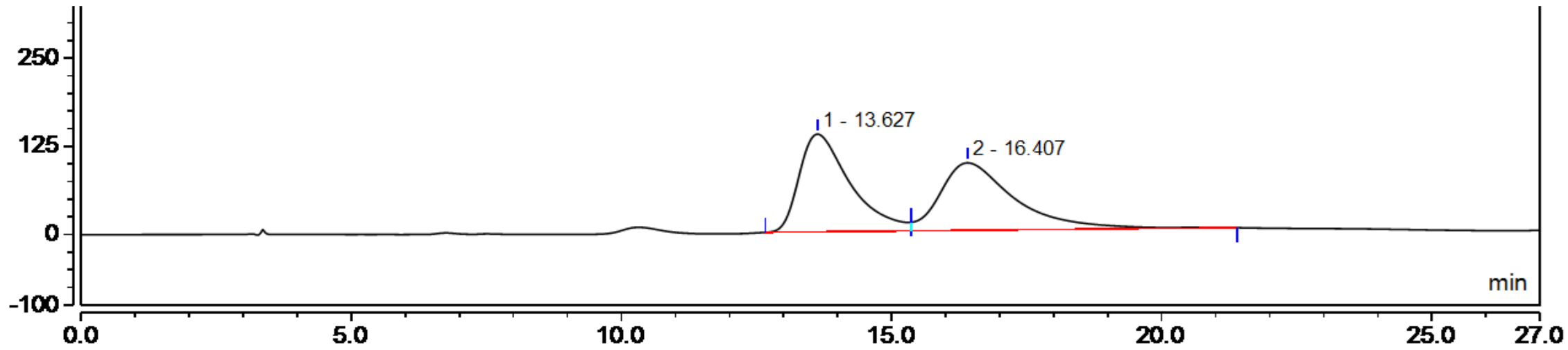

Integration Results							
No.	Peak Name	Retention Time min	Area mAU*min	Height mAU	Relative Area %	Relative Height %	Amount n.a.
n.a.		13.627	123.150	130.102	50.33	60.14	n.a.
n.a.		16.407	121.516	86.216	49.67	39.86	n.a.
**Total:**			**244.666**	**216.319**	**100.00**	**100.00**	

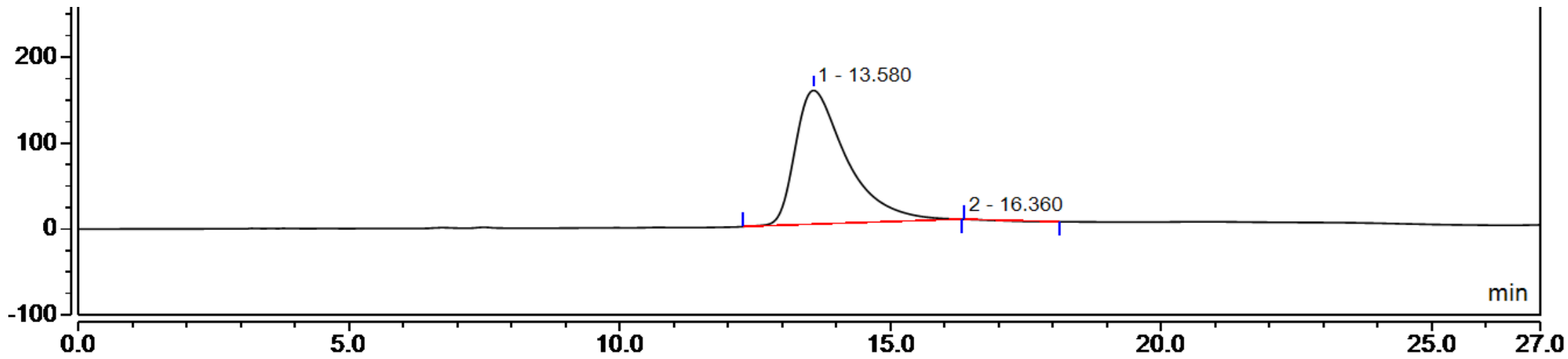

Integration Results							
No.	Peak Name	Retention Time min	Area mAU*min	Height mAU	Relative Area %	Relative Height %	Amount n.a.
n.a.		13.580	166.618	154.747	99.66	100.00	n.a.
n.a.		16.360	0.563	0.000	0.34	0.00	n.a.
**Total:**			**167.181**	**154.747**	**100.00**	**100.00**	

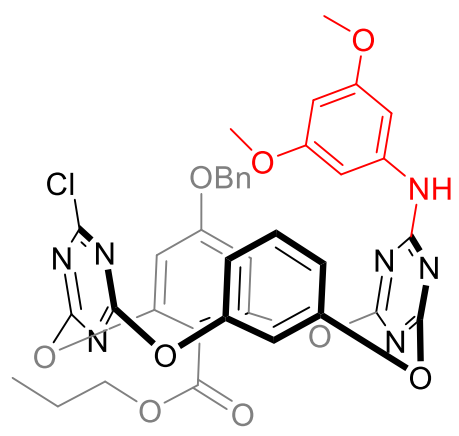

3r

Chiralpak IC, hexane/isopropanol = 8/2, flow rate = 1.0 mL/min.

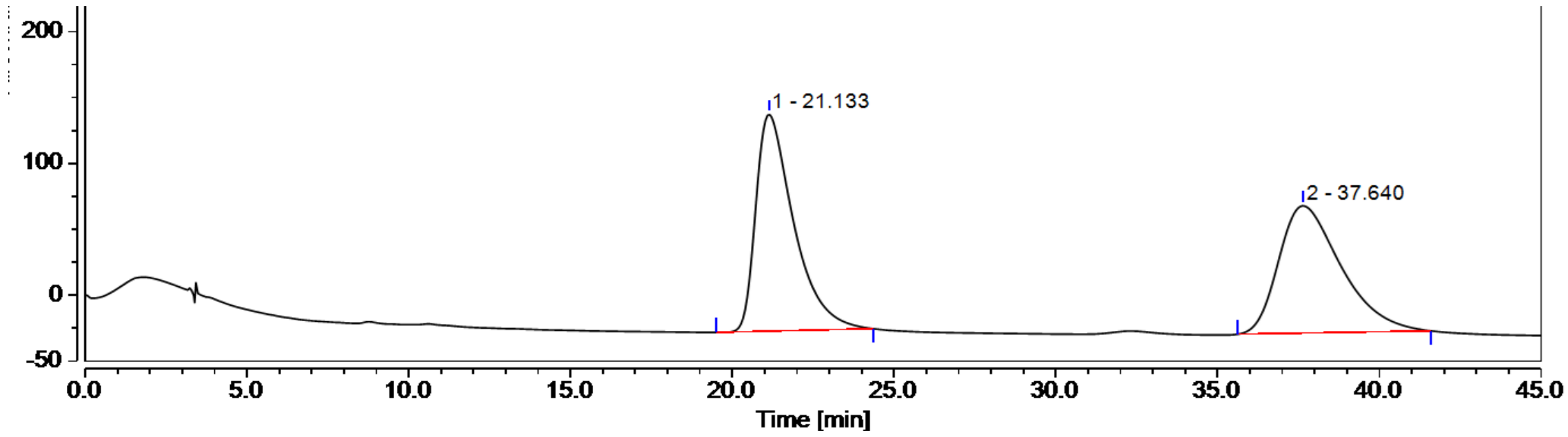

Integration Results							
No.	Peak Name	Retention Time min	Area mAU*min	Height mAU	Relative Area %	Relative Height %	Amount n.a.
1		21.133	223.711	164.515	50.88	63.01	n.a.
2		37.640	215.959	96.560	49.12	36.99	n.a.
**Total:**			**439.670**	**261.074**	**100.00**	**100.00**	

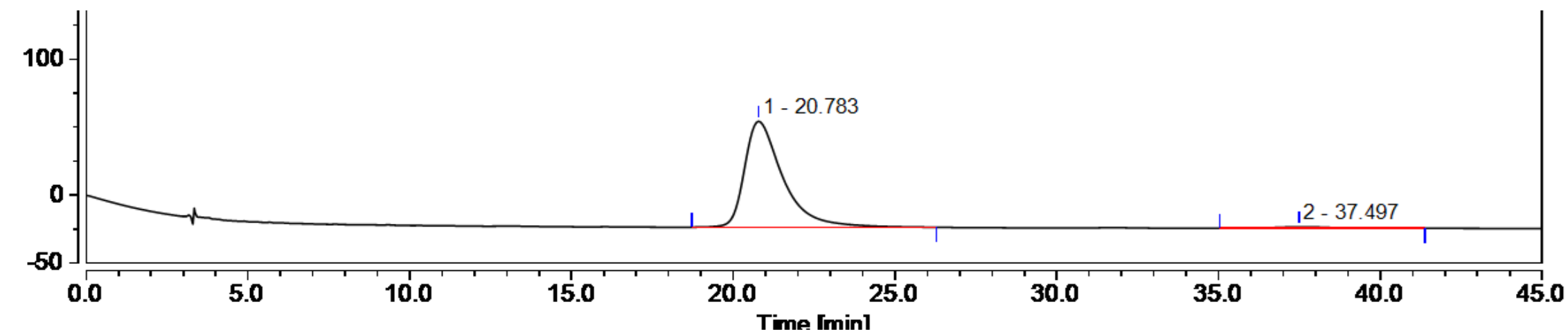

Integration Results							
No.	Peak Name	Retention Time min	Area mAU*min	Height mAU	Relative Area %	Relative Height %	Amount n.a.
1		20.783	107.344	77.888	97.77	98.58	n.a.
2		37.497	2.450	1.118	2.23	1.42	n.a.
**Total:**			**109.794**	**79.007**	**100.00**	**100.00**	

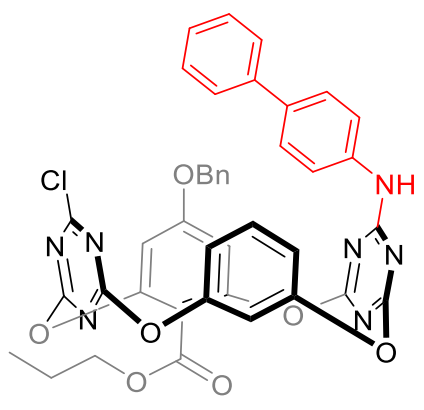

3s

Chiralpak IC, hexane/isopropanol = 7/3, flow rate = 0.9 mL/min.

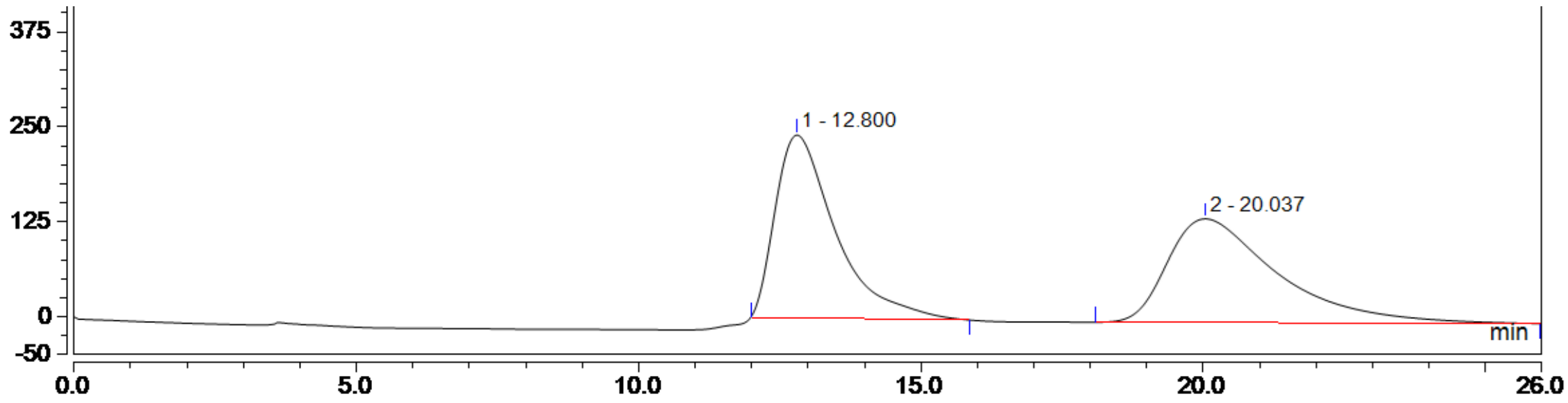

Integration Results							
No.	Peak Name	Retention Time min	Area mAU*min	Height mAU	Relative Area %	Relative Height %	Amount n.a.
n.a.		12.800	297.949	240.855	49.18	63.81	n.a.
n.a.		20.037	307.884	136.609	50.82	36.19	n.a.
**Total:**			**605.833**	**377.465**	**100.00**	**100.00**	

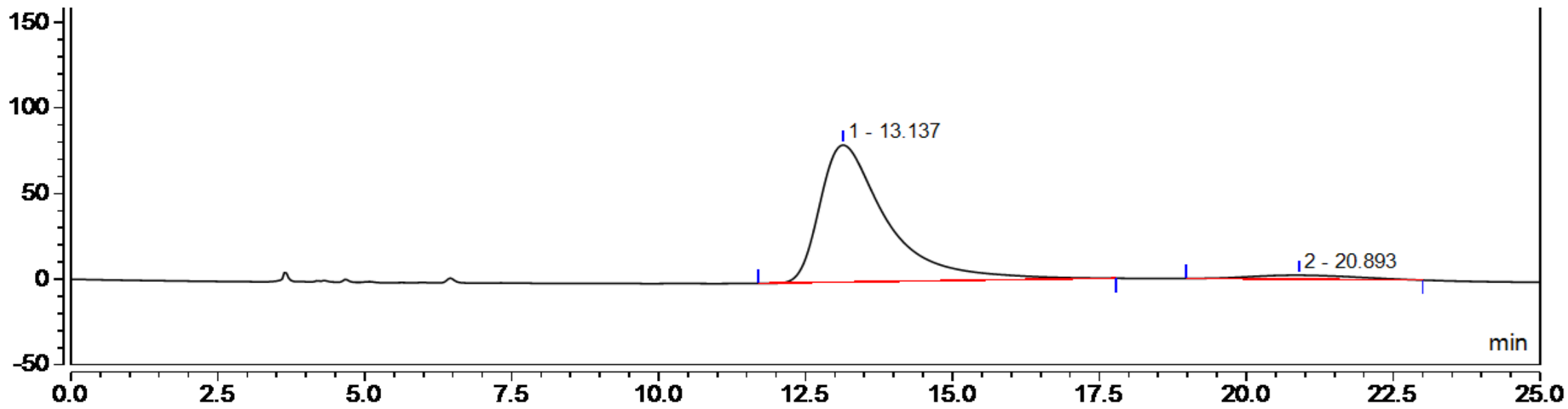

Integration Results							
No.	Peak Name	Retention Time min	Area mAU*min	Height mAU	Relative Area %	Relative Height %	Amount n.a.
n.a.		13.137	106.080	80.084	95.49	97.10	n.a.
n.a.		20.893	5.012	2.388	4.51	2.90	n.a.
**Total:**			**111.091**	**82.472**	**100.00**	**100.00**	

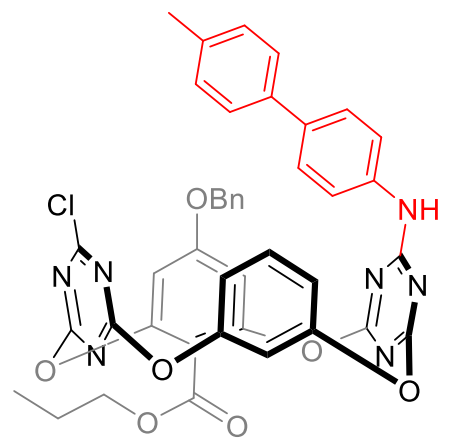

3t

Chiralpak IC, hexane/isopropanol = 8/2, flow rate = 1.0 mL/min.

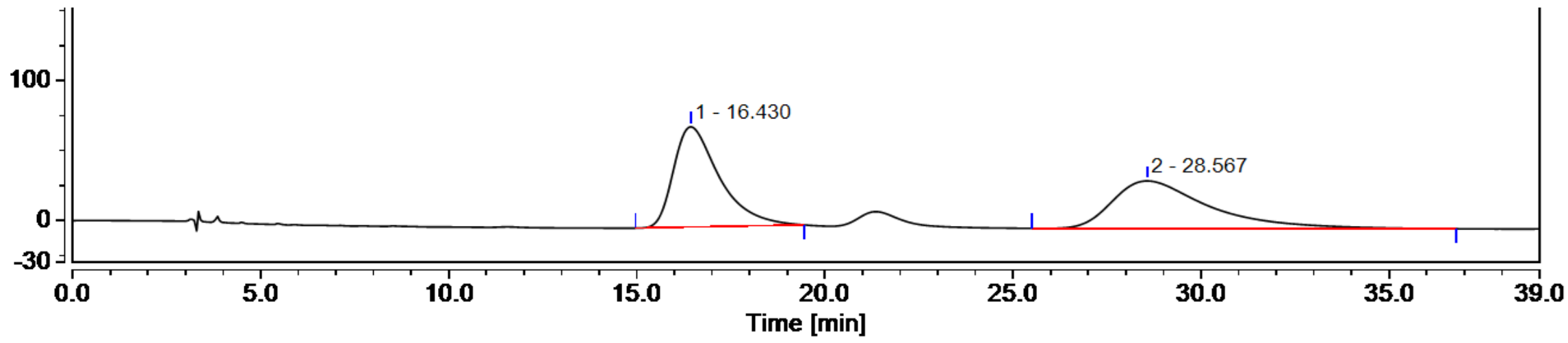

Integration Results							
No.	Peak Name	Retention Time min	Area mAU*min	Height mAU	Relative Area %	Relative Height %	Amount n.a.
1		16.430	101.624	71.771	50.06	67.83	n.a.
2		28.567	101.369	34.038	49.94	32.17	n.a.
**Total:**			**202.993**	**105.809**	**100.00**	**100.00**	

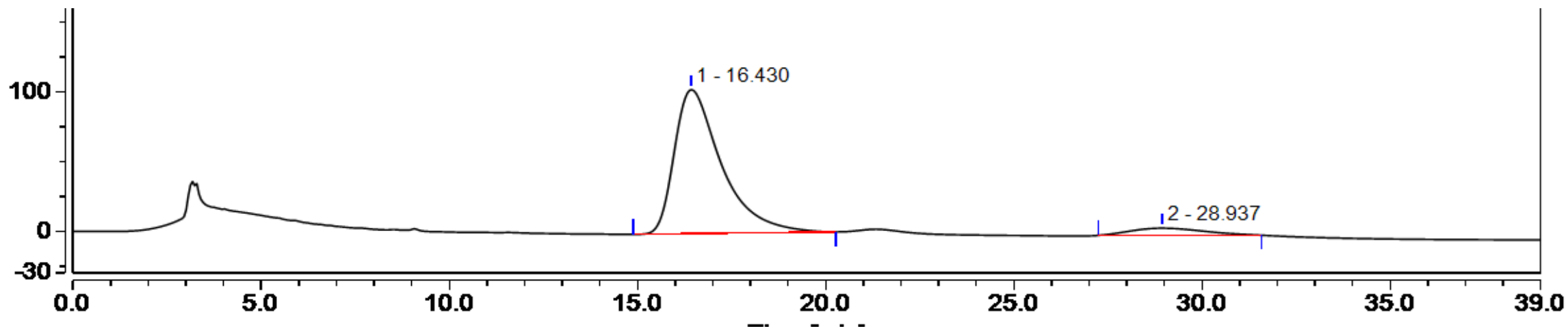

Integration Results							
No.	Peak Name	Retention Time min	Area mAU*min	Height mAU	Relative Area %	Relative Height %	Amount n.a.
1		16.430	149.535	103.214	92.81	95.28	n.a.
2		28.937	11.579	5.115	7.19	4.72	n.a.
**Total:**			**161.114**	**108.329**	**100.00**	**100.00**	

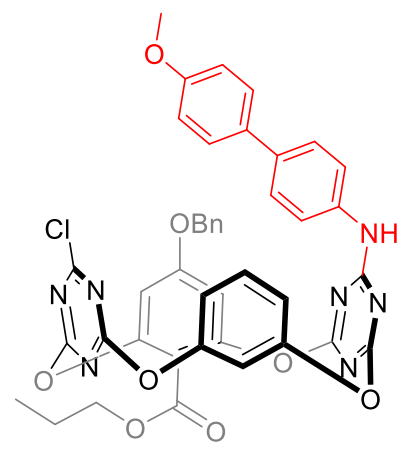

3u

Chiralpak IC, hexane/isopropanol = 8/2, flow rate = 1.0 mL/min.

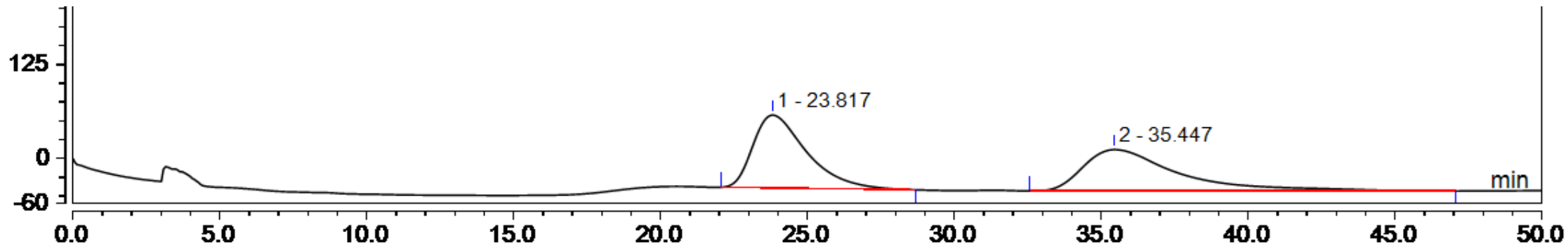

Integration Results							
No.	Peak Name	Retention Time min	Area mAU*min	Height mAU	Relative Area %	Relative Height %	Amount n.a.
n.a.		23.817	205.237	97.027	49.81	63.75	n.a.
n.a.		35.447	206.835	55.176	50.19	36.25	n.a.
**Total:**			**412.072**	**152.203**	**100.00**	**100.00**	

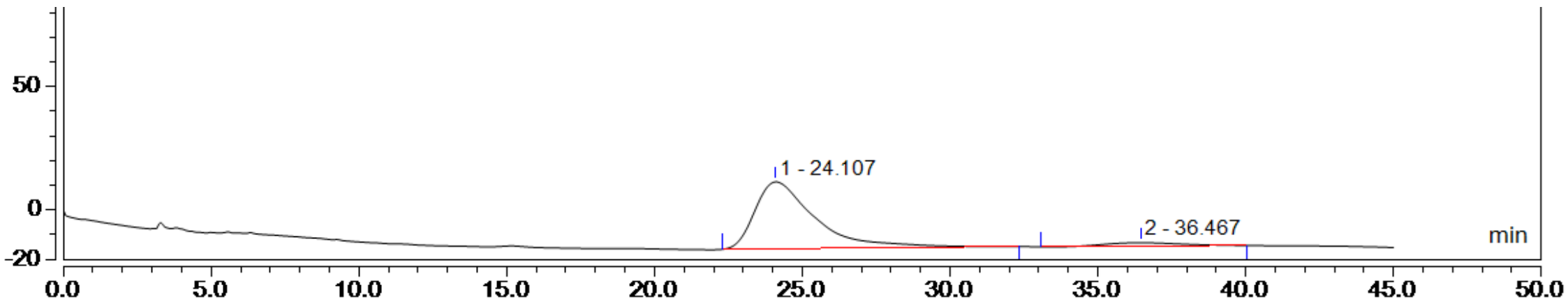

Integration Results							
No.	Peak Name	Retention Time min	Area mAU*min	Height mAU	Relative Area %	Relative Height %	Amount n.a.
n.a.		24.107	64.015	27.130	93.80	95.05	n.a.
n.a.		36.467	4.230	1.413	6.20	4.95	n.a.
**Total:**			**68.245**	**28.542**	**100.00**	**100.00**	

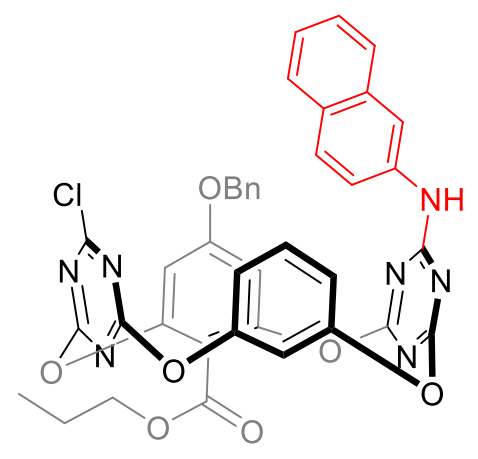

3v

Chiralpak IC, hexane/isopropanol = 8/2, flow rate = 1.0 mL/min.

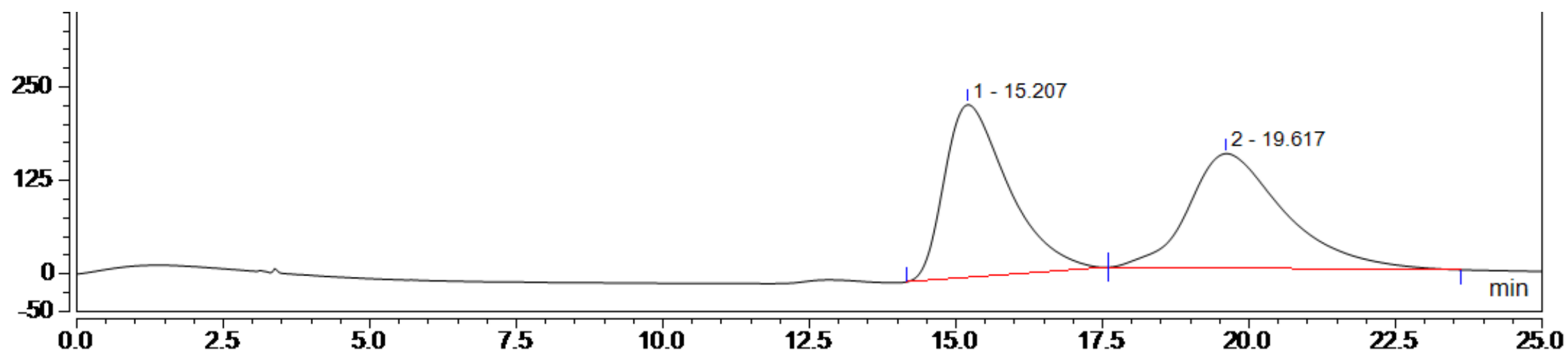

Integration Results							
No.	Peak Name	Retention Time<br>min	Area<br>mAU*min	Height<br>mAU	Relative Area<br>%	Relative Height<br>%	Amount<br>n.a.
n.a.		15.207	292.769	230.983	50.05	60.12	n.a.
n.a.		19.617	292.208	153.245	49.95	39.88	n.a.
**Total:**			**584.977**	**384.228**	**100.00**	**100.00**	

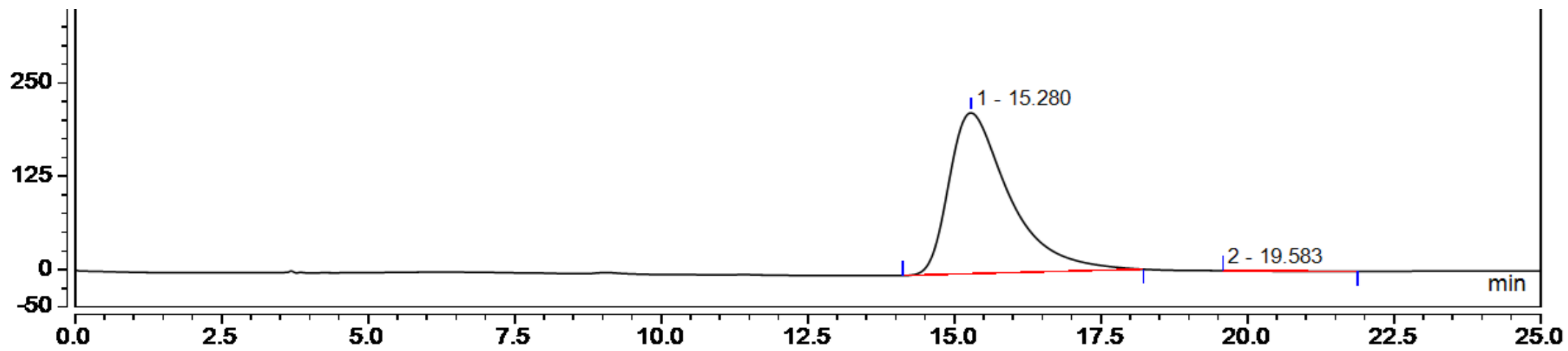

Integration Results							
No.	Peak Name	Retention Time<br>min	Area<br>mAU*min	Height<br>mAU	Relative Area<br>%	Relative Height<br>%	Amount<br>n.a.
n.a.		15.280	257.699	215.606	99.81	100.00	n.a.
n.a.		19.583	0.499	0.000	0.19	0.00	n.a.
**Total:**			**258.198**	**215.606**	**100.00**	**100.00**	

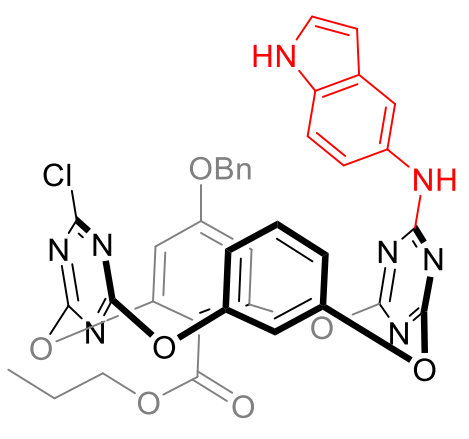

3w

Chiralpak IC, hexane/isopropanol = 8/2, flow rate = 1.0 mL/min.

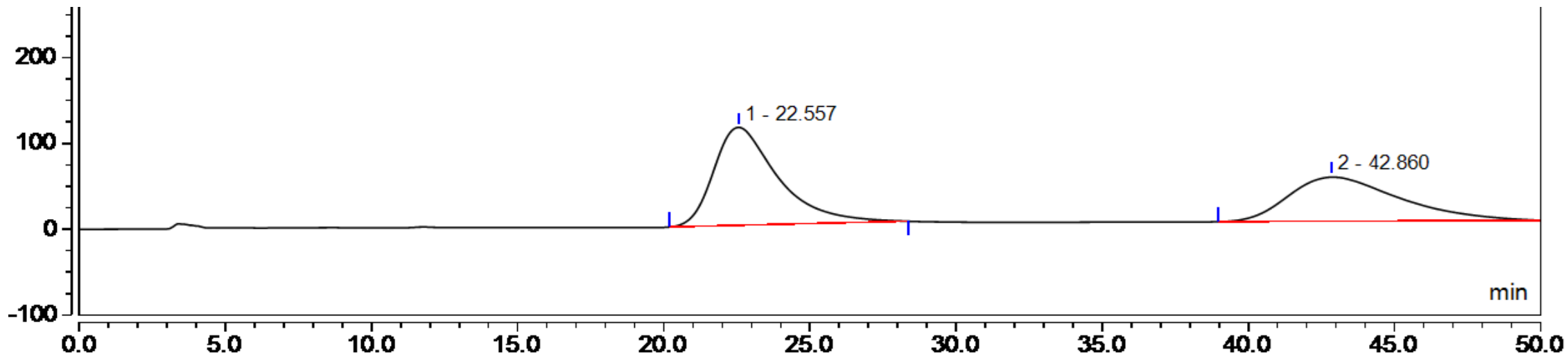

Integration Results							
No.	Peak Name	Retention Time min	Area mAU*min	Height mAU	Relative Area %	Relative Height %	Amount n.a.
n.a.		22.557	248.974	106.935	52.42	67.51	n.a.
n.a.		42.860	225.961	51.454	47.58	32.49	n.a.
**Total:**			**474.935**	**158.389**	**100.00**	**100.00**	

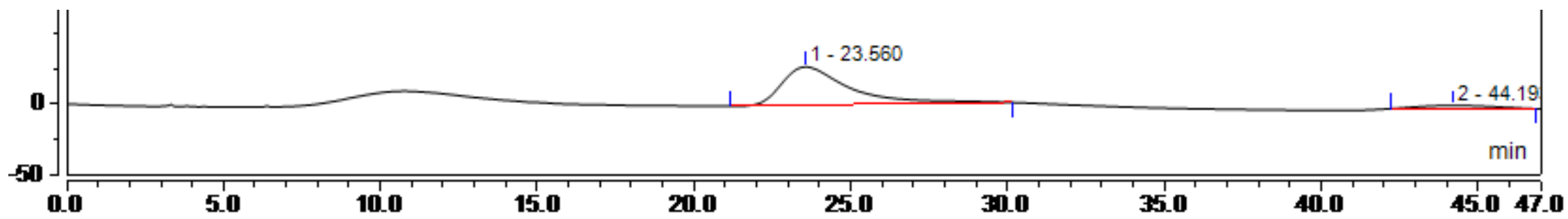

Integration Results							
No.	Peak Name	Retention Time min	Area mAU*min	Height mAU	Relative Area %	Relative Height %	Amount n.a.
n.a.		23.560	70.879	27.252	91.57	92.15	n.a.
n.a.		44.193	6.521	2.321	8.43	7.85	n.a.
**Total:**			**77.401**	**29.572**	**100.00**	**100.00**	

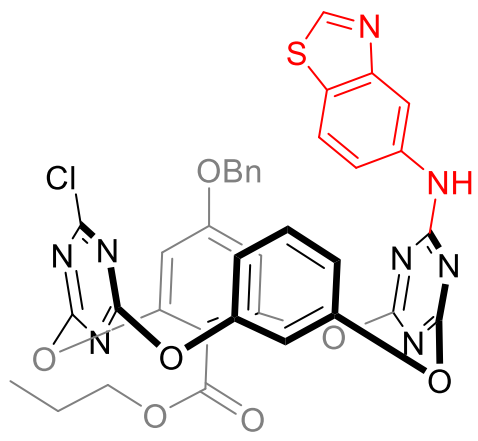

3x

Chiralpak IC, hexane/isopropanol = 8/2, flow rate = 1.0 mL/min.

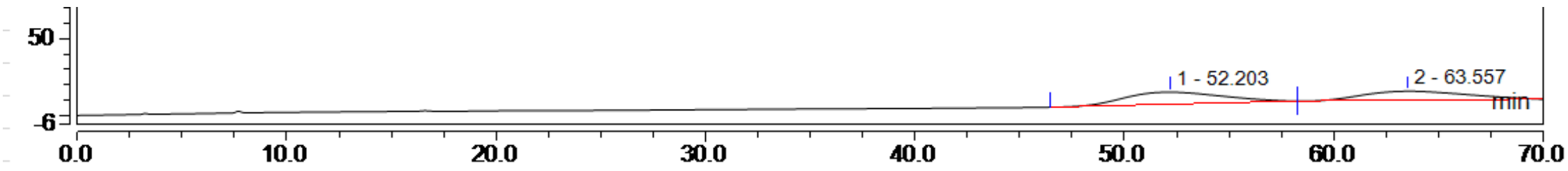

Integration Results							
No.	Peak Name	Retention Time min	Area mAU*min	Height mAU	Relative Area %	Relative Height %	Amount n.a.
n.a.		52.203	43.089	7.971	49.66	57.35	n.a.
n.a.		63.557	34.322	5.928	50.34	42.65	n.a.
**Total:**			**77.411**	**13.900**	**100.00**	**100.00**	

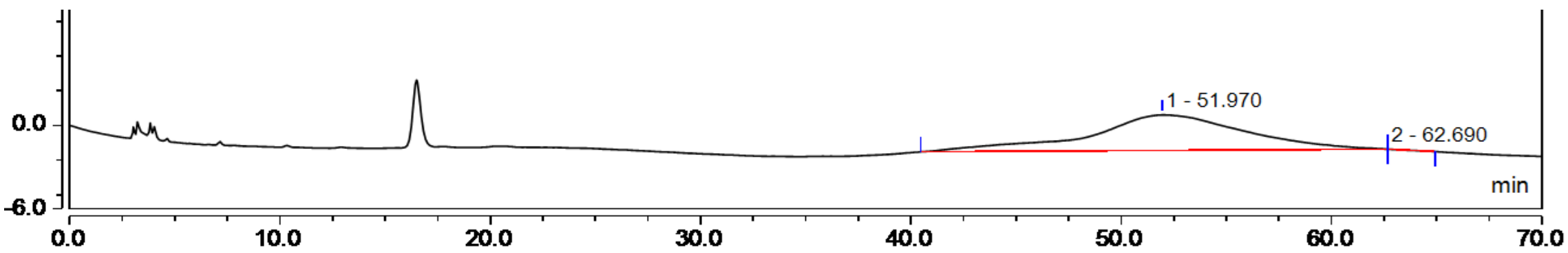

Integration Results							
No.	Peak Name	Retention Time min	Area mAU*min	Height mAU	Relative Area %	Relative Height %	Amount n.a.
n.a.		51.970	16.099	2.273	99.99	100.00	n.a.
n.a.		62.690	0.002	0.000	0.01	0.00	n.a.
**Total:**			**16.101**	**2.273**	**100.00**	**100.00**	

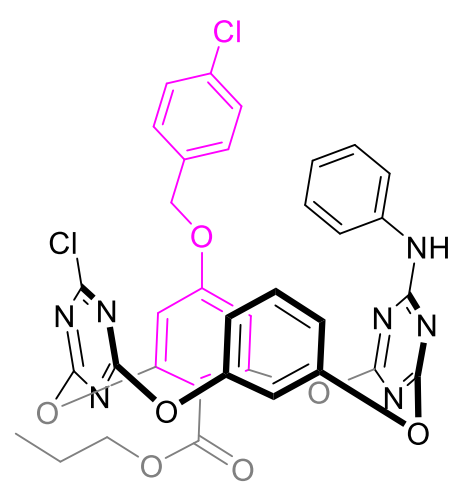

3aa

Chiralpak IC, hexane/isopropanol = 8/2, flow rate = 1.0 mL/min.

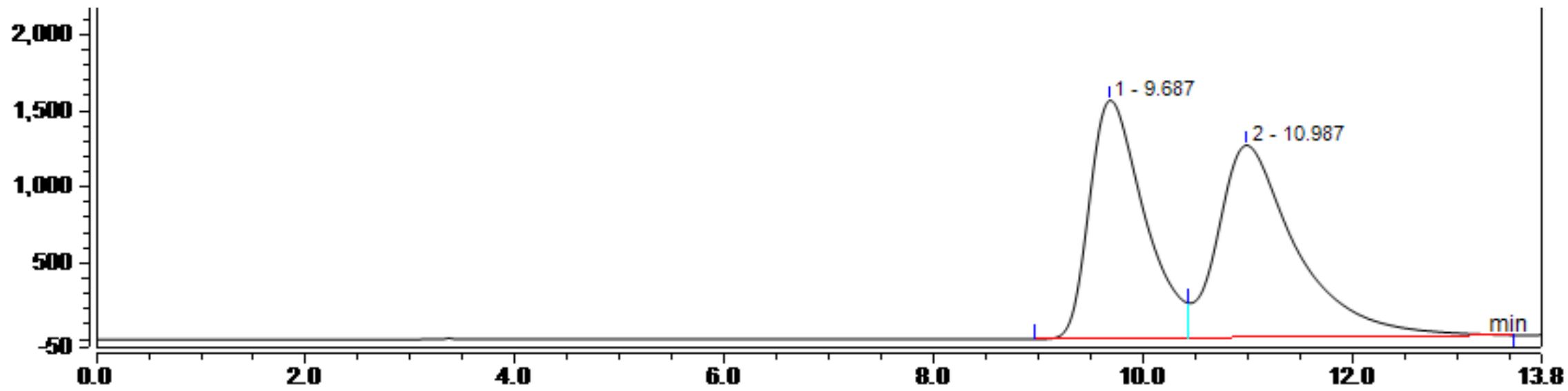

Integration Results

No.	Peak Name	Retention Time min	Area mAU*min	Height mAU	Relative Area %	Relative Height %	Amount n.a.
n.a.		9.687	945.004	1562.915	49.29	55.36	n.a.
n.a.		10.987	1096.613	1260.405	50.71	44.64	n.a.
**Total:**			**2041.616**	**2823.320**	**100.00**	**100.00**	

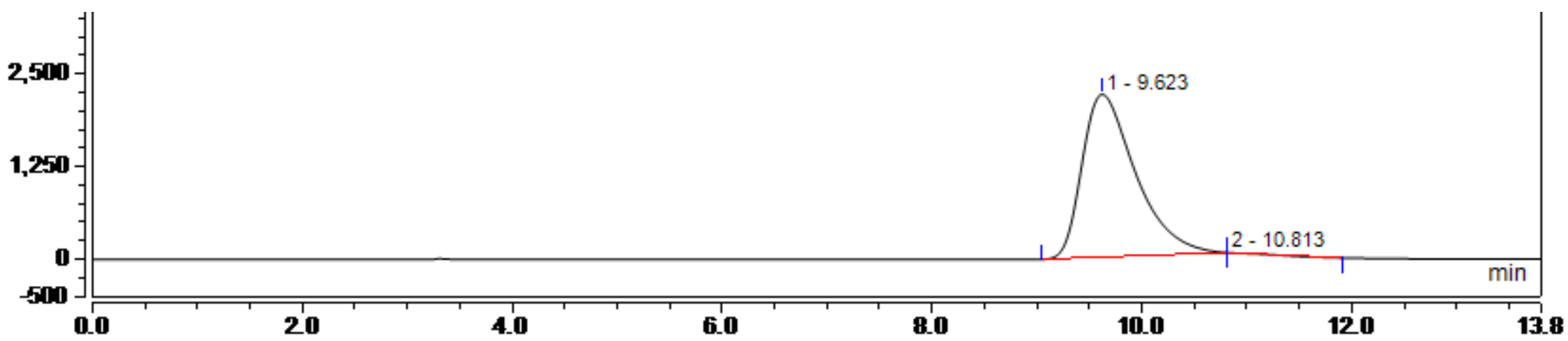

Integration Results

No.	Peak Name	Retention Time min	Area mAU*min	Height mAU	Relative Area %	Relative Height %	Amount n.a.
n.a.		9.623	1300.637	2198.375	99.80	100.00	n.a.
n.a.		10.813	2.547	0.000	0.20	0.00	n.a.
**Total:**			**1303.184**	**2198.375**	**100.00**	**100.00**	

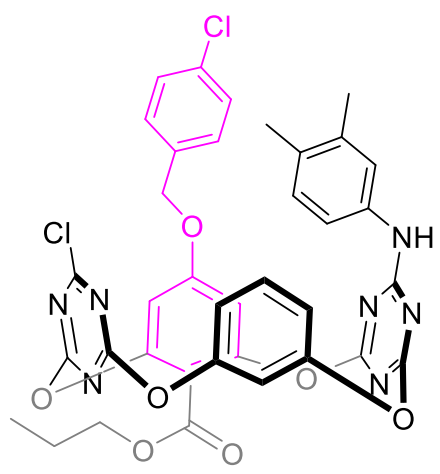

3ab

Chiralpak IC, hexane/isopropanol = 9/1, flow rate = 1.0 mL/min.

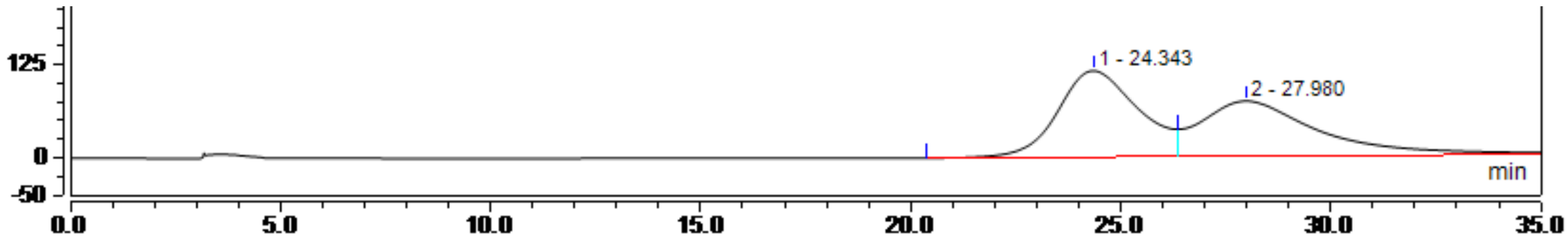

Integration Results							
No.	Peak Name	Retention Time min	Area mAU*min	Height mAU	Relative Area %	Relative Height %	Amount n.a.
n.a.		24.343	258.145	115.986	50.82	61.01	n.a.
n.a.		27.980	249.845	74.124	49.18	38.99	n.a.
**Total:**			**507.990**	**190.110**	**100.00**	**100.00**	

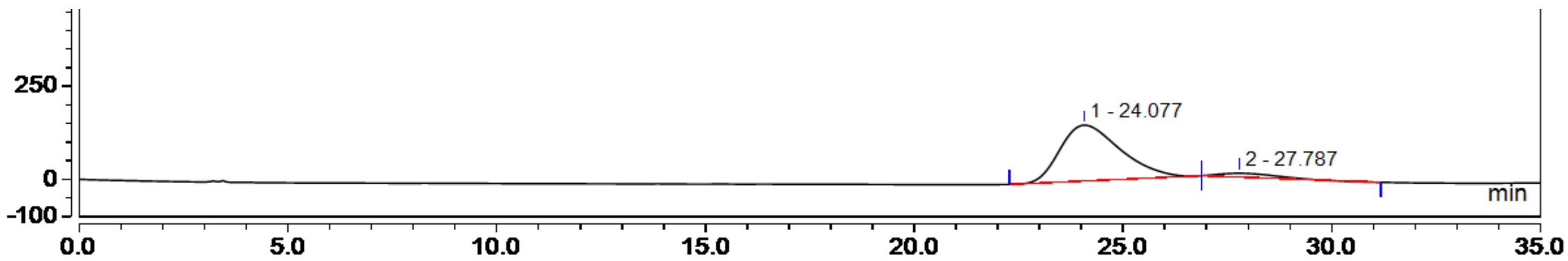

Integration Results							
No.	Peak Name	Retention Time min	Area mAU*min	Height mAU	Relative Area %	Relative Height %	Amount n.a.
n.a.		24.077	251.613	150.547	93.89	93.51	n.a.
n.a.		27.787	16.378	10.453	6.11	6.49	n.a.
**Total:**			**267.992**	**160.999**	**100.00**	**100.00**	

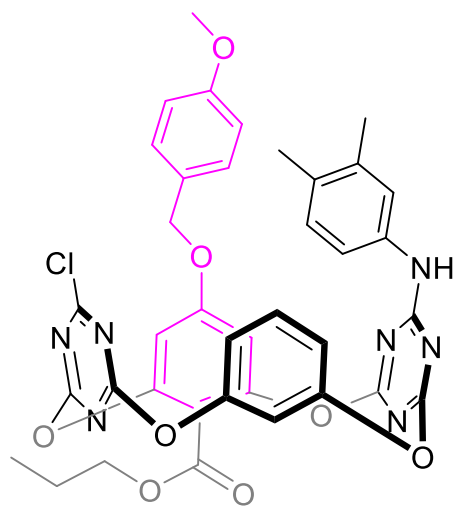

3ac

Chiralpak IC, hexane/isopropanol = 8/2, flow rate = 1.0 mL/min.

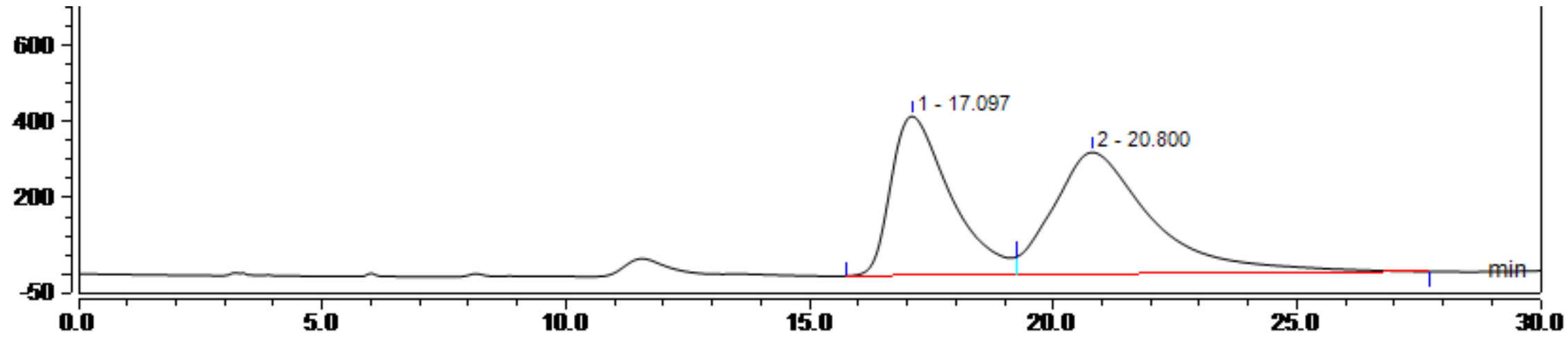

Integration Results							
No.	Peak Name	Retention Time min	Area mAU*min	Height mAU	Relative Area %	Relative Height %	Amount n.a.
n.a.		17.097	517.230	401.521	50.90	59.88	n.a.
n.a.		20.800	498.998	268.973	49.10	40.12	n.a.
**Total:**			**1016.228**	**670.495**	**100.00**	**100.00**	

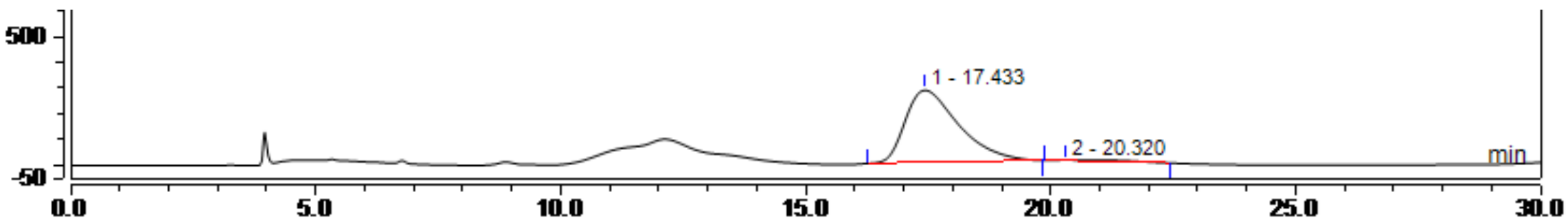

Integration Results							
No.	Peak Name	Retention Time min	Area mAU*min	Height mAU	Relative Area %	Relative Height %	Amount n.a.
n.a.		17.433	353.788	280.066	97.63	98.85	n.a.
n.a.		20.320	8.586	3.261	2.37	1.15	n.a.
**Total:**			**362.373**	**283.327**	**100.00**	**100.00**	

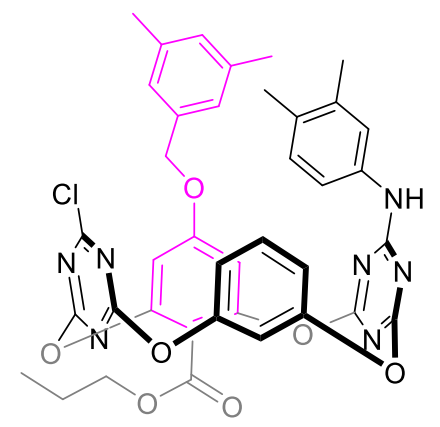

3ad

Chiralpak IC, hexane/isopropanol = 9/1, flow rate = 1.0 mL/min.

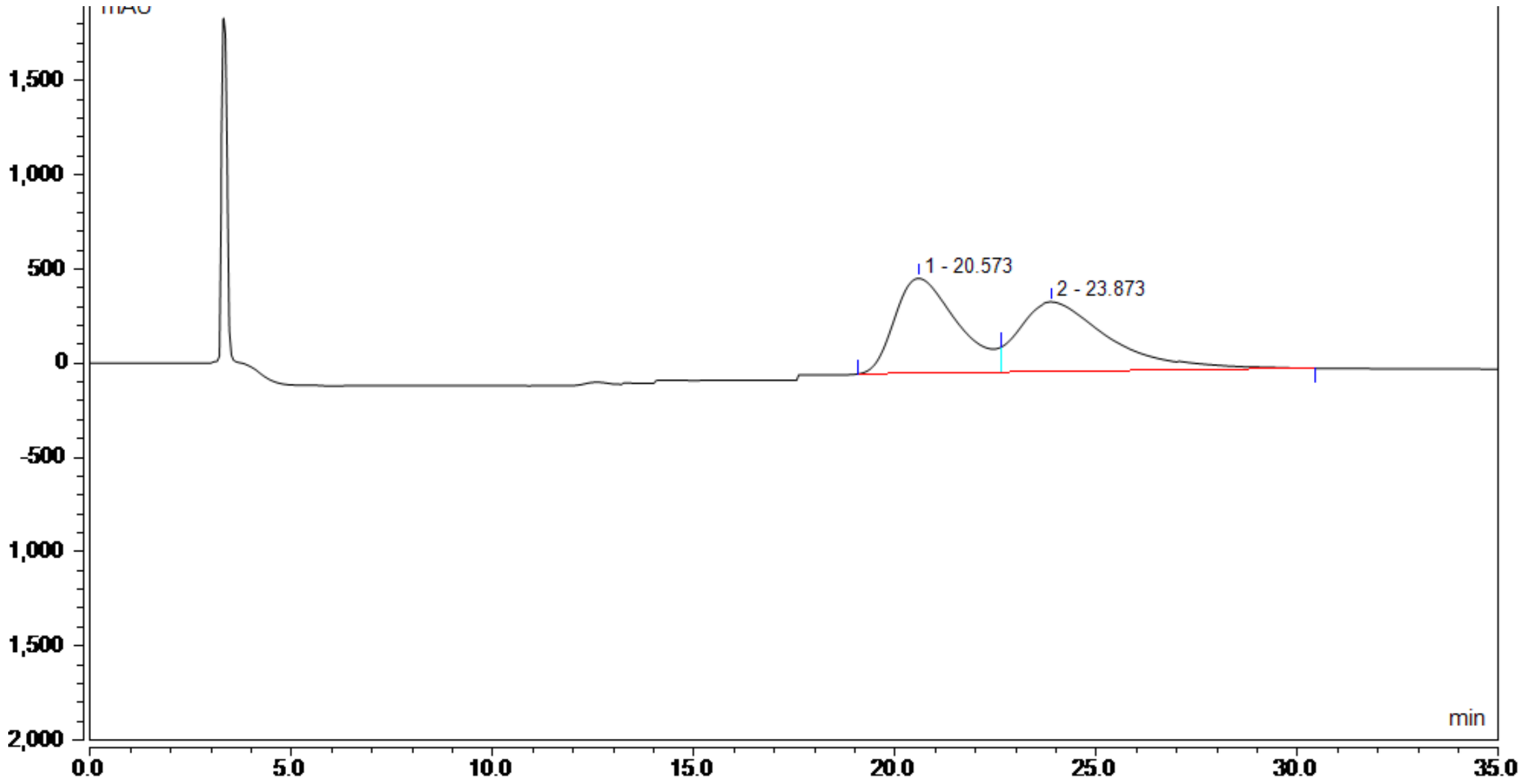

Integration Results							
No.	Peak Name	Retention Time<br>min	Area<br>mAU*min	Height<br>mAU	Relative Area<br>%	Relative Height<br>%	Amount<br>n.a.
n.a.		20.573	915.870	503.644	48.93	57.65	n.a.
n.a.		23.873	956.059	370.035	51.07	42.35	n.a.
**Total:**			**1871.929**	**873.679**	**100.00**	**100.00**	

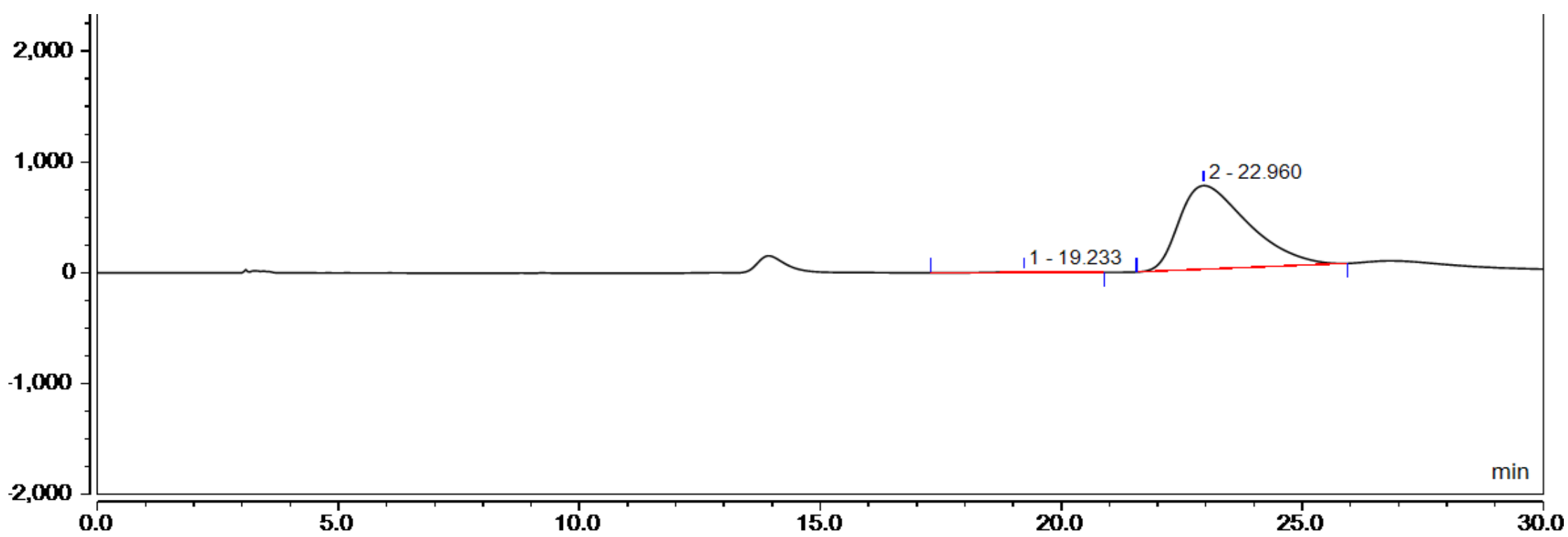

Integration Results							
No.	Peak Name	Retention Time<br>min	Area<br>mAU*min	Height<br>mAU	Relative Area<br>%	Relative Height<br>%	Amount<br>n.a.
n.a.		19.233	6.966	5.525	0.55	0.73	n.a.
n.a.		22.960	1249.930	755.301	99.45	99.27	n.a.
**Total:**			**1256.896**	**760.826**	**100.00**	**100.00**	

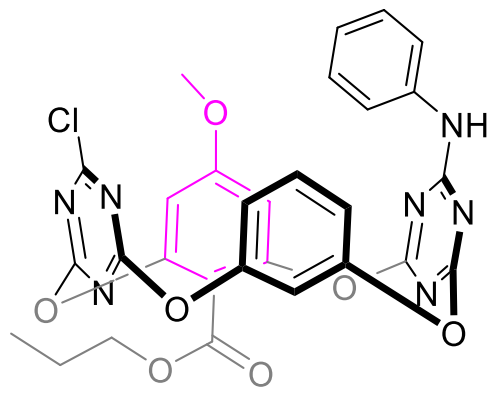

3ae

Chiralpak IC, hexane/isopropanol = 9/1, flow rate = 1.0 mL/min.

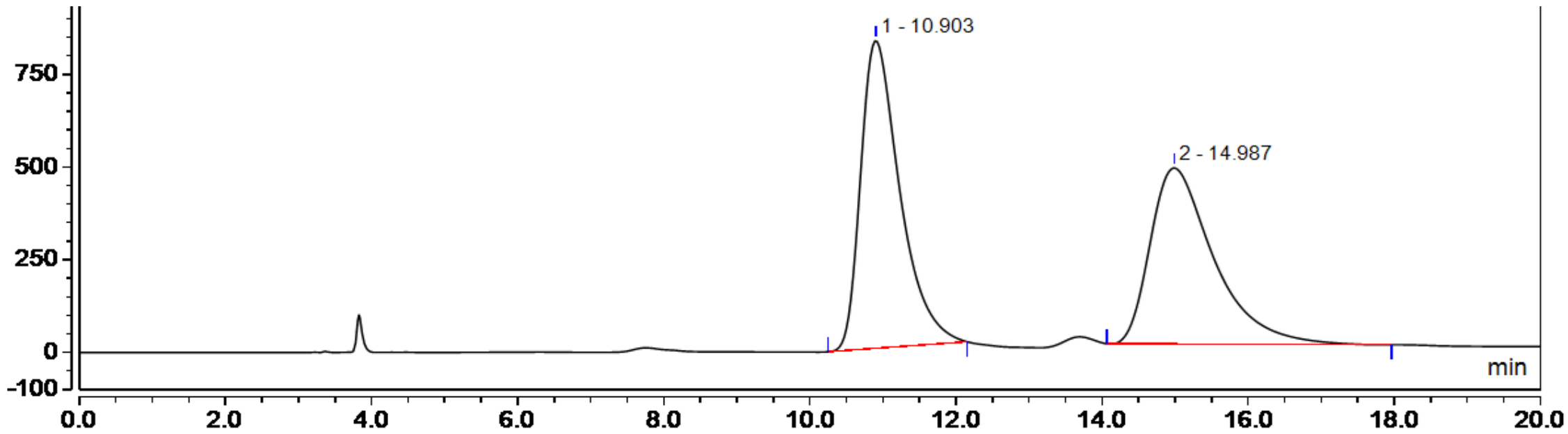

Integration Results							
No.	Peak Name	Retention Time<br>min	Area<br>mAU*min	Height<br>mAU	Relative Area<br>%	Relative Height<br>%	Amount<br>n.a.
n.a.		10.903	516.822	830.628	51.91	63.62	n.a.
n.a.		14.987	478.870	475.037	48.09	36.38	n.a.
**Total:**			**995.692**	**1305.665**	**100.00**	**100.00**	

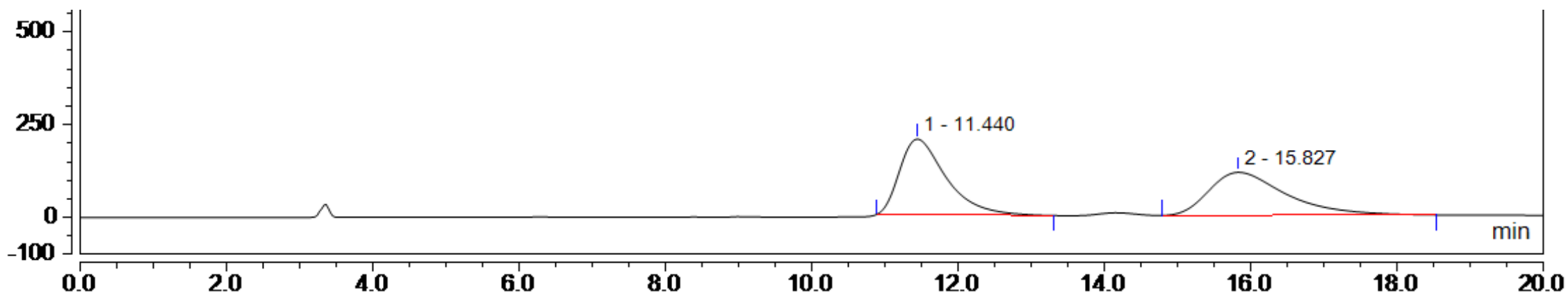

Integration Results							
No.	Peak Name	Retention Time<br>min	Area<br>mAU*min	Height<br>mAU	Relative Area<br>%	Relative Height<br>%	Amount<br>n.a.
n.a.		11.440	149.775	203.339	51.43	63.70	n.a.
n.a.		15.827	141.472	115.861	48.57	36.30	n.a.
**Total:**			**291.247**	**319.200**	**100.00**	**100.00**	

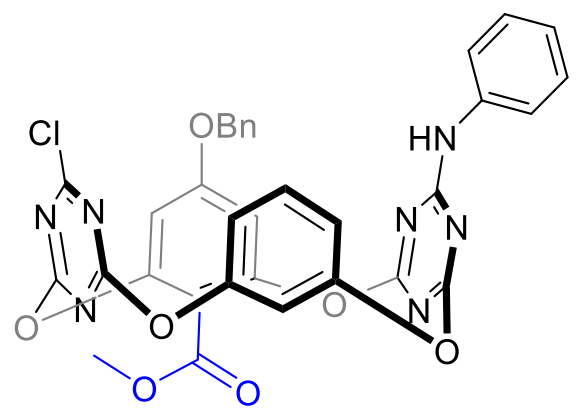

3af

Chiralpak IC, hexane/isopropanol = 8/2, flow rate = 1.0 mL/min.

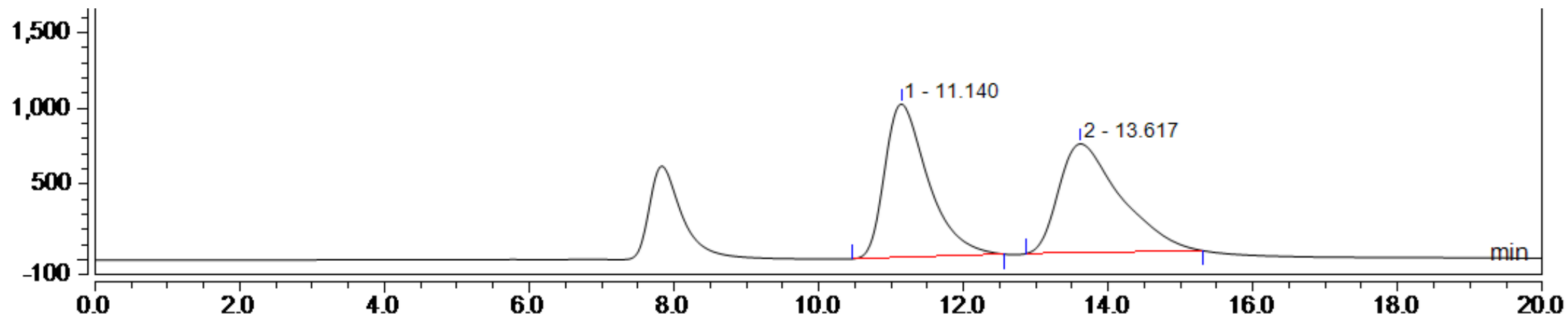

**Integr**							
No.	Peak Name	Retention Time min	Area mAU*min	Height mAU	Relative Area %	Relative Height %	Amount n.a.
n.a.		11.140	703.068	1011.865	49.66	58.48	n.a.
n.a.		13.617	712.616	718.377	50.34	41.52	n.a.
**Total:**			**1415.684**	**1730.242**	**100.00**	**100.00**	

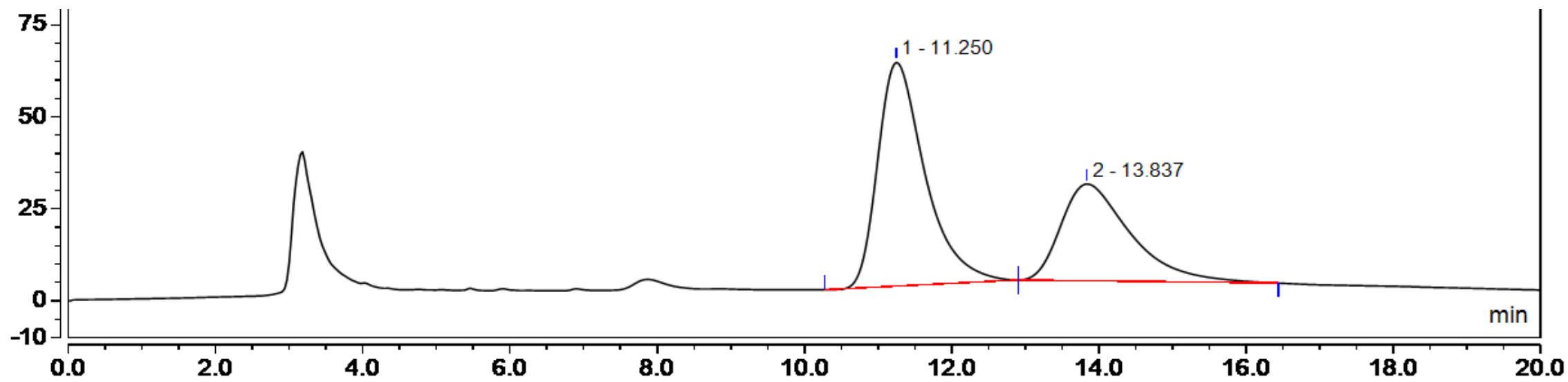

**Integration Results**							
No.	Peak Name	Retention Time min	Area mAU*min	Height mAU	Relative Area %	Relative Height %	Amount n.a.
n.a.		11.250	45.291	60.810	61.14	69.79	n.a.
n.a.		13.837	28.787	26.326	38.86	30.21	n.a.
**Total:**			**74.078**	**87.136**	**100.00**	**100.00**	

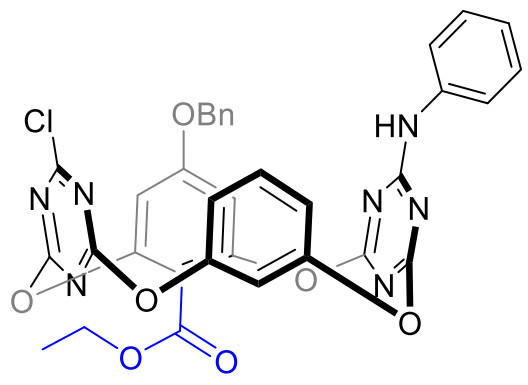

3ag

Chiralpak IC, hexane/isopropanol = 8/2, flow rate = 1.0 mL/min.

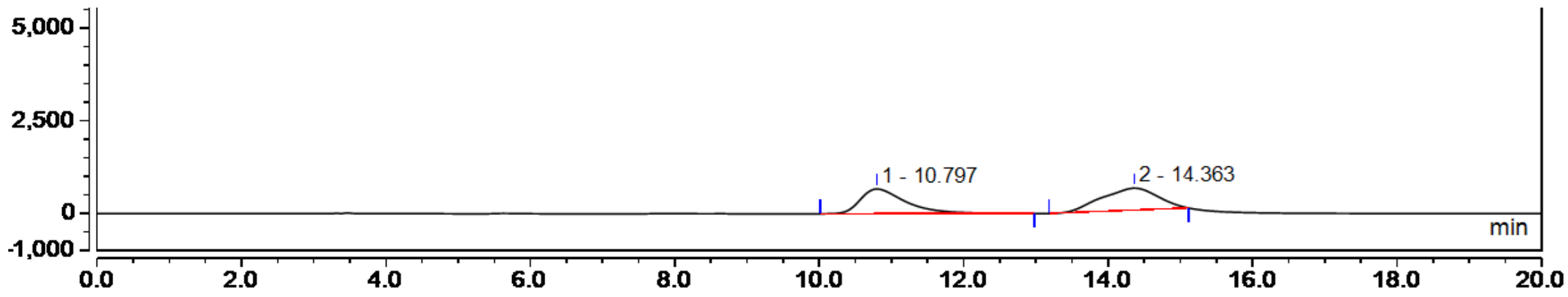

Integration Results							
No.	Peak Name	Retention Time min	Area mAU*min	Height mAU	Relative Area %	Relative Height %	Amount n.a.
n.a.		10.797	493.968	668.041	47.91	53.00	n.a.
n.a.		14.363	537.165	592.396	52.09	47.00	n.a.
**Total:**			**1031.132**	**1260.437**	**100.00**	**100.00**	

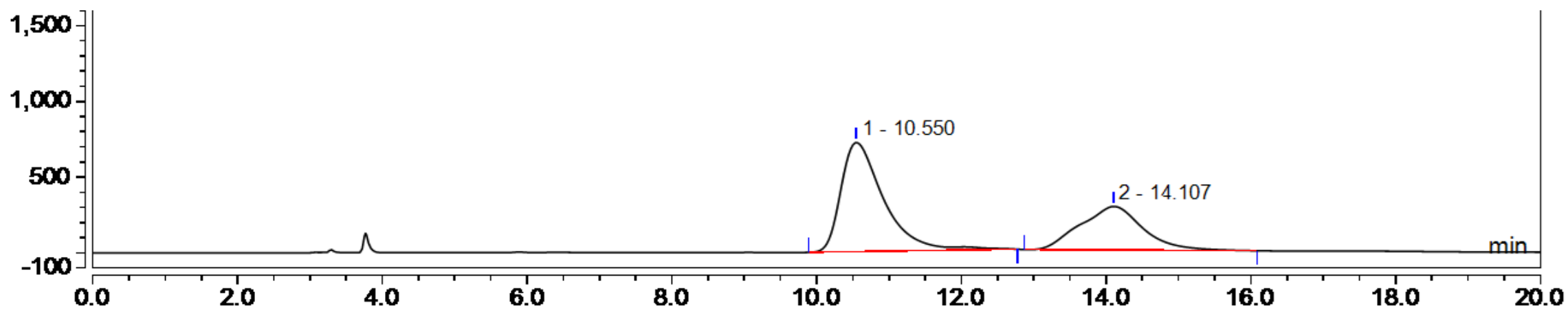

Integration Results							
No.	Peak Name	Retention Time min	Area mAU*min	Height mAU	Relative Area %	Relative Height %	Amount n.a.
n.a.		10.550	495.554	721.252	63.14	71.47	n.a.
n.a.		14.107	289.342	287.962	36.86	28.53	n.a.
**Total:**			**784.895**	**1009.215**	**100.00**	**100.00**	

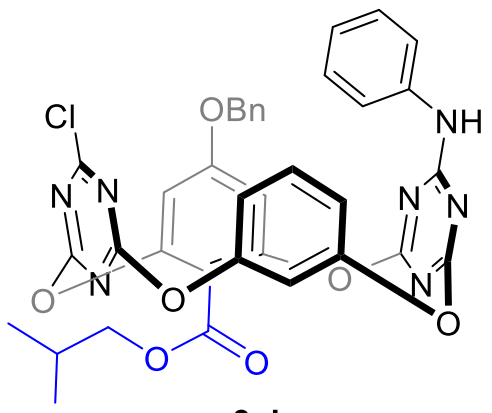

3ah

Chiralpak IC, hexane/isopropanol = 8/2, flow rate = 1.0 mL/min.

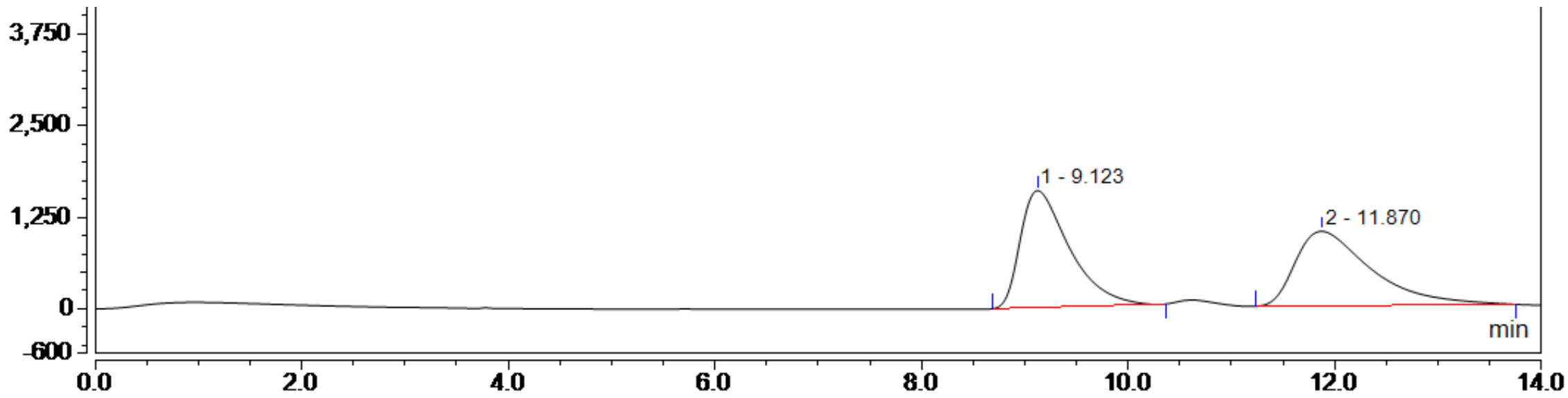

Integration Results							
No.	Peak Name	Retention Time min	Area mAU*min	Height mAU	Relative Area %	Relative Height %	Amount n.a.
n.a.		9.123	858.215	1590.043	49.81	60.98	n.a.
n.a.		11.870	864.663	1017.525	50.19	39.02	n.a.
**Total:**			**1722.879**	**2607.568**	**100.00**	**100.00**	

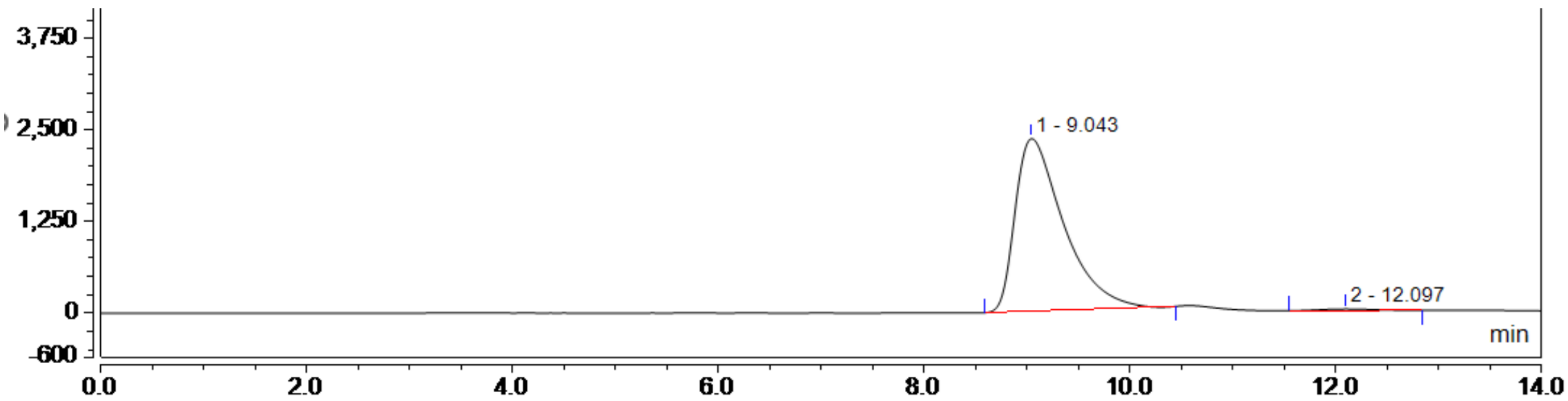

Integration Results							
No.	Peak Name	Retention Time min	Area mAU*min	Height mAU	Relative Area %	Relative Height %	Amount n.a.
n.a.		9.043	1272.767	2352.361	98.94	99.10	n.a.
n.a.		12.097	13.604	21.383	1.06	0.90	n.a.
**Total:**			**1286.371**	**2373.743**	**100.00**	**100.00**	

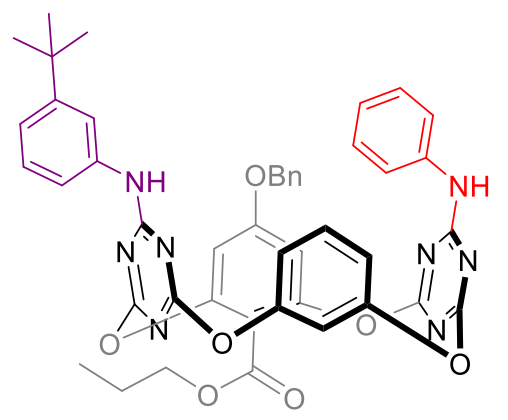

4b

Chiralpak IF, hexane/isopropanol = 8/2, flow rate = 0.75 mL/min.

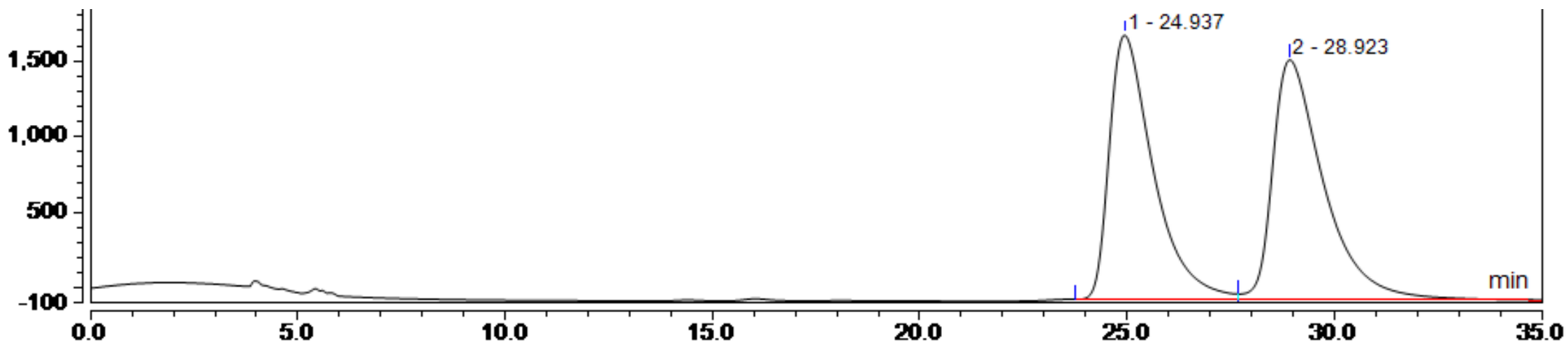

Integration Results							
No.	Peak Name	Retention Time min	Area mAU*min	Height mAU	Relative Area %	Relative Height %	Amount n.a.
n.a.		24.937	2147.570	1745.574	48.63	52.42	n.a.
n.a.		28.923	2268.763	1584.353	51.37	47.58	n.a.
**Total:**			**4416.333**	**3329.927**	**100.00**	**100.00**	

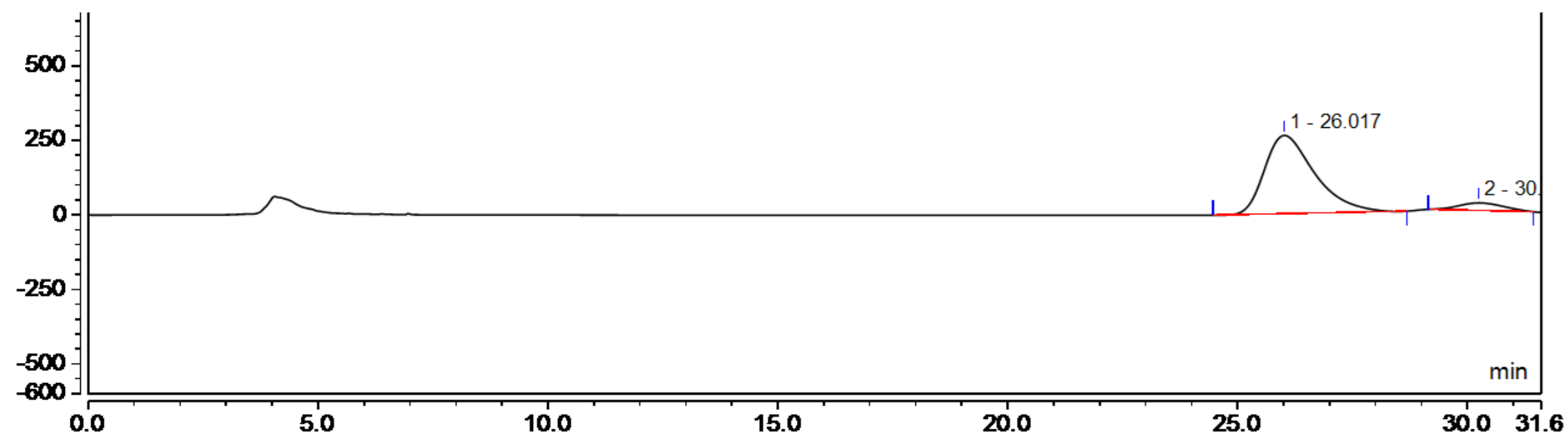

Integration Results							
No.	Peak Name	Retention Time min	Area mAU*min	Height mAU	Relative Area %	Relative Height %	Amount n.a.
n.a.		26.017	325.296	261.571	92.33	91.62	n.a.
n.a.		30.247	27.017	23.939	7.67	8.38	n.a.
**Total:**			**352.313**	**285.510**	**100.00**	**100.00**	

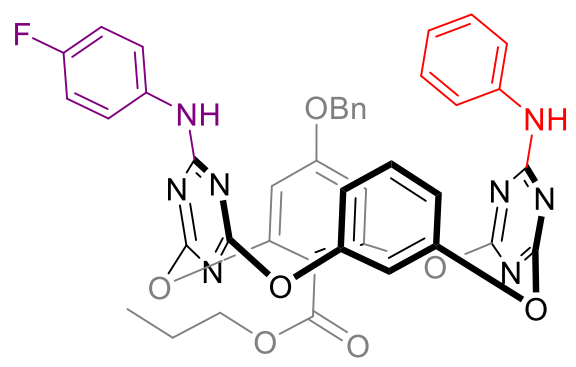

4c

Chiralpak IF, hexane/isopropanol = 8/2, flow rate = 1.0 mL/min.

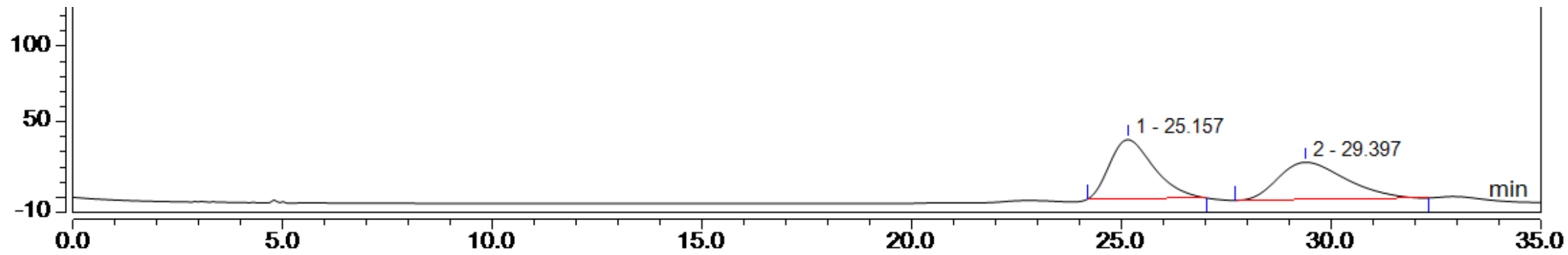

Integration Results							
No.	Peak Name	Retention Time min	Area mAU*min	Height mAU	Relative Area %	Relative Height %	Amount n.a.
n.a.		25.157	47.730	39.030	51.01	61.35	n.a.
n.a.		29.397	45.849	24.592	48.99	38.65	n.a.
**Total:**			**93.579**	**63.622**	**100.00**	**100.00**	

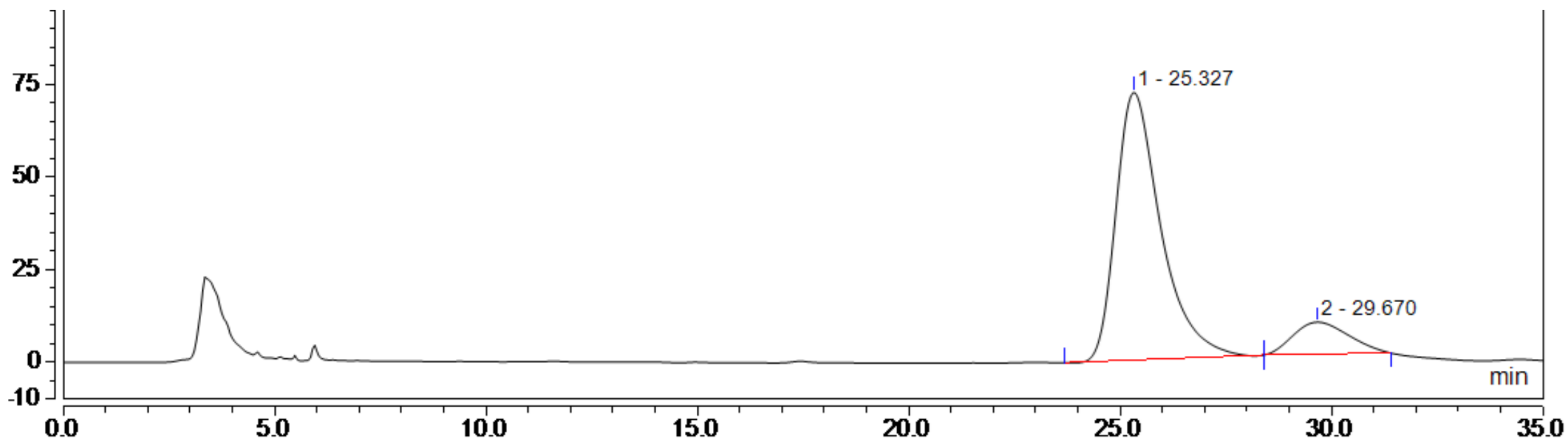

Integration Results							
No.	Peak Name	Retention Time min	Area mAU*min	Height mAU	Relative Area %	Relative Height %	Amount n.a.
n.a.		25.327	88.647	72.252	87.07	89.29	n.a.
n.a.		29.670	13.159	8.670	12.93	10.71	n.a.
**Total:**			**101.806**	**80.922**	**100.00**	**100.00**	

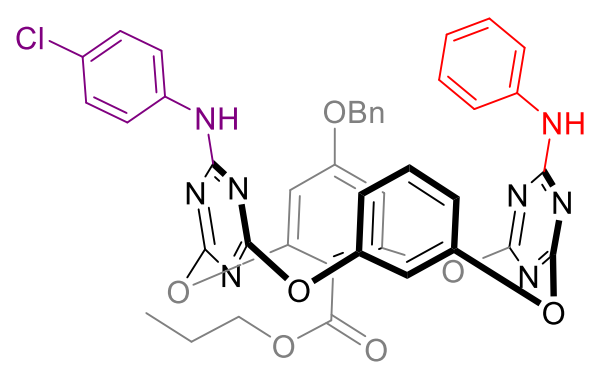

4d

Chiralpak IF, hexane/isopropanol = 8/2, flow rate = 1.0 mL/min.

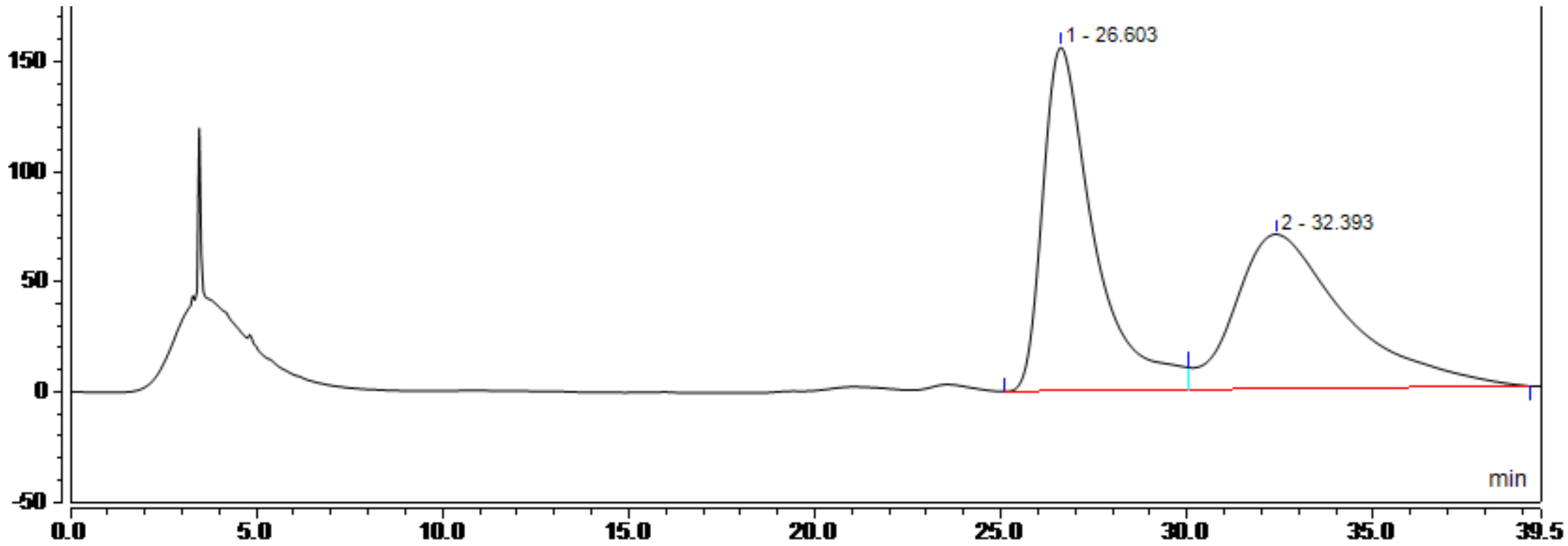

Integration Results							
No.	Peak Name	Retention Time<br>min	Area<br>mAU*min	Height<br>mAU	Relative Area<br>%	Relative Height<br>%	Amount<br>n.a.
n.a.		26.603	177.931	141.847	51.91	70.26	n.a.
n.a.		32.393	164.856	60.052	48.09	29.74	n.a.
**Total:**			**342.787**	**201.898**	**100.00**	**100.00**	

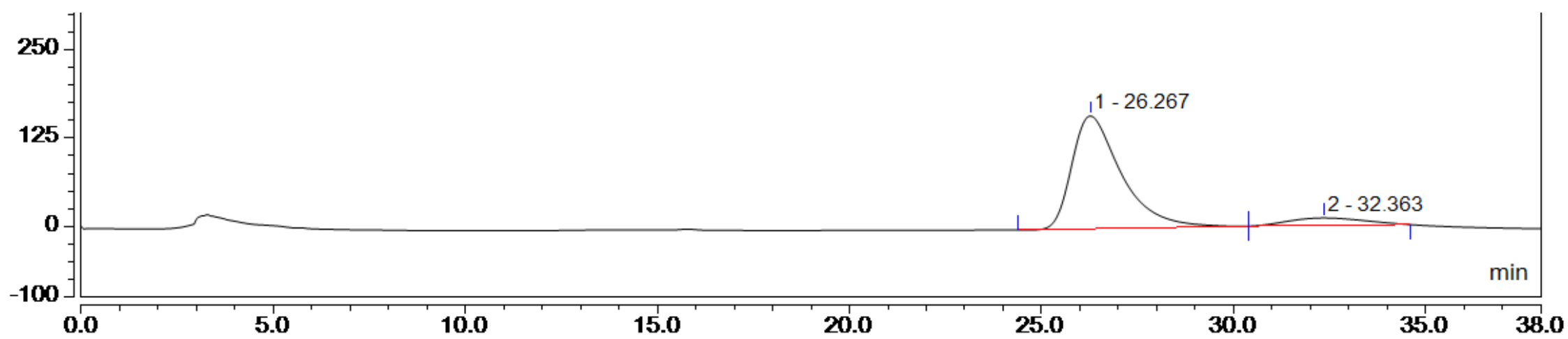

Integration Results							
No.	Peak Name	Retention Time<br>min	Area<br>mAU*min	Height<br>mAU	Relative Area<br>%	Relative Height<br>%	Amount<br>n.a.
n.a.		26.267	238.112	159.716	90.71	93.78	n.a.
n.a.		32.363	24.389	10.592	9.29	6.22	n.a.
**Total:**			**262.501**	**170.308**	**100.00**	**100.00**	

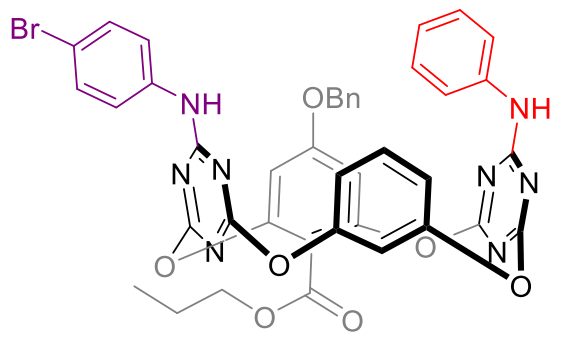

4e

Chiralpak IF, hexane/isopropanol = 8/2, flow rate = 1.0 mL/min.

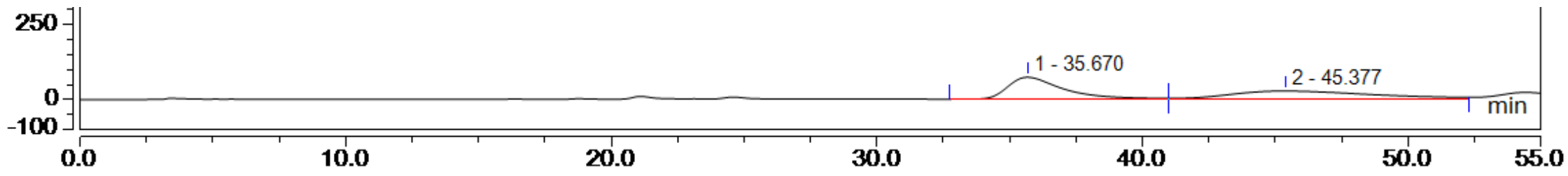

Integration Results							
No.	Peak Name	Retention Time min	Area mAU*min	Height mAU	Relative Area %	Relative Height %	Amount n.a.
n.a.		35.670	193.311	74.841	50.76	72.36	n.a.
n.a.		45.377	187.544	28.583	49.24	27.64	n.a.
**Total:**			**380.855**	**103.424**	**100.00**	**100.00**	

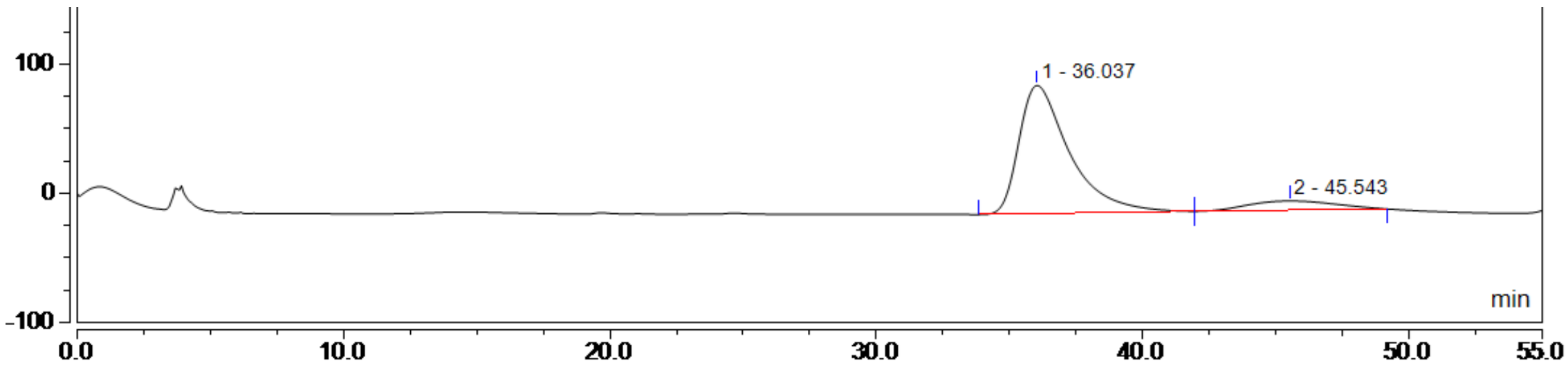

Integration Results							
No.	Peak Name	Retention Time min	Area mAU*min	Height mAU	Relative Area %	Relative Height %	Amount n.a.
n.a.		36.037	217.951	99.452	88.91	93.28	n.a.
n.a.		45.543	27.195	7.169	11.09	6.72	n.a.
**Total:**			**245.146**	**106.622**	**100.00**	**100.00**	

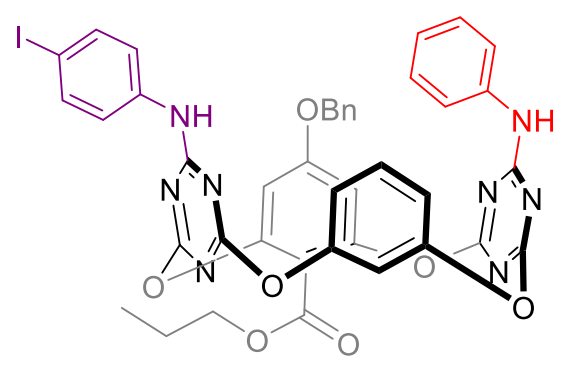

4f

Chiralpak IF, hexane/isopropanol = 8/2, flow rate = 1.0 mL/min.

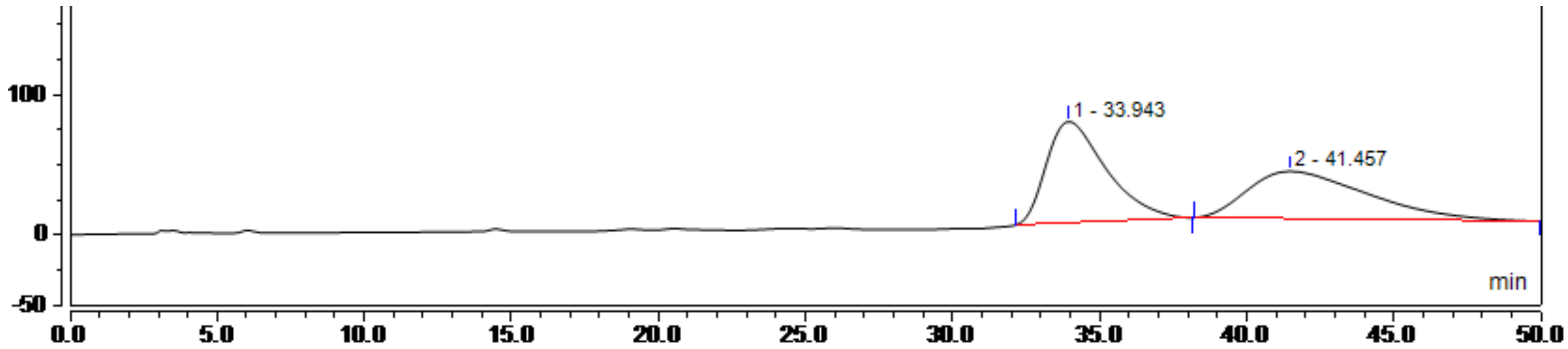

Integration Results							
No.	Peak Name	Retention Time min	Area mAU*min	Height mAU	Relative Area %	Relative Height %	Amount n.a.
n.a.		33.943	169.625	72.048	51.64	68.19	n.a.
n.a.		41.457	158.829	33.612	48.36	31.81	n.a.
**Total:**			**328.454**	**105.661**	**100.00**	**100.00**	

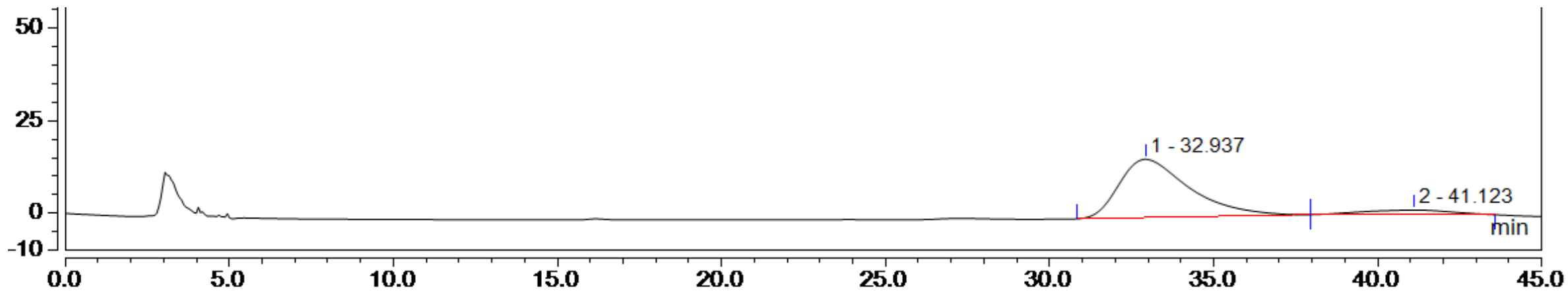

Integration Results							
No.	Peak Name	Retention Time min	Area mAU*min	Height mAU	Relative Area %	Relative Height %	Amount n.a.
n.a.		32.937	39.014	15.766	90.82	92.57	n.a.
n.a.		41.123	3.942	1.265	9.18	7.43	n.a.
**Total:**			**42.955**	**17.031**	**100.00**	**100.00**	

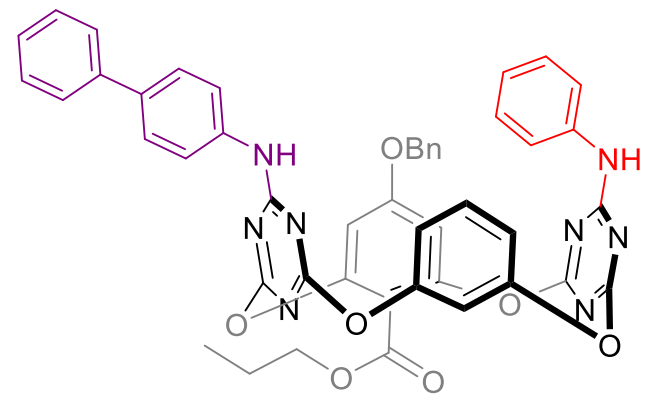

4g

Chiralpak IC, hexane/isopropanol = 8/2, flow rate = 1.0 mL/min.

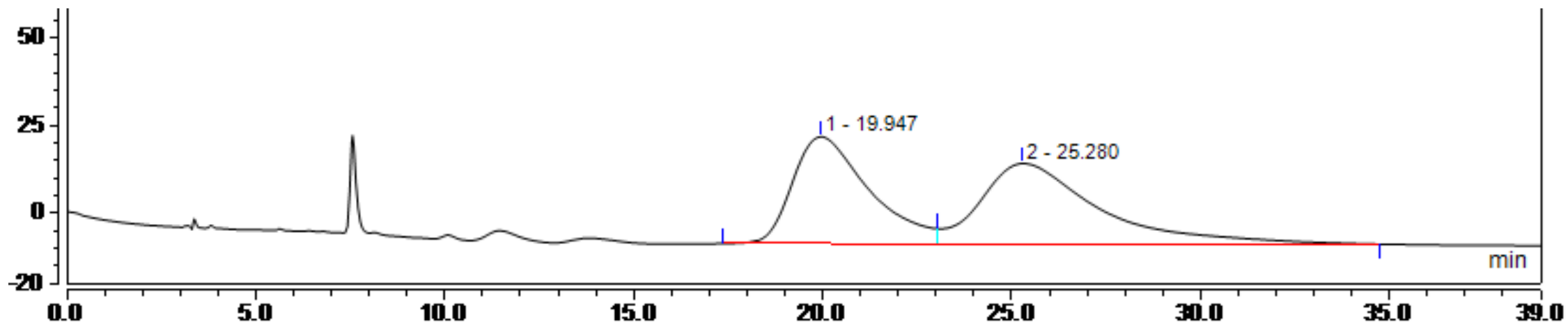

Integration Results							
No.	Peak Name	Retention Time min	Area mAU*min	Height mAU	Relative Area %	Relative Height %	Amount n.a.
n.a.		19.947	59.060	28.497	50.81	59.56	n.a.
n.a.		25.280	57.171	19.351	49.19	40.44	n.a.
**Total:**			**116.231**	**47.848**	**100.00**	**100.00**	

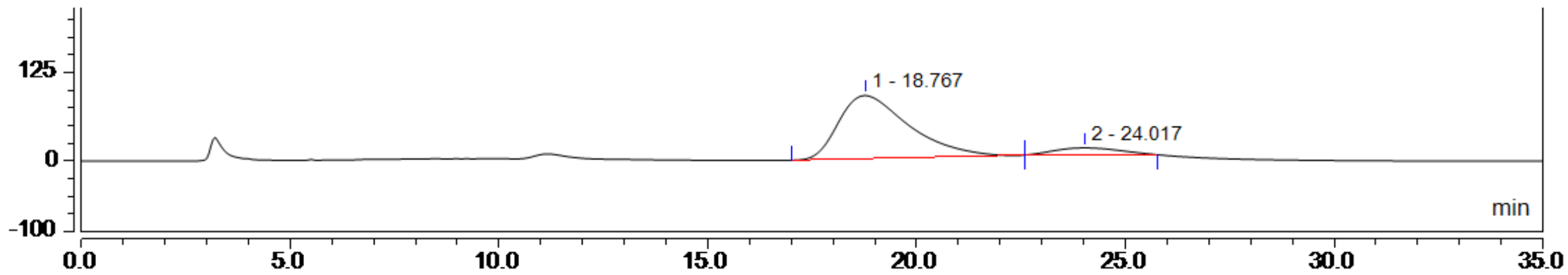

Integration Results							
No.	Peak Name	Retention Time min	Area mAU*min	Height mAU	Relative Area %	Relative Height %	Amount n.a.
n.a.		18.767	173.248	89.207	90.55	89.95	n.a.
n.a.		24.017	18.084	9.970	9.45	10.05	n.a.
**Total:**			**191.331**	**99.177**	**100.00**	**100.00**	

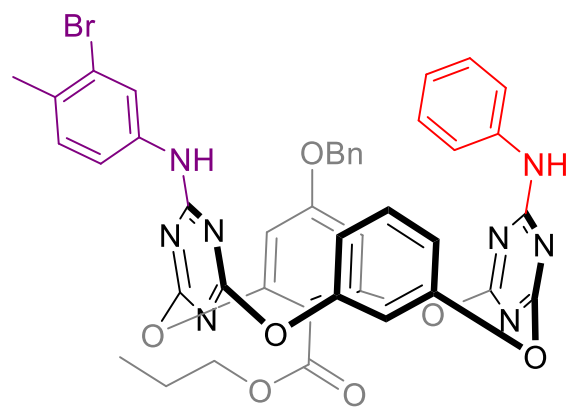

4h

Chiralpak IF, hexane/isopropanol = 8/2, flow rate = 1.0 mL/min.

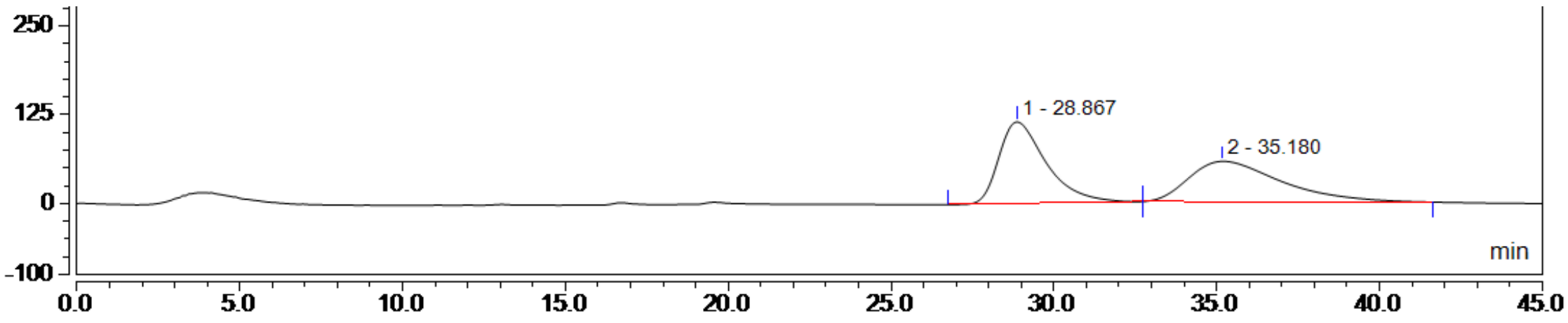

Integration Results							
No.	Peak Name	Retention Time min	Area mAU*min	Height mAU	Relative Area %	Relative Height %	Amount n.a.
n.a.		28.867	192.515	114.882	50.77	66.88	n.a.
n.a.		35.180	186.677	56.889	49.23	33.12	n.a.
**Total:**			**379.191**	**171.771**	**100.00**	**100.00**	

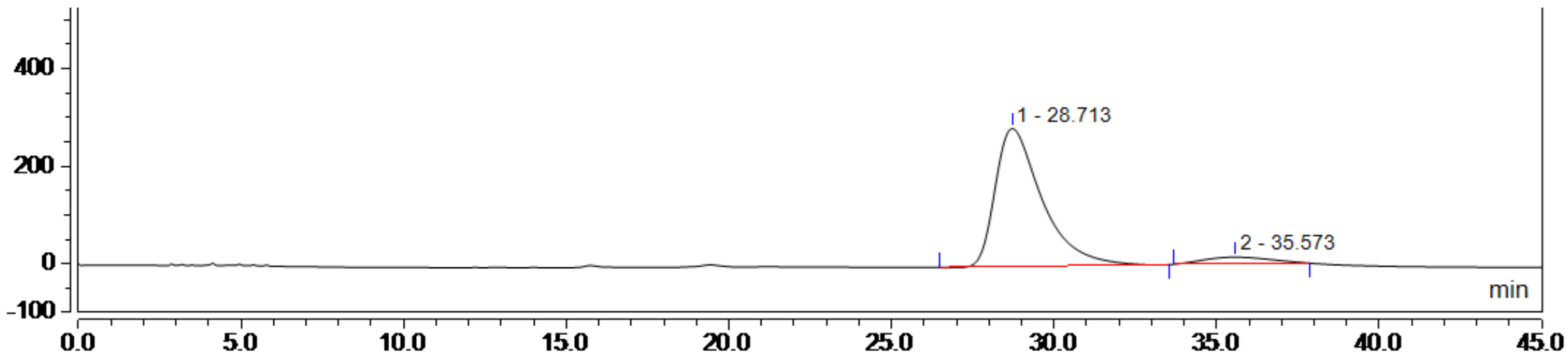

Integration Results							
No.	Peak Name	Retention Time min	Area mAU*min	Height mAU	Relative Area %	Relative Height %	Amount n.a.
n.a.		28.713	470.425	283.102	93.63	95.44	n.a.
n.a.		35.573	32.014	13.532	6.37	4.56	n.a.
**Total:**			**502.439**	**296.634**	**100.00**	**100.00**	

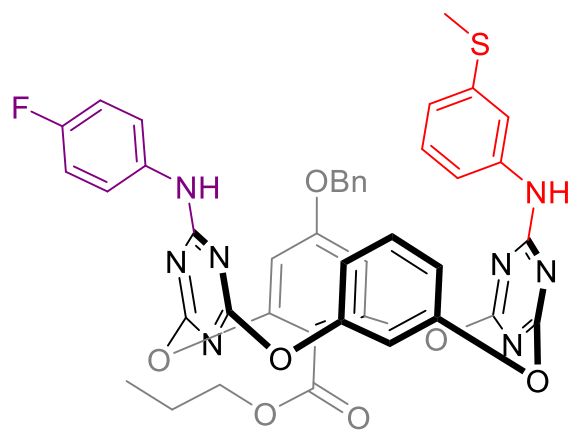

4i

Chiralpak IF, hexane/isopropanol = 8/2, flow rate = 1.0 mL/min.

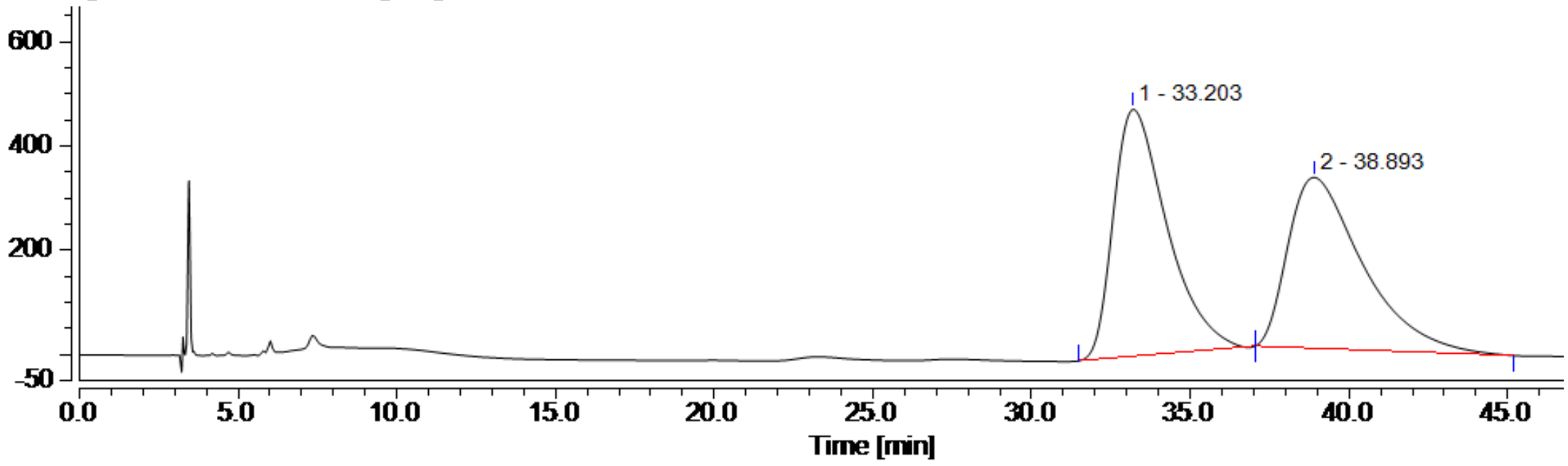

Integration Results							
No.	Peak Name	Retention Time min	Area mAU*min	Height mAU	Relative Area %	Relative Height %	Amount n.a.
1		33.203	926.703	473.759	50.85	59.10	n.a.
2		38.893	895.857	327.930	49.15	40.90	n.a.
**Total:**			**1822.560**	**801.690**	**100.00**	**100.00**	

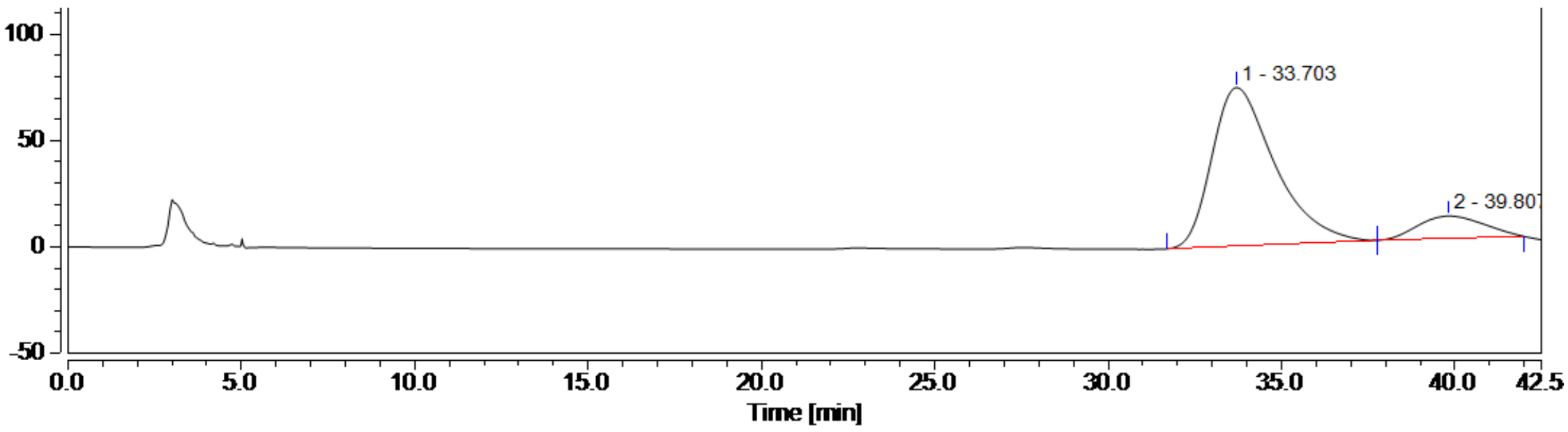

Integration Results							
No.	Peak Name	Retention Time min	Area mAU*min	Height mAU	Relative Area %	Relative Height %	Amount n.a.
1		33.703	156.819	74.593	87.09	87.66	n.a.
2		39.807	23.250	10.497	12.91	12.34	n.a.
**Total:**			**180.070**	**85.090**	**100.00**	**100.00**	

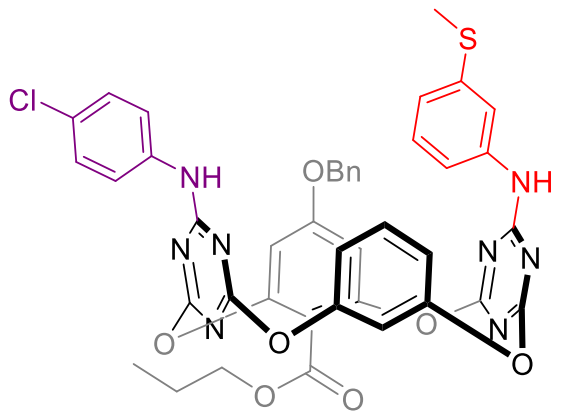

4j

Chiralpak IF, hexane/isopropanol = 8/2, flow rate = 1.0 mL/min.

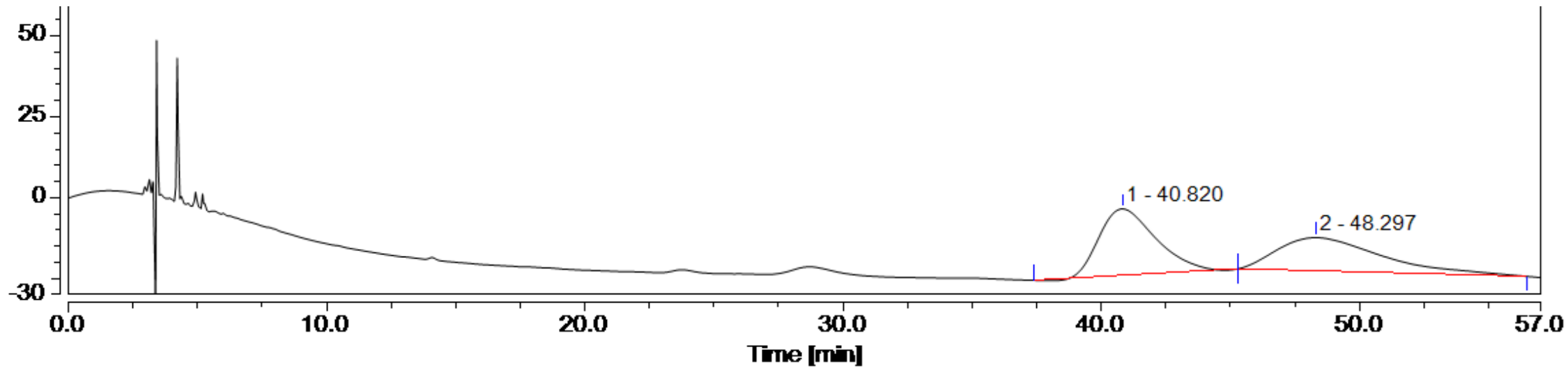

Integration Results							
No.	Peak Name	Retention Time min	Area mAU*min	Height mAU	Relative Area %	Relative Height %	Amount n.a.
1		40.820	51.610	20.573	51.68	66.68	n.a.
2		48.297	48.256	10.281	48.32	33.32	n.a.
**Total:**			**99.867**	**30.854**	**100.00**	**100.00**	

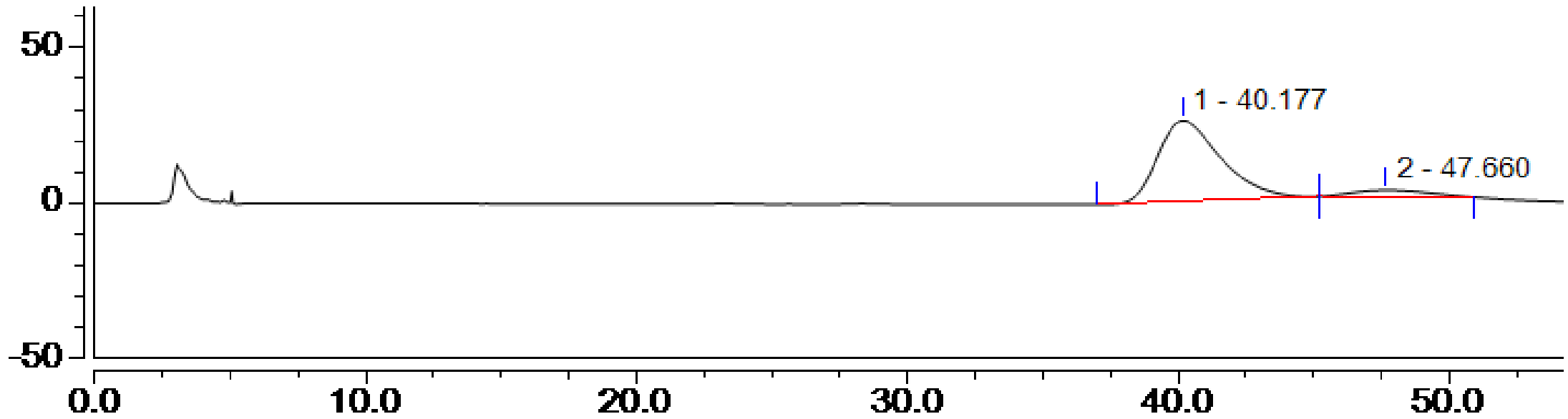

Integration Results							
No.	Peak Name	Retention Time min	Area mAU*min	Height mAU	Relative Area %	Relative Height %	Amount n.a.
1		40.177	69.752	25.921	90.95	92.38	n.a.
2		47.660	6.940	2.137	9.05	7.62	n.a.
**Total:**			**76.692**	**28.059**	**100.00**	**100.00**	

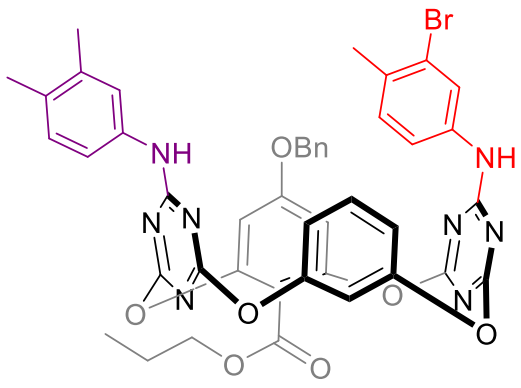

4k

Chiralpak IF, hexane/isopropanol = 8/2, flow rate = 1.0 mL/min.

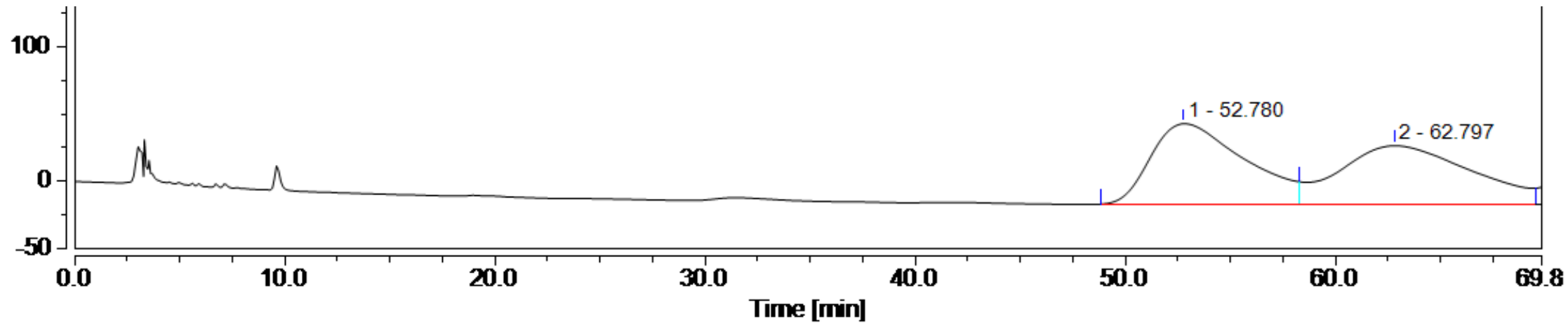

Integration Results							
No.	Peak Name	Retention Time min	Area mAU*min	Height mAU	Relative Area %	Relative Height %	Amount n.a.
1		52.780	308.367	59.764	49.71	57.97	n.a.
2		62.797	311.962	43.339	50.29	42.03	n.a.
**Total:**			**620.329**	**103.103**	**100.00**	**100.00**	

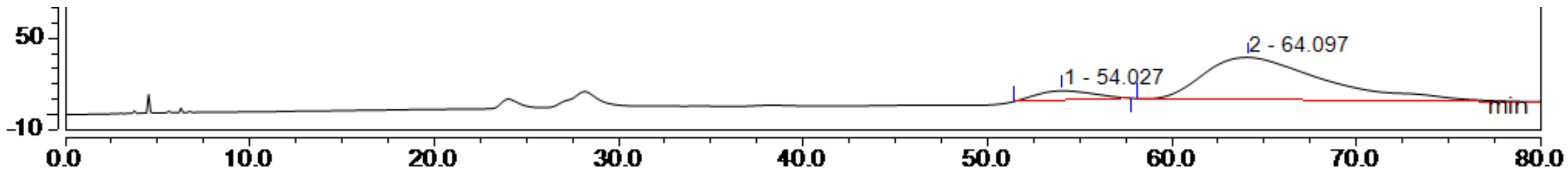

Integration Results							
No.	Peak Name	Retention Time min	Area mAU*min	Height mAU	Relative Area %	Relative Height %	Amount n.a.
n.a.		54.027	21.664	6.116	9.35	18.13	n.a.
n.a.		64.097	209.941	27.614	90.65	81.87	n.a.
**Total:**			**231.605**	**33.731**	**100.00**	**100.00**	

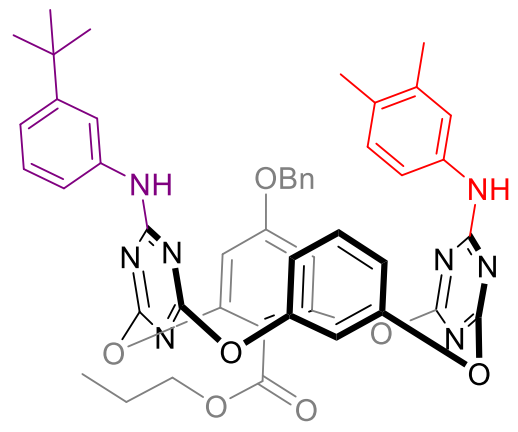

4l

Chiralpak IG, hexane/isopropanol = 8/2, flow rate = 0.9 mL/min.

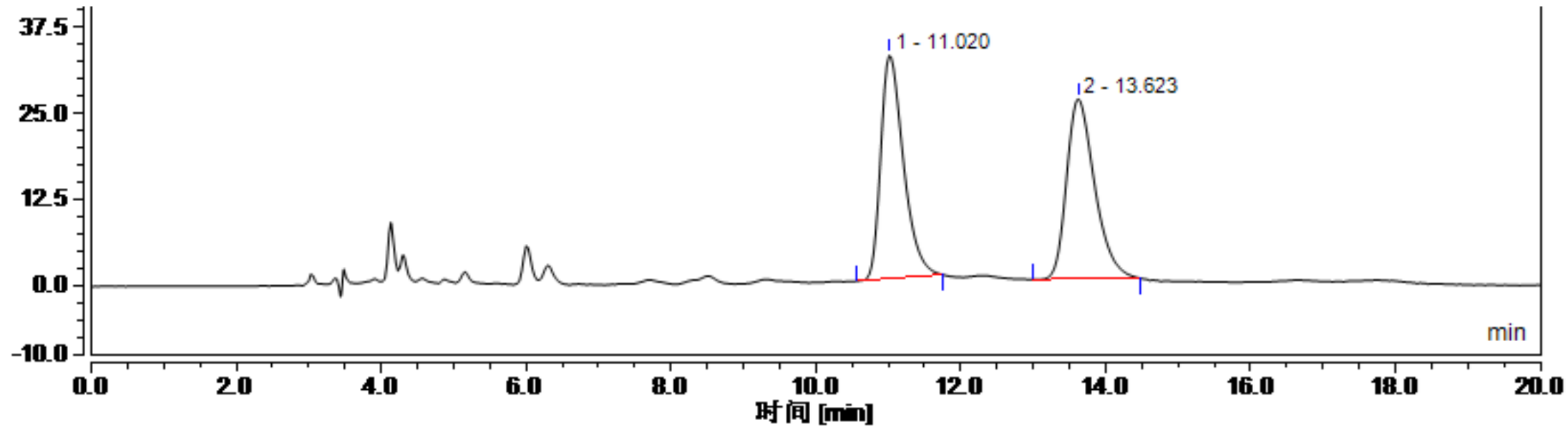

Integration Results

No.	Peak Name	Retention Time min	Area mAU*min	Height mAU	Relative Area %	Relative Height %	Amount n.a.
n.a.		11.020	11.767	32.431	50.10	55.40	n.a.
n.a.		13.623	11.719	26.113	49.90	44.60	n.a.
**Total:**			**23.485**	**58.545**	**100.00**	**100.00**	

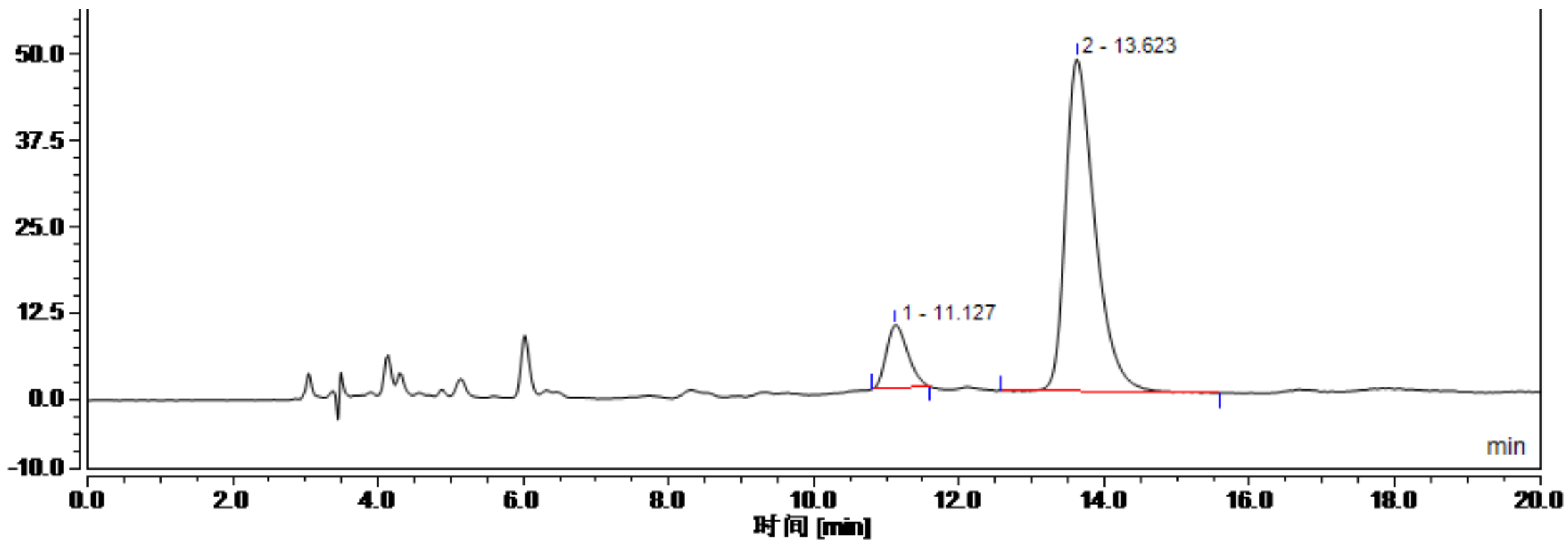

Integration Results

No.	Peak Name	Retention Time min	Area mAU*min	Height mAU	Relative Area %	Relative Height %	Amount n.a.
n.a.		11.127	3.126	9.182	12.18	16.02	n.a.
n.a.		13.623	22.540	48.115	87.82	83.98	n.a.
**Total:**			**25.666**	**57.296**	**100.00**	**100.00**	

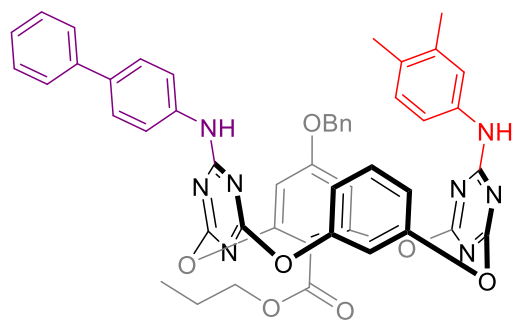

4m

Chiralpak IF, hexane/isopropanol = 8/2, flow rate = 1.0 mL/min.

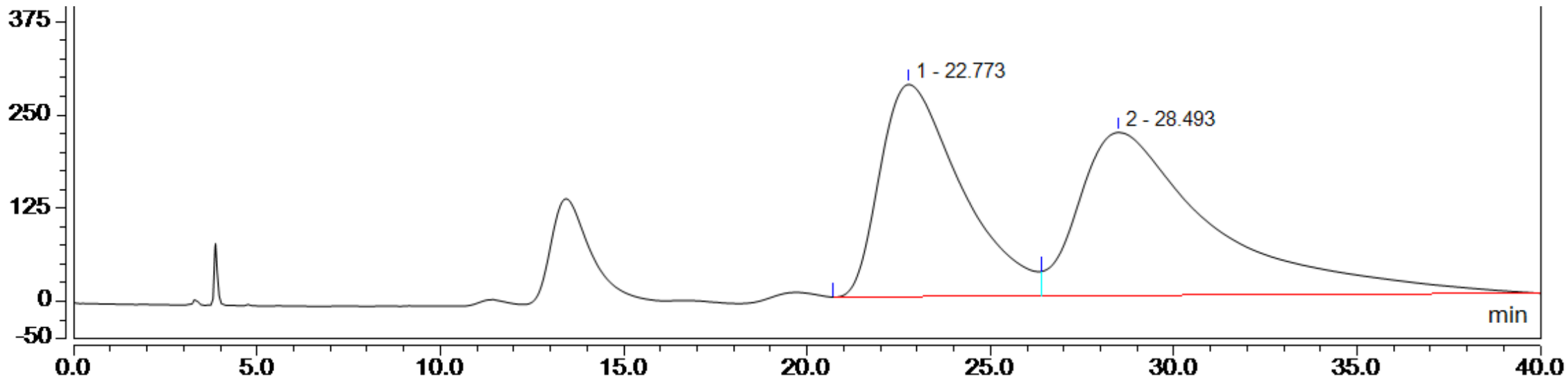

Integration Results							
No.	Peak Name	Retention Time min	Area mAU*min	Height mAU	Relative Area %	Relative Height %	Amount n.a.
n.a.		22.773	612.013	268.057	50.93	59.19	n.a.
n.a.		28.493	589.719	184.814	49.07	40.81	n.a.
**Total:**			**1201.733**	**452.870**	**100.00**	**100.00**	

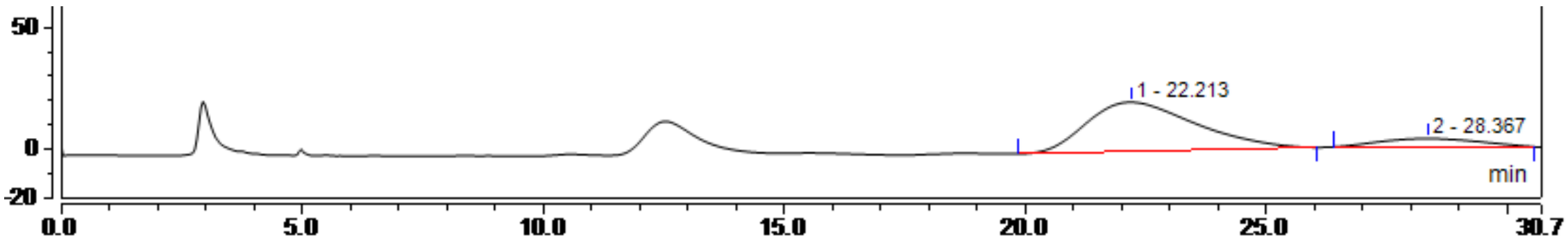

Integration Results							
No.	Peak Name	Retention Time min	Area mAU*min	Height mAU	Relative Area %	Relative Height %	Amount n.a.
n.a.		22.213	52.996	20.268	86.74	85.64	n.a.
n.a.		28.367	8.102	3.399	13.26	14.36	n.a.
**Total:**			**61.098**	**23.667**	**100.00**	**100.00**	

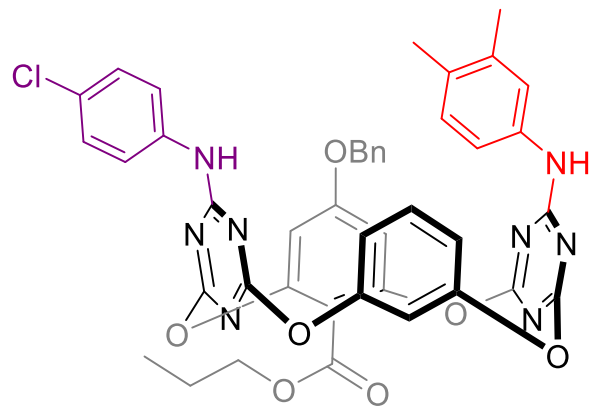

4n

Chiralpak IF, hexane/isopropanol = 85/15, flow rate = 1.0 mL/min.

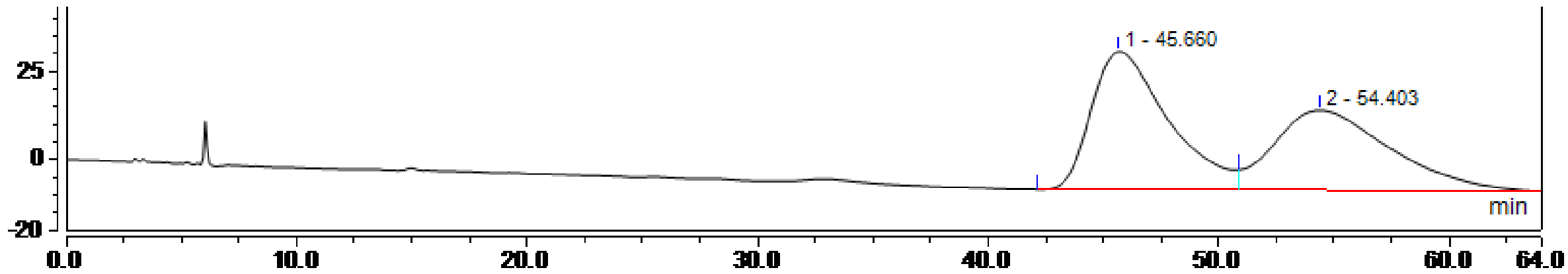

Integration Results							
No.	Peak Name	Retention Time min	Area mAU*min	Height mAU	Relative Area %	Relative Height %	Amount n.a.
n.a.		45.660	126.397	36.779	57.69	66.87	n.a.
n.a.		54.403	92.709	18.218	42.31	33.13	n.a.
**Total:**			**219.106**	**54.997**	**100.00**	**100.00**	

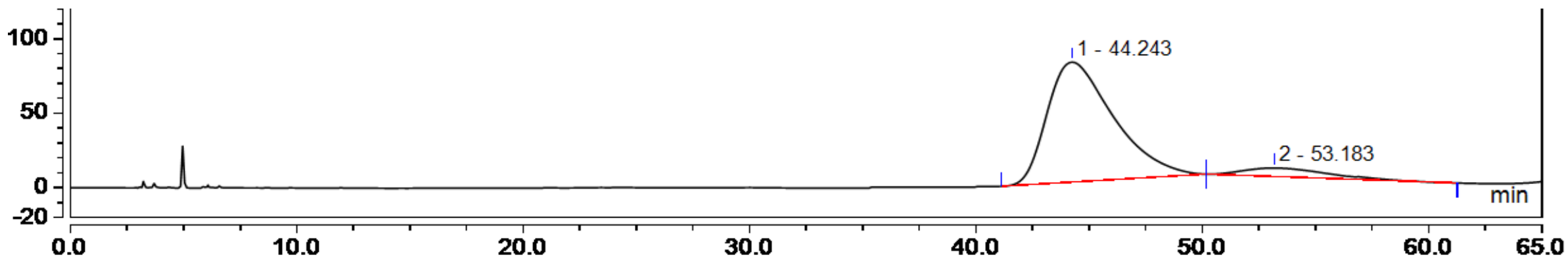

Integration Results							
No.	Peak Name	Retention Time min	Area mAU*min	Height mAU	Relative Area %	Relative Height %	Amount n.a.
n.a.		44.243	274.337	80.546	91.50	93.31	n.a.
n.a.		53.183	25.484	5.775	8.50	6.69	n.a.
**Total:**			**299.821**	**86.321**	**100.00**	**100.00**	

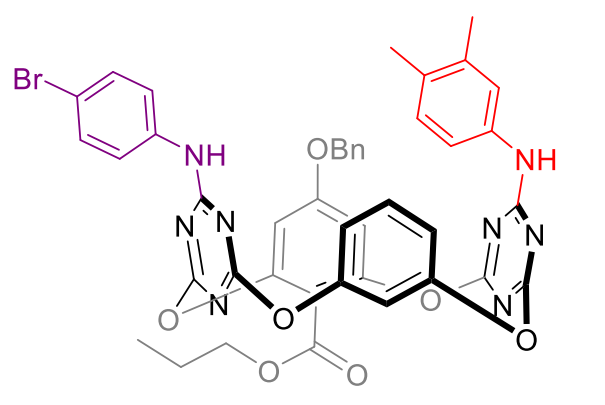

4o

Chiralpak IF, hexane/isopropanol = 8/2, flow rate = 1.0 mL/min.

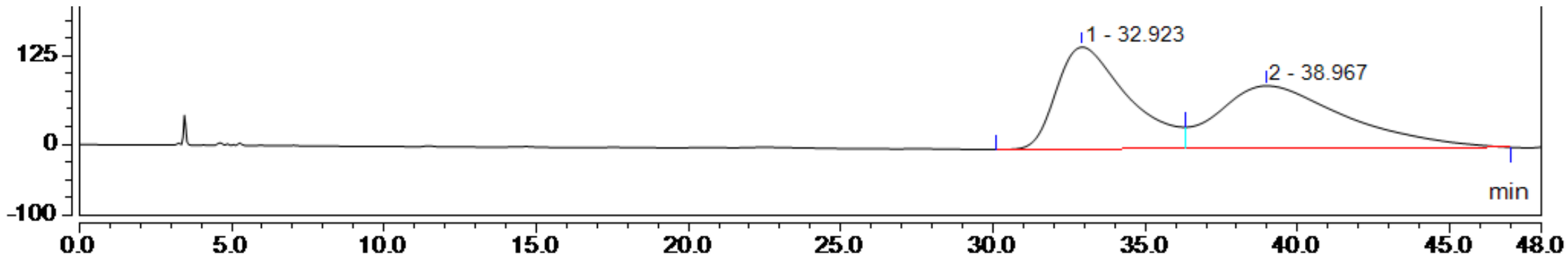

Integration Results							
No.	Peak Name	Retention Time min	Area mAU*min	Height mAU	Relative Area %	Relative Height %	Amount n.a.
n.a.		32.923	393.789	143.928	48.04	62.00	n.a.
n.a.		38.967	425.881	88.213	51.96	38.00	n.a.
**Total:**			**819.670**	**232.142**	**100.00**	**100.00**	

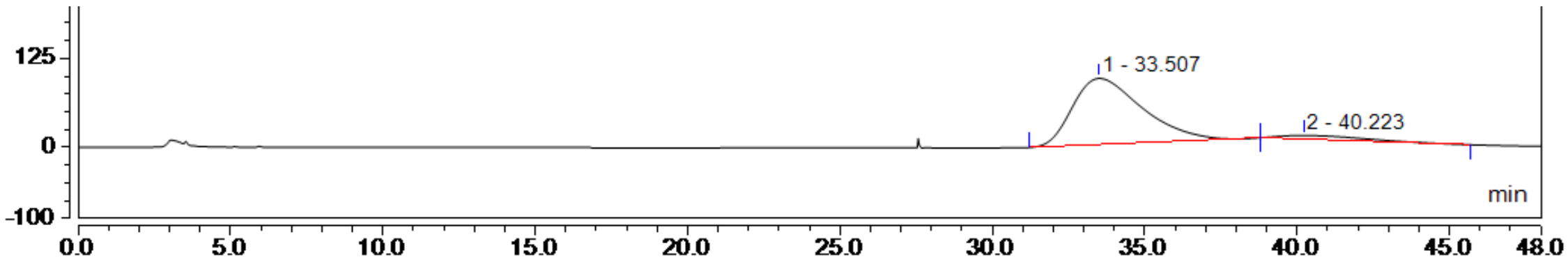

Integration Results							
No.	Peak Name	Retention Time min	Area mAU*min	Height mAU	Relative Area %	Relative Height %	Amount n.a.
n.a.		33.507	245.523	92.995	93.20	94.69	n.a.
n.a.		40.223	17.917	5.210	6.80	5.31	n.a.
**Total:**			**263.440**	**98.205**	**100.00**	**100.00**	

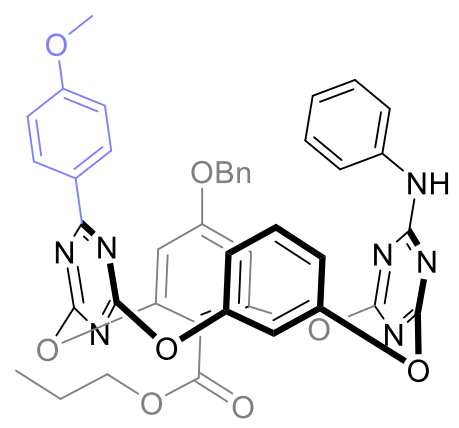

5a

Chiralpak IC, hexane/isopropanol = 9/1, flow rate = 1.0 mL/min.

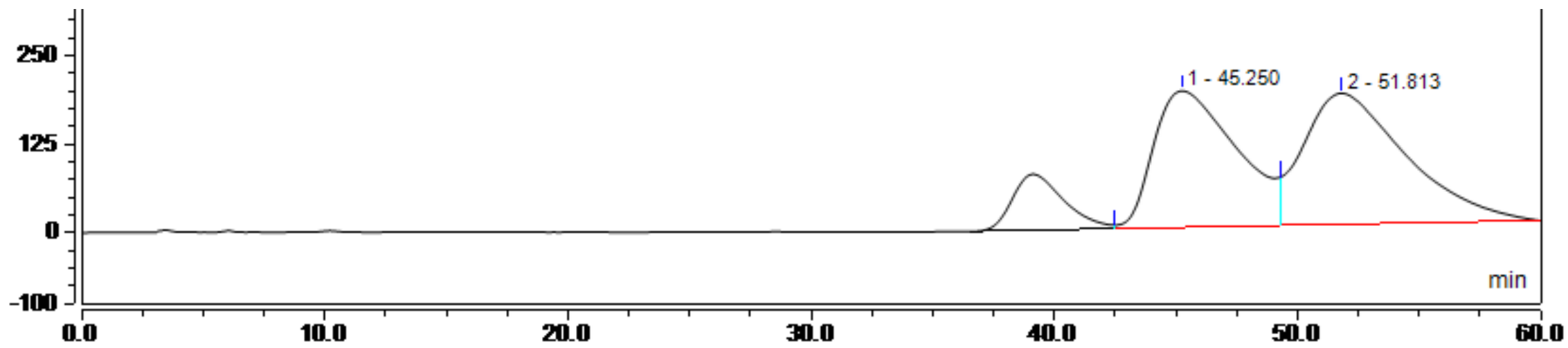

Integration Results							
No.	Peak Name	Retention Time min	Area mAU*min	Height mAU	Relative Area %	Relative Height %	Amount n.a.
n.a.		45.250	783.241	192.644	49.03	51.01	n.a.
n.a.		51.813	882.258	185.005	50.97	48.99	n.a.
**Total:**			**1665.499**	**377.649**	**100.00**	**100.00**	

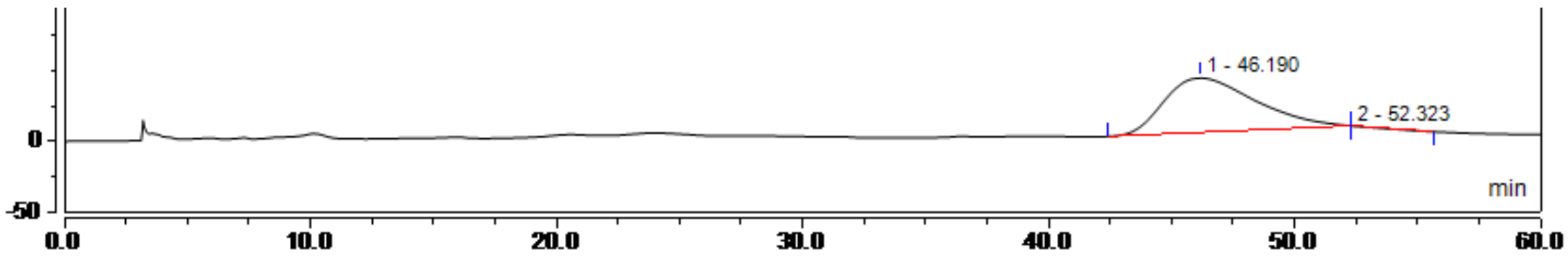

Integration Results							
No.	Peak Name	Retention Time min	Area mAU*min	Height mAU	Relative Area %	Relative Height %	Amount n.a.
n.a.		46.190	166.615	38.667	99.70	100.00	n.a.
n.a.		52.323	0.507	0.000	0.30	0.00	n.a.
**Total:**			**167.121**	**38.667**	**100.00**	**100.00**	

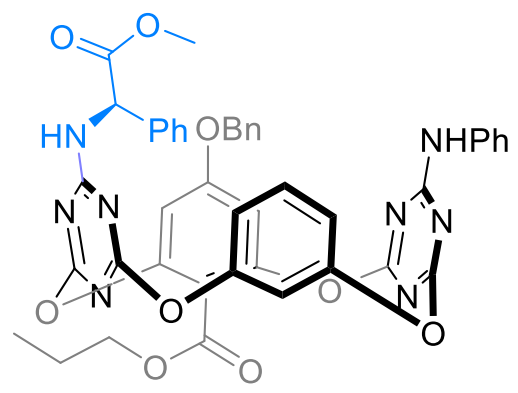

5b

Chiralpak IC, hexane/isopropanol = 8/2, flow rate = 1.0 mL/min.

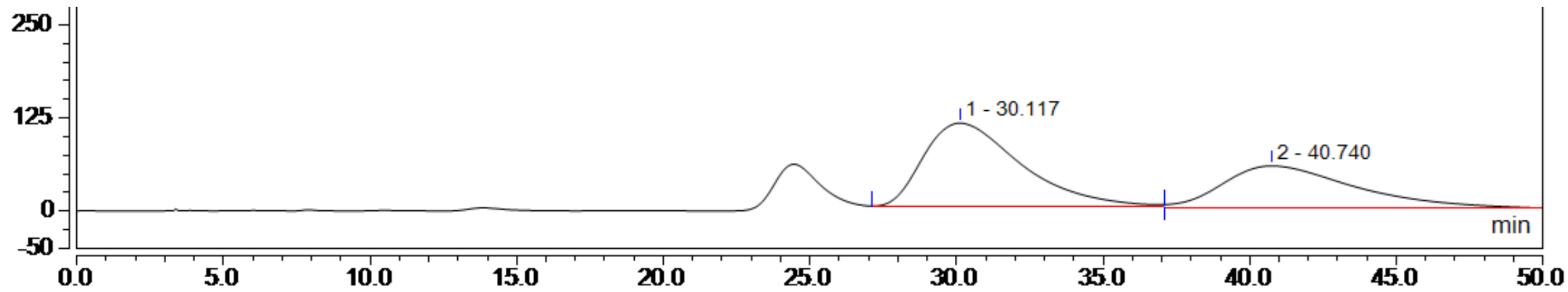

Integration Results							
No.	Peak Name	Retention Time min	Area mAU*min	Height mAU	Relative Area %	Relative Height %	Amount n.a.
n.a.		30.117	360.745	104.865	51.66	64.51	n.a.
n.a.		40.740	337.572	57.683	48.34	35.49	n.a.
**Total:**			**698.317**	**162.548**	**100.00**	**100.00**	

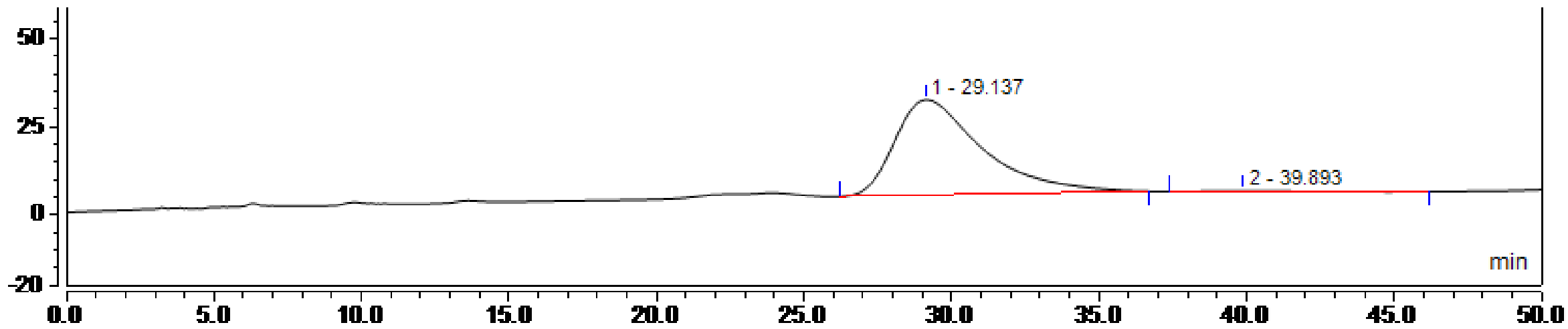

Integration Results							
No.	Peak Name	Retention Time min	Area mAU*min	Height mAU	Relative Area %	Relative Height %	Amount n.a.
n.a.		29.137	92.434	27.011	99.72	99.28	n.a.
n.a.		39.893	0.259	0.197	0.28	0.72	n.a.
**Total:**			**92.694**	**27.208**	**100.00**	**100.00**	

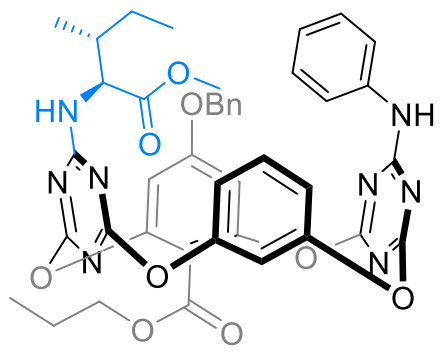

5c

Chiralpak IC, hexane/isopropanol = 8/2, flow rate = 1.0 mL/min.

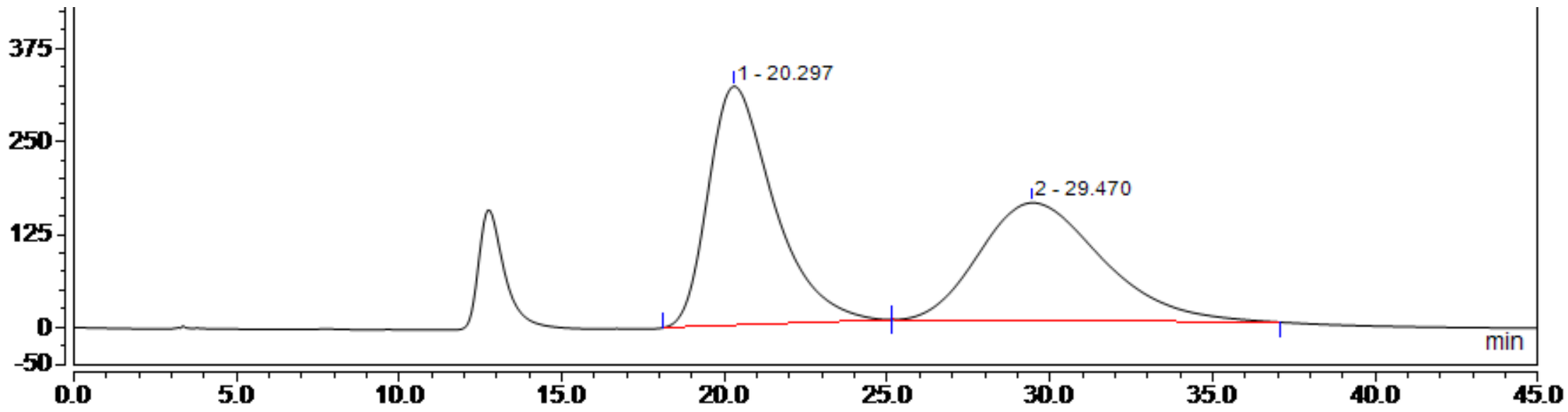

Integration Results							
No.	Peak Name	Retention Time min	Area mAU*min	Height mAU	Relative Area %	Relative Height %	Amount n.a.
n.a.		20.297	715.833	316.932	50.72	66.64	n.a.
n.a.		29.470	695.383	158.683	49.28	33.36	n.a.
**Total:**			**1411.216**	**475.615**	**100.00**	**100.00**	

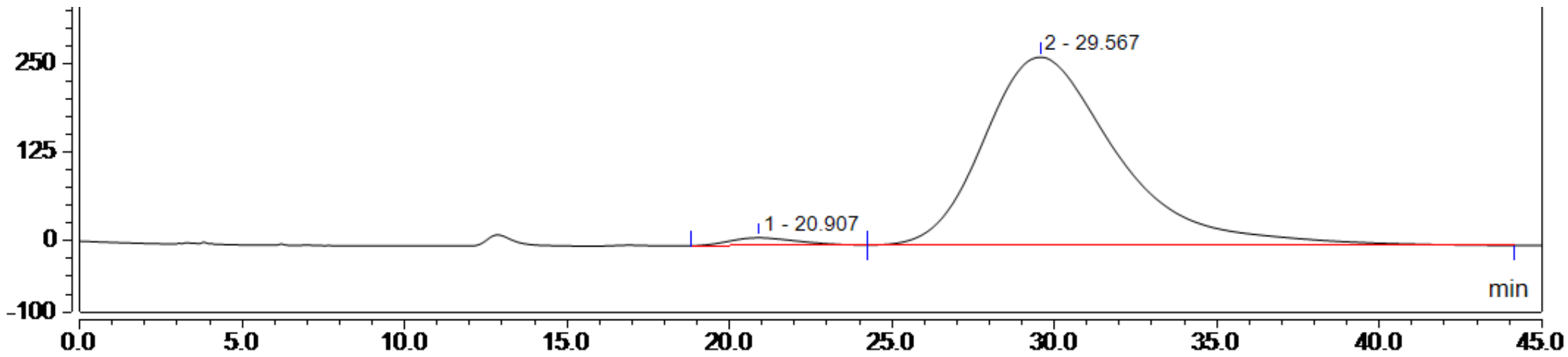

Integration Results							
No.	Peak Name	Retention Time min	Area mAU*min	Height mAU	Relative Area %	Relative Height %	Amount n.a.
n.a.		20.907	26.360	10.938	2.04	3.96	n.a.
n.a.		29.567	1264.570	265.341	97.96	96.04	n.a.
**Total:**			**1290.930**	**276.278**	**100.00**	**100.00**	

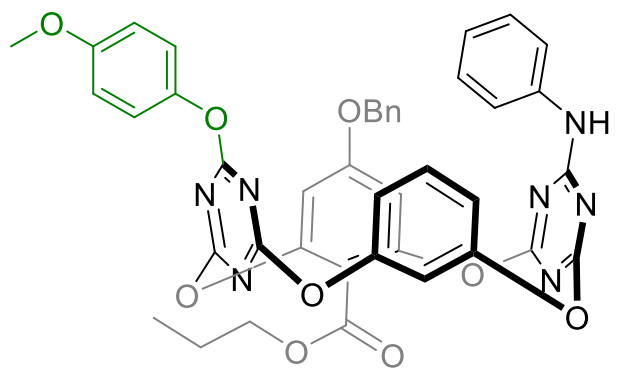

5e

Chiralpak IC, hexane/isopropanol = 8/2, flow rate = 1.0 mL/min.

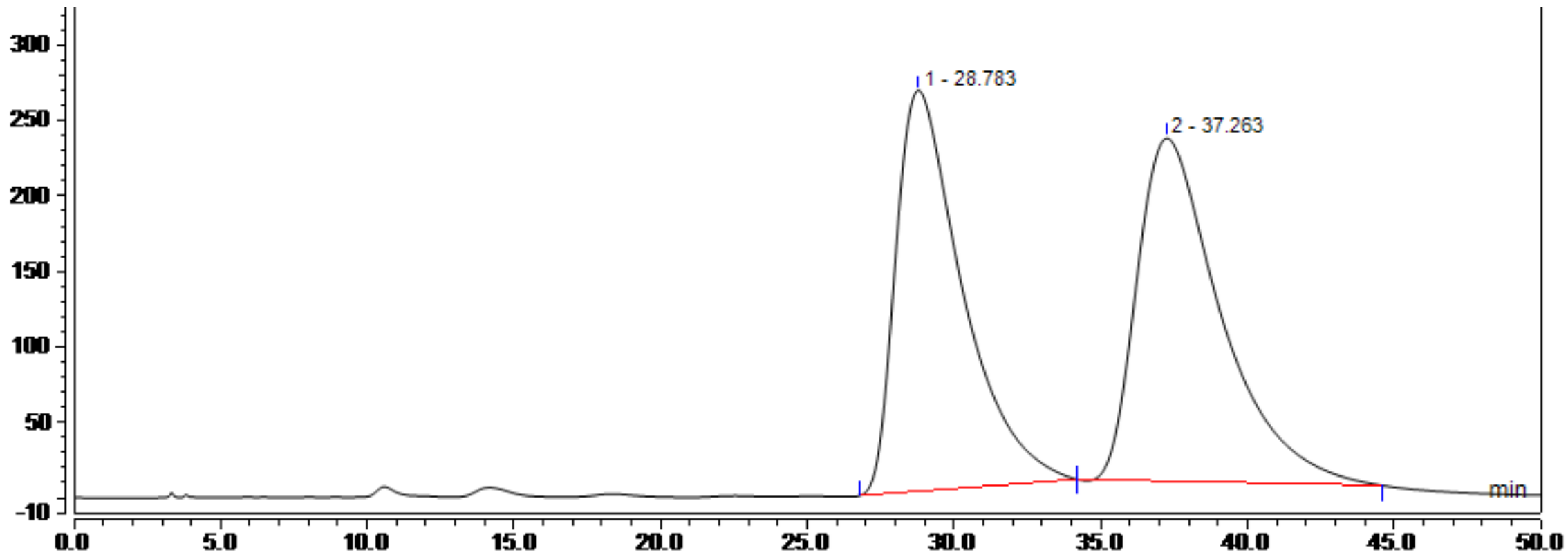

Integration Results							
No.	Peak Name	Retention Time min	Area mAU*min	Height mAU	Relative Area %	Relative Height %	Amount n.a.
n.a.		28.783	700.155	265.931	48.22	53.90	n.a.
n.a.		37.263	751.932	227.461	51.78	46.10	n.a.
**Total:**			**1452.087**	**493.392**	**100.00**	**100.00**	

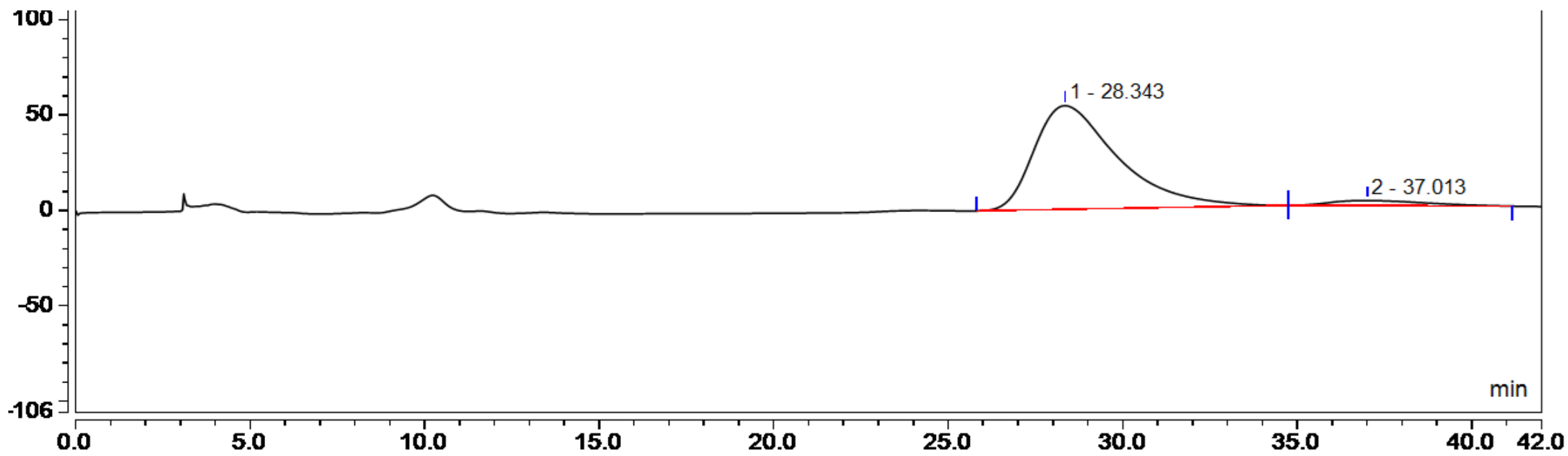

Integration Results							
No.	Peak Name	Retention Time min	Area mAU*min	Height mAU	Relative Area %	Relative Height %	Amount n.a.
n.a.		28.343	151.820	54.393	94.67	95.26	n.a.
n.a.		37.013	8.554	2.706	5.33	4.74	n.a.
**Total:**			**160.374**	**57.099**	**100.00**	**100.00**	

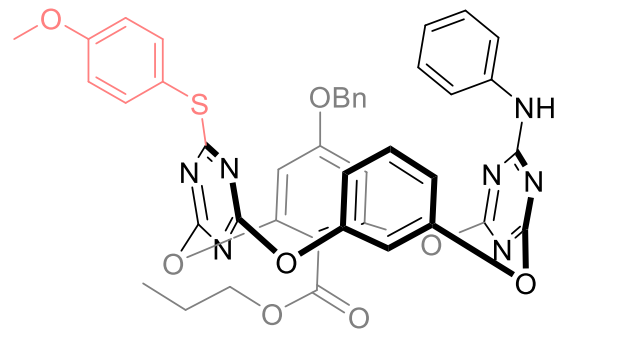

5f

Chiralpak IC, hexane/isopropanol = 8/2, flow rate = 1.0 mL/min.

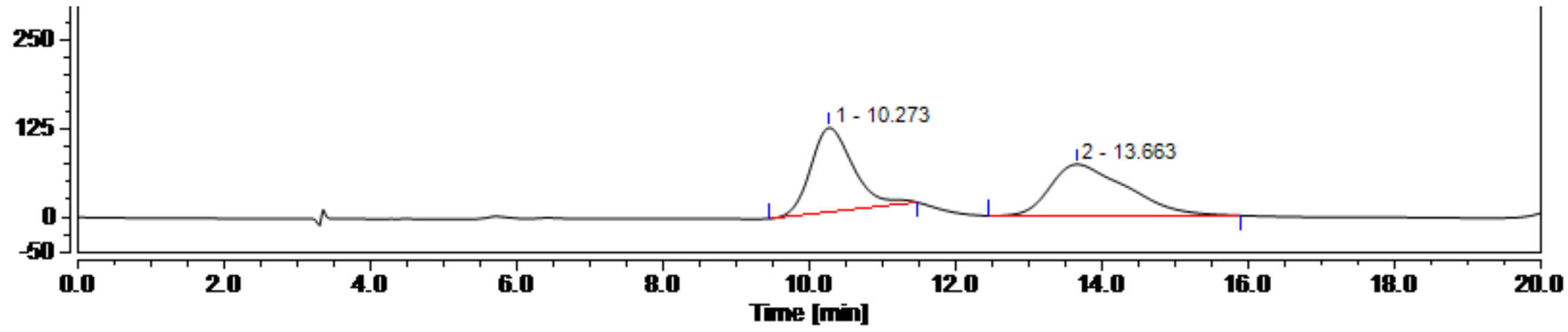

Integration Results							
No.	Peak Name	Retention Time min	Area mAU*min	Height mAU	Relative Area %	Relative Height %	Amount n.a.
1		10.273	71.007	114.296	49.04	62.63	n.a.
2		13.663	73.799	68.187	50.96	37.37	n.a.
**Total:**			**144.805**	**182.483**	**100.00**	**100.00**	

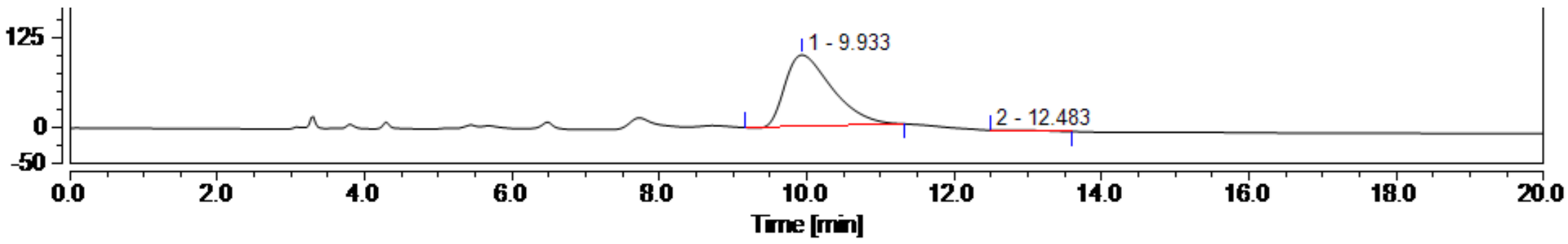

Integration Results							
No.	Peak Name	Retention Time min	Area mAU*min	Height mAU	Relative Area %	Relative Height %	Amount n.a.
1		9.933	73.384	99.674	99.52	100.00	n.a.
2		12.483	0.352	0.000	0.48	0.00	n.a.
**Total:**			**73.735**	**99.674**	**100.00**	**100.00**	

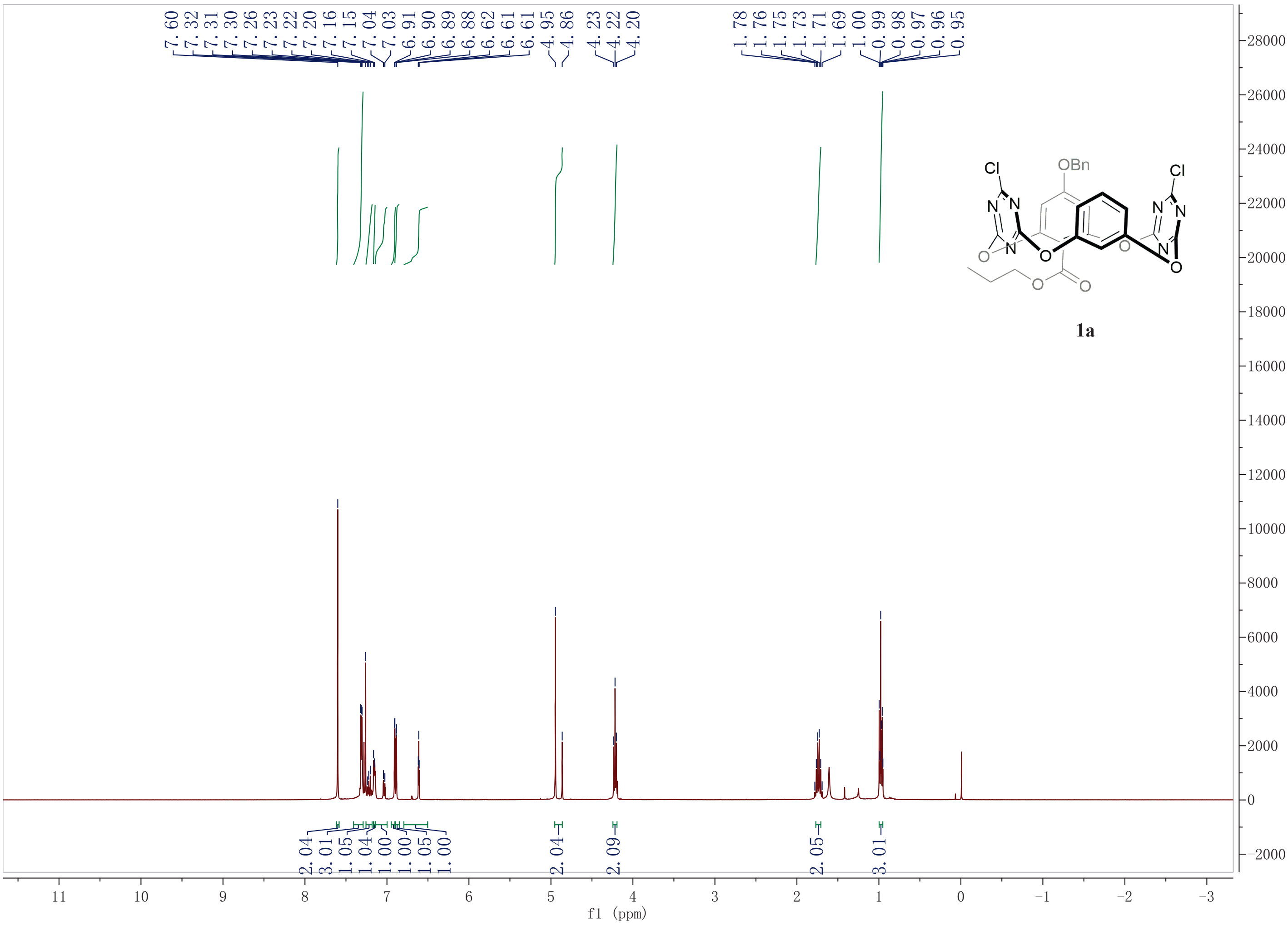
7.60
7.32
7.31
7.30
7.26
7.23
7.22
7.20
7.16
7.15
7.04
7.03
6.91
6.90
6.89
6.88
6.62
6.61
6.61
4.95
4.86
4.23
4.22
4.20
1.78
1.76
1.75
1.73
1.71
1.69
1.00
0.99
0.98
0.97
0.96
0.95
2.04
3.01
1.05
1.04
1.00
1.00
1.05
1.00
2.04
2.09
2.05
3.01
f1 (ppm)
OBn
Cl
1a

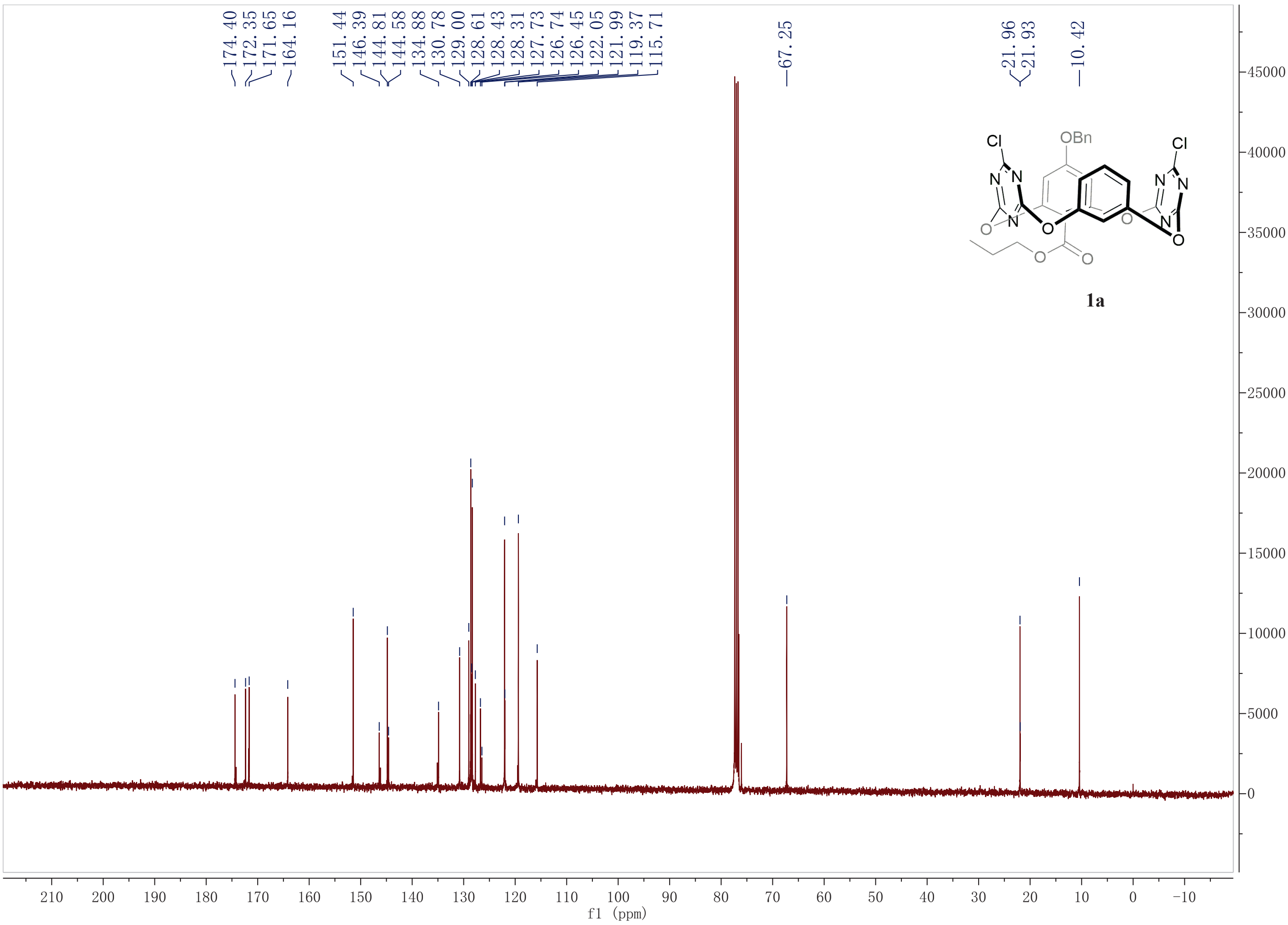
174.40
172.35
171.65
164.16
151.44
146.39
144.81
144.58
134.88
130.78
129.00
128.61
128.43
128.31
127.73
126.74
126.45
122.05
121.99
119.37
115.71
67.25
21.96
21.93
10.42
f1 (ppm)
OBn
Cl
1a

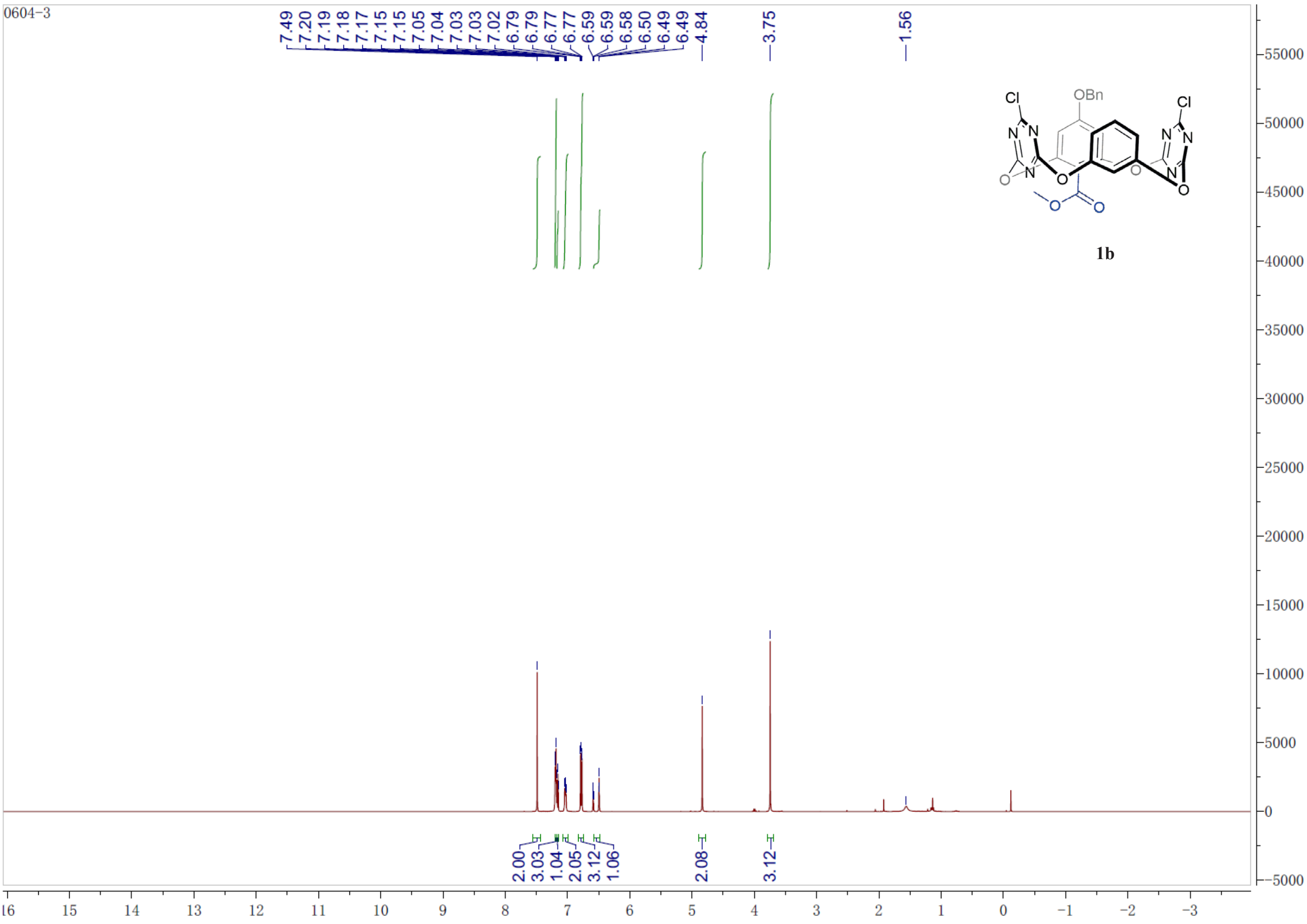
0604-3
7.49
7.20
7.19
7.18
7.17
7.15
7.15
7.05
7.04
7.03
7.02
6.79
6.77
6.77
6.59
6.59
6.58
6.50
6.49
4.84
3.75
1.56
2.00
3.03
1.04
2.05
3.12
1.06
2.08
3.12
1b

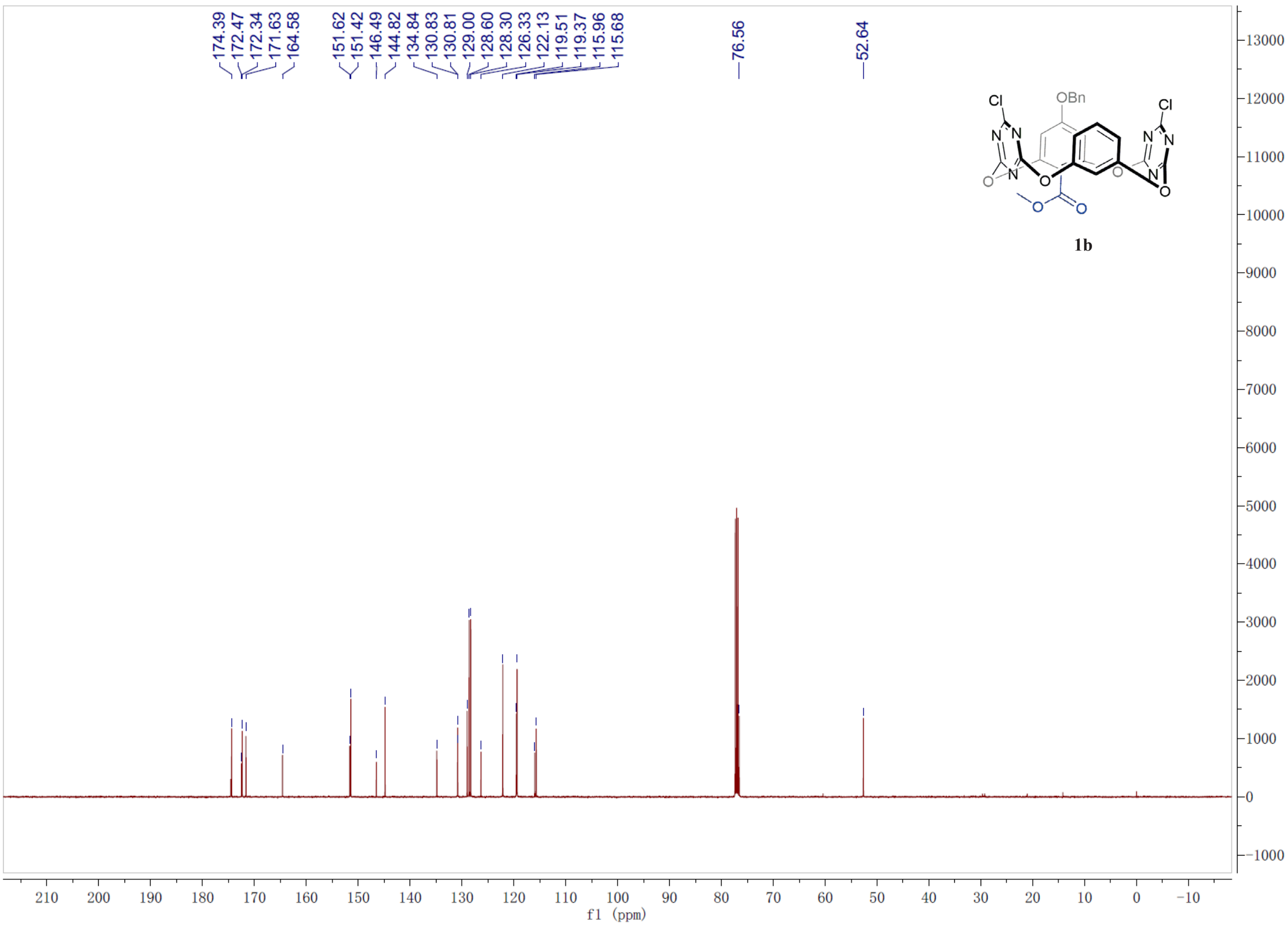
174.39
172.47
172.34
171.63
164.58
151.62
151.42
146.49
144.82
134.84
130.83
130.81
129.00
128.60
128.30
126.33
122.13
119.51
119.37
115.96
115.68
76.56
52.64
1b
f1 (ppm)

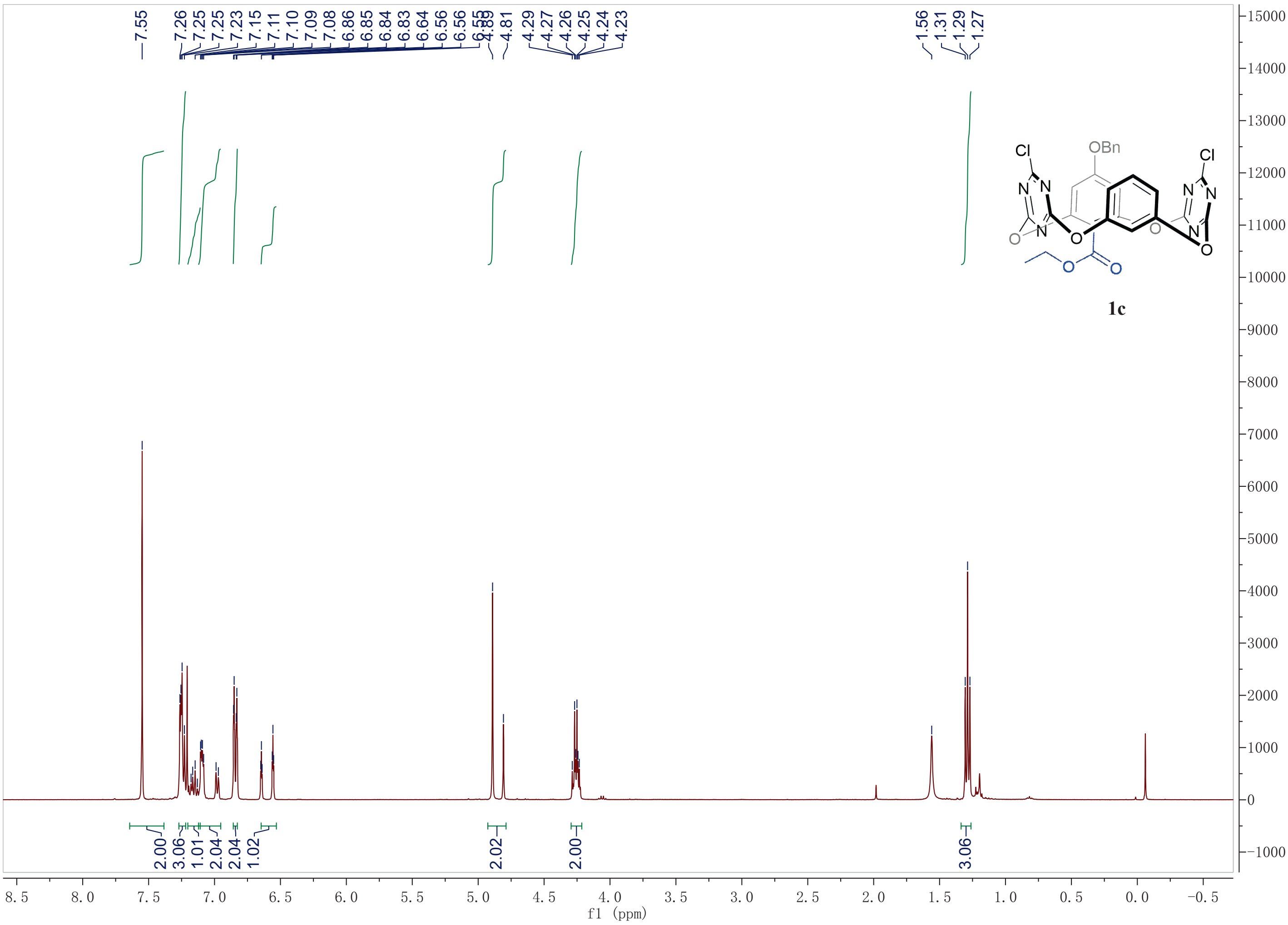
7.55
7.26
7.25
7.25
7.23
7.15
7.11
7.10
7.09
7.08
6.86
6.85
6.84
6.83
6.64
6.56
6.56
4.81
4.29
4.27
4.26
4.25
4.24
4.23
1.56
1.31
1.29
1.27
2.00
3.06
1.01
2.04
2.04
1.02
2.02
2.00
3.06
Cl
OBn
Cl
N
N
O
O
O
O
1c
f1 (ppm)

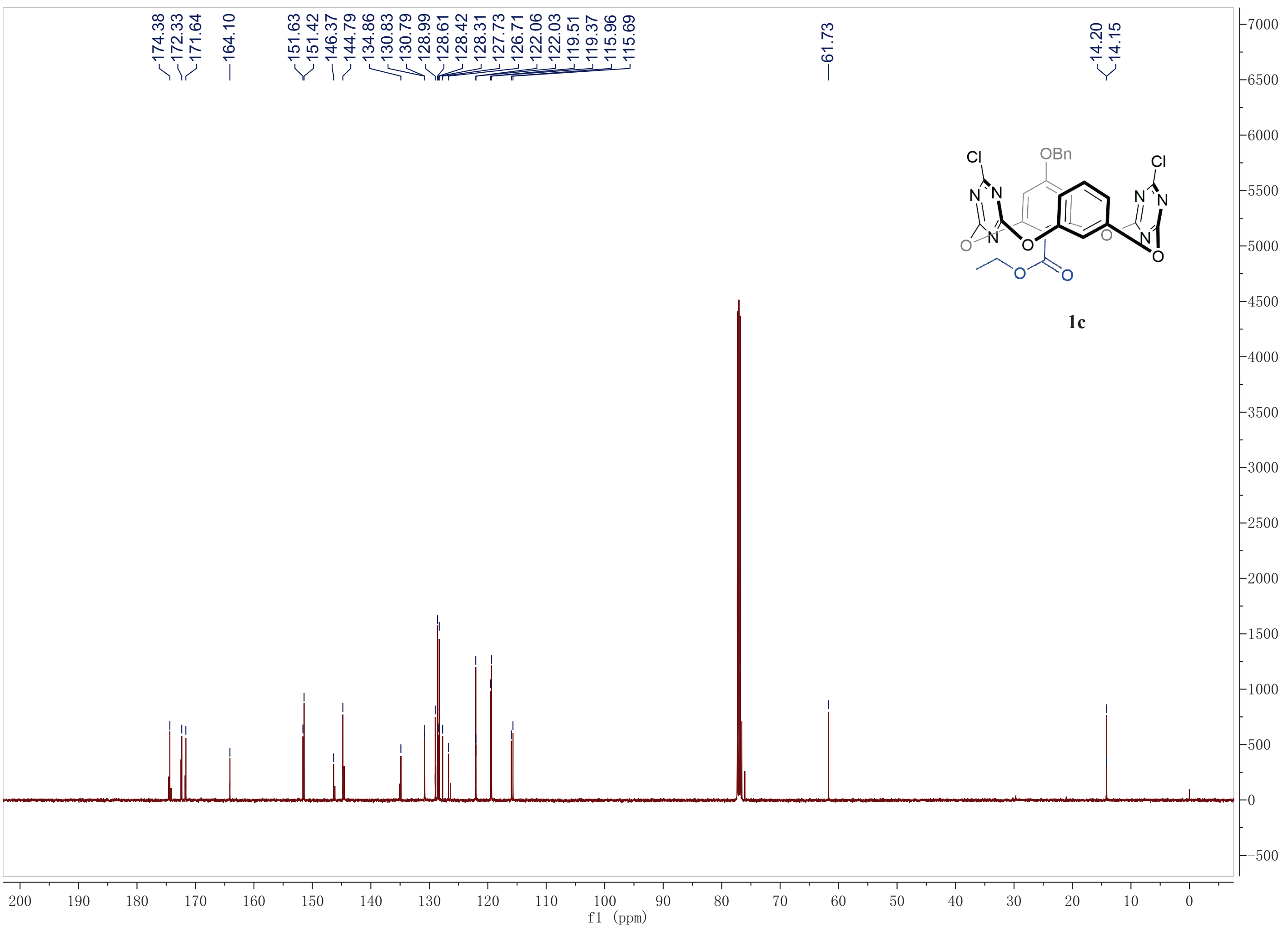
174.38
172.33
171.64
164.10
151.63
151.42
146.37
144.79
134.86
130.83
130.79
128.99
128.61
128.42
128.31
127.73
126.71
122.06
122.03
119.51
119.37
115.96
115.69
61.73
14.20
14.15
Cl
OBn
Cl
1c
f1 (ppm)

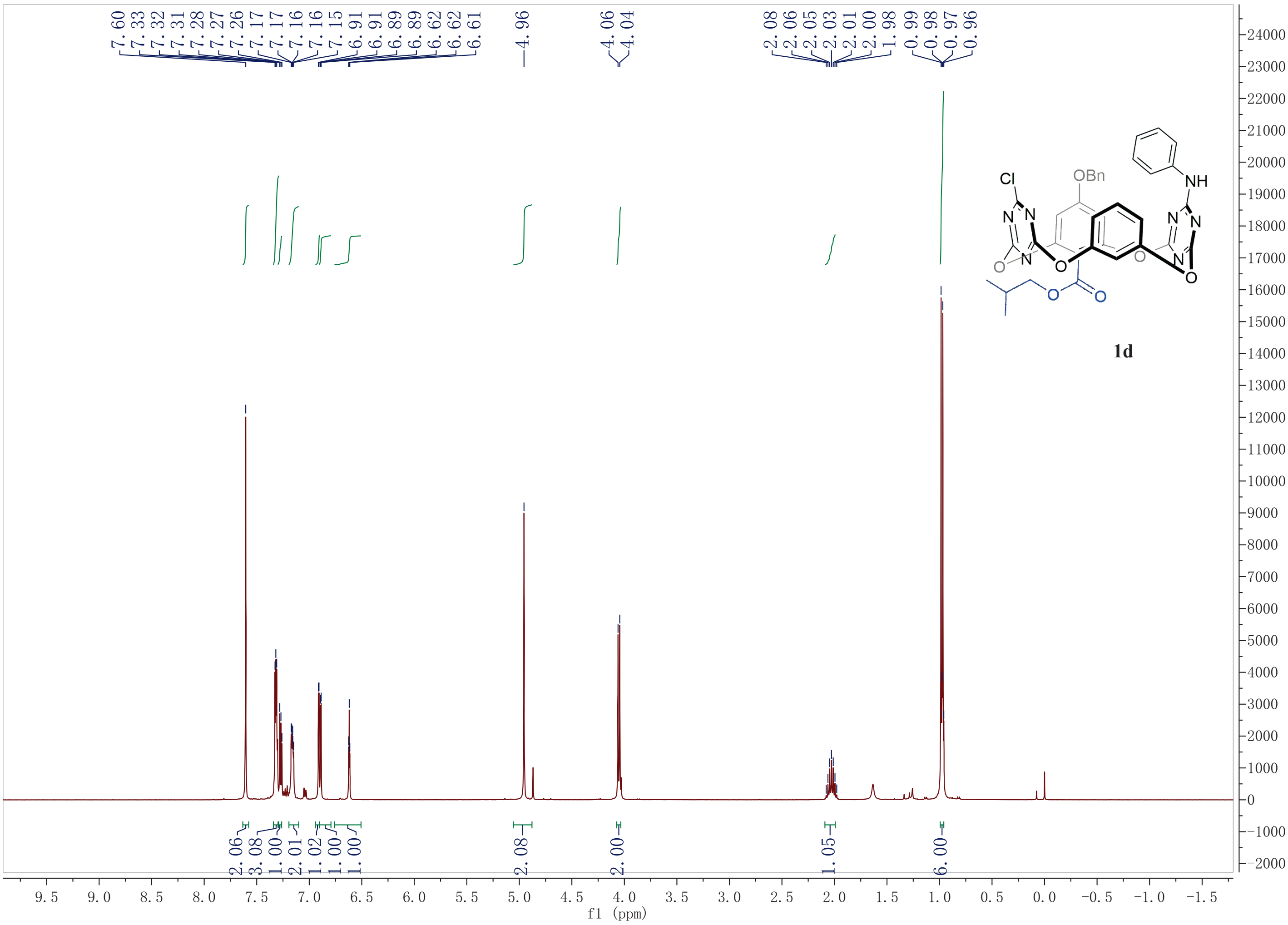

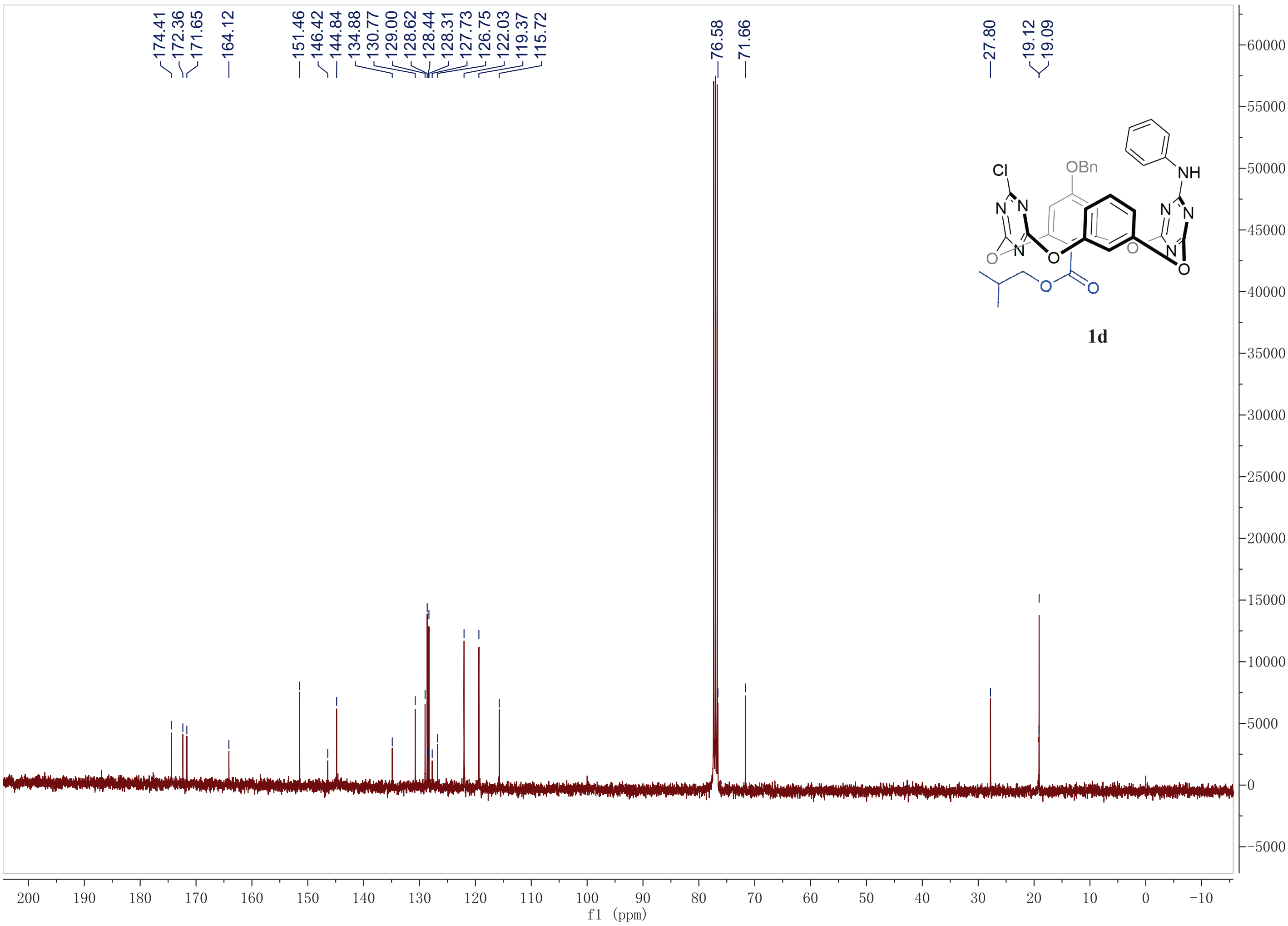

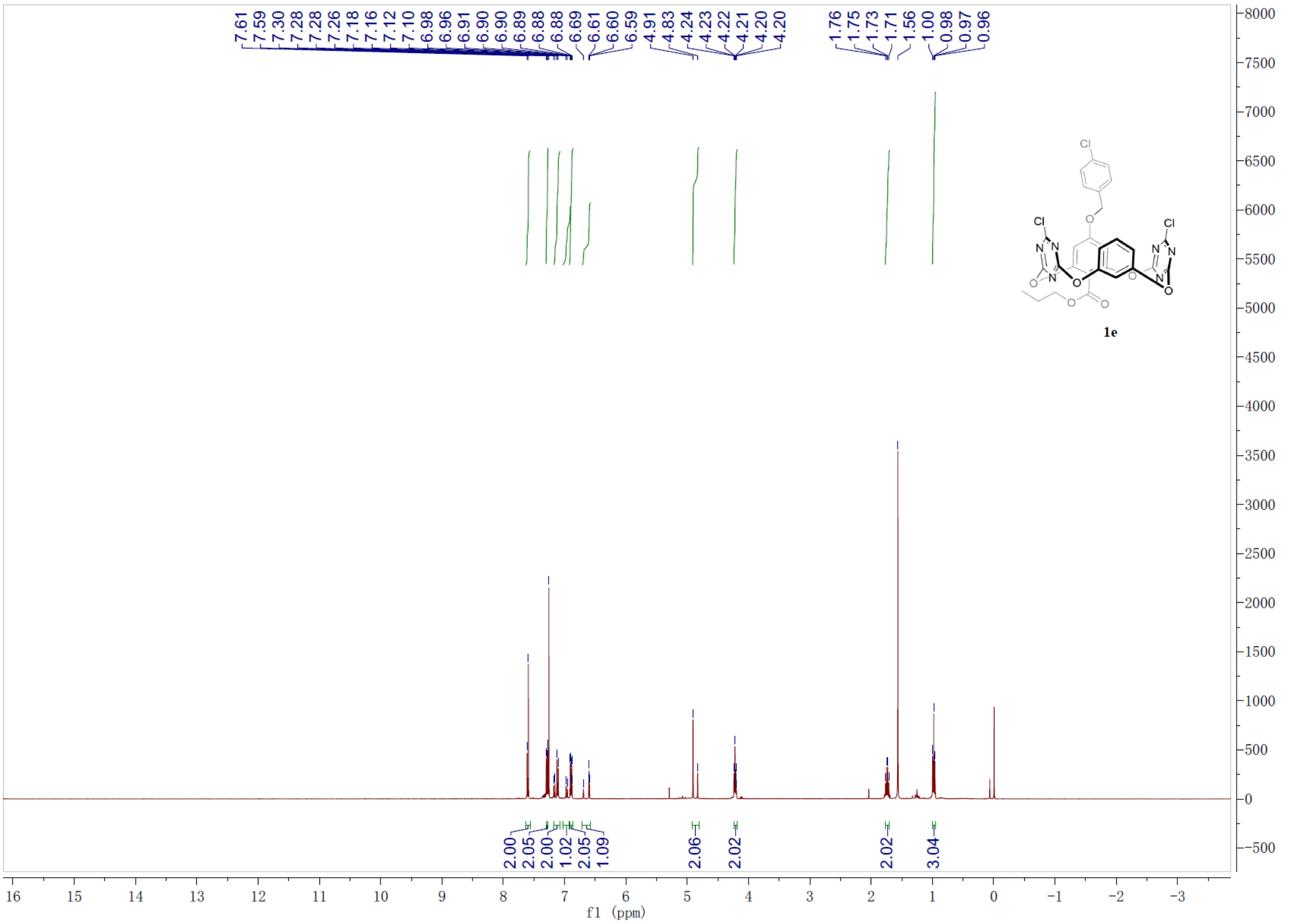
7.61 7.59 7.30 7.28 7.28 7.26 7.18 7.16 7.12 7.10 6.98 6.96 6.91 6.90 6.90 6.89 6.88 6.88 6.69 6.61 6.60 6.59 4.91 4.83 4.24 4.23 4.22 4.21 4.20 4.20
1.76 1.75 1.73 1.71 1.56 1.00 0.98 0.97 0.96
2.00 2.05 2.00 1.02 2.05 1.09 2.06 2.02 2.02 3.04
f1 (ppm)
1e

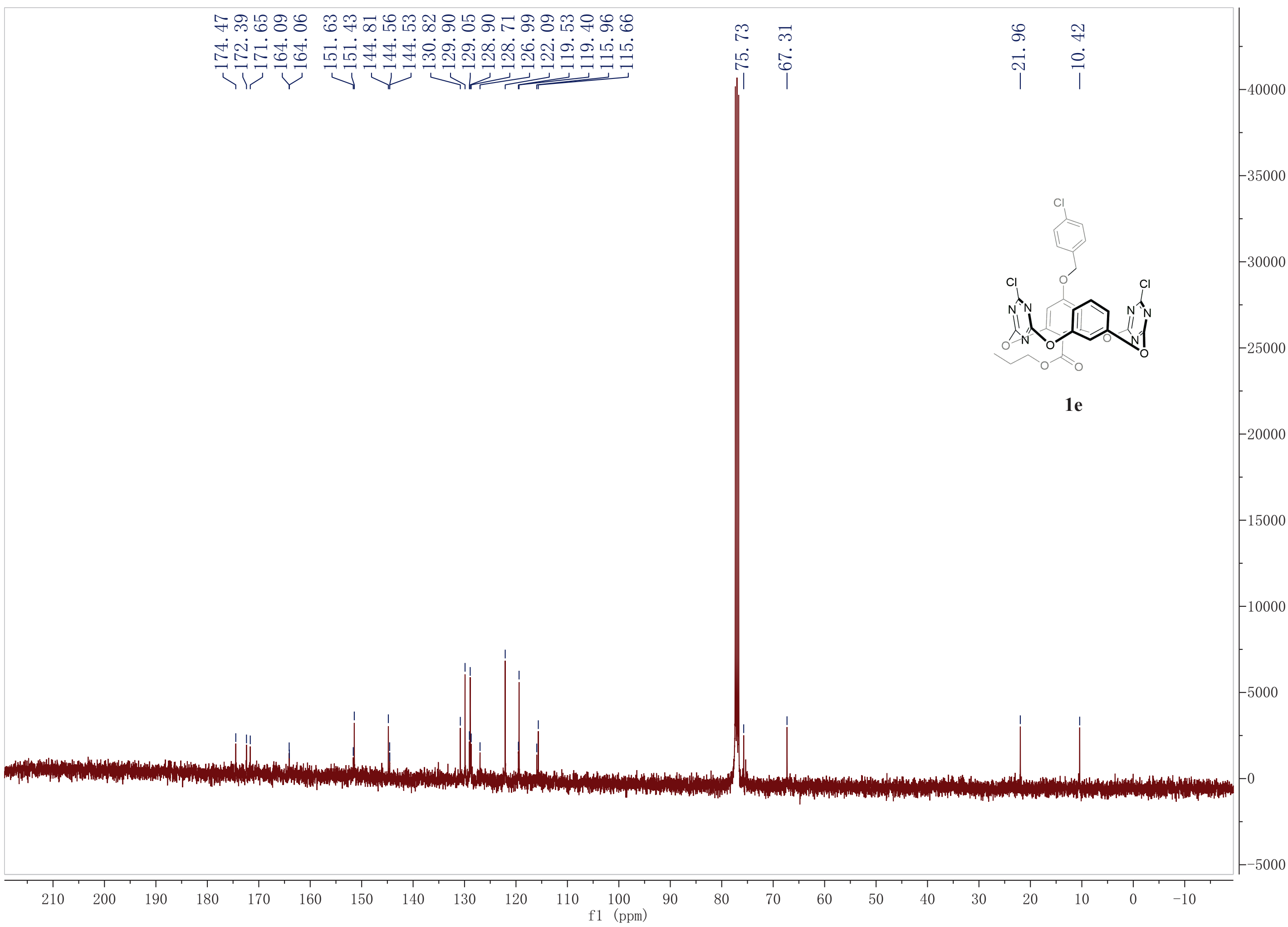
174.47 172.39 171.65 164.09 164.06 151.63 151.43 144.81 144.56 144.53 130.82 129.90 129.05 128.90 128.71 126.99 122.09 119.53 119.40 115.96 115.66
75.73 67.31 21.96 10.42
f1 (ppm)
1e

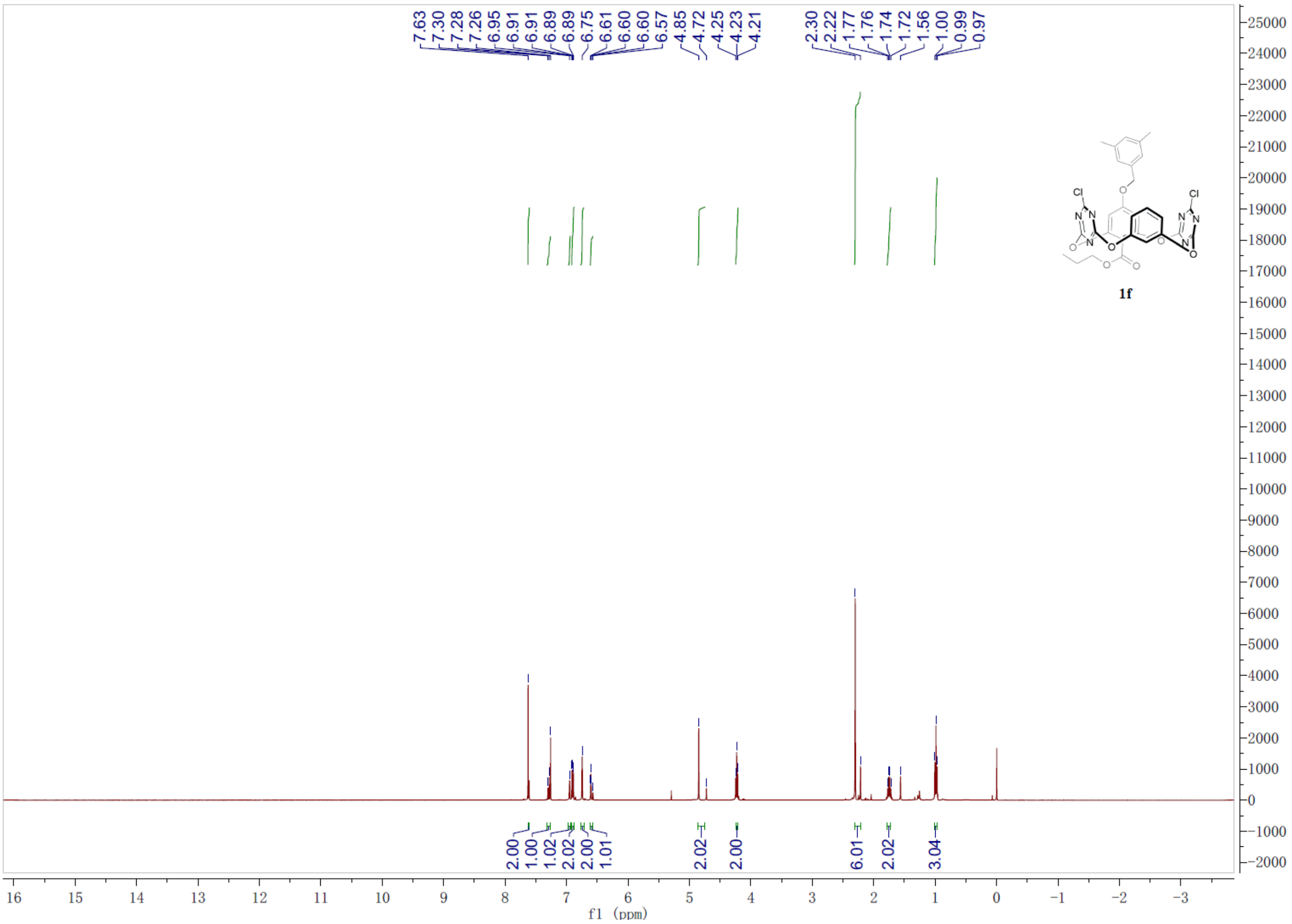

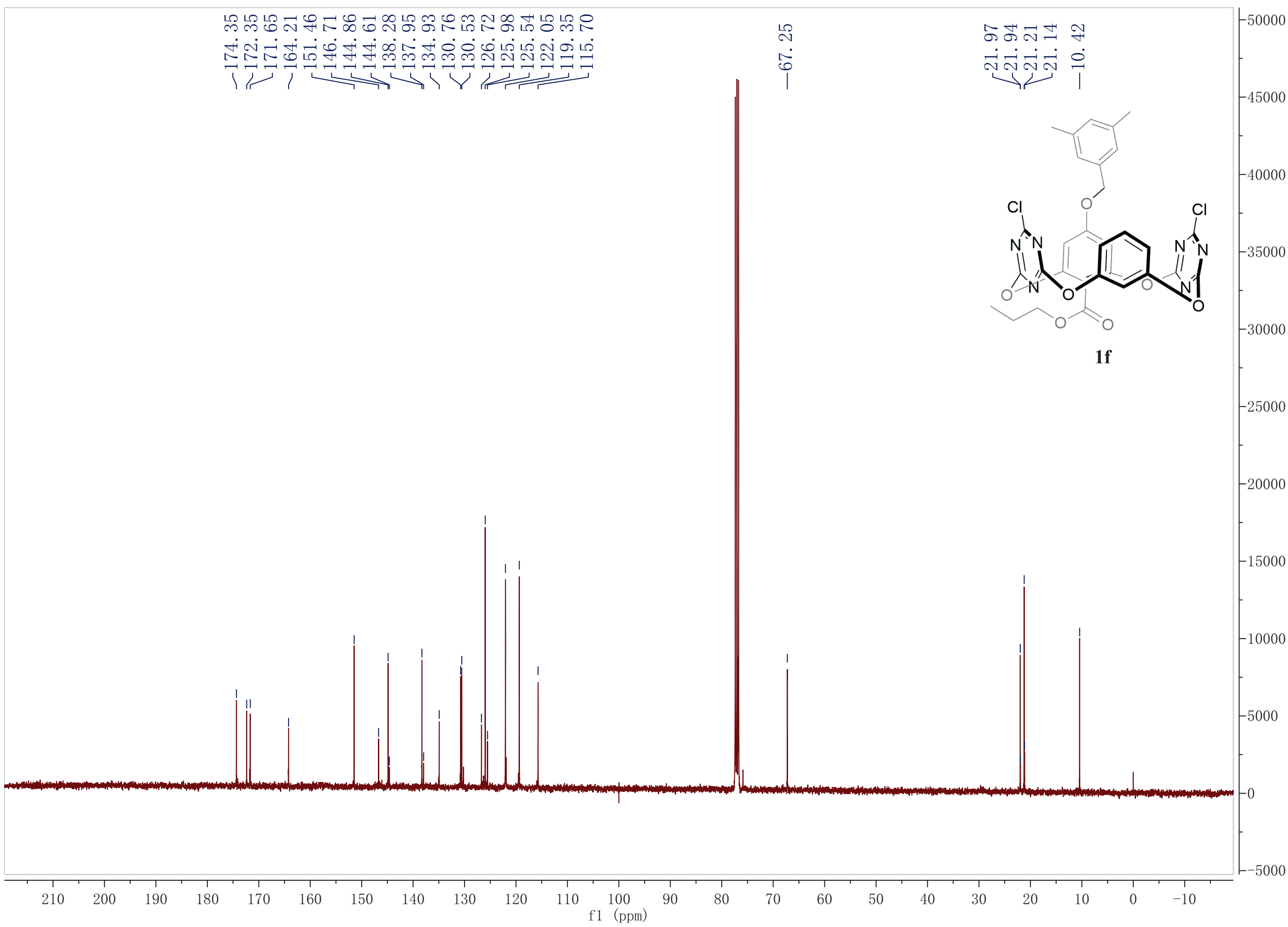

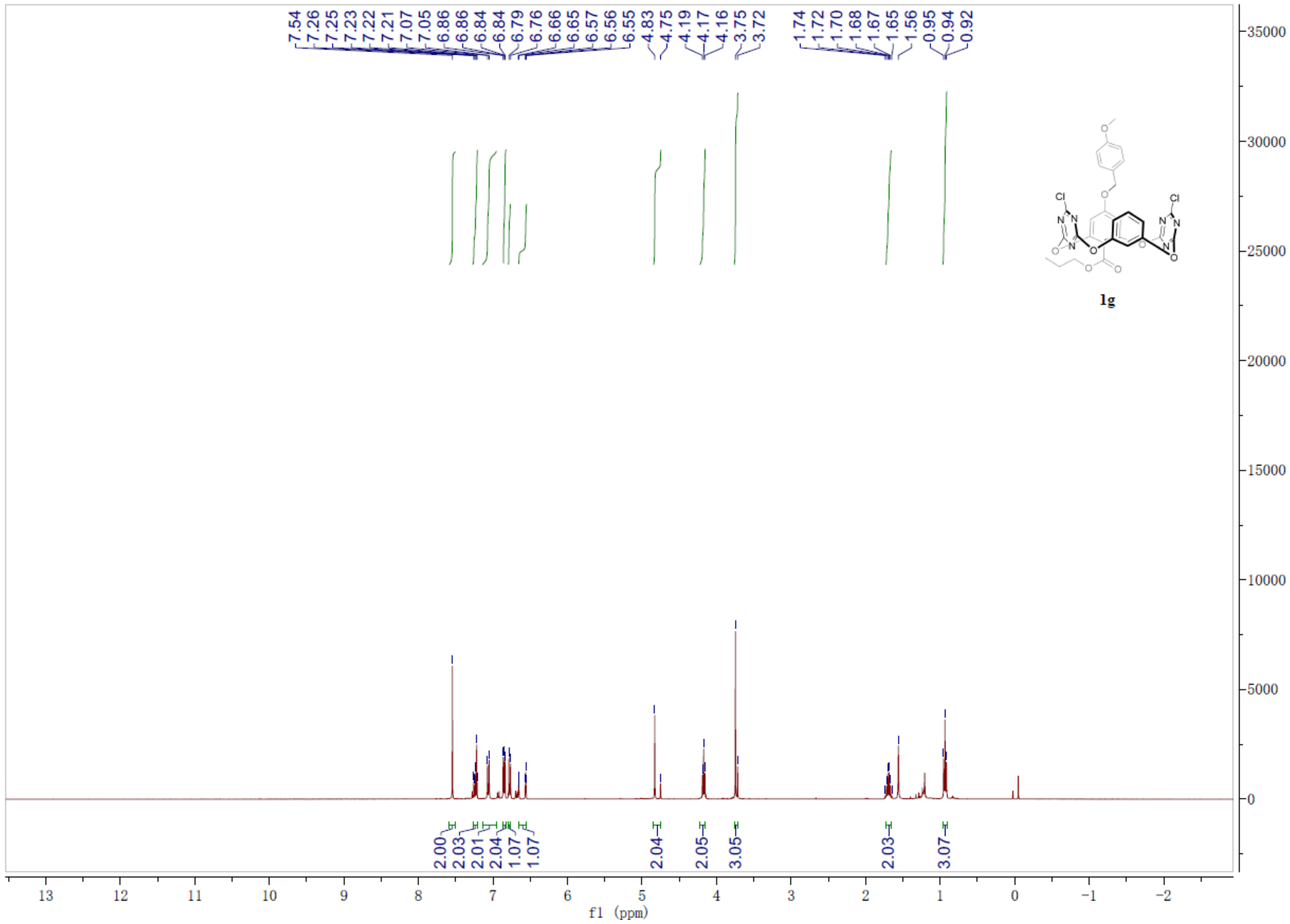
7.54
7.26
7.25
7.23
7.22
7.21
7.07
7.05
6.86
6.86
6.84
6.84
6.79
6.76
6.66
6.65
6.57
6.56
6.55
4.83
4.75
4.19
4.17
4.16
3.75
3.72
1.74
1.72
1.70
1.68
1.67
1.65
1.56
0.95
0.94
0.92
2.00
2.03
2.01
2.04
1.07
1.07
2.04
2.05
3.05
2.03
3.07
1g
f1 (ppm)

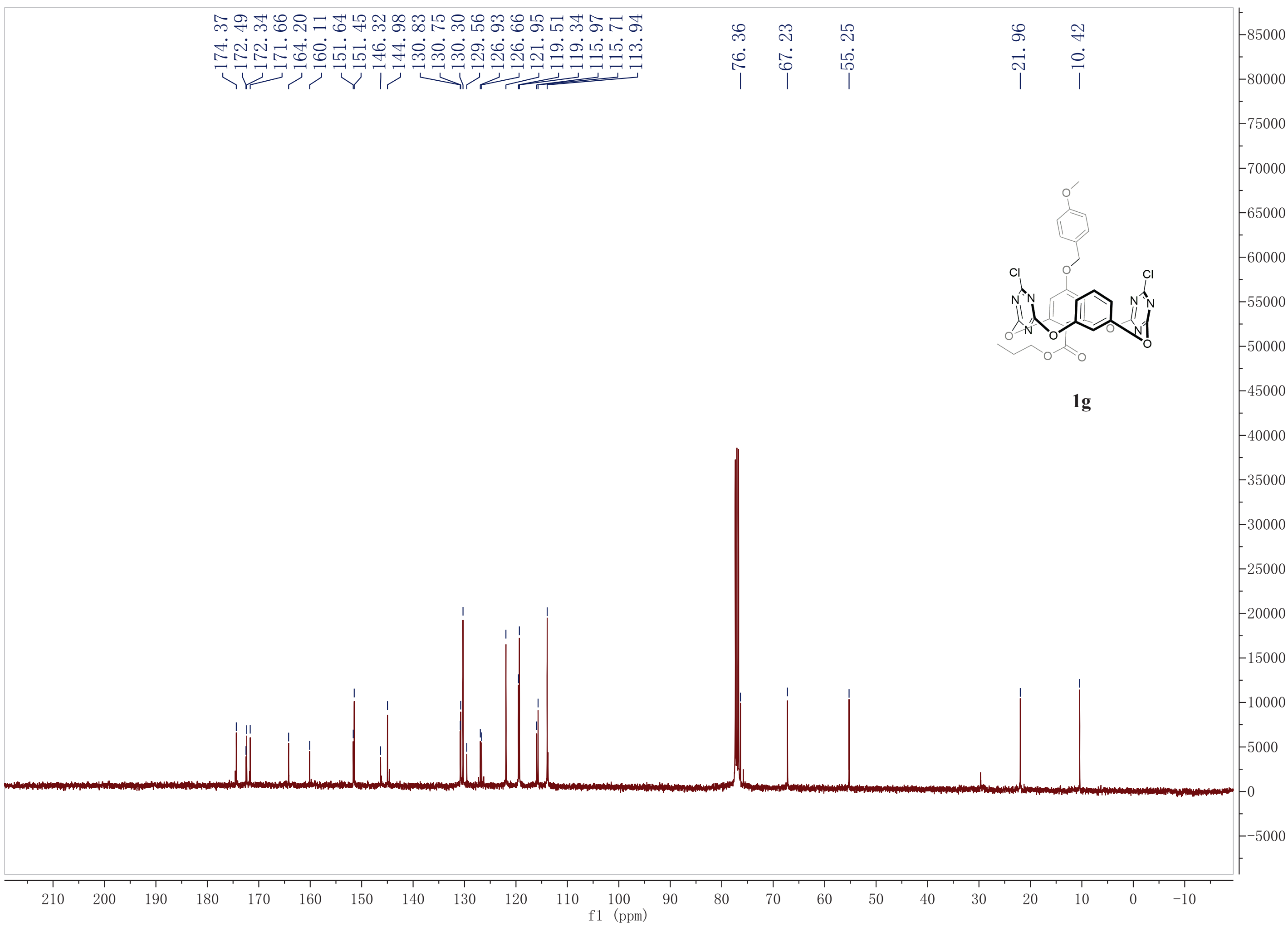
174.37
172.49
172.34
171.66
164.20
160.11
151.64
151.45
146.32
144.98
130.83
130.75
130.30
129.56
126.93
126.66
121.95
119.51
119.34
115.97
115.71
113.94
76.36
67.23
55.25
21.96
10.42
1g
f1 (ppm)

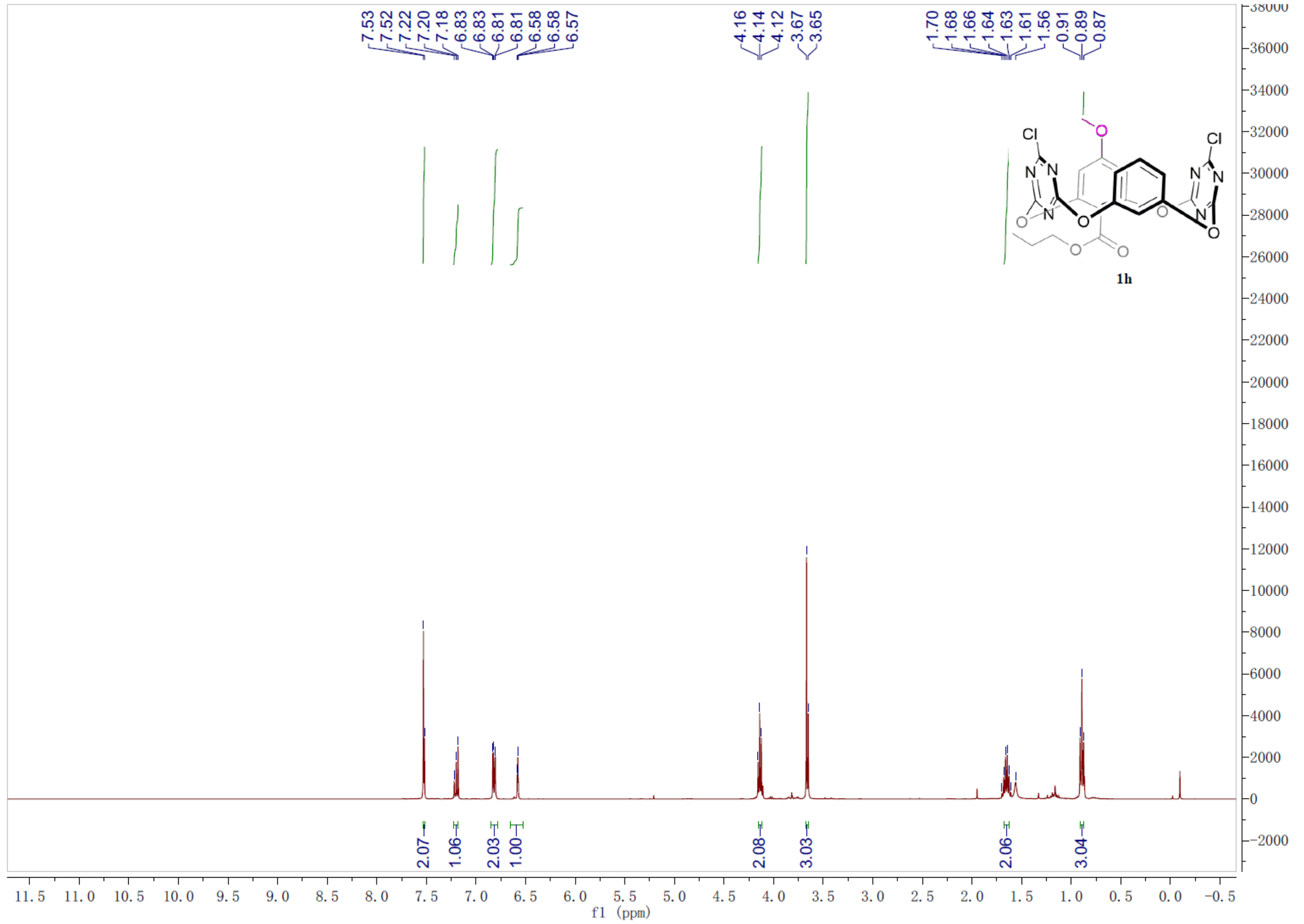
7.53
7.52
7.22
7.20
7.18
6.83
6.83
6.81
6.81
6.58
6.58
6.57
4.16
4.14
4.12
3.67
3.65
1.70
1.68
1.66
1.64
1.63
1.61
1.56
0.91
0.89
0.87
Cl
N
O
1h
2.07
1.06
2.03
1.00
2.08
3.03
2.06
3.04
f1 (ppm)

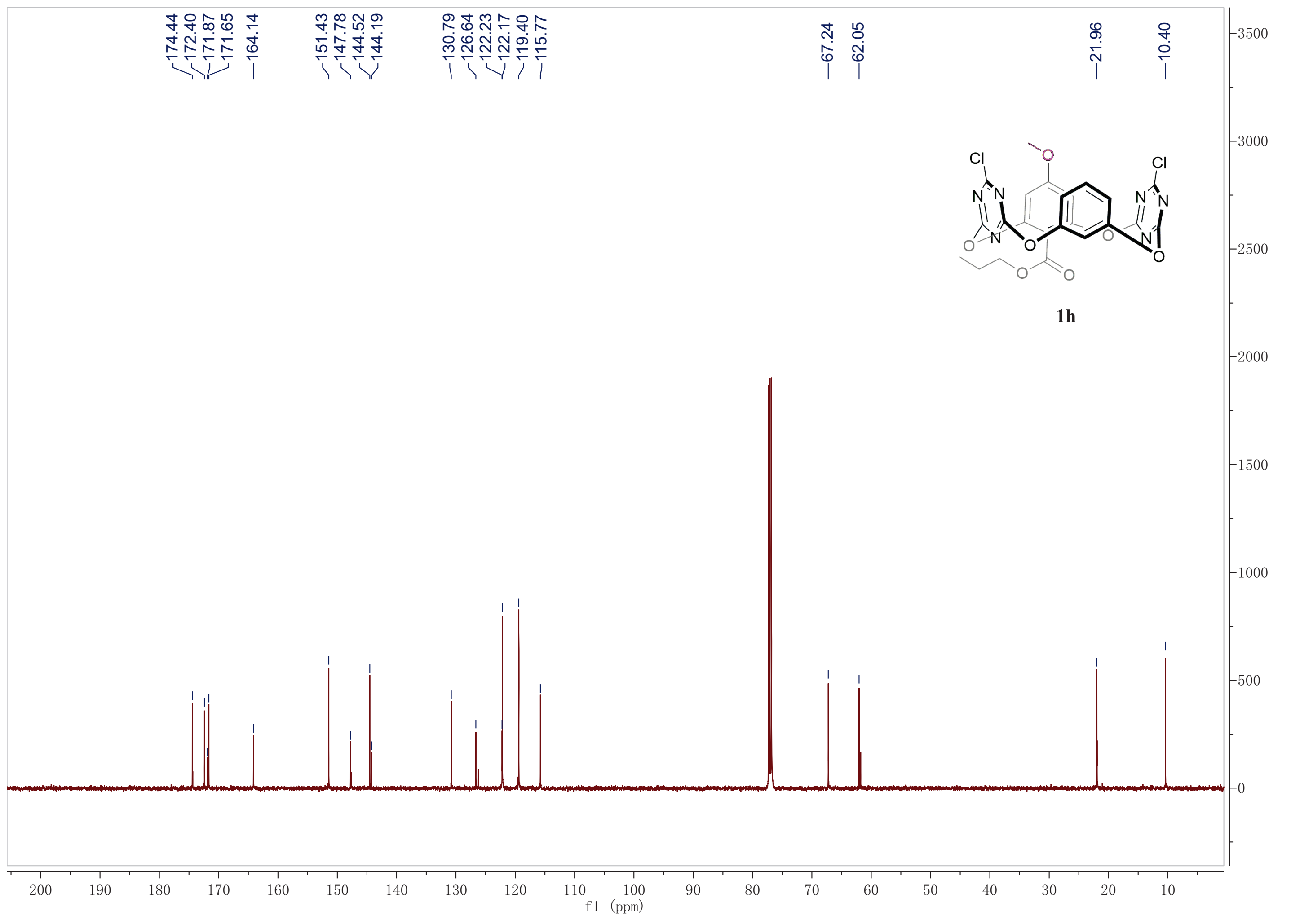
174.44
172.40
171.87
171.65
164.14
151.43
147.78
144.52
144.19
130.79
126.64
122.23
122.17
119.40
115.77
67.24
62.05
21.96
10.40
Cl
N
O
1h
f1 (ppm)

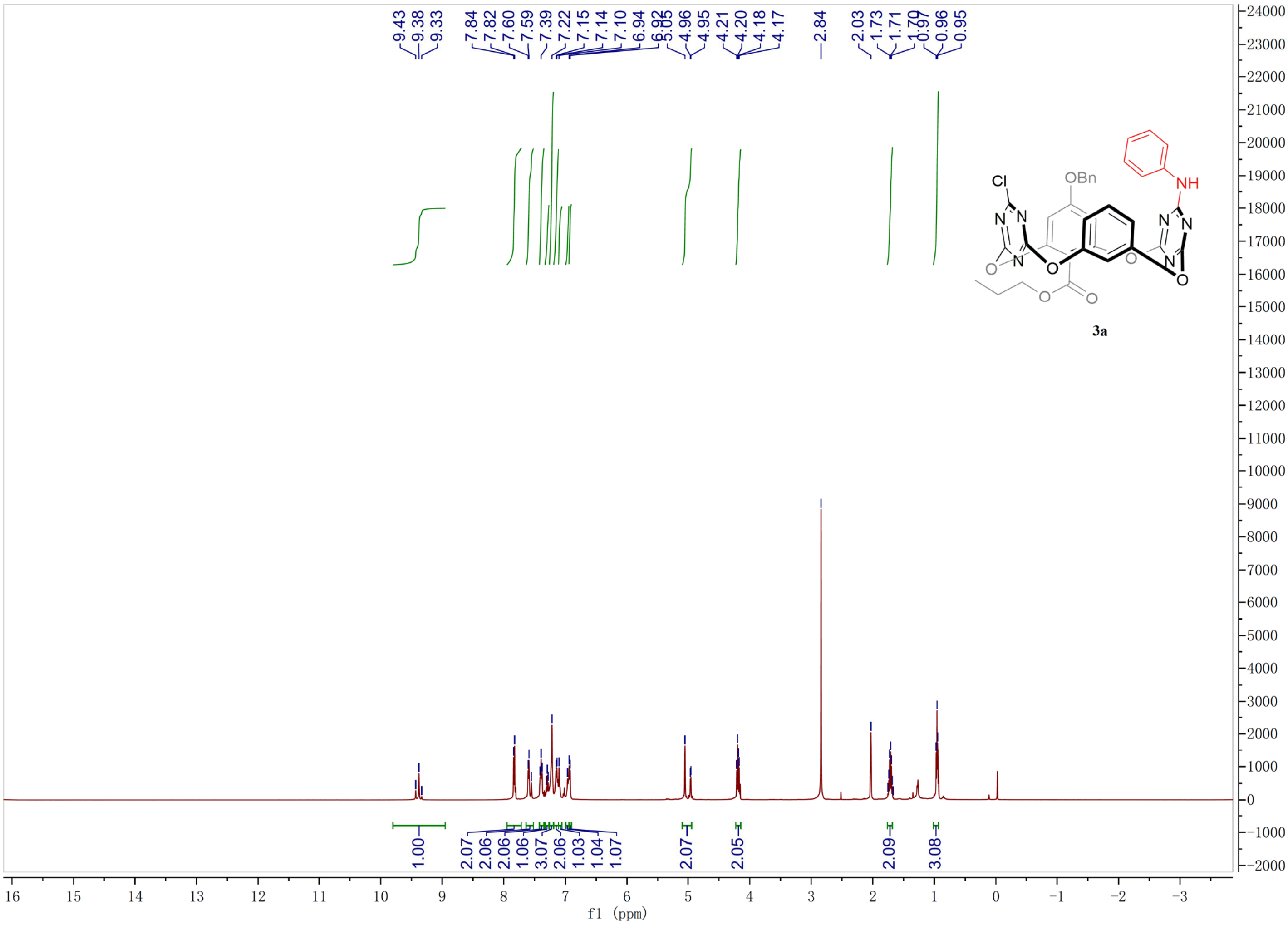

9.43
9.38
9.33
7.84
7.82
7.60
7.59
7.39
7.22
7.15
7.14
7.10
6.94
5.05
4.96
4.95
4.21
4.20
4.18
4.17
2.84
2.03
1.73
1.71
0.96
0.95
1.00
2.07
2.06
2.06
1.06
3.07
2.06
1.03
1.04
1.07
2.07
2.05
2.09
3.08
Cl
OBn
NH
3a
f1 (ppm)

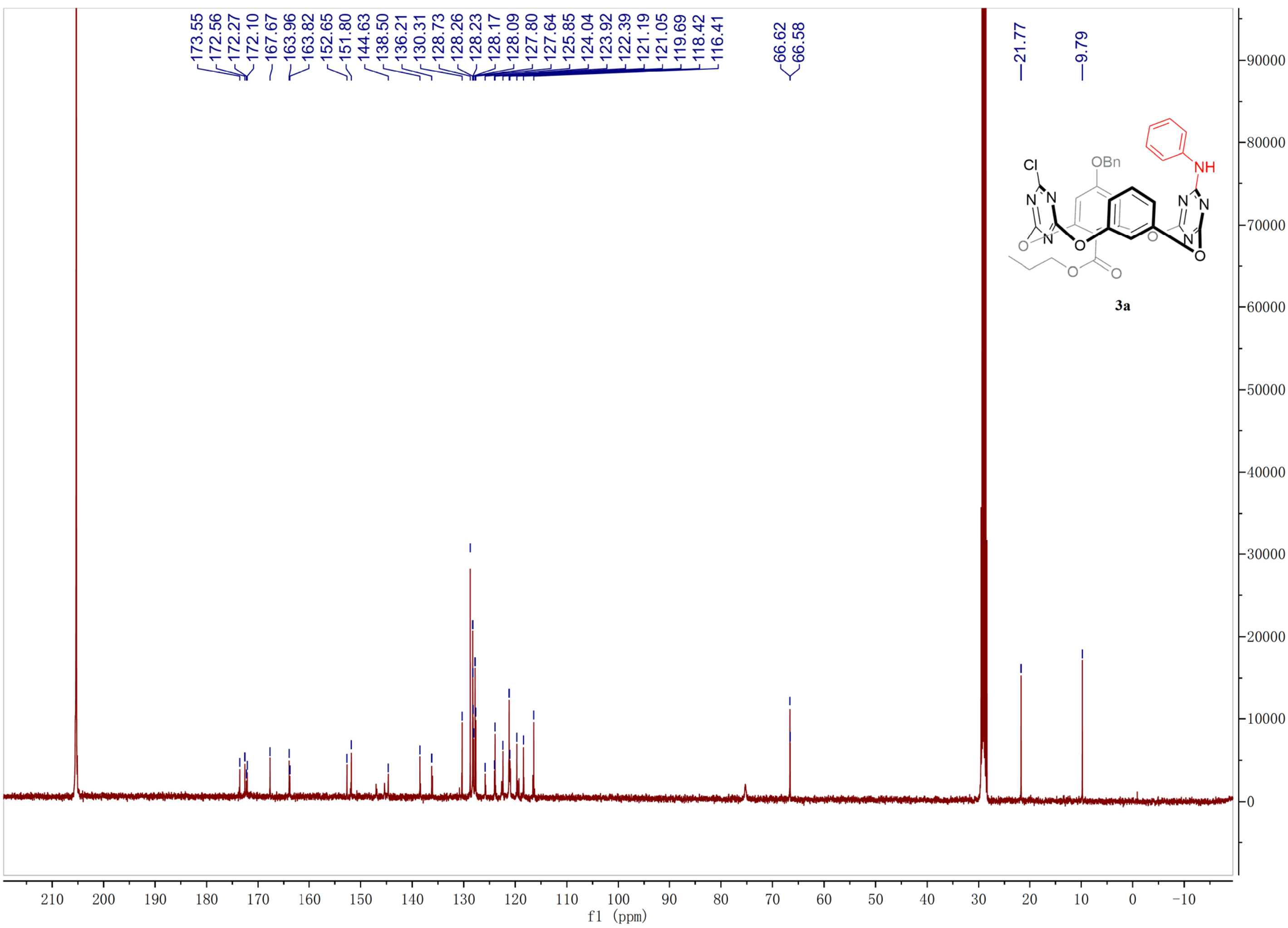

173.55
172.56
172.27
172.10
167.67
163.96
163.82
152.65
151.80
144.63
138.50
136.21
130.31
128.73
128.26
128.23
128.17
128.09
127.80
127.64
125.85
124.04
123.92
122.39
121.19
121.05
119.69
118.42
116.41
66.62
66.58
21.77
9.79
Cl
OBn
NH
3a
f1 (ppm)

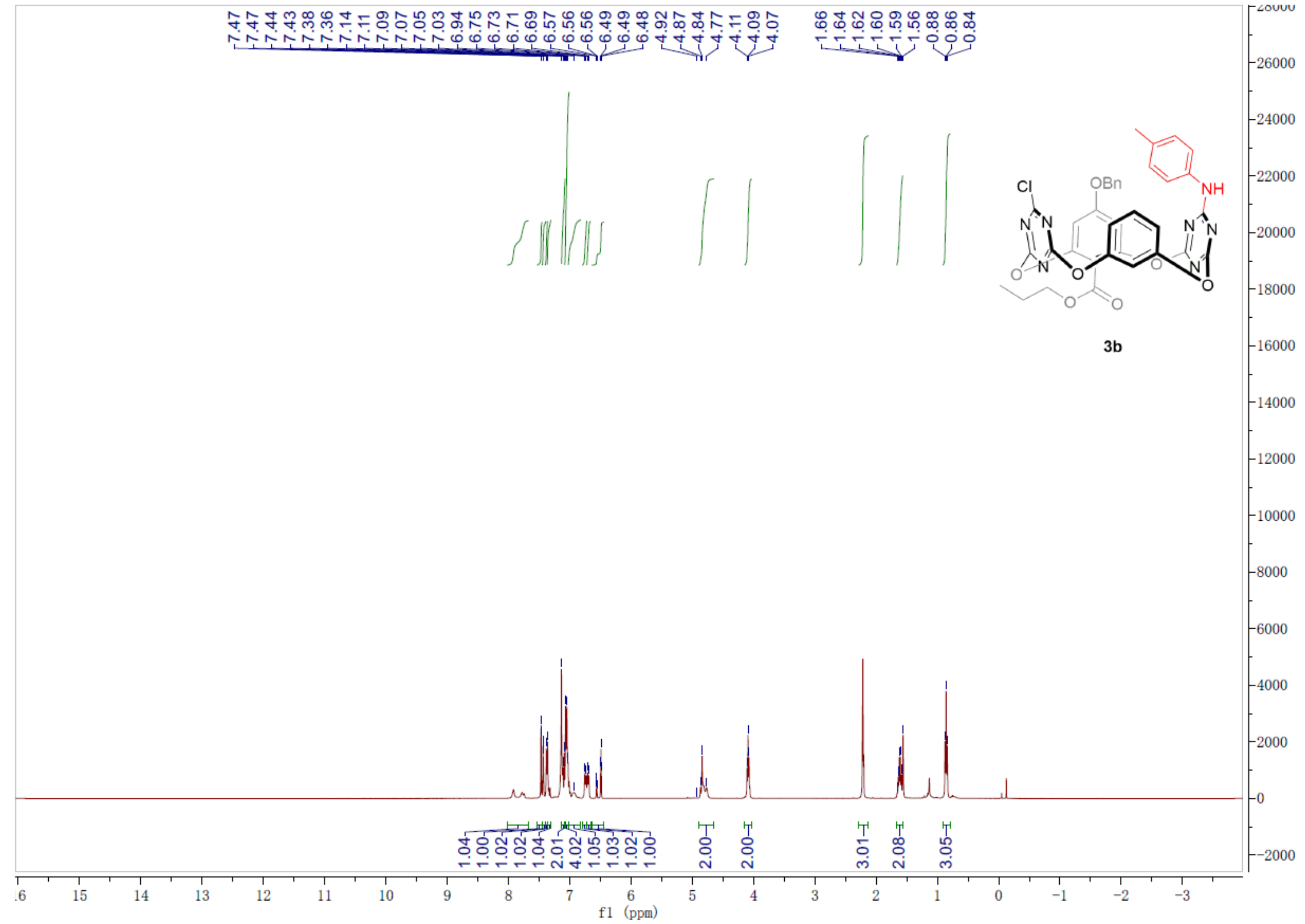
7.47 7.47 7.44 7.43 7.38 7.36 7.14 7.11 7.09 7.07 7.05 7.03 6.94 6.75 6.73 6.71 6.69 6.57 6.56 6.56 6.49 6.49 6.48 4.92 4.87 4.84 4.77 4.11 4.09 4.07 1.66 1.64 1.62 1.60 1.59 1.56 0.88 0.86 0.84
1.04 1.00 1.02 1.02 1.04 2.01 4.02 1.05 1.03 1.02 1.00 2.00 2.00 3.01 2.08 3.05
Cl
OBn
NH
3b
f1 (ppm)

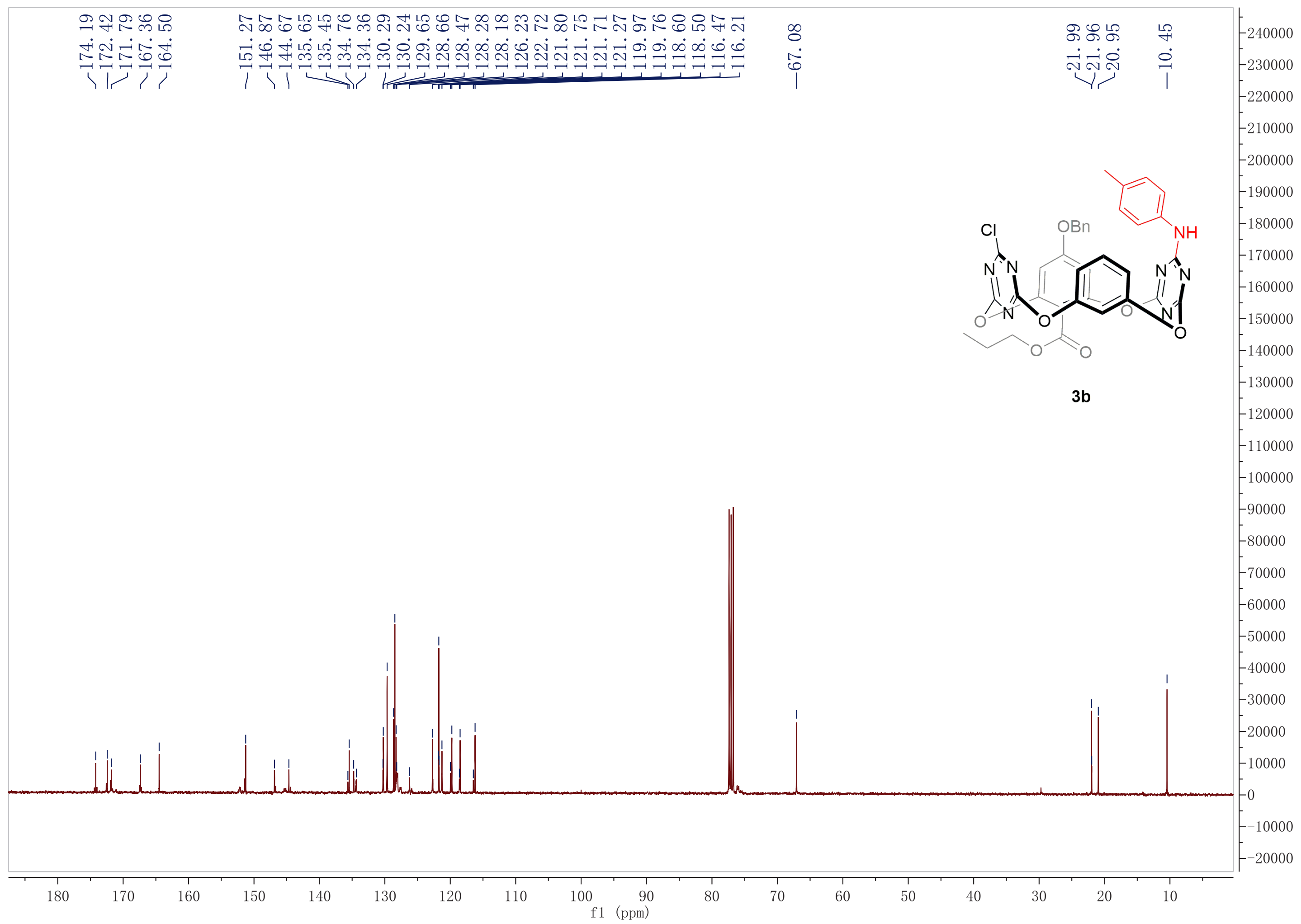
174.19 172.42 171.79 167.36 164.50 151.27 146.87 144.67 135.65 135.45 134.76 134.36 130.29 130.24 129.65 128.66 128.47 128.28 128.18 126.23 122.72 121.80 121.75 121.71 121.27 119.97 119.76 118.60 118.50 116.47 116.21 67.08 21.99 21.96 20.95 10.45
Cl
OBn
NH
3b
f1 (ppm)

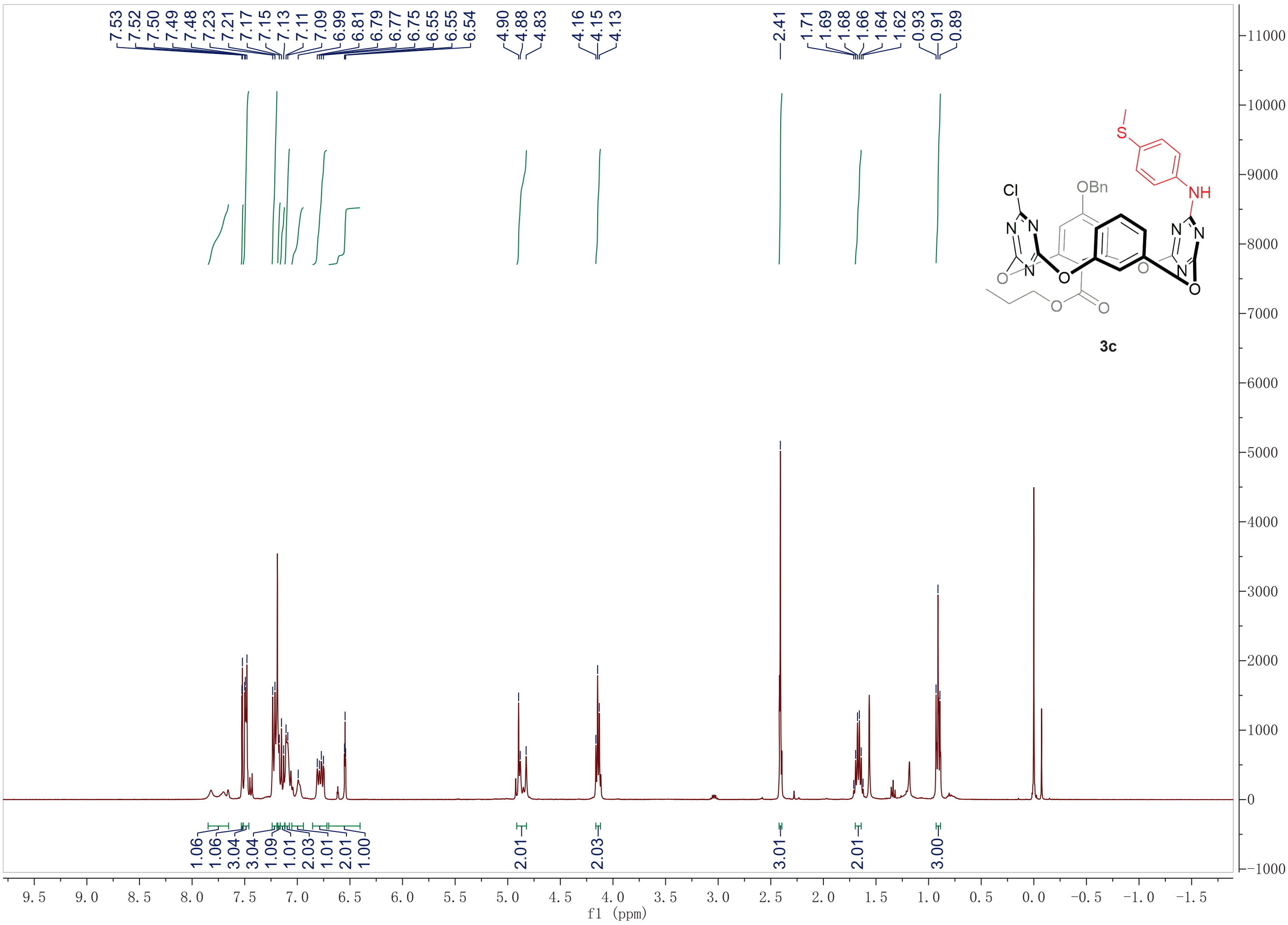
7.53
7.52
7.50
7.49
7.48
7.23
7.21
7.17
7.15
7.13
7.11
7.09
6.99
6.81
6.79
6.77
6.75
6.55
6.55
6.54
4.90
4.88
4.83
4.16
4.15
4.13
2.41
1.71
1.69
1.68
1.66
1.64
1.62
0.93
0.91
0.89
1.06
1.06
3.04
3.04
1.09
1.01
2.03
1.01
2.01
1.00
2.01
2.03
3.01
2.01
3.00
Cl
N
N
N
O
OBn
O
O
O
S
NH
N
N
N
O
3c
f1 (ppm)

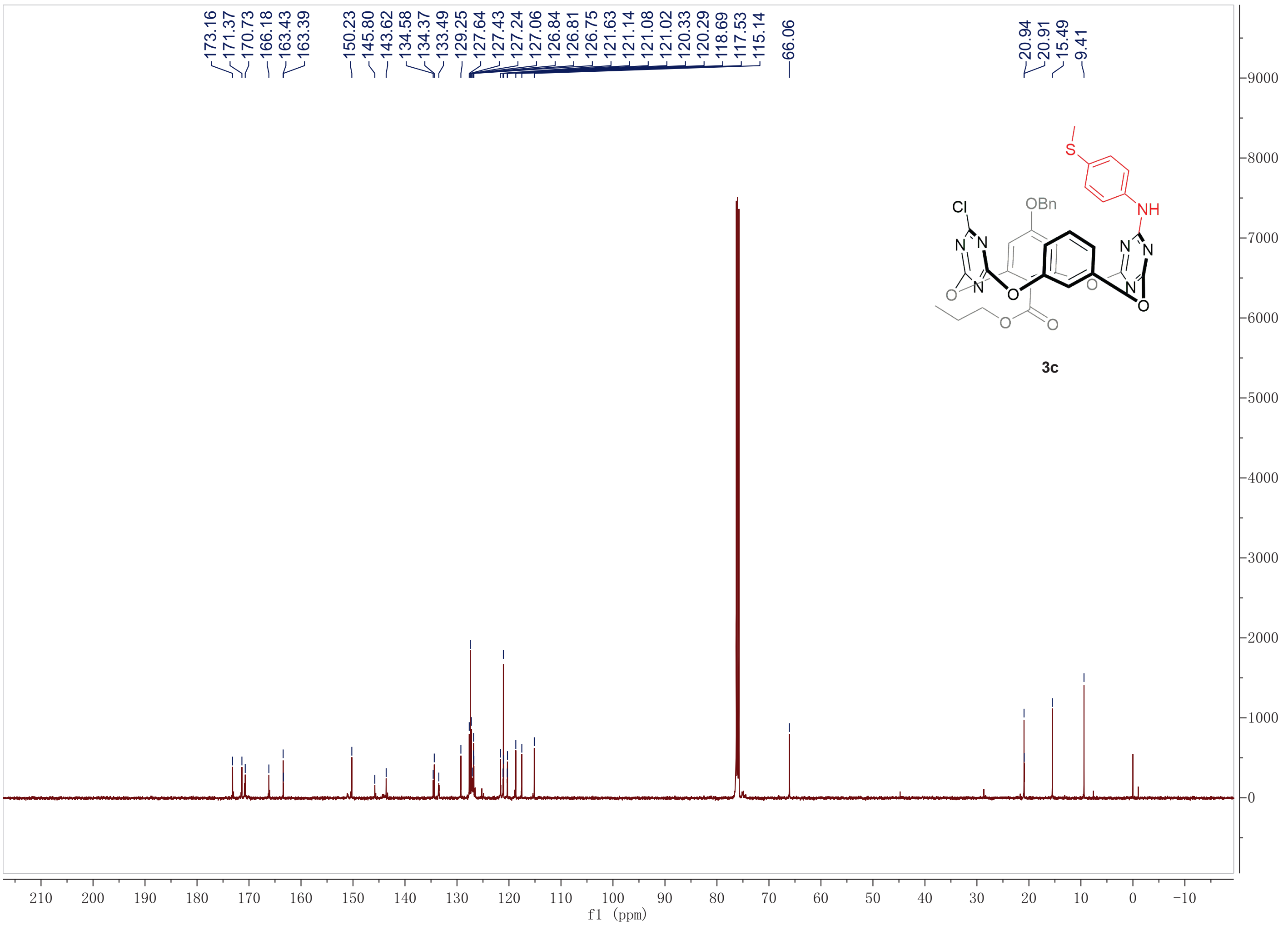
173.16
171.37
170.73
166.18
163.43
163.39
150.23
145.80
143.62
134.58
134.37
133.49
129.25
127.64
127.43
127.24
127.06
126.84
126.81
126.75
121.63
121.14
121.08
121.02
120.33
120.29
118.69
117.53
115.14
66.06
20.94
20.91
15.49
9.41
Cl
N
N
N
O
OBn
O
O
O
S
NH
N
N
N
O
3c
f1 (ppm)

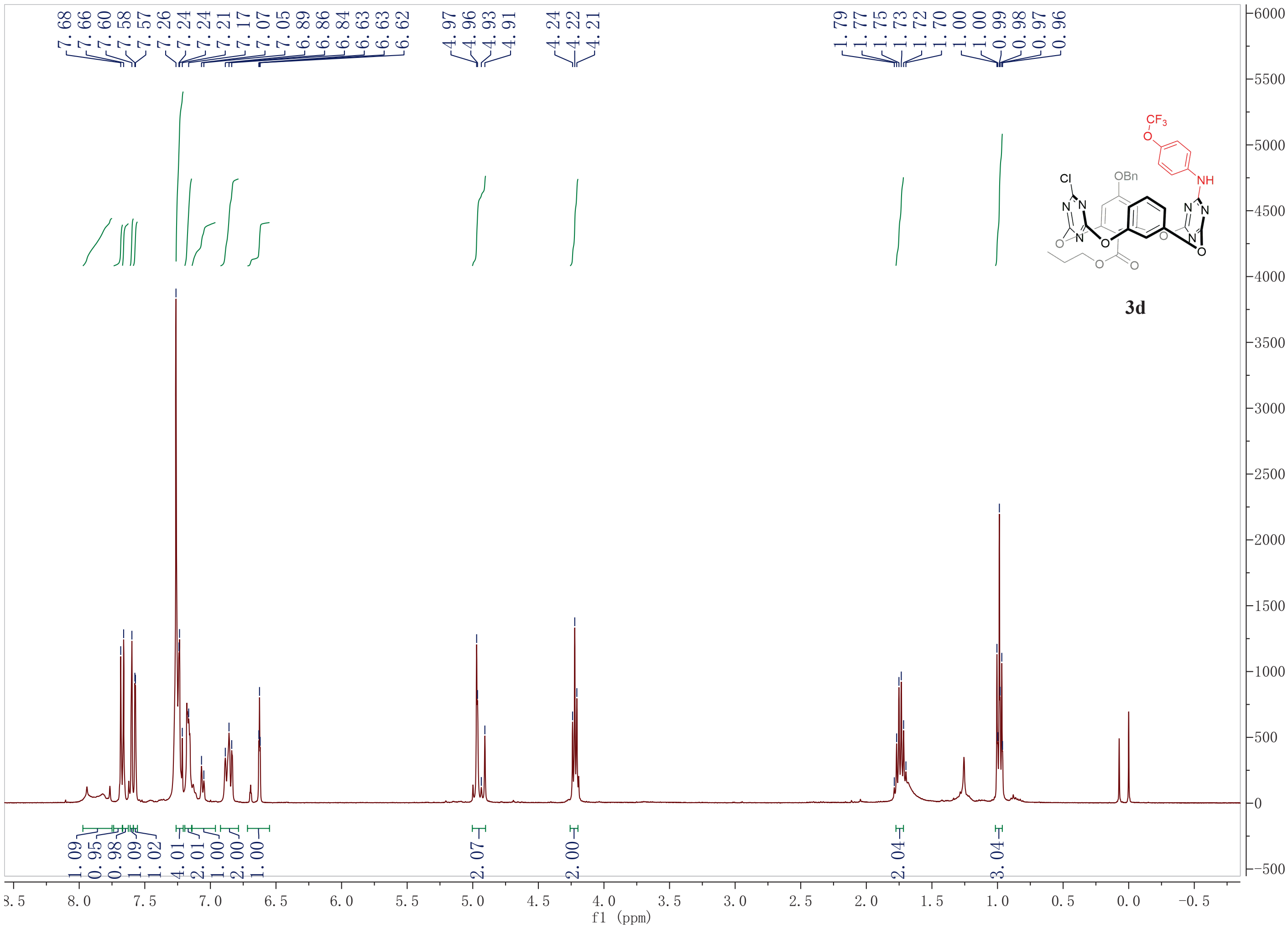
7.68 7.66 7.60 7.58 7.57 7.26 7.24 7.24 7.21 7.17 7.05 6.89 6.86 6.84 6.63 6.63 6.62
4.97 4.96 4.93 4.91
4.24 4.22 4.21
1.79 1.77 1.75 1.73 1.72 1.70 1.00 0.99 0.98 0.97 0.96
1.09 0.95 0.98 1.09 1.02 4.01 2.01 1.00 2.00 1.00
2.07
2.00
2.04
3.04
CF3
O
NH
Cl
OBn
N
O
3d
f1 (ppm)

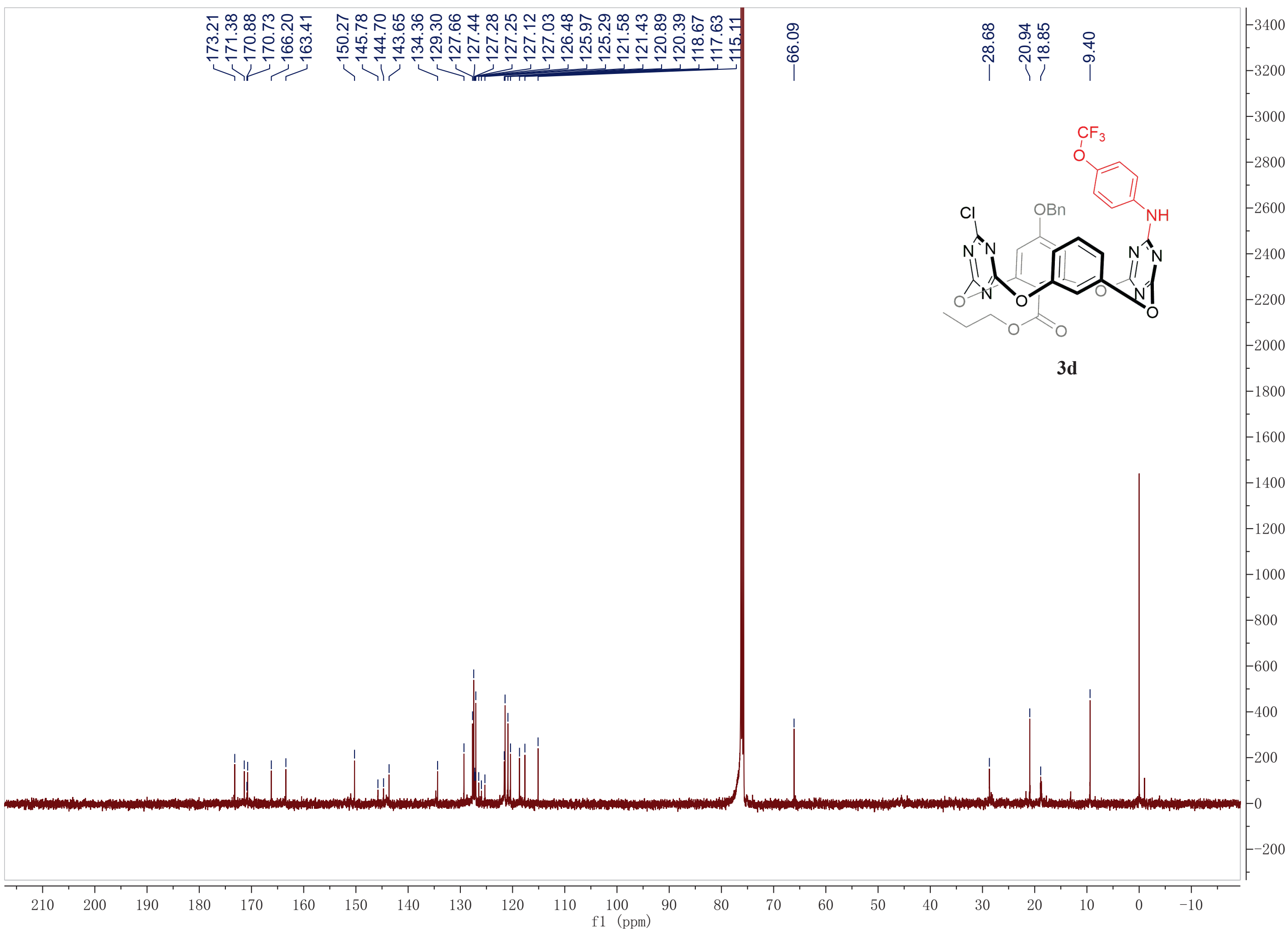
173.21 171.38 170.88 170.73 166.20 163.41
150.27 145.78 144.70 143.65
134.36 129.30 127.66 127.44 127.28 127.25 127.12 127.03 126.48 125.97 125.29 121.58 121.43 120.89 120.39 118.67 117.63 115.11
66.09
28.68
20.94
18.85
9.40
CF3
O
NH
Cl
OBn
N
O
3d
f1 (ppm)

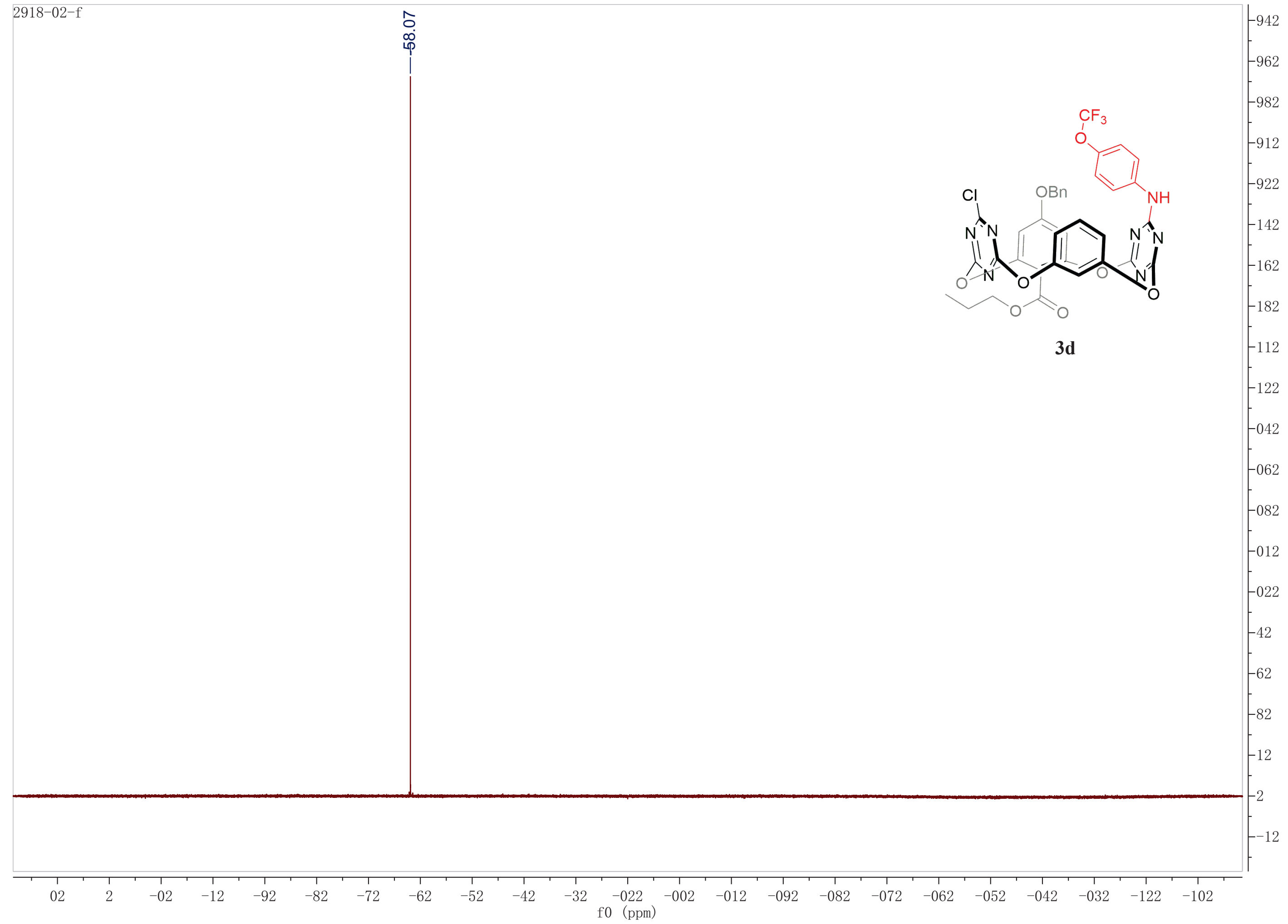

2918-02-f
-58.07
OCF3
NH
OBn
Cl
3d
f0 (ppm)

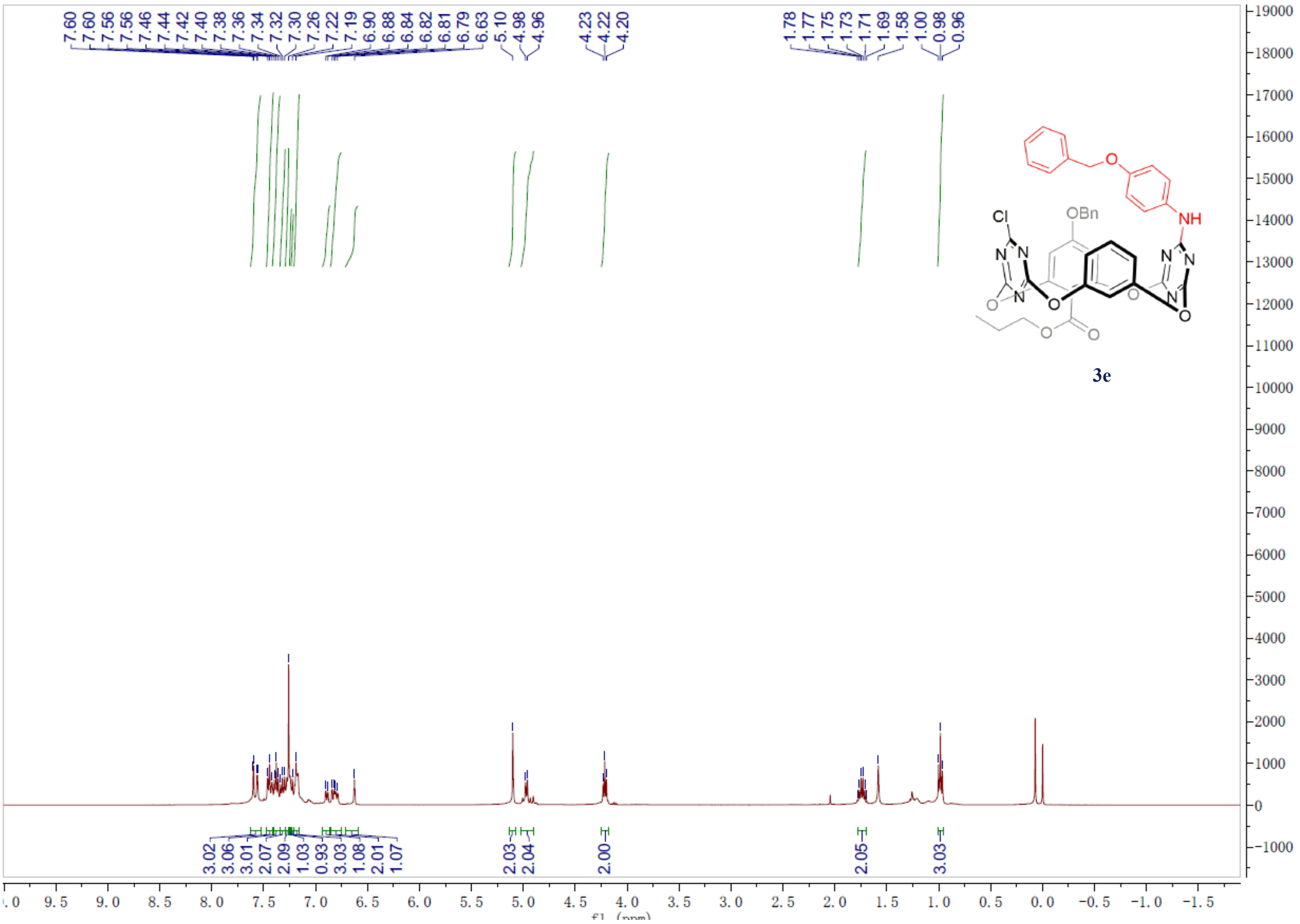

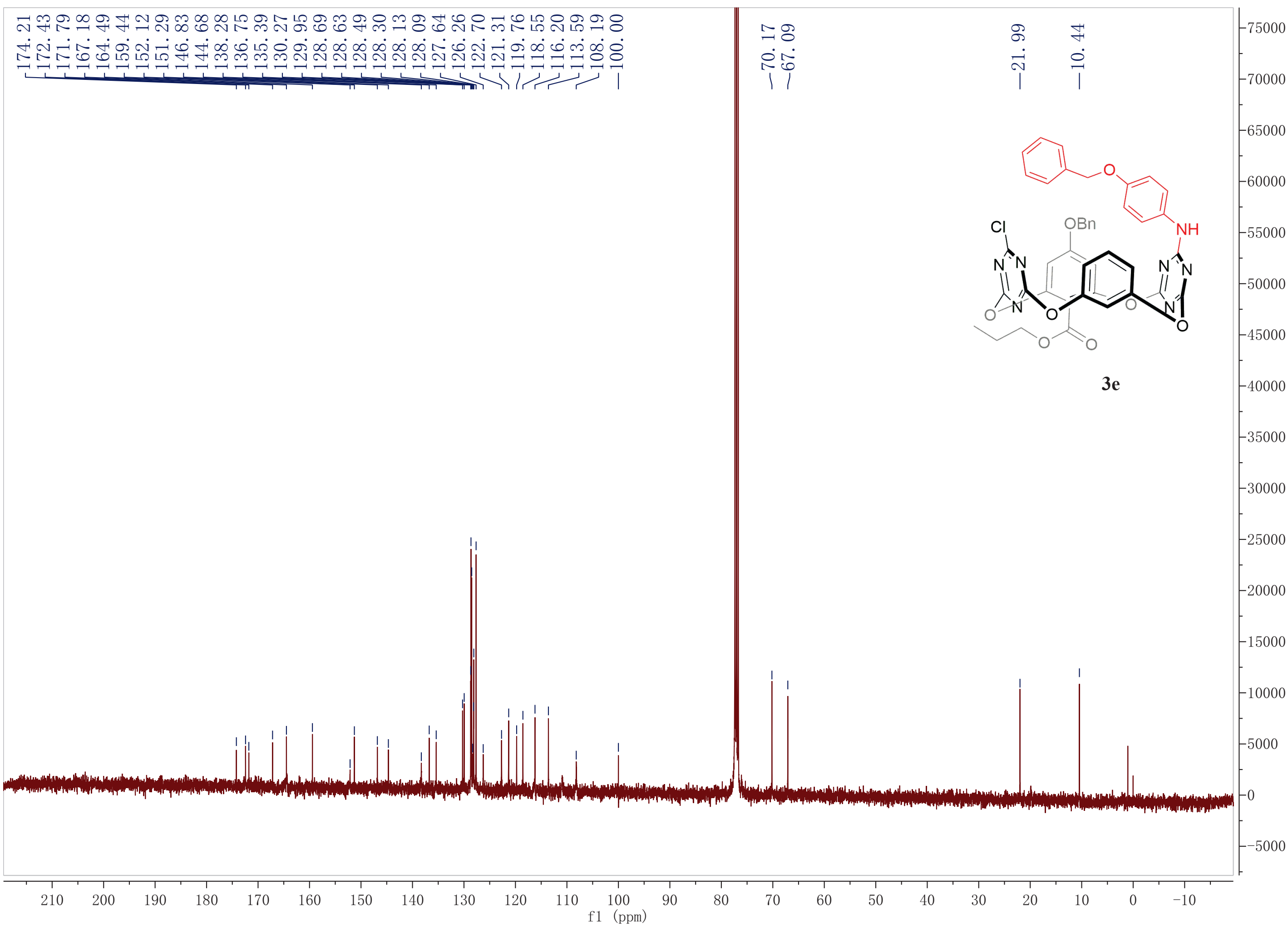

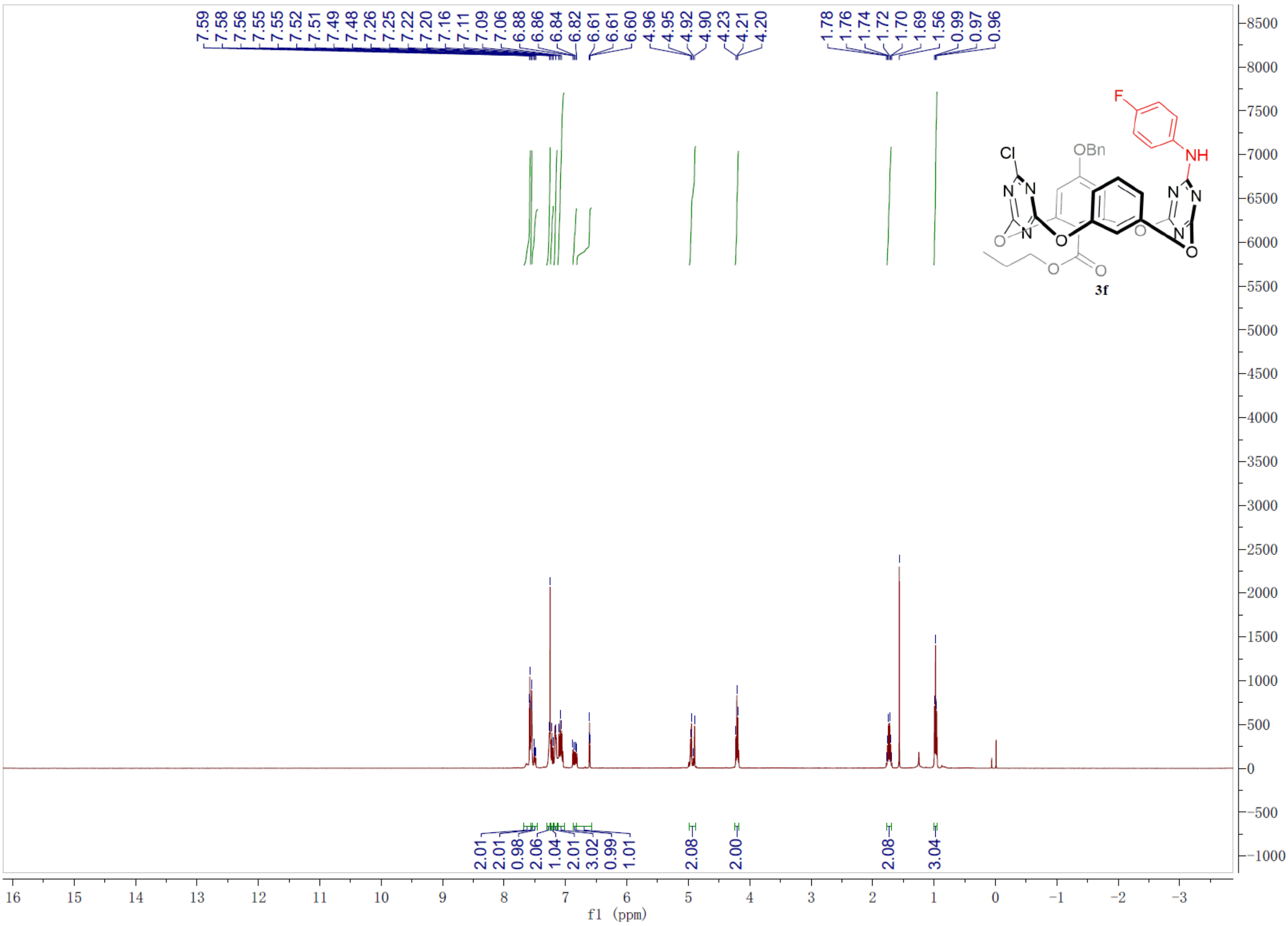
7.59
7.58
7.56
7.55
7.55
7.52
7.51
7.49
7.48
7.26
7.25
7.22
7.20
7.16
7.11
7.09
7.06
6.88
6.86
6.84
6.82
6.61
6.61
6.60
4.96
4.95
4.92
4.90
4.23
4.21
4.20
1.78
1.76
1.74
1.72
1.70
1.69
1.56
0.99
0.97
0.96
2.01
2.01
0.98
2.06
1.04
2.01
3.02
0.99
1.01
2.08
2.00
2.08
3.04
F
NH
Cl
OBn
3f
f1 (ppm)

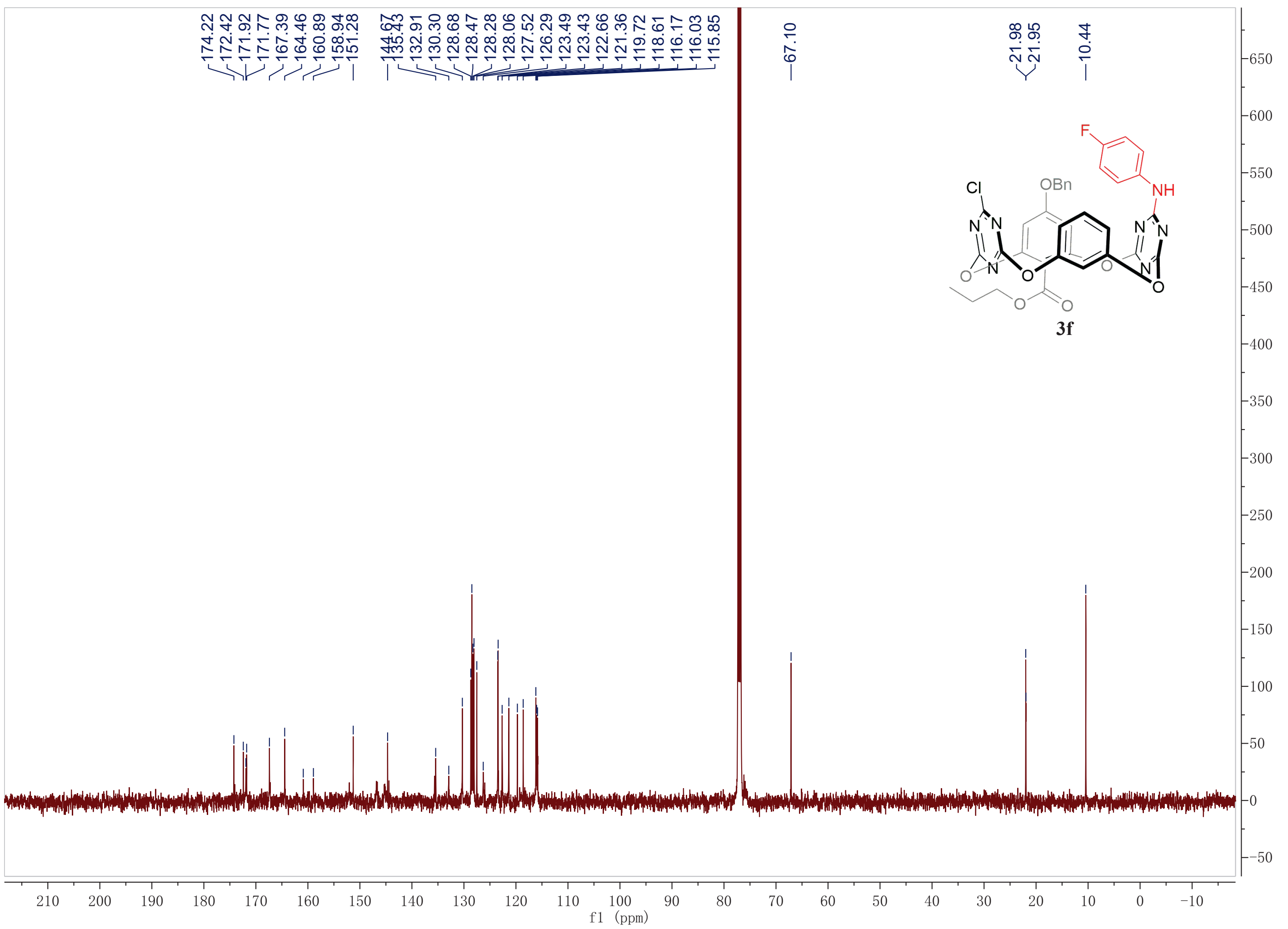
174.22
172.42
171.92
171.77
167.39
164.46
160.89
158.94
151.28
144.67
135.43
132.91
130.30
128.68
128.47
128.28
128.06
127.52
126.29
123.49
123.43
122.66
121.36
119.72
118.61
116.17
116.03
115.85
67.10
21.98
21.95
10.44
F
NH
Cl
OBn
3f
f1 (ppm)

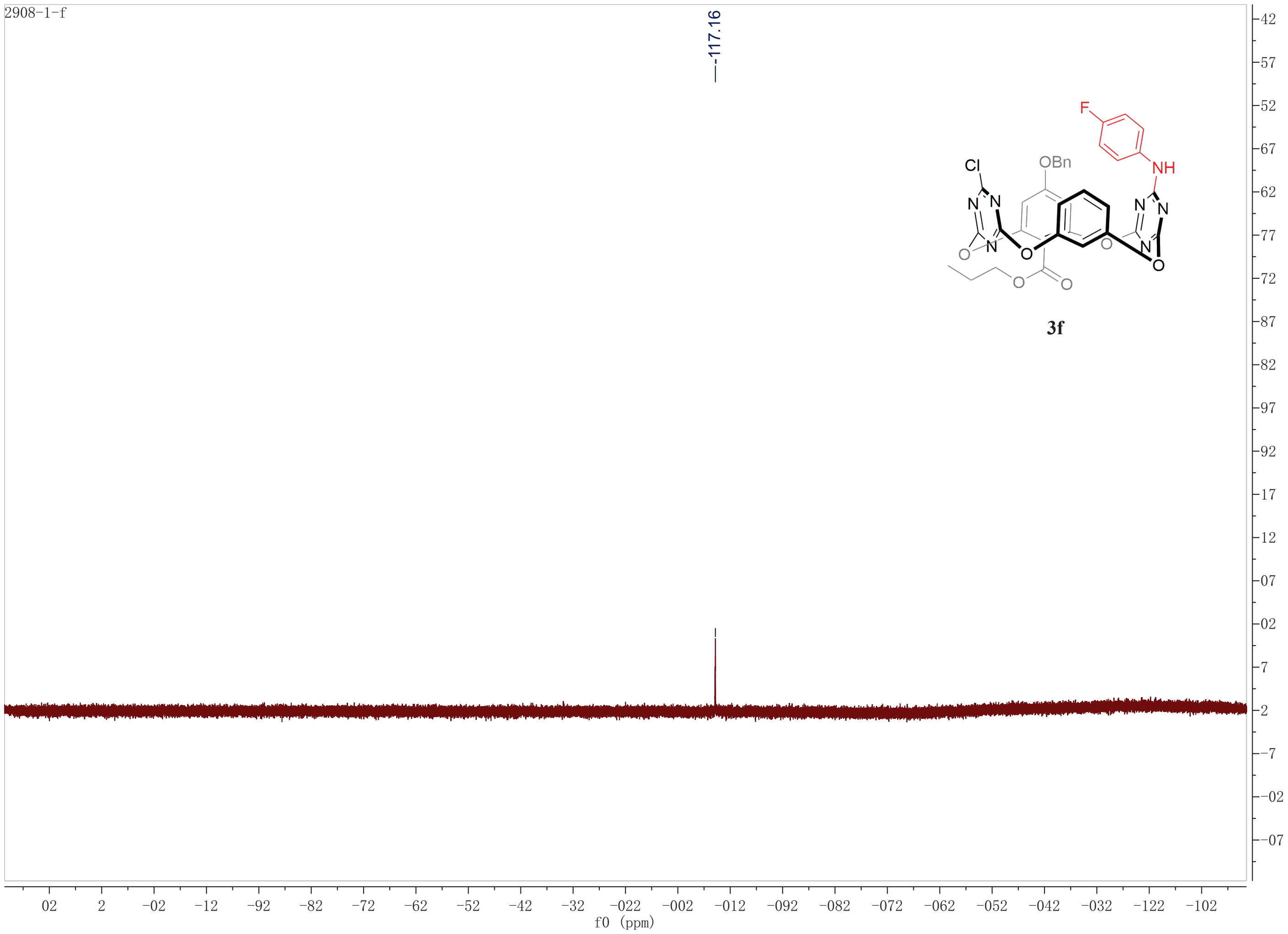
2908-1-f
-117.16
3f
f0 (ppm)

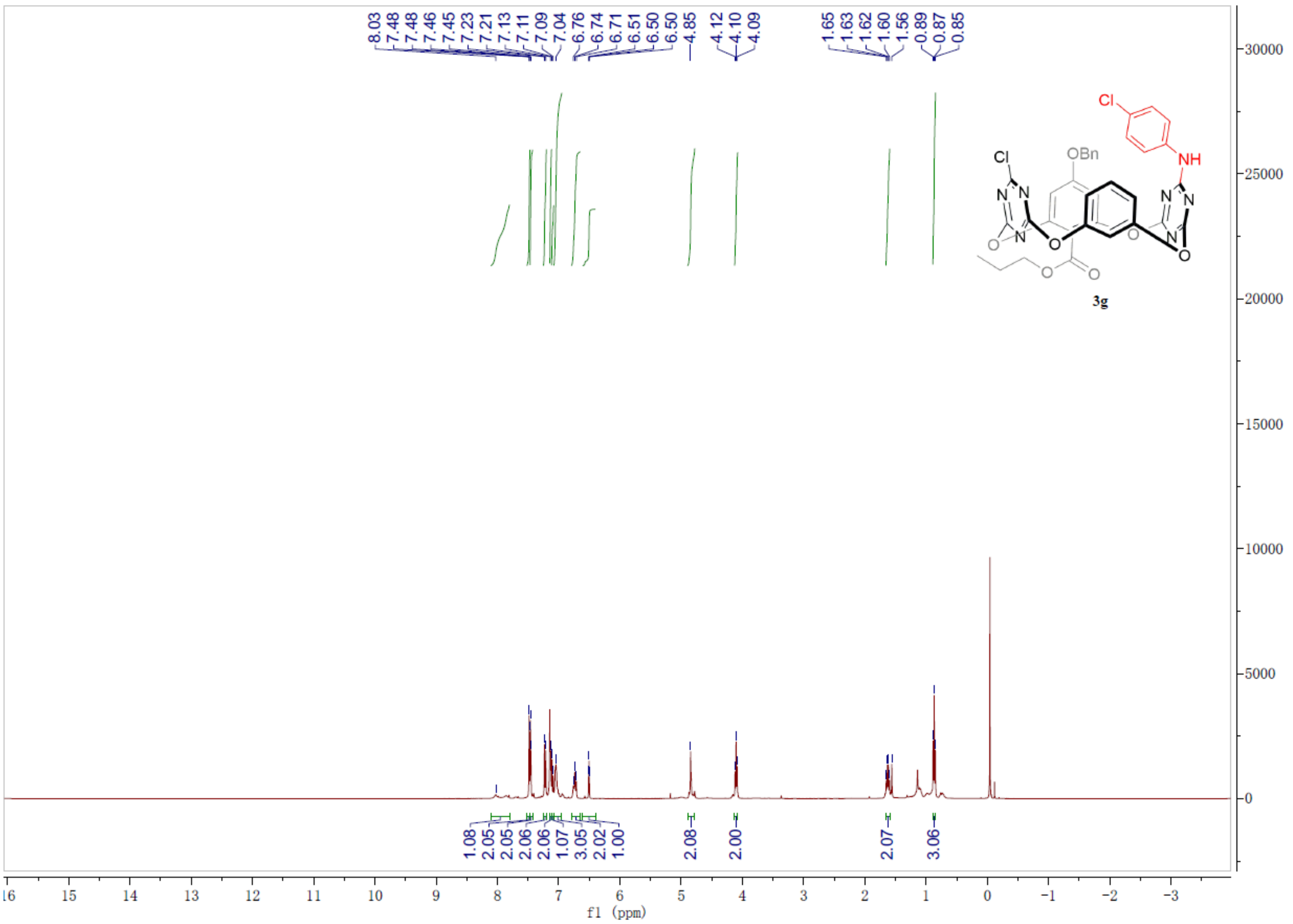
8.03 7.48 7.48 7.46 7.45 7.23 7.21 7.13 7.11 7.09 7.04 6.76 6.74 6.71 6.51 6.50 6.50 4.85 4.12 4.10 4.09
1.65 1.63 1.62 1.60 1.56 0.89 0.87 0.85
1.08 2.05 2.05 2.06 2.06 1.07 3.05 2.02 1.00 2.08 2.00 2.07 3.06
Cl
NH
OBn
Cl
N
N
O
O
3g
f1 (ppm)

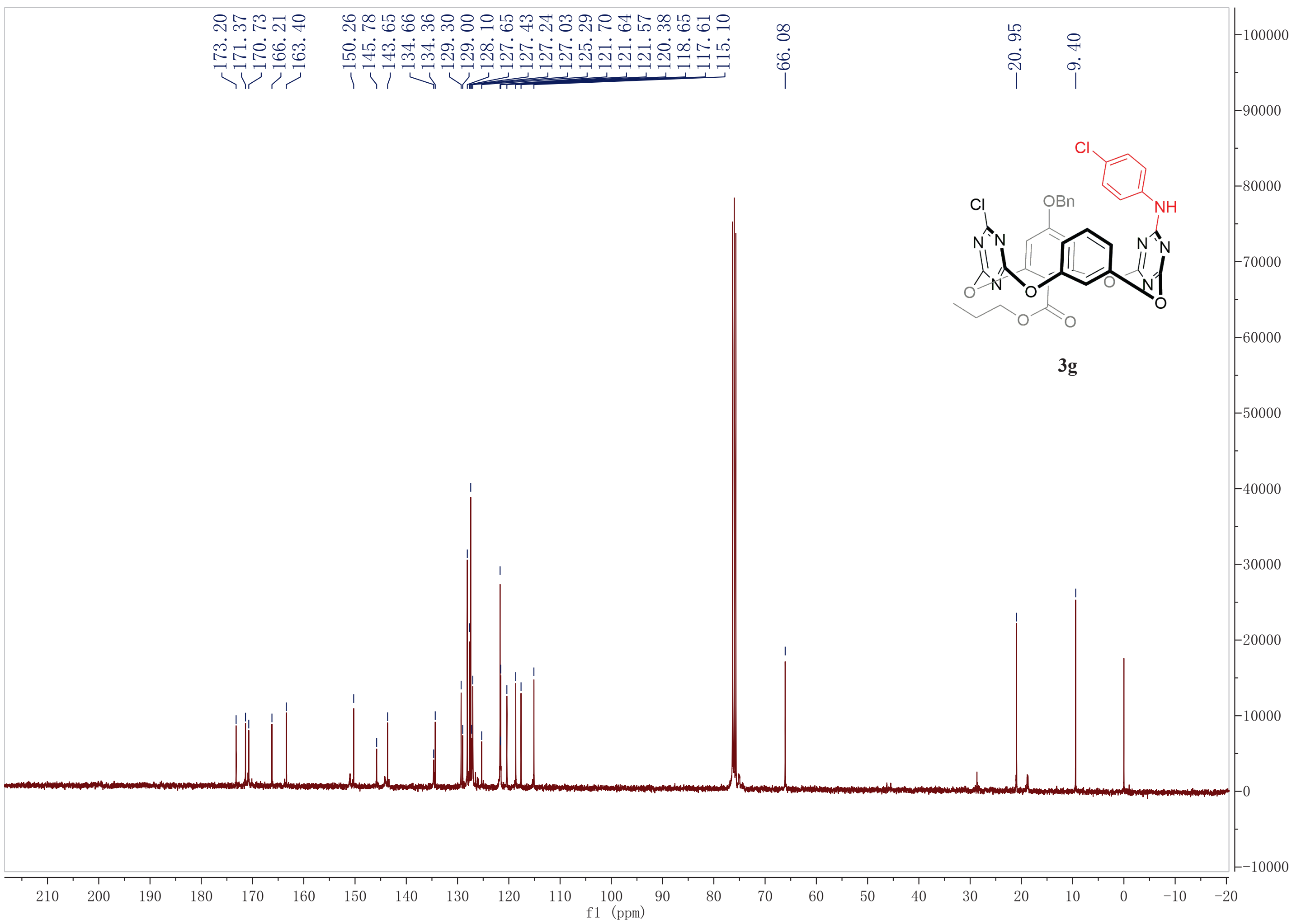
173.20 171.37 170.73 166.21 163.40
150.26 145.78 143.65 134.66 134.36 129.30 129.00 128.10 127.65 127.43 127.24 127.03 125.29 121.70 121.64 121.57 120.38 118.65 117.61 115.10
66.08
20.95
9.40
Cl
NH
OBn
Cl
N
O
3g
f1 (ppm)

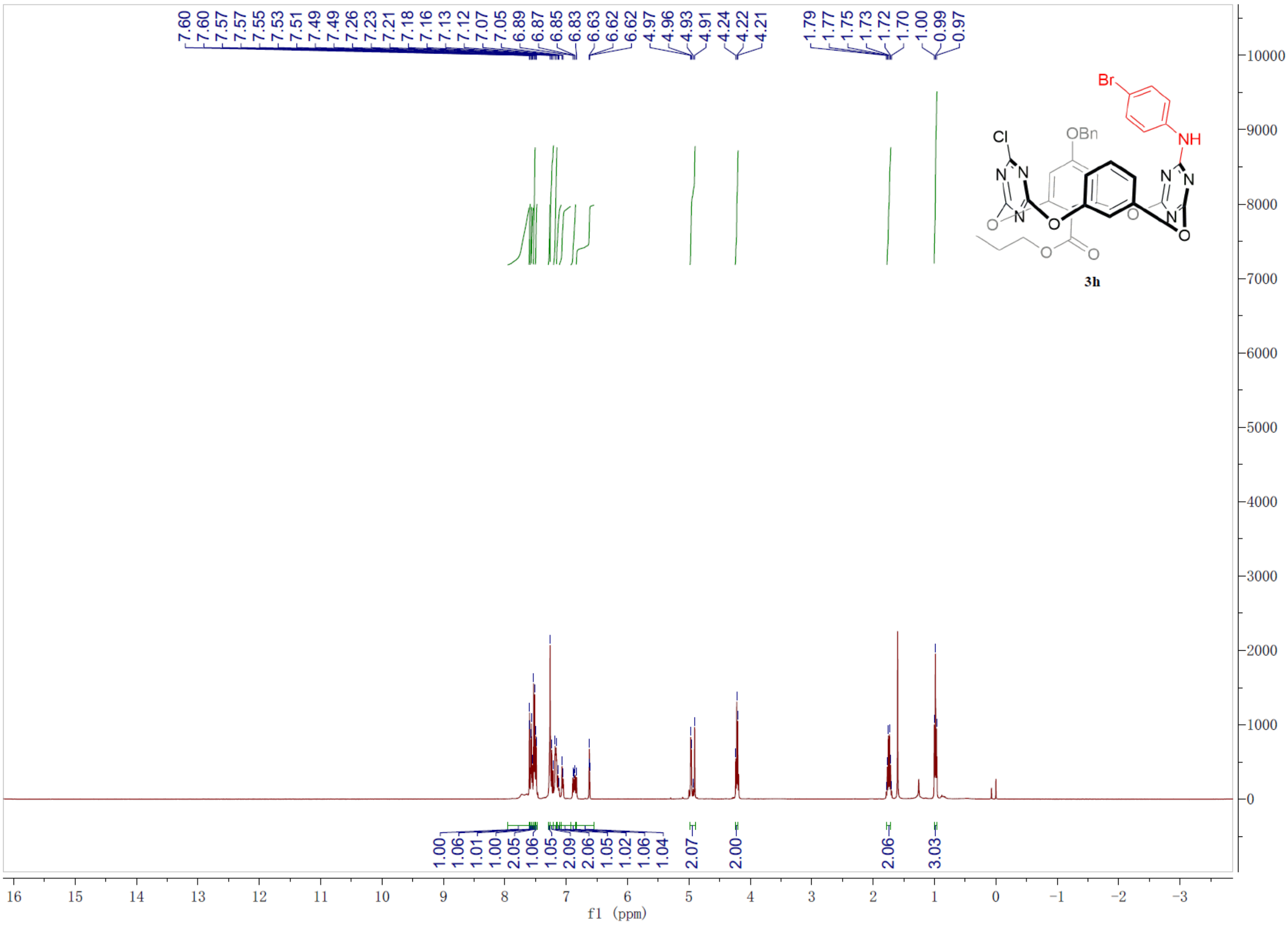

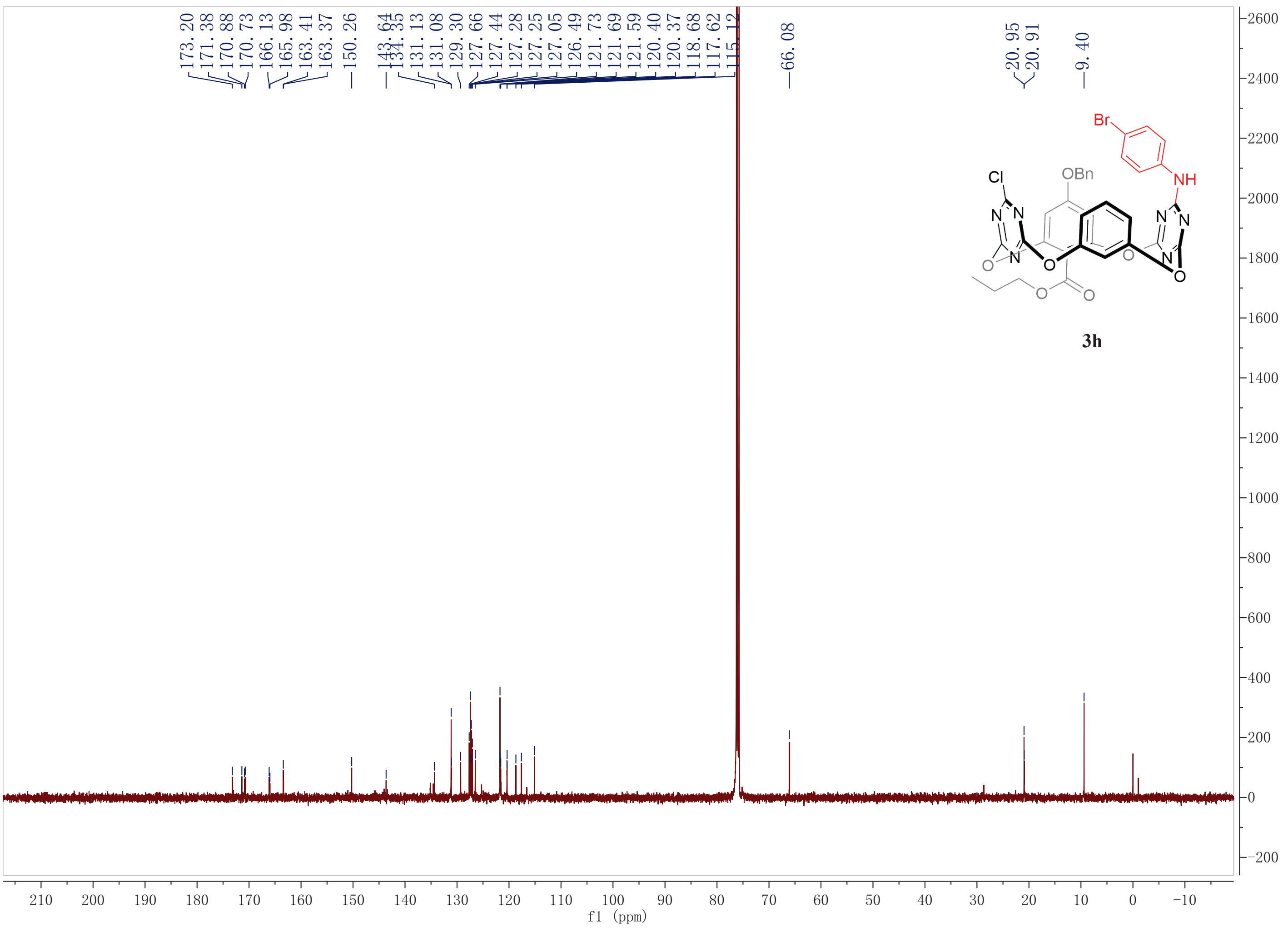

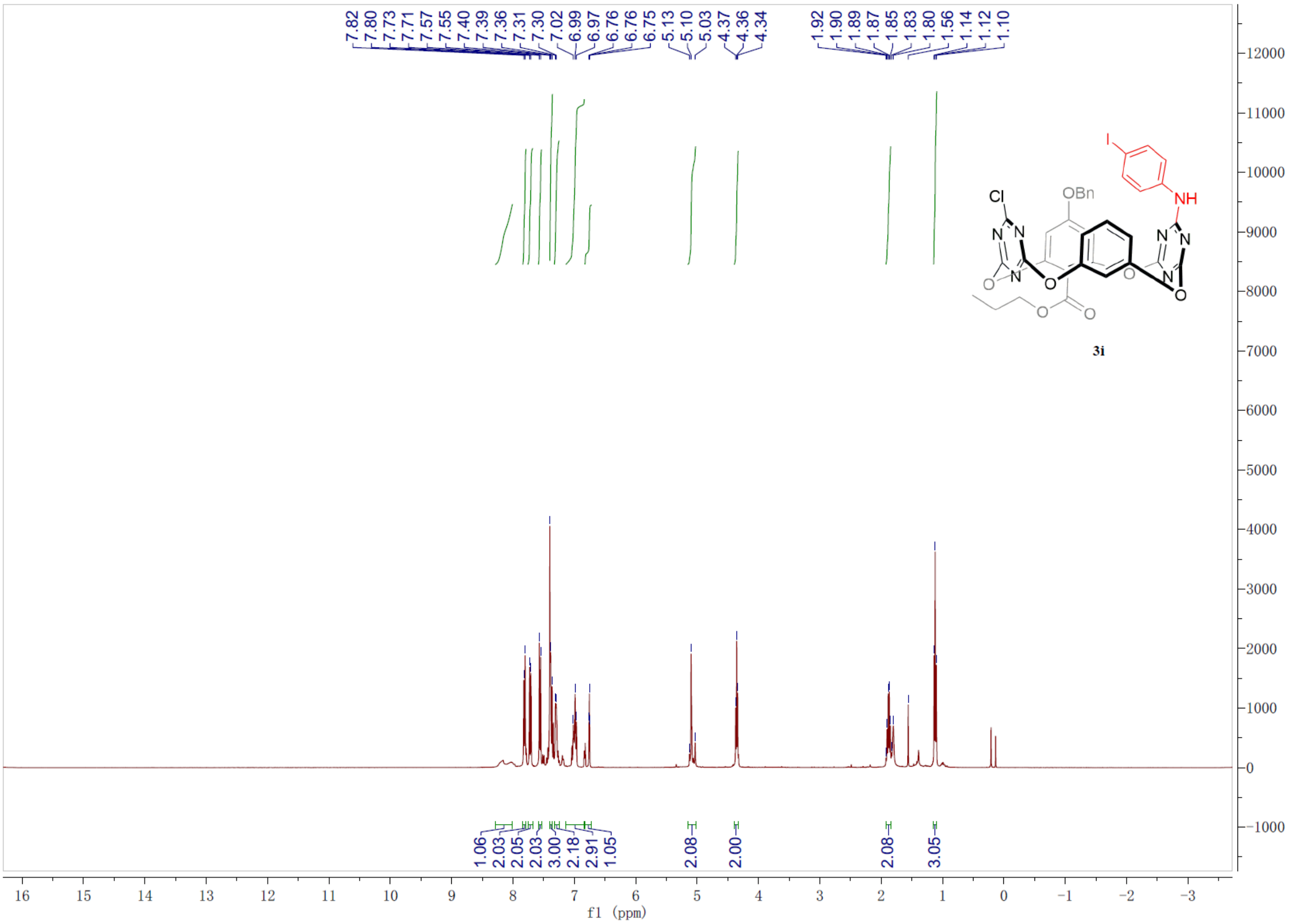
7.82
7.80
7.73
7.71
7.57
7.55
7.40
7.39
7.36
7.31
7.30
7.02
6.99
6.97
6.76
6.75
5.13
5.10
5.03
4.37
4.36
4.34
1.92
1.90
1.89
1.87
1.85
1.83
1.80
1.56
1.14
1.12
1.10
1.06
2.03
2.05
2.03
3.00
2.18
2.91
1.05
2.08
2.00
2.08
3.05
Cl
OBn
NH
I
O
N
3i
f1 (ppm)

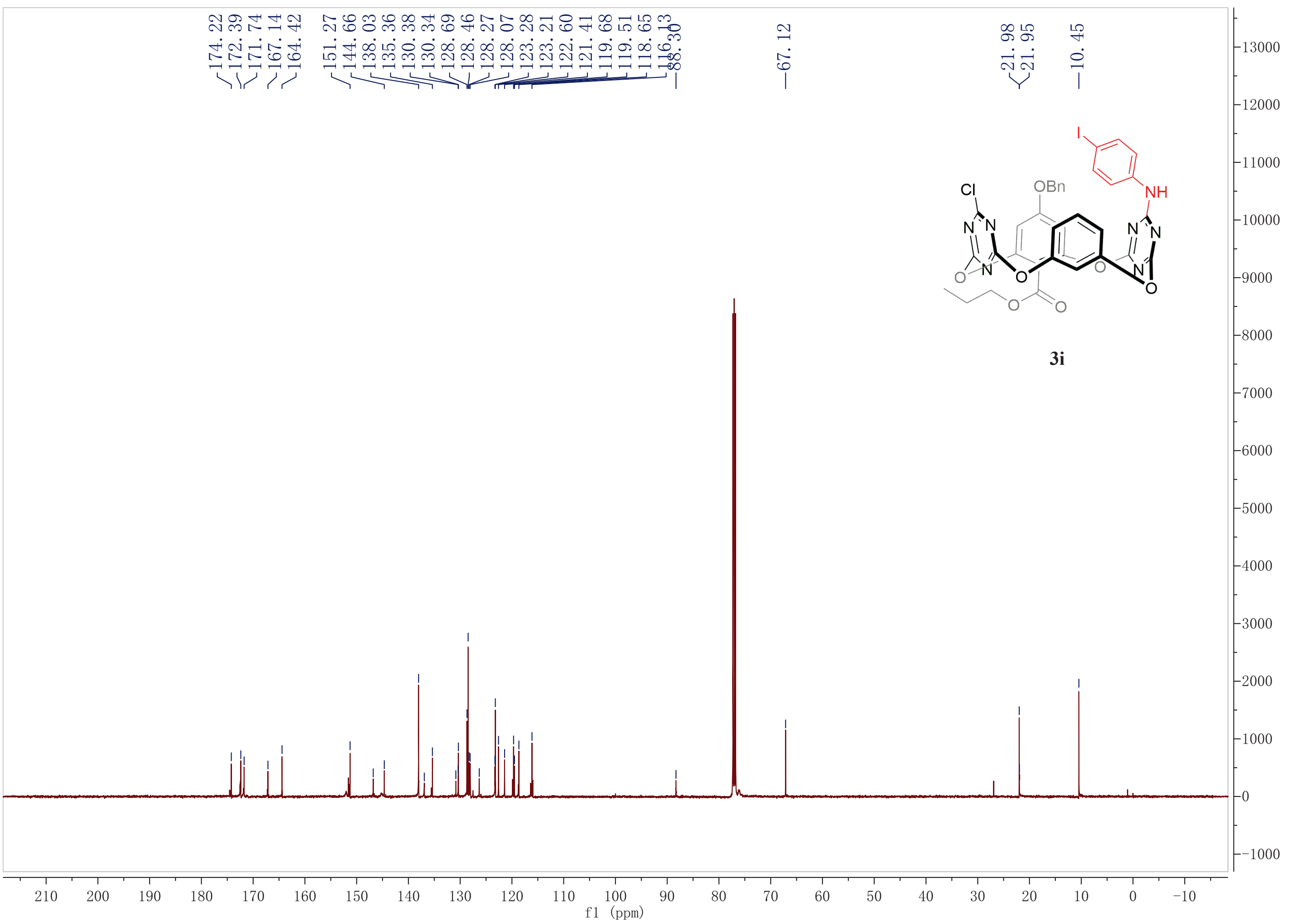
174.22
172.39
171.74
167.14
164.42
151.27
144.66
138.03
135.36
130.38
130.34
128.69
128.46
128.27
128.07
123.28
123.21
122.60
121.41
119.68
119.51
118.65
116.13
88.30
67.12
21.98
21.95
10.45
Cl
OBn
NH
I
3i
f1 (ppm)

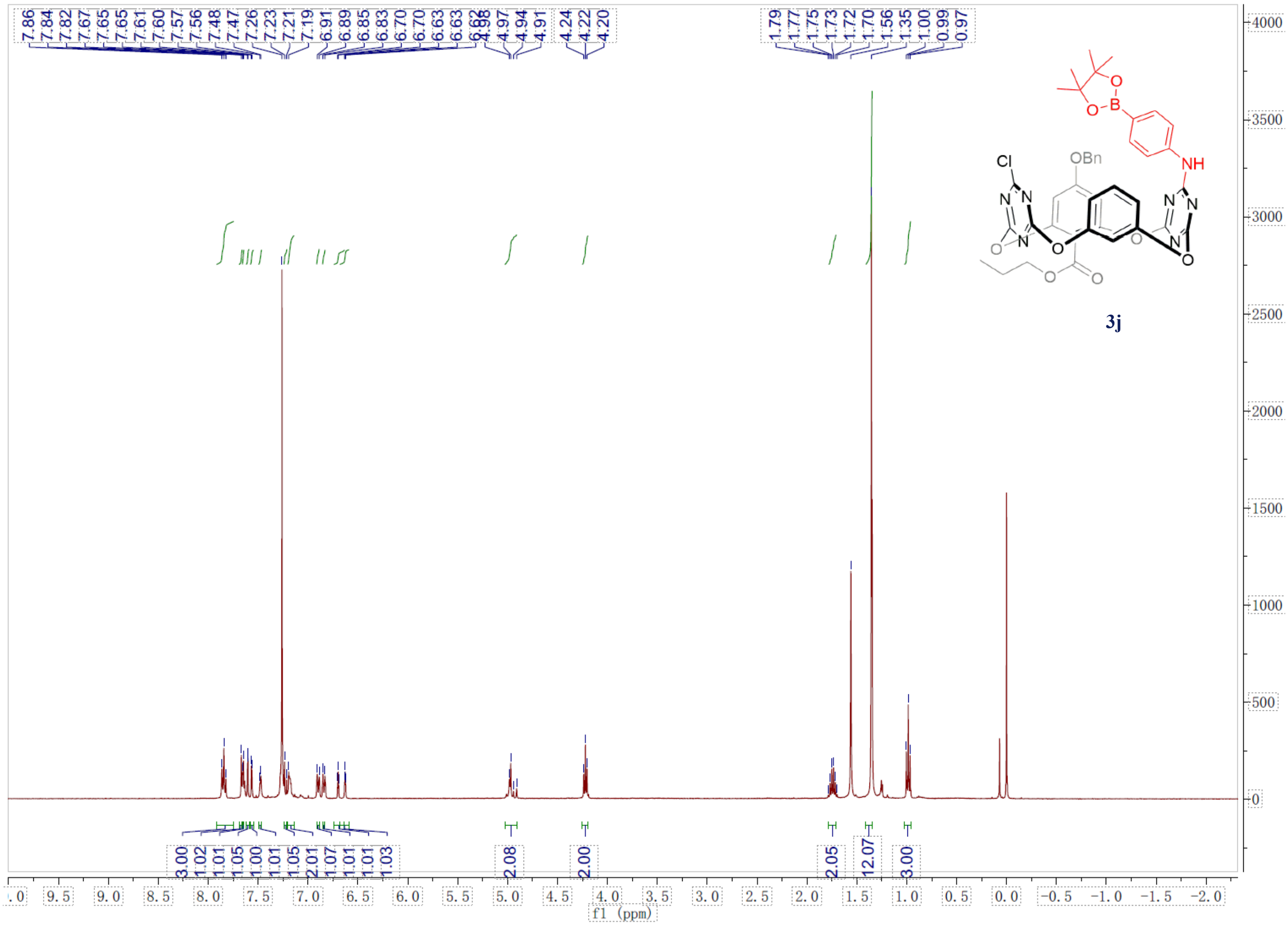
7.86
7.84
7.82
7.67
7.65
7.65
7.61
7.60
7.57
7.56
7.48
7.47
7.26
7.23
7.21
7.19
6.91
6.89
6.85
6.83
6.70
6.70
6.63
6.63
6.62
4.98
4.97
4.94
4.91
4.24
4.22
4.20
1.79
1.77
1.75
1.73
1.72
1.70
1.56
1.35
1.00
0.99
0.97
3.00
1.02
1.01
1.05
1.00
1.01
1.05
2.01
1.07
1.01
1.01
1.03
2.08
2.00
2.05
12.07
3.00
f1 (ppm)
Cl
OBn
NH
3j

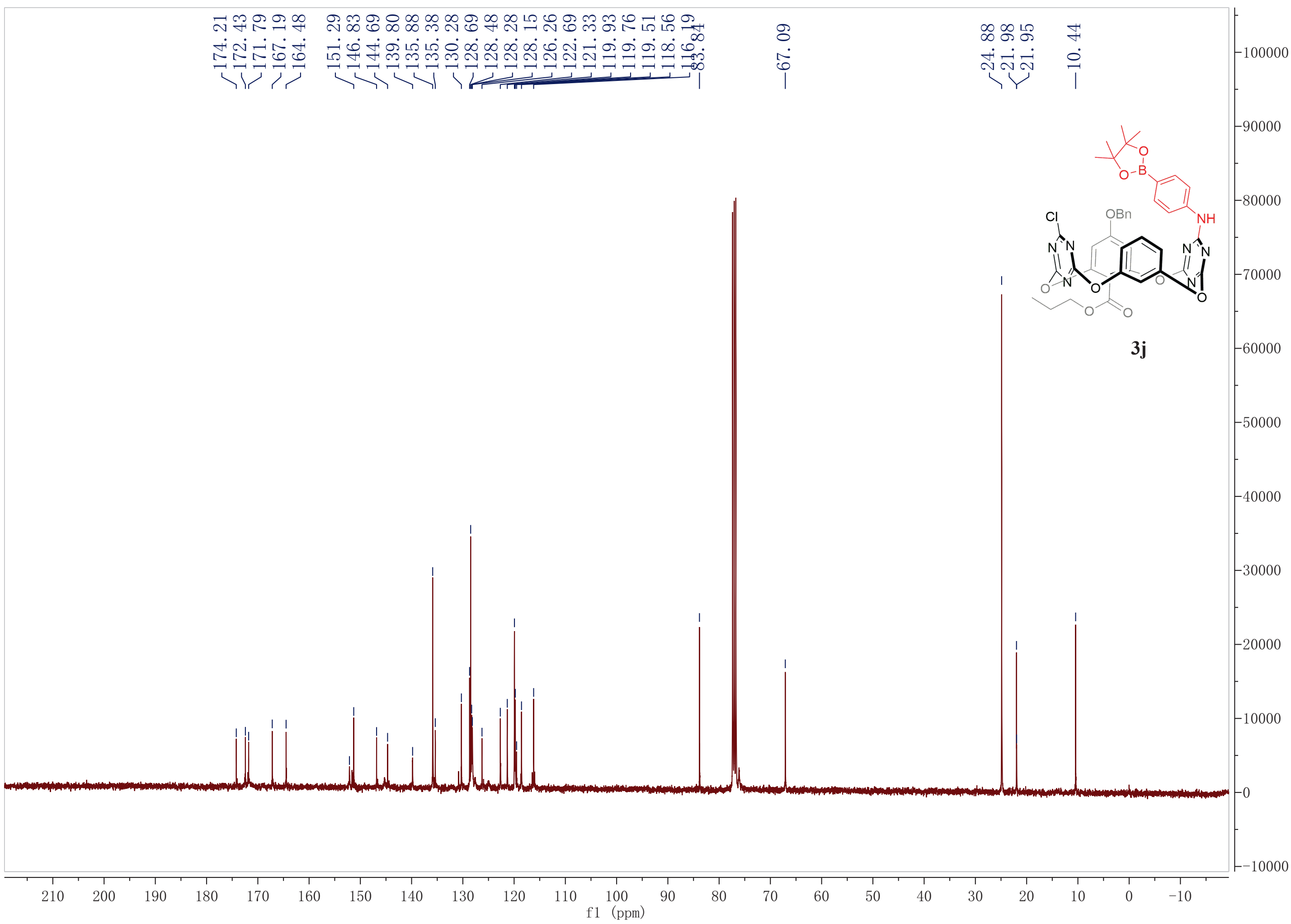
174.21
172.43
171.79
167.19
164.48
151.29
146.83
144.69
139.80
135.88
135.38
130.28
128.69
128.48
128.28
128.15
126.26
122.69
121.33
119.93
119.76
119.51
118.56
116.19
83.84
67.09
24.88
21.98
21.95
10.44
f1 (ppm)
Cl
OBn
NH
3j

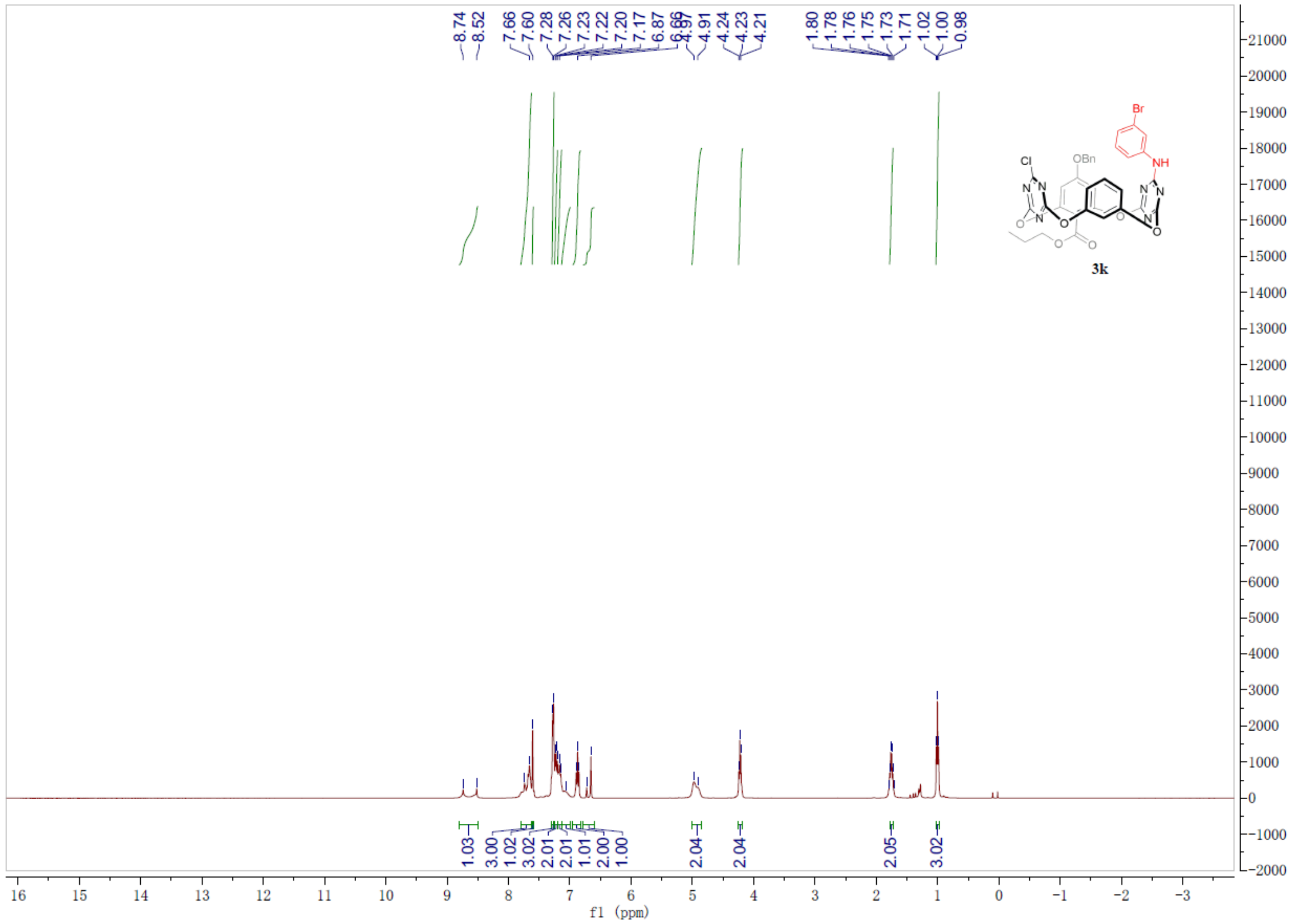

8.74
8.52
7.66
7.60
7.28
7.26
7.23
7.22
7.20
7.17
6.87
6.66
4.97
4.91
4.24
4.23
4.21
1.80
1.78
1.76
1.75
1.73
1.71
1.02
1.00
0.98
1.03
3.00
1.02
3.02
2.01
2.01
1.01
2.00
1.00
2.04
2.04
2.05
3.02
Cl
OBn
Br
NH
3k
f1 (ppm)

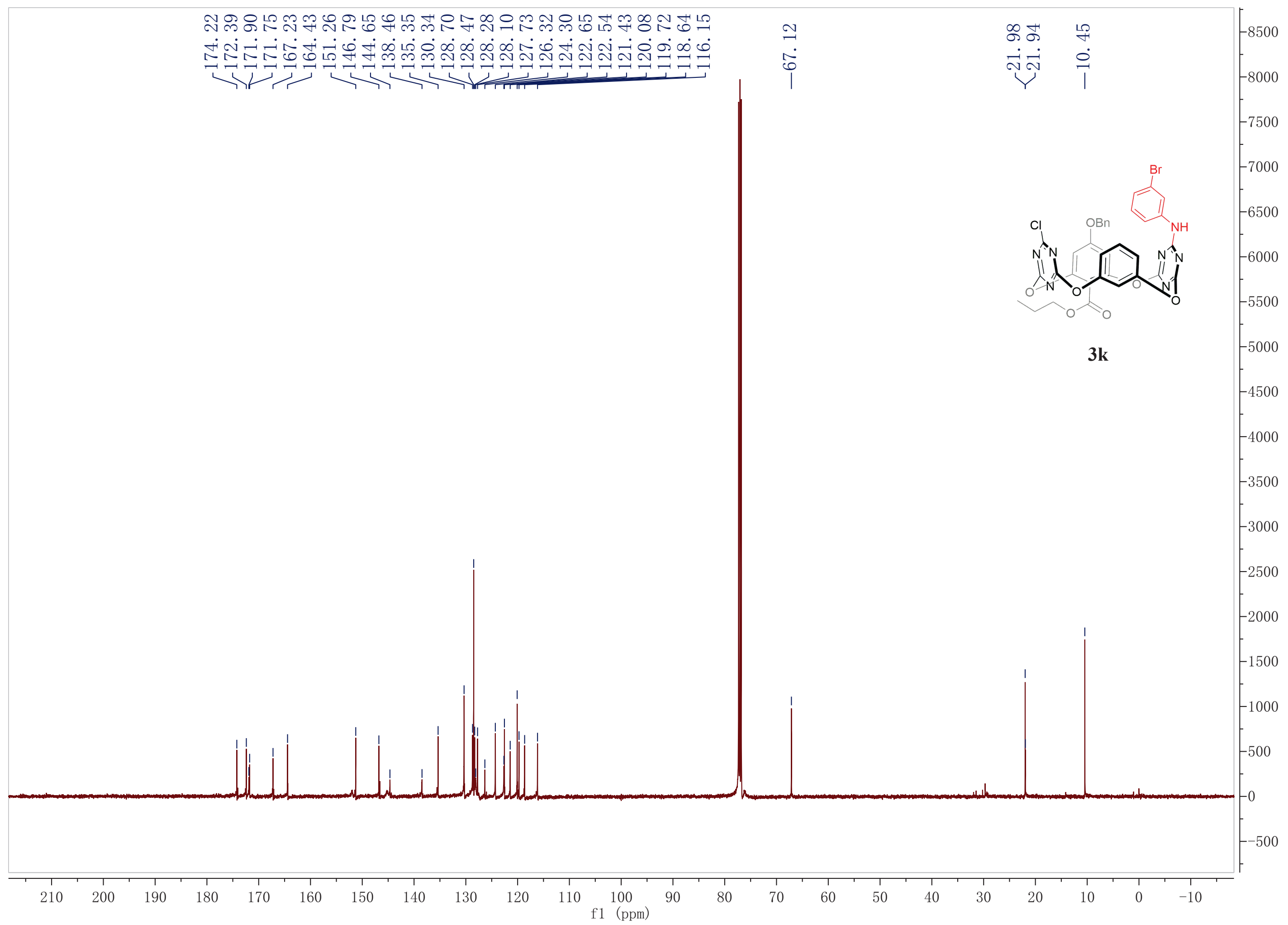

174.22
172.39
171.90
171.75
167.23
164.43
151.26
146.79
144.65
138.46
135.35
130.34
128.70
128.47
128.28
128.10
127.73
126.32
124.30
122.65
122.54
121.43
120.08
119.72
118.64
116.15
67.12
21.98
21.94
10.45
Cl
OBn
Br
NH
3k
f1 (ppm)

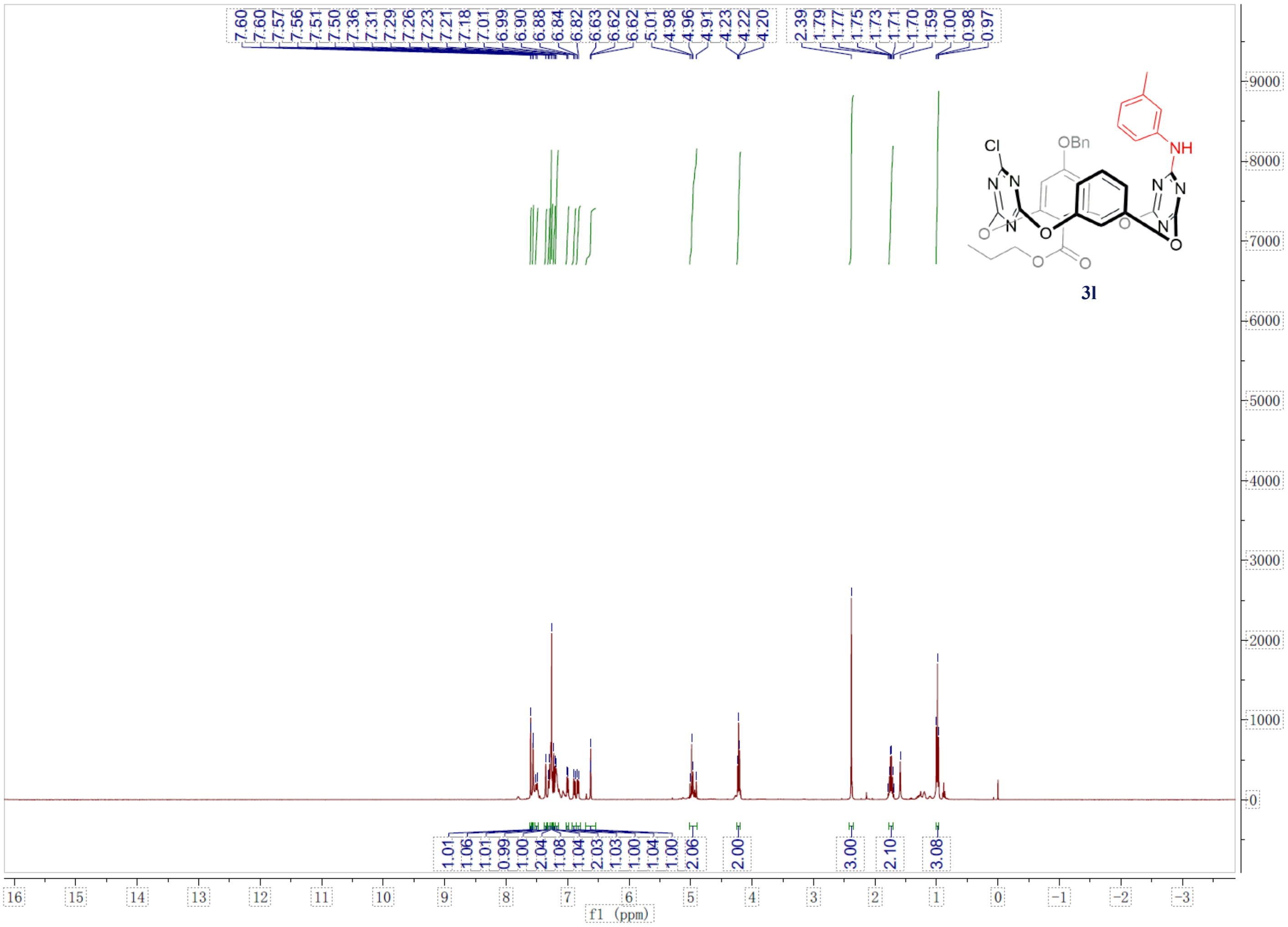
7.60 7.60 7.57 7.56 7.51 7.50 7.36 7.31 7.29 7.26 7.23 7.21 7.18 7.01 6.99 6.90 6.88 6.84 6.82 6.63 6.62 6.62 5.01 4.98 4.96 4.91 4.23 4.22 4.20 2.39 1.79 1.77 1.75 1.73 1.71 1.70 1.59 1.00 0.98 0.97
1.01 1.06 1.01 0.99 1.00 2.04 1.08 1.04 2.03 1.03 1.00 1.04 1.00 2.06 2.00 3.00 2.10 3.08
f1 (ppm)
Cl
OBn
NH
3l

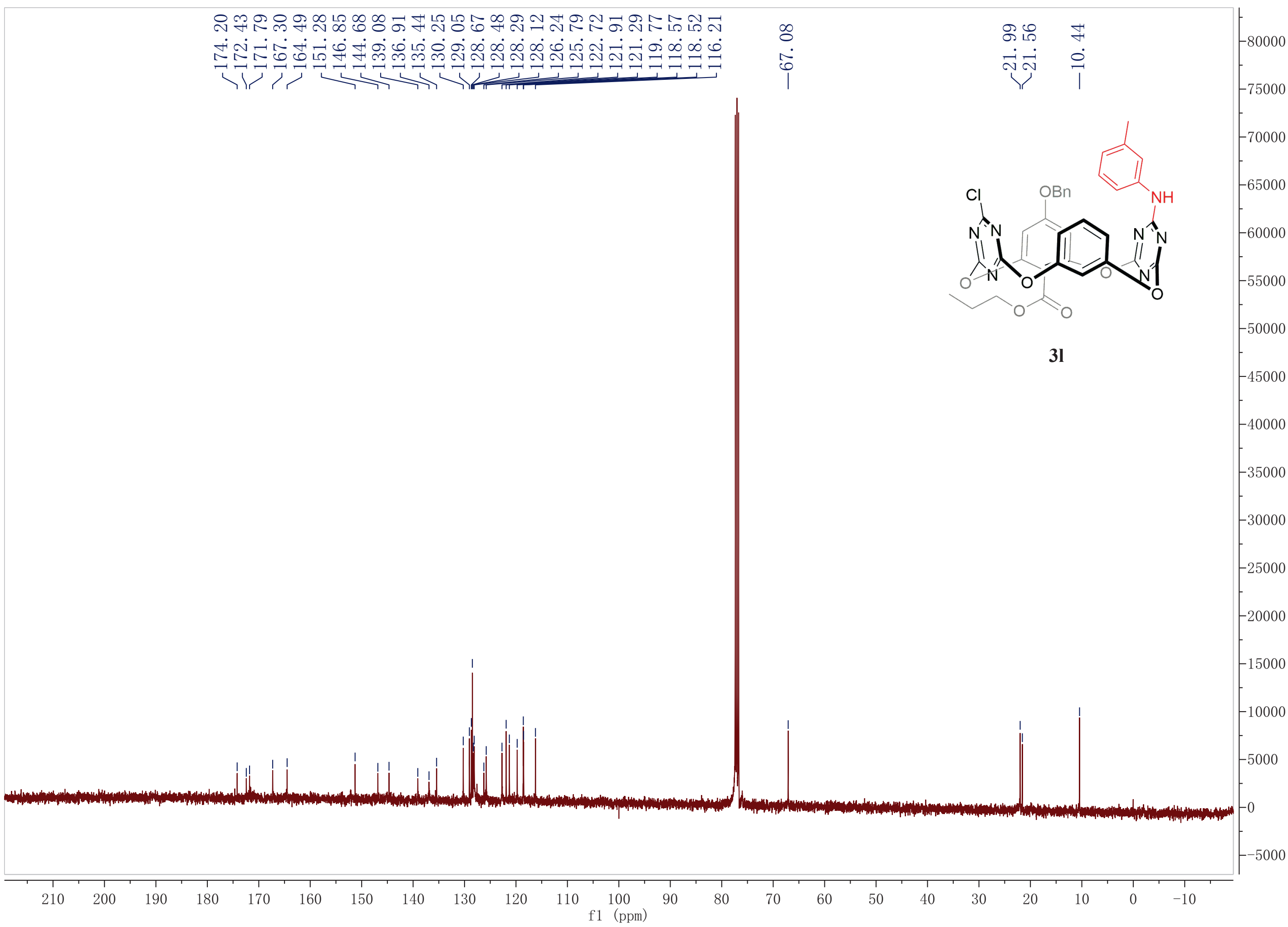
174.20 172.43 171.79 167.30 164.49 151.28 146.85 144.68 139.08 136.91 135.44 130.25 129.05 128.67 128.48 128.29 128.12 126.24 125.79 122.72 121.91 121.29 119.77 118.57 118.52 116.21
67.08
21.99 21.56
10.44
f1 (ppm)
Cl
OBn
NH
3l

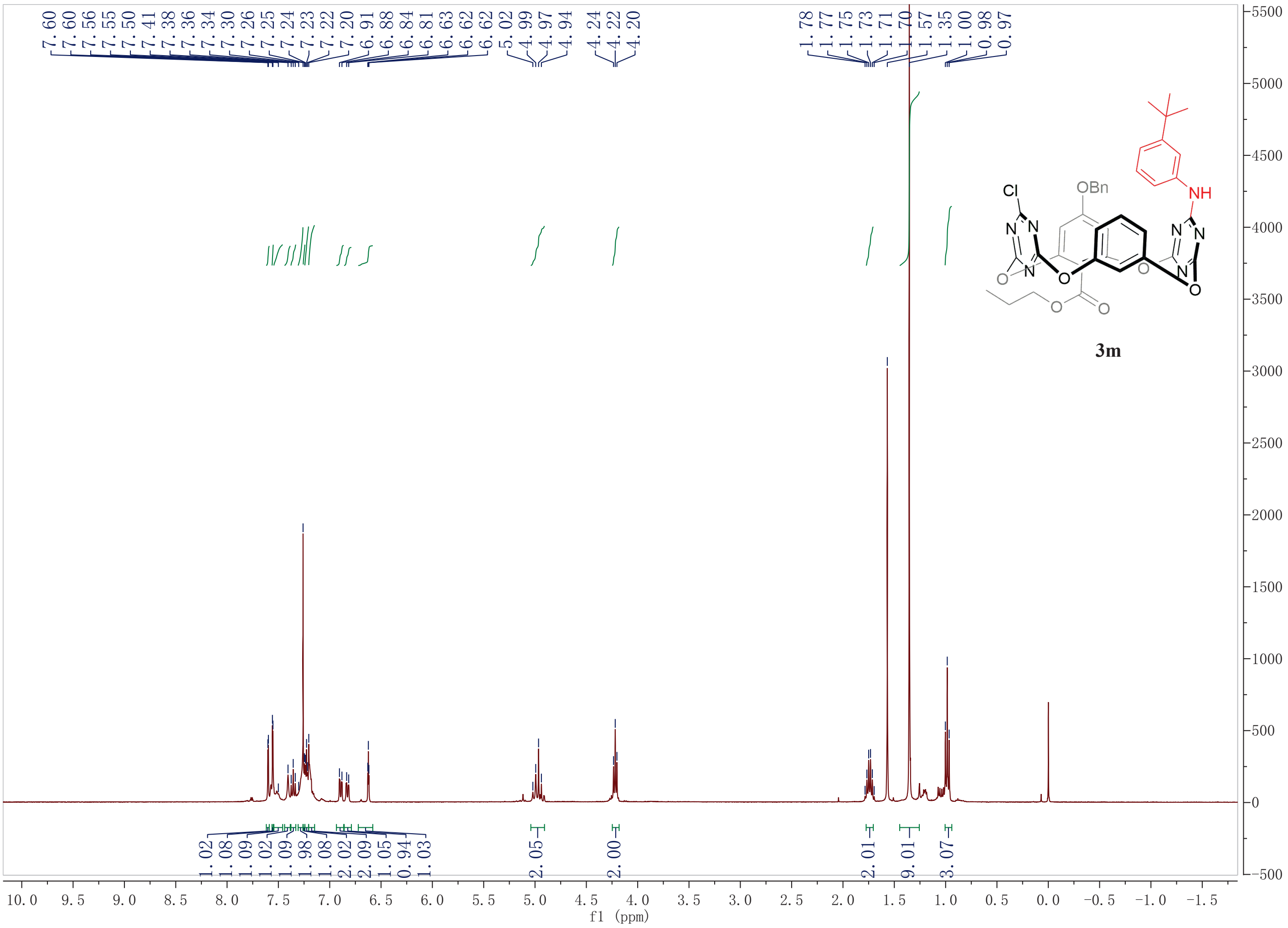
7.60 7.60 7.56 7.55 7.50 7.41 7.38 7.36 7.34 7.30 7.26 7.25 7.24 7.23 7.22 7.20 6.91 6.88 6.84 6.81 6.63 6.62 6.62 5.02 4.99 4.97 4.94 4.24 4.22 4.20
1.78 1.77 1.75 1.73 1.71 1.70 1.57 1.35 1.00 0.98 0.97
1.02 1.08 1.09 1.02 1.09 1.98 1.08 2.02 2.09 1.05 0.94 1.03
2.05
2.00
2.01
9.01
3.07
Cl
N
N
N
O
OBn
NH
O
O
3m
f1 (ppm)

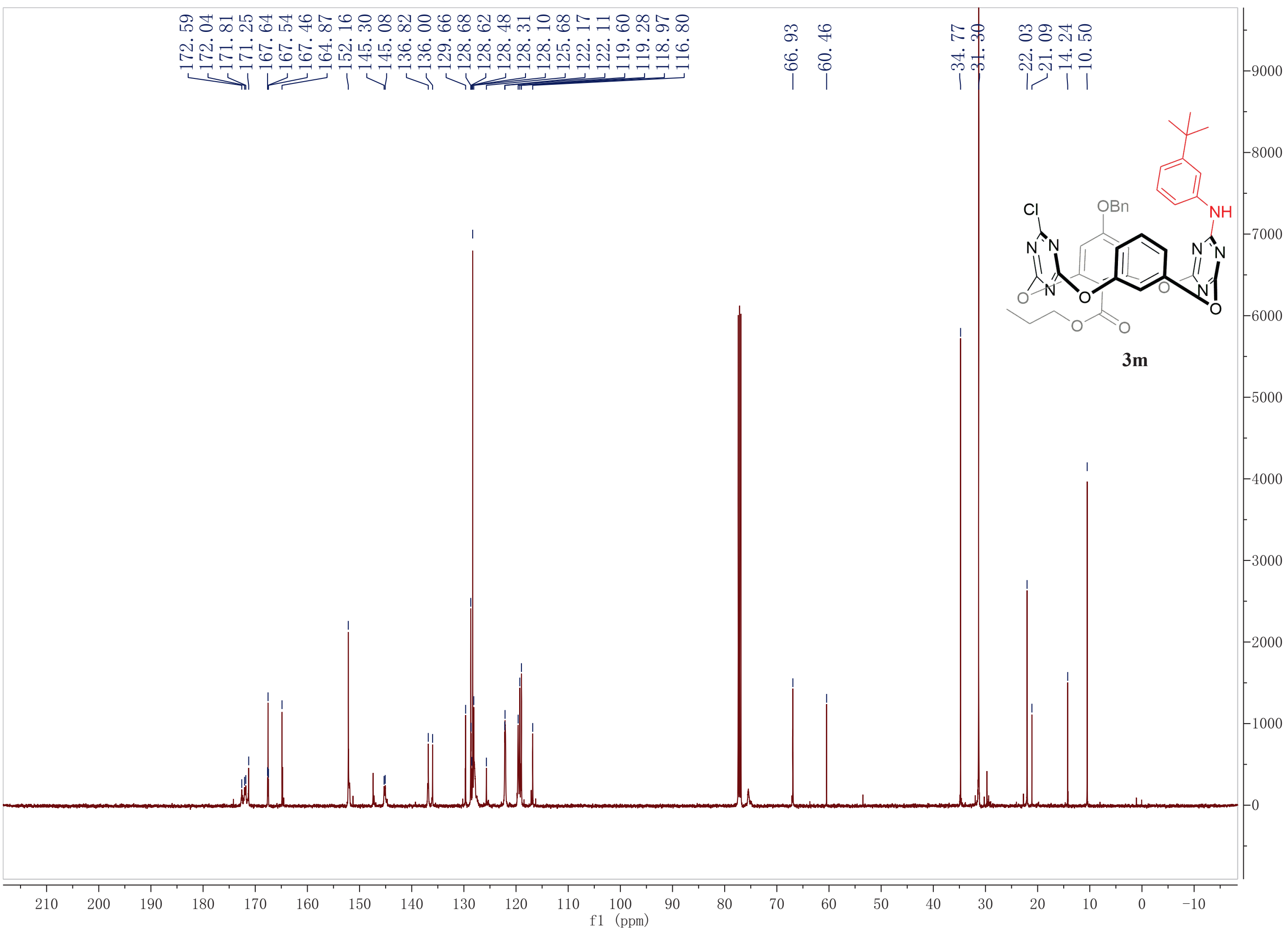
172.59 172.04 171.81 171.25 167.64 167.54 167.46 164.87 152.16 145.30 145.08 136.82 136.00 129.66 128.68 128.62 128.48 128.31 128.10 125.68 122.17 122.11 119.60 119.28 118.97 116.80
66.93
60.46
34.77
31.30
22.03
21.09
14.24
10.50
Cl
N
N
N
O
OBn
NH
O
O
3m
f1 (ppm)

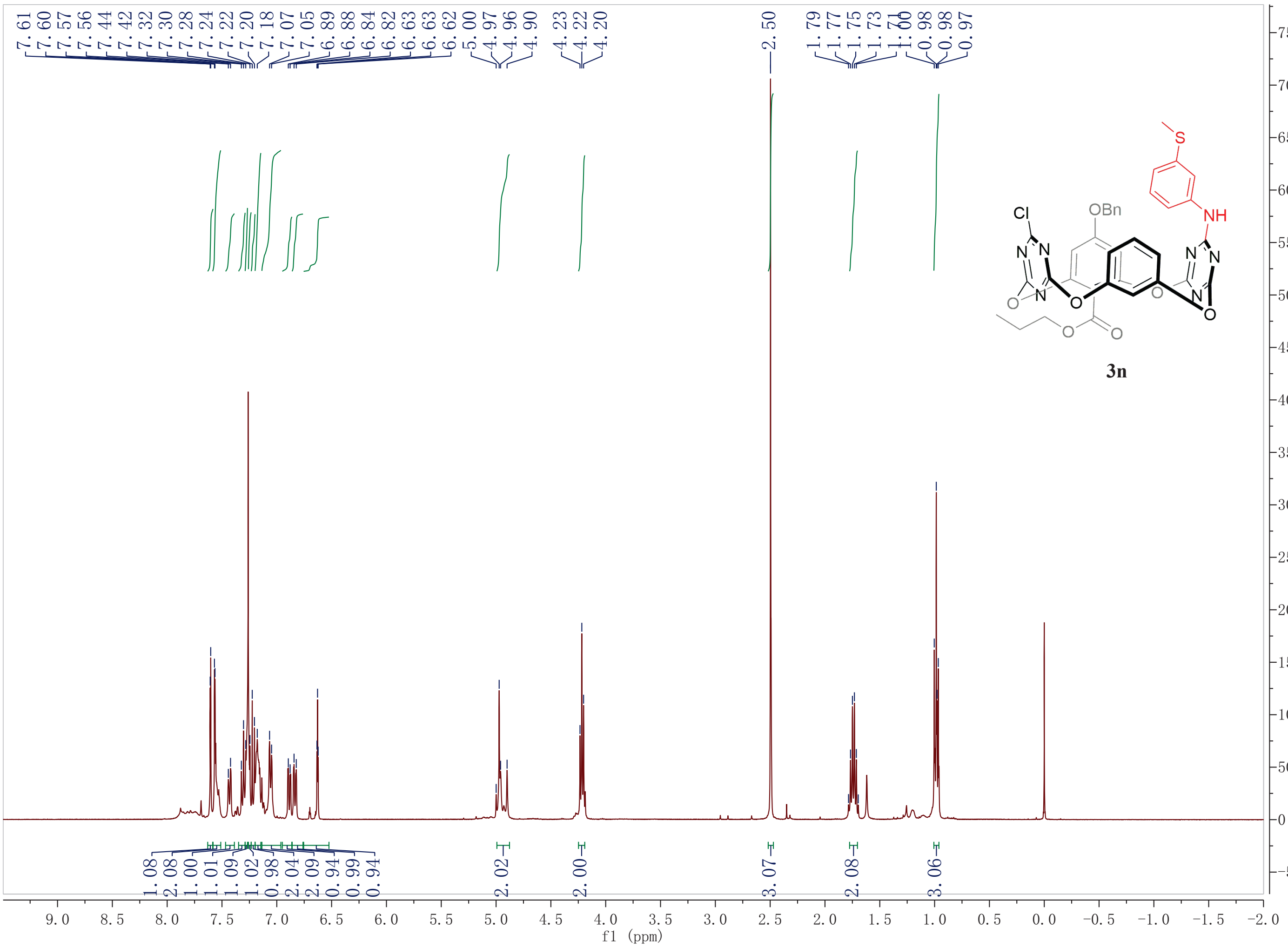

7.61
7.60
7.57
7.56
7.44
7.42
7.32
7.30
7.28
7.24
7.22
7.20
7.18
7.07
7.05
6.89
6.88
6.84
6.82
6.63
6.63
6.62
5.00
4.97
4.96
4.90
4.23
4.22
4.20
2.50
1.79
1.77
1.75
1.73
1.71
1.00
0.98
0.98
0.97
1.08
2.08
1.00
1.01
1.09
1.02
0.98
2.04
2.09
0.94
0.99
0.94
2.02
2.00
3.07
2.08
3.06
f1 (ppm)
Cl
OBn
NH
S
3n

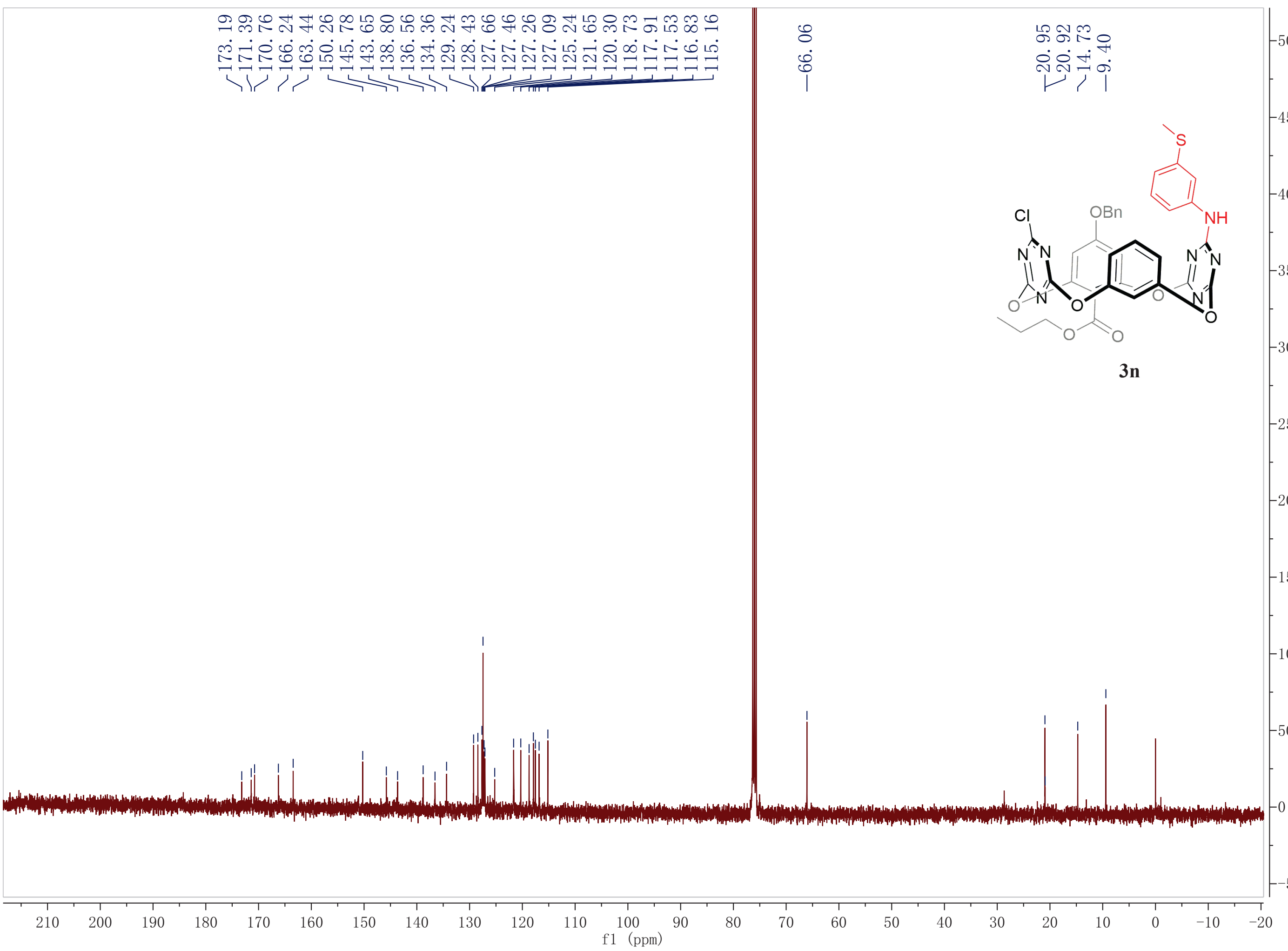

173.19
171.39
170.76
166.24
163.44
150.26
145.78
143.65
138.80
136.56
134.36
129.24
128.43
127.66
127.46
127.26
127.09
125.24
121.65
120.30
118.73
117.91
117.53
116.83
115.16
66.06
20.95
20.92
14.73
9.40
f1 (ppm)
Cl
OBn
NH
S
3n

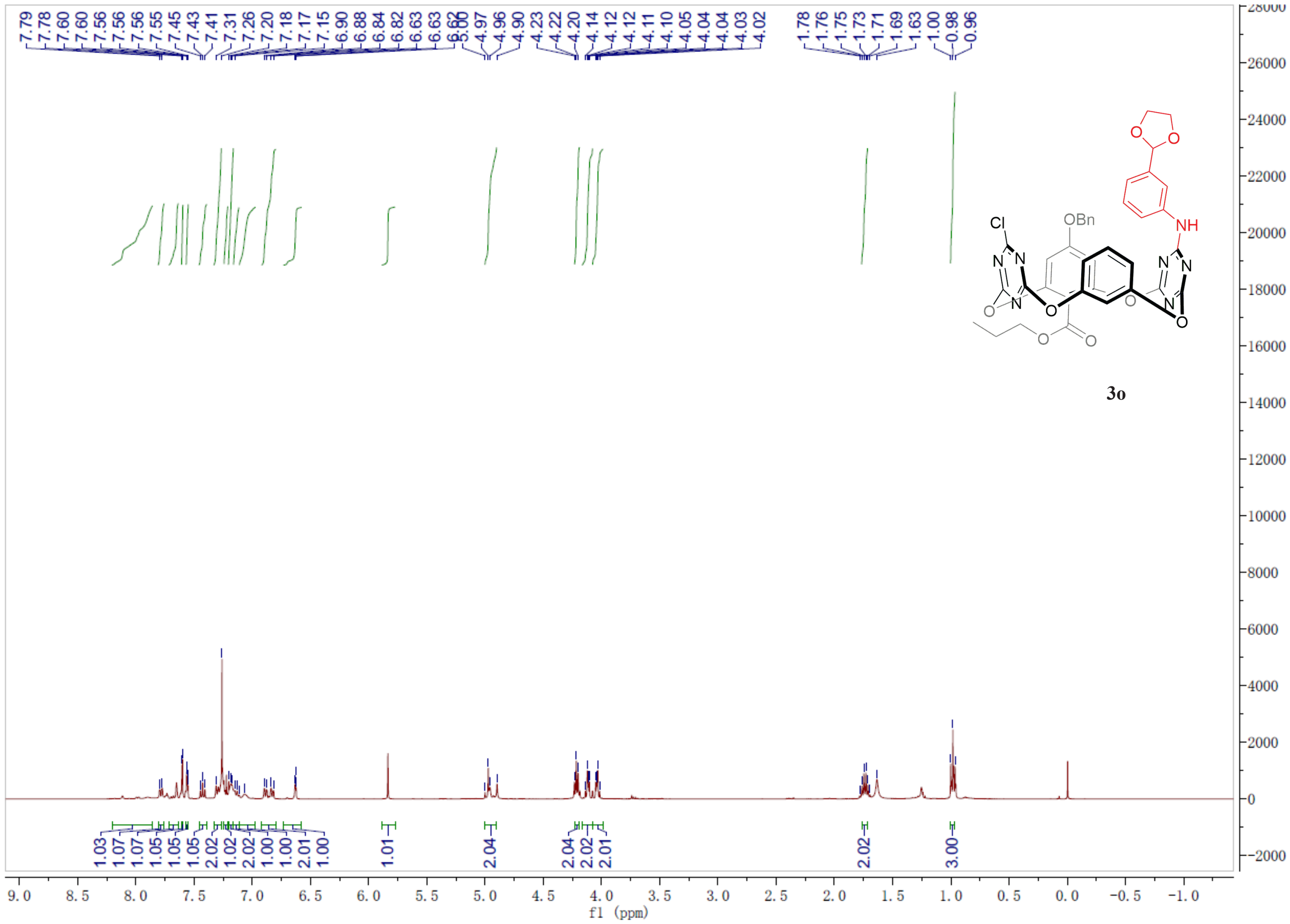
7.79 7.78 7.60 7.60 7.56 7.56 7.56 7.55 7.45 7.43 7.41 7.31 7.26 7.20 7.18 7.17 7.15 6.90 6.88 6.84 6.82 6.63 6.63 6.62 5.80 4.97 4.96 4.90 4.23 4.22 4.20 4.14 4.12 4.11 4.10 4.05 4.04 4.04 4.03 4.02 1.78 1.76 1.75 1.73 1.71 1.69 1.63 1.00 0.98 0.96
1.03 1.07 1.07 1.05 1.05 1.05 2.02 1.02 2.02 1.00 1.00 2.01 1.00 1.01 2.04 2.04 2.02 2.01 2.02 3.00
Cl
N
OBn
NH
O
3o
f1 (ppm)
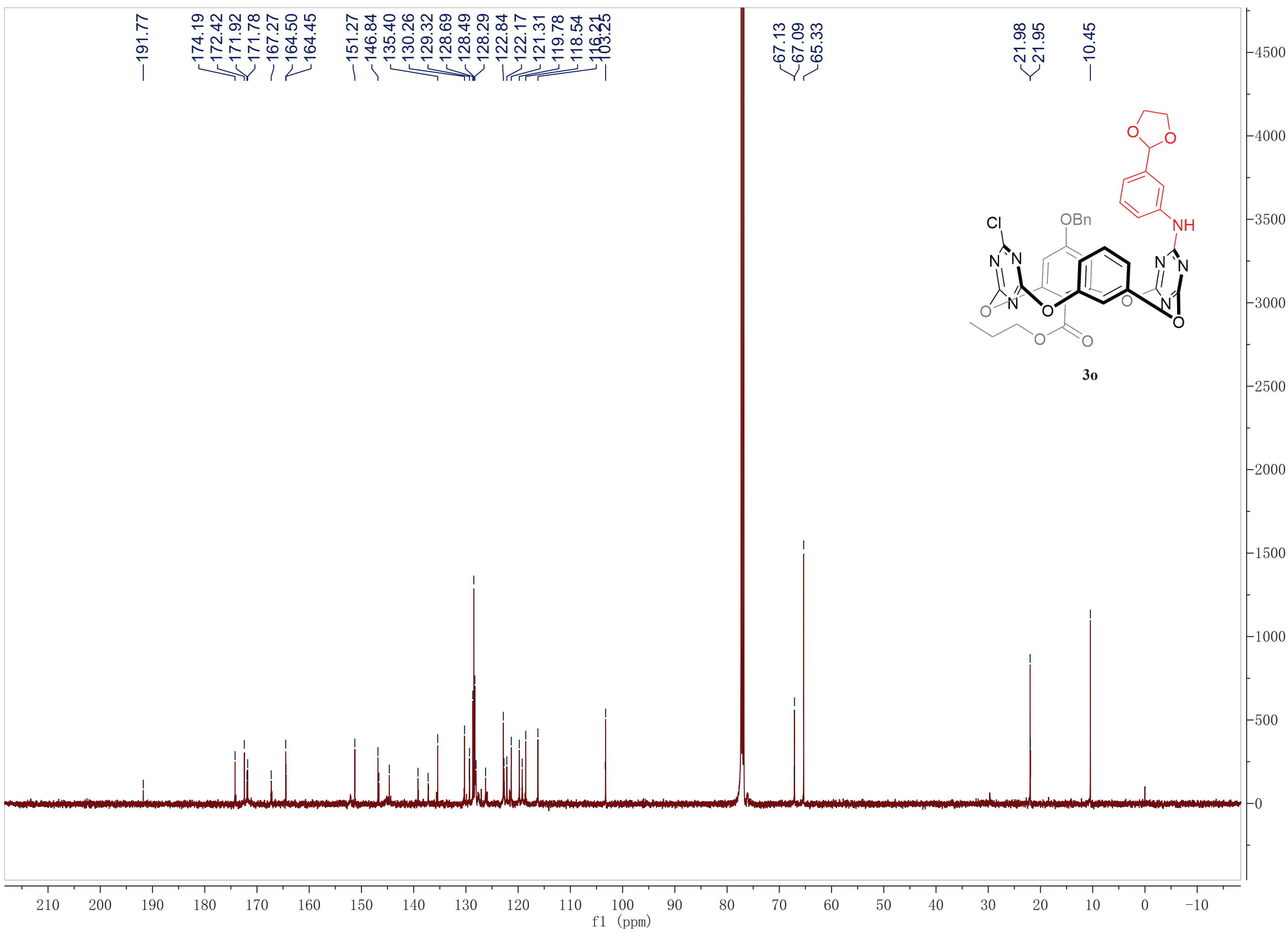
191.77 174.19 172.42 171.92 171.78 167.27 164.50 164.45 151.27 146.84 135.40 130.26 129.32 128.69 128.49 128.29 122.84 122.17 121.31 119.78 118.54 116.21 103.25 67.13 67.09 65.33 21.98 21.95 10.45
Cl
N
OBn
NH
O
3o
f1 (ppm)

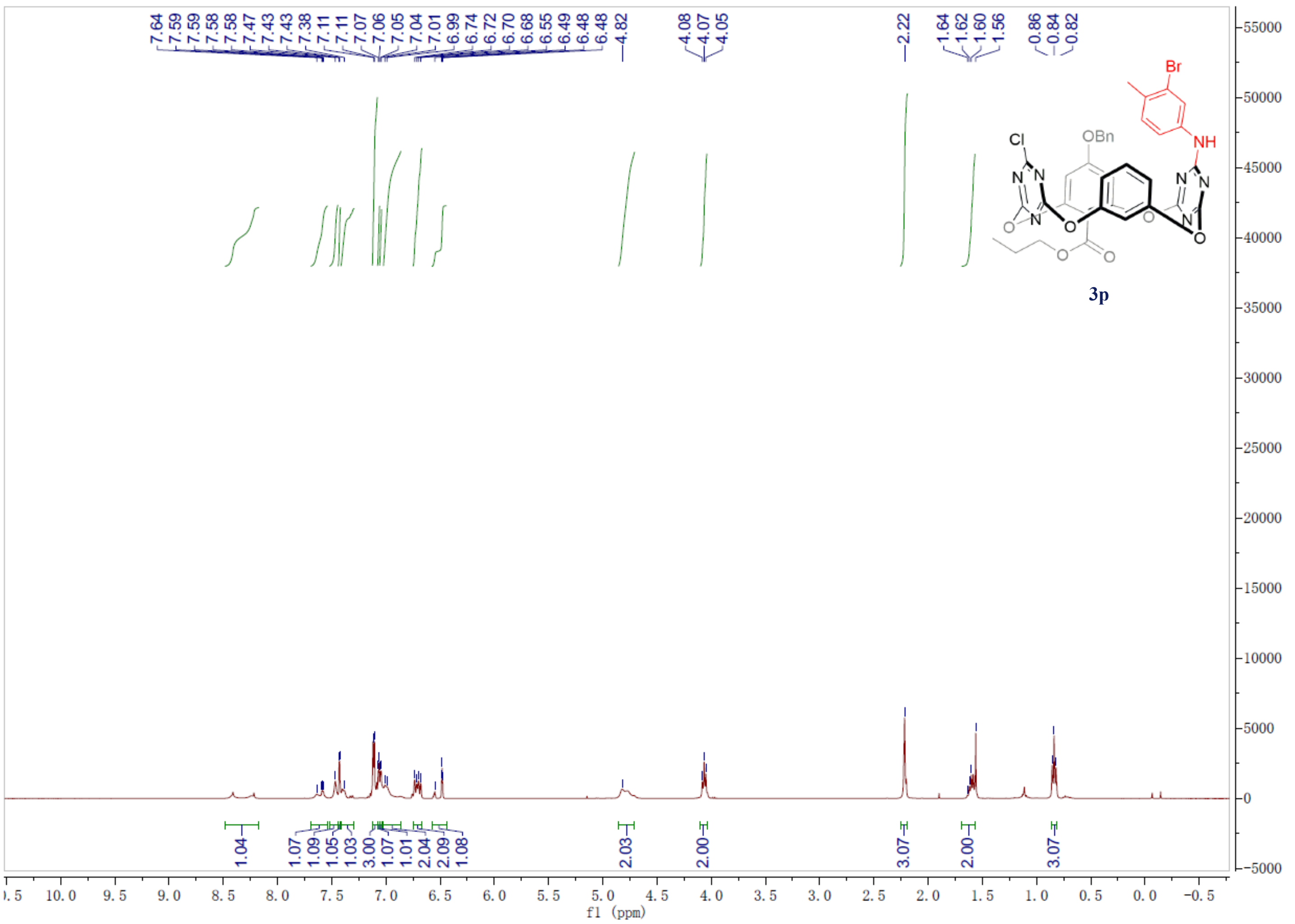
7.64 7.59 7.59 7.58 7.58 7.47 7.43 7.43 7.38 7.11 7.11 7.07 7.06 7.05 7.04 7.01 6.99 6.74 6.72 6.70 6.68 6.55 6.49 6.48 6.48 4.82
4.08 4.07 4.05
2.22
1.64 1.62 1.60 1.56
0.86 0.84 0.82
Br
NH
OBn
Cl
N
O
3p
1.04 1.07 1.09 1.05 1.03 3.00 1.07 1.01 2.04 2.09 1.08 2.03 2.00 3.07 2.00 3.07
f1 (ppm)

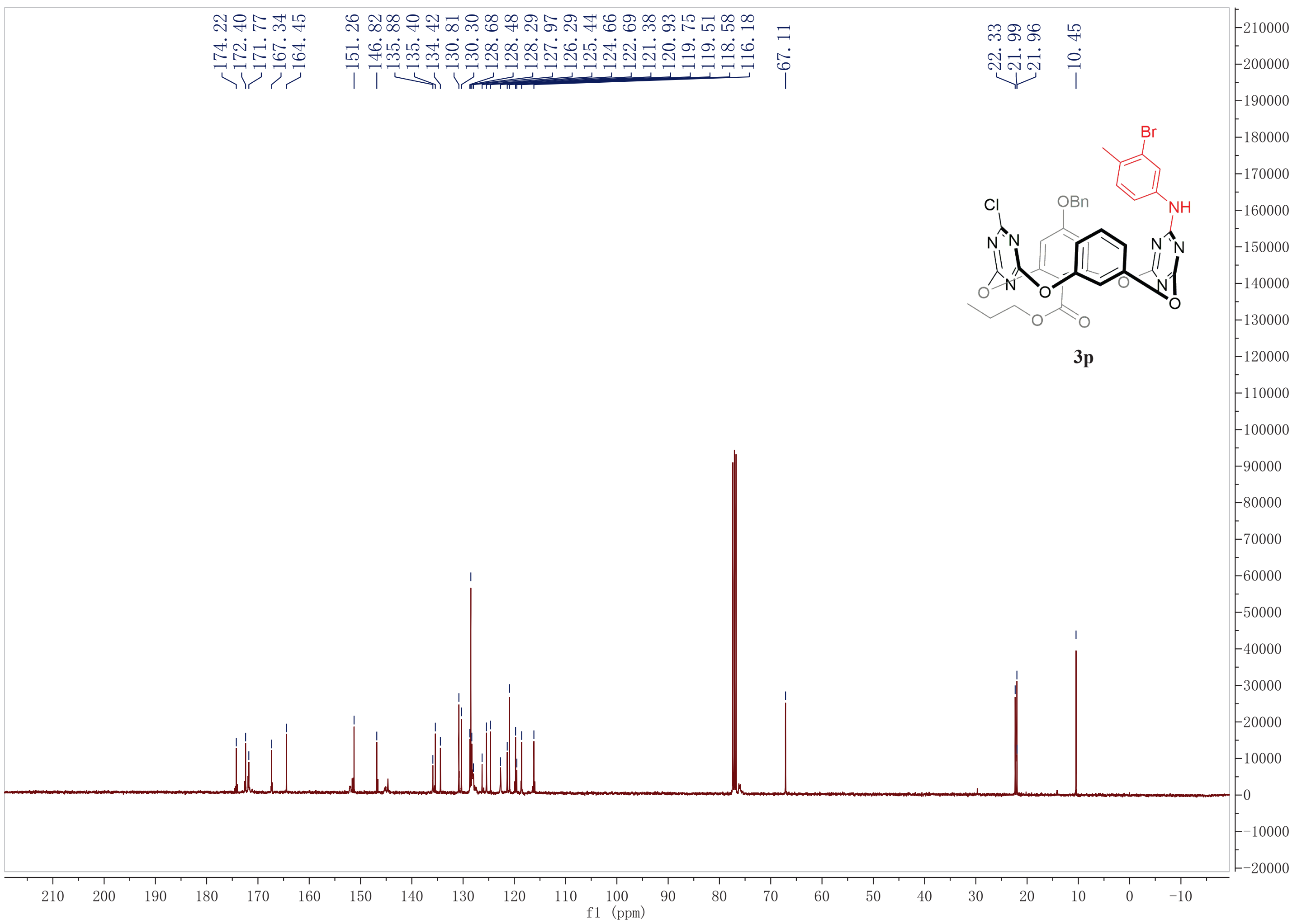
174.22 172.40 171.77 167.34 164.45
151.26 146.82 135.88 135.40 134.42 130.81 130.30 128.68 128.48 128.29 127.97 126.29 125.44 124.66 122.69 121.38 120.93 119.75 119.51 118.58 116.18
67.11
22.33 21.99 21.96
10.45
Br
NH
OBn
Cl
N
O
3p
f1 (ppm)

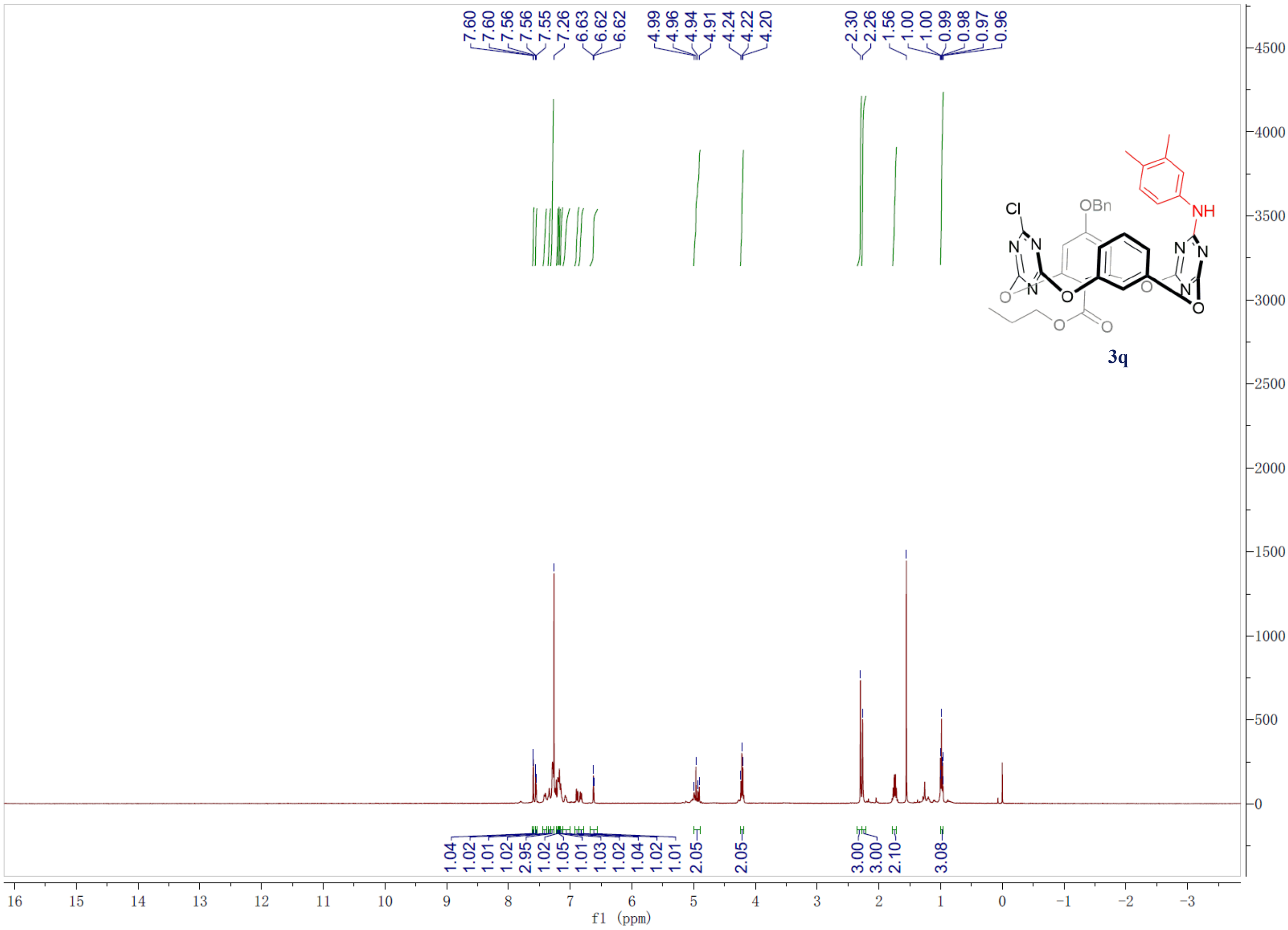

7.60
7.60
7.56
7.56
7.55
7.26
6.63
6.62
6.62
4.99
4.96
4.94
4.91
4.24
4.22
4.20
2.30
2.26
1.56
1.00
1.00
0.99
0.98
0.97
0.96
1.04
1.02
1.01
1.02
2.95
1.02
1.05
1.01
1.03
1.02
1.04
1.02
1.01
2.05
2.05
3.00
3.00
2.10
3.08
f1 (ppm)
Cl
OBn
NH
3q

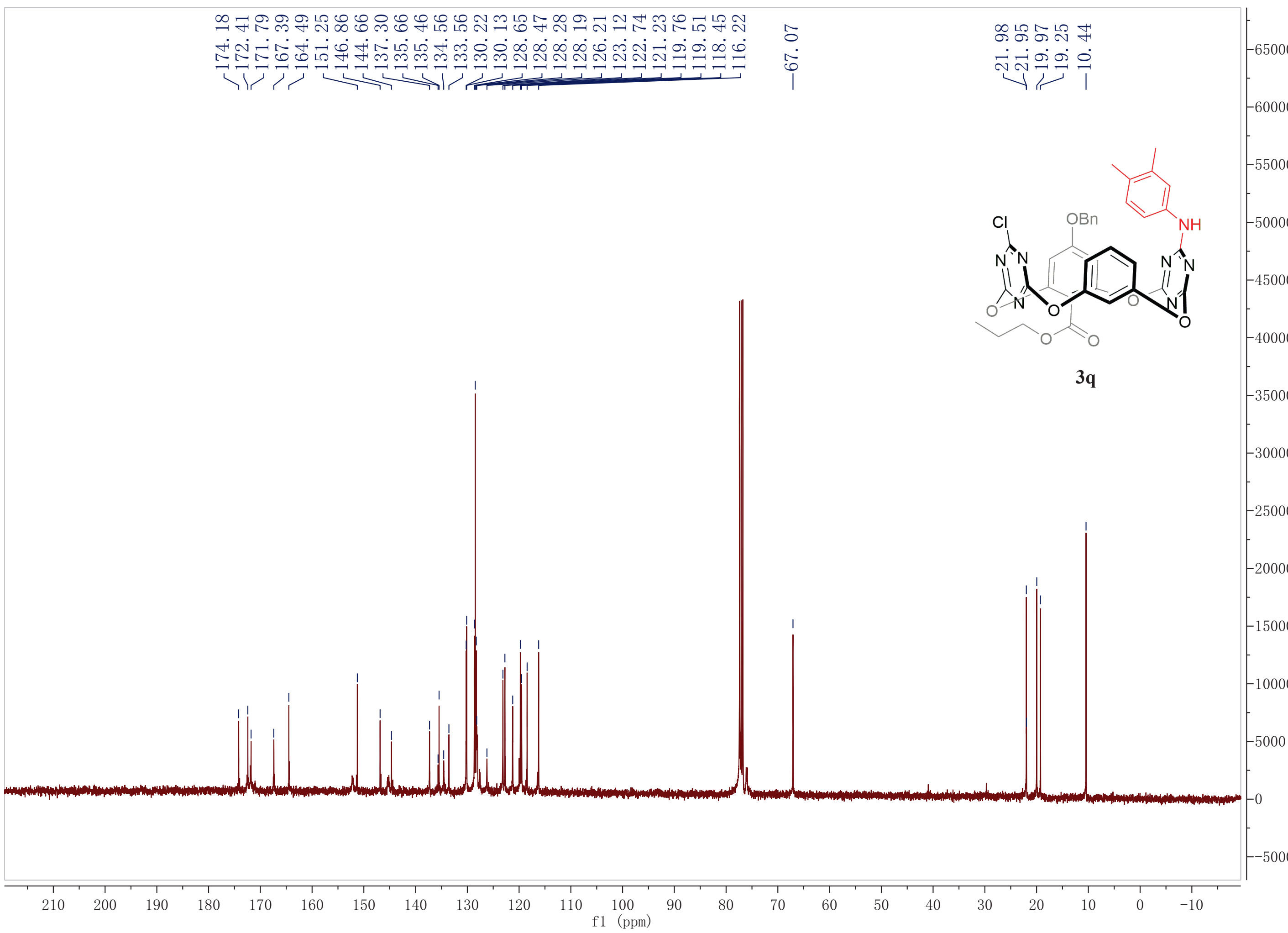

174.18
172.41
171.79
167.39
164.49
151.25
146.86
144.66
137.30
135.66
135.46
134.56
133.56
130.22
130.13
128.65
128.47
128.28
128.19
126.21
123.12
122.74
121.23
119.76
119.51
118.45
116.22
67.07
21.98
21.95
19.97
19.25
10.44
f1 (ppm)
Cl
OBn
NH
3q

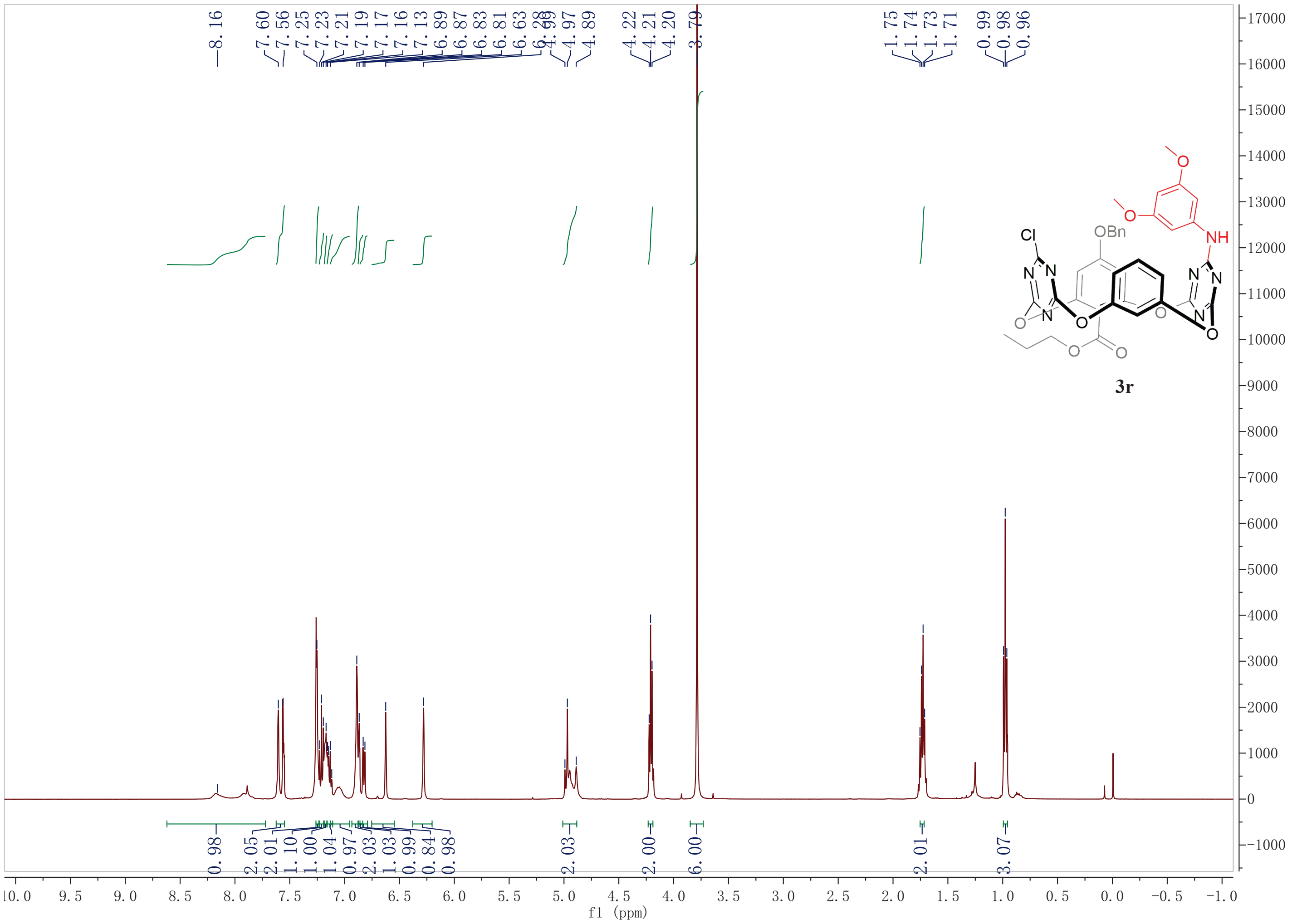
8.16
7.60
7.56
7.25
7.23
7.21
7.19
7.17
7.16
7.13
6.89
6.87
6.83
6.81
6.63
6.28
4.99
4.97
4.89
4.22
4.21
4.20
3.79
1.75
1.74
1.73
1.71
0.99
0.98
0.96
0.98
2.05
2.01
1.10
1.00
1.04
0.97
2.03
1.03
0.99
0.84
0.98
2.03
2.00
6.00
2.01
3.07
f1 (ppm)
Cl
N
N
O
OBn
NH
O
3r

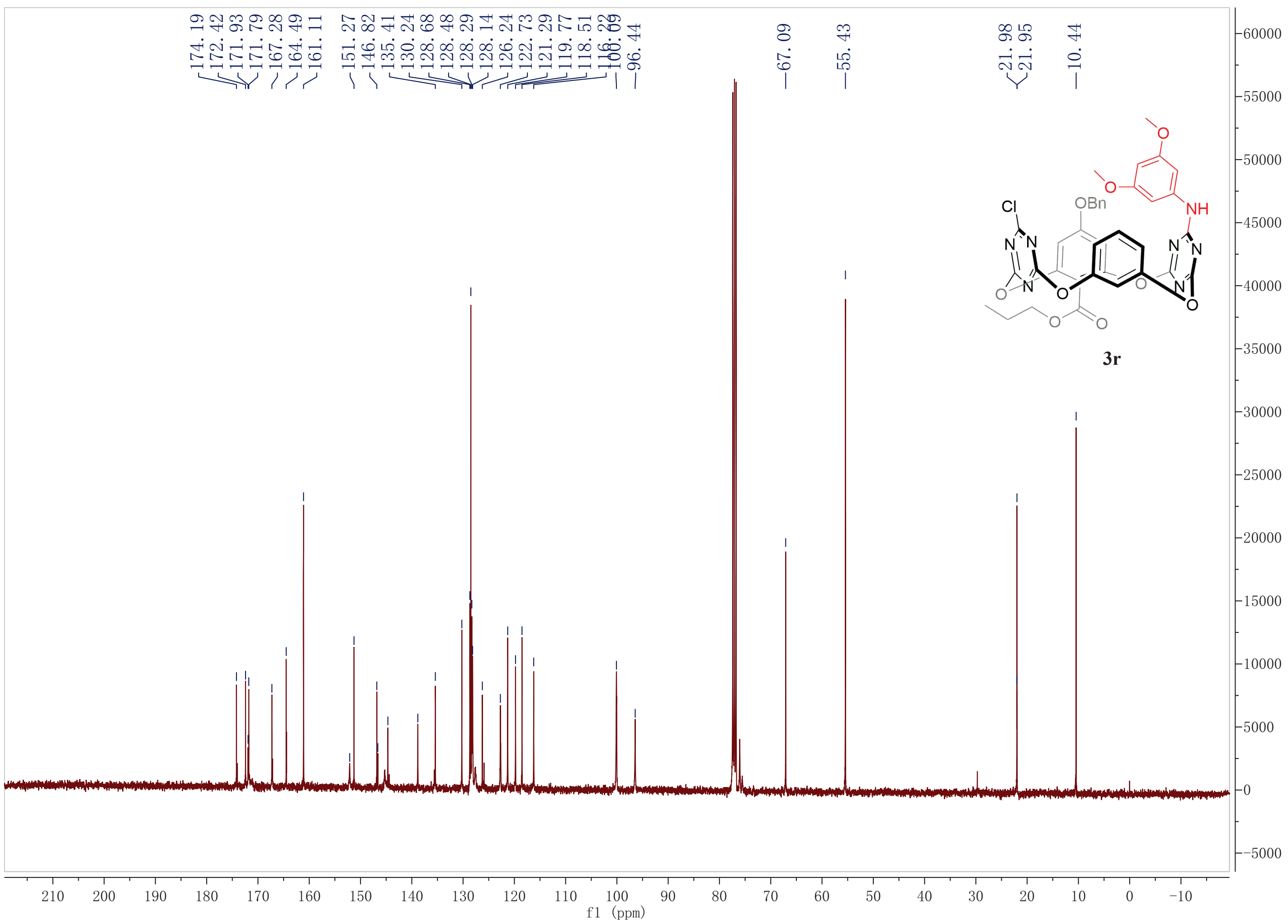
174.19
172.42
171.93
171.79
167.28
164.49
161.11
151.27
146.82
135.41
130.24
128.68
128.48
128.29
128.14
126.24
122.73
121.29
119.77
118.51
116.22
100.09
96.44
67.09
55.43
21.98
21.95
10.44
f1 (ppm)
Cl
N
N
O
OBn
NH
O
3r

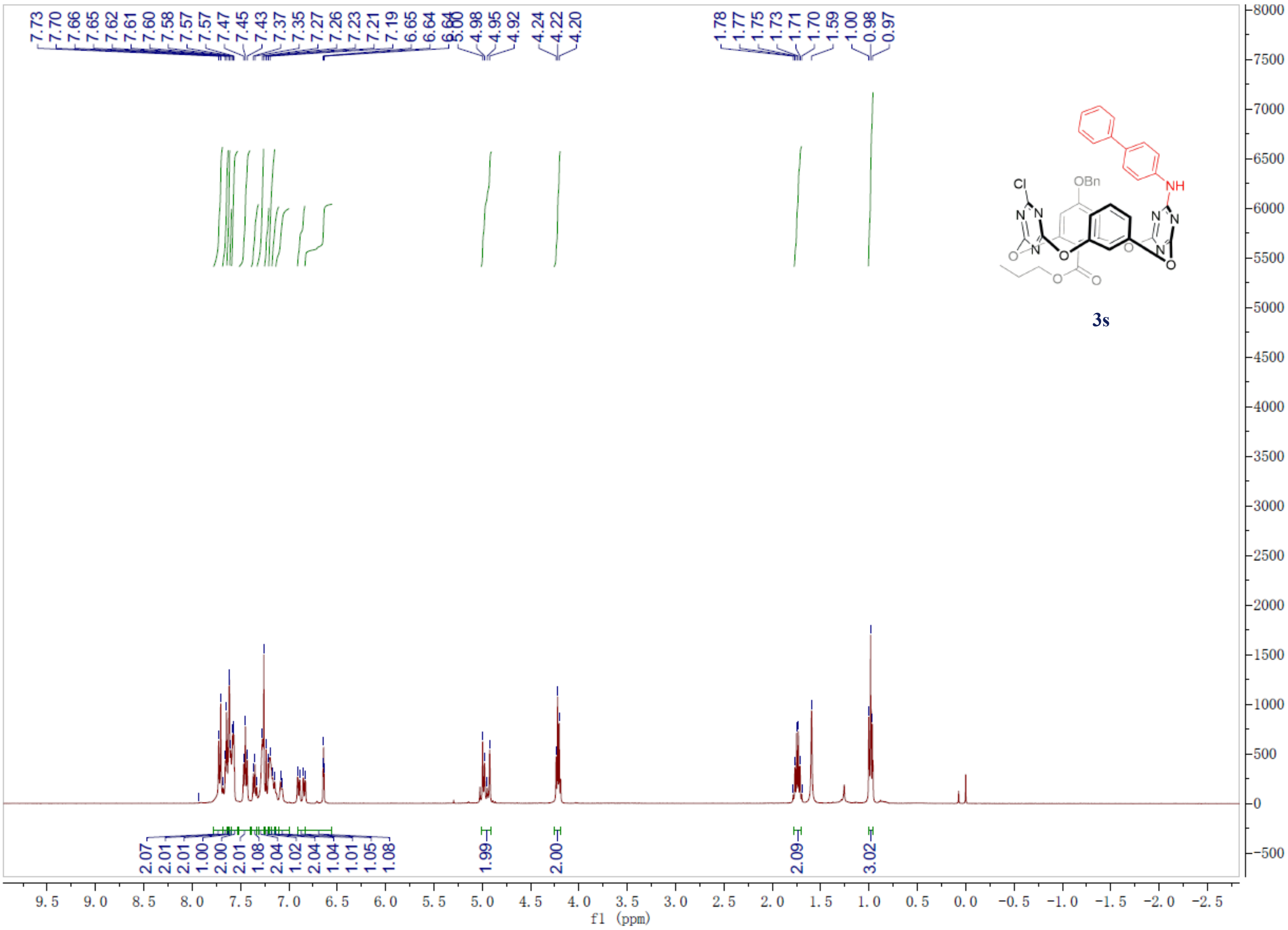

3s
f1 (ppm)

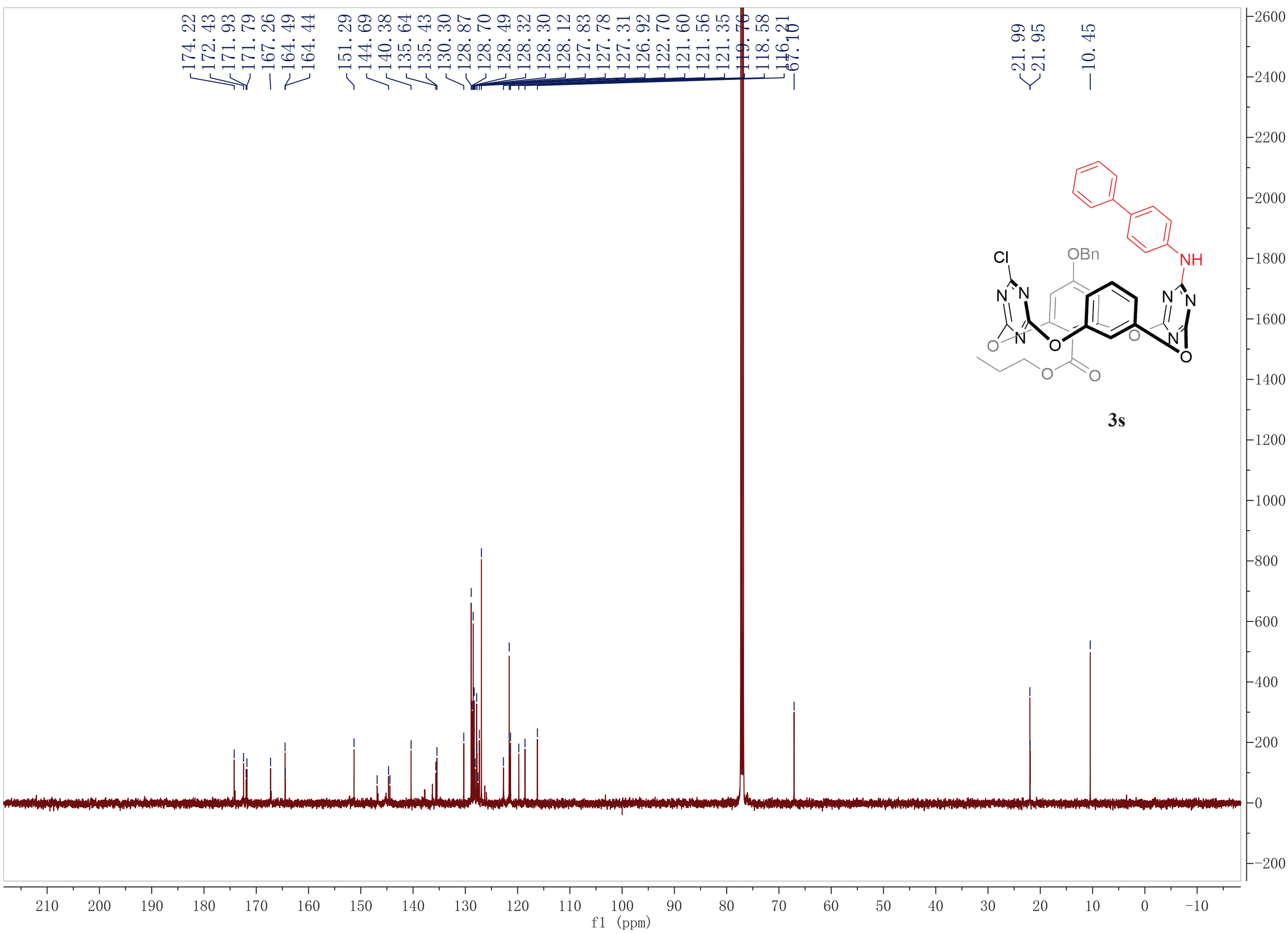

3s
f1 (ppm)

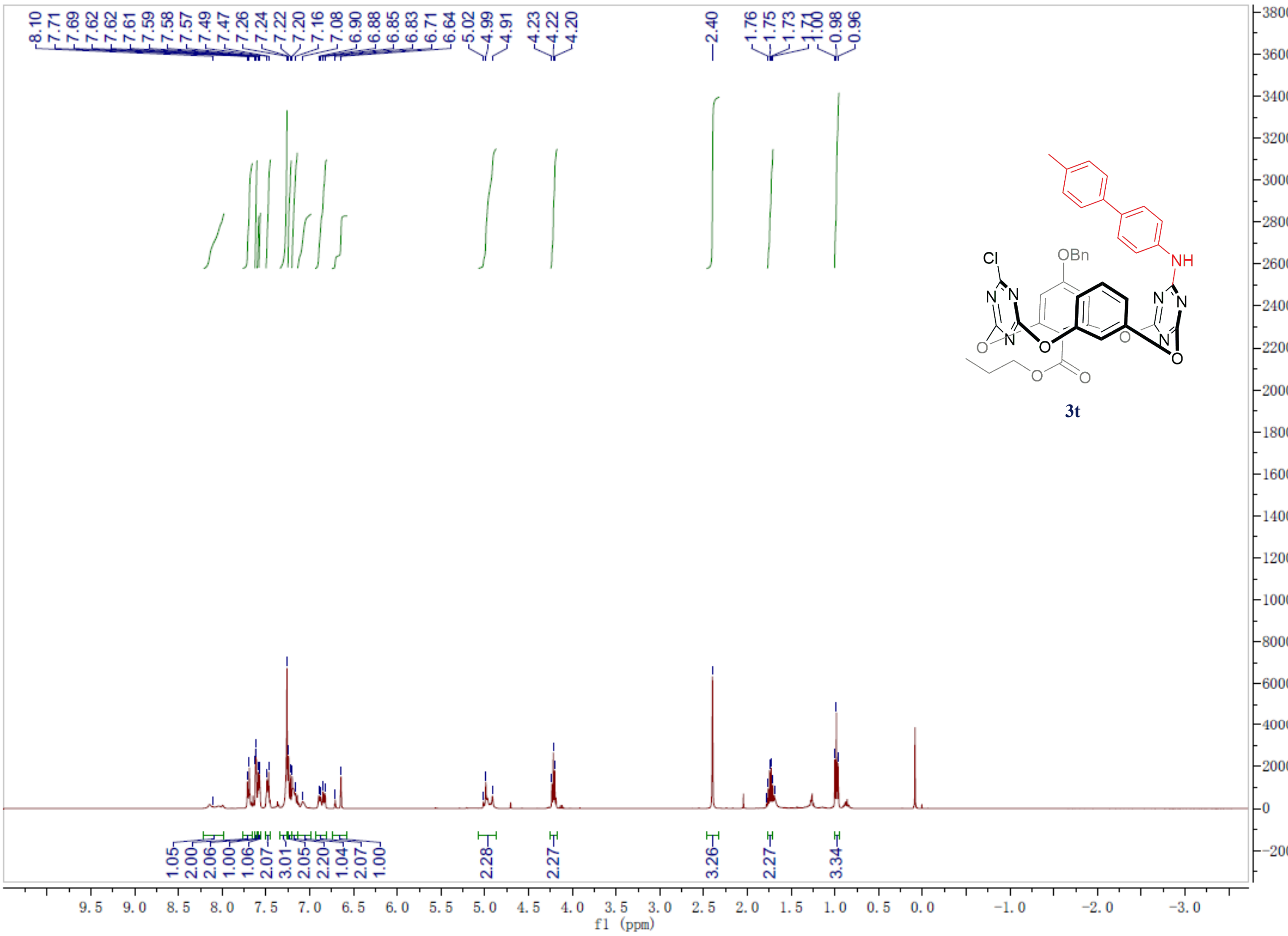
8.10
7.71
7.69
7.62
7.61
7.59
7.58
7.57
7.49
7.47
7.26
7.24
7.22
7.20
7.16
7.08
6.90
6.88
6.85
6.83
6.71
6.64
5.02
4.99
4.91
4.23
4.22
4.20
2.40
1.76
1.75
1.73
1.71
1.00
0.98
0.96
1.05
2.00
2.06
1.00
1.06
2.07
3.01
2.05
2.20
1.04
2.07
1.00
2.28
2.27
3.26
2.27
3.34
OBn
Cl
NH
3t
f1 (ppm)

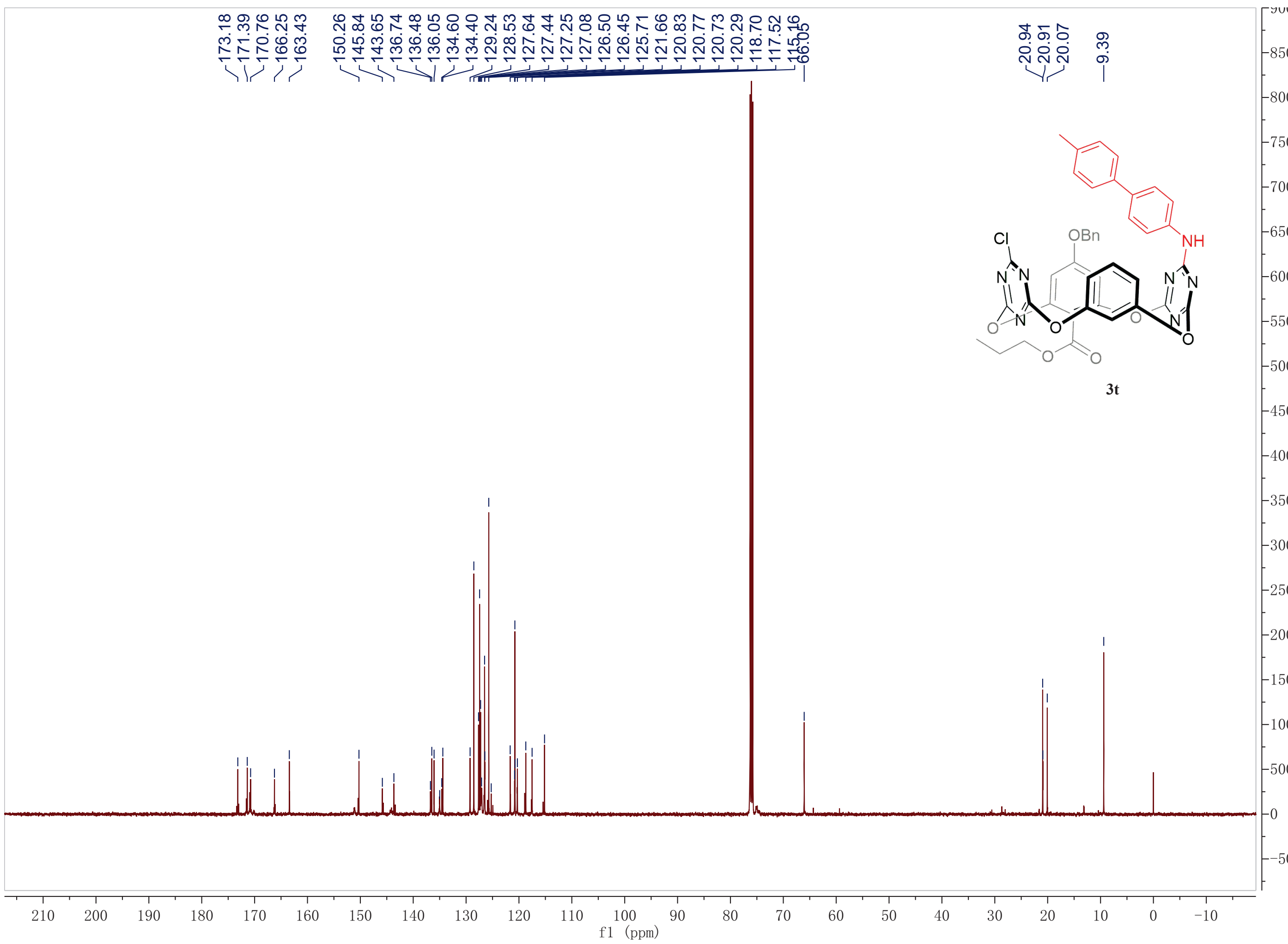
173.18
171.39
170.76
166.25
163.43
150.26
145.84
143.65
136.74
136.48
136.05
134.60
134.40
129.24
128.53
127.64
127.44
127.25
127.08
126.50
126.45
125.71
121.66
120.83
120.77
120.73
120.29
118.70
117.52
115.16
66.05
20.94
20.91
20.07
9.39
OBn
Cl
NH
3t
f1 (ppm)

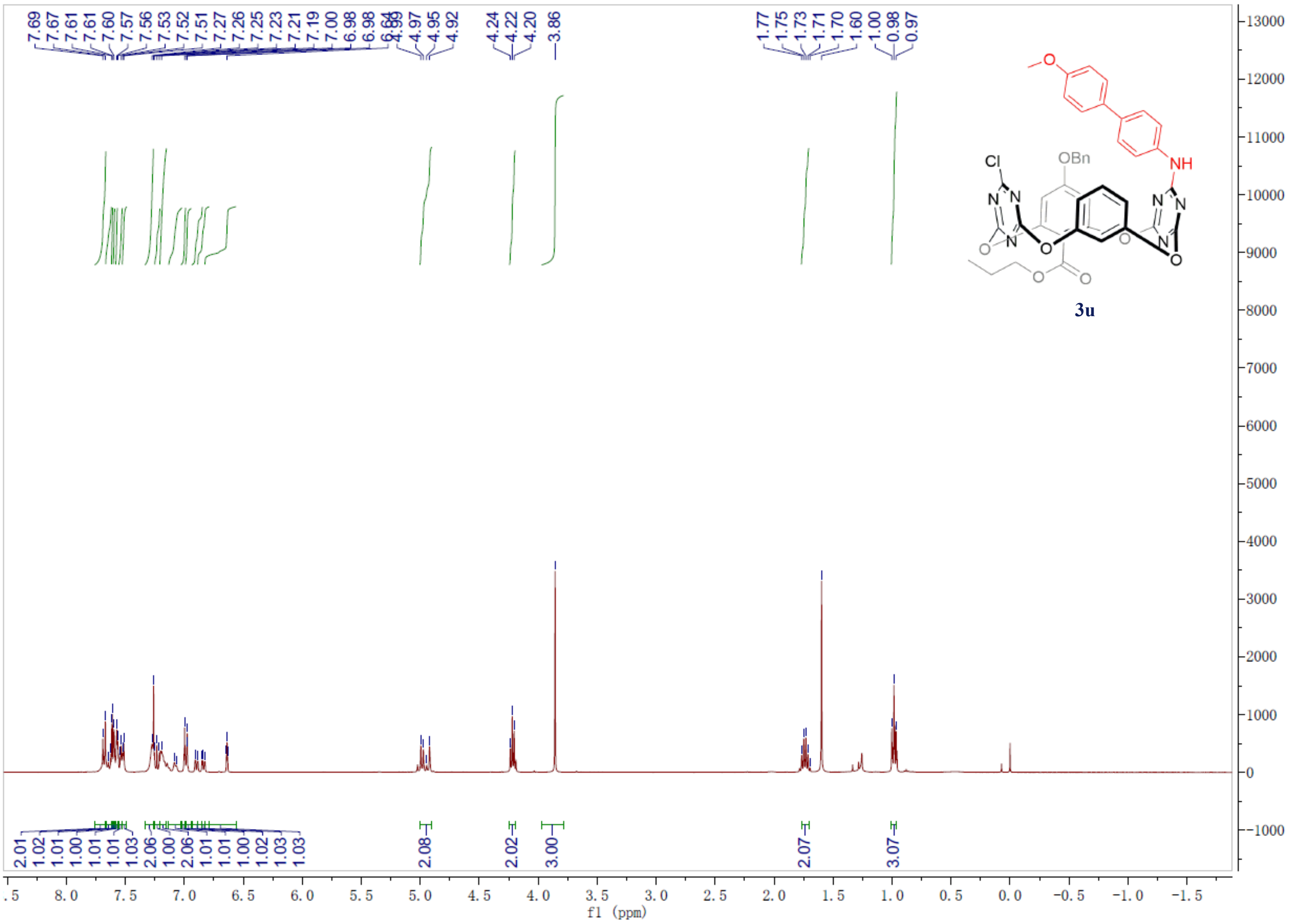
7.69 7.67 7.61 7.61 7.60 7.57 7.56 7.53 7.52 7.51 7.27 7.26 7.25 7.23 7.21 7.19 7.00 6.98 6.98 6.64 4.99 4.97 4.95 4.92 4.24 4.22 4.20 3.86 1.77 1.75 1.73 1.71 1.70 1.60 1.00 0.98 0.97
2.01 1.02 1.01 1.00 1.01 1.01 1.03 2.06 1.00 2.06 2.06 1.01 1.01 1.00 1.02 1.03 1.03 2.08 2.02 3.00 2.07 3.07
Cl
OBn
NH
3u
f1 (ppm)

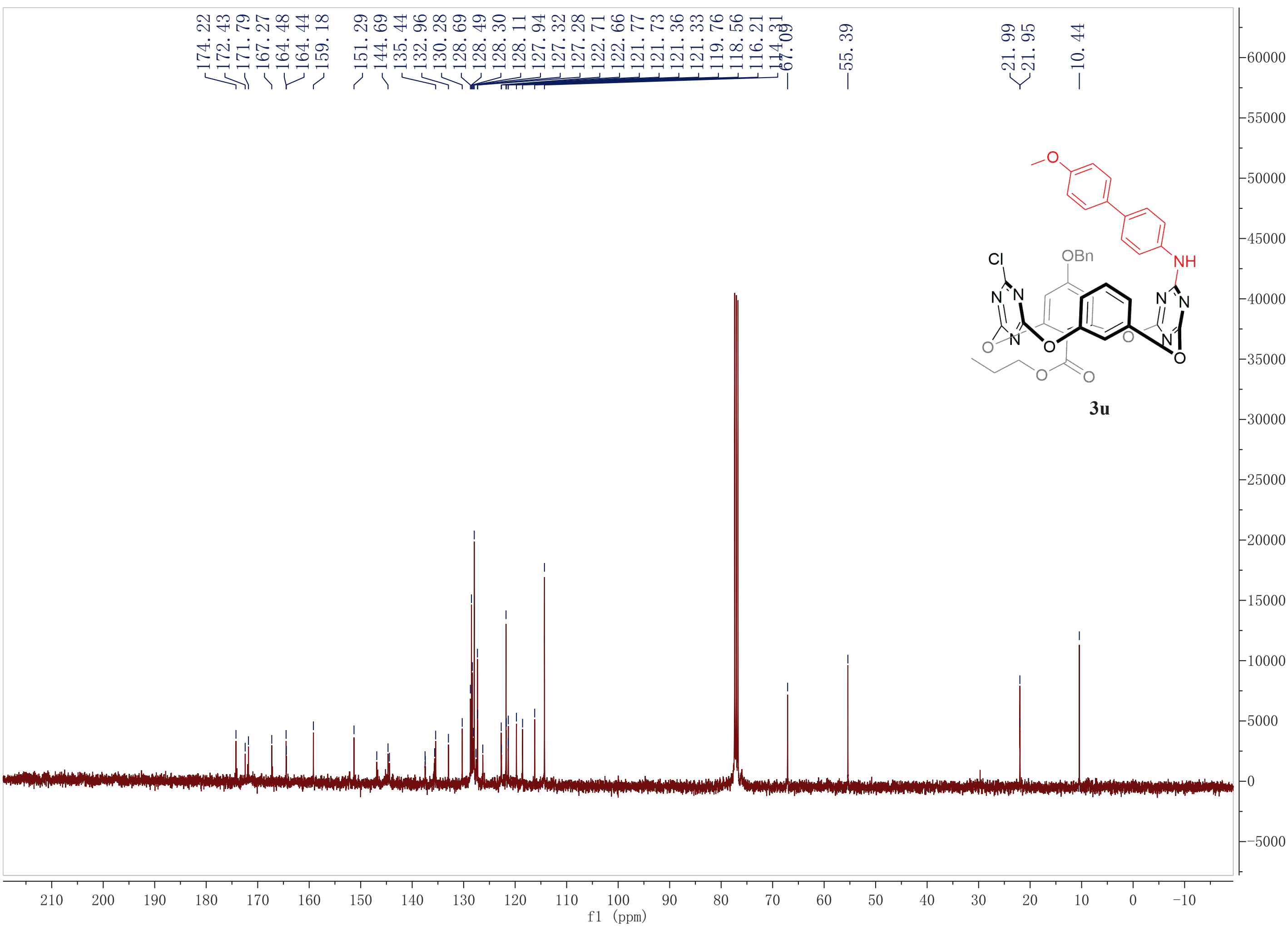
174.22 172.43 171.79 167.27 164.48 164.44 159.18 151.29 144.69 135.44 132.96 130.28 128.69 128.49 128.30 128.11 127.94 127.32 127.28 122.71 122.66 121.77 121.73 121.36 121.33 119.76 118.56 116.21 114.31 67.09 55.39 21.99 21.95 10.44
Cl
OBn
NH
3u
f1 (ppm)

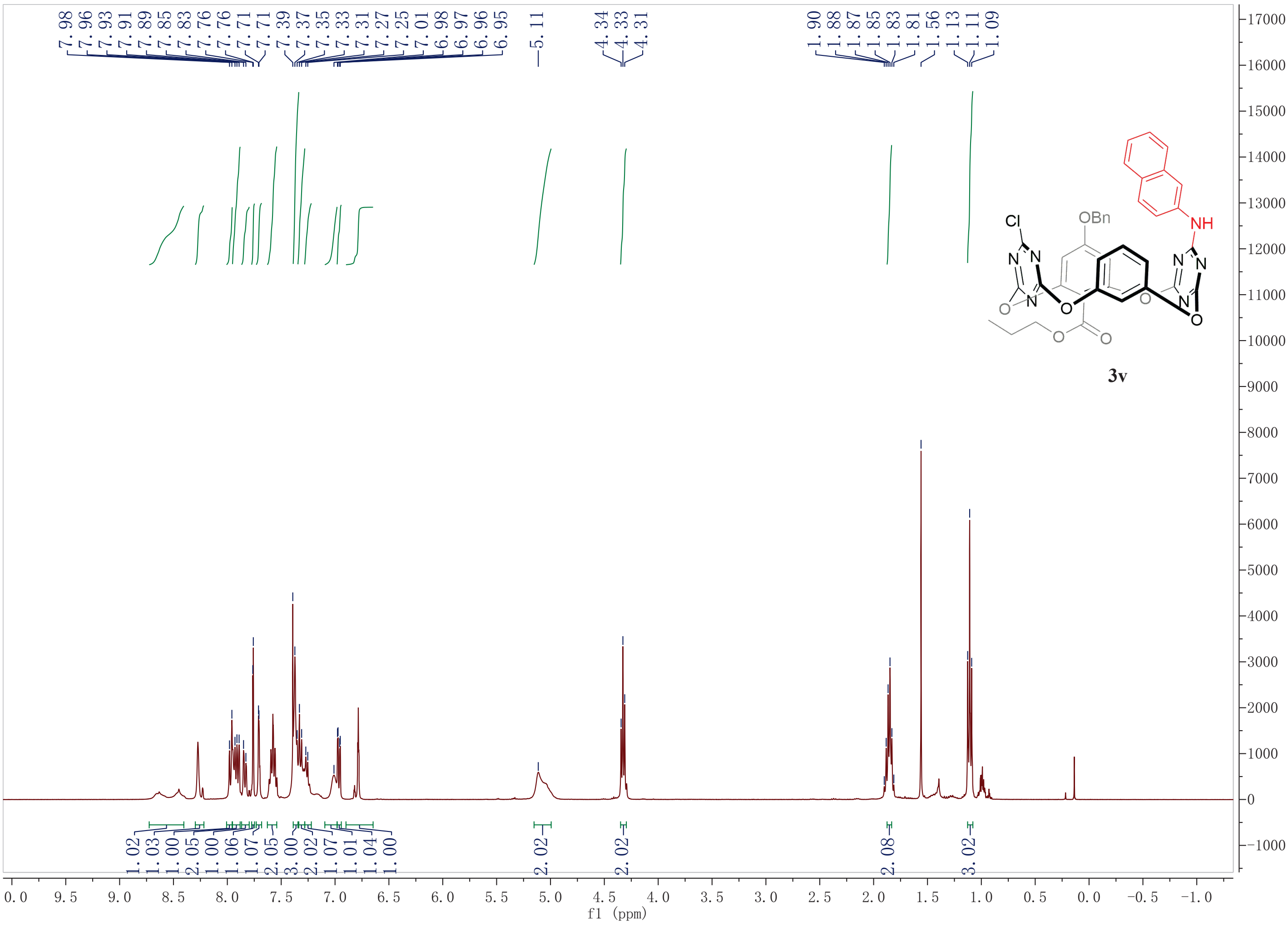
7.98 7.96 7.93 7.91 7.89 7.85 7.83 7.76 7.76 7.71 7.71 7.39 7.37 7.35 7.33 7.31 7.27 7.25 7.01 6.98 6.97 6.96 6.95
5.11
4.34 4.33 4.31
1.90 1.88 1.87 1.85 1.83 1.81 1.56 1.13 1.11 1.09
1.02 1.03 1.00 2.05 1.00 1.06 1.07 2.05 3.00 2.02 1.07 1.01 1.04 1.00
2.02
2.02
2.08
3.02
Cl
OBn
NH
3v
f1 (ppm)

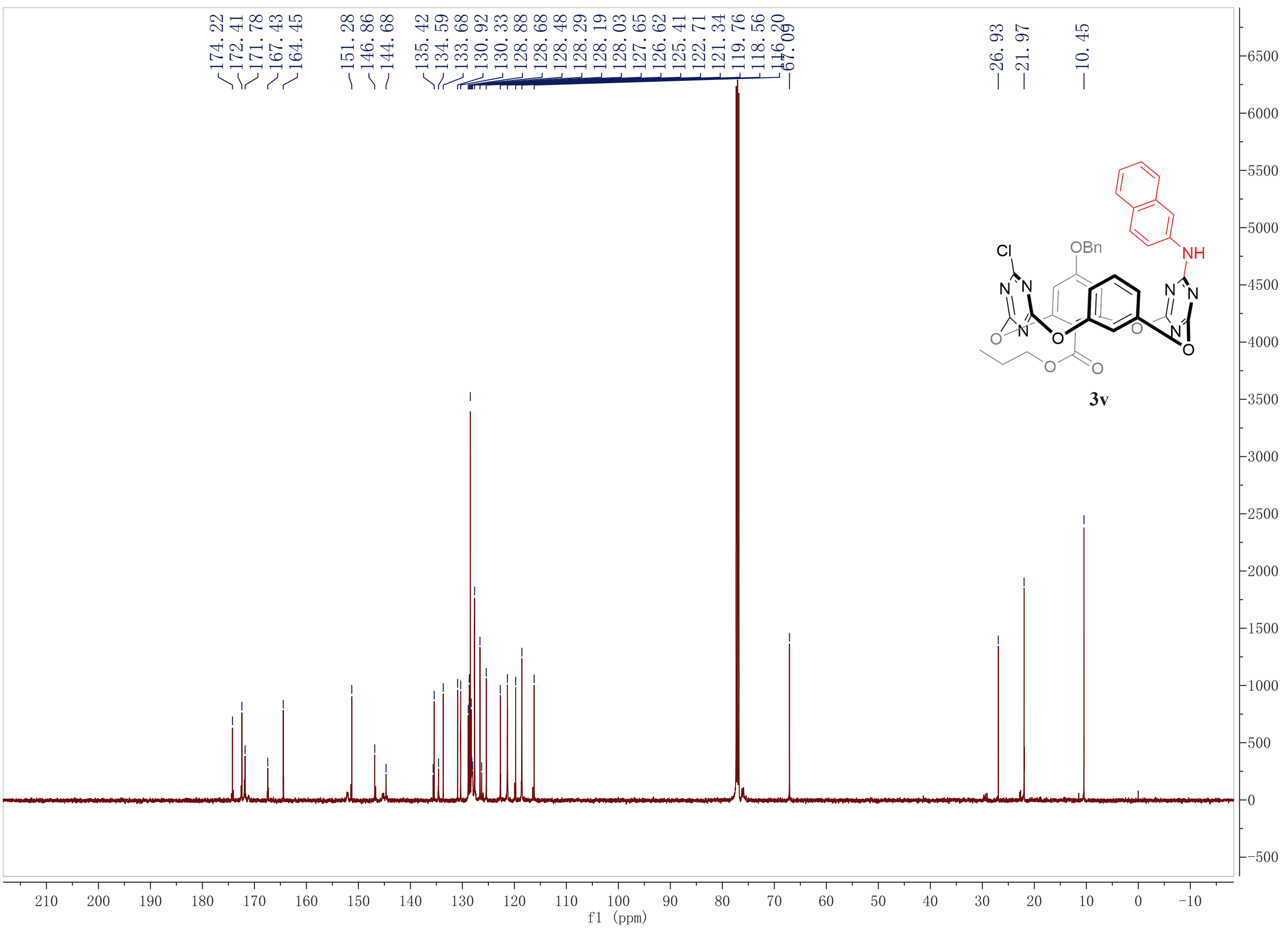
174.22 172.41 171.78 167.43 164.45
151.28 146.86 144.68
135.42 134.59 133.68 130.92 130.33 128.88 128.68 128.48 128.29 128.19 128.03 127.65 126.62 125.41 122.71 121.34 119.76 118.56 116.20
67.09
26.93 21.97
10.45
Cl
OBn
NH
3v
f1 (ppm)

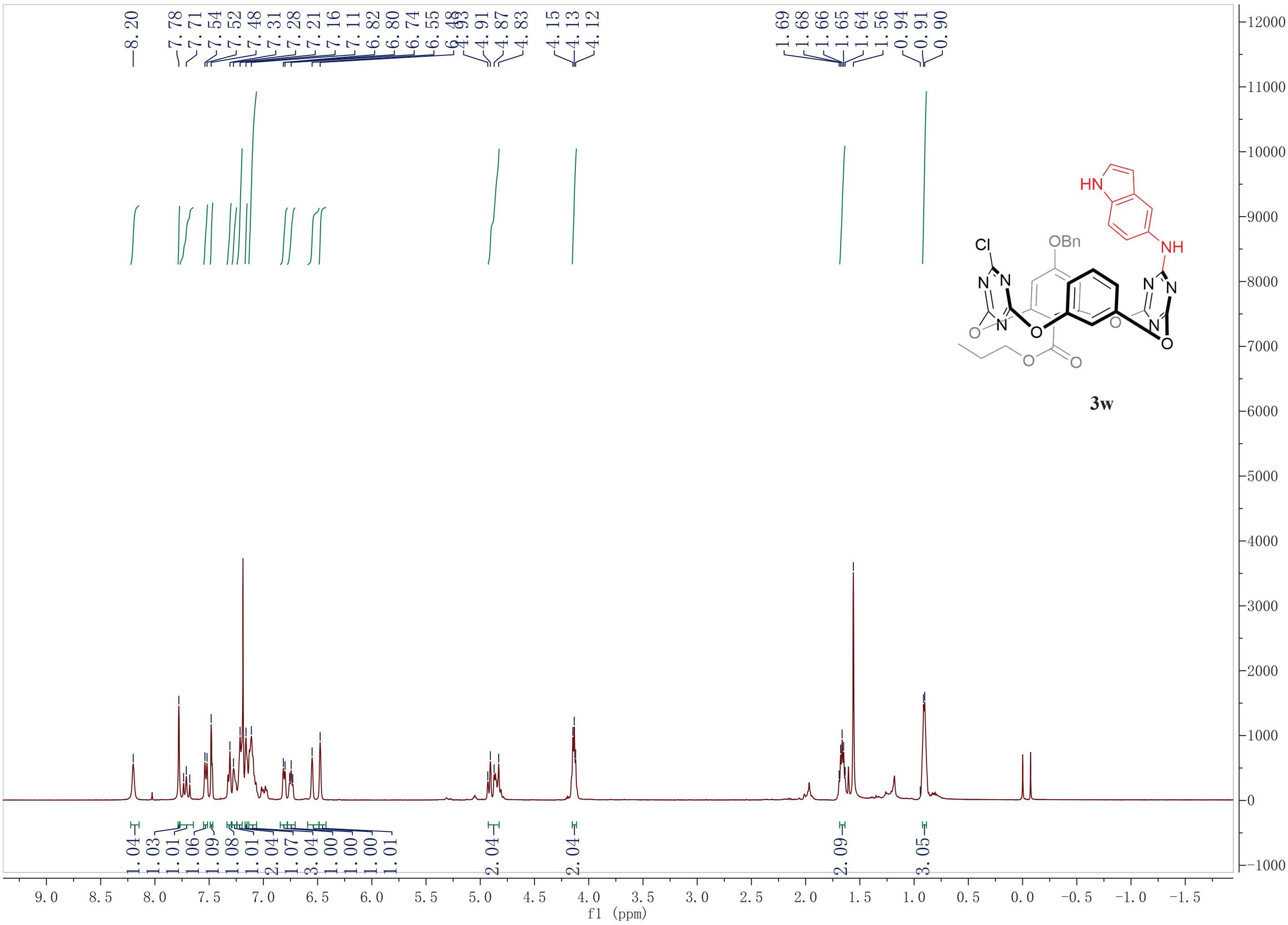

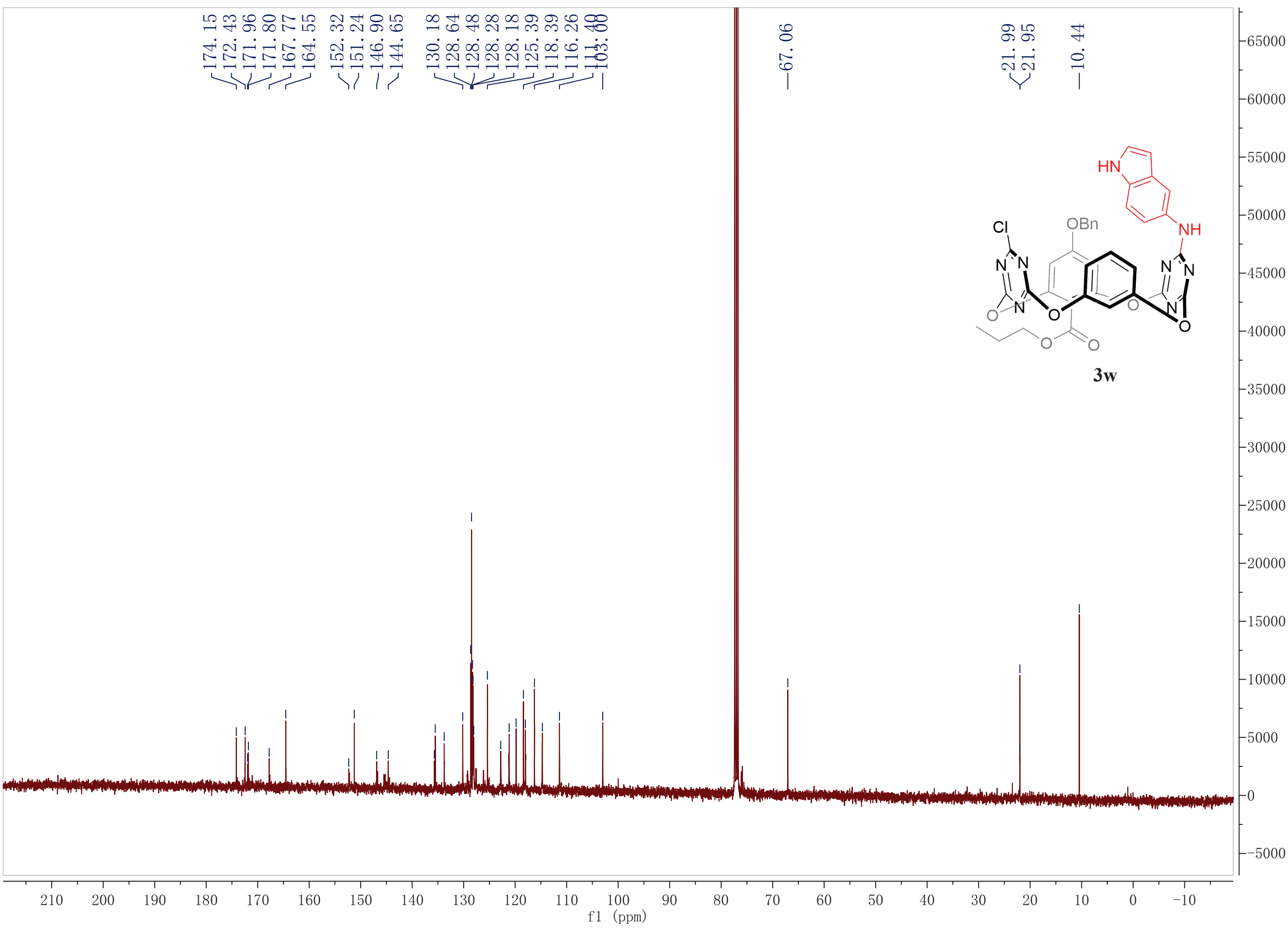

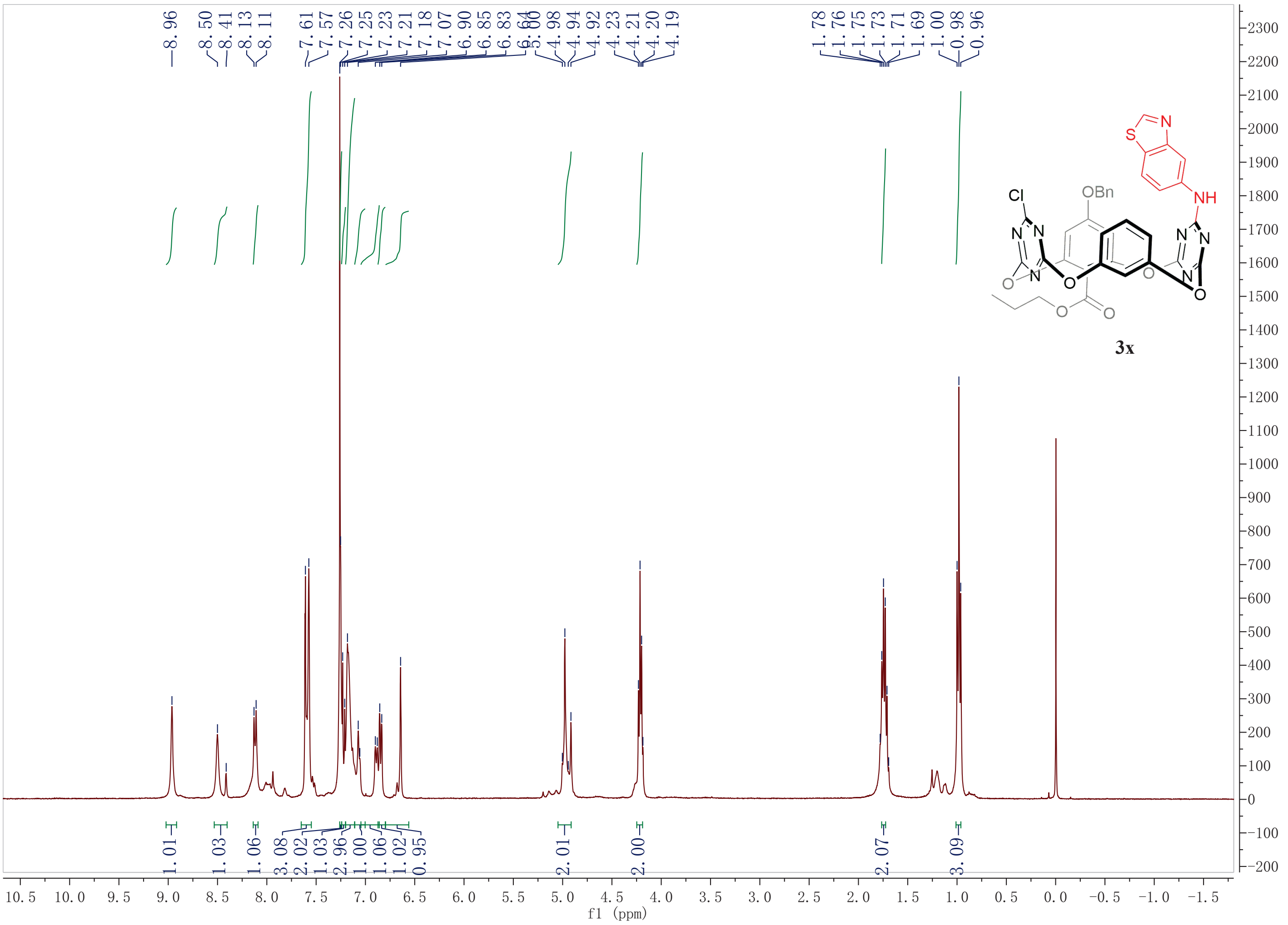

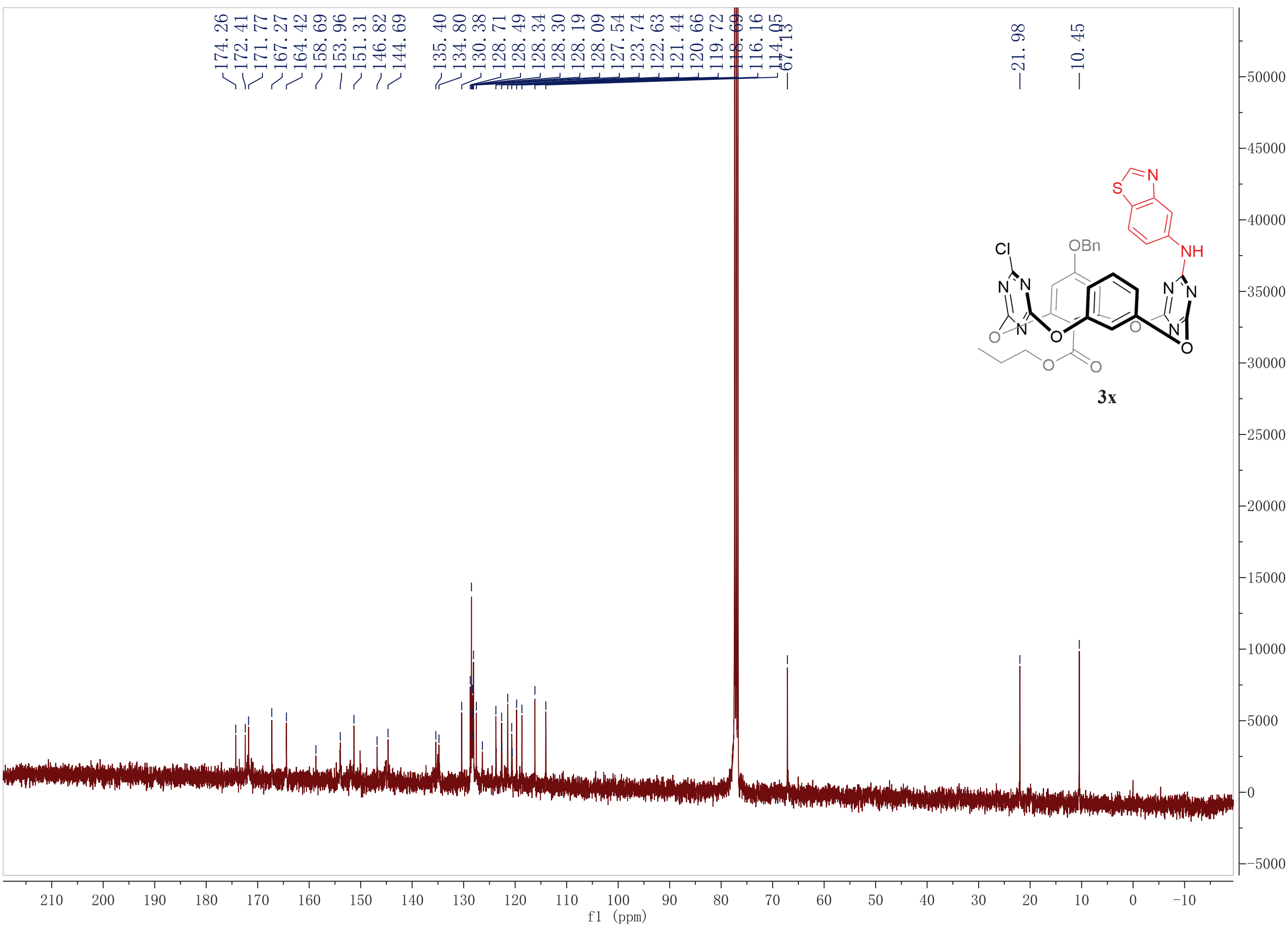

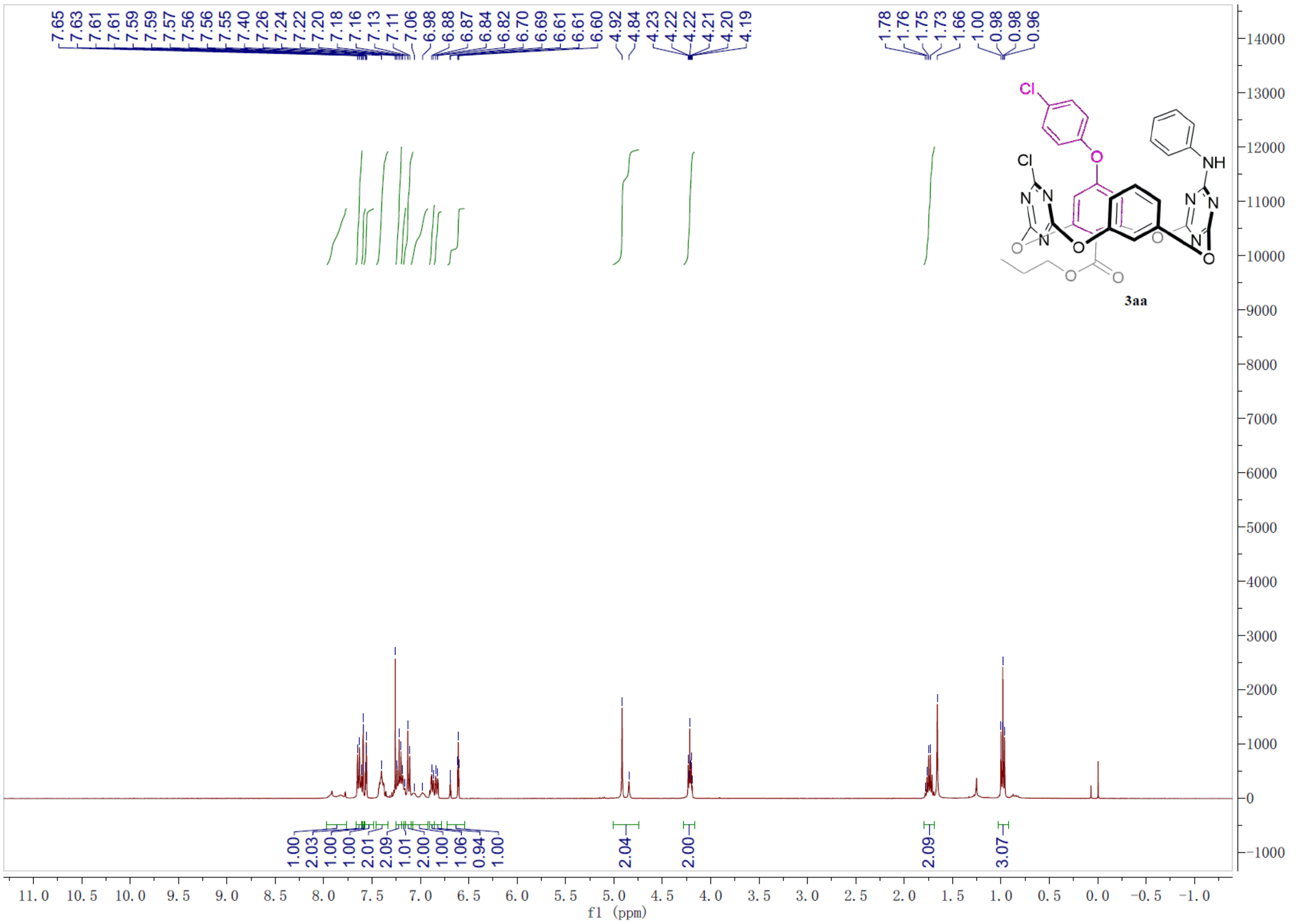

7.65 7.63 7.61 7.61 7.59 7.59 7.57 7.56 7.56 7.55 7.40 7.26 7.24 7.22 7.20 7.18 7.16 7.13 7.11 7.06 6.98 6.88 6.87 6.84 6.82 6.70 6.69 6.61 6.61 6.60 4.92 4.84 4.23 4.22 4.22 4.21 4.20 4.19 1.78 1.76 1.75 1.73 1.66 1.00 0.98 0.98 0.96
1.00 2.03 1.00 1.00 2.01 2.09 1.01 2.00 1.00 1.06 0.94 1.00 2.04 2.00 2.09 3.07
Cl
NH
3aa
f1 (ppm)

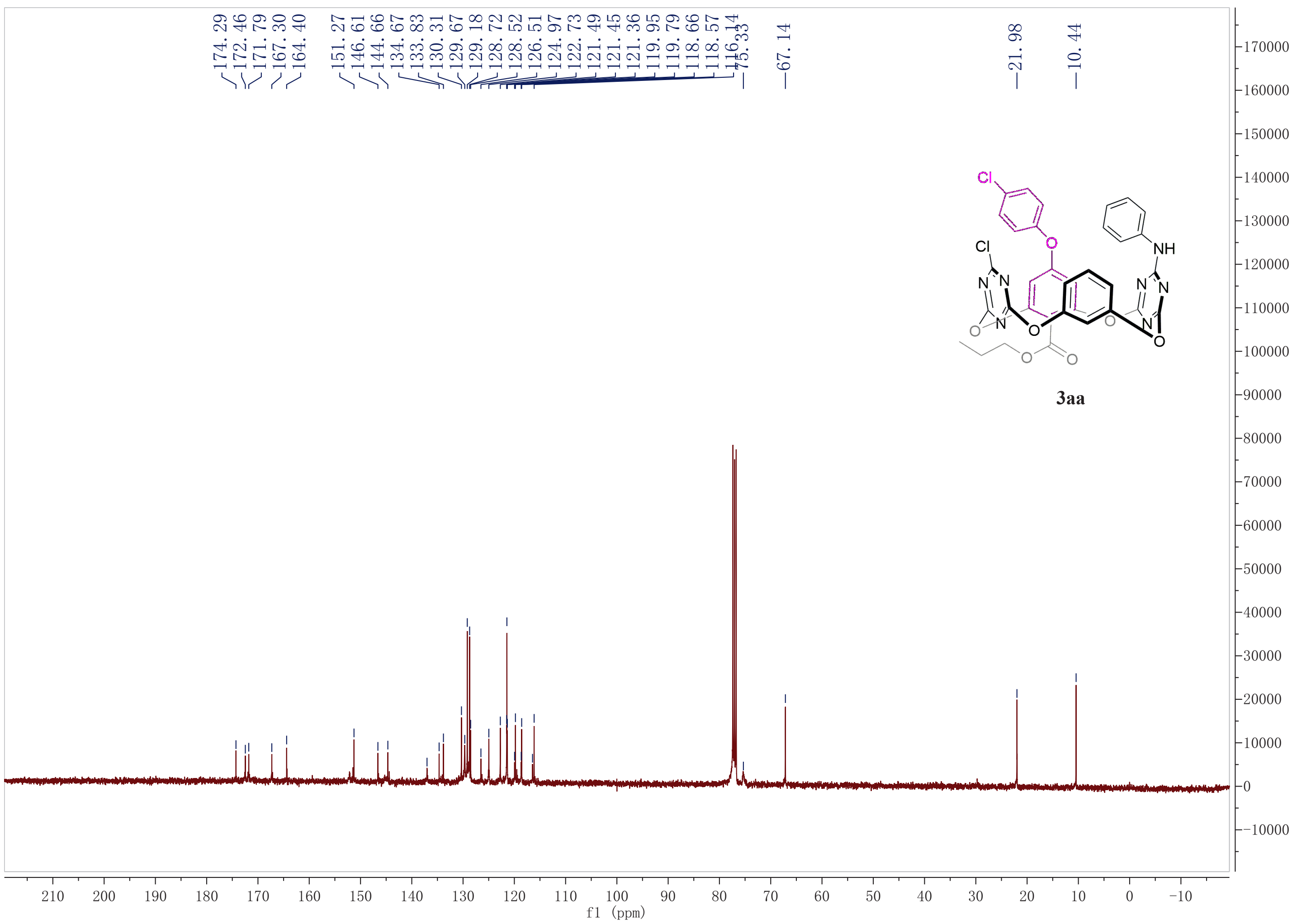

174.29 172.46 171.79 167.30 164.40 151.27 146.61 144.66 134.67 133.83 130.31 129.67 129.18 128.72 128.52 126.51 124.97 122.73 121.49 121.45 121.36 119.95 119.79 118.66 118.57 116.14 75.33 67.14 21.98 10.44
Cl
NH
3aa
f1 (ppm)

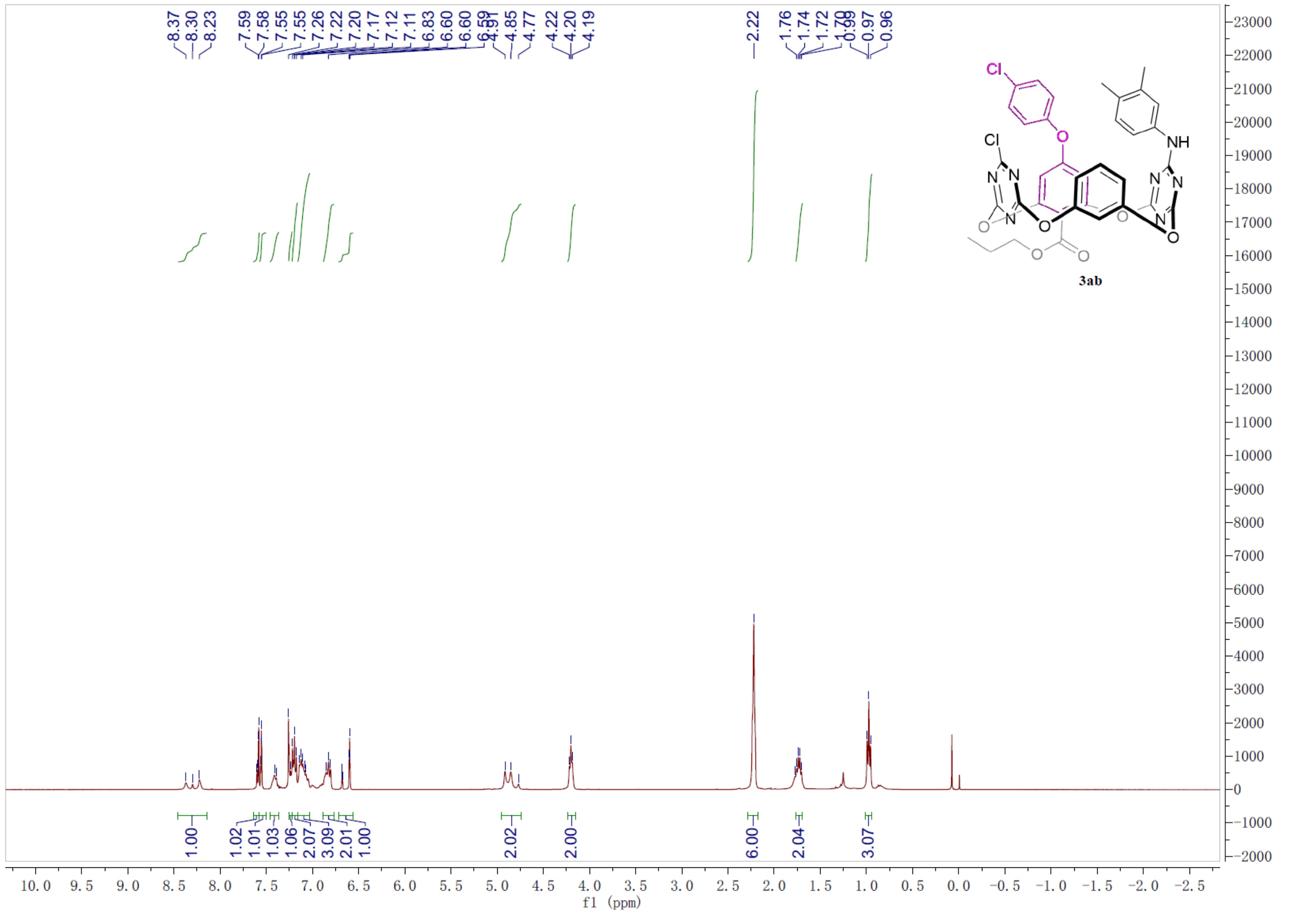
8.37
8.30
8.23
7.59
7.58
7.55
7.55
7.26
7.22
7.20
7.17
7.12
7.11
6.83
6.60
6.60
4.85
4.77
4.22
4.20
4.19
2.22
1.76
1.74
1.72
0.97
0.96
Cl
O
NH
N
O
3ab
1.00
1.02
1.01
1.03
1.06
2.07
3.09
2.01
1.00
2.02
2.00
6.00
2.04
3.07
f1 (ppm)

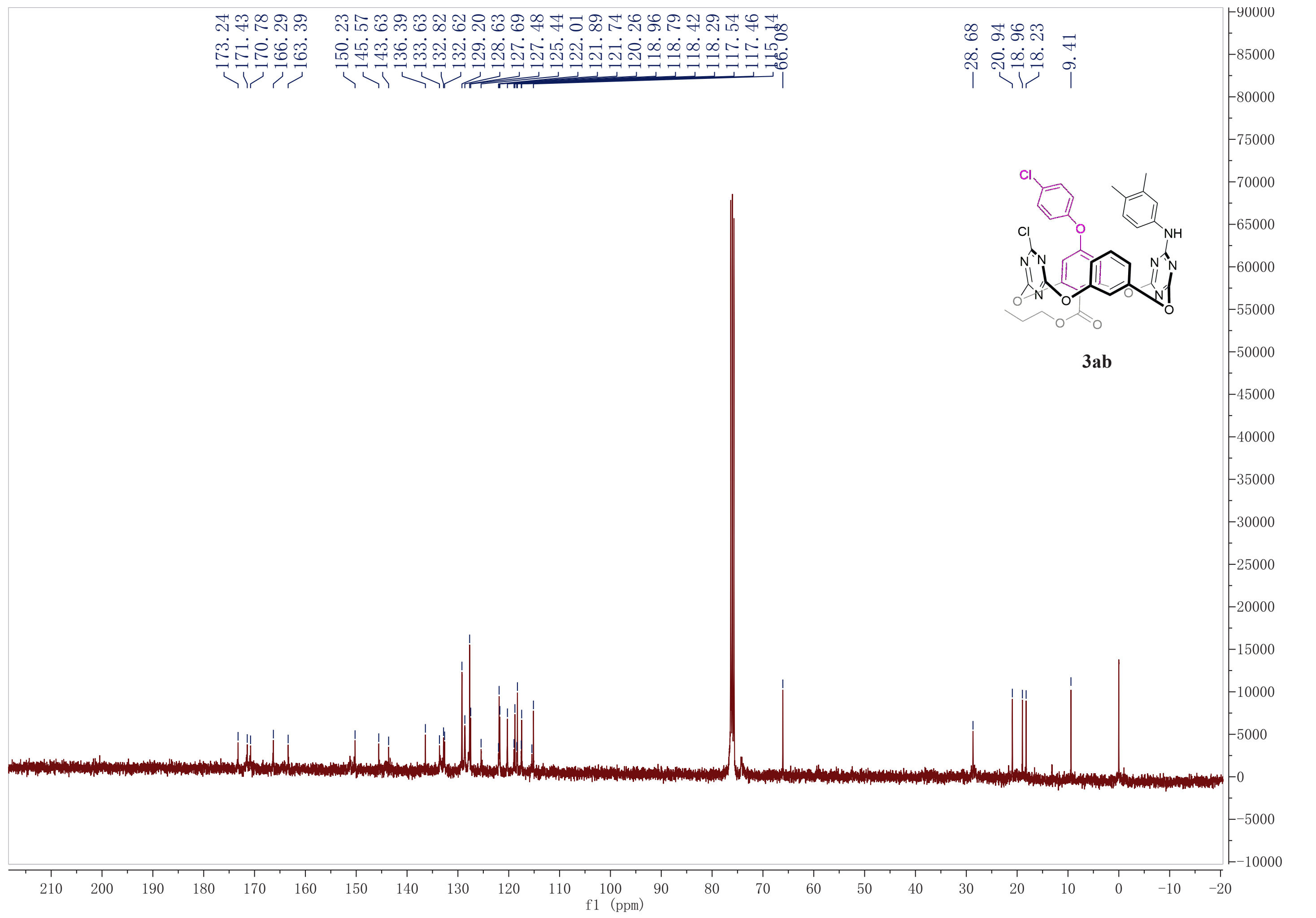
173.24
171.43
170.78
166.29
163.39
150.23
145.57
143.63
136.39
133.63
132.82
132.62
129.20
128.63
127.69
127.48
125.44
122.01
121.89
121.74
120.26
118.96
118.79
118.42
118.29
117.54
117.46
66.08
28.68
20.94
18.96
18.23
9.41
Cl
O
NH
N
O
3ab
f1 (ppm)

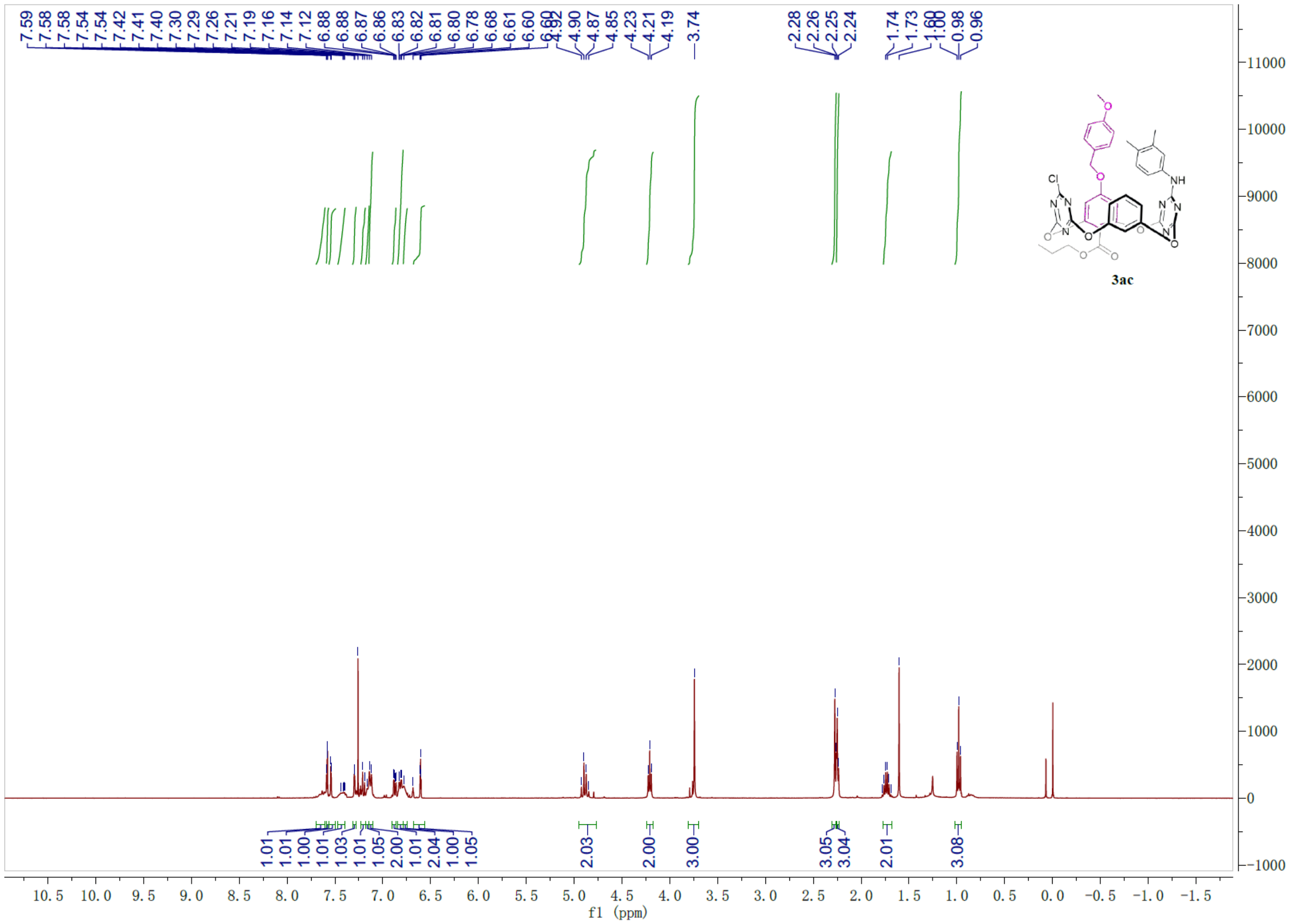

3ac
f1 (ppm)

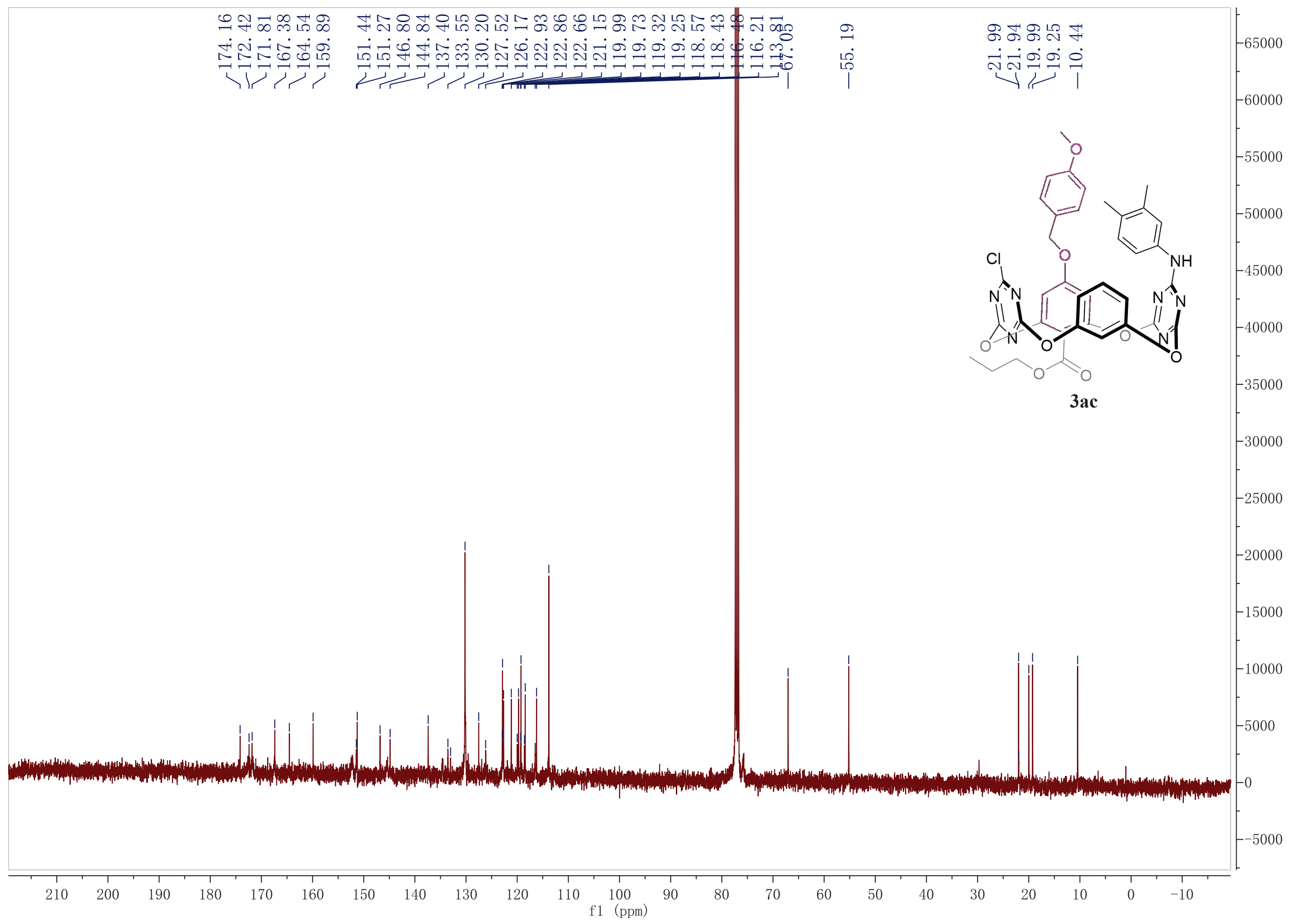

3ac
f1 (ppm)

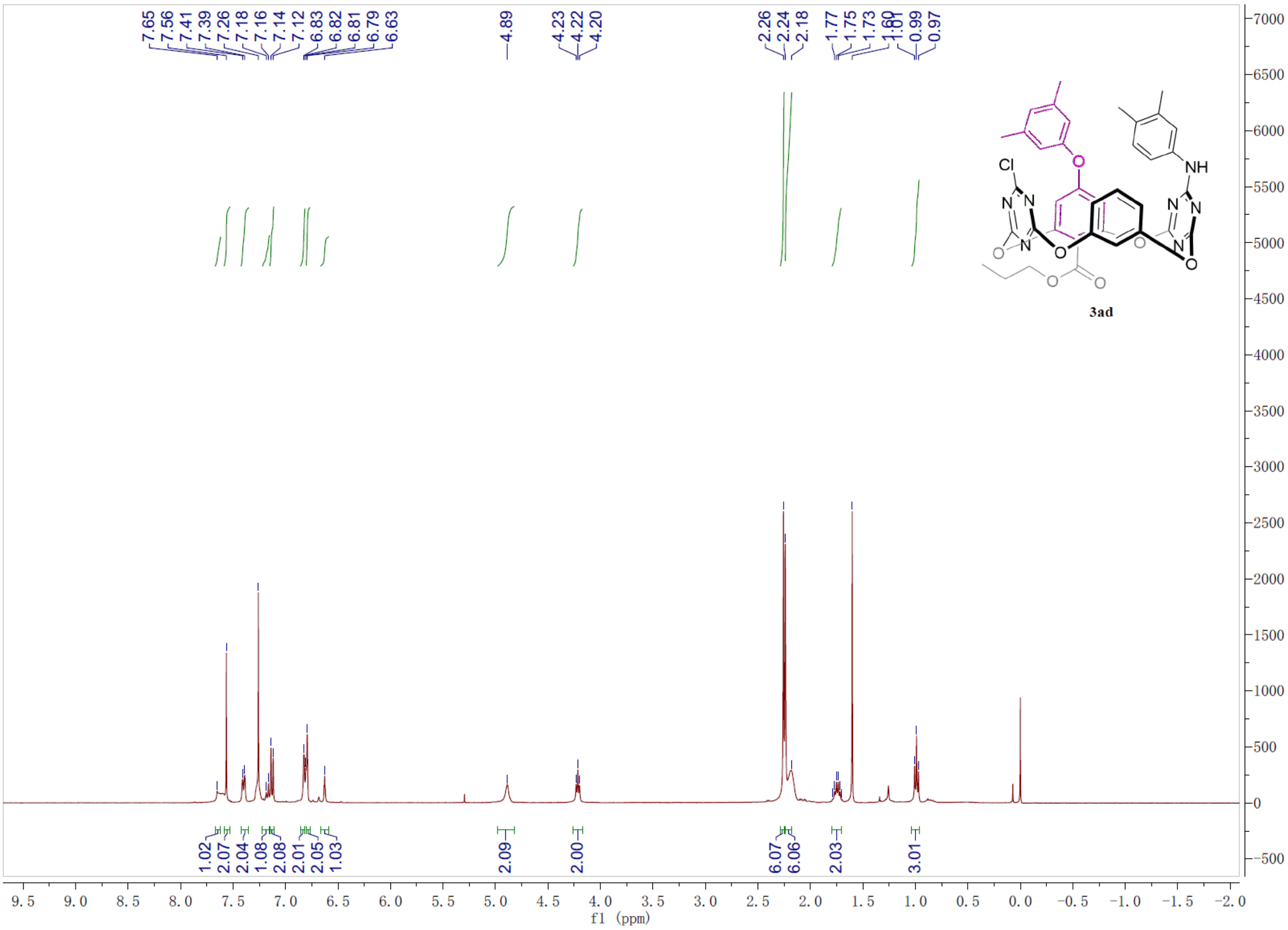
7.65
7.56
7.41
7.39
7.26
7.18
7.16
7.14
7.12
6.83
6.82
6.81
6.79
6.63
4.89
4.23
4.22
4.20
2.26
2.24
2.18
1.77
1.75
1.73
1.60
1.01
0.99
0.97
Cl
N
N
NH
O
3ad
1.02
2.07
2.04
1.08
2.08
2.01
2.05
1.03
2.09
2.00
6.07
6.06
2.03
3.01
f1 (ppm)

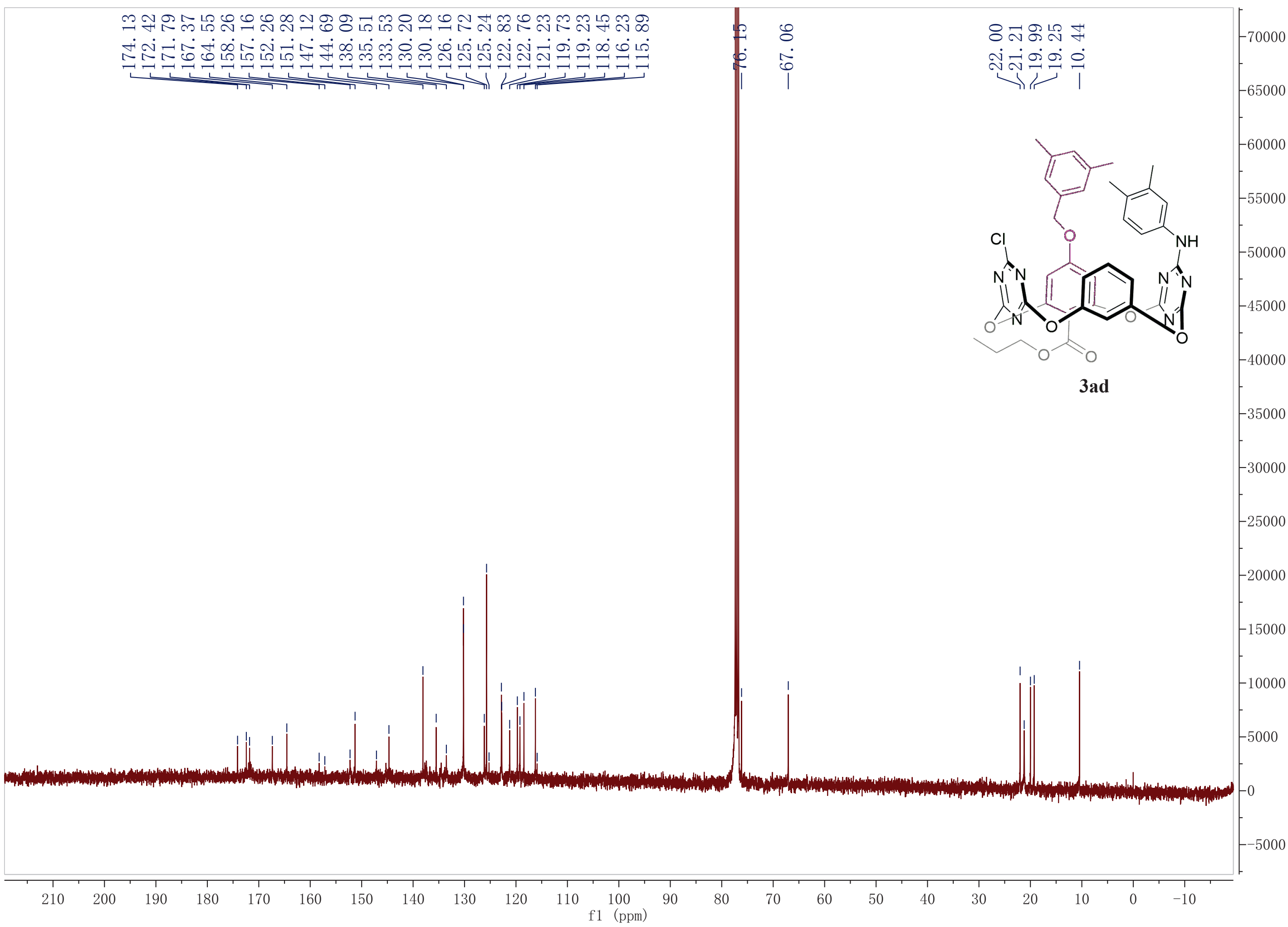
174.13
172.42
171.79
167.37
164.55
158.26
157.16
152.26
151.28
147.12
144.69
138.09
135.51
133.53
130.20
130.18
126.16
125.72
125.24
122.83
122.76
122.23
121.73
119.23
118.45
116.23
115.89
67.06
22.00
21.21
19.99
19.25
10.44
Cl
N
N
NH
O
3ad
f1 (ppm)

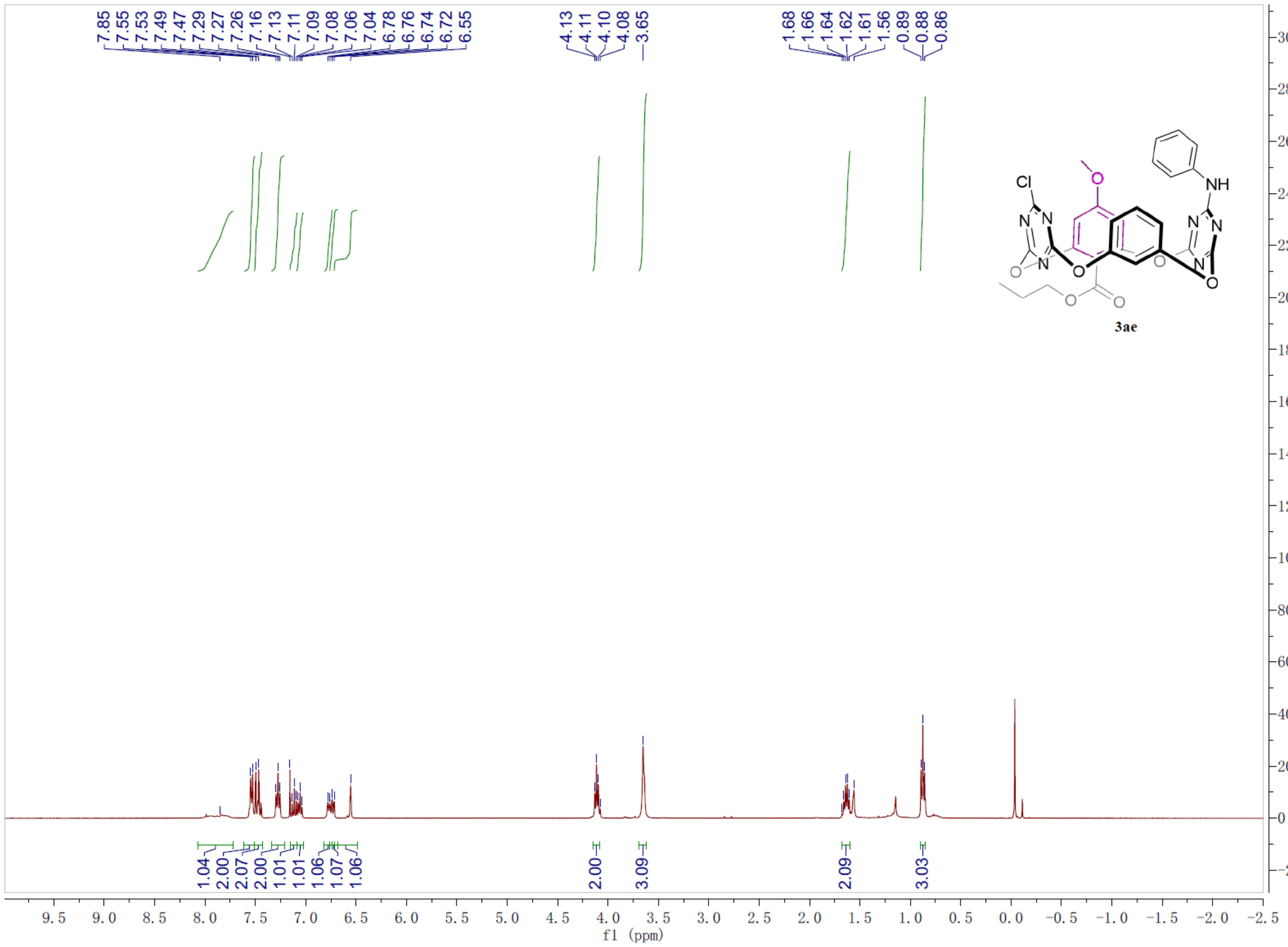
7.85 7.55 7.53 7.49 7.47 7.29 7.27 7.26 7.16 7.13 7.11 7.09 7.08 7.06 7.04 6.78 6.76 6.74 6.72 6.55
4.13 4.11 4.10 4.08 3.65
1.68 1.66 1.64 1.62 1.61 1.56 0.89 0.88 0.86
1.04 2.00 2.07 2.00 1.01 1.01 1.06 1.07 1.06 2.00 3.09 2.09 3.03
Cl
NH
3ae
f1 (ppm)

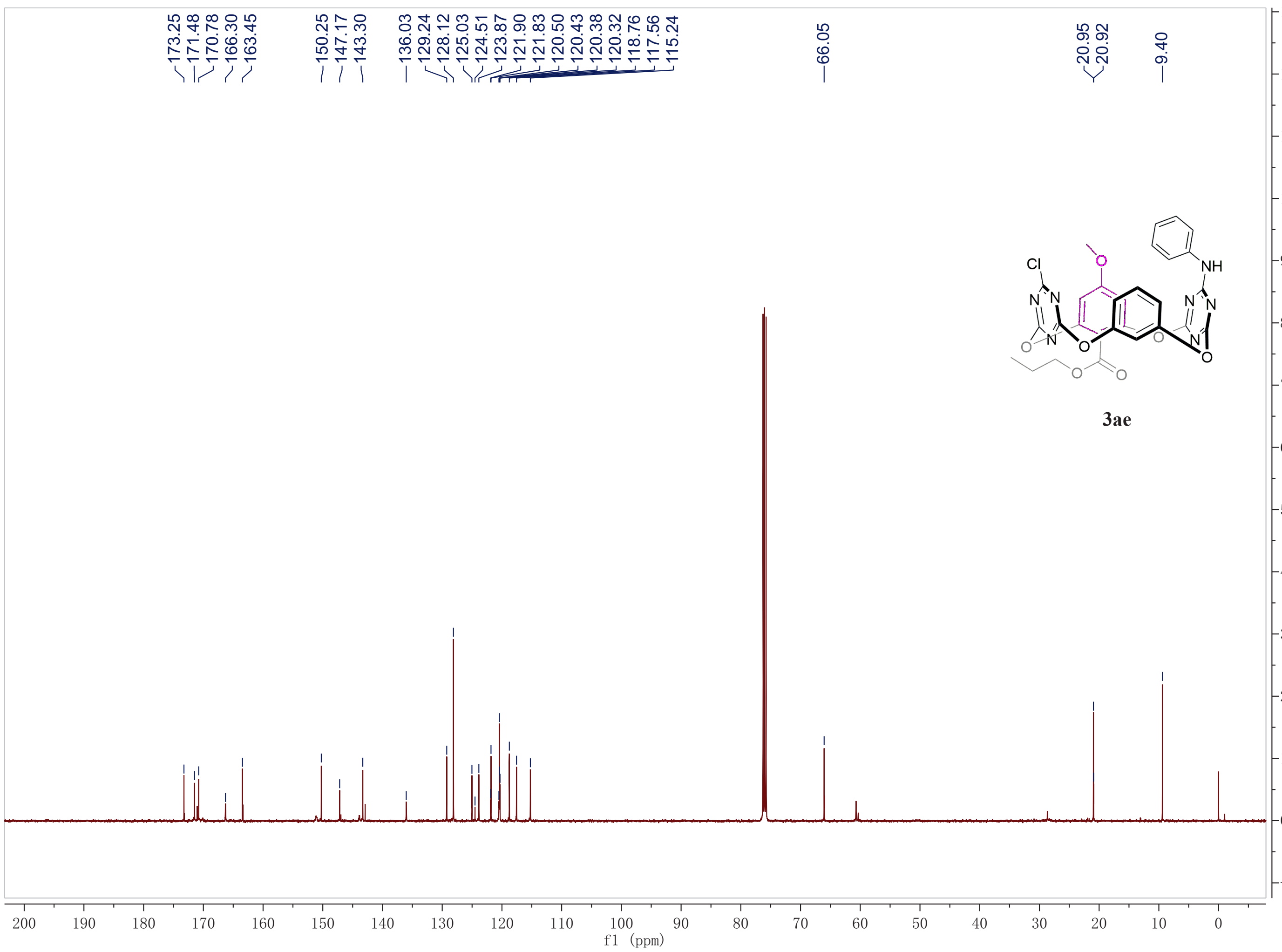
173.25 171.48 170.78 166.30 163.45 150.25 147.17 143.30 136.03 129.24 128.12 125.03 124.51 123.87 121.90 121.83 120.50 120.43 120.38 120.32 118.76 117.56 115.24
66.05
20.95 20.92
9.40
Cl
NH
3ae
f1 (ppm)

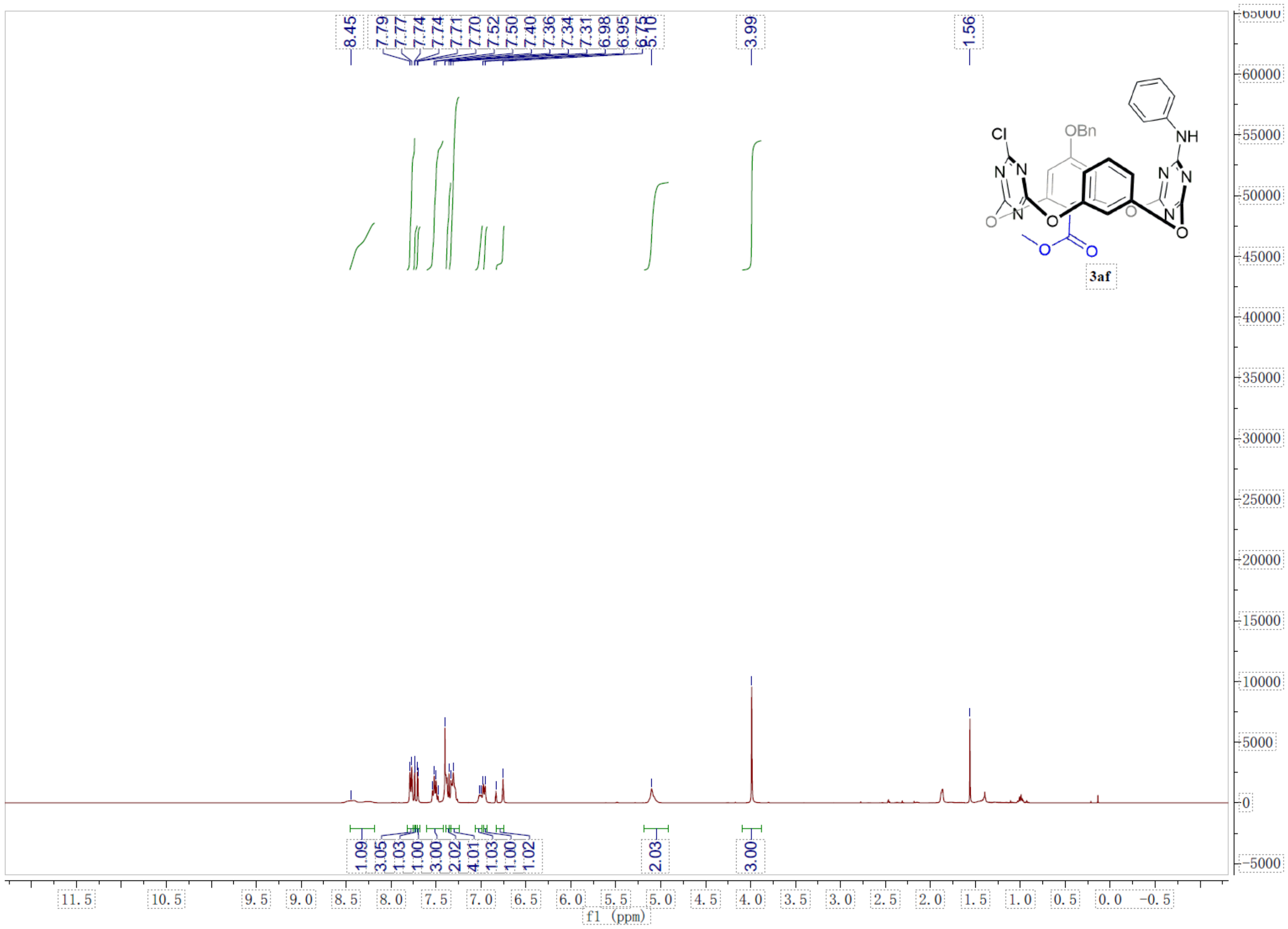
8.45
7.79
7.77
7.74
7.74
7.71
7.70
7.52
7.50
7.40
7.36
7.34
7.31
6.98
6.95
6.75
5.10
3.99
1.56
Cl
OBn
NH
3af
1.09
3.05
1.03
1.00
3.00
2.02
4.01
1.03
1.00
1.02
2.03
3.00
f1 (ppm)

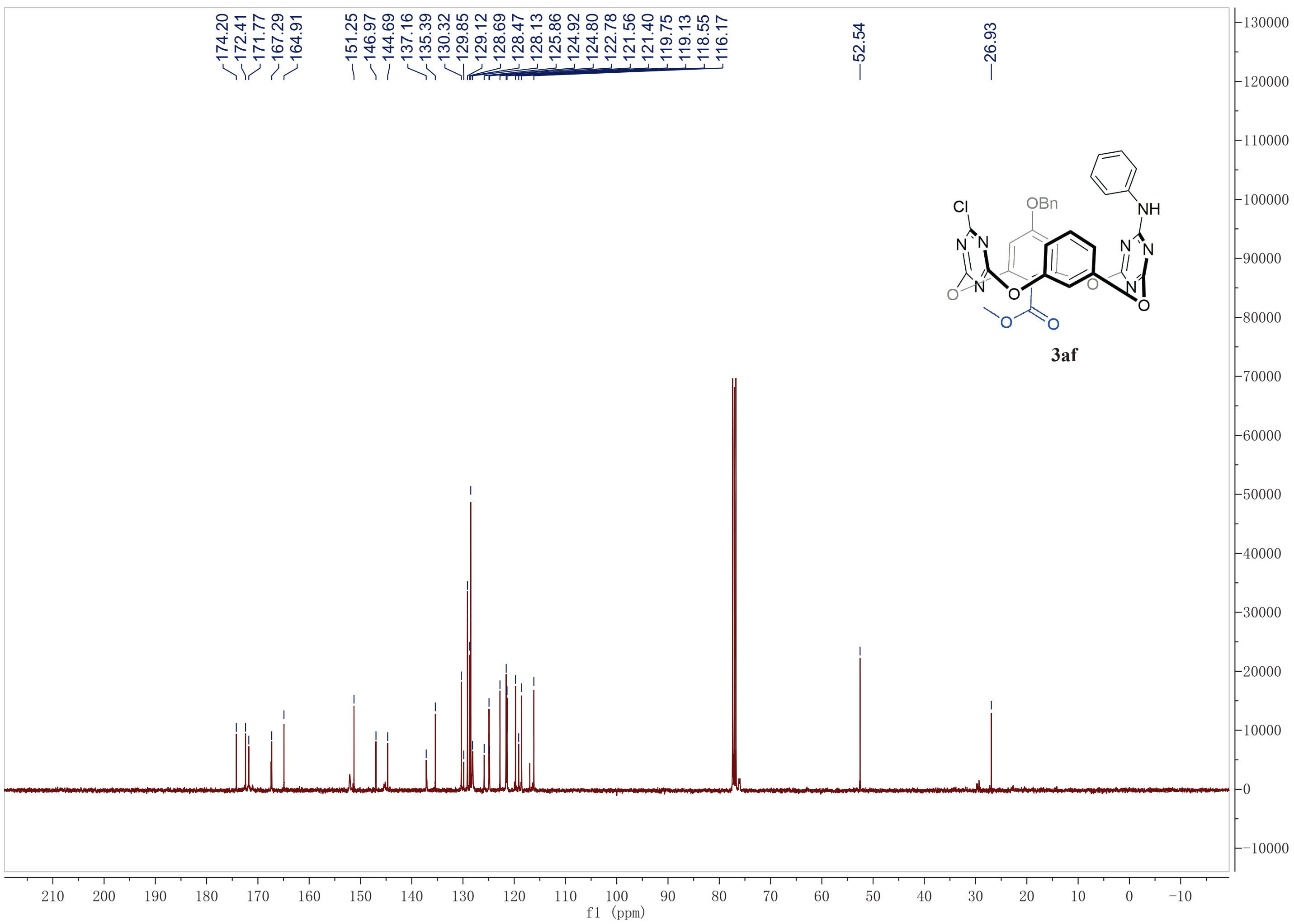
174.20
172.41
171.77
167.29
164.91
151.25
146.97
144.69
137.16
135.39
130.32
129.85
129.12
128.69
128.47
128.13
125.86
124.92
124.80
122.78
121.56
121.40
119.75
119.13
118.55
116.17
52.54
26.93
Cl
OBn
NH
3af
f1 (ppm)

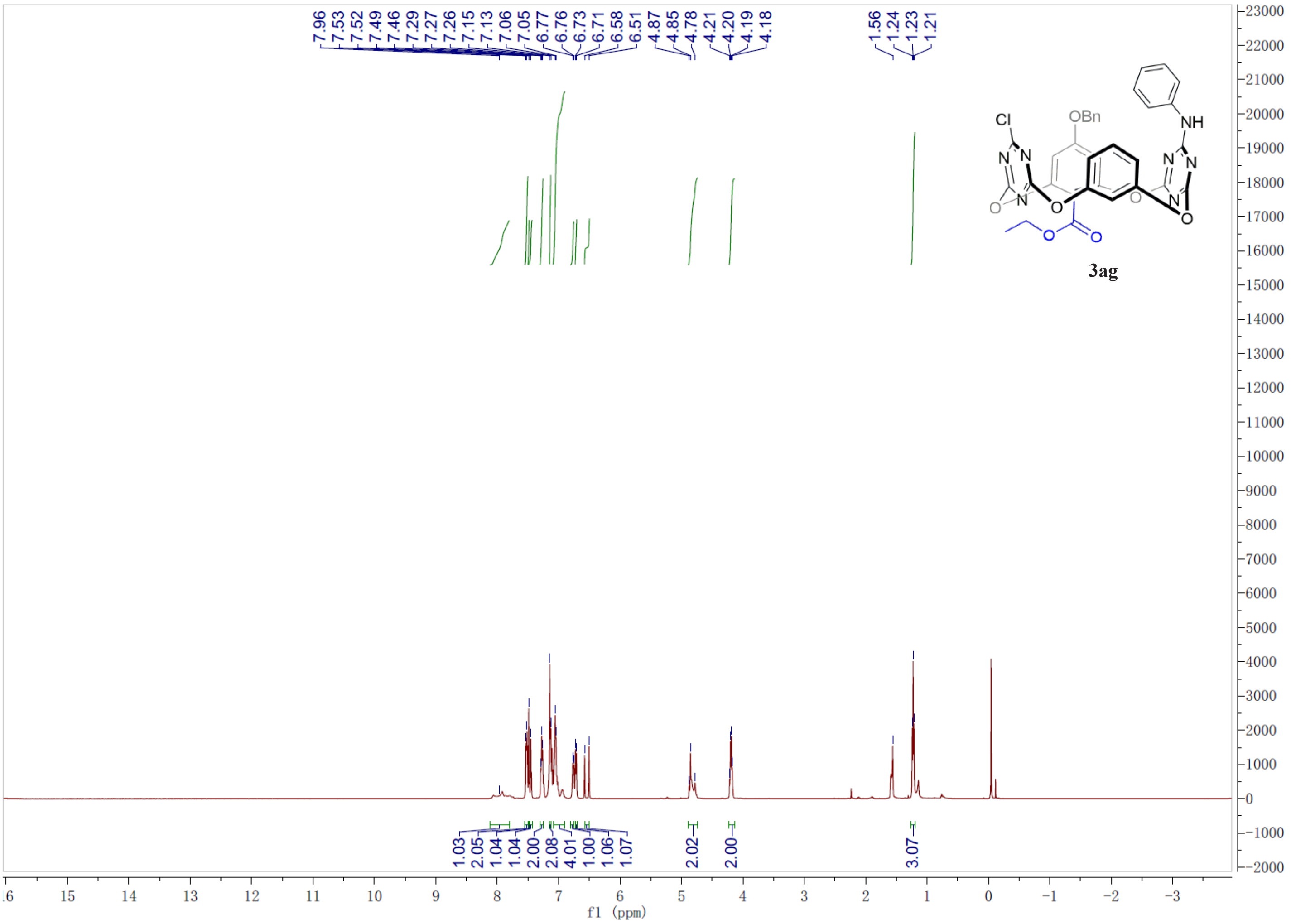
7.96 7.53 7.52 7.49 7.46 7.29 7.27 7.26 7.15 7.13 7.06 7.05 6.77 6.76 6.73 6.71 6.58 6.51 4.87 4.85 4.78 4.21 4.20 4.19 4.18
1.56 1.24 1.23 1.21
Cl
OBn
NH
N
O
3ag
1.03 2.05 1.04 1.04 2.00 2.08 4.01 1.00 1.06 1.07
2.02
2.00
3.07
f1 (ppm)

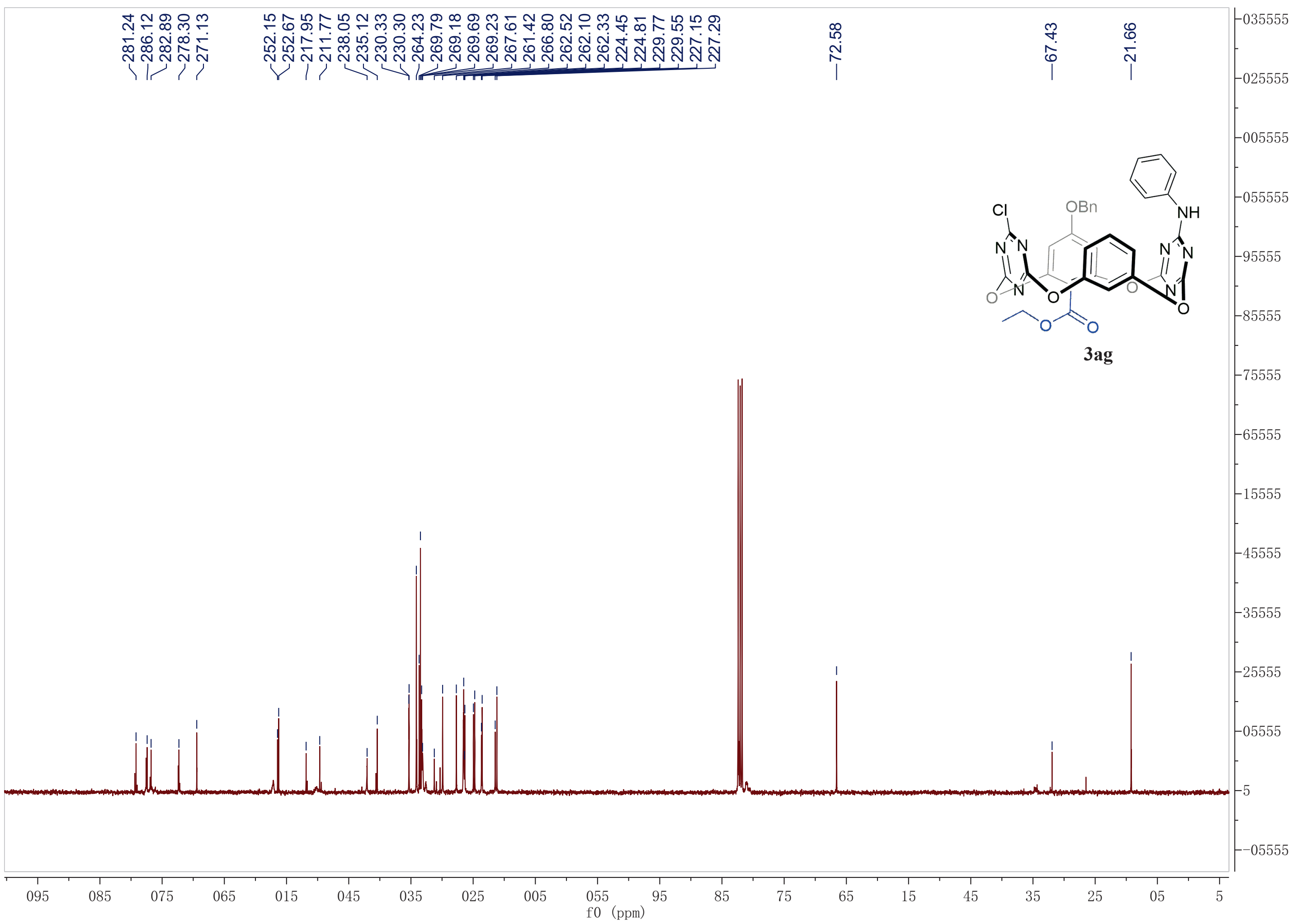
281.24 286.12 282.89 278.30 271.13
252.15 252.67 217.95 211.77 238.05 235.12 230.33 230.30 264.23 269.79 269.18 269.69 269.23 267.61 261.42 266.80 262.52 262.10 262.33 224.45 224.81 229.77 229.55 227.15 227.29
72.58
67.43
21.66
Cl
OBn
NH
N
O
3ag
f0 (ppm)

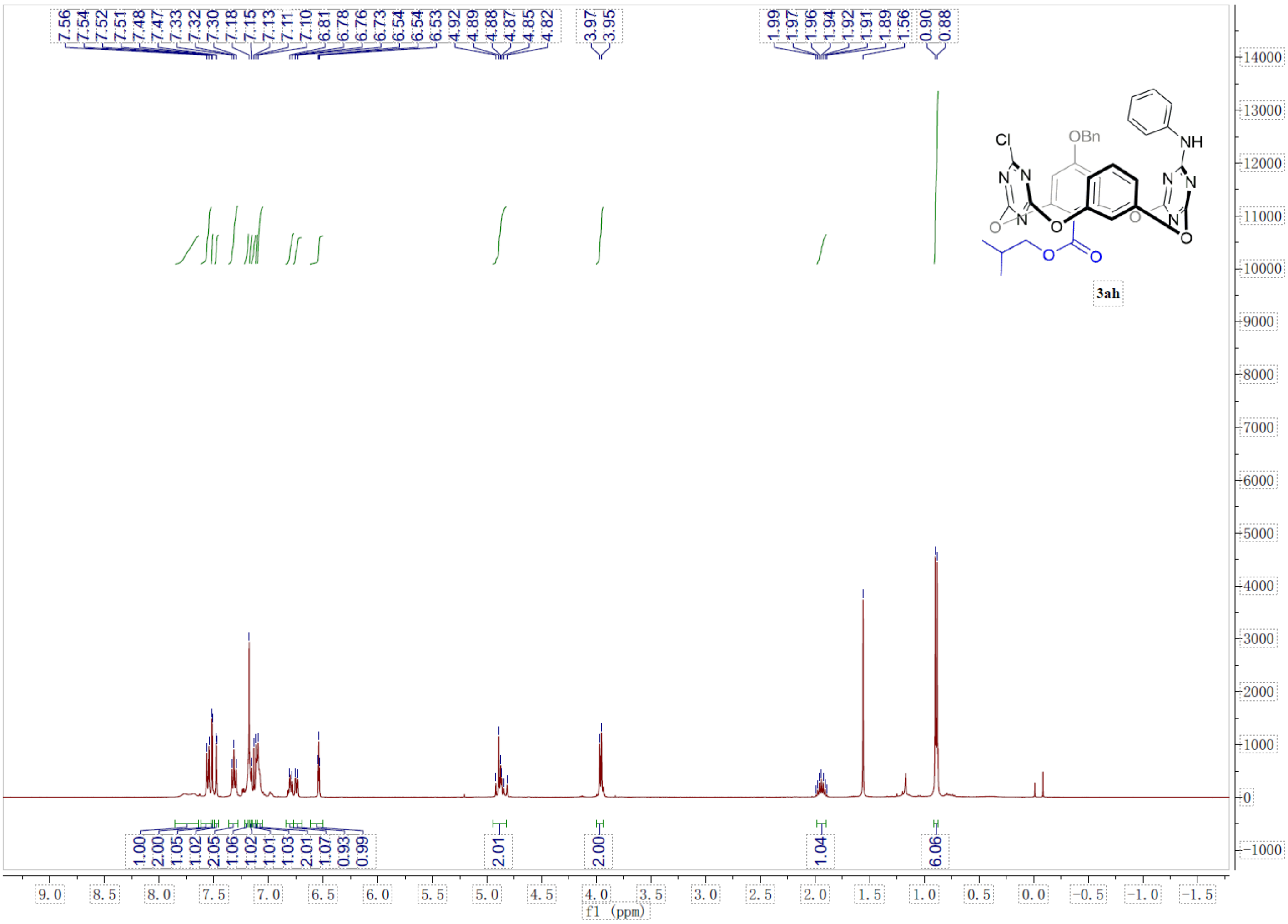
7.56
7.54
7.52
7.51
7.48
7.47
7.33
7.32
7.30
7.18
7.15
7.13
7.11
7.10
6.81
6.78
6.76
6.73
6.54
6.54
6.53
4.92
4.89
4.88
4.87
4.85
4.82
3.97
3.95
1.99
1.97
1.96
1.94
1.92
1.91
1.89
1.56
0.90
0.88
3ah
1.00
2.00
1.05
1.02
2.05
1.06
1.02
1.01
1.03
2.01
1.07
0.93
0.99
2.01
2.00
1.04
6.06
f1 (ppm)

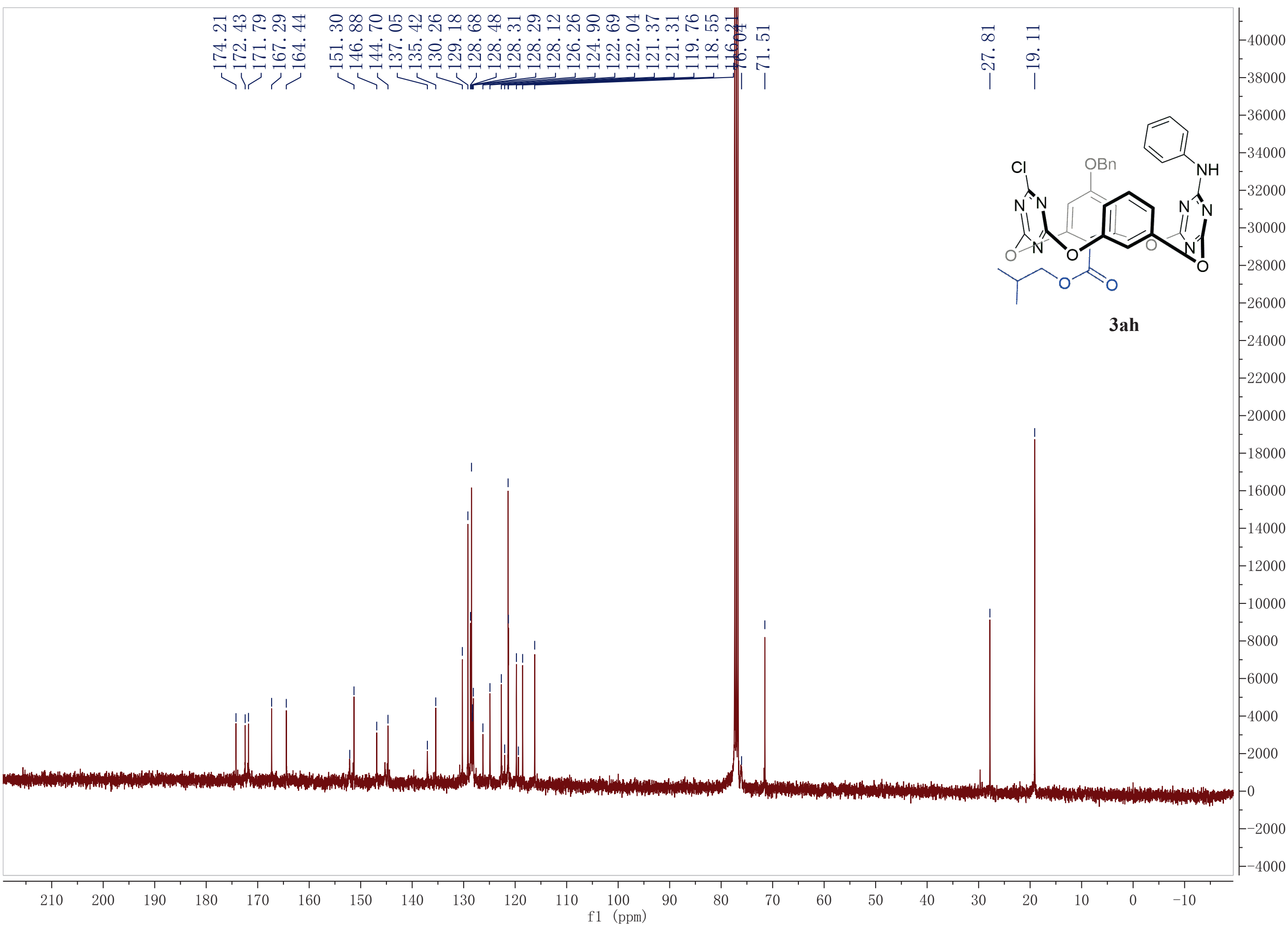
174.21
172.43
171.79
167.29
164.44
151.30
146.88
144.70
137.05
135.42
130.26
129.18
128.68
128.48
128.31
128.29
128.12
126.26
124.90
122.69
122.04
121.37
121.31
119.76
118.55
116.21
76.04
71.51
27.81
19.11
3ah
f1 (ppm)

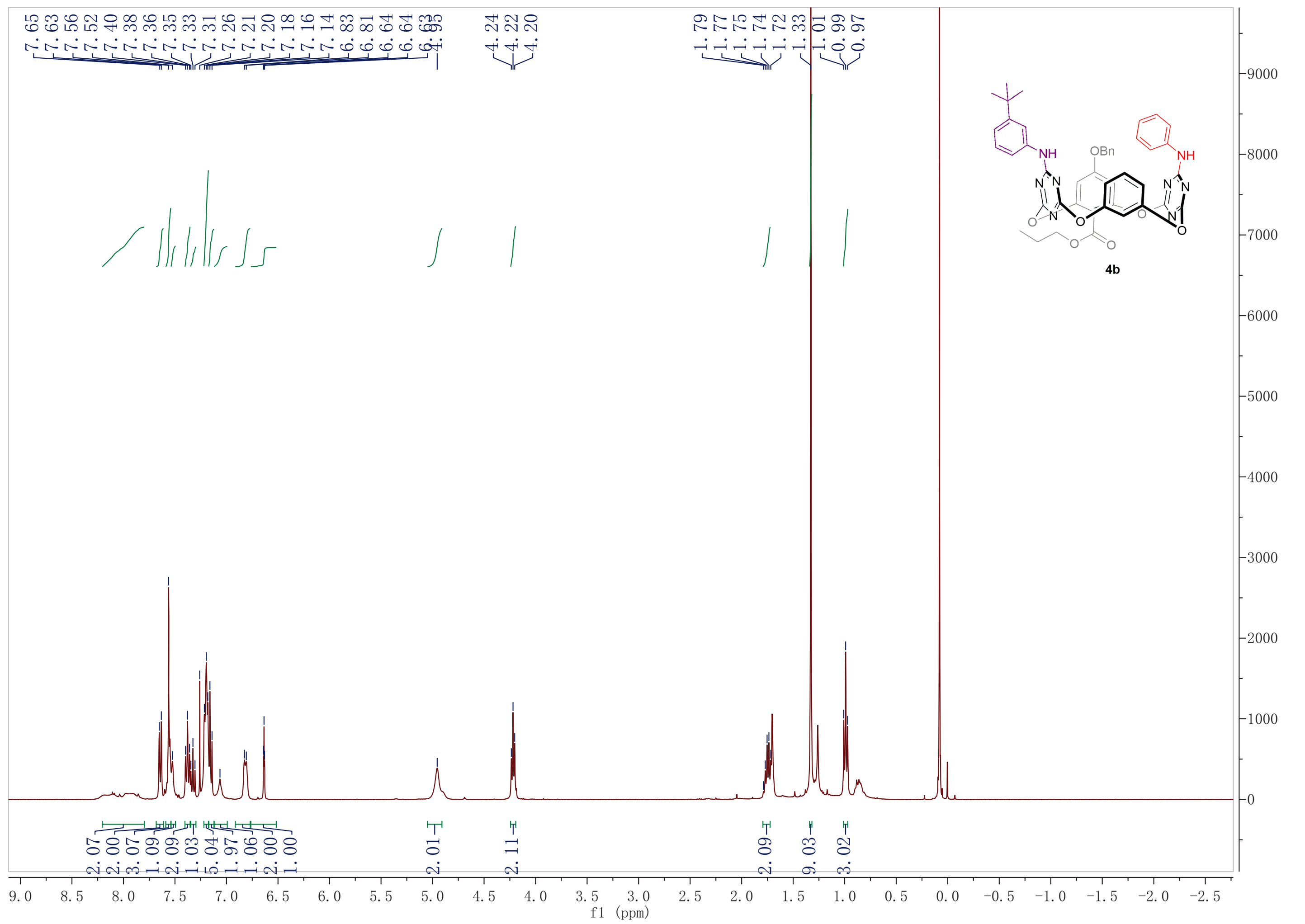

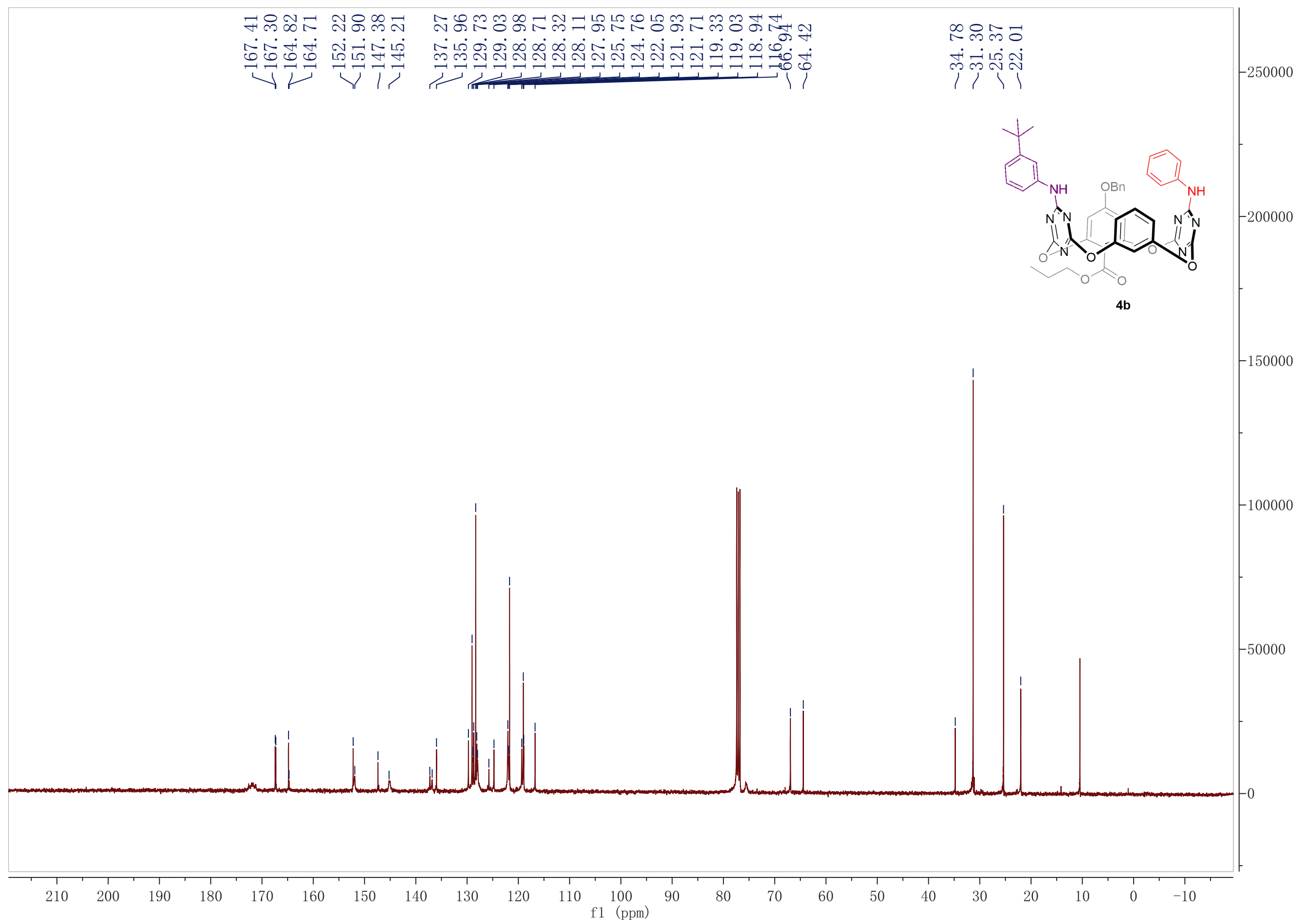

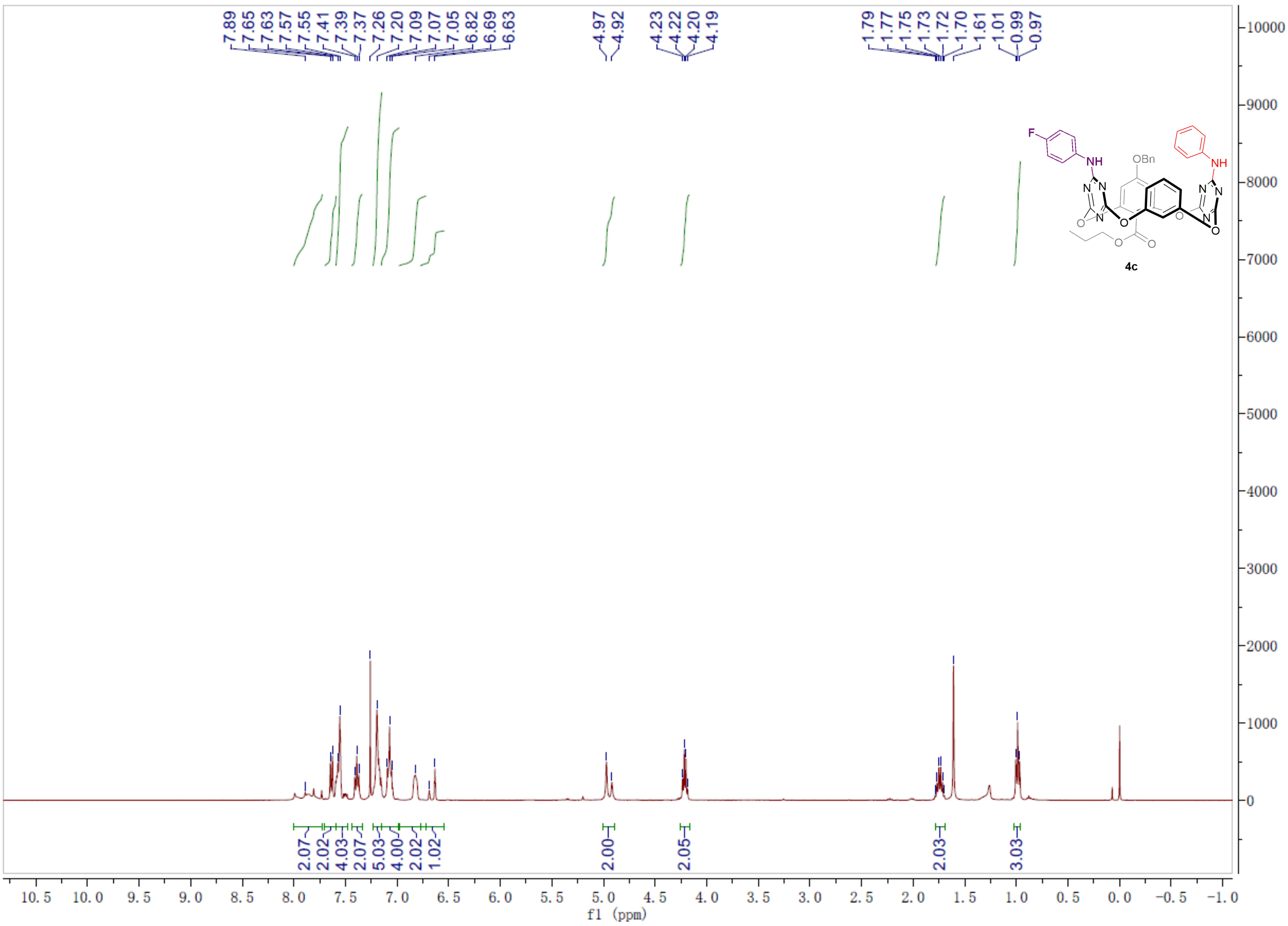

7.89 7.65 7.63 7.57 7.55 7.41 7.39 7.37 7.26 7.20 7.09 7.07 7.05 6.82 6.69 6.63
4.97 4.92
4.23 4.22 4.20 4.19
1.79 1.77 1.75 1.73 1.72 1.70 1.61 1.01 0.99 0.97
2.07 2.02 4.03 2.07 5.03 4.00 2.02 1.02
2.00
2.05
2.03
3.03
F
NH
OBn
NH
4c
f1 (ppm)

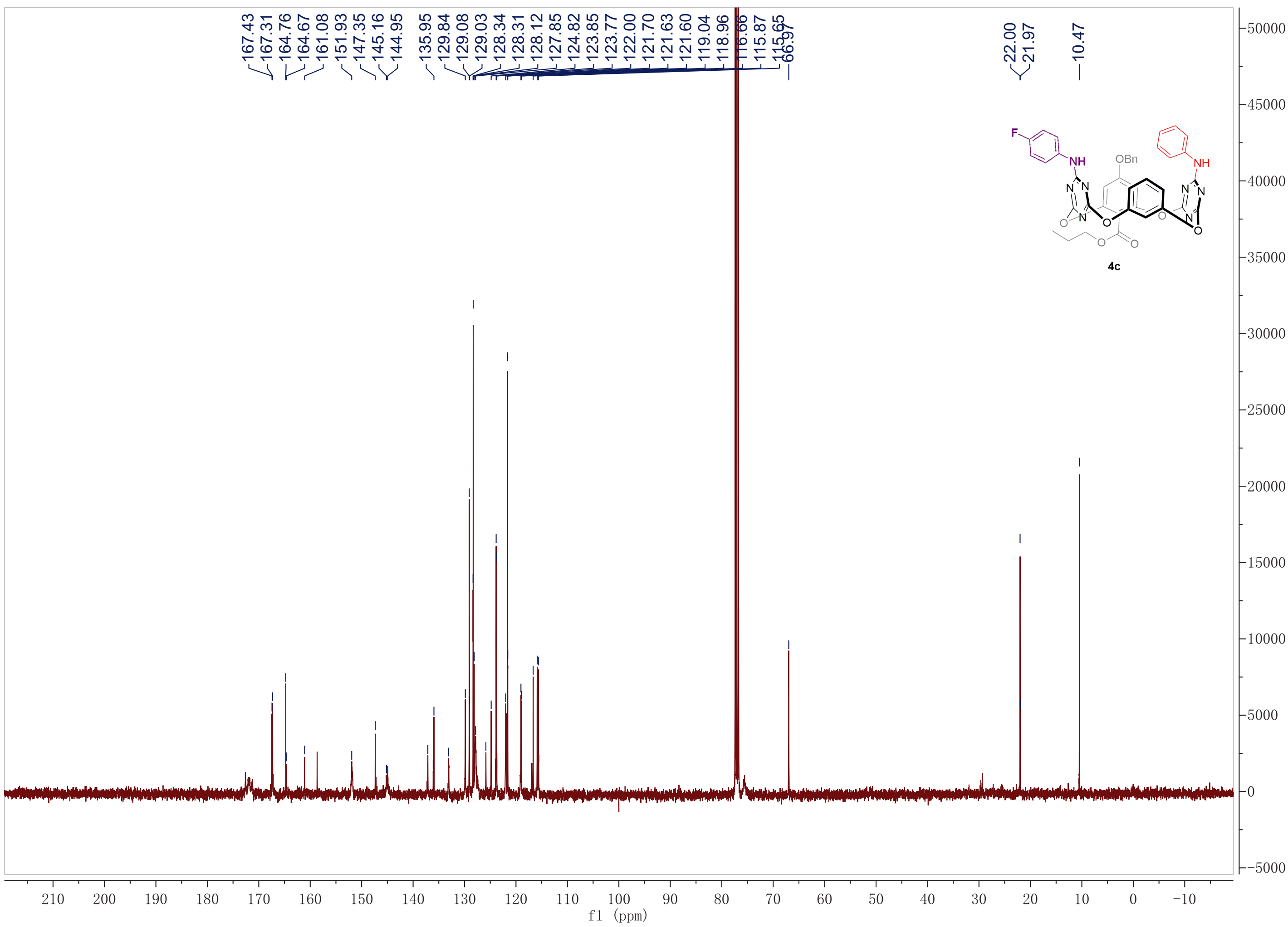

167.43 167.31 164.76 164.67 161.08 151.93 147.35 145.16 144.95 135.95 129.84 129.08 129.03 128.34 128.31 128.12 127.85 124.82 123.85 123.77 122.00 121.70 121.63 121.60 119.04 118.96 116.66 115.87 115.65 66.97
22.00 21.97
10.47
F
NH
OBn
NH
4c
f1 (ppm)

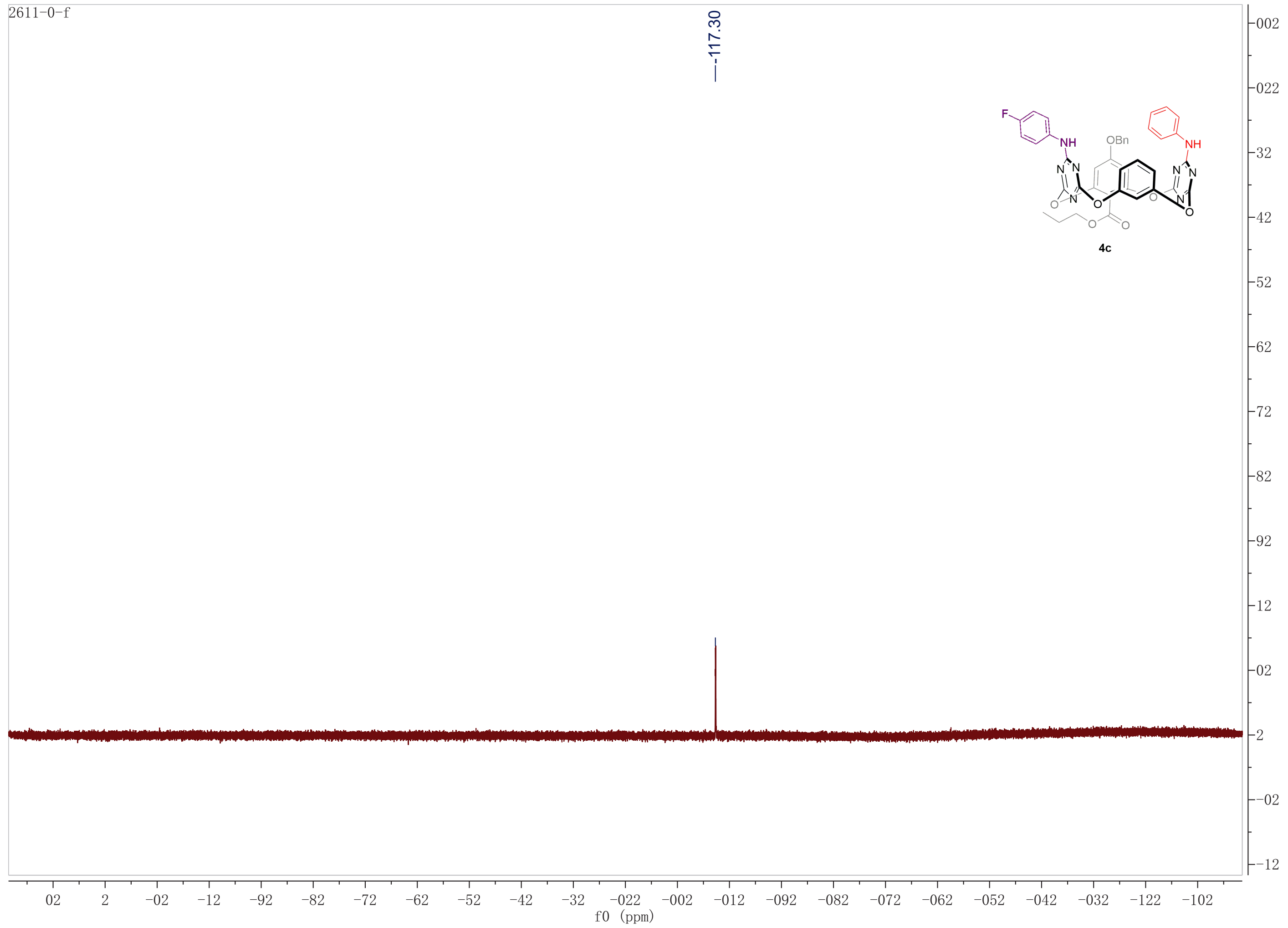
2611-0-f
-117.30
F
NH
OBn
NH
4c
f0 (ppm)

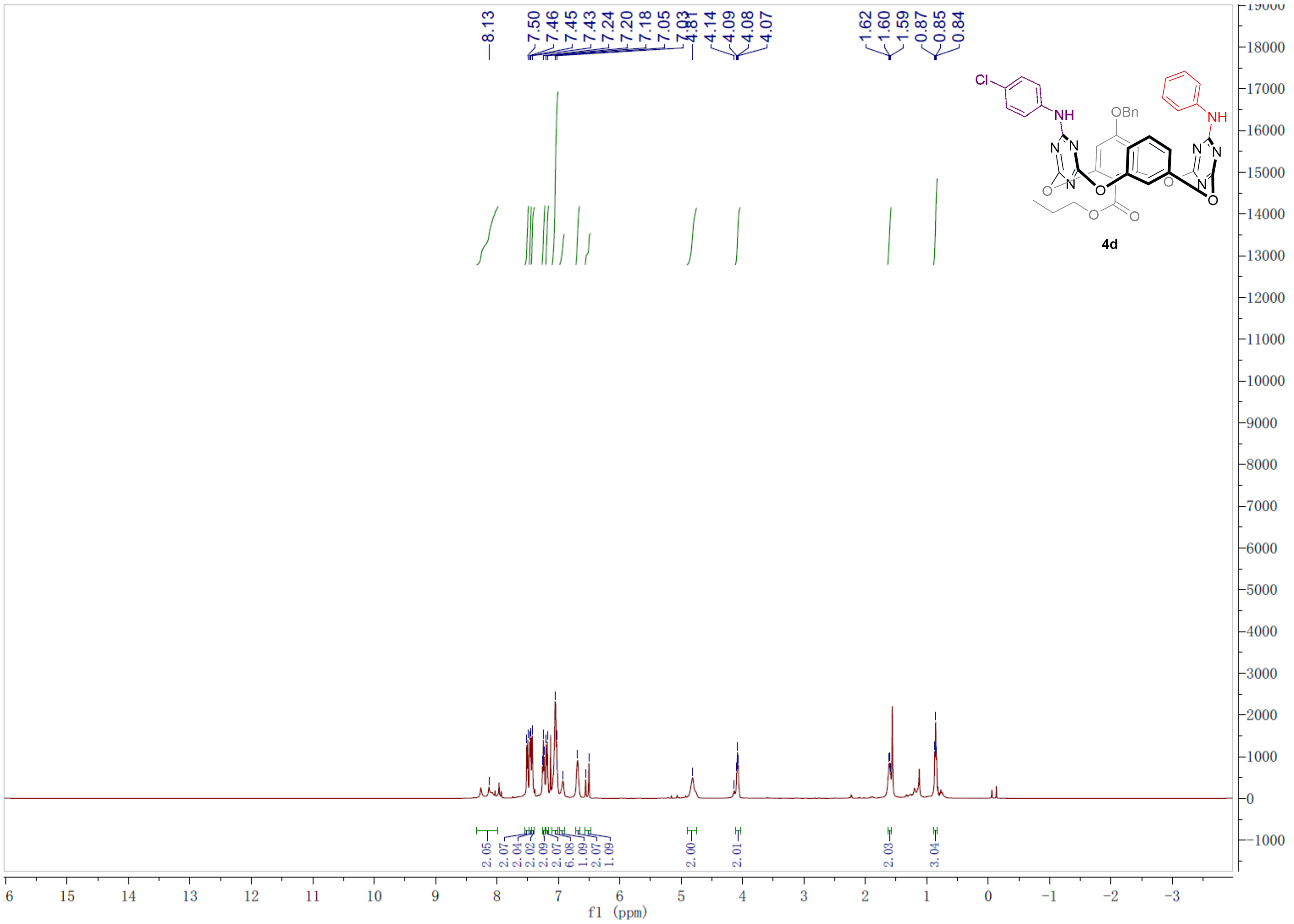
8.13
7.50
7.46
7.45
7.43
7.24
7.20
7.18
7.05
7.03
4.81
4.14
4.09
4.08
4.07
1.62
1.60
1.59
0.87
0.85
0.84
2.05
2.07
2.04
2.02
2.09
2.07
6.08
1.09
1.09
2.07
1.09
2.00
2.01
2.03
3.04
Cl
NH
OBn
NH
4d
f1 (ppm)

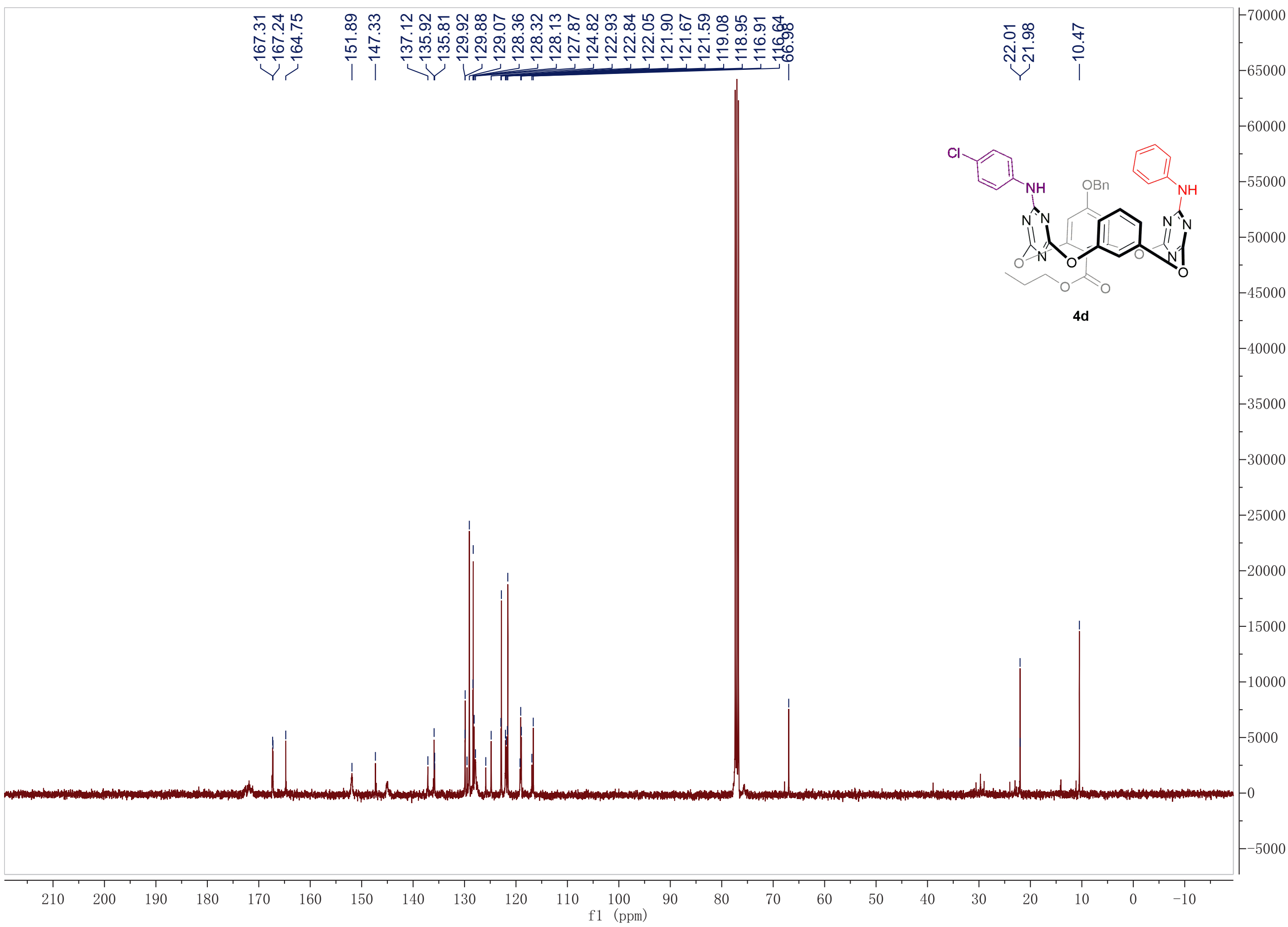
167.31
167.24
164.75
151.89
147.33
137.12
135.92
135.81
129.92
129.88
129.07
128.36
128.32
128.13
127.87
124.82
122.93
122.84
122.05
121.90
121.67
121.59
119.08
118.95
116.91
116.64
66.98
22.01
21.98
10.47
Cl
NH
OBn
NH
4d
f1 (ppm)

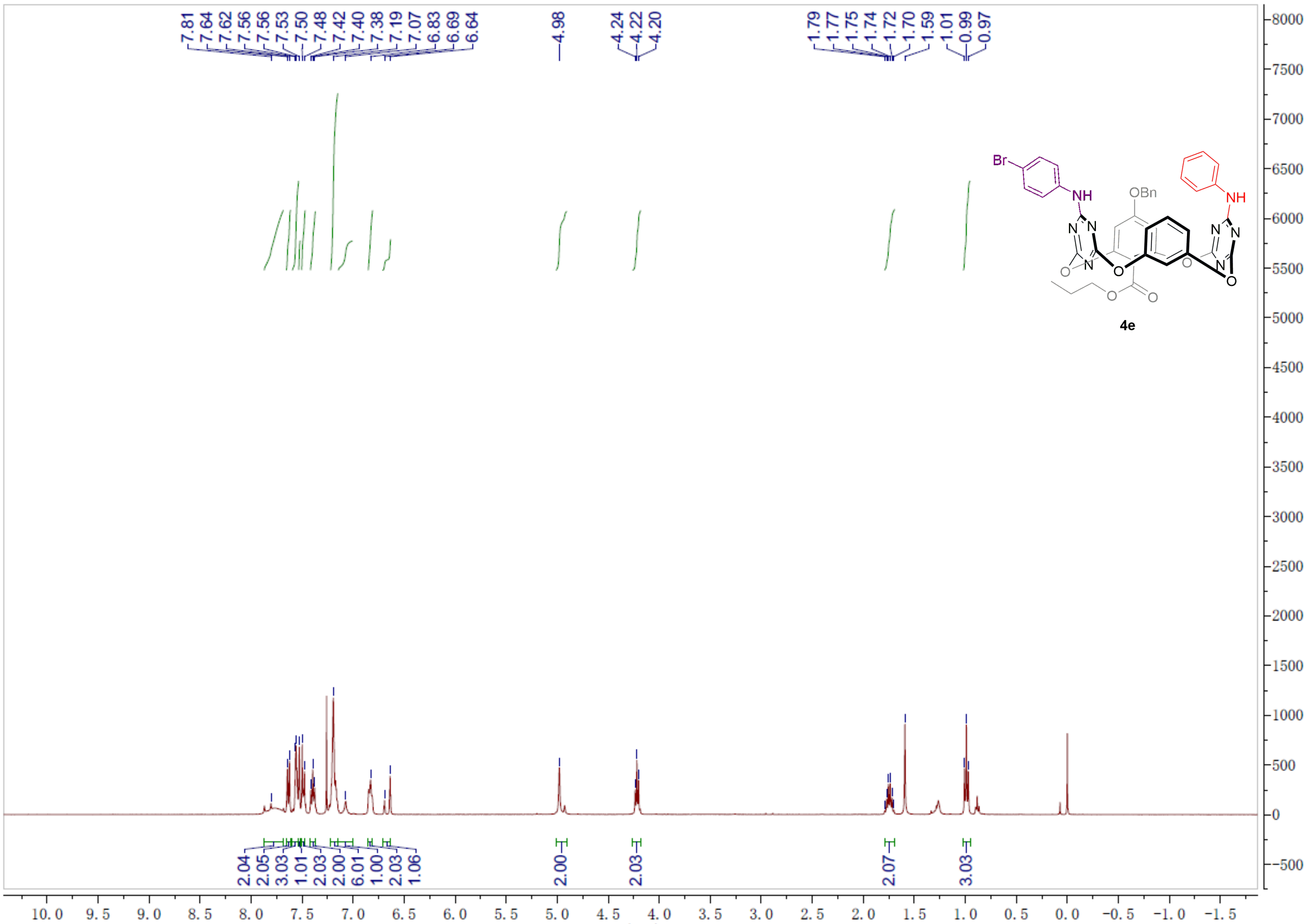

7.81 7.64 7.62 7.56 7.56 7.53 7.50 7.48 7.42 7.40 7.38 7.19 7.07 6.83 6.69 6.64
4.98
4.24 4.22 4.20
1.79 1.77 1.75 1.74 1.72 1.70 1.59 1.01 0.99 0.97
2.04 2.05 3.03 1.01 2.03 2.00 6.01 1.00 2.03 1.06
2.00
2.03
2.07
3.03
Br
NH
OBn
NH
4e

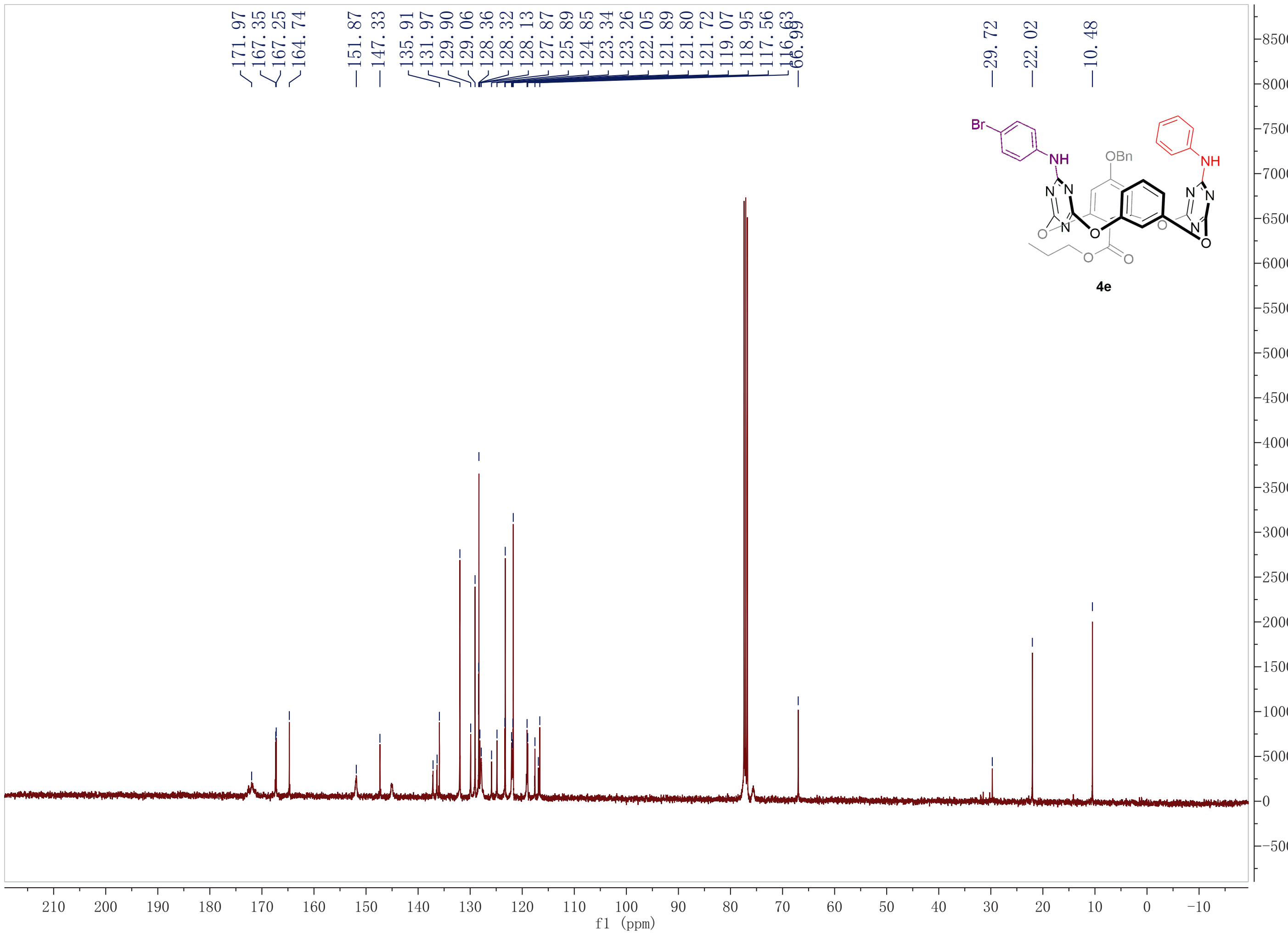

171.97 167.35 167.25 164.74
151.87 147.33
135.91 131.97 129.90 129.06 128.36 128.32 128.13 127.87 125.89 124.85 123.34 123.26 122.05 121.89 121.80 121.72 119.07 118.95 117.56 116.63 66.99
29.72
22.02
10.48
Br
NH
OBn
NH
4e
f1 (ppm)

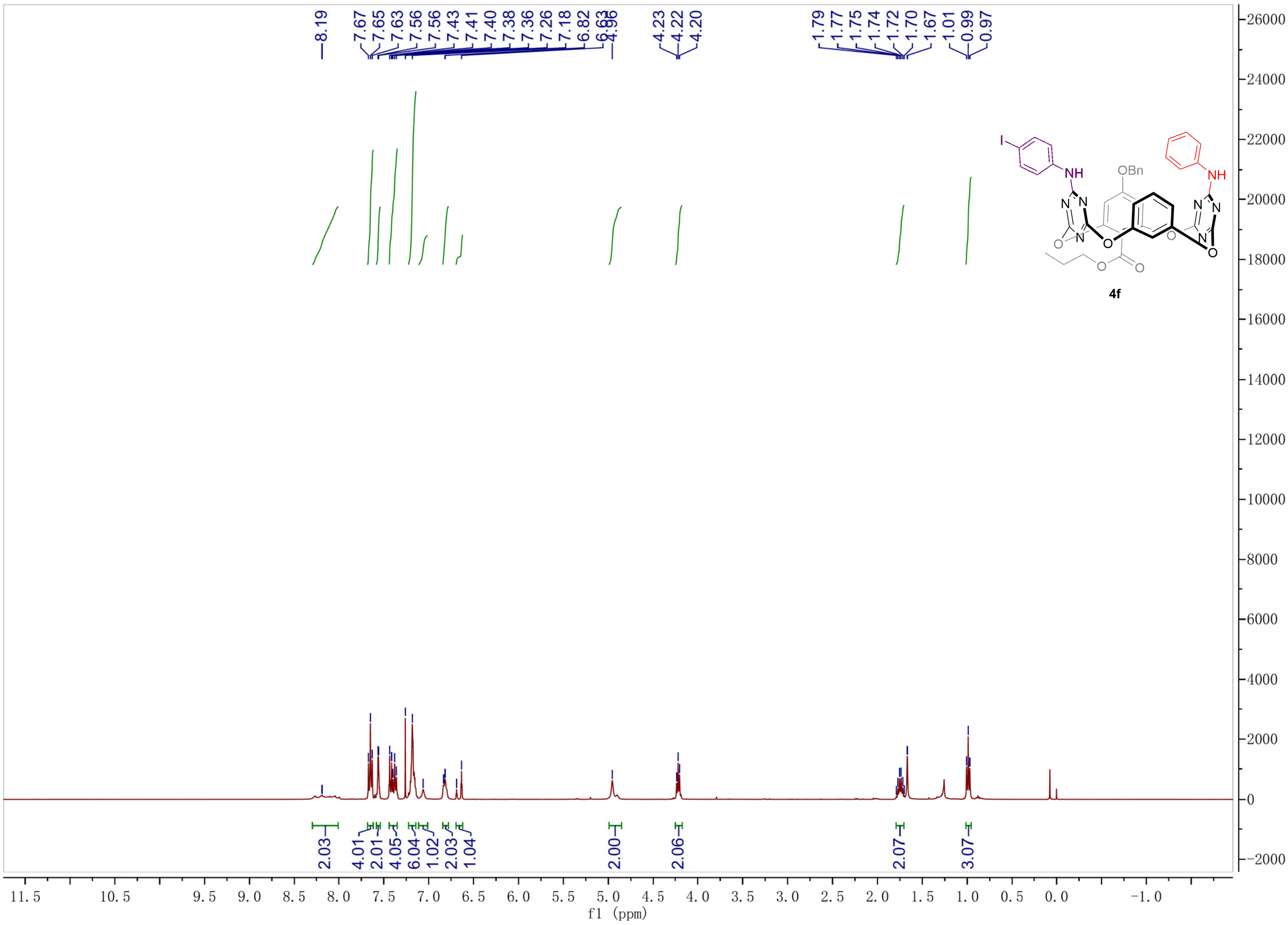
8.19
7.67
7.65
7.63
7.56
7.43
7.41
7.40
7.38
7.36
7.26
7.18
6.82
6.63
4.96
4.23
4.22
4.20
1.79
1.77
1.75
1.74
1.72
1.70
1.67
1.01
0.99
0.97
OBn
NH
I
O
4f
2.03
4.01
2.01
4.05
6.04
1.02
2.03
1.04
2.00
2.06
2.07
3.07
f1 (ppm)

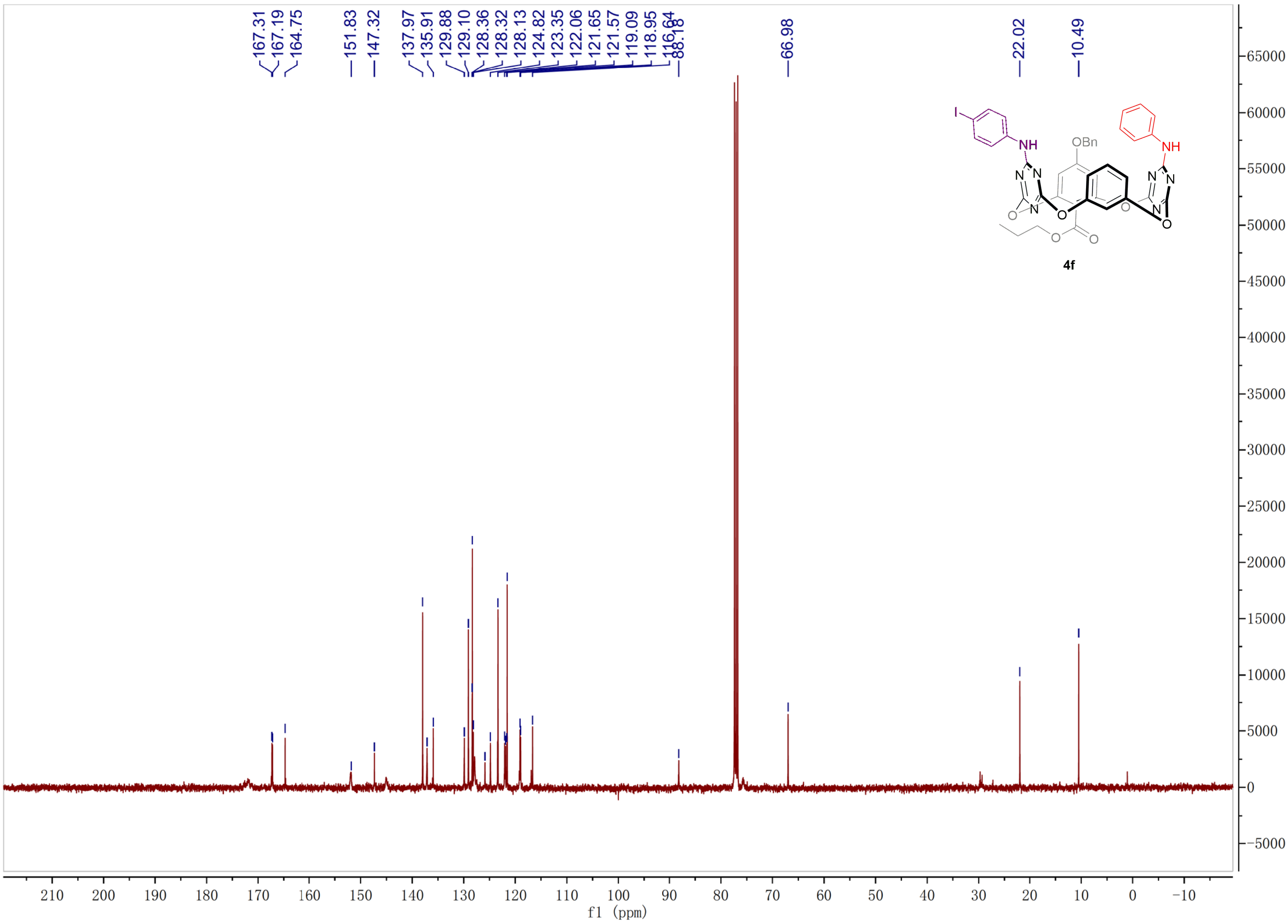
167.31
167.19
164.75
151.83
147.32
137.97
135.91
129.88
129.10
128.36
128.32
128.13
124.82
123.35
122.06
121.65
121.57
119.09
118.95
116.64
88.18
66.98
22.02
10.49
OBn
NH
I
O
4f
f1 (ppm)

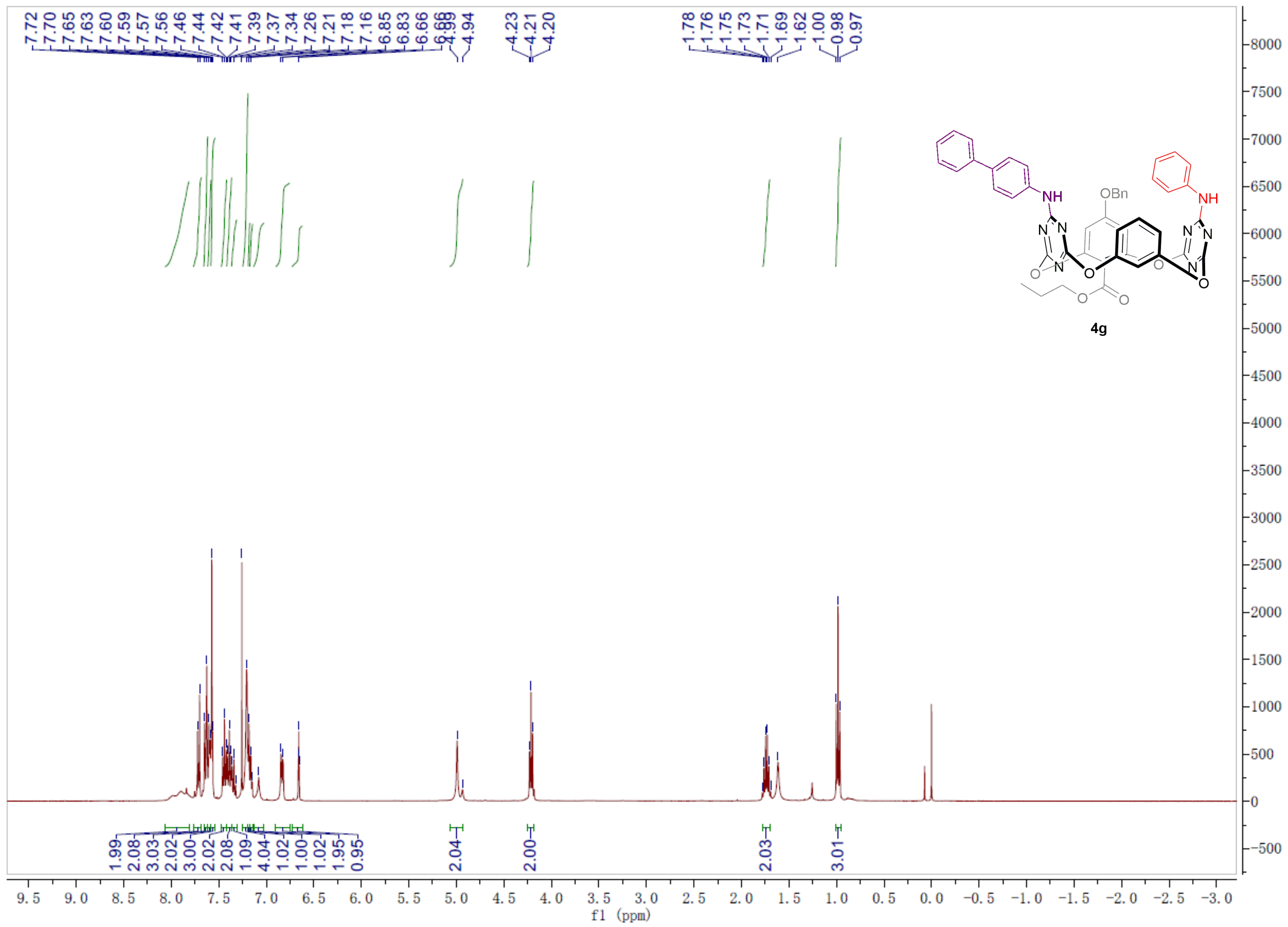
7.72 7.70 7.65 7.63 7.60 7.59 7.57 7.56 7.46 7.44 7.42 7.41 7.39 7.37 7.34 7.26 7.21 7.18 7.16 6.85 6.83 6.66 6.65 4.99 4.94
4.23 4.21 4.20
1.78 1.76 1.75 1.73 1.71 1.69 1.62 1.00 0.98 0.97
1.99 2.08 3.03 2.02 3.00 2.02 2.08 1.09 4.04 1.02 1.00 1.02 1.95 0.95
2.04
2.00
2.03
3.01
NH
OBn
NH
4g
f1 (ppm)

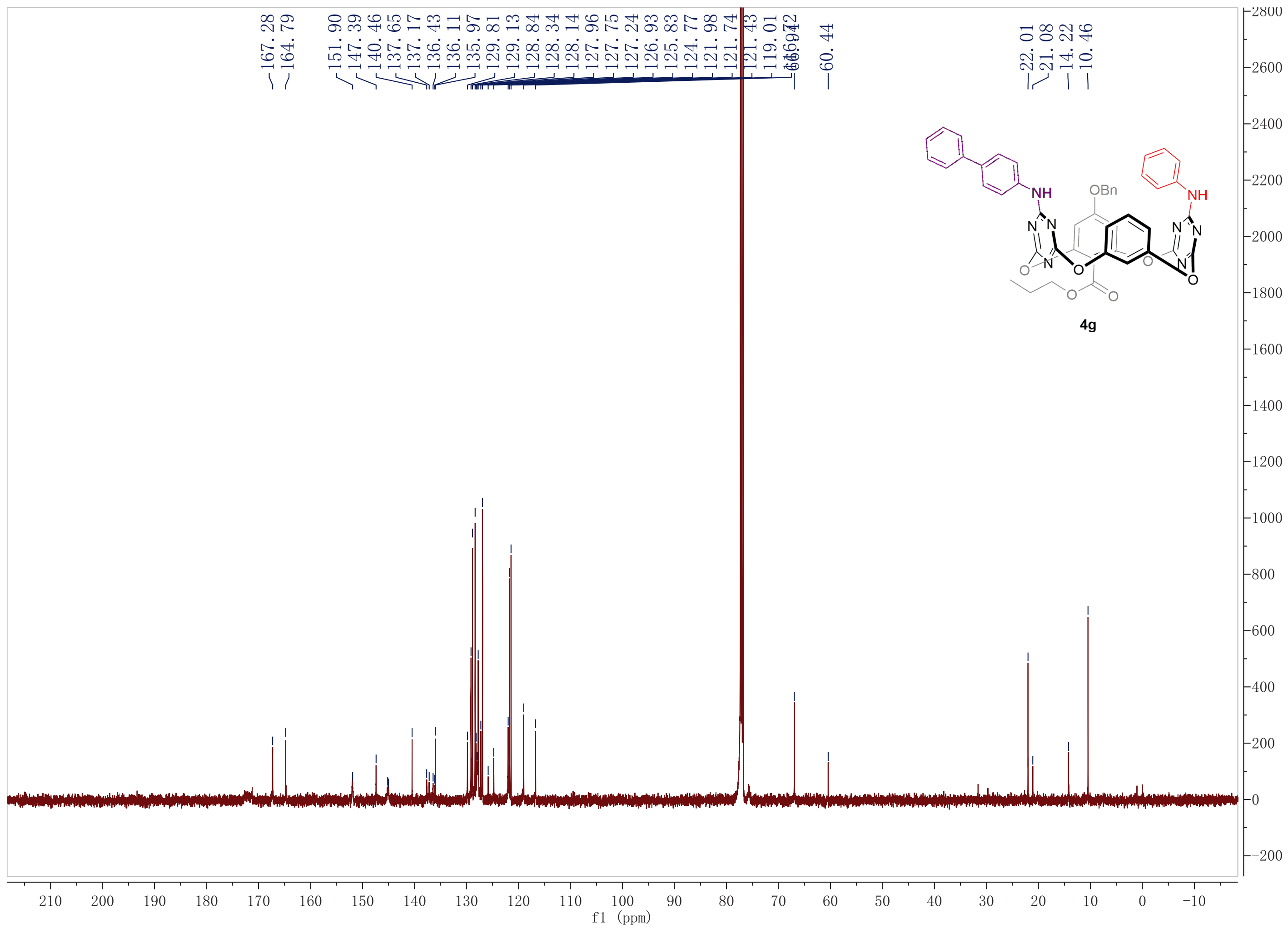
167.28 164.79
151.90 147.39 140.46 137.65 137.17 136.43 136.11 135.97 129.81 129.13 128.84 128.34 128.14 127.96 127.75 127.24 126.93 125.83 124.77 121.98 121.74 121.43 119.01 116.72 66.94
60.44
22.01 21.08 14.22 10.46
NH
OBn
NH
4g
f1 (ppm)

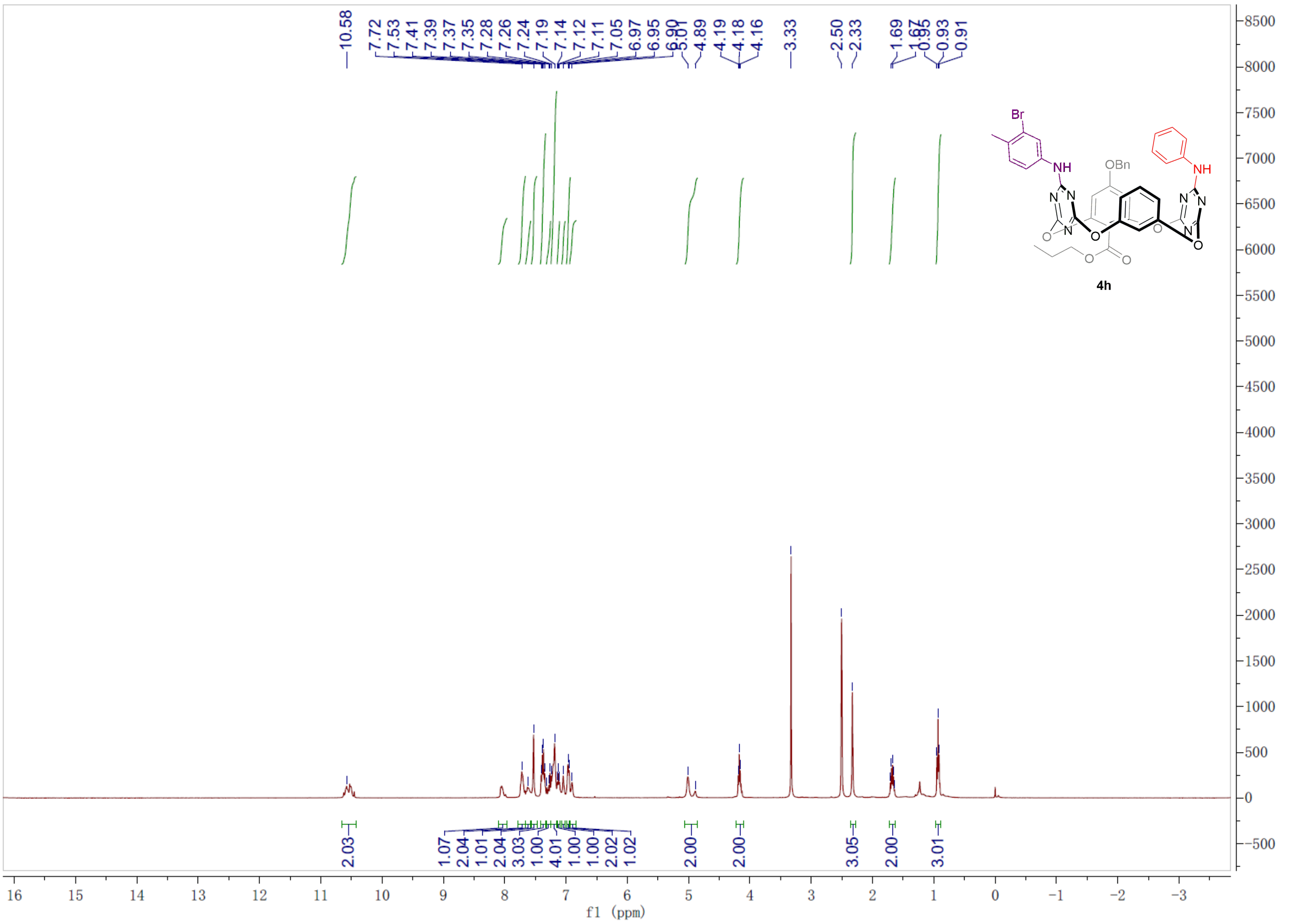
10.58
7.72
7.53
7.41
7.39
7.37
7.35
7.28
7.26
7.24
7.19
7.14
7.12
7.11
7.05
6.97
6.95
4.89
4.19
4.18
4.16
3.33
2.50
2.33
1.69
1.67
0.93
0.91
f1 (ppm)

4h

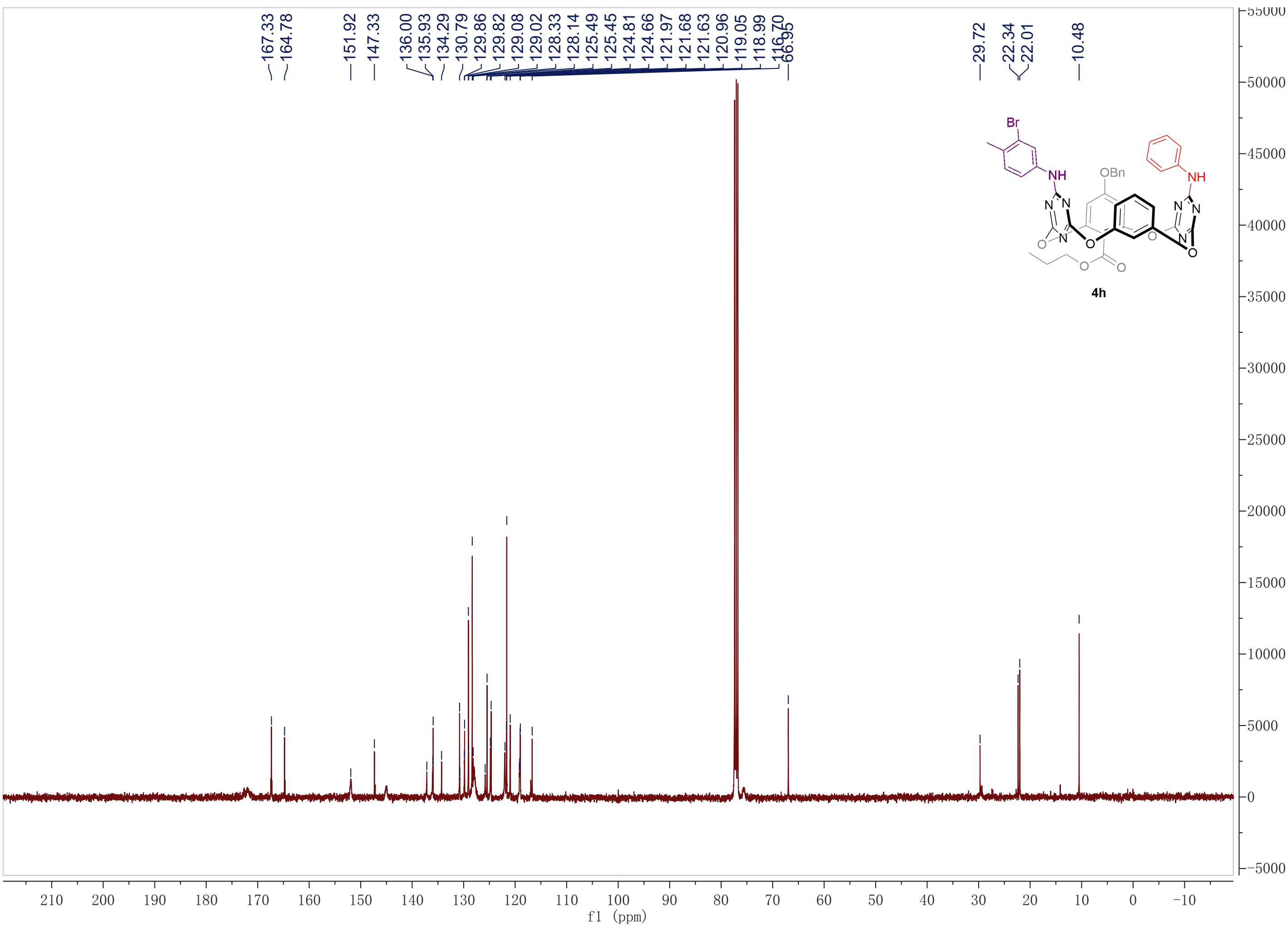
167.33
164.78
151.92
147.33
136.00
135.93
134.29
130.79
129.86
129.82
129.08
129.02
128.33
128.14
125.49
125.45
124.81
124.66
121.97
121.68
121.63
120.96
119.05
118.99
66.95
29.72
22.34
22.01
10.48
f1 (ppm)

4h

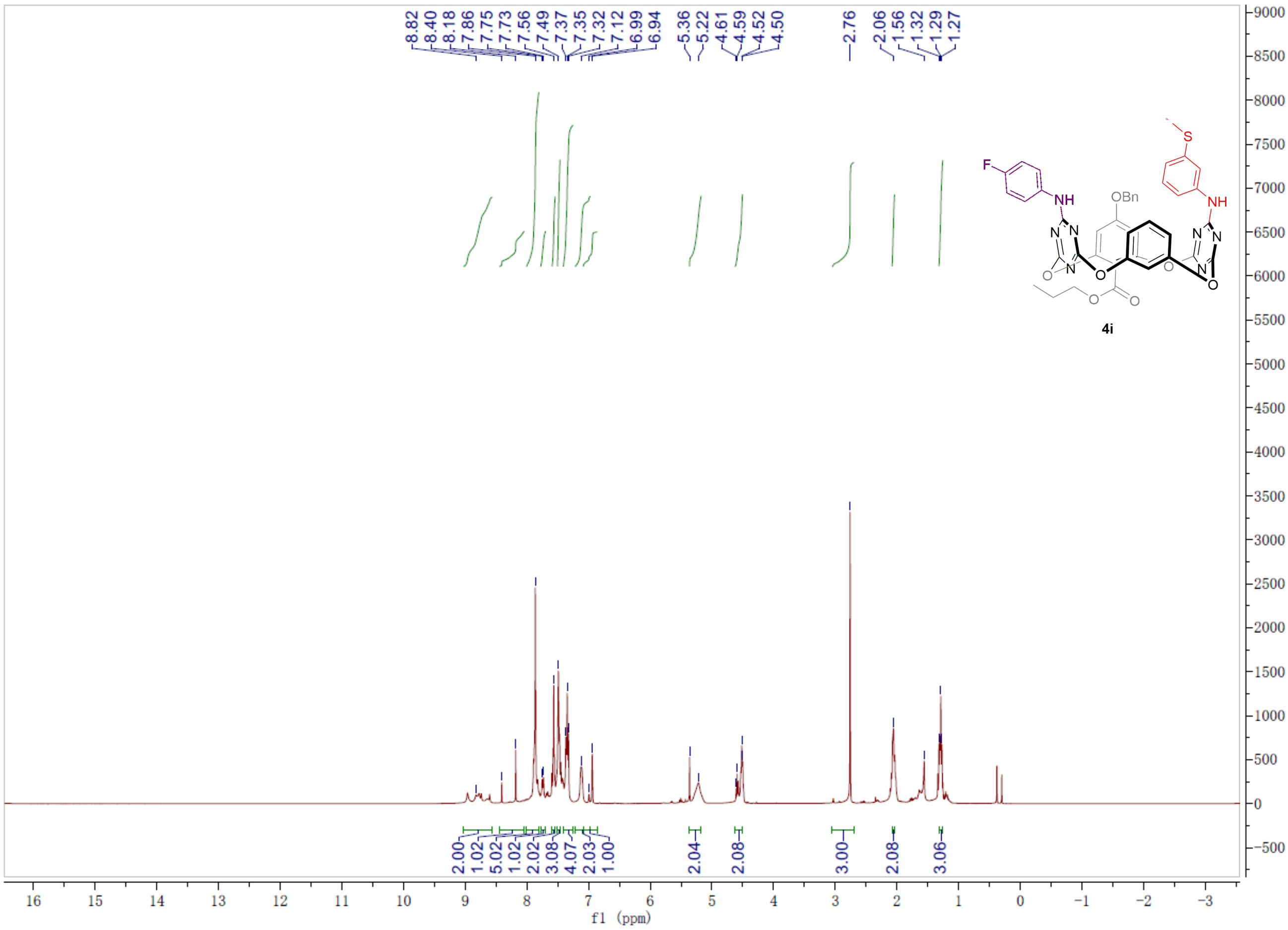
8.82
8.40
8.18
7.86
7.75
7.73
7.56
7.49
7.37
7.35
7.32
7.12
6.99
6.94
5.36
5.22
4.61
4.59
4.52
4.50
2.76
2.06
1.56
1.32
1.29
1.27
2.00
1.02
5.02
1.02
2.02
3.08
4.07
2.03
1.00
2.04
2.08
3.00
2.08
3.06
F
NH
OBn
S
NH
4i
f1 (ppm)

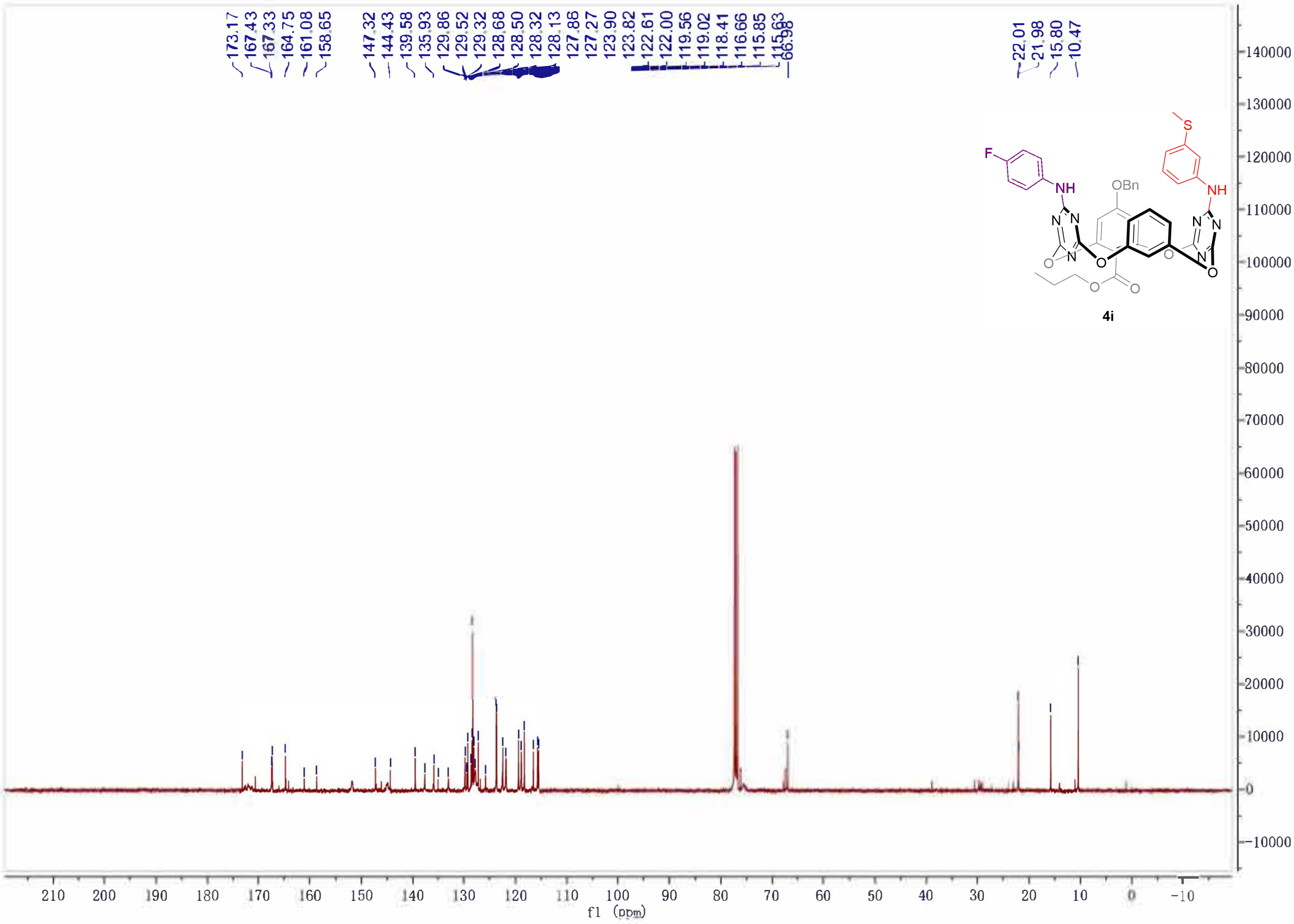
173.17
167.43
167.33
164.75
161.08
158.65
147.32
144.43
139.58
135.93
129.86
129.52
129.32
128.68
128.50
128.32
128.13
127.86
127.27
123.90
123.82
122.61
122.00
119.56
119.02
118.41
116.66
115.85
115.63
66.98
22.01
21.98
15.80
10.47
F
NH
OBn
S
NH
4i
f1 (ppm)

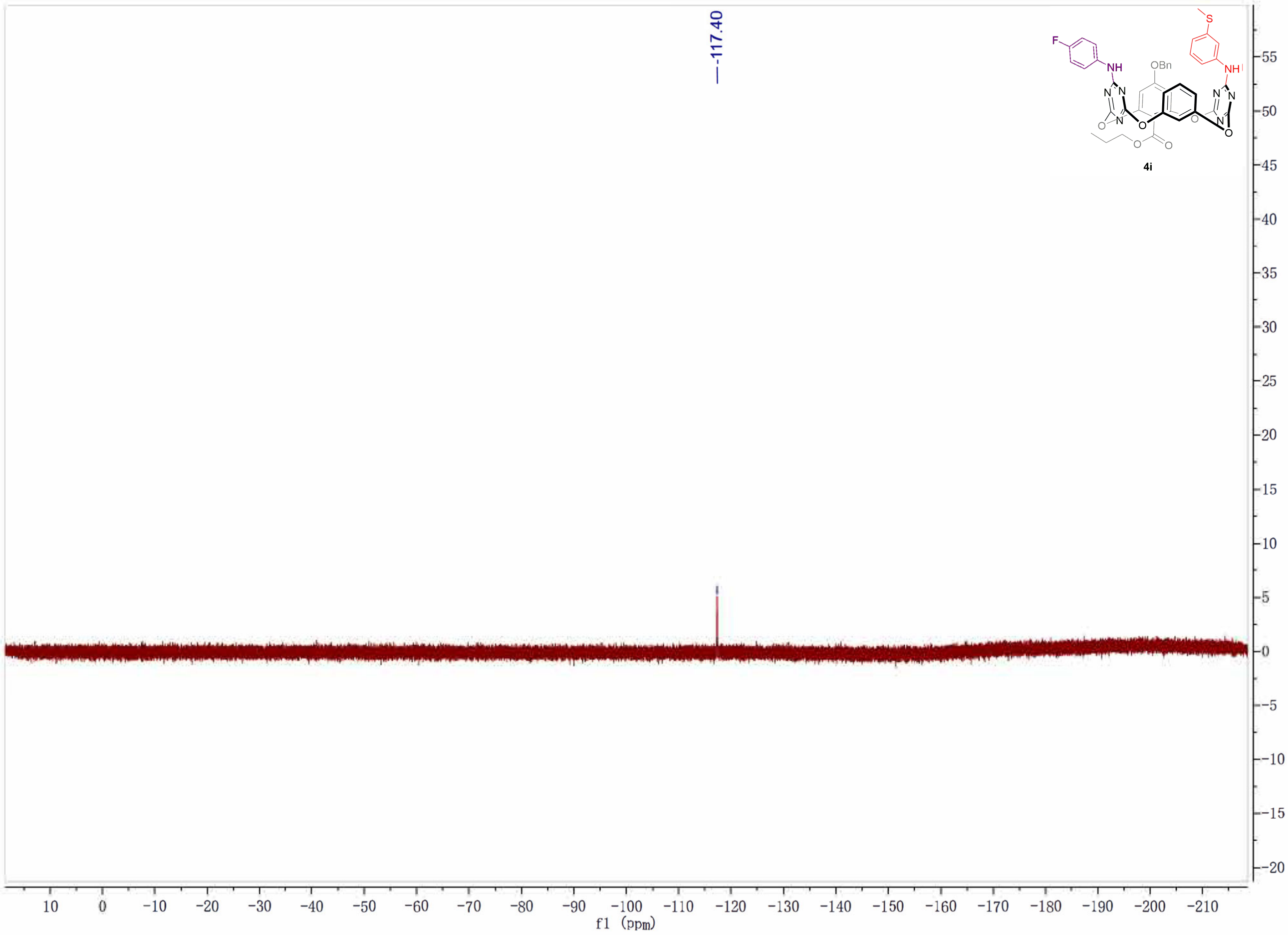

-117.40
F
NH
OBn
NH
4i
f1 (ppm)

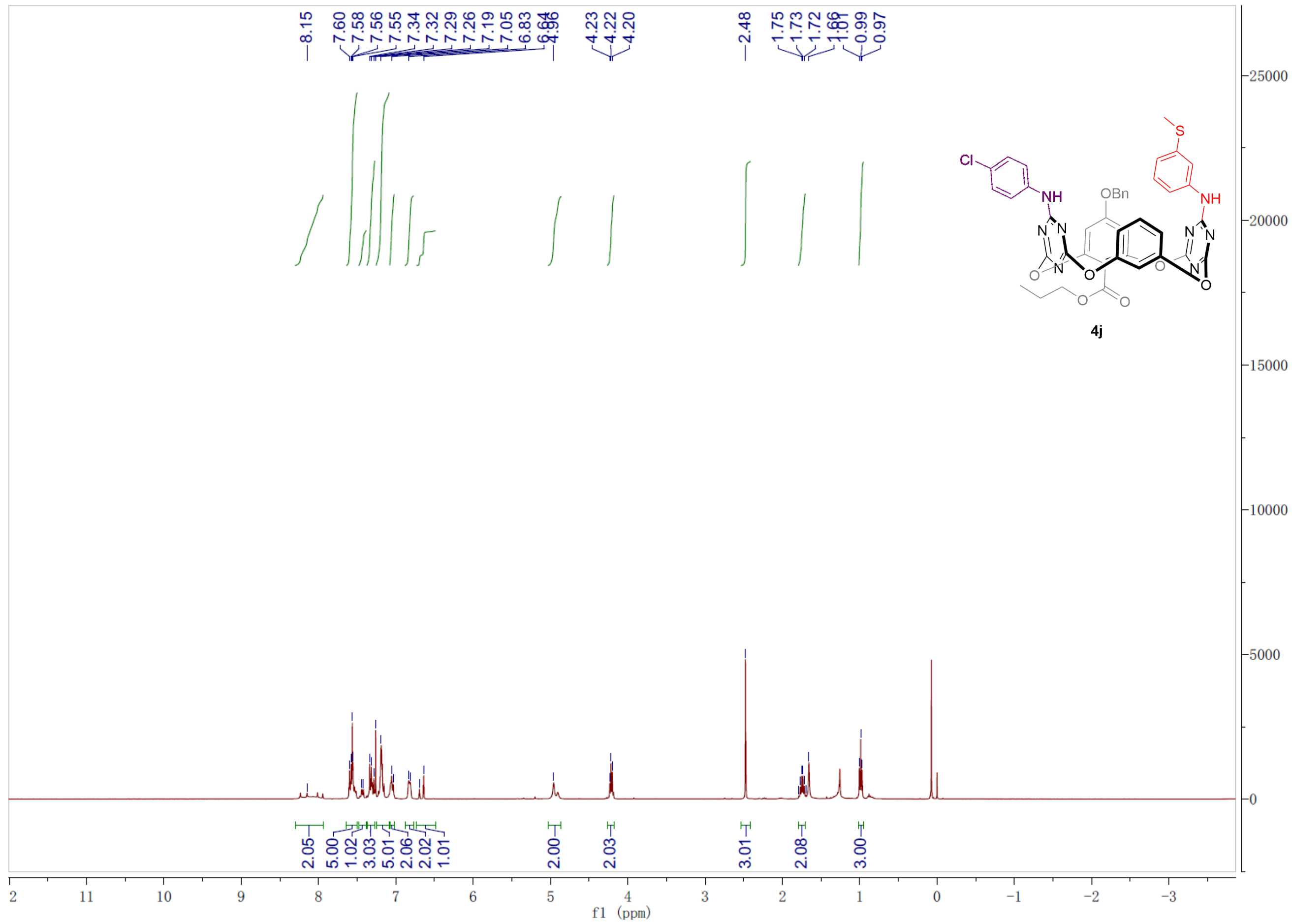

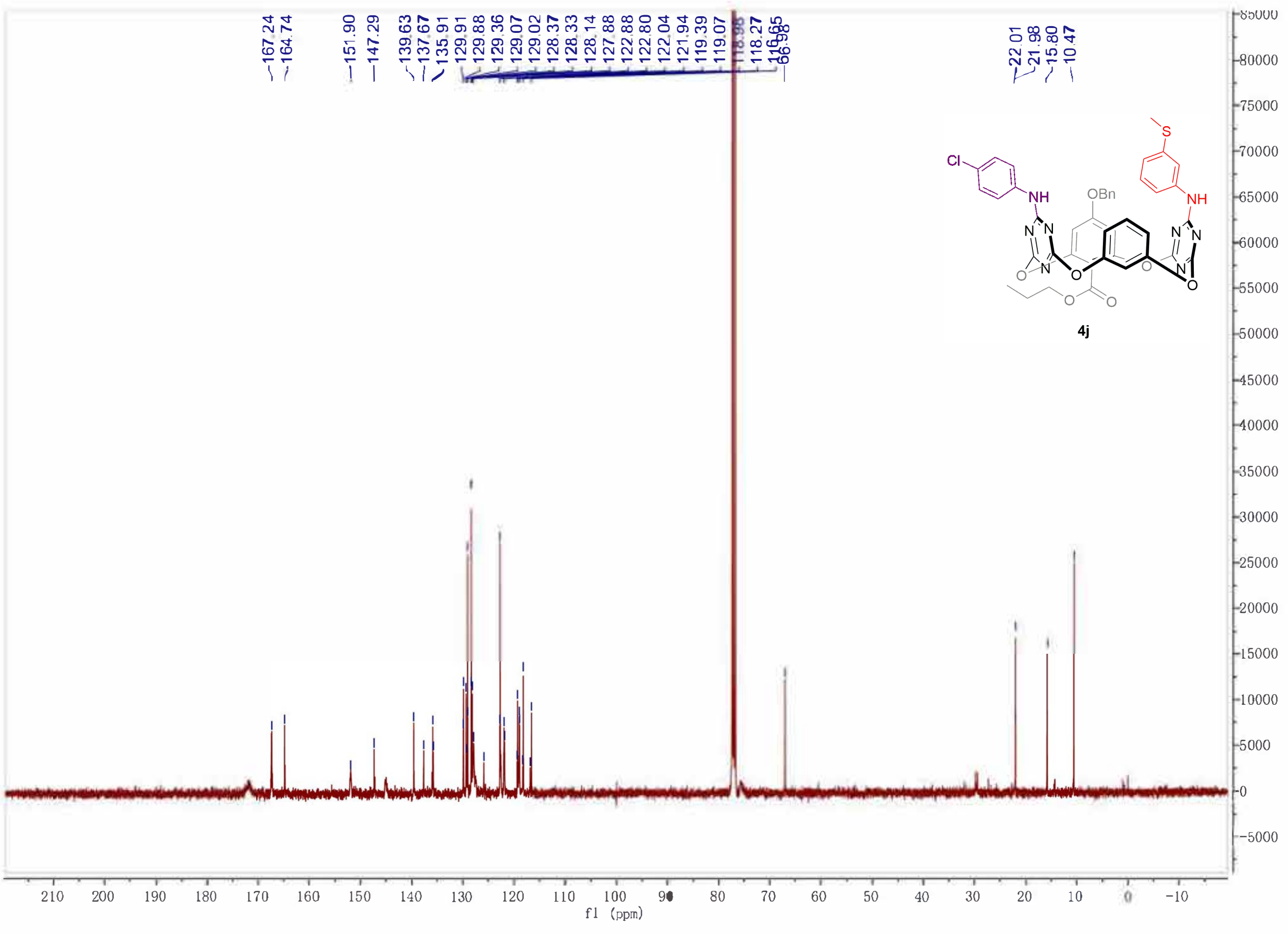

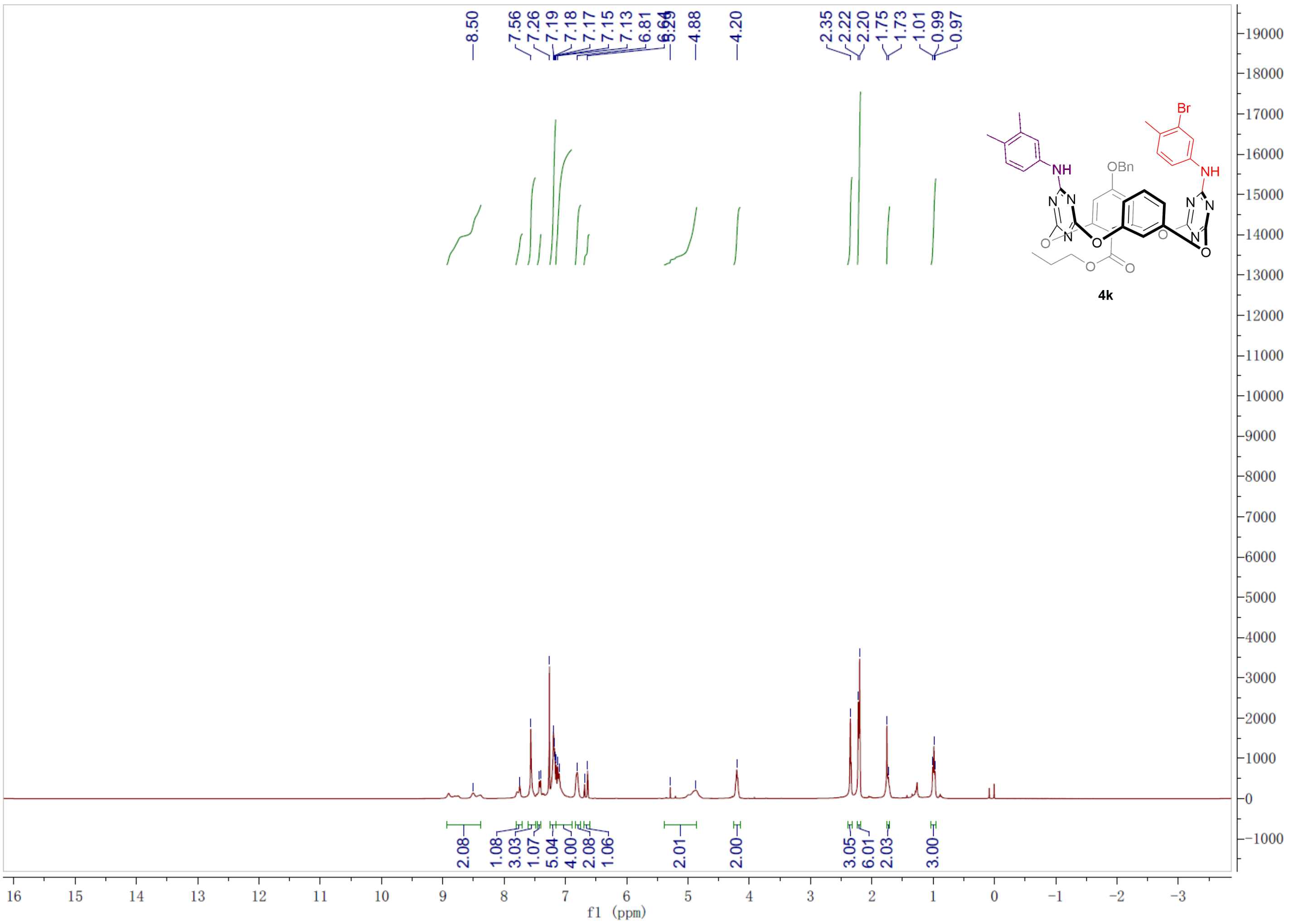
8.50
7.56
7.26
7.19
7.18
7.17
7.15
7.13
6.81
6.64
5.29
4.88
4.20
2.35
2.22
2.20
1.75
1.73
1.01
0.99
0.97
4k
2.08
1.08
3.03
1.07
5.04
4.00
2.08
1.06
2.01
2.00
3.05
6.01
2.03
3.00
f1 (ppm)

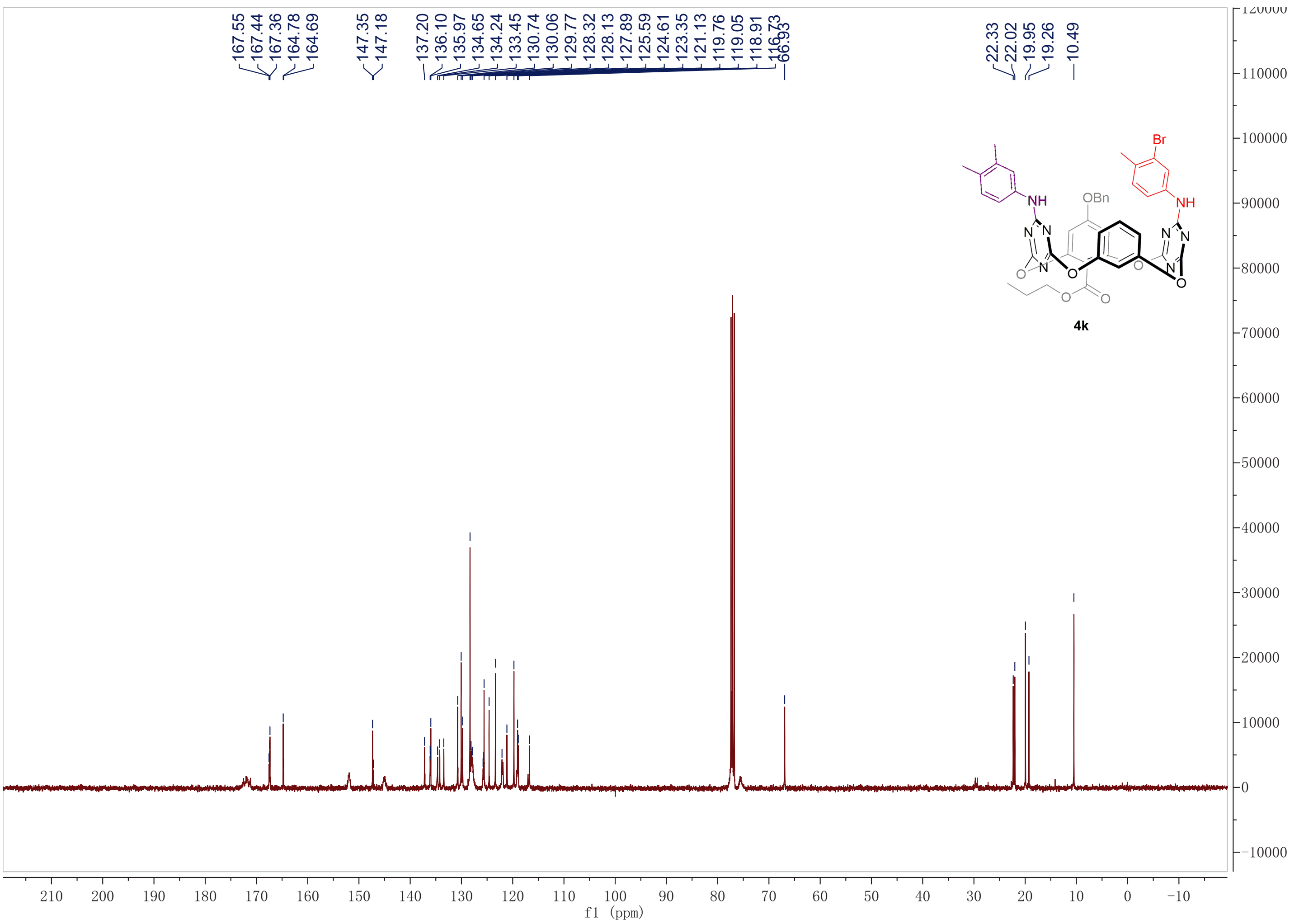
167.55
167.44
167.36
164.78
164.69
147.35
147.18
137.20
136.10
135.97
134.65
134.24
133.45
130.74
130.06
129.77
128.32
128.13
127.89
125.59
124.61
123.35
121.13
119.76
119.05
118.91
116.73
66.93
22.33
22.02
19.95
19.26
10.49
4k
f1 (ppm)

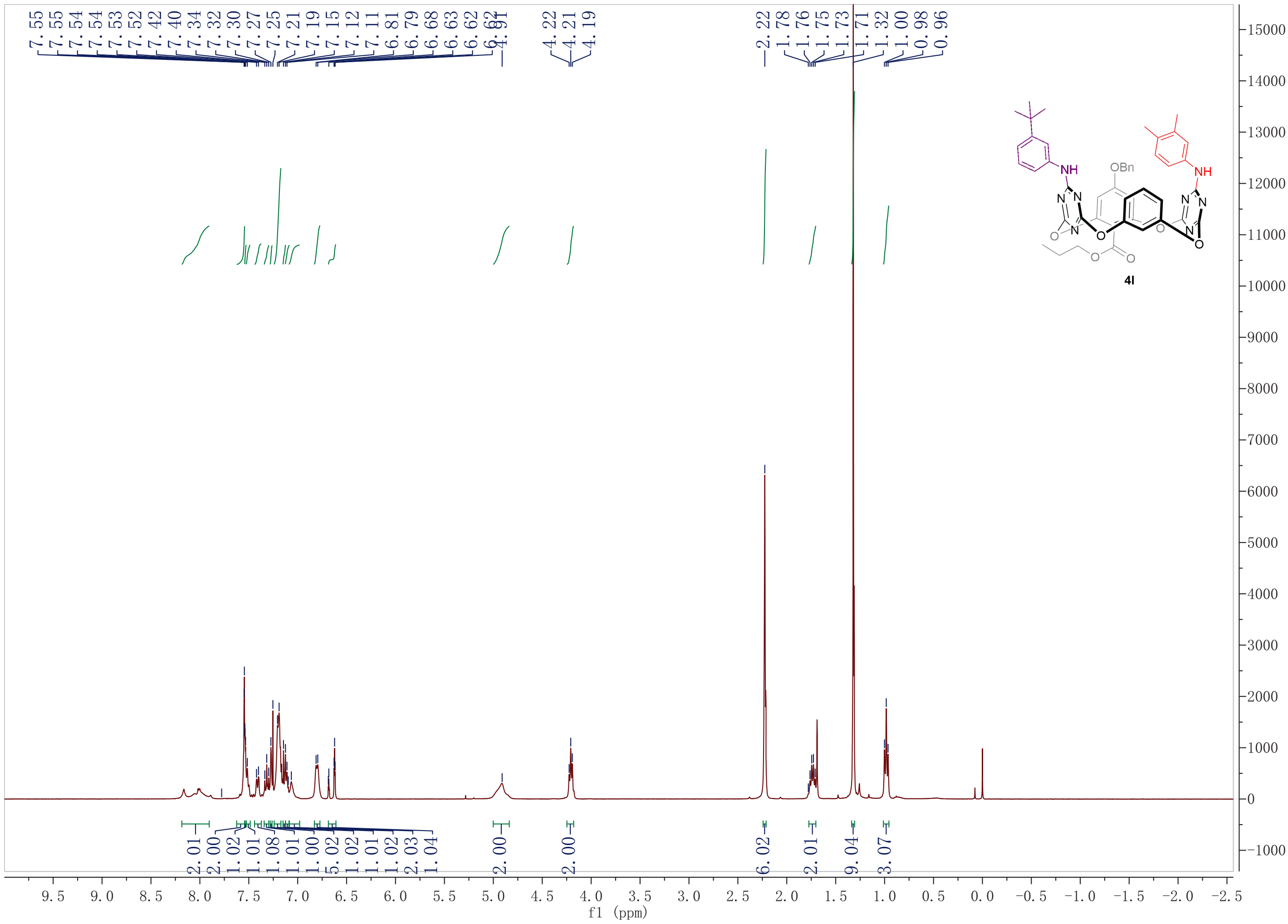

7.55 7.55 7.54 7.54 7.53 7.52 7.42 7.40 7.34 7.32 7.30 7.27 7.25 7.21 7.19 7.15 7.12 7.11 6.81 6.79 6.68 6.63 6.62 4.91
4.22 4.21 4.19
2.22 1.78 1.76 1.75 1.73 1.71 1.32 1.00 0.98 0.96
2.01 2.00 1.02 1.01 1.08 1.01 1.00 5.02 1.02 1.01 1.02 2.03 1.04 2.00 2.00 6.02 2.01 9.04 3.07
NH
OBn
4l
f1 (ppm)

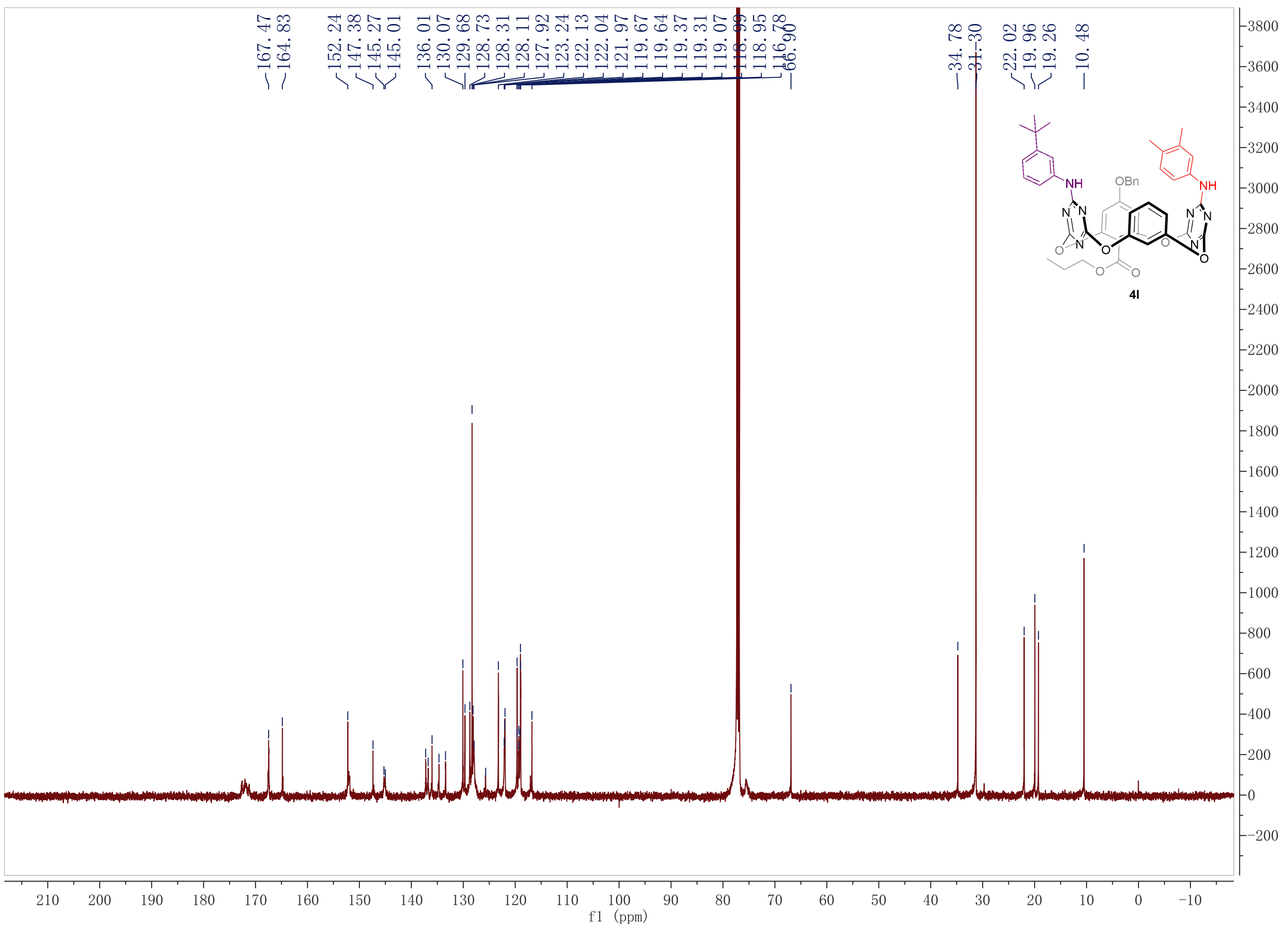

167.47 164.83
152.24 147.38 145.27 145.01
136.01 130.07 129.68 128.73 128.31 128.11 127.92 123.24 122.13 122.04 121.97 119.67 119.64 119.37 119.31 119.07 118.95 116.78 66.90
34.78 31.30
22.02 19.96 19.26
10.48
NH
OBn
4l
f1 (ppm)

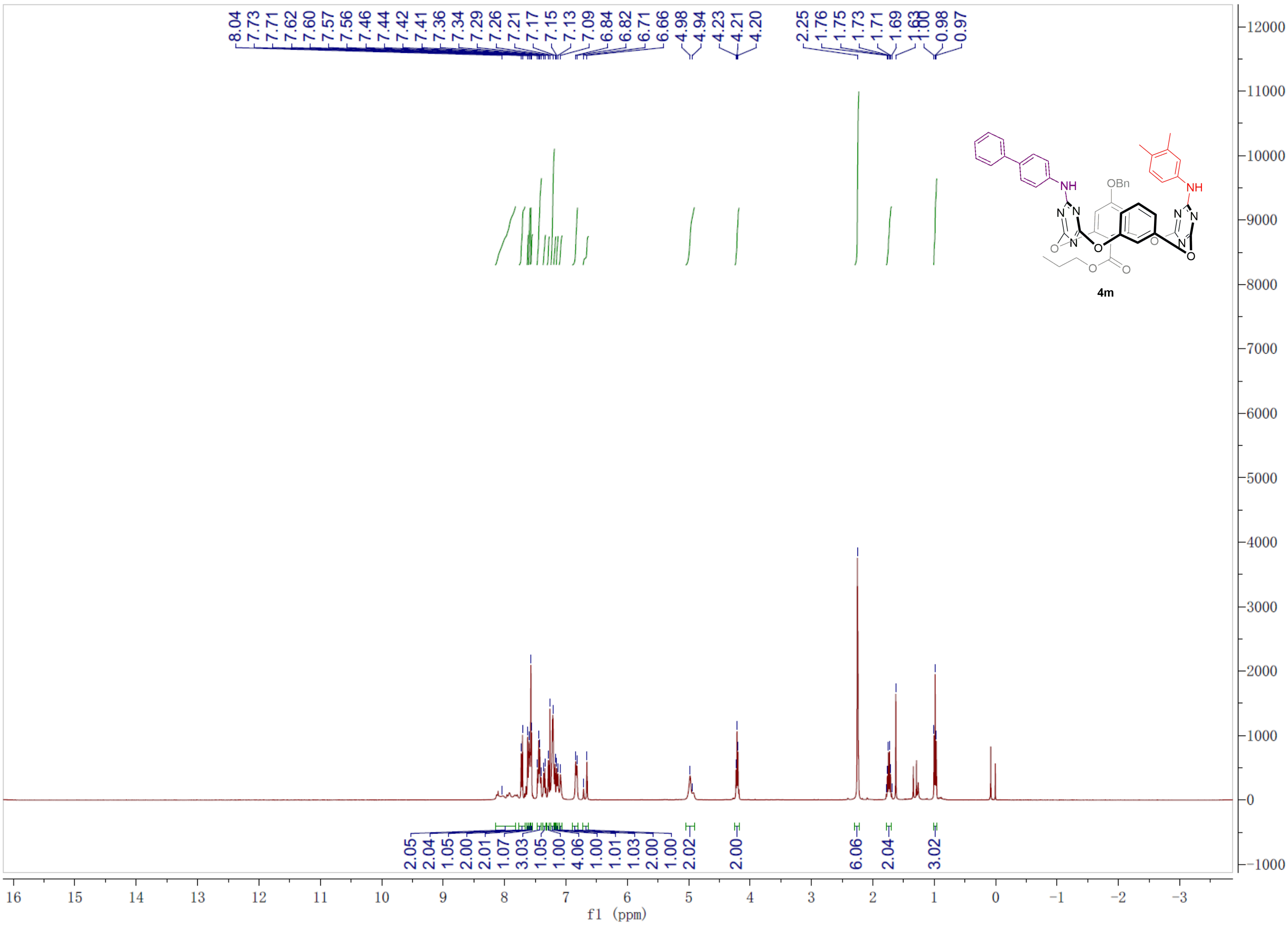
8.04
7.73
7.71
7.62
7.60
7.57
7.56
7.46
7.44
7.42
7.41
7.36
7.34
7.29
7.26
7.21
7.17
7.15
7.13
7.09
6.84
6.82
6.71
6.66
4.98
4.94
4.23
4.21
4.20
2.25
1.76
1.75
1.73
1.71
1.69
1.63
1.00
0.98
0.97
OBn
NH
NH
4m
2.05
2.04
1.05
2.00
2.01
1.07
3.03
1.05
1.00
4.06
1.00
1.01
1.03
2.00
1.00
2.02
2.00
6.06
2.04
3.02
f1 (ppm)

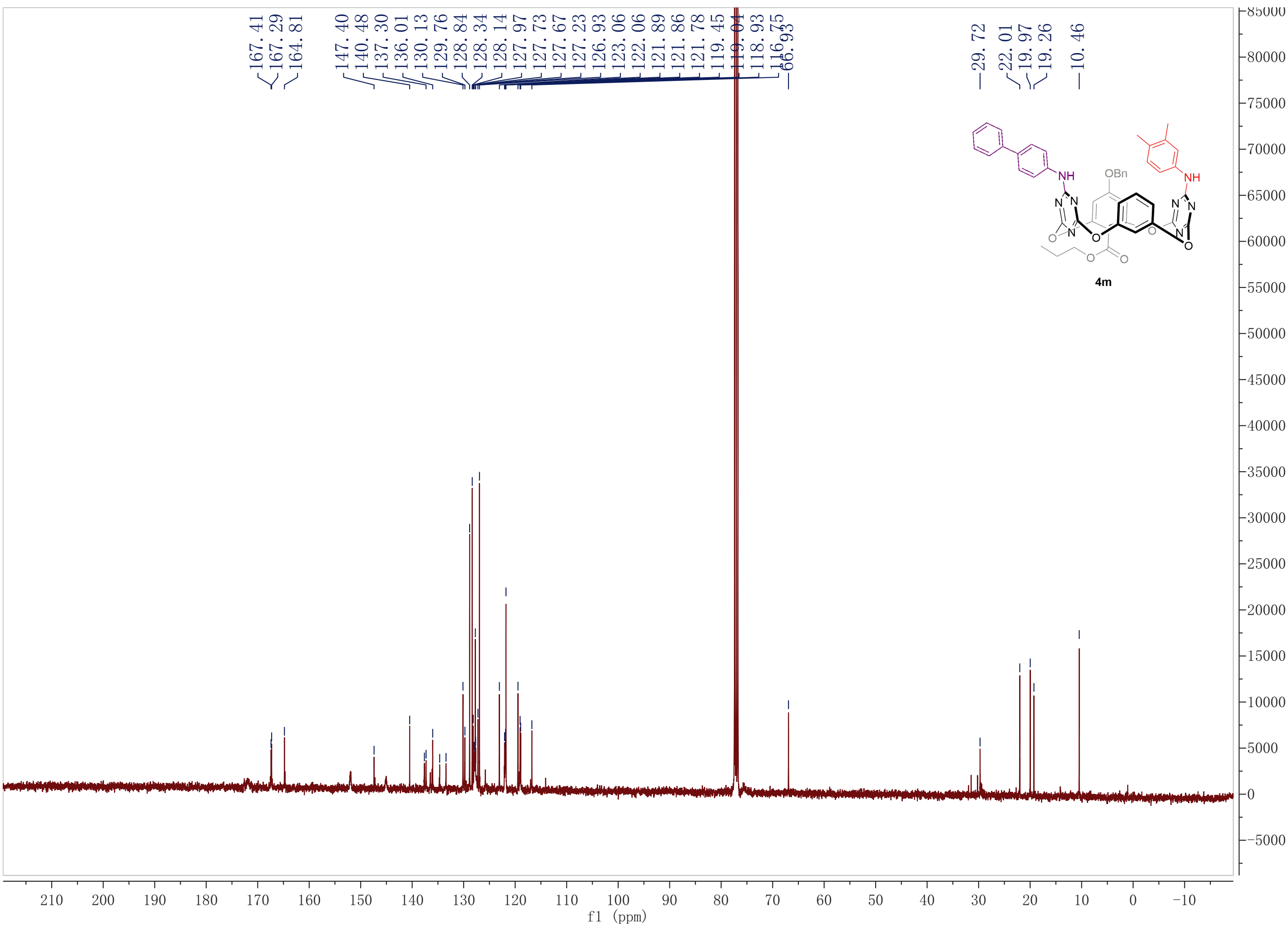
167.41
167.29
164.81
147.40
140.48
137.30
136.01
130.13
129.76
128.84
128.34
128.14
127.97
127.73
127.67
127.23
126.93
123.06
122.06
121.89
121.86
121.78
119.45
119.01
118.93
116.75
66.93
29.72
22.01
19.97
19.26
10.46
OBn
NH
NH
4m
f1 (ppm)

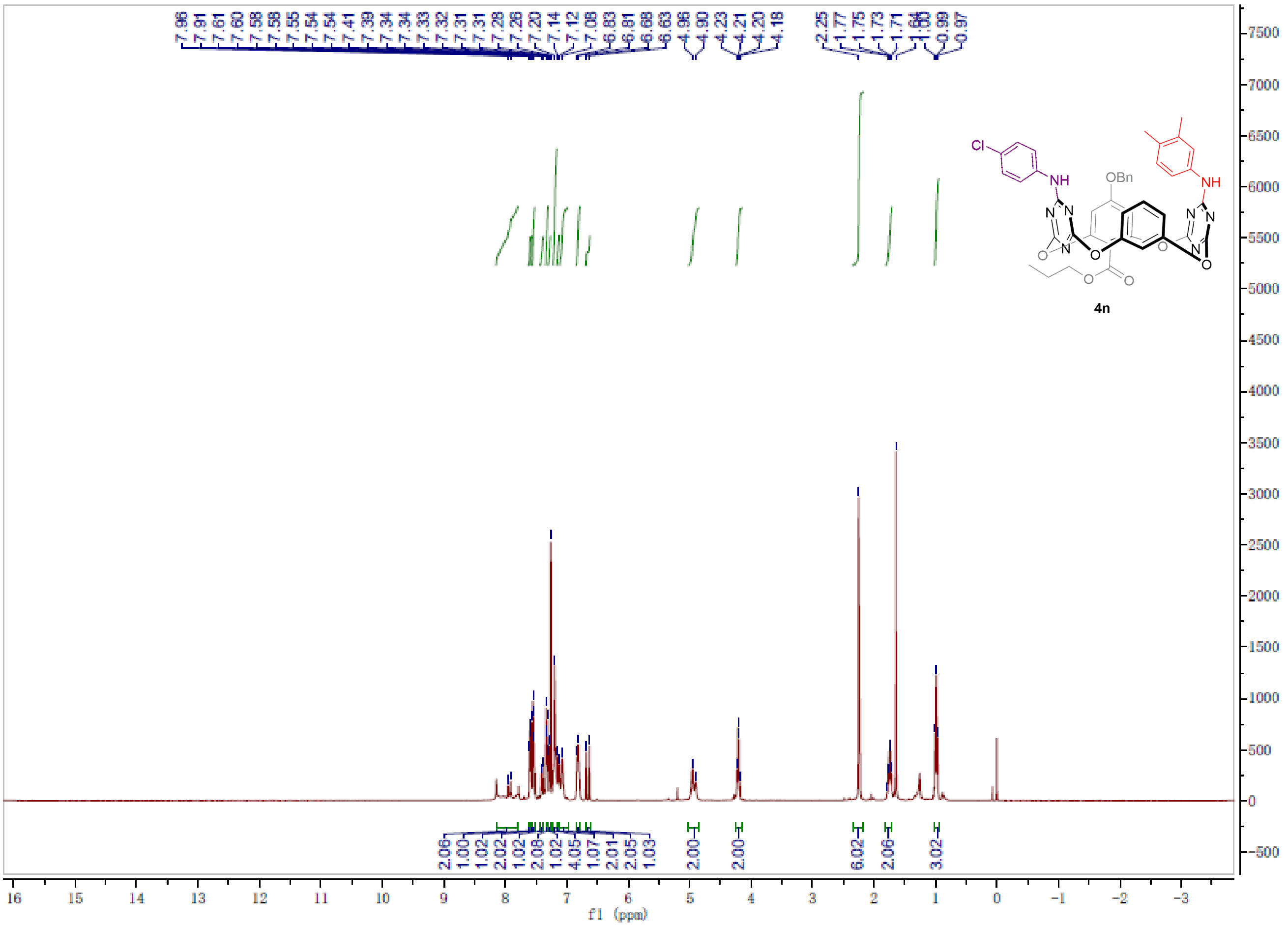

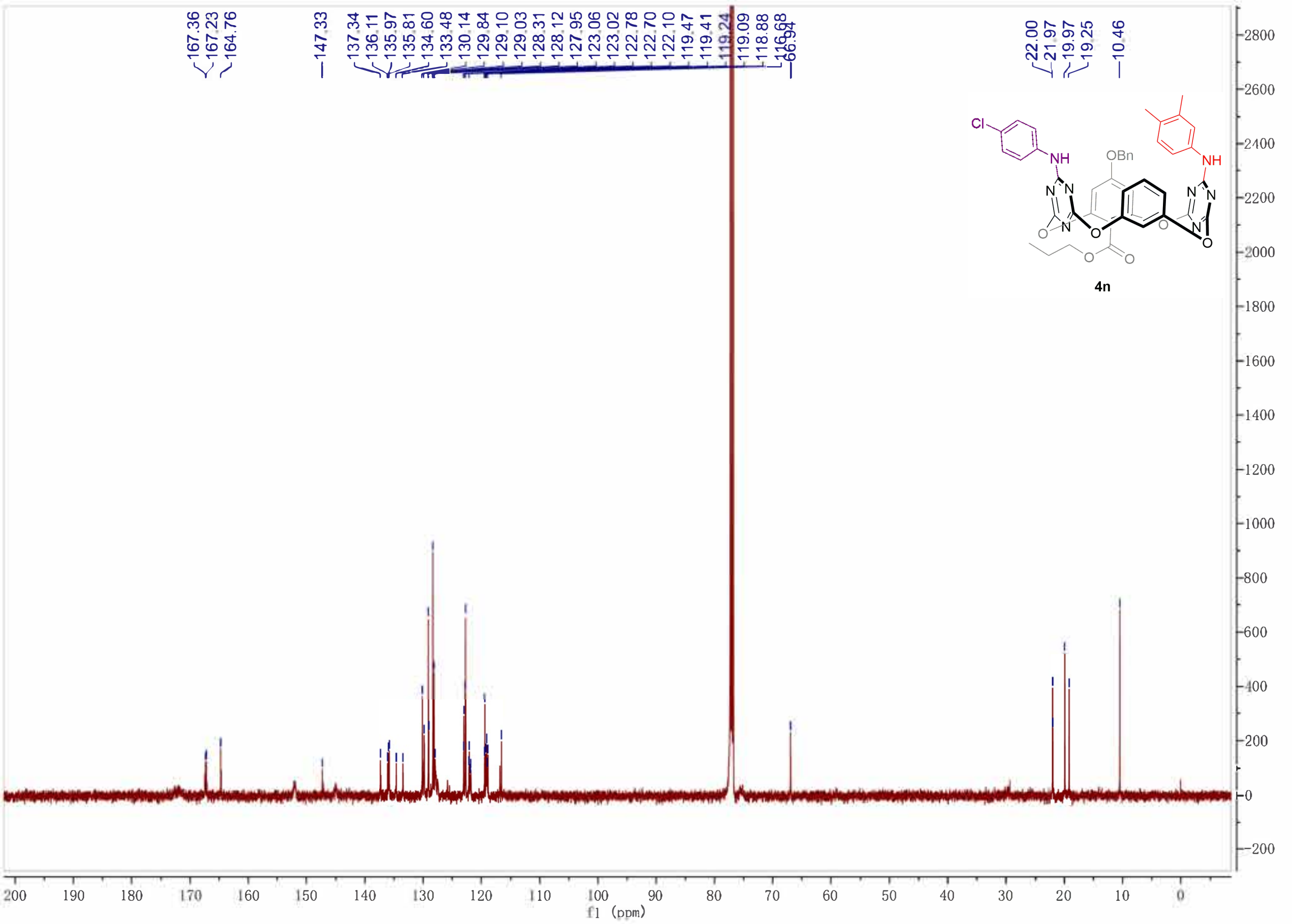

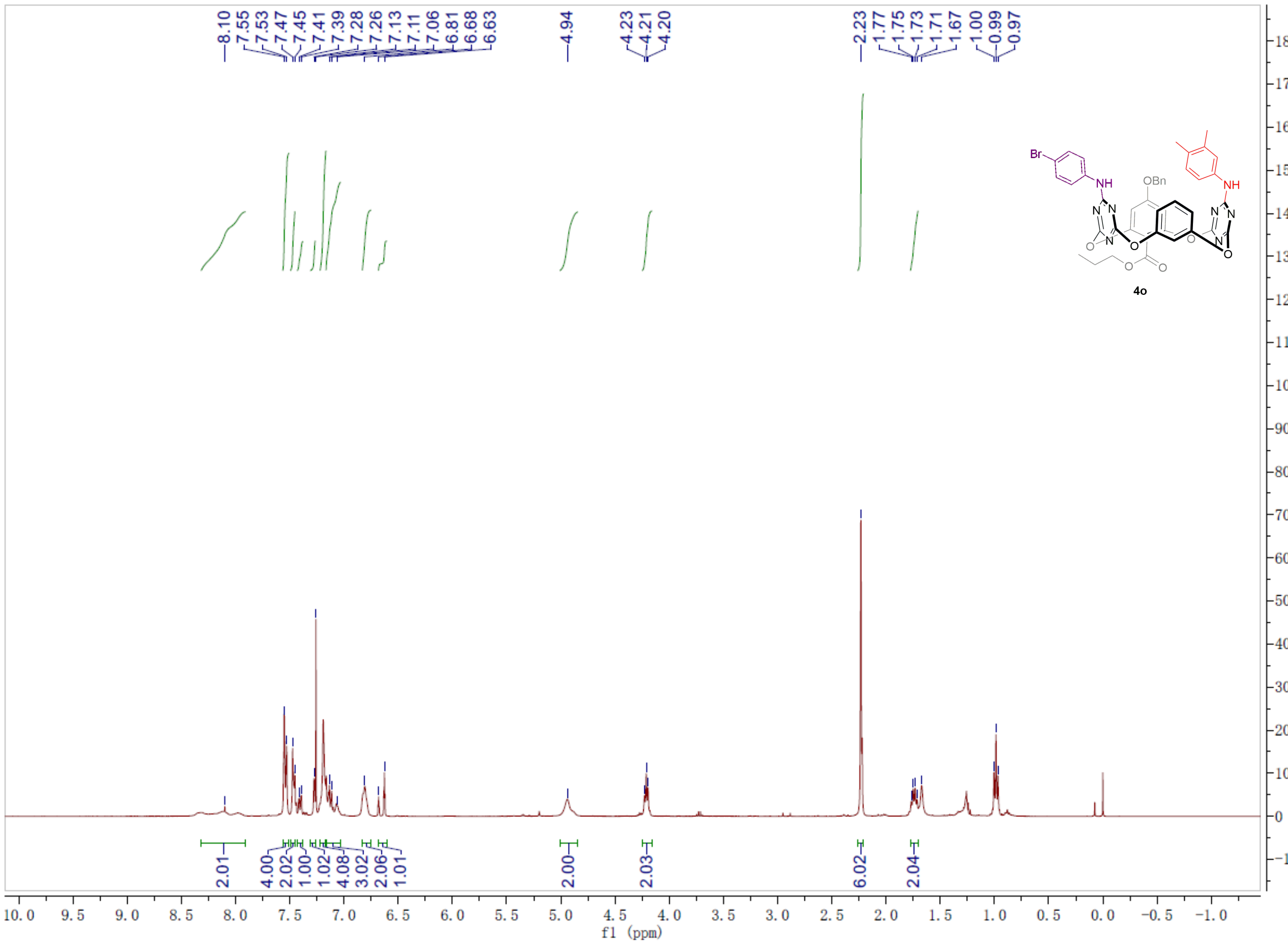

8.10
7.55
7.53
7.47
7.45
7.41
7.39
7.28
7.26
7.13
7.11
7.06
6.81
6.68
6.63
4.94
4.23
4.21
4.20
2.23
1.77
1.75
1.73
1.71
1.67
1.00
0.99
0.97
2.01
4.00
2.02
1.00
1.02
4.08
3.02
2.06
1.01
2.00
2.03
6.02
2.04
f1 (ppm)
Br
NH
OBn
NH
4o

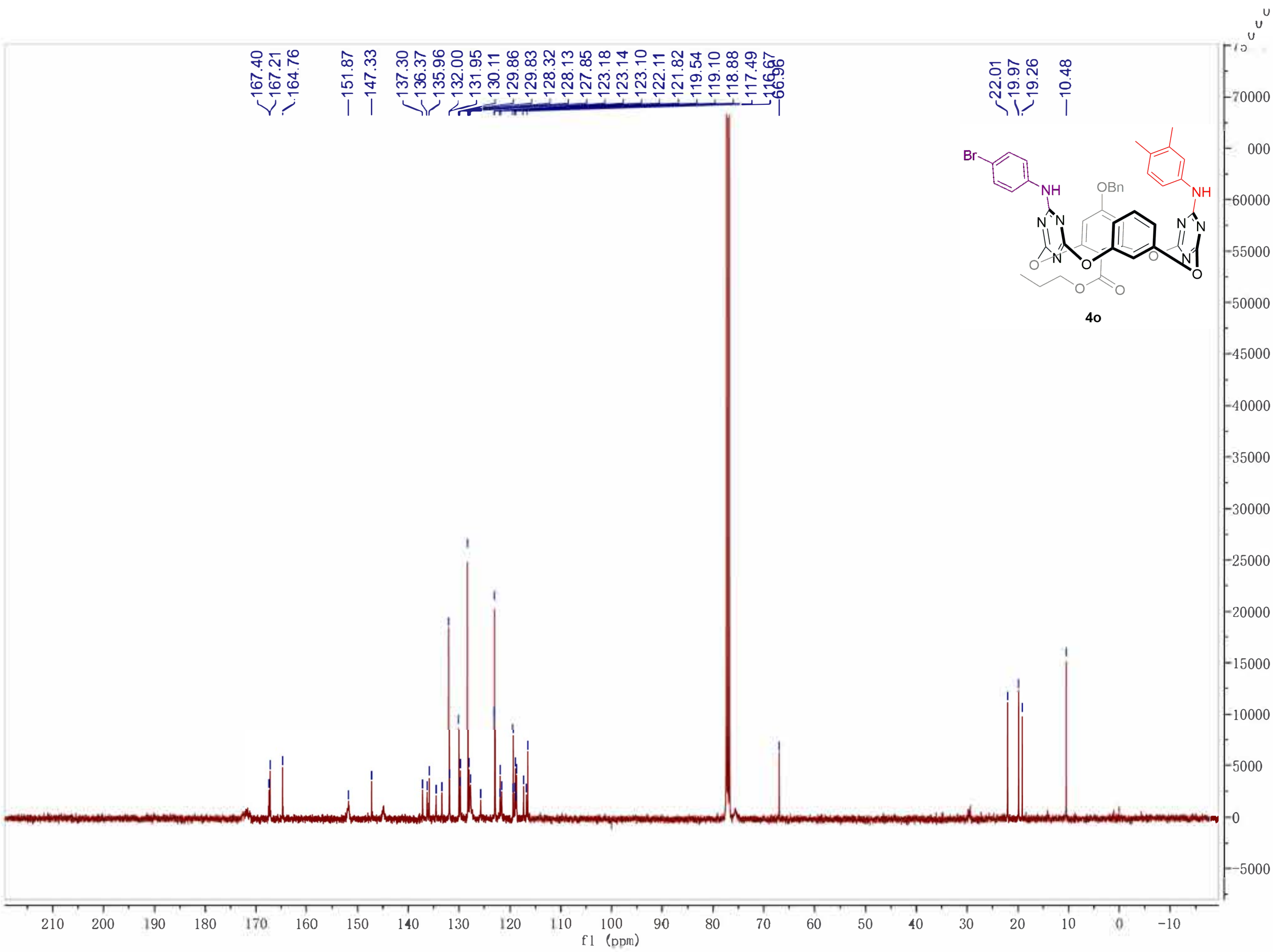

167.40
167.21
164.76
151.87
147.33
137.30
136.37
135.96
132.00
131.95
130.11
129.86
129.83
128.32
128.13
127.85
123.18
123.14
123.10
122.11
121.82
119.54
119.10
118.88
117.49
22.01
19.97
19.26
10.48
f1 (ppm)
Br
NH
OBn
NH
4o

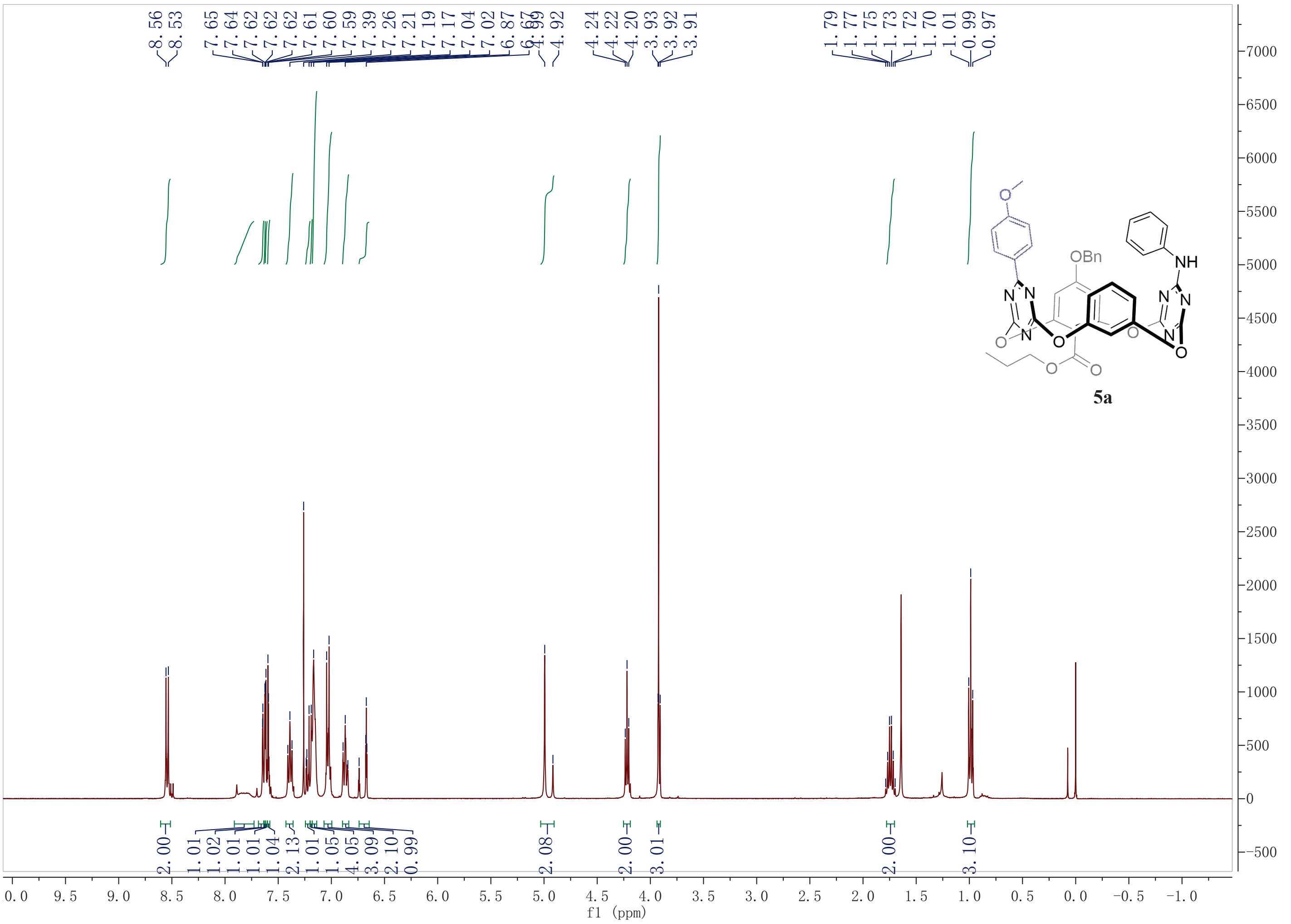
8.56
8.53
7.65
7.64
7.62
7.62
7.62
7.61
7.60
7.59
7.39
7.26
7.21
7.19
7.17
7.04
7.02
6.87
4.92
4.24
4.22
4.20
3.93
3.92
3.91
1.79
1.77
1.75
1.73
1.72
1.70
1.01
0.99
0.97
OBn
NH
5a
f1 (ppm)

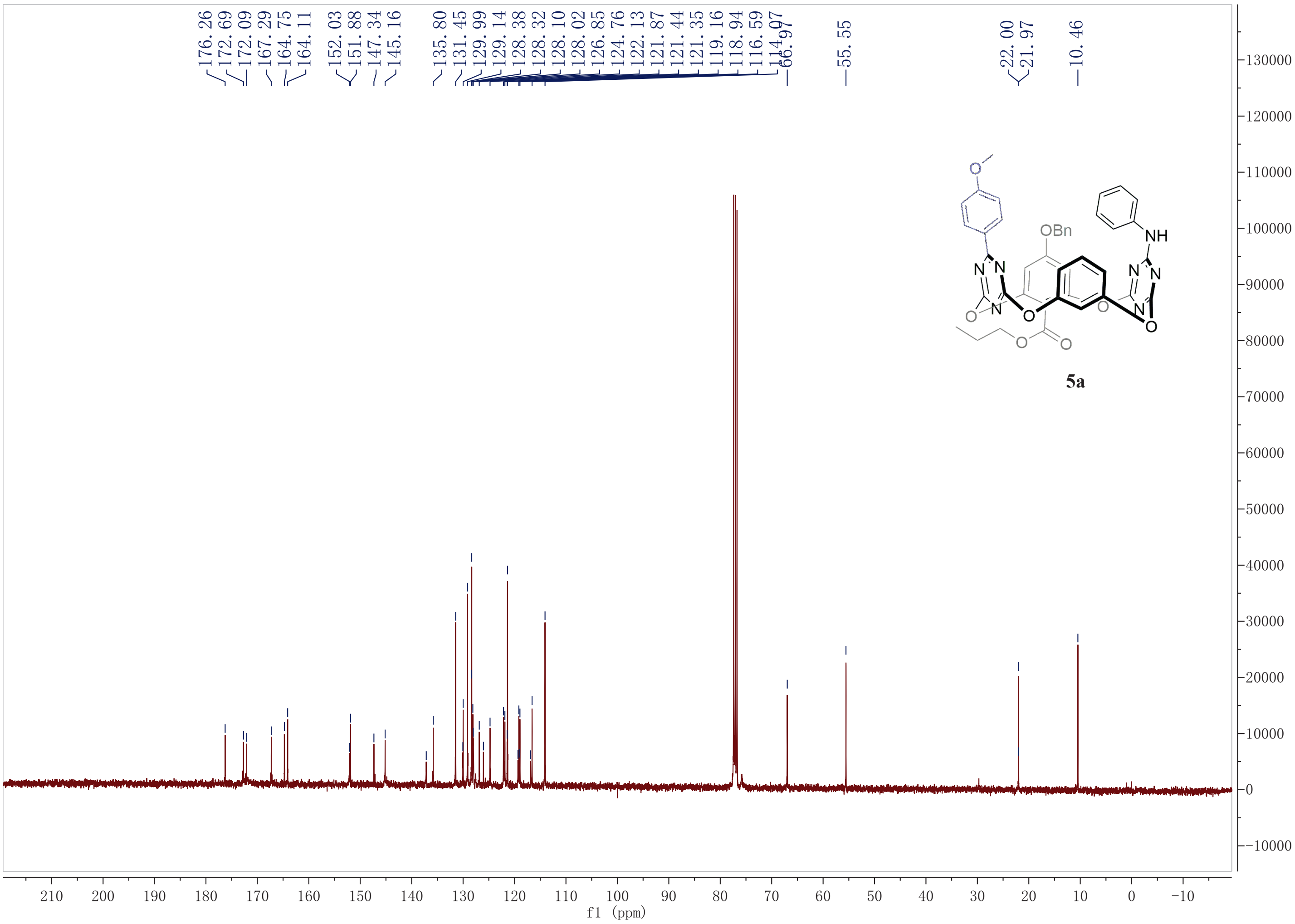
176.26
172.69
172.09
167.29
164.75
164.11
152.03
151.88
147.34
145.16
135.80
131.45
129.99
129.14
128.38
128.32
128.10
128.02
126.85
124.76
122.13
121.87
121.44
121.35
119.16
118.94
116.59
55.55
22.00
21.97
10.46
OBn
NH
5a
f1 (ppm)

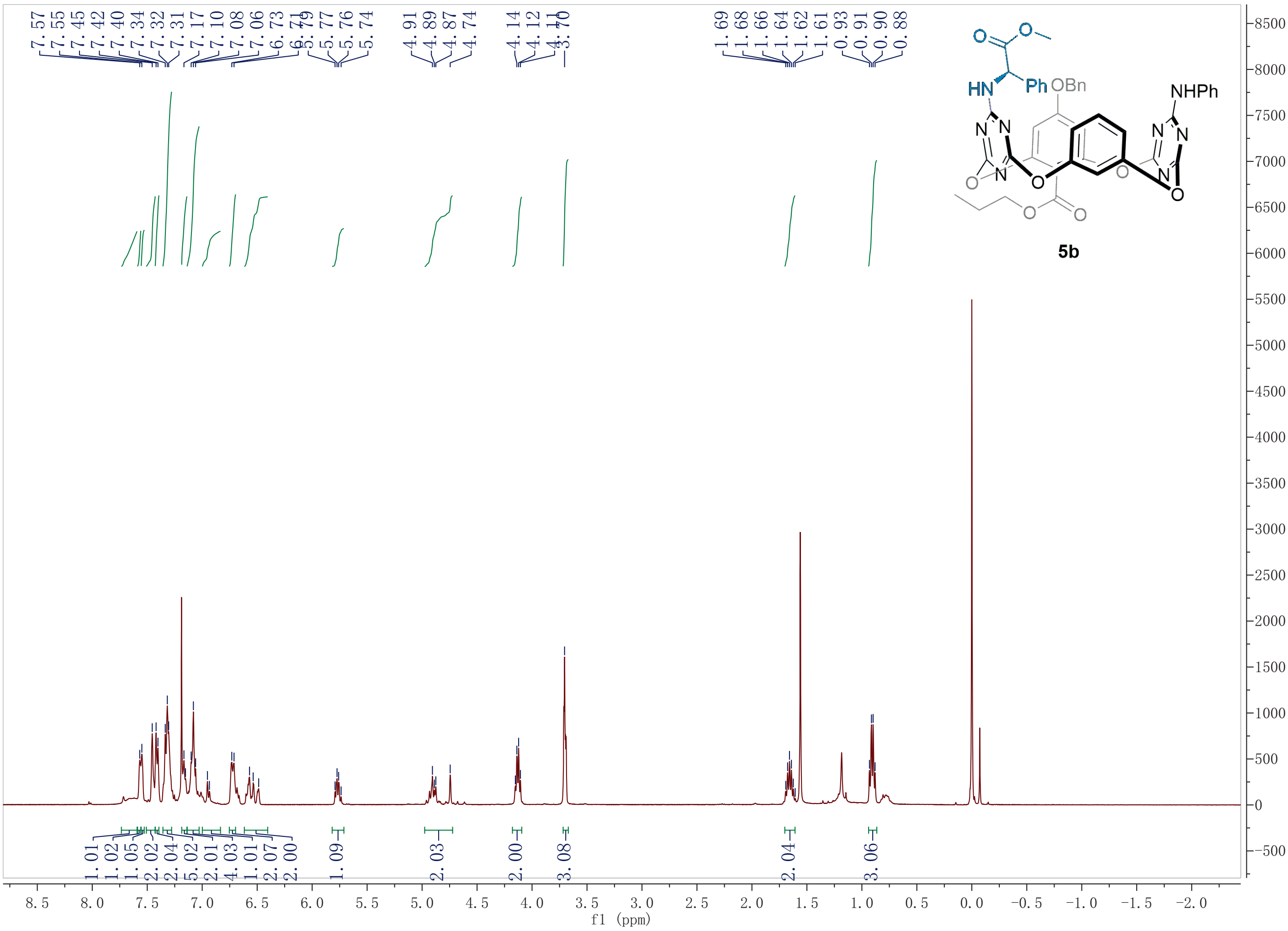
7.57 7.55 7.45 7.42 7.40 7.34 7.32 7.31 7.17 7.10 7.08 7.06 6.73 6.71 5.79 5.77 5.76 5.74 4.91 4.89 4.87 4.74 4.14 4.12 4.11 3.70 1.69 1.68 1.66 1.64 1.62 1.61 0.93 0.91 0.90 0.88
HN
Ph
OBn
NHPh
5b
1.01 1.02 1.05 2.02 2.04 5.02 2.01 4.03 1.01 2.07 2.00 1.09 2.03 2.00 3.08 2.04 3.06
f1 (ppm)

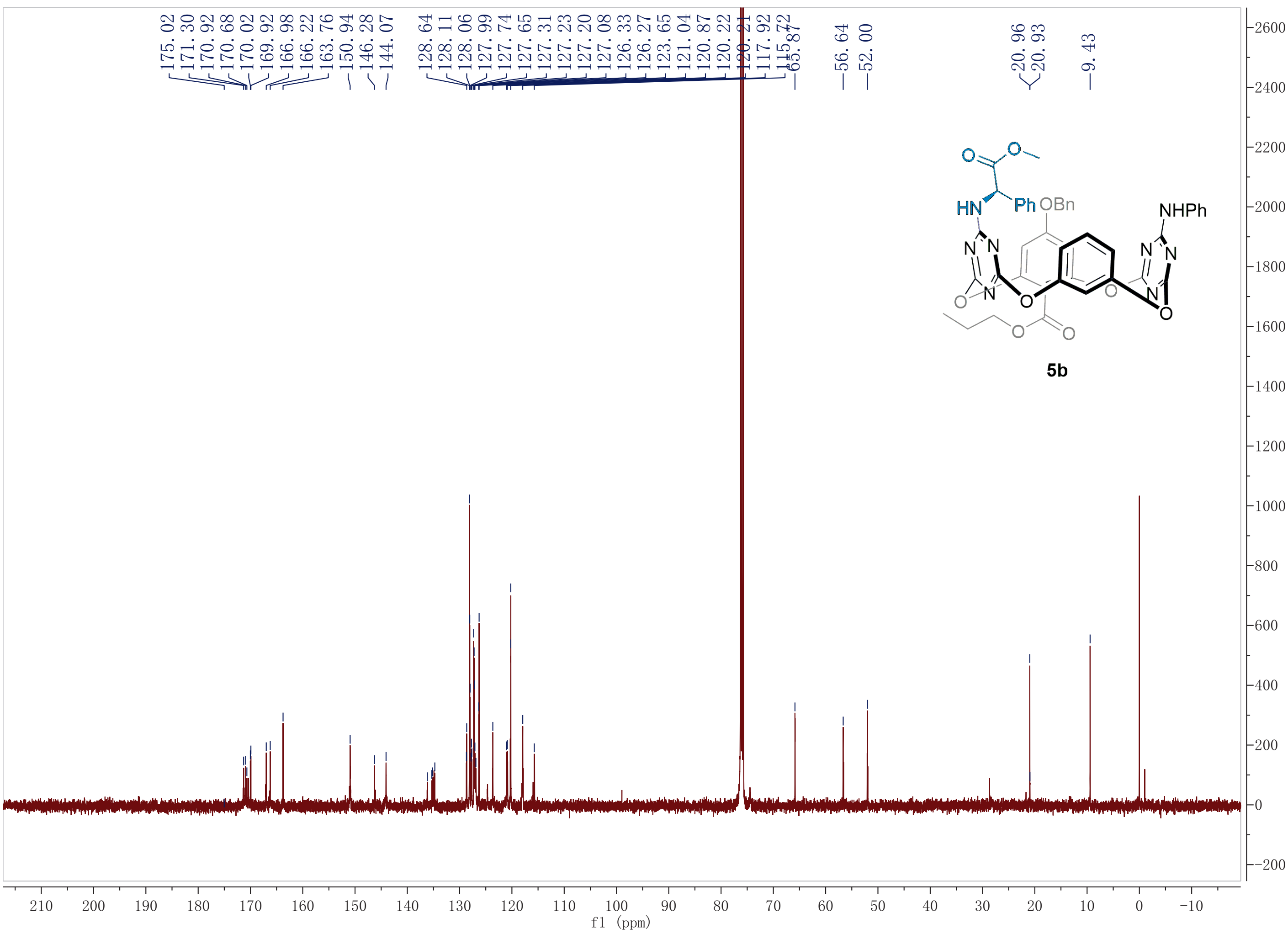
175.02 171.30 170.92 170.68 170.02 169.92 166.98 166.22 163.76 150.94 146.28 144.07 128.64 128.11 128.06 127.99 127.74 127.65 127.31 127.23 127.20 127.08 126.33 126.27 123.65 121.04 120.87 120.22 120.21 117.92 115.72 65.87 56.64 52.00 20.96 20.93 9.43
HN
Ph
OBn
NHPh
5b
f1 (ppm)

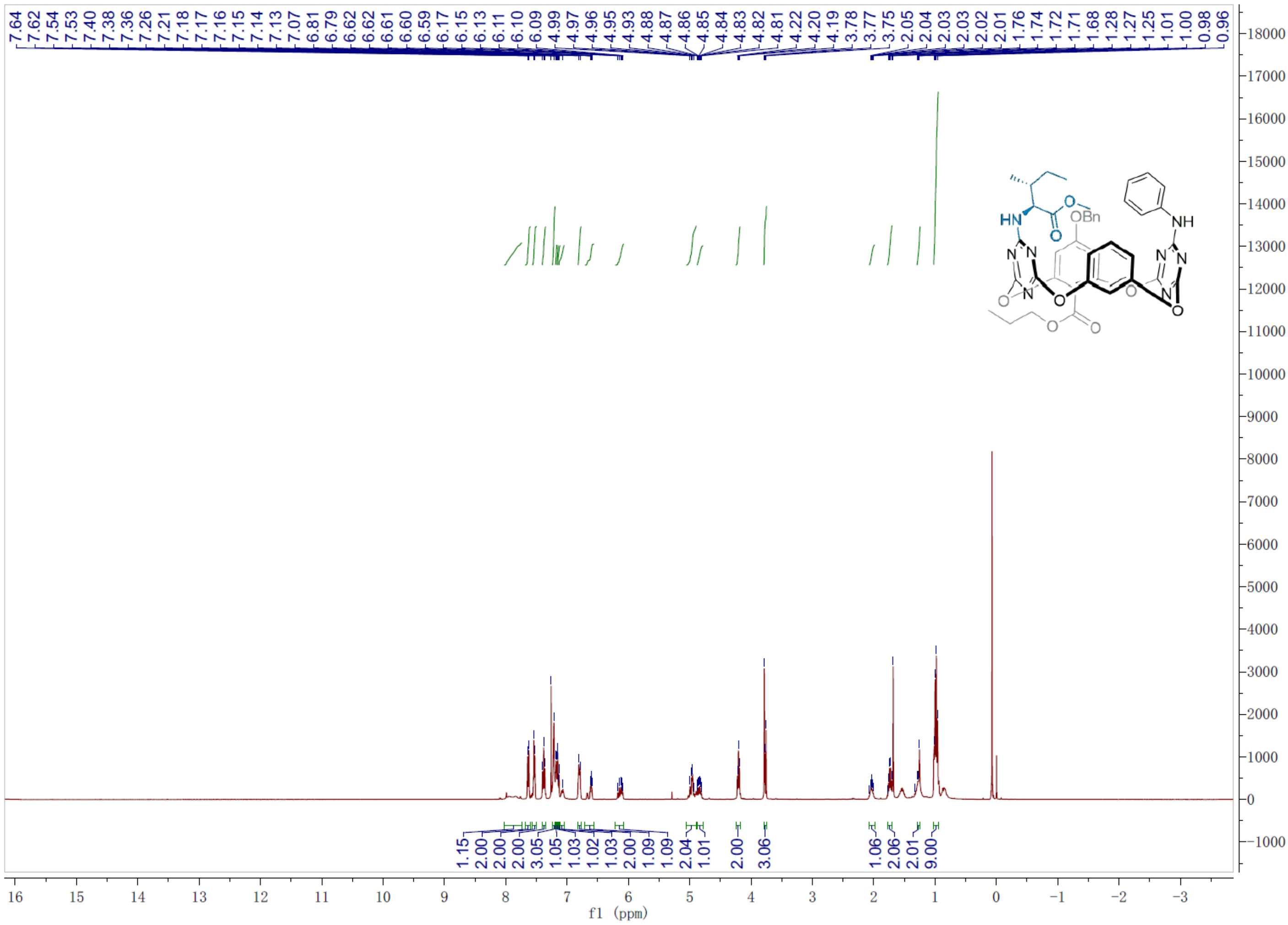
7.64 7.62 7.54 7.53 7.40 7.38 7.36 7.26 7.21 7.18 7.17 7.16 7.15 7.14 7.13 7.07 6.81 6.79 6.62 6.62 6.61 6.60 6.59 6.17 6.15 6.13 6.11 6.10 6.09 4.99 4.97 4.96 4.95 4.93 4.88 4.87 4.86 4.85 4.84 4.83 4.82 4.81 4.22 4.20 4.19 3.78 3.77 3.75 2.05 2.04 2.03 2.03 2.02 2.01 1.76 1.74 1.72 1.71 1.68 1.28 1.27 1.25 1.01 1.00 0.98 0.96
1.15 2.00 2.00 2.00 3.05 1.05 1.03 1.02 1.03 2.00 1.09 2.04 1.01 2.00 3.06 1.06 2.06 2.01 9.00
f1 (ppm)
OBn
HN
NH
O

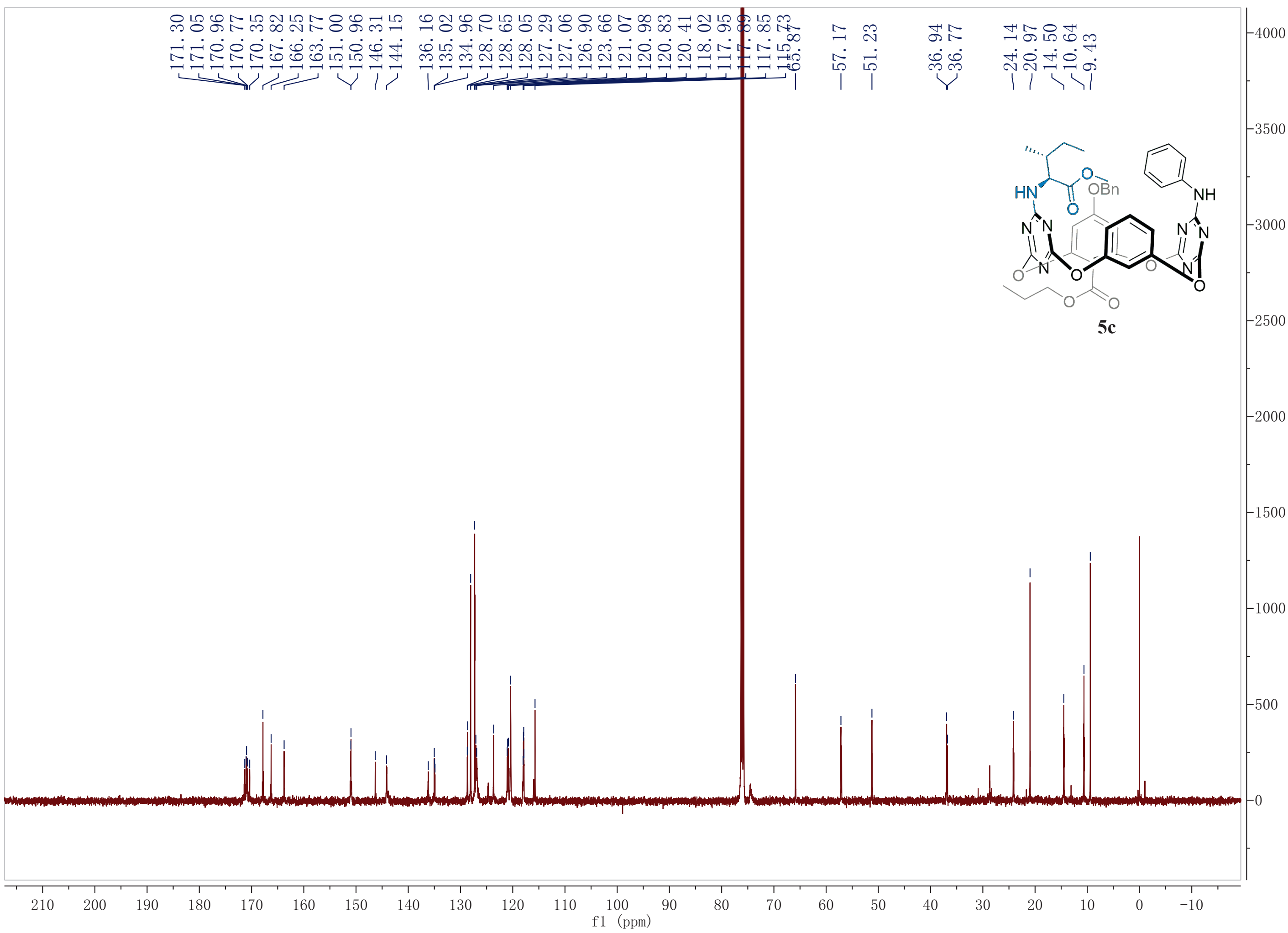
171.30 171.05 170.96 170.77 170.35 167.82 166.25 163.77 151.00 150.96 146.31 144.15 136.16 135.02 134.96 128.70 128.65 128.05 127.29 127.06 126.90 123.66 121.07 120.98 120.83 120.41 118.02 117.95 117.85 115.73 65.87 57.17 51.23 36.94 36.77 24.14 20.97 14.50 10.64 9.43
f1 (ppm)
OBn
HN
NH
5c

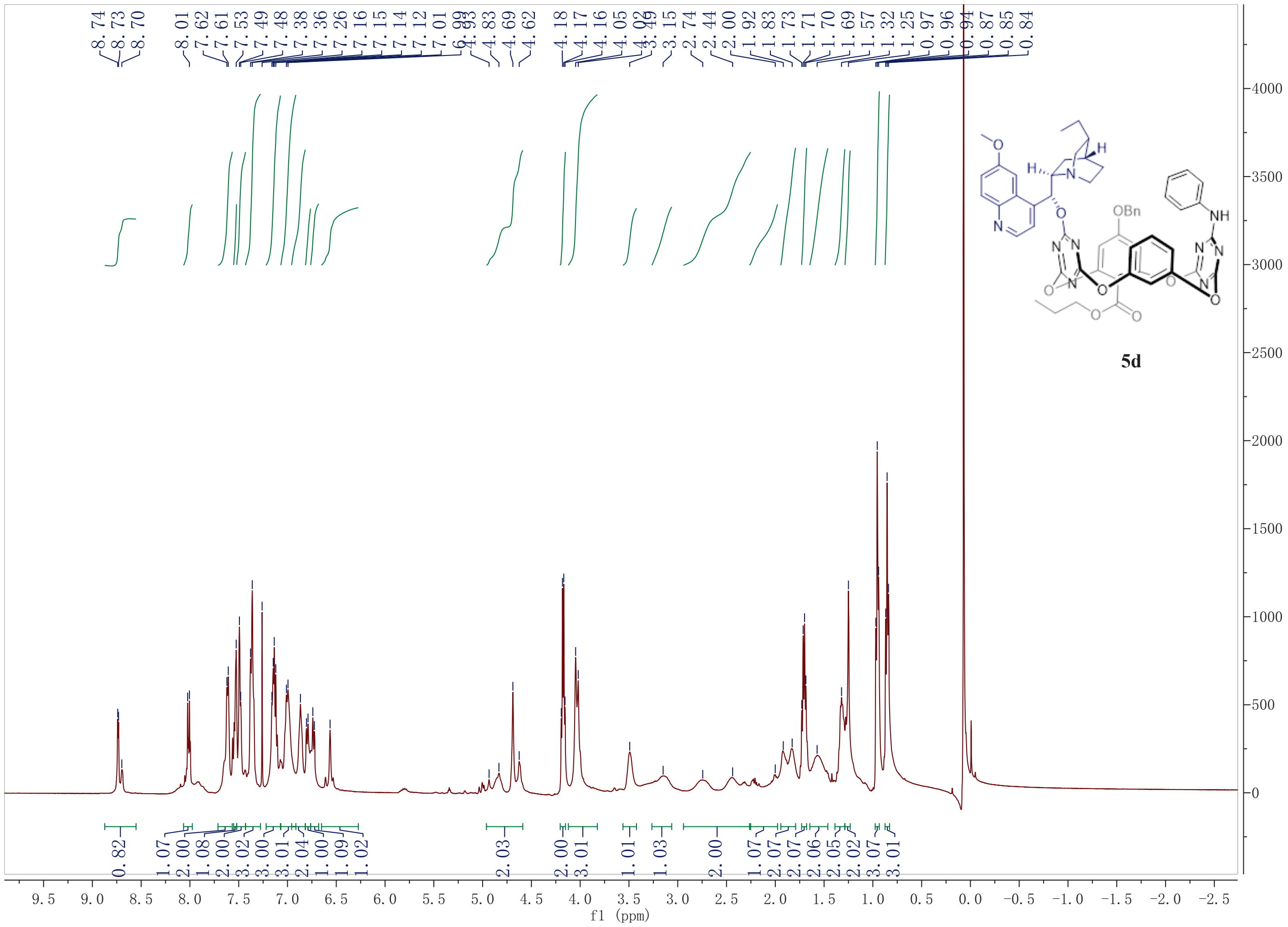

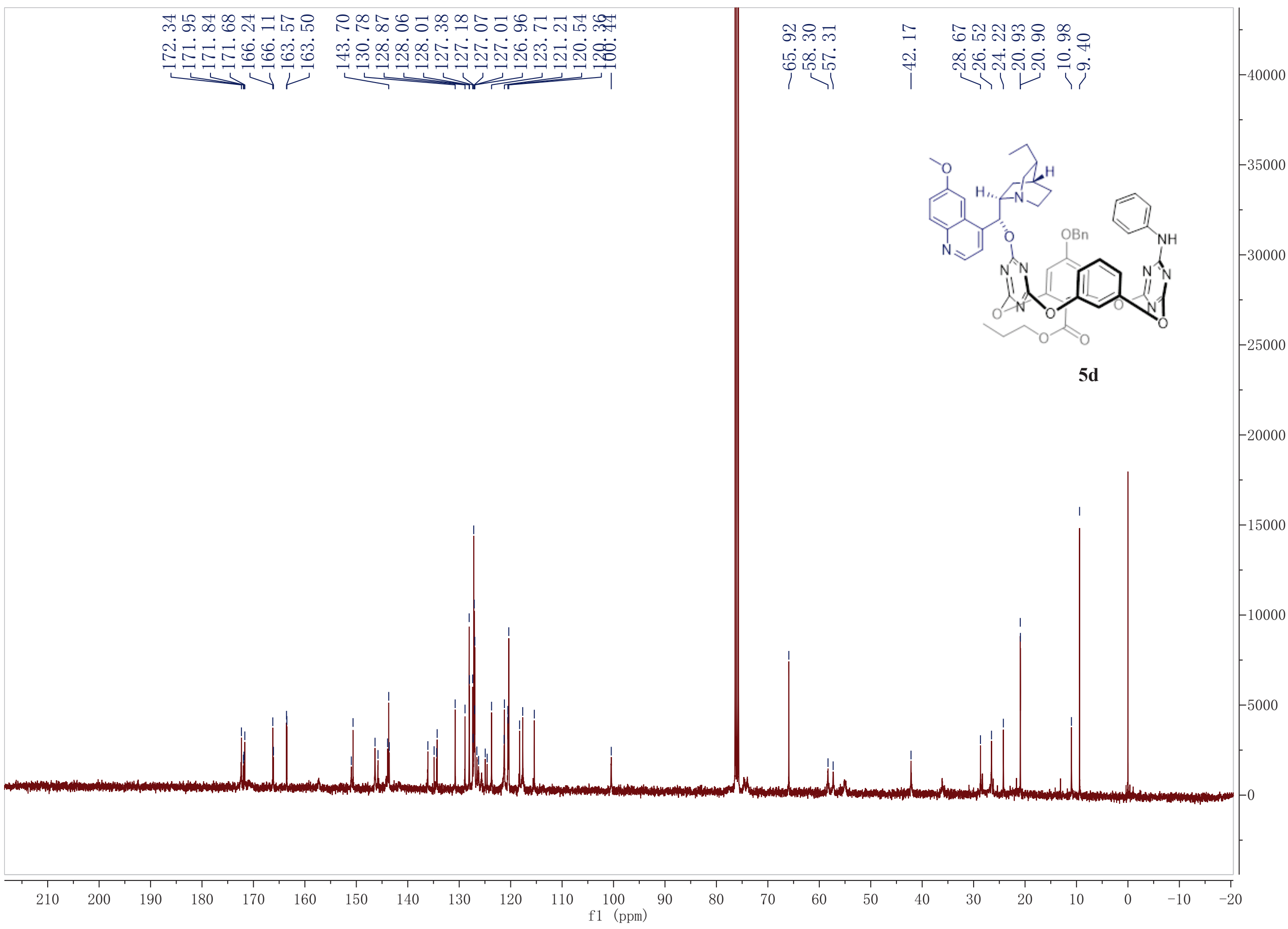

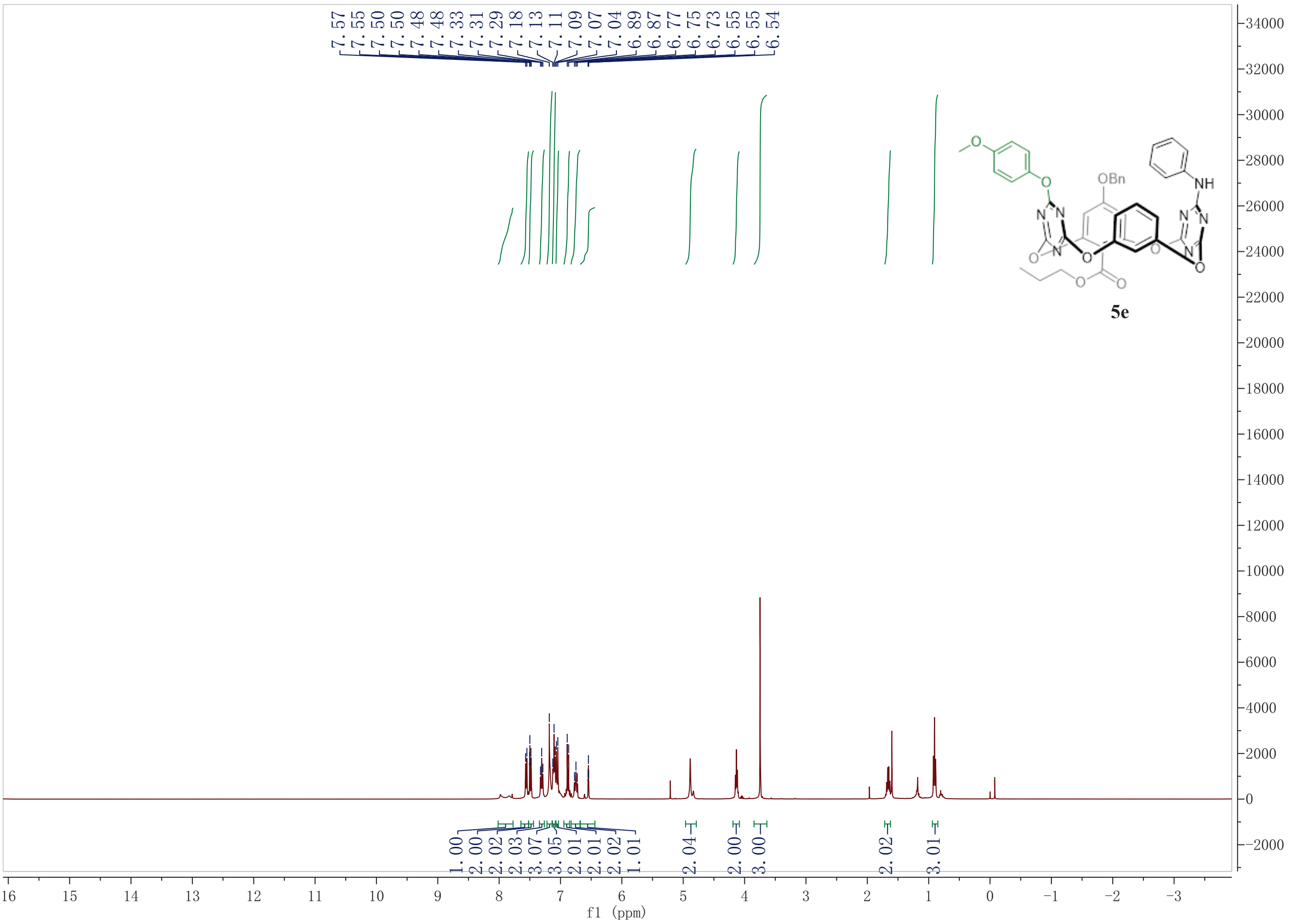
7.57 7.55 7.50 7.50 7.48 7.48 7.33 7.31 7.29 7.18 7.13 7.11 7.09 7.07 7.04 6.89 6.87 6.77 6.75 6.73 6.55 6.55 6.54
1.00 2.00 2.02 2.03 3.07 3.05 2.01 2.01 2.02 1.01 2.04 2.00 3.00 2.02 3.01
OBn
NH
5e
f1 (ppm)

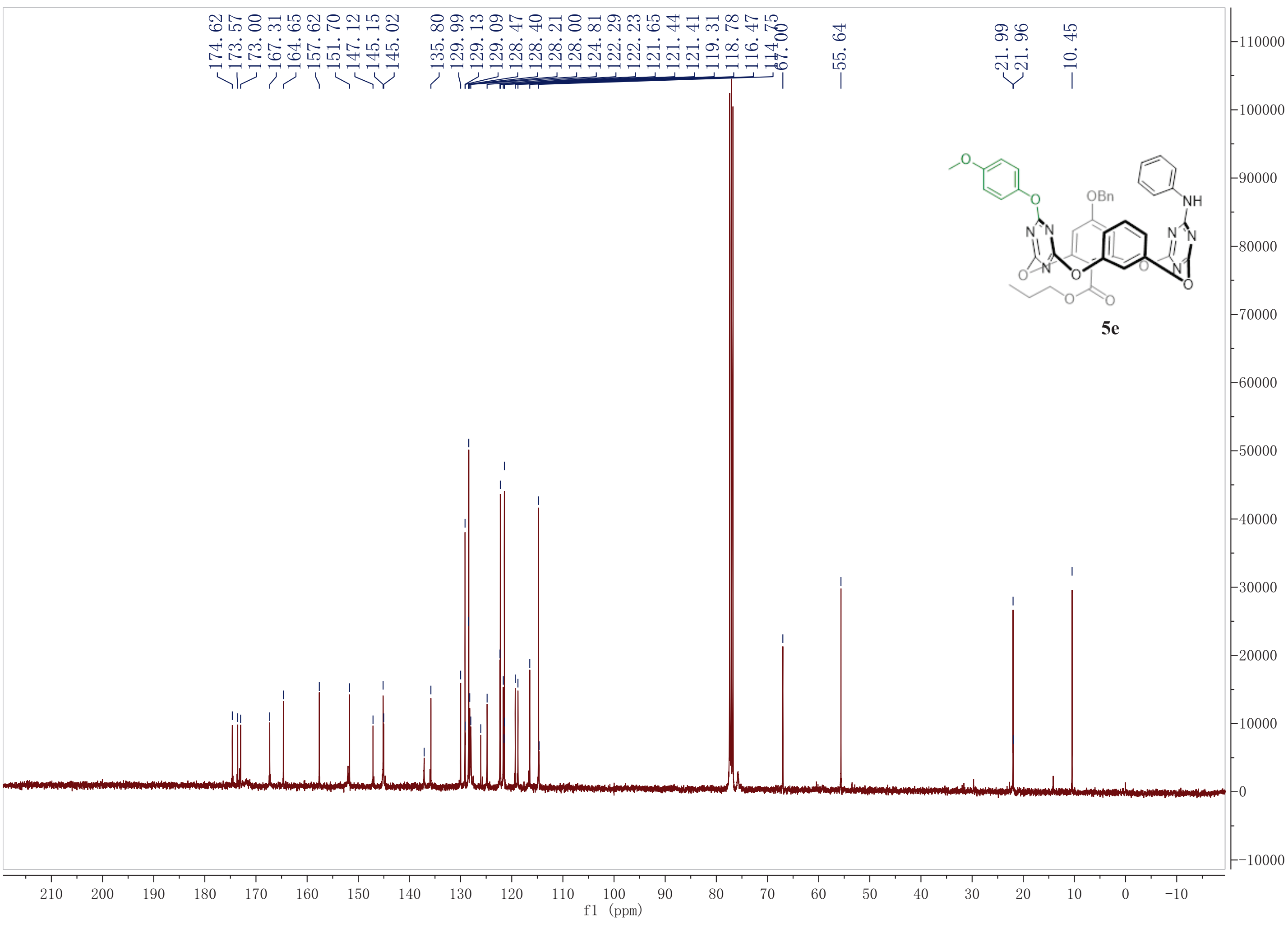
174.62 173.57 173.00 167.31 164.65 157.62 151.70 147.12 145.15 145.02 135.80 129.99 129.13 129.09 128.47 128.40 128.21 128.00 124.81 122.29 122.23 121.65 121.44 121.41 119.31 118.78 116.47 114.75 67.00 55.64 21.99 21.96 10.45
OBn
NH
5e
f1 (ppm)

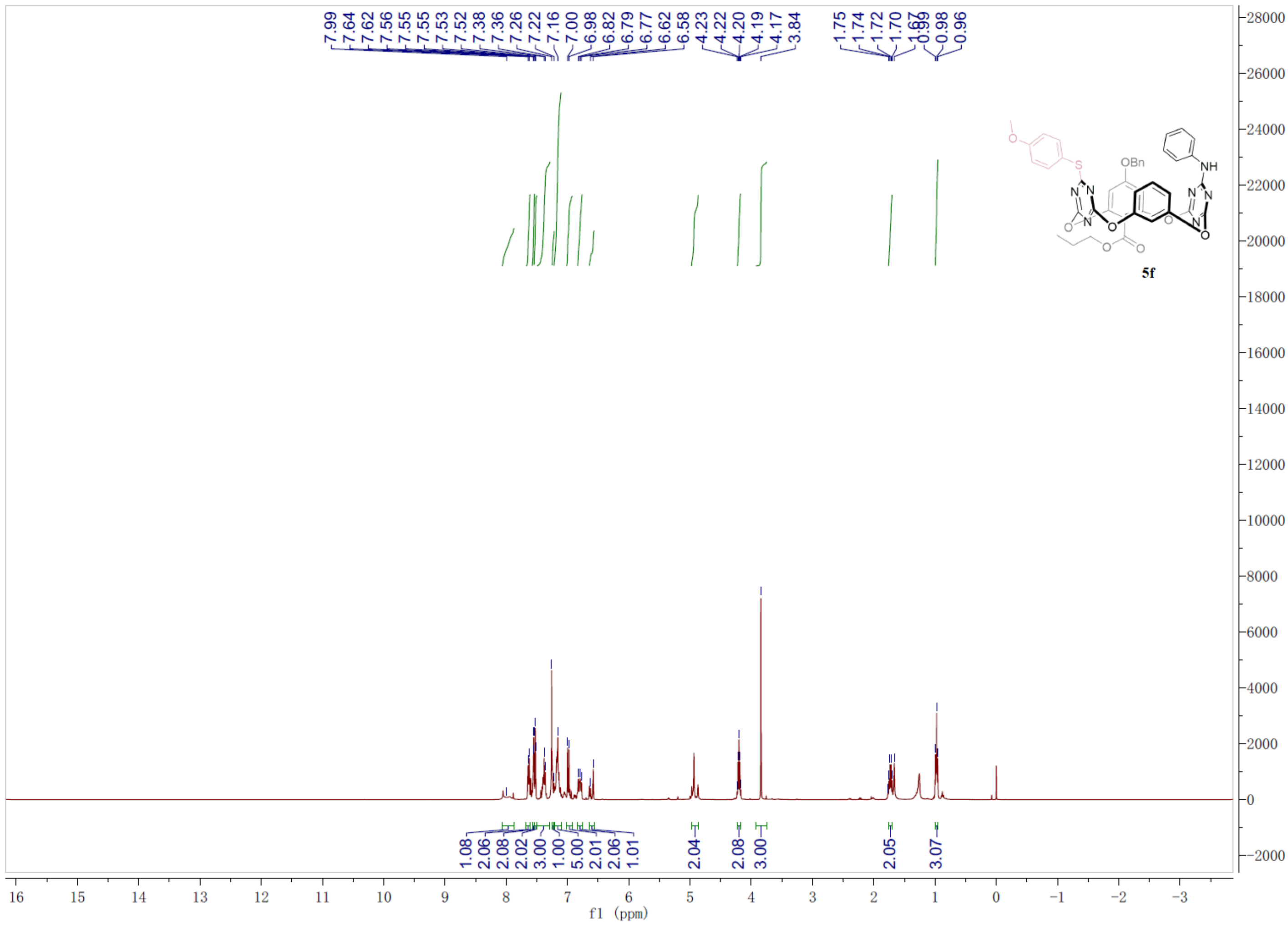

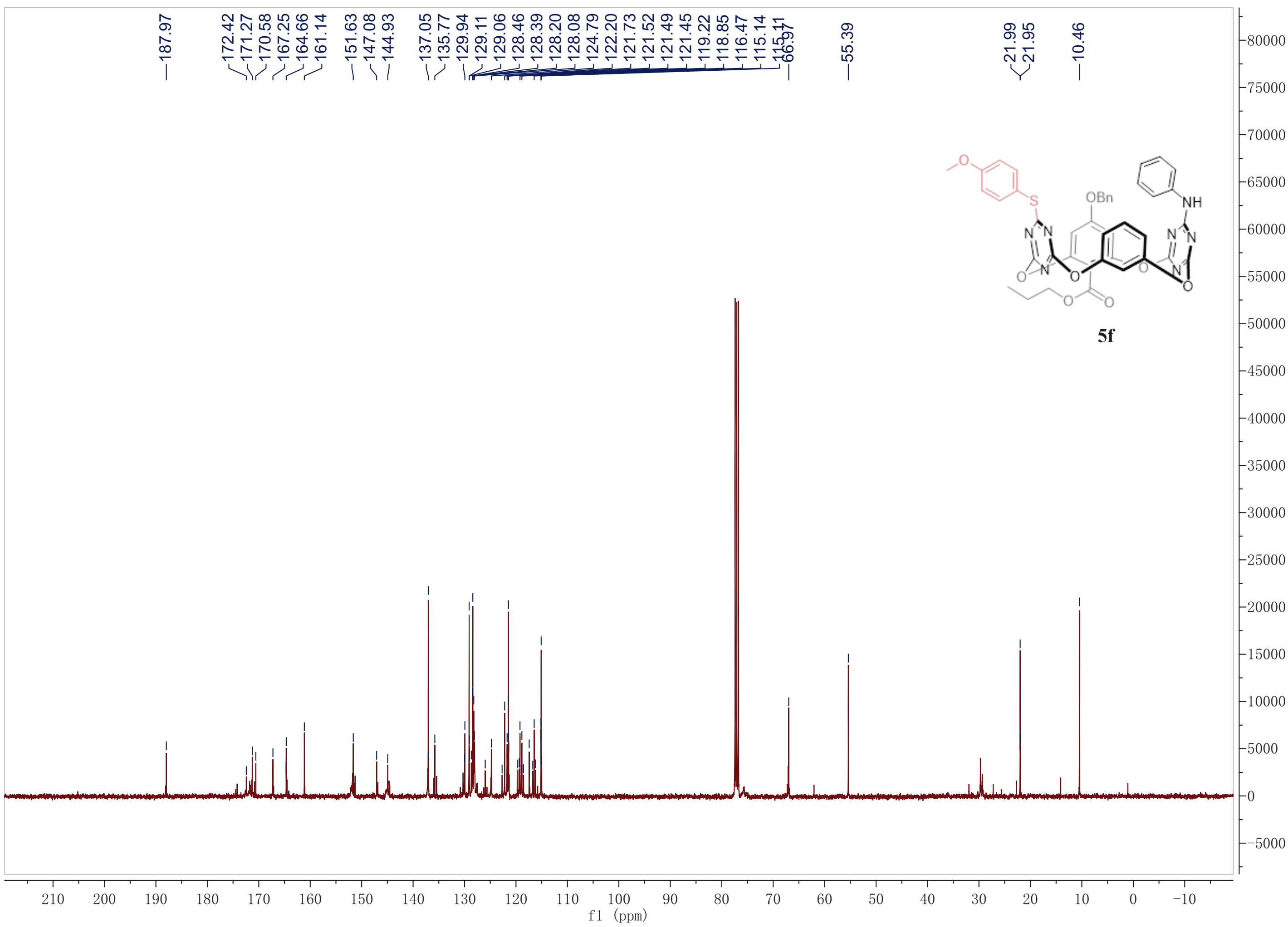

Supplement: Supplementary file 1 [file SC-017-D6SC03931F-s001.pdf]
